# Dynamic Alterations of Site-Specific N-Glycoform and Plasma Proteasome Signatures Stratify Chemotherapy Benefit in Pancreatic Ductal Adenocarcinoma

**DOI:** 10.1016/j.mcpro.2026.101642

**Published:** 2026-08-17

**Authors:** Shanying Gui, Yaqiong Zhang, Tao Xia, Jiazi Qian, Zemin Fang, Wenhui Ouyang, Jie Wang, Jie Chen, Xiaojun Ren, Jianxin Lyu, Wei Cui, Hezhi Fang

**Affiliations:** 1 School of Basic Medical Sciences and Forensic Medicine, Hangzhou Medical College, Hangzhou, Zhejiang, China; 2 Department of Clinical Laboratory, Taizhou Central Hospital (Taizhou University Hospital), Taizhou, Zhejiang, China; 3 Department of Hepatobiliary and Pancreatic Surgery, The Affiliated Lihuili Hospital of Ningbo University, Ningbo, Zhejiang, China; 4 School of Laboratory Medicine and Life sciences, Wenzhou Medical University, Wenzhou, Zhejiang, China; 5 School of Laboratory Medicine and Bioengineering, Hangzhou Medical College, Hangzhou, Zhejiang, China; 6 Department of Clinical Laboratory, State Key Laboratory of Molecular Oncology, National Cancer Center/National Clinical Research Center for Cancer/Cancer Hospital, Chinese Academy of Medical Sciences and Peking Union Medical College, Beijing, China

**Keywords:** pancreatic ductal adenocarcinoma, chemotherapy response, N-glycosylation, site-specific N-glycoforms, plasma proteomics, proteasome, treatment-response biomarkers

## Abstract

Pancreatic ductal adenocarcinoma (PDAC) exhibits profound systemic remodeling during chemotherapy, yet how site-specific intact N-glycosylation in plasma is rewired by treatment remains largely undefined. Here, we perform an integrated plasma multi-omics study combining intact N-glycopeptidomics, deep proteomics, and metabolomics in a longitudinal cohort with paired pre- and post-chemotherapy samples and follow-ups (n = 177). Patients were further stratified according to radiographic treatment response. We identified 17,501 intact N-glycopeptides corresponding to 16,754 glycoforms, together with 6045 proteins and 1080 annotated metabolites. Paired analyses revealed extensive chemotherapy-associated changes across all three omics layers. Temporal glycopeptide and protein patterns were consistently enriched for complement, coagulation, extracellular-matrix, proteasome, and immune-related annotations, while oxidative-phosphorylation–related signatures showed relative depletion after treatment. Exploratory comparisons of within-patient changes (Δ = Post − Pre) between patients with partial response (PR) and those with stable or progressive disease highlighted 140 glycoforms, 18 glycosylation sites, and 103 proteins with response-associated patterns. These included coordinated increases in several proteasome-related plasma proteins in the PR group, as well as site- and glycoform-specific changes in complement- and acute-phase-related proteins. Response-associated differences in circulating metabolites further involved pyrimidine-, amino-acid-, and dipeptide-related features. Finally, leakage-controlled nested cross-validation prioritized 11 recurrent N-glycopeptide candidates and yielded modest prognostic discrimination, with a C-index of 0.591 and a 12-months area under the curve of 0.712. Collectively, this study provides a site- and glycoform-resolved view of chemotherapy-associated plasma molecular changes in PDAC and identifies candidate response- and prognosis-related features for future validation.

## Highlights

• Paired plasma multi-omics maps treatment-associated changes in PDAC.
• Site-specific intact N-glycoforms reveal longitudinal treatment patterns.
• Complement and coagulation annotations recur across two plasma omics layers.
• Proteasome-related proteins show exploratory response-associated trends.
• Eleven pretreatment N-glycoforms yield modest 12-months prognostic performance.

## Graphical Abstract

**Figure:**
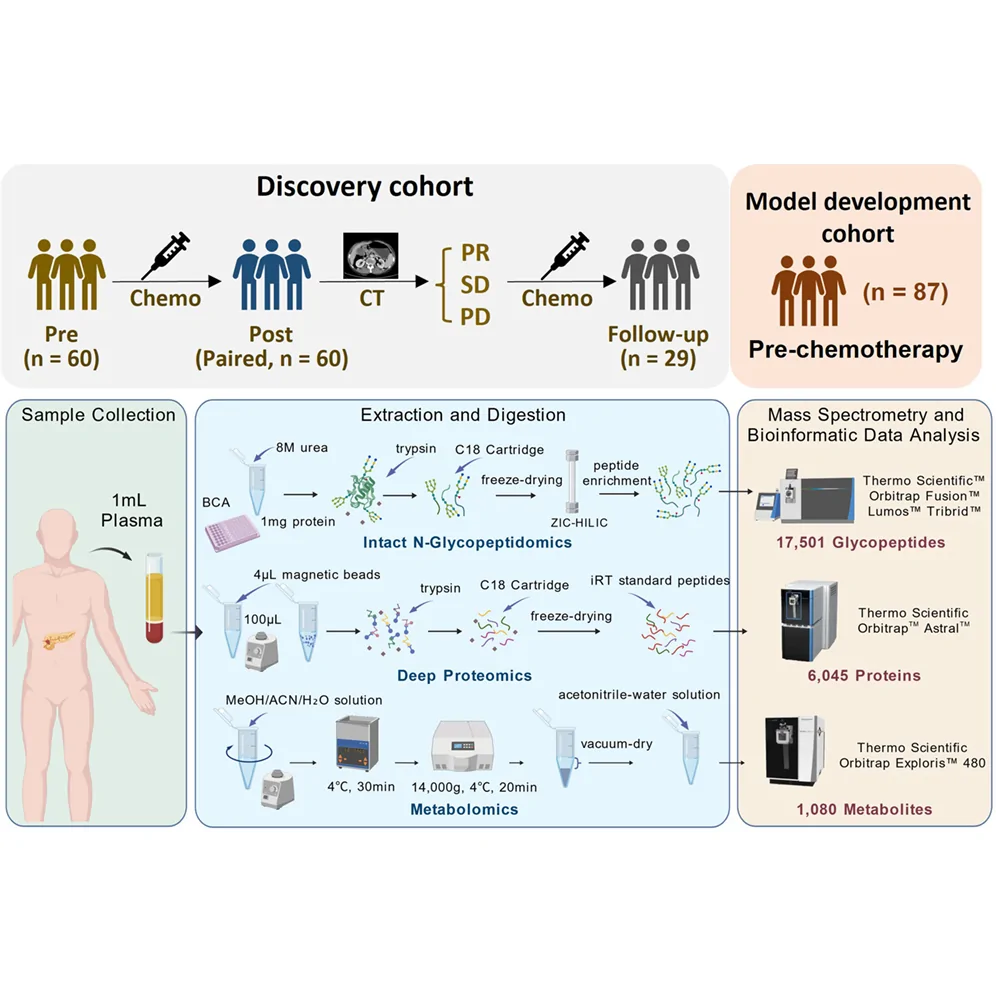

## In Brief

Using paired plasma samples collected before and after chemotherapy, Gui *et al*. integrated intact N-glycopeptidomics, proteomics, and metabolomics to map treatment-associated molecular changes in pancreatic ductal adenocarcinoma. Site-specific N-glycoforms and circulating proteins showed coordinated complement, coagulation, and proteasome-related patterns, including exploratory differences associated with radiographic response. An 11-feature pretreatment N-glycoform panel provided modest short-term prognostic discrimination. This study offers a longitudinal plasma multi-omics resource and candidate response- and prognosis-associated features for independent validation.

Pancreatic ductal adenocarcinoma (PDAC) is among the most lethal malignancies, with a dismal overall 5-years survival rate of only around 10% for all stages (1). Such poor outcomes stem from PDAC’s propensity for late diagnosis, aggressive biology, and resistance to therapy (2). Objective response rates (ORR) to current first-line chemotherapy in PDAC remain modest. In the pivotal metastatic setting, multi-drug regimens such as gemcitabine + nab-paclitaxel (GnP) increased ORR to 23% from 9.4% with gemcitabine monotherapy (3); moreover, FOLFIRINOX achieved an ORR of 31.6% (4), yet most patients still do not experience radiographic responses, and disease progression is common. FOLFIRINOX and GnP have modestly improved the median survival to ∼11 months and ∼8.5 months, respectively, but durable responses are rare (3, 4). Even with the more recent triplet NALIRIFOX regimen in NAPOLI-3, the improvement in overall survival and ORR remains similar to GnP (5). These data indicate that empiric regimen selection is nearing its ceiling, underscoring the need for biomarker-guided patient stratification to match individuals to the most effective chemotherapy.

In current practice, the carbohydrate antigen 19-9 (CA19-9) remains the most widely used plasma biomarker for PDAC (6) (https://pubmed.ncbi.nlm.nih.gov/26530370/), yet its utility is fundamentally constrained. Approximately 5 to 10% of patients are Lewis antigen–negative and therefore incapable of producing CA19-9, and non-malignant biliary obstruction or inflammatory conditions can yield spurious CA19-9 elevations (7). The absence of robust, treatment-informative plasma biomarkers represents a major barrier to response stratification in PDAC.

Recent plasma- and serum-based multi-omics studies in PDAC have begun to establish that such circulating signals are clinically meaningful. Large real-world cohorts using targeted immuno-oncology panels have demonstrated that pretreatment serum samples with baseline level proteins could stratify short or long overall survival, indicating that serum proteome possibly encoded prognostic information rather than diagnostic signals only (8) Longitudinal proteomic and immune profiling has further shown that standard cytotoxic regimens such as FOLFIRINOX can rapidly increase pro-inflammatory cytokines after only the first treatment cycle, and predict early disease progression during chemotherapy (9). Moreover, recent high-depth serum proteomics in chemotherapy-containing regimens has similarly identified metabolic and inflammatory protein signatures associated with durable clinical benefit, reinforcing the concept that systemic host–tumor biology is quantifiable in blood (10). By contrast, glycoproteomic analyses in PDAC have so far focused predominantly on cross-sectional case–control discrimination. Aberrant protein glycosylation and altered metabolomics are hallmarks of cancer that can fundamentally alter cell signaling, immune recognition, and metastasis potential (11, 12, 13). Multiple studies have defined recurrent PDAC-associated glycosylation features (including hyper-sialylated and hyper-fucosylated N-glycans) and quantified glycopeptides with diagnostic performance (https://www.sciencedirect.com/science/article/pii/S1535947623001986?via%3Dihub). However, the dynamics of site-specific glycosylation in PDAC remain essentially uncharacterized. In particular, treatment-induced remodeling of glycoforms has not been systematically mapped onto objective chemotherapy response or clinical outcome. As a result, there is currently no validated circulating glycosylation signature that captures therapy-driven molecular reprogramming or predicts chemotherapy sensitivity in PDAC.

Here we present a comprehensive plasma multi-omics study of chemotherapy response in PDAC, combining in-depth proteomics, intact N-glycopeptide profiling, and metabolomic analyses, with a discovery cohort of 60 patients with PDAC from whom paired plasma samples were collected at baseline (pre-treatment) and after completion of first-line chemotherapy (post-treatment), along with follow-up timepoints. A total of 17,501 intact N-glycopeptides, 6045 plasma proteins, and 1080 metabolite features were quantified, constituting one of the most comprehensive multi-omics atlases of chemotherapy-associated plasma remodeling in PDAC to date. We leveraged paired pre- and post-treatment samples to model within-patient molecular changes and examined their relationships with radiographic response categories (partial response versus stable/progressive disease). This analysis revealed pronounced, response-dependent reprogramming of the circulating glycoproteome and metabolome following chemotherapy. Collectively, these findings expand the molecular framework for understanding systemic treatment responses in PDAC and establish a resource to support future development of clinically actionable circulating signatures.

## Experimental procedures

### Sex as a Biological Variable

This study included both male and female participants, and the overall findings were similar in both sexes.

### Experimental Design and Statistical Rationale

Human plasma samples were collected from PDAC patients treated at Zhejiang Provincial People’s Hospital, Hangzhou Medical College. All human studies reported in this manuscript were conducted in accordance with the principles of the Declaration of Helsinki and were approved by the Ethics Committee of Zhejiang Provincial People’s Hospital (approval No. 2022QT010).

A total of 87 patients were enrolled between January 2022 and March 2024. The discovery cohort comprised 60 patients who received standard first-line GnP (n = 53) or FOLFIRINOX (n = 7) chemotherapy; patient-level details are provided in Supplementary Data 1. Plasma samples were collected longitudinally at baseline before chemotherapy (Pre; n = 60), at the first treatment-response assessment (Post; n = 60), and at subsequent follow-up assessments (Post2-Post6; n = 29) when available. Sample scheduling, the interval from baseline to the first post-treatment sampling and the intervals between consecutive follow-up samples, was listed in Supplementary Data 1. Treatment response was assessed radiographically and classified as partial response (PR), stable disease (SD), or progressive disease (PD) according to Response Evaluation Criteria in Solid Tumors (RECIST), version 1.1. For prognostic modeling, an independent set of pre-treatment plasma samples (n = 28) was additionally included. Morning fasting peripheral blood samples were centrifuged to isolate plasma, which was aliquoted and stored at −80 °C until analysis.

Each biological plasma sample was processed and analyzed once for each omics layer. For the peptide-based mass spectrometry analyses, pooled quality-control (QC) samples were prepared by combining aliquots from the study samples and were injected throughout each analytical batch to monitor instrument stability and analytical reproducibility. A pooled QC sample was analyzed after every 10 study-sample injections; these QC injections were technical replicates of the same pooled peptide preparation. Quantified features were retained for downstream analysis only after the layer-specific QC and missingness filters described below had been applied.

The sample size was determined by the number of eligible patients with available paired plasma samples for multi-omics analysis. No formal power calculation was performed because of the exploratory nature of the study. Paired analyses were used to evaluate molecular changes from before to after treatment. Response-associated features were evaluated by first calculating the within-patient change (Δ = Post − Pre) and then comparing these changes between the PR and nonresponsive (N) groups. Pretreatment samples were used for prognostic modeling, which was evaluated using a leakage-controlled nested cross-validation framework. Multiple-testing correction was performed using the Benjamini–Hochberg procedure where indicated, and all statistical tests were two-sided unless stated otherwise.

### Intact N-glycopeptidomics

Protein concentrations in plasma samples were determined using a BCA protein assay kit. For each sample, 1 mg of protein extract was transferred to a new microcentrifuge tube, and the final volume was adjusted to 200 μl with 8 M urea. Proteins were reduced by adding 20 μl of 0.5 M TCEP and incubating at 37 °C for 1 h. Alkylation was then performed by adding 40 μl of 1 M iodoacetamide, followed by incubation for 40 min at room temperature in the dark.

Proteins were precipitated by adding pre-chilled acetone at a sample-to-acetone volume ratio of 1:5 and incubating the mixture overnight at −20 °C. The precipitated proteins were collected by centrifugation at 12,000×*g* for 20 min at 4 °C, and the supernatant was discarded. The pellet was washed with 1 ml of pre-chilled 90% acetone, vortexed thoroughly, and centrifuged again at 12,000×*g* for 20 min at 4 °C and repeated twice. Th pellet was dissolved in 1 ml of 100 mM triethylammonium bicarbonate (TEAB) after acetone evaporated. Trypsin was added at an enzyme-to-protein mass ratio of 1:50, and digestion was performed overnight at 37 °C. The resulting peptides were desalted using C18 cartridges, quantified using the Pierce 23,275 peptide assay kit, and lyophilized for subsequent enrichment.

For glycopeptide enrichment, a HILIC microcolumn was prepared using ZIC-HILIC (Merck Millipore, Merck KGaA, Darmstadt, Germany) loaded with a C8 disk. Lyophilized peptides were dissolved in 80% acetonitrile (ACN) with 1% trifluoroacetic acid (TFA) and repeatedly loaded onto the microcolumn. The microcolumn was then washed with 80% ACN with 1% TFA, and sequentially eluted with 0.1% TFA, 25 mM ammonium bicarbonate (NH_4_HCO_3_), and 50% ACN. The enriched glycopeptide samples were reconstituted in 10 μl of solvent A (0.1% formic acid in water) and analyzed by nanoLC–MS/MS. Chromatographic separation was performed using an EASY-nano-LC 1200 system coupled to an Orbitrap Fusion Lumos Tribrid mass spectrometer (Thermo Fisher Scientific, MA, USA). A 5 μl aliquot of each sample was loaded onto a C18 analytical column, and separated at a flow rate of 250 nl/min using the following gradient: 2% Mobile phase B (0.1% formic acid in acetonitrile) for 3 min, increased to 80% B over 114 min, increased to 95% B within 1 min, and maintained at 95% B for 5 min. The electrospray voltage was set to 2 kV.

The mass spectrometer was operated in data-dependent acquisition (DDA) mode with automatic switching between MS and MS/MS acquisition. Full MS scans were acquired over an m/z range of 400 to 2000 at a resolution of 120,000, with an automatic gain control (AGC) target of 800,000 and a maximum injection time of 100 ms. Dynamic exclusion was set to one occurrence with an exclusion duration of 30 s. MS/MS spectra were acquired in the Orbitrap using higher-energy collisional dissociation (HCD) at a resolution of 15,000, with an isolation window of 4 m/z, an AGC target of 200,000, a maximum injection time of 22 ms, and a normalized collision energy of 30%. Stepped collision energy was enabled with an offset of ±10%.

Raw MS/MS data were searched using pGlyco 3.0 (pFind Studio) against the 2024 UniProt *Homo sapiens* protein database (20,608 entries) and the pGlyco-N human glycan database. Trypsin was specified as the proteolytic enzyme, and up to two missed cleavages were allowed. Precursor- and fragment-ion mass tolerances were set to 10 and 20 ppm, respectively. Carbamidomethylation of cysteine (+57.02 Da) was specified as a fixed modification, and oxidation of methionine (+15.99 Da) was specified as a variable modification. Glycopeptide identifications were filtered at a total false discovery rate (FDR) of 1%. Label-free quantification was performed using pGlyQuant (pFind Studio).

For intact N-glycopeptidomic preprocessing, quantitative results generated by pGlyQuant were filtered before downstream analysis. Features with missing values in more than 70% of all samples were excluded. For retained features, missing values were imputed using the k-nearest neighbors (KNN) method. Sample-level median normalization was followed by log10 transformation and standardization to generate the processed matrix used in downstream analyses (Supplementary Data 2). Differentially abundant intact N-glycopeptides for the pre-versus post-treatment comparison were defined using thresholds of |log2 fold change (FC)| > 1 and FDR <0.01 (Supplementary Data 3).

### Plasma Proteomics

Mass spectrometric data acquisition for plasma proteomics was performed using a Thermo Scientific Vanquish Neo ultra-high-performance liquid chromatography (UHPLC) system coupled to an Orbitrap Astral mass spectrometer equipped with a Thermo Scientific Easy-Spray electrospray ionization source. Peptide separation was performed on an ES906 analytical column (1.9 μm particle size). Mobile phase A consisted of 0.1% formic acid in water, and mobile phase B consisted of 0.1% formic acid in 80% acetonitrile. Peptides were separated by gradient elution before MS analysis.

For MS acquisition, full MS1 scans were acquired over an m/z range of 380 to 980 at a resolution of 240,000 at m/z 200. The normalized AGC target was set to 500%, and the maximum injection time was 5 ms. MS2 spectra were acquired in data-independent acquisition (DIA) mode using 299 isolation windows, each with a width of 2 m/z. High-energy collision dissociation was performed with a collision energy of 25 eV. For MS2 acquisition, the normalized AGC target was set to 500%, and the maximum injection time was 3 ms.

DIA raw files were processed using DIA-NN version 1.8.1 in library-free mode against the Swiss-Prot *H. sapiens* protein database downloaded on February 7, 2024 (20,434 entries; swissprot_Homo_sapiens_20434_20240207). Trypsin specificity was applied, with one missed cleavage allowed. Carbamidomethylation of cysteine was specified as a fixed modification, whereas methionine oxidation and protein N-terminal acetylation were specified as variable modifications. DIA-NN performed internal mass-accuracy calibration and optimization; therefore, fixed user-defined precursor- and fragment-ion mass tolerances were not specified. Protein identifications were controlled at an FDR of <1%.

For downstream proteomic analysis, proteins with missing values in more than 50% of samples were excluded. For the remaining proteins, missing values were imputed using the KNN method. The resulting protein abundance matrix was then subjected to median normalization, log10-transformed and standardized for further analyses (Supplementary Data 4). Paired pre- and post-treatment differences were assessed using paired two-tailed t-tests. Differentially expressed proteins (DEPs) were defined as proteins with |log2FC| ≥ 0.585 and *p* < 0.05; Benjamini–Hochberg-adjusted *p* values are also reported to allow evaluation after multiple-testing correction (Supplementary Data 5).

### Untargeted Plasma Metabolomics

Polar metabolites were extracted from plasma using methanol/acetonitrile/water and analyzed by UHPLC–MS/MS on a Vanquish LC system coupled to an Orbitrap Exploris 480 mass spectrometer (Thermo Fisher Scientific) in positive- and negative-ionization modes. QC samples prepared by pooling aliquots from study samples were interspersed throughout the analytical run to monitor instrument stability. Raw files were converted to mzXML format and processed using XCMS for peak detection, alignment, and retention-time correction, followed by metabolite annotation and downstream statistical analysis (Supplementary Data 6). Following the same process as protein paired comparison, metabolites met the differential-abundance criteria (|log2FC| > 0.4 and FDR <0.1) after treatment are reported in Supplementary Data 7.

### Bioinformatic and Statistical Analysis

Unless otherwise specified, all statistical tests were two-sided. Continuous variables were compared using Student’s *t* test or the Wilcoxon rank-sum test, as appropriate, and categorical variables were compared using the chi-square test or Fisher’s exact test. For paired pre-versus post-treatment omics analyses, differential abundance was assessed using the fold-change and significance thresholds specified for each omics layer. Within-patient changes were calculated as Δ = Post − Pre and compared between the PR and N groups. *p* values were adjusted for multiple testing using the Benjamini–Hochberg procedure where indicated. Functional enrichment analyses were performed using Kyoto Encyclopedia of Genes and Genomes (KEGG) and Molecular Signatures Database (MSigDB) gene sets implemented in clusterProfiler.

To identify outcome-associated molecular features while minimizing information leakage, prognostic modeling was performed within a nested cross-validation framework. Feature selection was restricted to the training partition of each outer fold, followed by elastic-net penalized Cox regression with hyperparameter tuning in the inner cross-validation loop. Model performance was evaluated using out-of-fold predictions, Harrell’s concordance index (C-index), and time-dependent receiver operating characteristic curves.

## Results

### Patients, Sample Collection, and Study Design

**Fig. 1:**
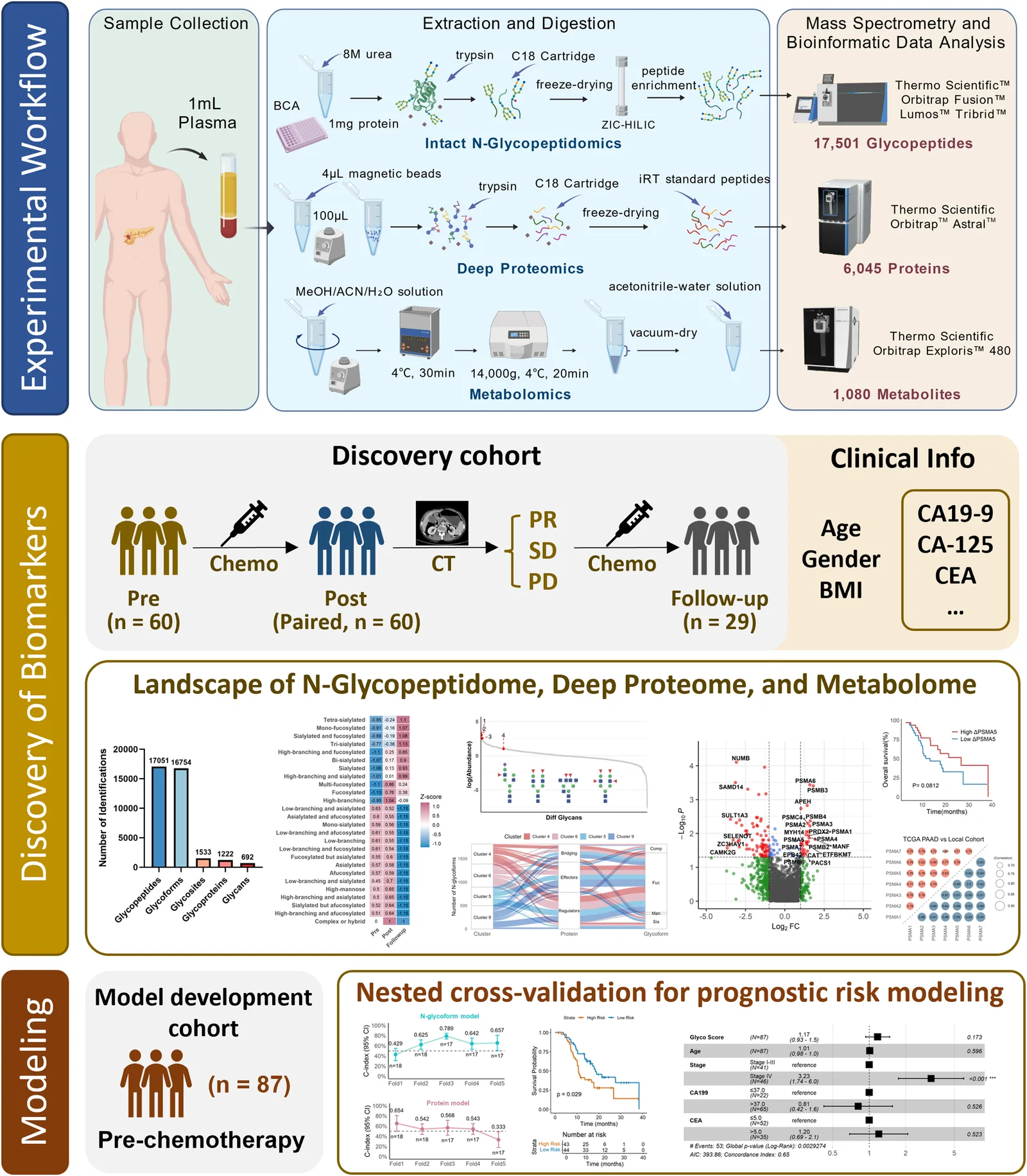
**Study design and multi-omics workflow.** 177 plasma samples from 87 pancreatic ductal adenocarcinoma (PDAC) patients were collected before and after chemotherapy. Alterations in the plasma N-glycoform, proteome, metabolome between chemosensitive and chemoresistant patients may reveal underlying mechanisms of chemosensitivity and potential biomarkers for PDAC. In the discovery cohort of 60 patients, paired samples were obtained before (Pre, n = 60) and after the first round of chemotherapy (Post, n = 60), as well as after subsequent rounds (Follow-up samples with 15 patients, n = 29). Another 27 patients were recruited for prognostic risk modeling. Multiomics data from these samples were integrated to construct a machine-learning based model to predict chemosensitivity, which was then evaluated in the independent validation cohort. Figure created with BioGDP.com.

The study workflow is summarized in Figure 1. The discovery cohort comprised 60 patients with PDAC who received GnP or FOLFIRINOX, with plasma collected at baseline (Pre; n = 60) and at the first treatment-response assessment (Post; n = 60), a median of 78 days after baseline (interquartile range [IQR], 65–97 days). 15 patients contributed 29 additional longitudinal samples, with a median interval of 70 days between consecutive collections (IQR, 34–151 days). Pretreatment samples from an additional 27 patients were included for prognostic modeling, yielding 87 patients overall. Samples were collected and aliquoted according to standardized procedures for parallel omics analyses.

**Fig. 2:**
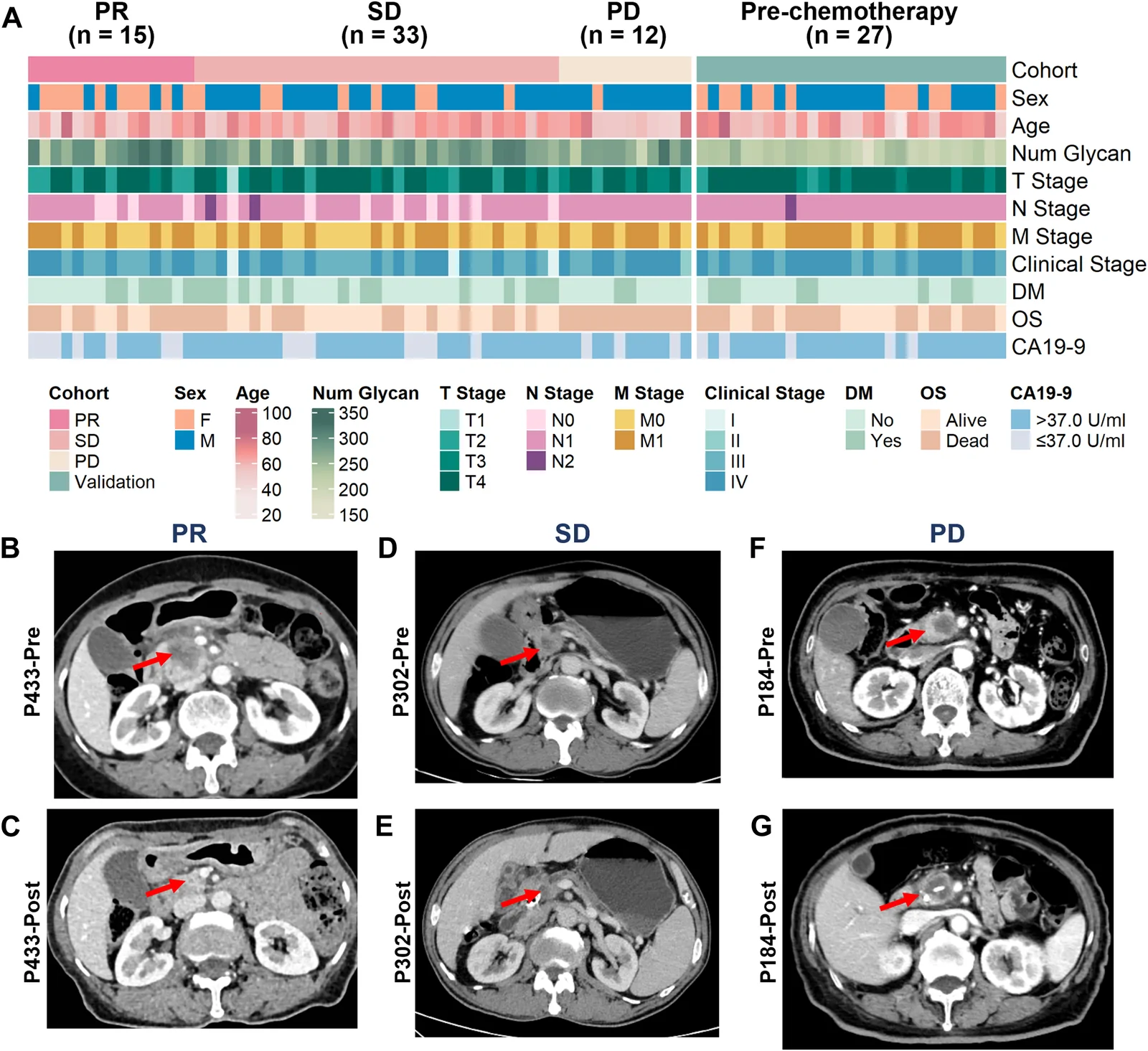
**Clinical characteristics and radiographic response assessment.***A*, clinical annotation heatmap for the 87 PDAC patients included in this study, including the discovery cohort stratified by radiographic response and the independent pre-chemotherapy cohort used for prognostic modeling. Response categories were defined according to RECIST 1.1 as partial response (PR), stable disease (SD), or progressive disease (PD). Clinical variables include sex, age, number of quantified glycans per patient, T stage, N stage, M stage, clinical stage, diabetes mellitus status (DM), overall survival status (OS), and baseline CA19-9 status. *B–G*, representative axial computed tomography images from patients with PR (*B*-*C*), SD (*D*-*E*), and PD (*F*-*G*) before and after one cycle of chemotherapy. *Red arrows* were added indicating the tumor location.

Clinical characteristics are shown in Figure 2 and Supplementary Data 1. Radiographic responses were classified according to RECIST version 1.1 as partial response (PR), stable disease (SD), or progressive disease (PD). Only radiographic response was significantly associated with overall survival, while no independent associations were observed for sex, age, chemotherapy regimen, or liver metastasis (Supplementary Fig. S1). Subsequent analyses evaluated treatment-associated molecular changes and whether these within-patient changes differed across response groups.

We performed plasma N-glycopeptidome (site-specific intact N-glycopeptides), in-depth proteome, and metabolomics profiling to characterize molecular patterns associated with chemotherapy and treatment response. Compared with the previous single analyzer instrument, we achieved a significantly greater depth of N-glycopeptidome and proteome coverage, identifying a total of 17,501 glycopeptides and 6045 proteins in plasma (Fig. 1). Method robustness of the three omics was confirmed by repeated measurements of pooled QC samples (Supplementary Figs. S2–S4).

### Landscape of Intact N-glycopeptides in PDAC Plasma

**Fig. 3:**
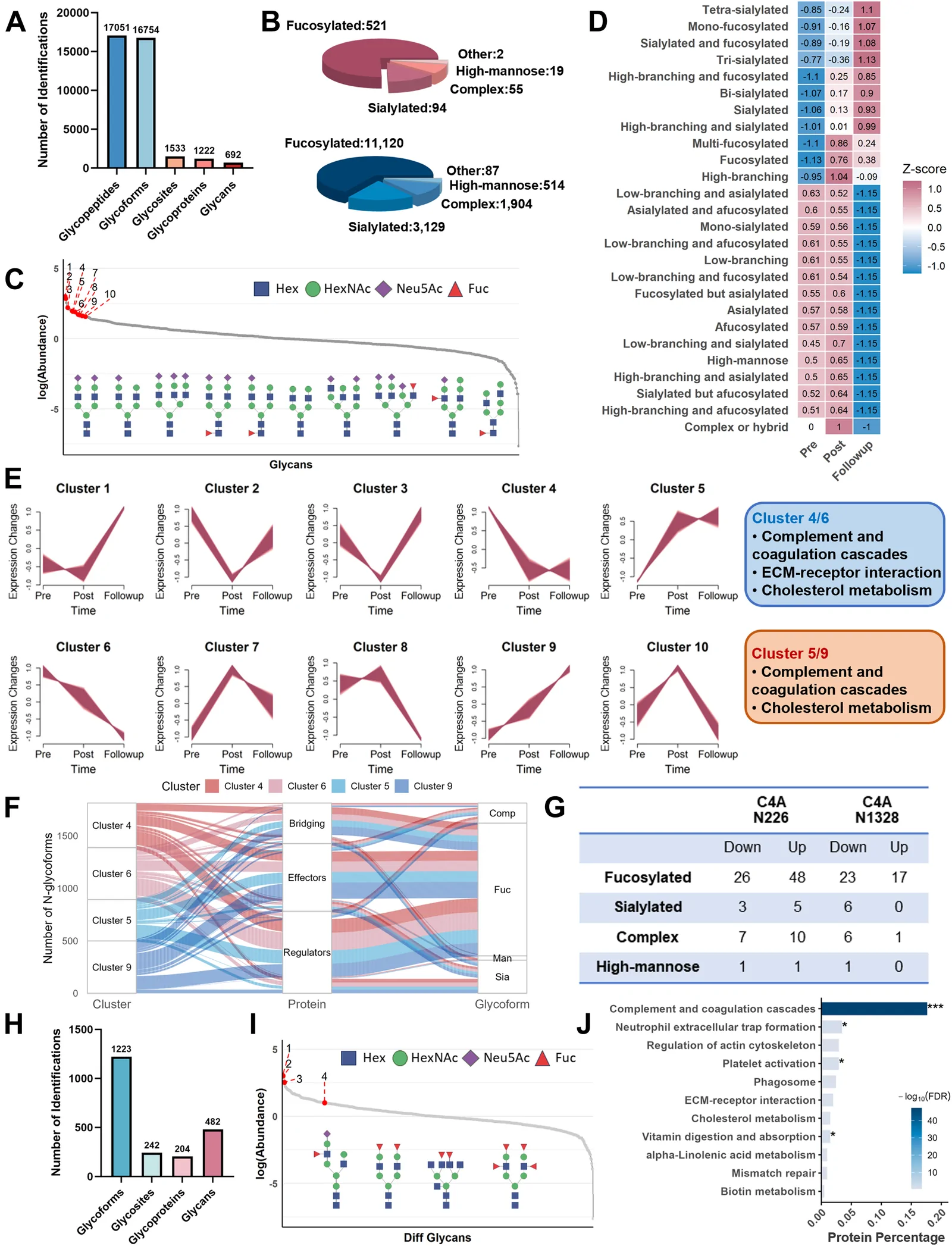
**Intact N-glycopeptide-based glycoproteomic profiling of plasma before and after chemotherapy.***A*, Total numbers of unique glycopeptides, glycoforms, glycosylation sites (glycosites), glycoproteins, and glycans identified in all samples. *B*, Pie charts showing the proportions of glycan types among identified glycans (*red*) and glycoforms (*blue*). *C*, structures of the top 10 identified glycans by intensity (abundance). *D*, heatmap showing the expression of various glycan types across the three sample groups (Pre, Post, Followup); *E*, soft clustering of glycoprotein expression in time-series via Mfuzz; *F*, Sankey diagram demonstrating the number of N-glycoforms of different types identified in plasma complement proteins from Cluster 4/6/5/9 (as defined in *panel E*), with glycoform types including fucose (Fuc), sialic acid (Sia), mannose (Man), and complex (Comp). *G*, Glycan types that are up- or down-regulated at two glycosites (N226 and N1328) of the complement protein C4A; *H*, total number of differentially expressed glycoforms, glycosites, glycoproteins, and glycans between Pre and Post samples; *I*, structures of the top four differentially expressed glycans; *J*, Kyoto Encyclopedia of Genes and Genomes (KEGG) pathways enriched among the differentially expressed glycoproteins. *Blue square*: hexose (Hex); green circle: N-Acetylhexosamine (HexNAc); *purple diamond*: N-Acetylneuraminic acid (Neu5Ac); *red triangle*: fucose (Fuc).

Across all samples, 17,501 intact N-glycopeptides were identified, comprising 16,754 glycoforms and 692 distinct N-glycans (Fig. 3*A*, Supplementary Fig. S5). These glycopeptides mapped to 1222 glycoproteins and 1533 unique N-glycosylation sites at a total FDR of 1.0%. Over 60% of glycosites were first-time discovered due to the great depth of coverage (Supplementary Fig. S6*A*). Approximately 85% of glycoproteins harbored one detected glycosylation site, and 58% of sites carried one detected glycan species (Supplementary Fig. S6, *B*–*D*). Fucosylated structures accounted for 521 of 692 glycans and 11,120 of 16,754 glycoforms, and fucosylated or sialylated structures were prominent among the most abundant glycans (Fig. 3, *B* and *C*).

To minimize potential confounding factors, only patients treated with the GnP regimen were included for further analysis. Plasma glycans with similar structures were first aggregated into 26 structural categories (14). Highly branched sialylated and/or fucosylated categories tended to increase over time, whereas less-branched, asialylated, or afucosylated categories tended to decrease (Fig. 3*D*). Time-series clustering identified decreasing Clusters four and six and increasing Clusters 5 and 9 (Fig. 3*E*). Proteins represented in these clusters were enriched for complement and coagulation cascades, ECM-receptor interaction, and cholesterol metabolism (Supplementary Data 8). Notably, the complement-related set included 48 proteins and 2190 site-specific glycopeptides, many of which were fucosylated (Fig. 3*F*). C4A illustrated the site specificity of these patterns: fucosylated glycoforms at C4A|N226 tended to increase, whereas fucosylated and sialylated glycoforms at C4A|N1328 tended to decrease (Fig. 3*G*), underscoring marked site-specific remodeling of complement glycoforms after chemotherapy.

In paired pre- and post-GnP samples, 482 glycans met the differential-abundance criteria (|log2FC| > 1 and FDR <0.01), representing approximately 70% of quantified glycans (Figs. 3*H*, Supplementary Fig. S7, and Supplementary Data 3). Fucosylated glycans were prominent among the most strongly altered species (Fig. 3*I*). Proteins carrying treatment-associated glycoforms were enriched for complement/coagulation, adhesion, and ECM-related annotations (Fig. 3*J*). Together, these findings delineate a comprehensive landscape of circulating glycoform changes after treatment in PDAC and indicate a dynamic remodeling of glycosylation throughout treatment.

### Plasma Proteomic Alterations after Chemotherapy

**Fig. 4:**
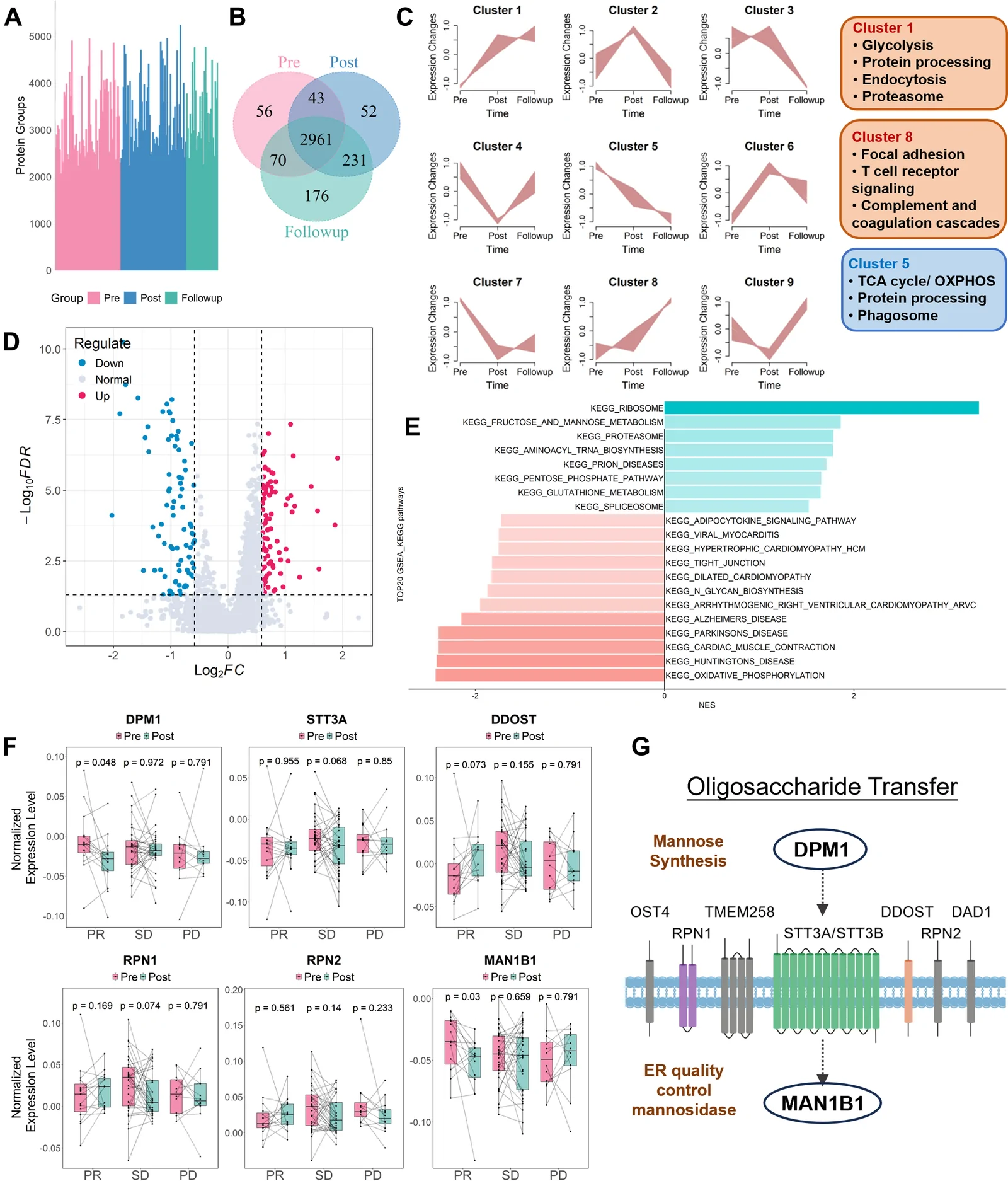
**In-depth plasma proteomic profiling before and after chemotherapy.***A*, Number of proteins identified in each plasma sample across the discovery cohort. *B*, Venn diagram showing the overlap of proteins identified in Pre, Post, and Follow-up samples. (*C*) Mfuzz soft clustering of plasma protein abundance across the three sampling time points, with representative enriched biological processes and pathways annotated for selected clusters. *D*, Volcano plot showing differentially abundant plasma proteins between paired Pre and Post samples. *E*, gene set enrichment analysis (GSEA) of paired Pre versus Post plasma proteomic changes. *F*, Boxplots showing plasma abundance changes of selected proteins related to N-glycan biosynthesis and endoplasmic reticulum-associated glycoprotein processing across response groups. Paired samples from the same patient are connected by grey lines. *G*, schematic representation of the oligosaccharyltransferase-associated N-glycosylation machinery in the endoplasmic reticulum. DPM1, dolichyl-phosphate mannose synthase subunit 1; STT3A, STT3 oligosaccharyltransferase complex catalytic subunit *A*; DDOST, dolichyl-diphosphooligosaccharide-protein glycosyltransferase non-catalytic subunit; RPN1/2, ribophorin I/II; MAN1B1, mannosidase alpha class 1B1.

Plasma proteomics identified 6045 proteins, including 2866 detected across all three sampling groups (Fig. 4, *A* and *B*). The number of detected proteins remained broadly stable over time. Mfuzz analysis identified nine temporal clusters: Clusters one and eight increased, whereas Cluster 5 decreased (Fig. 4*C*). Cluster one was enriched for carbon-metabolism and proteasome annotations; Cluster eight for focal adhesion, T-cell receptor signaling, and complement/coagulation annotations; and Cluster five for oxidative-phosphorylation-related annotations. These enrichment patterns describe groups of covarying circulating proteins in the energy-source pathways and host immunity through chemotherapy.

Paired pre- and post-treatment analysis identified 173 plasma proteins meeting the exploratory criteria (|log2FC| >= 0.585 and nominal *p* < 0.05), including 90 increased and 83 decreased proteins (Fig. 4*D* and Supplementary Data 5). Gene set enrichment analysis (GSEA) showed relative enrichment of ribosome- and proteasome-related signatures and relative depletion of oxidative-phosphorylation- and N-glycan-biosynthesis-related signatures after treatment (Fig. 4*E* and Supplementary Data 5). These plasma associations may link to but do not by themselves demonstrate tumor-cell damage or suppression of the corresponding intracellular pathways.

The KEGG N-glycan biosynthesis pathway was overall downregulated in post-chemotherapy plasma (Fig. 4*E*), but several constituent proteins revealed a bidirectional pattern by response group. In non-responders (SD/PD), core subunits of oligosaccharyltransferase (OST) on endoplasmic reticulum (ER) membrane all decreased, including the catalytic subunit A (STT3A), dolichyl-diphosphooligosaccharide-protein glycosyltransferase non-catalytic subunit (DDOST), and ribophorin I/II (RPN1/2), whereas DDOST and RPN1 increased in the PR group (Fig. 4*F*). Functionally, the ER-associated N-glycosylation axis starts from dolichyl-phosphate mannose synthase subunit 1 (DPM1) supplies mannose for assembly of the lipid-linked oligosaccharide donor to OST, to transfer this oligosaccharide onto nascent polypeptides in the ER, and mannosidase alpha class 1B1 (MAN1B1) trims high-mannose glycans for ER quality control (Fig. 4*G*) (15, 16, 17). Together, these observations nominate an ER N-glycosylation-related protein set for further study.

### Treatment-Associated Plasma Metabolomic Patterns

Untargeted plasma metabolomics detected 1080 annotated metabolites, predominantly lipids and organic acids (Supplementary Fig. S8*A* and Supplementary Data 6). In the paired comparison, 123 metabolites met the differential-abundance criteria (|log2FC| > 0.4 and FDR <0.1), with 60 increased and 63 decreased after treatment (Supplementary Data 7). The gemcitabine-related metabolite 2′,2′-difluoro-2′-deoxyuridine increased markedly (Supplementary Fig. S8*B*). Metabolite-set enrichment highlighted pyrimidine- and arginine-related annotations, with orotate, dihydroorotate, and dCMP among the differential metabolites, whereas branched-chain-amino-acid- and propanoate-related sets were relatively depleted (Supplementary Fig. S8, *C* and *D*). These results reflect differences in circulating metabolite abundance and do not directly measure metabolic flux or pathway activity.

### Dynamic Glycoform Changes Associated with Treatment Response

**Fig. 5:**
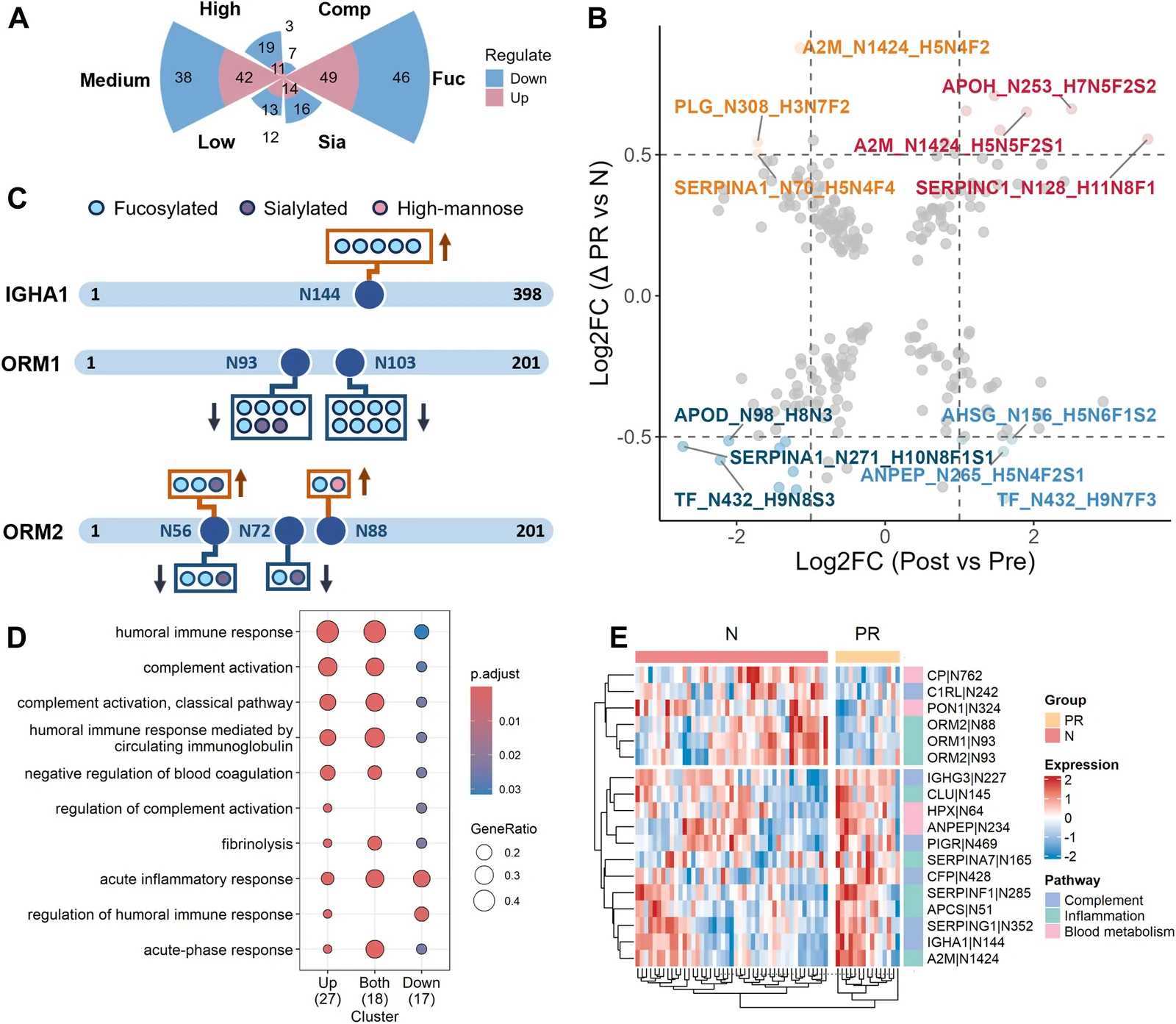
**Dynamic N-glycoform and glycosite occupancy changes associated with radiographic response.***A*, nightingale rose diagram summarizing the numbers of differential dynamic glycans between PR and non-PR patients, with glycans grouped by types including fucosylated (Fuc), sialylated (Sia), or complex (Comp), and by branching complexity including high (≥4 branches), medium (=3 branches), and low (≤2 branches). *B*, four-quadrant scatterplot showing chemotherapy-associated glycoform dynamics, with the x-axis representing the paired within-patient change after chemotherapy, and the y-axis represents the difference in dynamic change between PR and non-PR patients. Colored points denoted glycoforms with at least a 1.2-fold increase or decrease along the axes, and the top three glycoforms in each quadrant were labeled. *C*, lollipop plots showing site-specific N-glycoform changes on IGHA1, ORM1, and ORM2. Asparagine glycosylation sites are indicated along each protein, and arrows denote glycoforms with increased or decreased dynamic abundance in PR relative to the non-PR group. *D*, KEGG enrichment analysis of glycoproteins carrying dynamically altered glycoforms, stratified by whether the corresponding glycoforms were increased, decreased, or showed bidirectional changes in PR group. *E*, heatmap showing differentially altered glycosite occupancy between PR and N patients with functional categories of the corresponding glycoproteins annotated on the *right*.

To highlight features indicative of therapeutic benefit, SD and PD were combined as the non-responder group (N, n = 45), and evaluated against PR group using within-patient “dynamic” changes, ΔFeature via paired post samples subtracting the baseline levels. A total of 140 glycoforms met the exploratory criteria (|log2FC| > 0.5 and nominal *p* < 0.05), including 71 with larger increases and 69 with larger decreases in PR (Fig. 5*A* and Supplementary Data 9). Most of the response-associated Δglycoforms corresponds to the complement and coagulation proteins, such as A2M and SERPINA1, similar to chemotherapy-induced glycoform differences (Fig. 5, *B* and *C*). The direction of change varied by glycosylation site and glycoform, as illustrated by A2M|N1424 and the multiple response-associated glycoforms on ORM1 and ORM2. The 140 glycoforms mapped to 63 glycoproteins, with enrichment for complement activation and humoral immune response annotations (Fig. 5*D*).

After site-summed glycopeptide abundance was normalized to the corresponding protein abundance, 18 N-glycosylation sites met the exploratory occupancy criteria in the PR-versus-N comparison (|log2FC| > 1 and nominal *p* < 0.05), with 12 showing larger increases and six larger decreases in PR (Fig. 5*E* and Supplementary Data 9). Most occurred on different proteins; ORM2 contributed two sites, ORM2|N93 and ORM2|N88, with lower occupancy changes in PR. Increased sites were enriched for complement and humoral immune annotations, whereas decreased sites included acute-phase and antioxidant proteins. Together, these results provide a detailed treatment-responsive plasma glycosylation landscape encompassing plasma glycoforms, glycosite occupancy, and glycoprotein abundance, revealing molecular features associated with differential radiographic responses to chemotherapy in PDAC patients.

### Exploratory Plasma Proteomic Dynamics Associated with Treatment Response

**Fig. 6:**
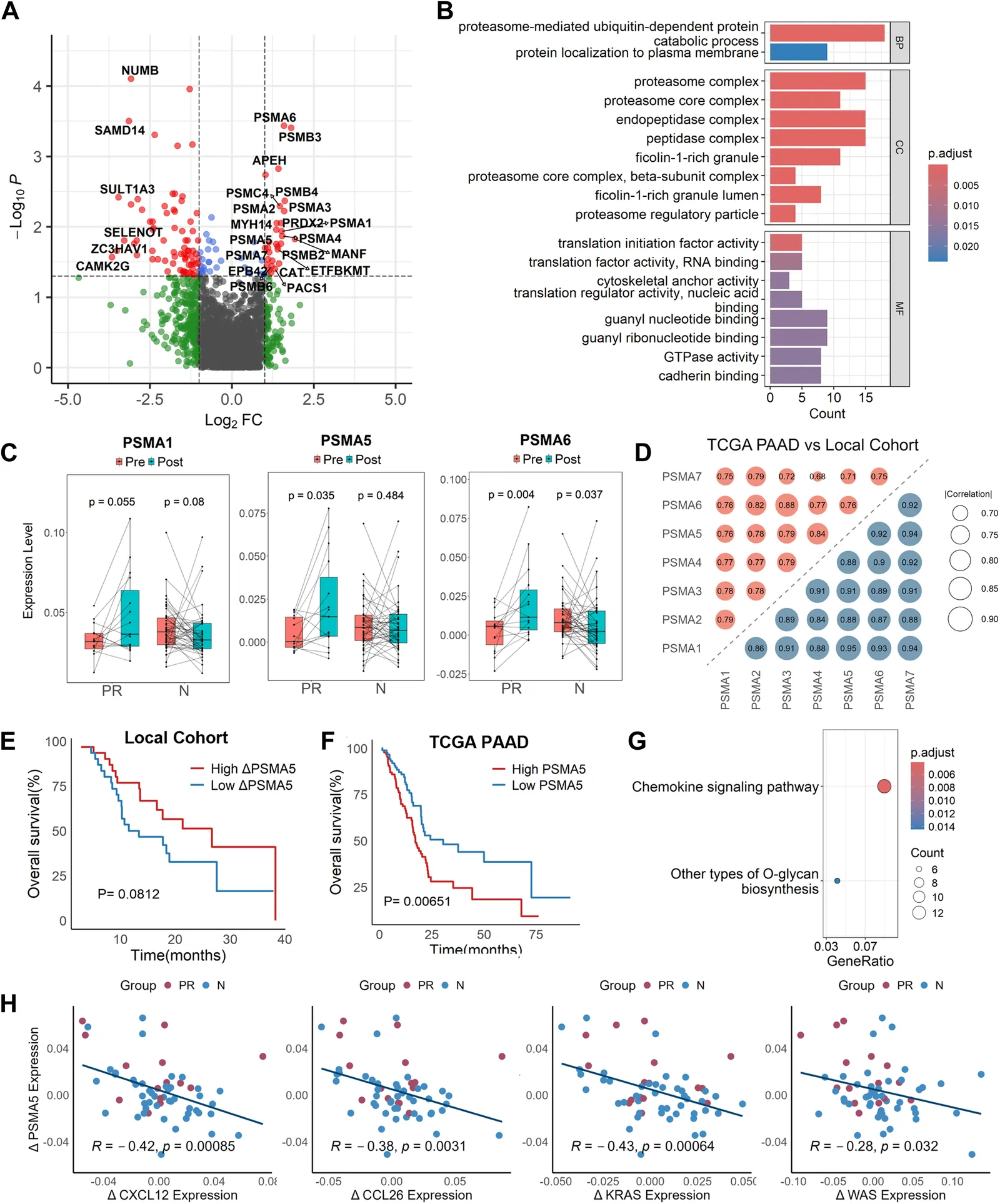
**Plasma proteasome-related protein dynamics in partial responders and non-PR patients.***A*, Volcano plot showing differential dynamic plasma protein changes between PR and non-PR group with the top 20 differentially altered proteins labeled. *B*, gene Ontology enrichment analysis of differentially altered dynamic proteins, including Biological Process (BP), Cellular Component (CC), and Molecular Function (MF) categories. *C*, Boxplots showing paired changes in selected proteasome subunits before and after chemotherapy in PR and non-PR patients with paired samples connected by *grey lines*. *D*, correlation matrix comparing proteasome alpha subunit relationships in the paired local plasma cohort and TCGA PAAD data. *Blue circles* represent correlations among dynamic plasma protein changes in our local paired cohort, whereas red circles represent correlations among expression levels in TCGA PAAD cohort. The correlation coefficient was labeled in the circle. *E*, Kaplan–Meier overall survival analysis of the paired local cohort stratified by high versus low dynamic PSMA5 change. *F*, Kaplan–Meier overall survival analysis of TCGA PAAD patients stratified by high versus low PSMA5 tumor expression (median cutoff). *G*, KEGG pathway enrichment analysis of plasma proteins negatively correlated with dynamic PSMA subunit changes in the local cohort. *H*, exploratory scatterplots showing the associations of dynamic changes between PSMA5 and selected chemokine- or signaling-related proteins with *red dots* representing PR samples and blue dots representing non-PR samples.

Within-patient protein changes were compared between the PR and N groups. Although 103 proteins met the nominal exploratory criteria (|log2FC| > 1 and raw *p* < 0.05), none remained significant after Benjamini-Hochberg correction (Supplementary Data 10). Accordingly, these proteins were carried forward only for exploratory trend analysis. Of the nominally selected proteins, 74 showed smaller and 29 larger changes in PR (Fig. 6*A*); 15 of the latter were proteasome subunits. Exploratory enrichment and correlation analyses also highlighted proteasome-related annotations and coordinated PSMA/PSMB changes in plasma (Fig. 6, *B*–*D* and Supplementary Figs. S9 and S10). Baseline expression patterns in TCGA pancreatic tumor tissue were examined as external context (Fig. 6*D* and Supplementary Fig. S11), but tissue expression and treatment-associated changes in plasma exhibits an unbalanced pattern following chemotherapy.

In the local cohort, a larger within-patient increase in plasma ΔPSMA5 was associated with longer survival (Fig. 6*E*), whereas higher baseline expression of PSMA5 and several other proteasome subunits in TCGA pancreatic tumor tissue was associated with shorter survival (Fig. 6*F* and Supplementary Fig. S12). This seemingly opposite observation may, indeed, reflect the effect of chemotherapy. Yet these observations were obtained from different biological compartments and study settings, therefore they do not establish a causal role in chemotherapy response.

To explore proteins that co-varied with proteasome-related plasma changes, the mean change across PSMA subunits was correlated with all quantified proteins. Proteins with nominal negative correlations (R < −0.3 and *p* < 0.05) were enriched for chemokine-signaling annotations (Fig. 6*G*). CCL24, CCL26, CXCL11, and CXCL12 showed weak-to-moderate negative correlations with PSMA5 change (Fig. 6*H*). These exploratory circulating-protein associations suggest that proteasome-related plasma dynamics may co-vary with changes in selected immune- and chemokine-related circulating proteins during chemotherapy.

Given the clinical distinction between PR and PD, we performed an exploratory analysis among GnP-treated patients with PR (n = 15) or PD (n = 8). Because of the small sample size, this analysis was limited to plasma proteomics. Weighted gene co-expression network analysis (WGCNA) identified a pink module with weak associations in opposite directions for PR and PD (Supplementary Fig. S13*A*). Its module eigengene tended to be higher in PR, but the difference was not statistically significant (Wilcoxon *p* = 0.213; Supplementary Fig. S13*B*). Proteins in this module were enriched for translation- and RNA-processing-related annotations (Supplementary Fig. S14). We next screened for plasma proteins with opposite treatment-associated changes in the PR and PD groups. Mesencephalic astrocyte-derived neurotrophic factor (MANF) was the only protein that increased in PR and decreased in PD; no protein was shared between those decreasing in PR and increasing in PD (Supplementary Fig. S15). This small, exploratory comparison suggests that MANF dynamics may differ by response category, but it does not establish MANF as a response biomarker and requires validation in a larger independent cohort.

### Exploratory Metabolomic Dynamics Associated with Treatment Response

In the PR-versus-N comparison of within-patient changes, 55 metabolites met the nominal exploratory criteria (|log2FC| > 0.4 and raw *p* < 0.05; Supplementary Data 11). Several dipeptides, including Pro-Leu, Ile-Val, and Pro-Ile, showed larger decreases in PR, whereas lysine, aspartic acid, and L-valine showed larger increases (Supplementary Fig. S16*A*). Enrichment analysis highlighted pyrimidine- and amino-acid-related metabolite sets (Supplementary Fig. S16*B*), providing hypothesis-generating associations in plasma metabolomics with differential treatment response.

### Exploratory N-glycopeptide-Based Prognostic Modeling

**Fig. 7:**
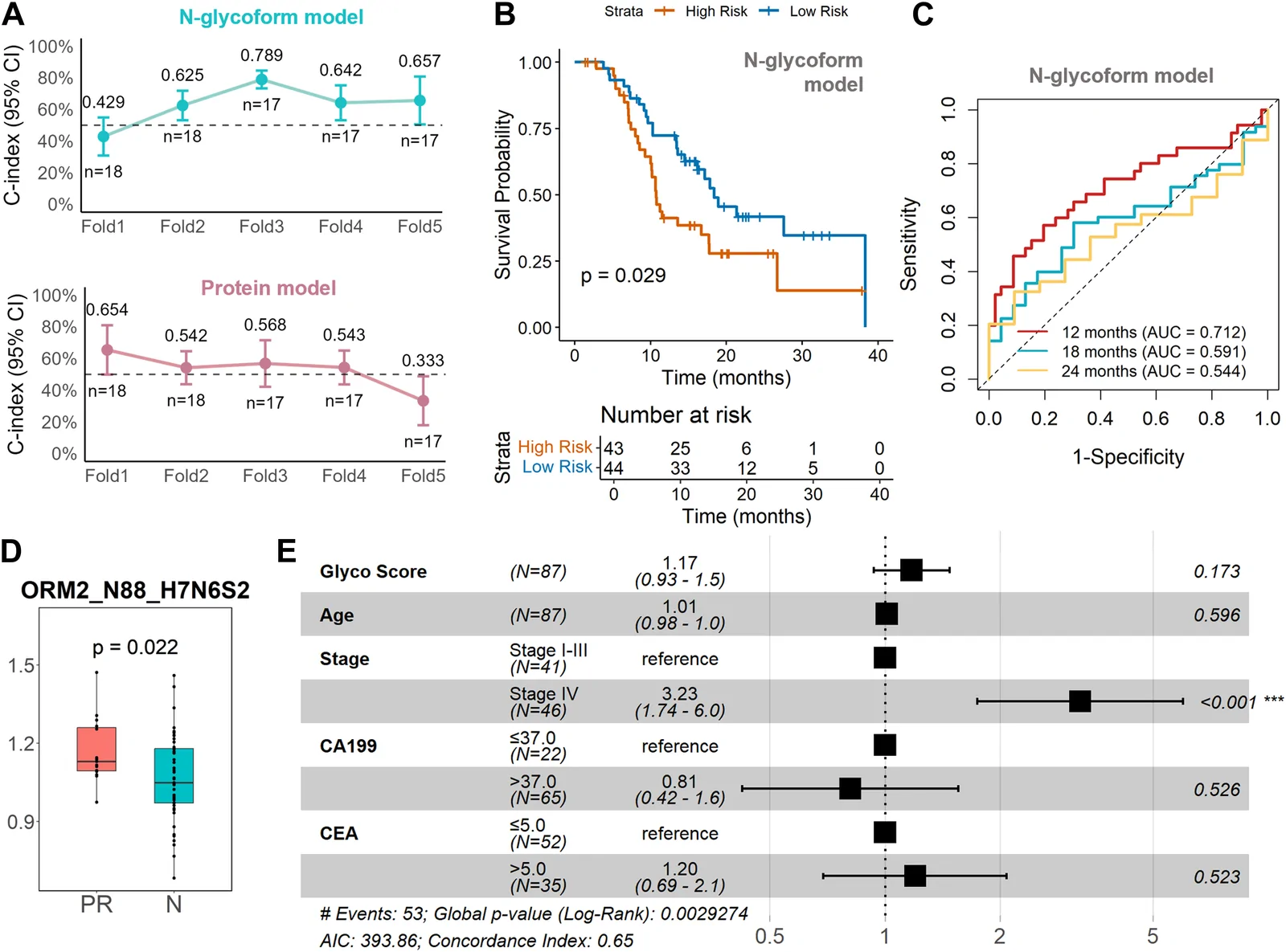
**Prognostic modeling using baseline plasma N-glycoform features.***A*, fold-wise performance of the baseline N-glycoform model and the baseline protein model evaluated using outer-loop cross-validation. Points indicate the C-index estimated in each held-out outer test fold, and error bars indicate 95% confidence intervals. *B*, Kaplan–Meier survival curves for patients stratified into high- and low-risk groups according to the cross-validated N-glycopeptide-derived risk score. *C*, time-dependent receiver operating characteristic (ROC) analysis of the N-glycoform risk score for predicting overall survival at 12, 18, and 24 months. *D*, Boxplot showing baseline abundance of ORM2_N88_H7N6S2 in PR and non-PR patients. *E*, multivariable Cox proportional hazards regression including the N-glycoform risk score and key clinical covariates (age, clinical stage, CA19-9, and CEA). Hazard ratios are shown with 95% confidence intervals. The N-glycoform risk score was modeled as a continuous variable. PR, partial response; non-PR, stable disease or progressive disease; AUC, area under the curve; C-index, concordance index.

To assess the prognostic utility of plasma glycosylation features, we implemented a leakage-controlled, fully nested cross-validation workflow using the complete N-glycopeptide matrix as model input. The N-glycopeptide model achieved a C-index of 0.591 (95% CI, 0.507–0.674), indicating modest out-of-fold discrimination (Fig. 7*A*). Cross-validated risk scores separated the high- and low-risk groups (log-rank *p* = 0.029; Fig. 7*B*). Time-dependent AUC was 0.712 at 12 months, 0.591 at 18 months, and 0.544 at 24 months (Fig. 7*C*), indicating that discrimination was strongest at the earliest evaluated time point and approached chance level later.

Eleven N-glycopeptides were selected in at least four outer folds and were therefore designated stable candidates within this dataset (Supplementary Data 12 and Supplementary Figs. S17). ORM2_N88_H7N6S2 was more abundant in PR than in N (nominal *p* = 0.022; Fig. 7*D*). Applying the same modeling framework to the plasma proteome did not produce a comparably consistent stratification signal (Supplementary Fig. S18). In the multivariable Cox model, stage IV was associated with worse survival than stages I-III (HR, 3.23; 95% CI, 1.74–6.00; *p* < 0.001), whereas the glyco-score was not statistically significant (HR, 1.17; 95% CI, 0.93–1.50; *p* = 0.173; Fig. 7*E*). Collectively, these results support the feasibility of leveraging plasma N-glycopeptide features as clinically informative biomarkers and suggest that glycoform-level measurements might be exploratory for further examination.

## Discussion

Plasma-omics studies in PDAC have identified circulating protein patterns associated with chemotherapy outcomes, frequently involving inflammation, coagulation, and complement modules (18) (https://www.nature.com/articles/s41591-023-02790-x). However, most studies have focused on total protein abundance, whereas longitudinal, site-specific glycoform changes in relation to objective treatment response remain less well characterized (19) (https://www.nature.com/articles/s41392-024-01886-1) (https://www.nature.com/articles/s41467-025-57916-1).

In this context, our work is differentiated by focusing on response-linked, longitudinal, site-specific intact N-glycopeptide trajectories alongside deep plasma proteome remodeling, rather than only reporting protein-level abundance shifts. Technically, our workflow is optimized for confidence and structural resolution of closely related glycoforms: extended nanoLC gradients improve chromatographic separation of glycopeptides (including near-isobaric/closely eluting species), and high ion-accumulation settings improve precursor statistics and MS/MS signal quality—both of which are crucial for robust site- and glycoform-level assignment in complex plasma. Conceptually, these design choices enable us to map therapy-associated glycosylation alteration onto clinically meaningful endpoints, thereby filling an unmet need suggested by prior PDAC response-centric proteomics (largely host-module driven) and by glycosylation studies that have been more diagnostic/prognostic than treatment-response focused (https://www.nature.com/articles/s41392-024-01886-1) (18, 19, 20). At the same time, recent advances in high-throughput plasma glycoproteomics (including narrow-window DIA on Orbitrap Astral) demonstrate the complementary value of fast, high-coverage acquisition for discovery at scale, while our strategy prioritizes separation and identification robustness needed to interpret glycoform-level biology in a response-stratified cohort (21).

A major finding from the glycopeptidomic layer was that complement- and coagulation-related glycoproteins accounted for a substantial fraction of the treatment- and response-associated glycoform patterns. C4A, A2M, ORM1/2, and IGHA1 illustrated that changes could differ by glycosylation site and glycan composition even when total protein abundance changed little. These observations are compatible with treatment-associated changes in systemic inflammatory or acute-phase processes, but plasma measurements cannot identify the producing cell type or determine whether the glycan changes alter complement function, tumor clearance, or chemotherapy sensitivity. Such possibilities require direct functional testing (11).

Proteasome-related proteins also showed coordinated plasma dynamics in the exploratory response analysis. A larger increase in several PSMA/PSMB subunits was observed in responders, whereas baseline expression of some proteasome genes in tumor tissue has been associated with adverse outcomes (22). These findings are not necessarily contradictory because tissue expression and longitudinal plasma abundance represent different compartments and time scales. Potential explanations include release from injured cells or changes in host-cell turnover, but the present data cannot distinguish among these possibilities or infer proteasome activity. The proteasome signal should therefore be regarded as a response-associated candidate for validation (23, 24, 25). The functional significance of these observations and their potential therapeutic implications require further experimental validation.

The N-glycopeptide model showed modest overall discrimination, with better performance at 12 months than at later time points. This pattern may reflect the limited ability of pretreatment plasma measurements to capture subsequent changes in therapy and disease course. The stable features, including the ORM2-derived glycopeptide, are candidate markers within this dataset; their clinical utility cannot be established without independent validation and assessment of calibration and added value over standard clinical factors.

Several limitations constrain our data interpretation. The study was conducted at a single center with a modest sample size, and some analyses involved small response subgroups and variable sampling intervals. The proteomic Delta comparison yielded no proteins significant after multiple-testing correction, so the raw-P-value-selected trends are hypothesis-generating. The prognostic model lacked external validation, and plasma measurements cannot resolve tissue or cellular origin, or causal mechanisms. Residual confounding by disease burden, treatment exposure, and host response also cannot be excluded.

In summary, longitudinal plasma multi-omics identified treatment-associated and response-associated patterns in intact N-glycopeptides, proteins, and metabolites in PDAC. Site-specific glycoforms and proteasome-related plasma dynamics emerged as candidates for further investigation, while the prognostic analysis provided preliminary evidence that glycoform-resolved measurements may contain information not captured by total protein abundance alone. These findings support further exploration in larger, independent cohorts and targeted experiments for research validation.

## Generative AI Statement

During the preparation of this work, the authors used ChatGPT to collect background information, polish the language and improve the clarity and readability of the manuscript. After using this tool, the authors reviewed and edited the content as needed and take full responsibility for the content of the publication.

## Ethics Approval

This study was conducted in accordance with the principles of the Declaration of Helsinki and was approved by the Ethics Committees of Zhejiang Provincial People's Hospital under ethical approval number 2022QT010.

## Data Availability

The proteome raw datasets have been deposited to the ProteomeXchange Consortium (PXD069472) via the iProX partner repository under Project ID, IPX0013306000 (https://www.iprox.cn/page/PSV023.html;?url=1784515027694HmZq, password: Fh7Q). The RAW and search files of intact N-glycopeptidomics and in-depth proteomics were uploaded into separate subprojects. The more detailed description could be found in the README file under the above link. The remaining data are available within the Supplementary Data.

## Supplemental data

This article contains supplemental data.

## Conflict of interest

The authors declare that they have no conflicts of interest with the contents of this article.
