# Supplementary material for: Dynamic Alterations of Site-Specific N-Glycoform and Plasma Proteasome Signatures Stratify Chemotherapy Benefit in Pancreatic Ductal Adenocarcinoma
Source: Mol Cell Proteomics. 2026 Aug 17;25(9):101642. doi: 10.1016/j.mcpro.2026.101642 (PMC13580066; doi:10.1016/j.mcpro.2026.101642)

Supplementary Figures

Figure S1. Clinical factors and overall survival. (A) Kaplan-Meier curves stratified by sex, age, chemotherapy, response, and liver metastasis; log-rank P values are shown. (B) Multivariable Cox regression showing hazard ratios and 95% confidence intervals.

A

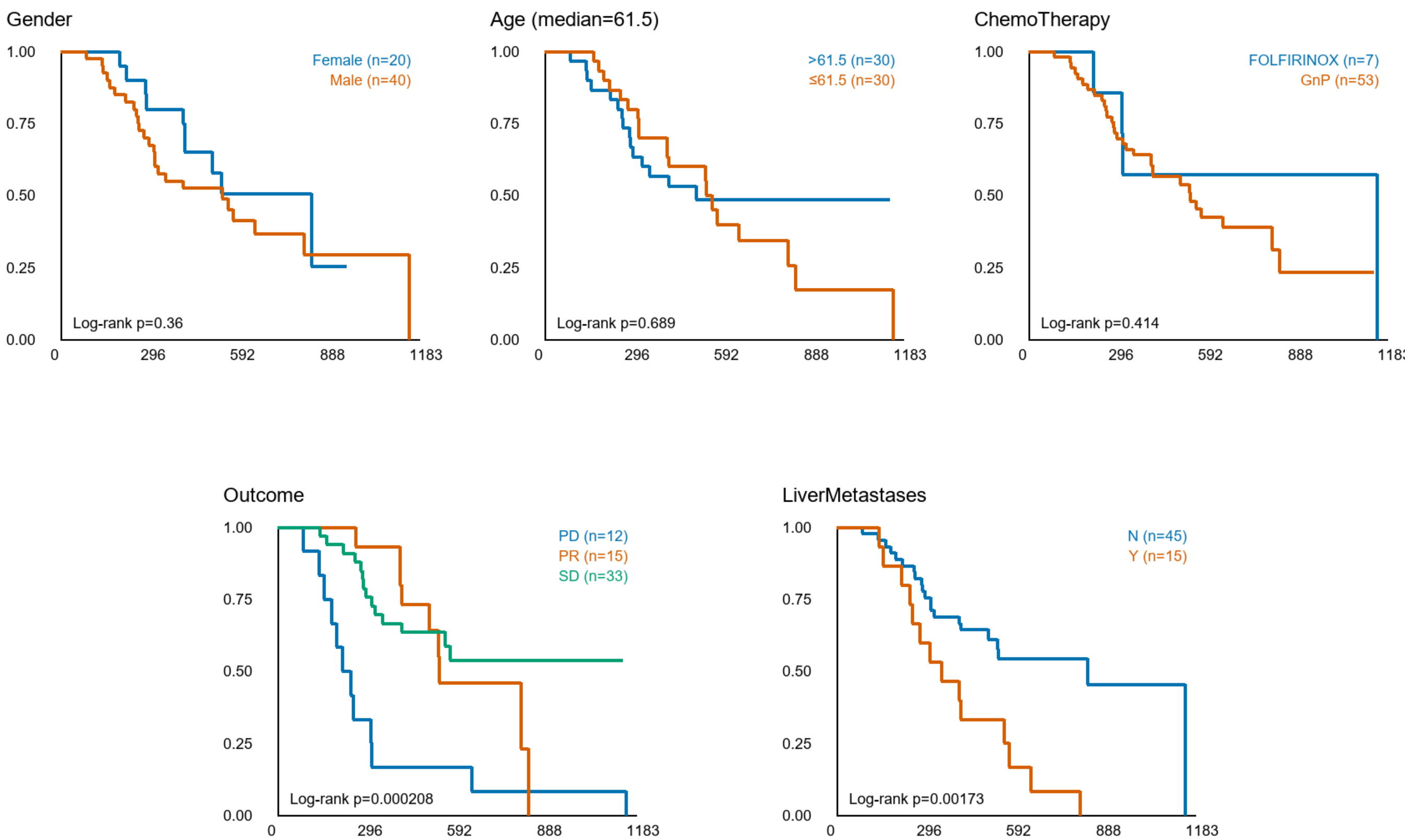

B

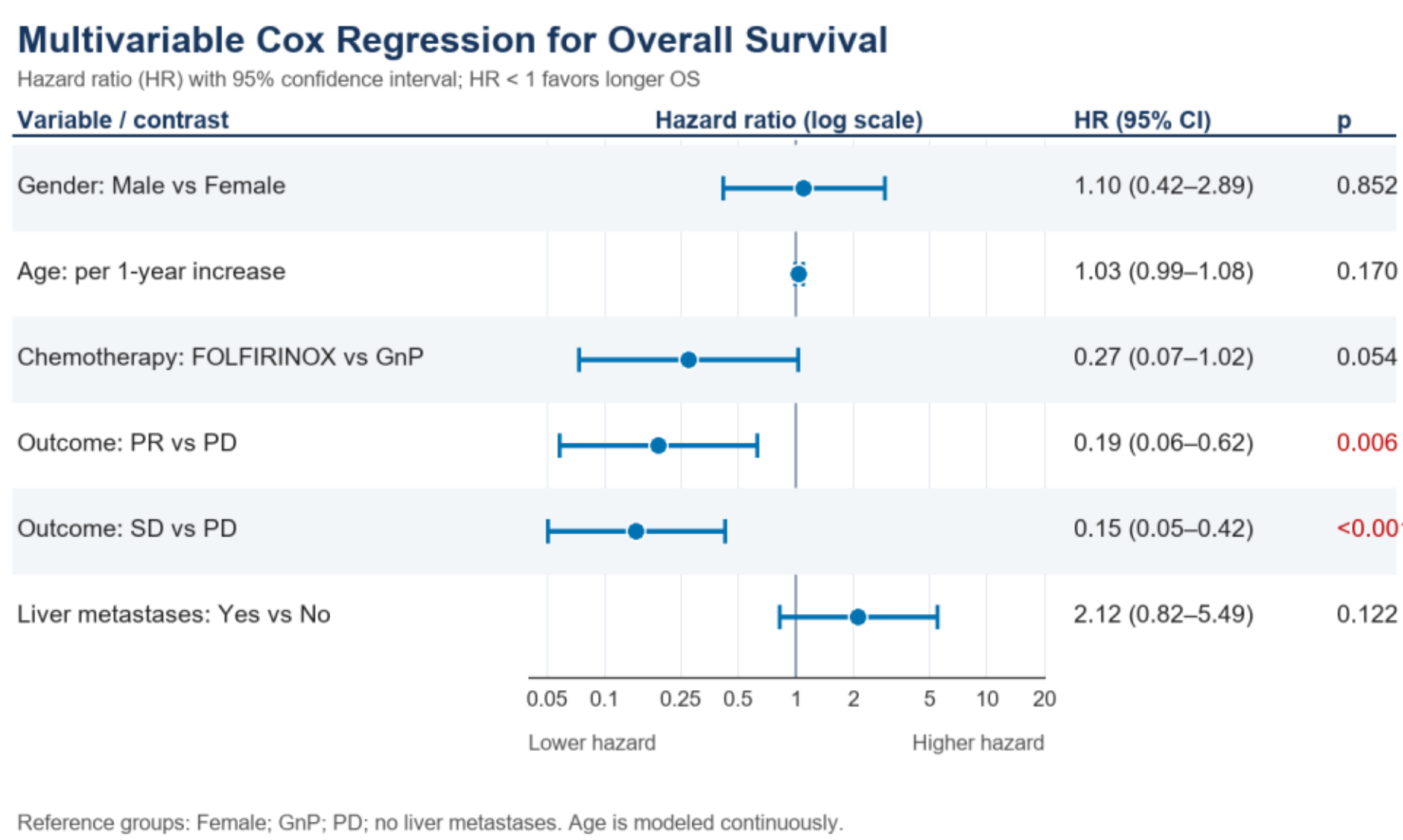

Figure S2. Quality control of intact N-glycopeptidomics. (A) Chromatographic peak full width at half maximum (FWHM) across analytical runs. (B) Distribution of feature-level quantitative coefficients of variation (CVs) calculated from repeated pooled quality-control (QC) injections after normalization.

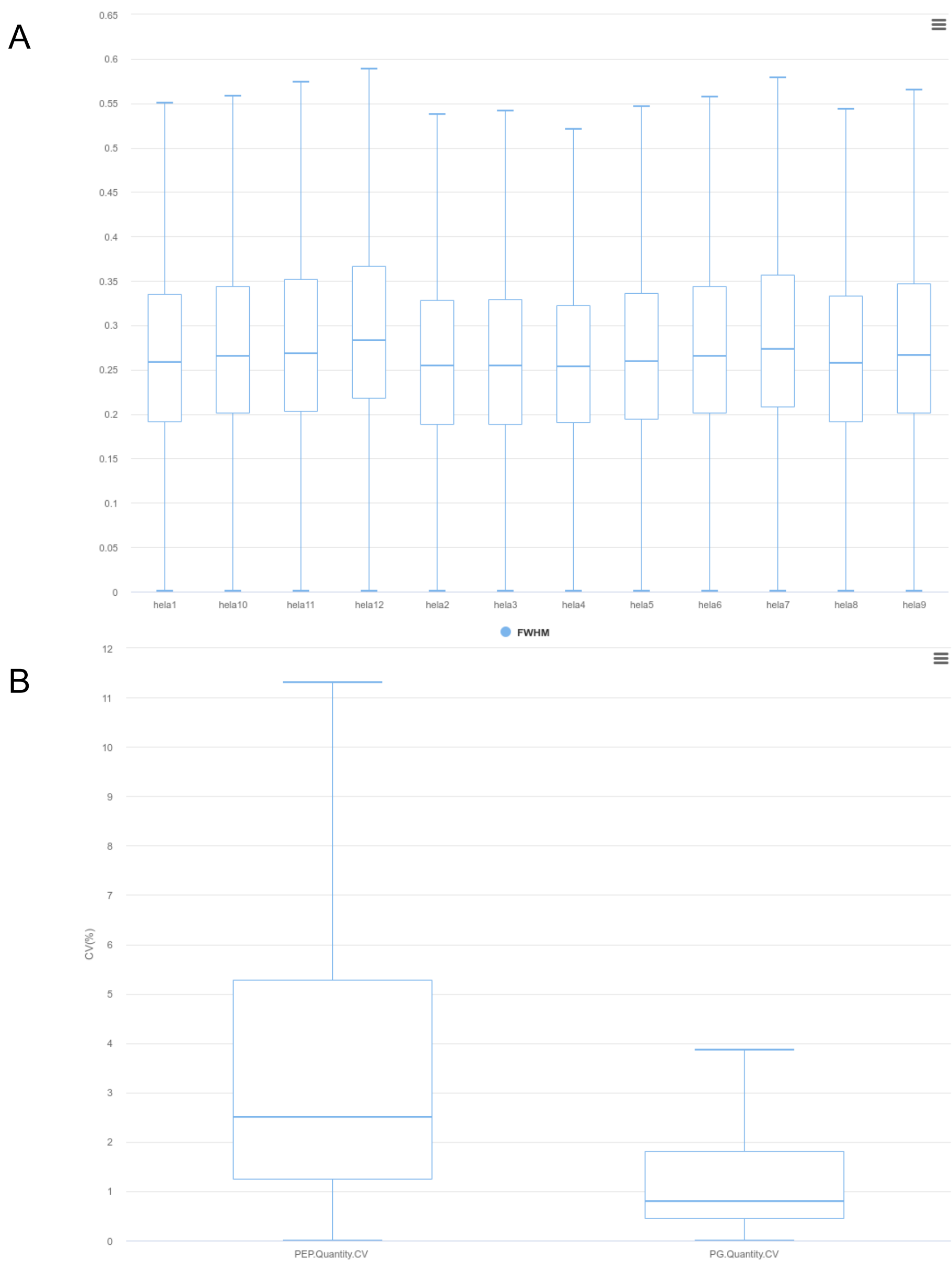

Figure S3. Quality control of deep plasma proteomics. (A) Chromatographic peak FWHM across analytical runs. (B) Distribution of feature-level quantitative CVs calculated from repeated pooled QC injections after normalization. (C) Principal component analysis (PCA) of study and pooled QC samples.

A

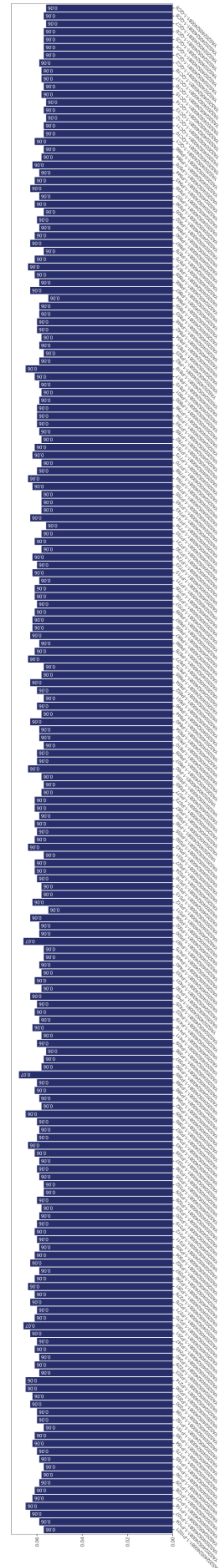

B

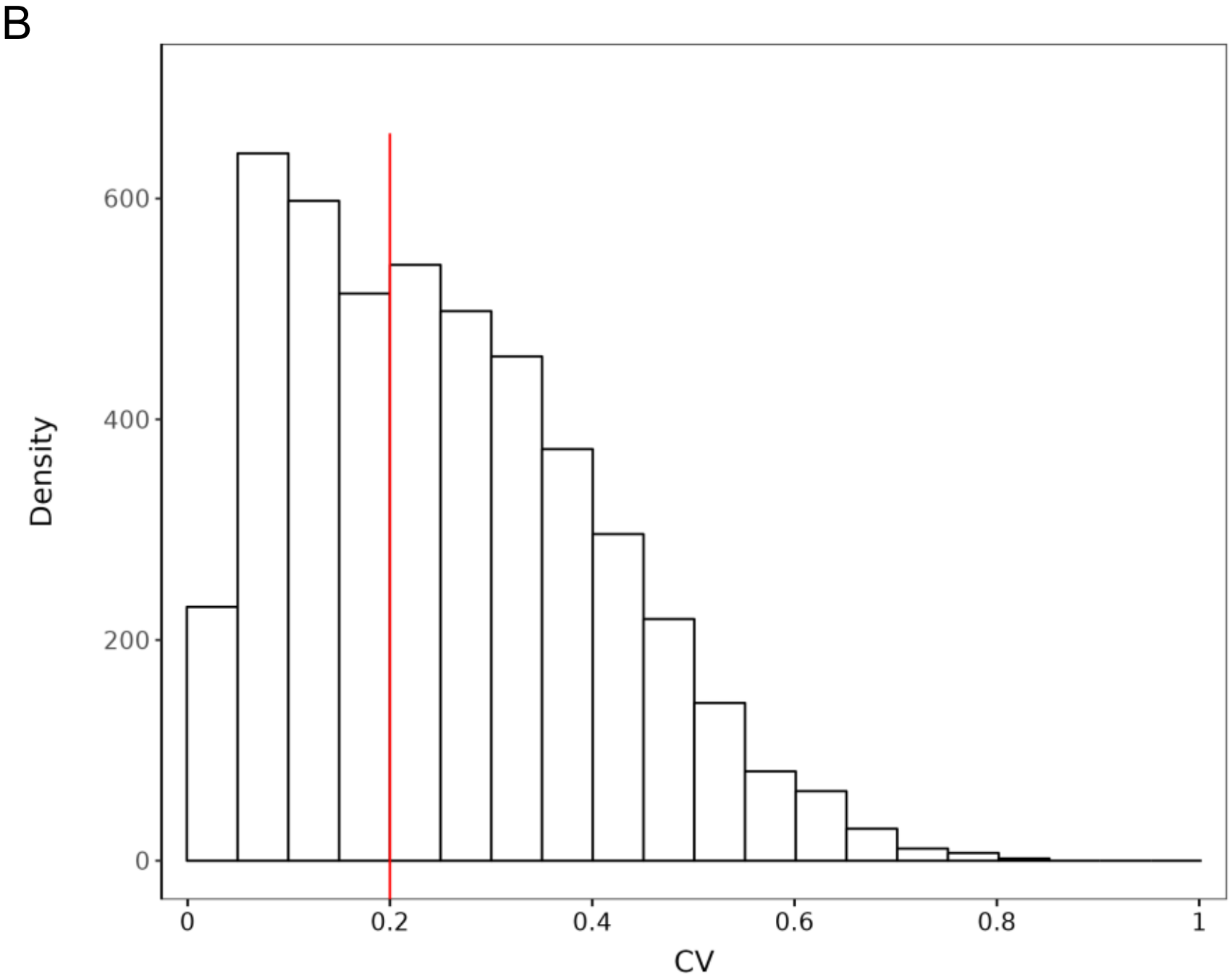

C

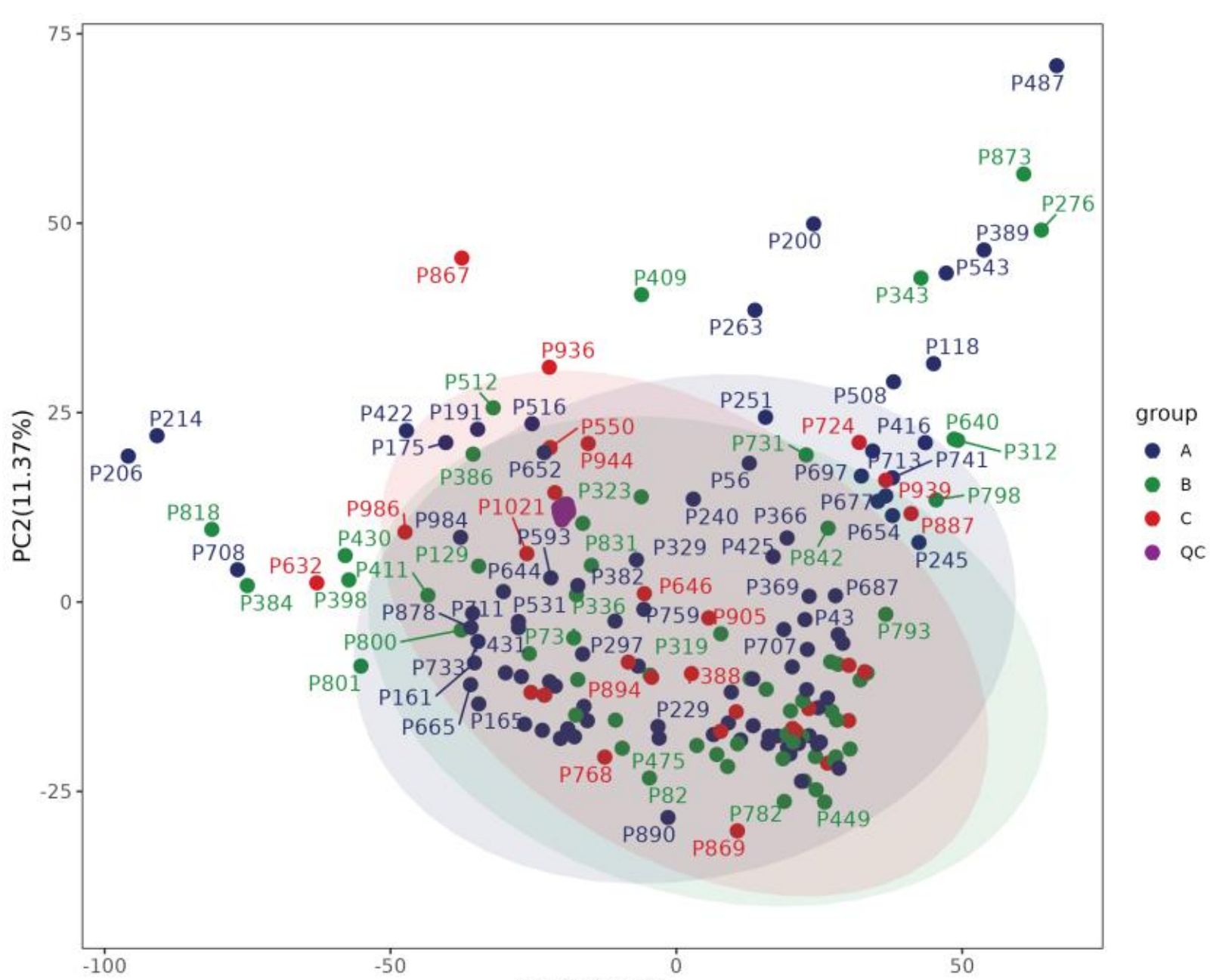

Figure S4. Quality control of untargeted plasma metabolomics. (A, B) Multivariate control charts (MCC) for pooled QC samples in positive- and negative-ionization modes, respectively. (C) PCA of study and pooled QC samples.

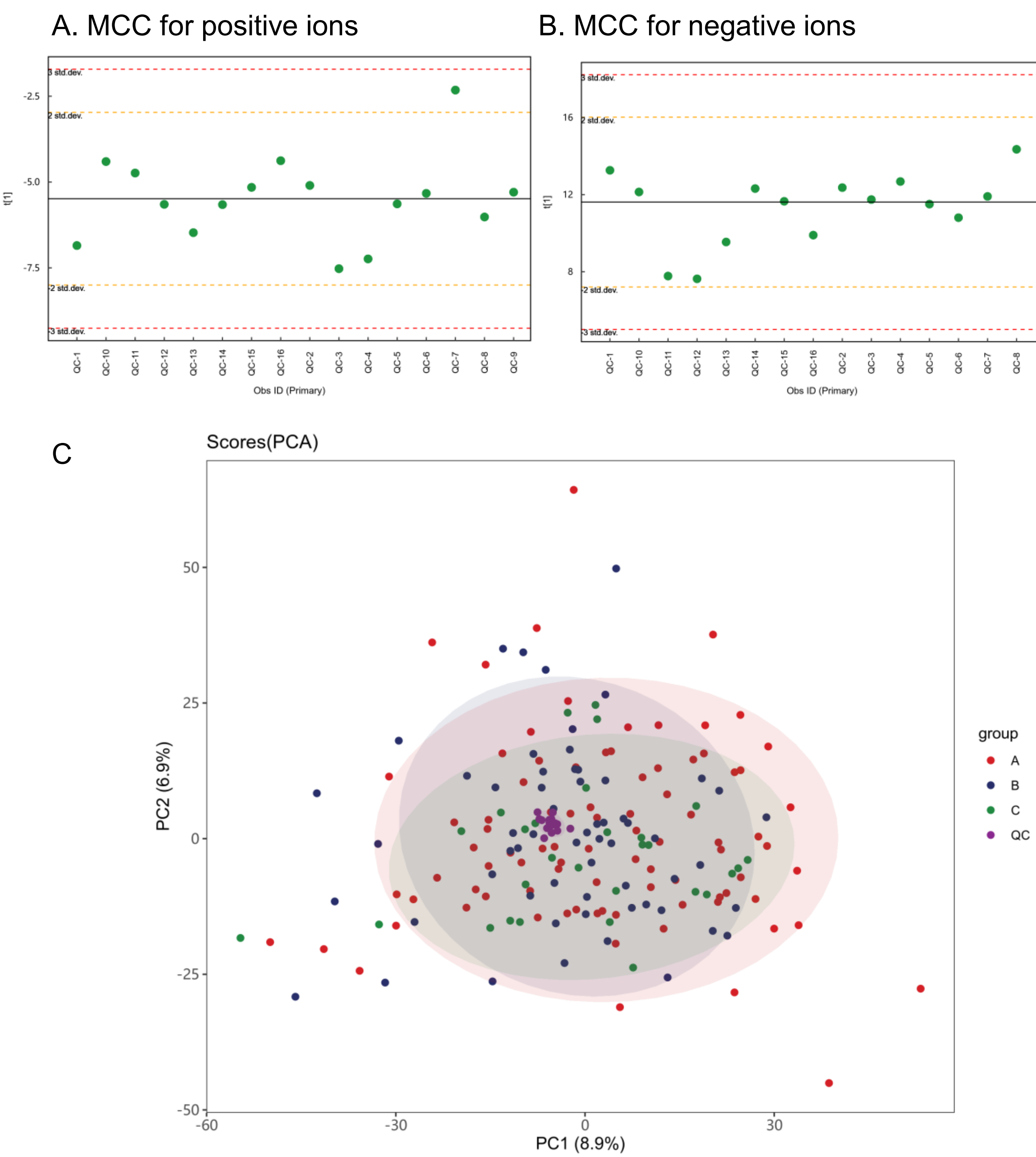

Figure S5. Detection of intact N-glycopeptides across longitudinal sampling groups. (A) Venn diagram of glycoforms detected in Pre, Post, and Follow-up samples. (B) Numbers of detected glycopeptides per sample in each group; P values are shown above the comparisons.

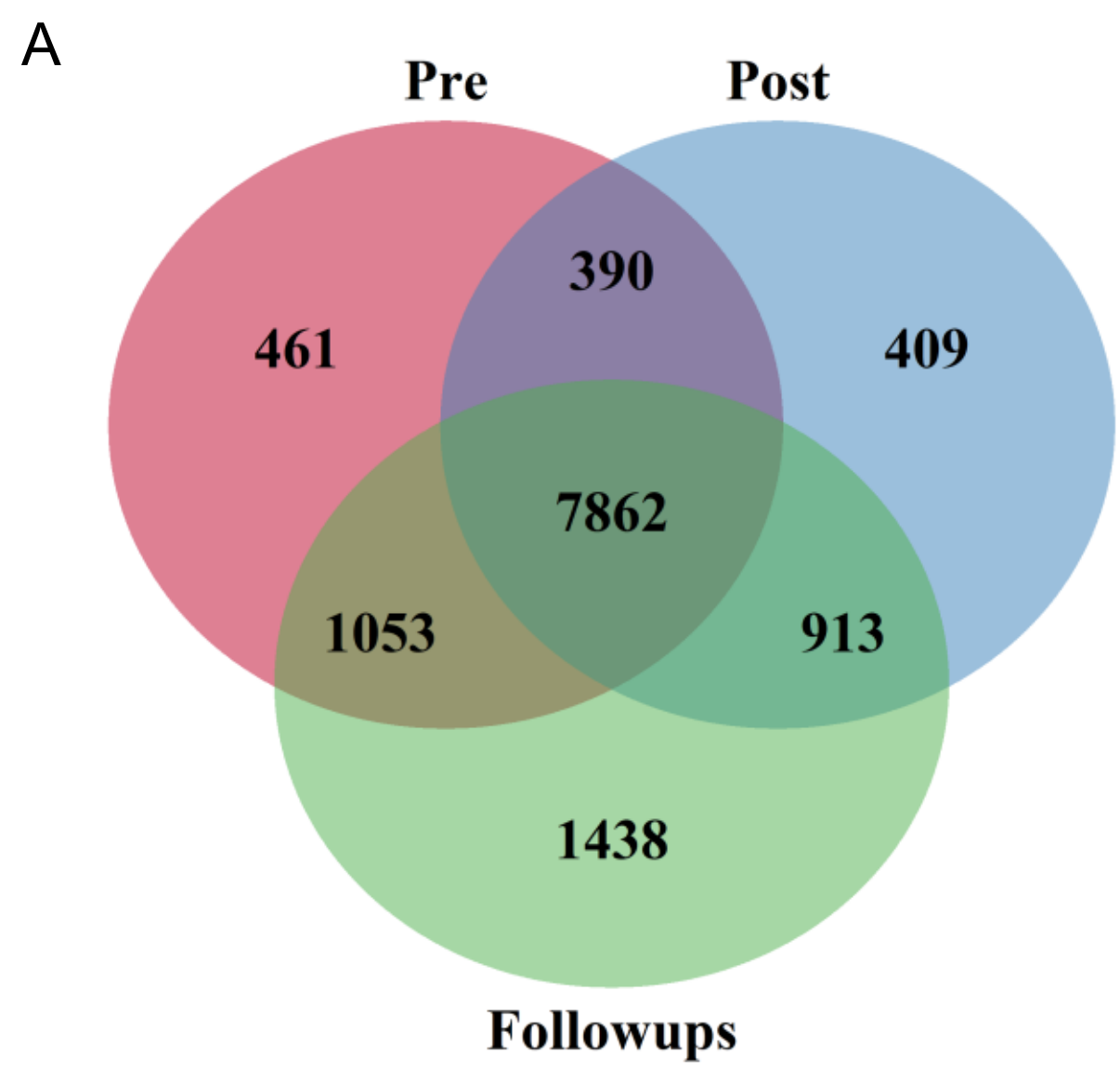

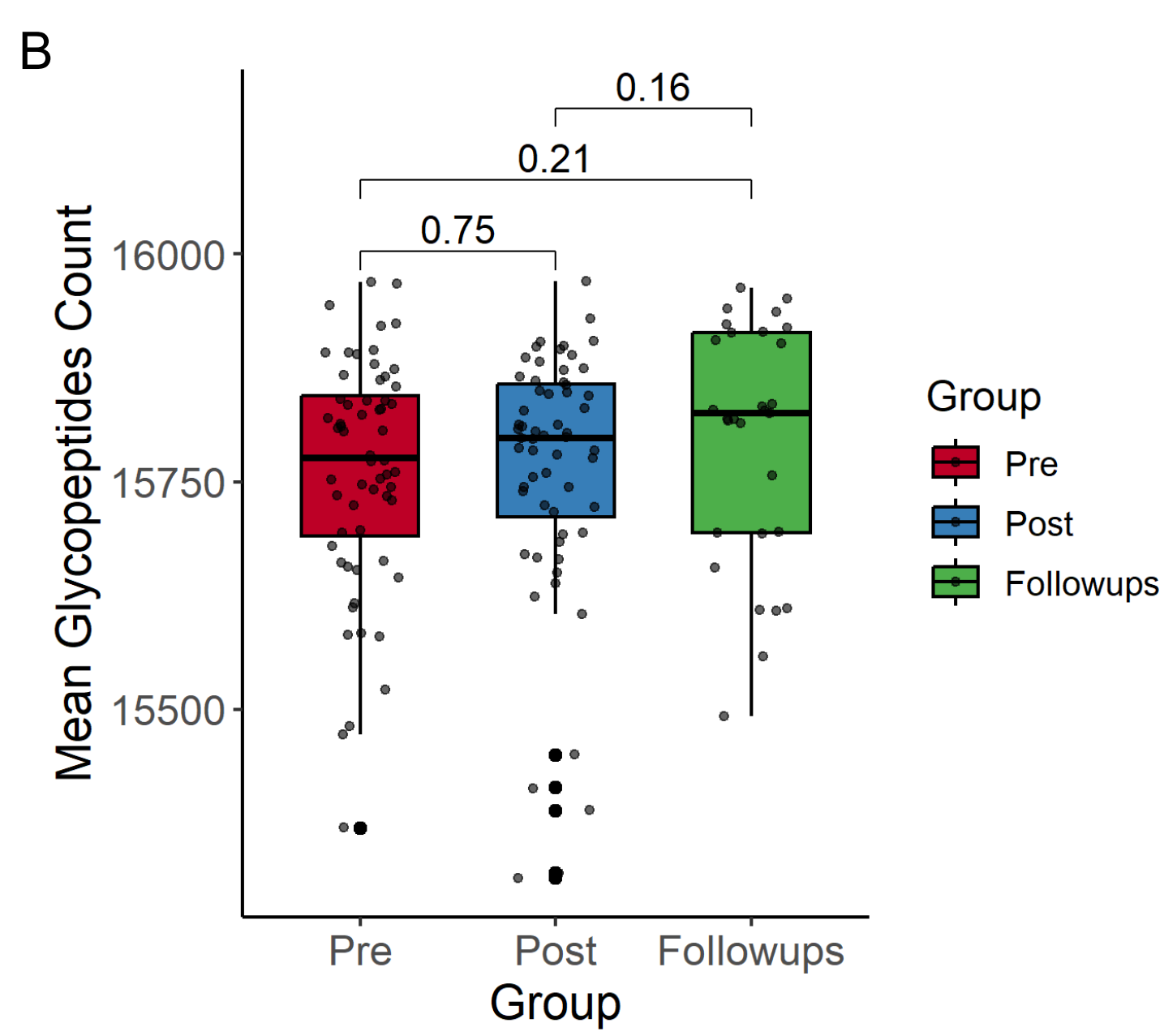

Figure S6. Summary of identified N-glycosylation sites. (A) Annotation sources for identified glycosites. (B) Number of glycosites per protein. (C) Number of glycans per glycosite. (D) Sequence motif surrounding identified glycosites.

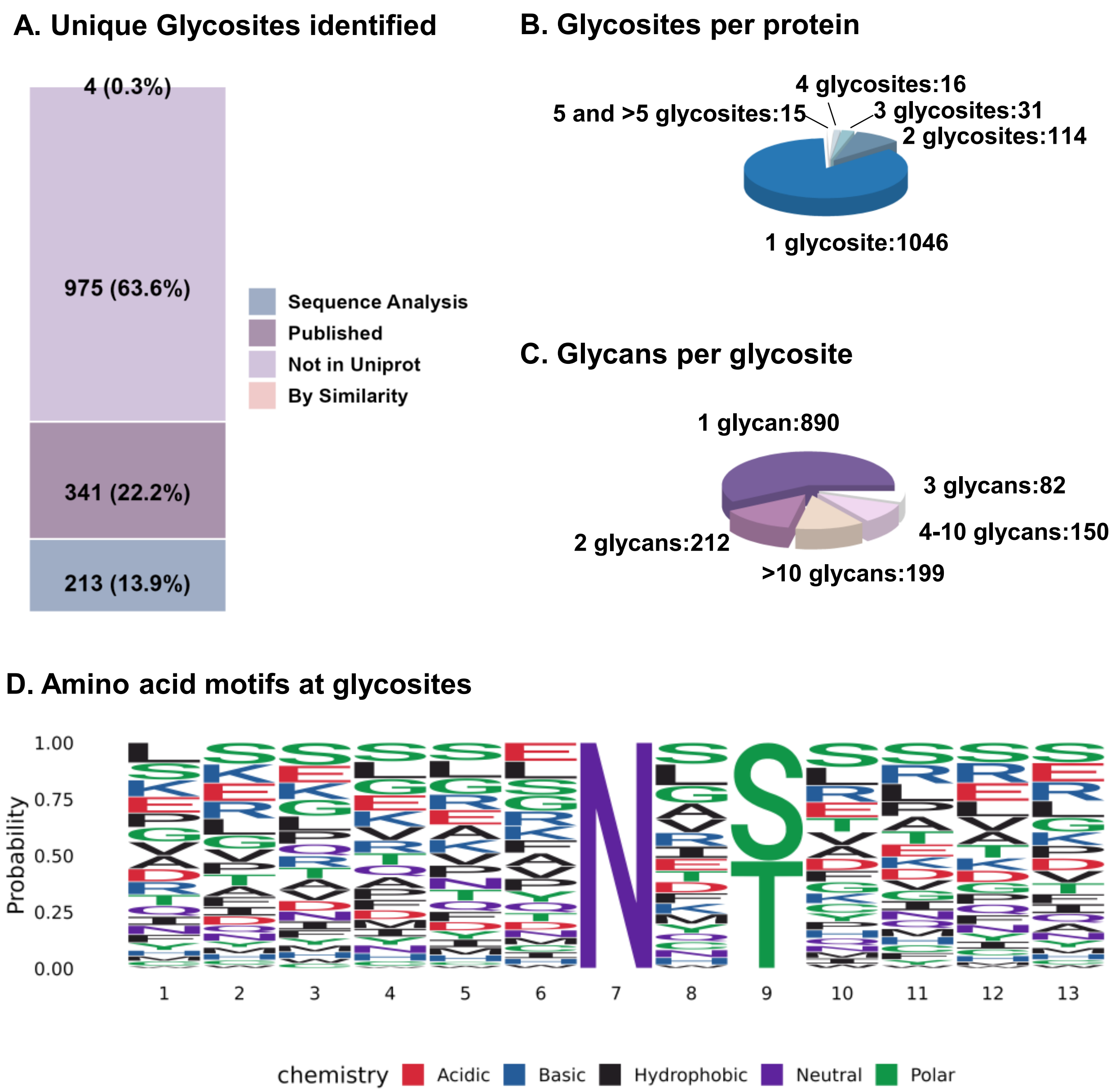

Figure S7. Treatment-associated changes in intact N-glycoforms and glycans. (A) PCA of Pre and Post samples. (B) Volcano plot of paired treatment-associated changes. (C) Counts of glycan structural classes in the total and differential feature sets. (D) Relative proportions of structural classes in each set.

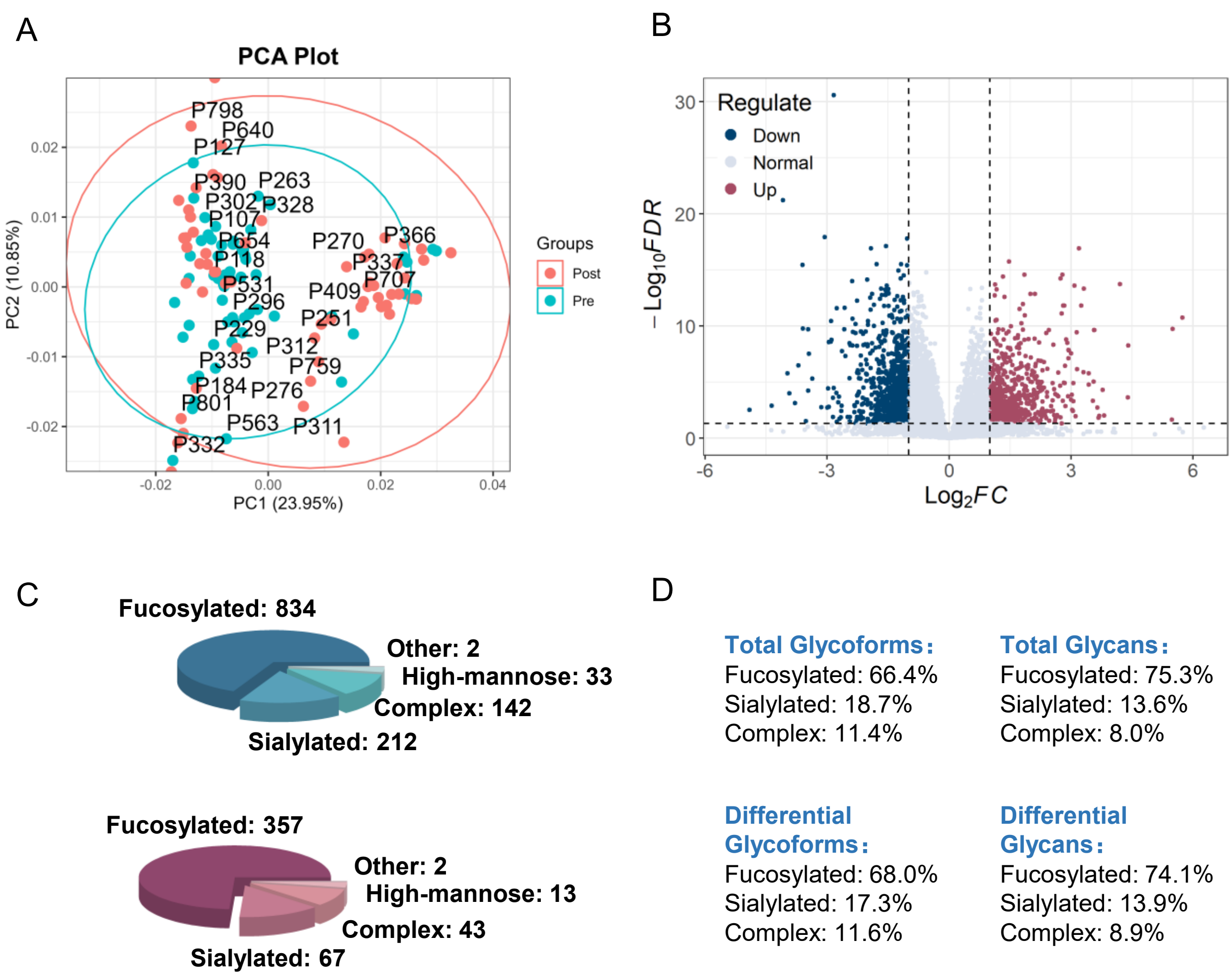

Figure S8. Untargeted plasma metabolomics. (A) Annotated metabolite classes. (B) Volcano plot and (C) PCA for Pre versus Post. (D) Enrichment of metabolite sets among increased and decreased features.

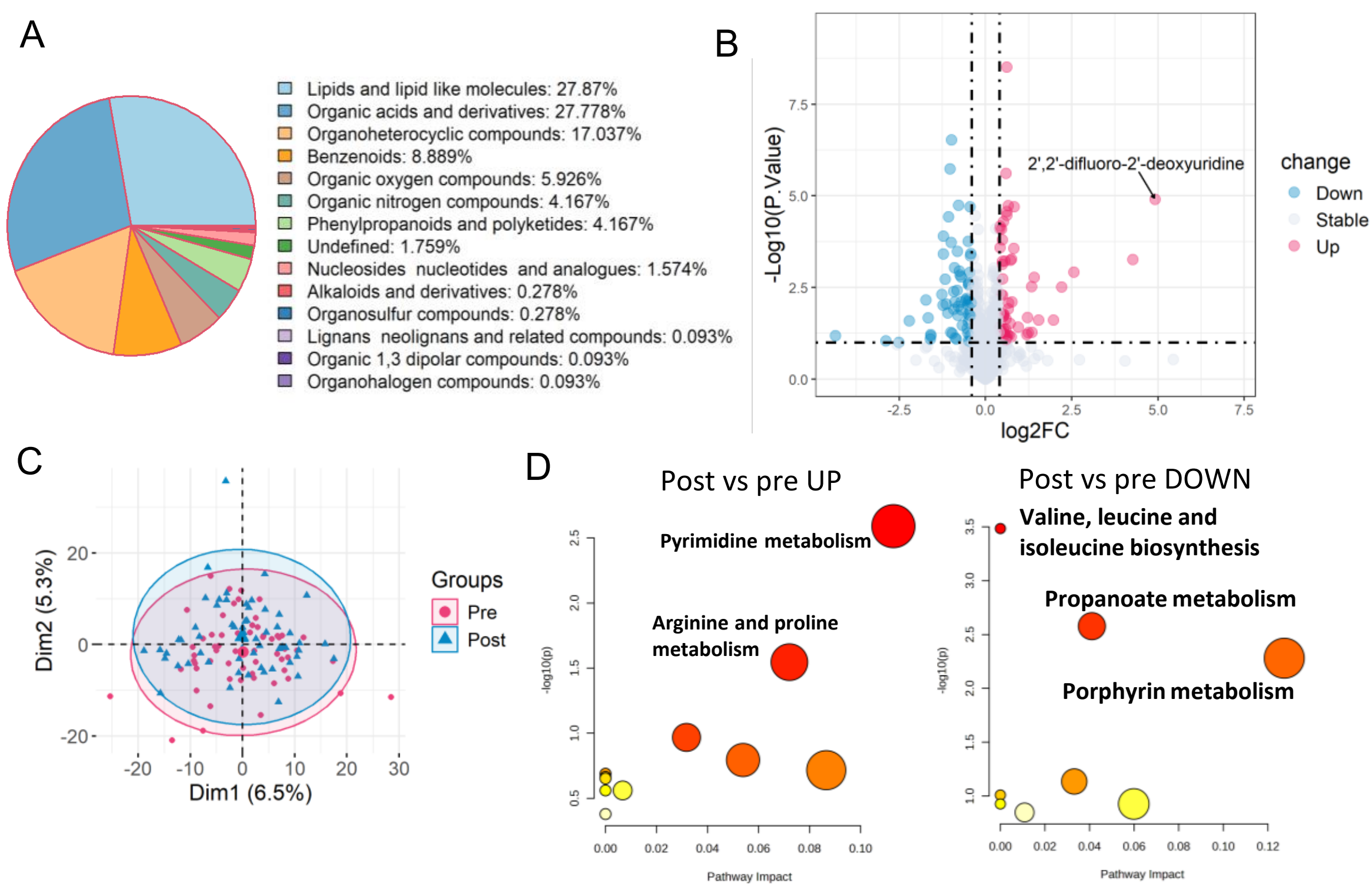

Figure S9. Paired pre- and post-treatment plasma abundance of (A) PSMA and (B) PSMB subunits in the PR and N groups. Lines connect paired samples; nominal P values are shown.

A. PSMA2/4/6/7

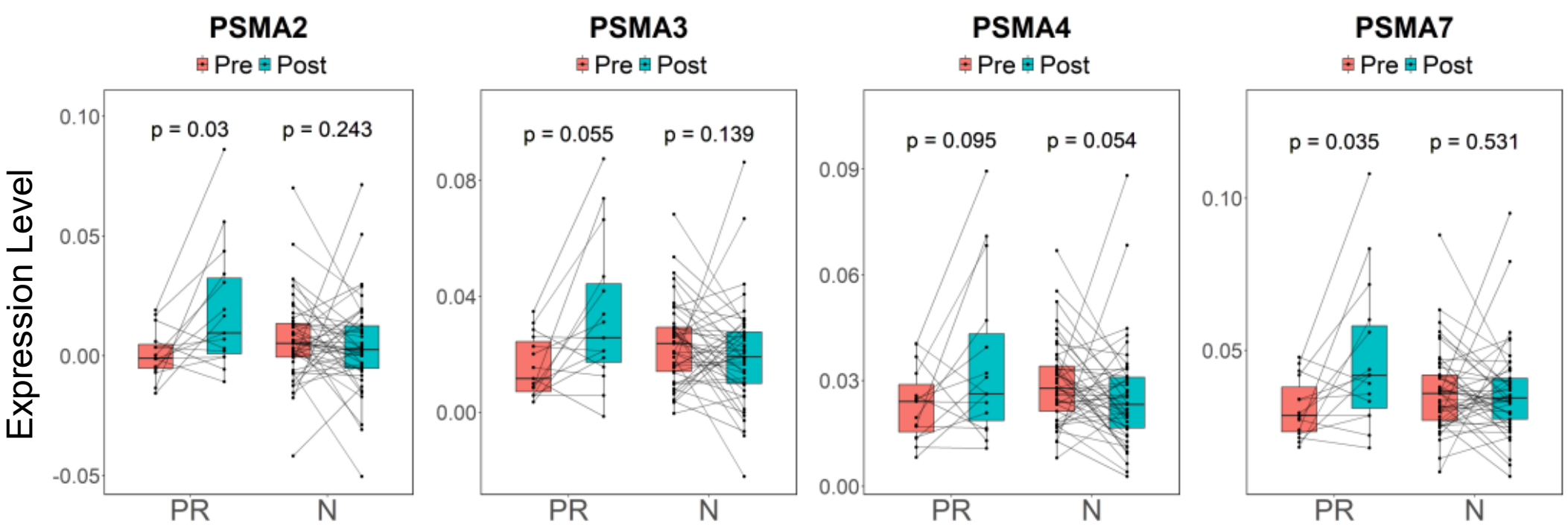

B. PSMB1-9

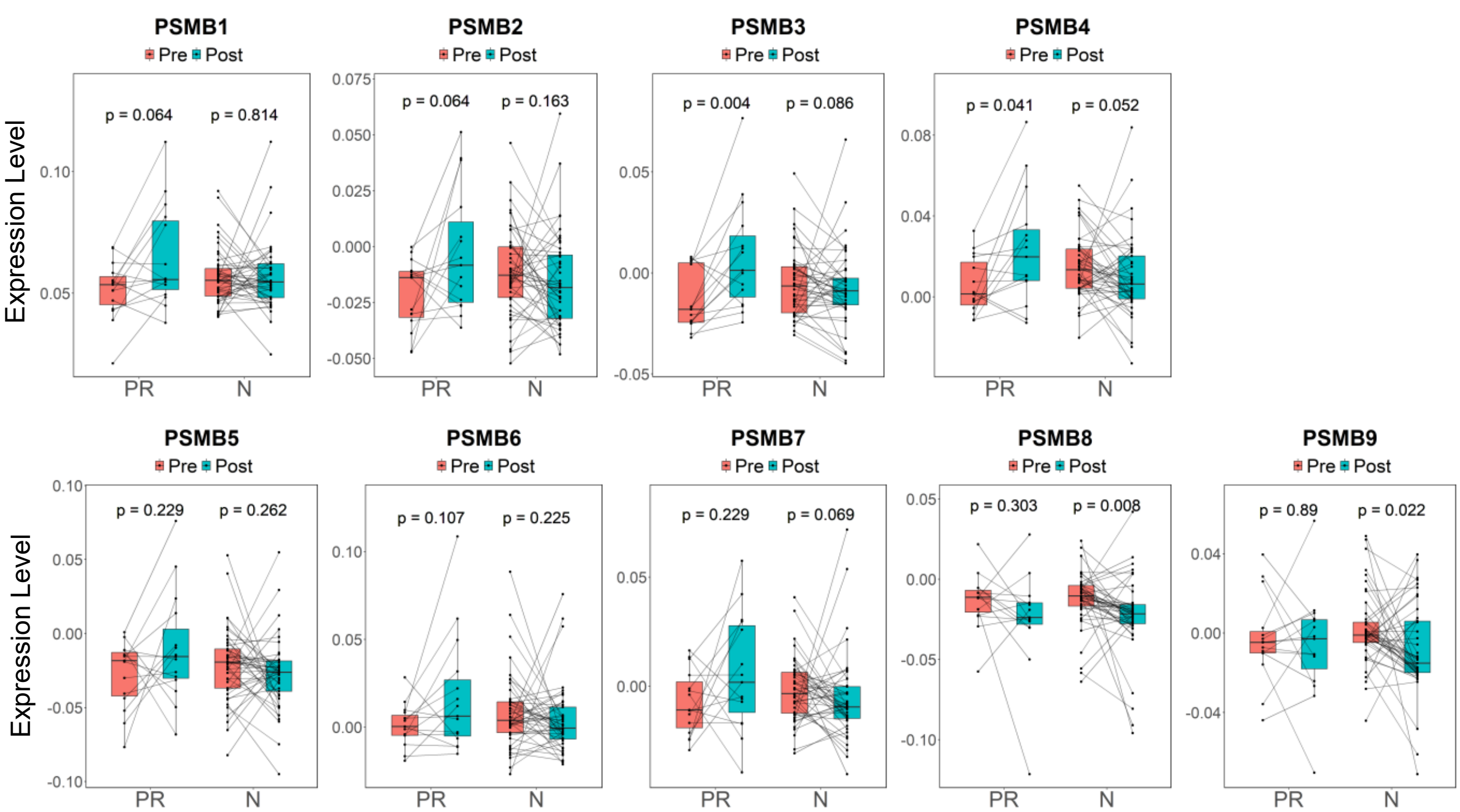

Figure S9 continued. (C) Paired pre- and post-treatment plasma abundance of PSMC and PSMD subunits in the PR and N groups; nominal P values are shown.

C. PSMC-PSMD

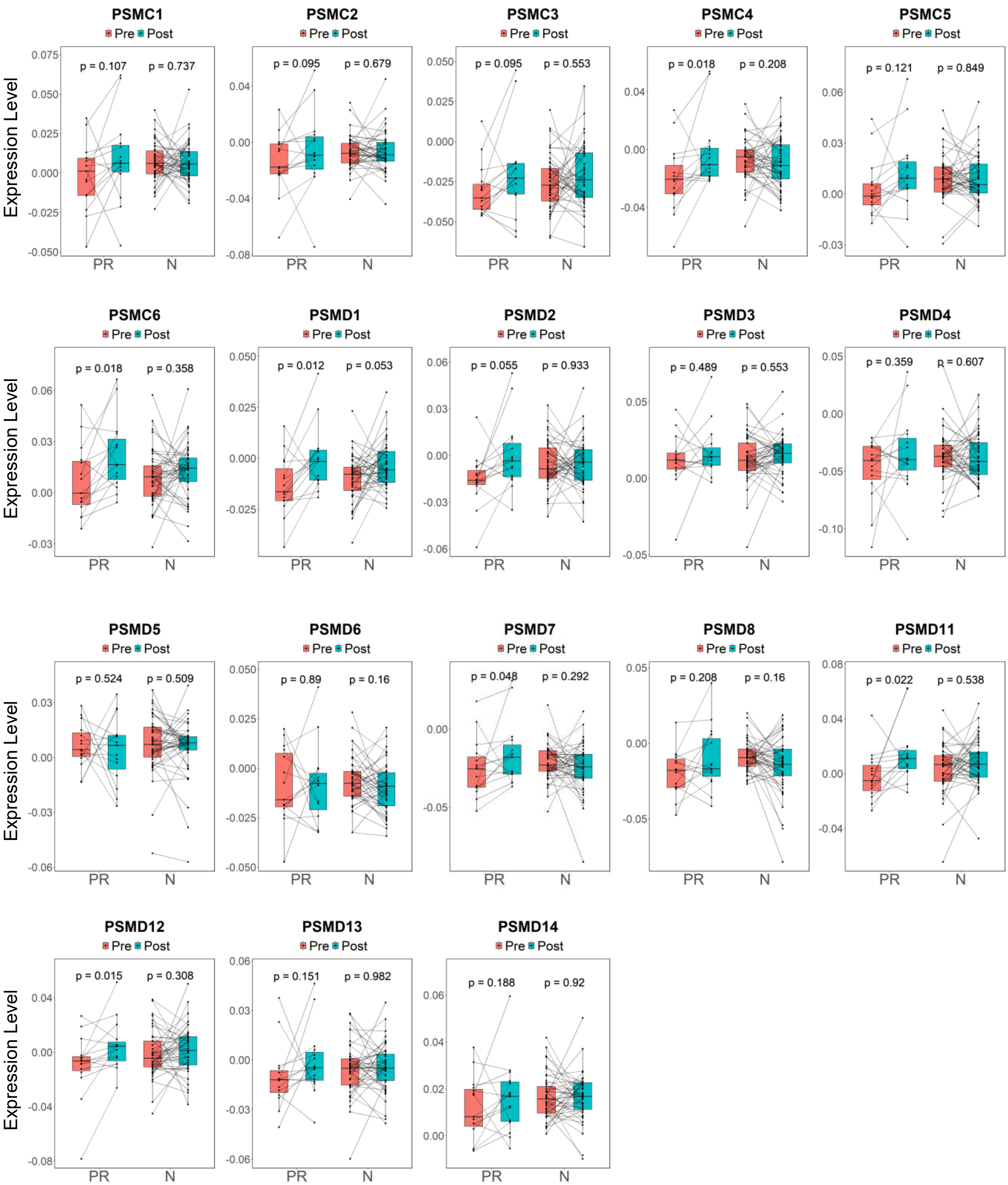

Figure S10. Pairwise correlations among proteasome subunits in TCGA PAAD tissue (blue) and the local plasma cohort (green). Circle size indicates |r|, and color indicates -log10(P).

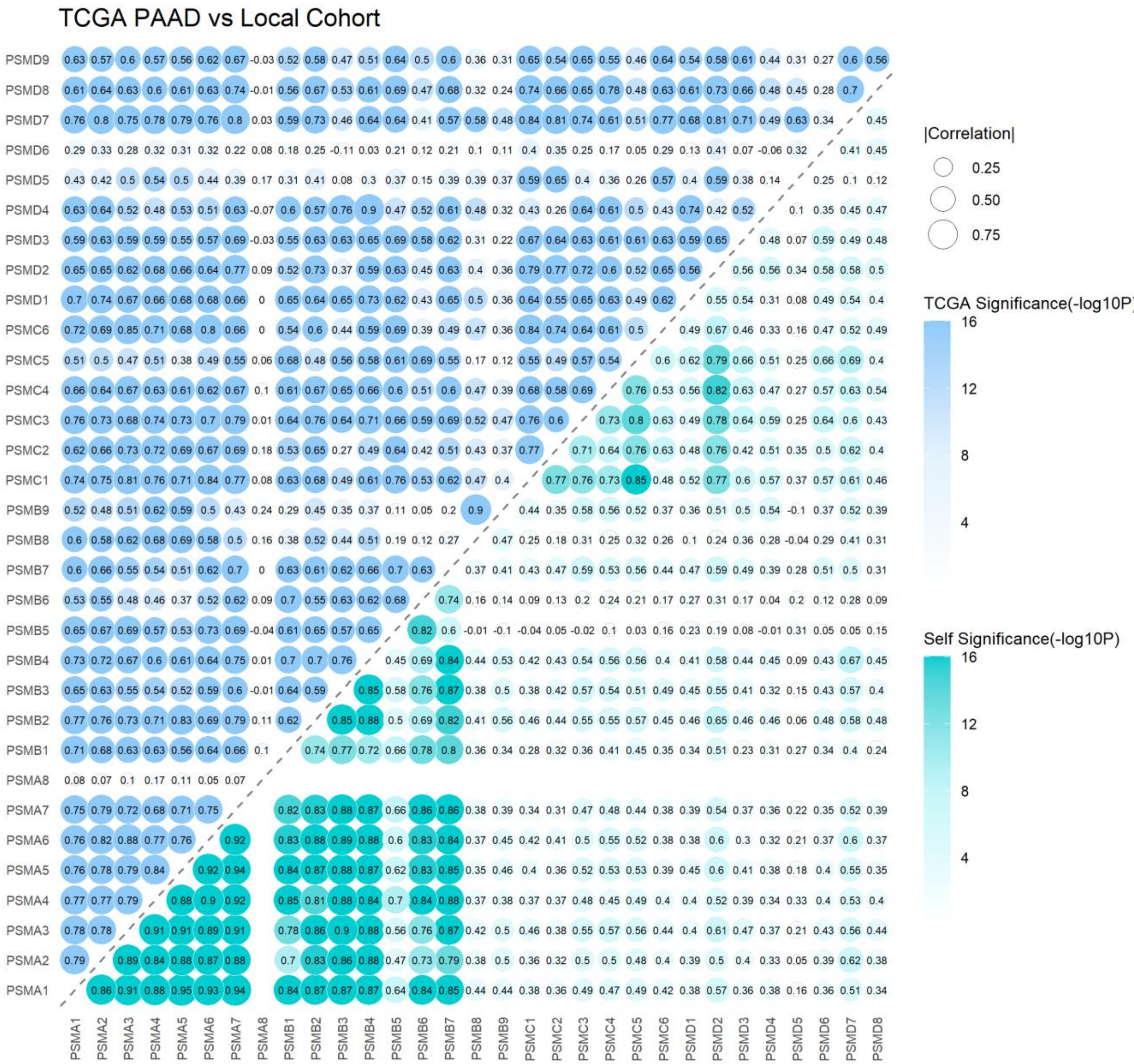

Figure S11. Proteasome-subunit expression in TCGA PAAD tumor and normal pancreatic tissues. Boxes show the distribution of log2-transformed TPM values.

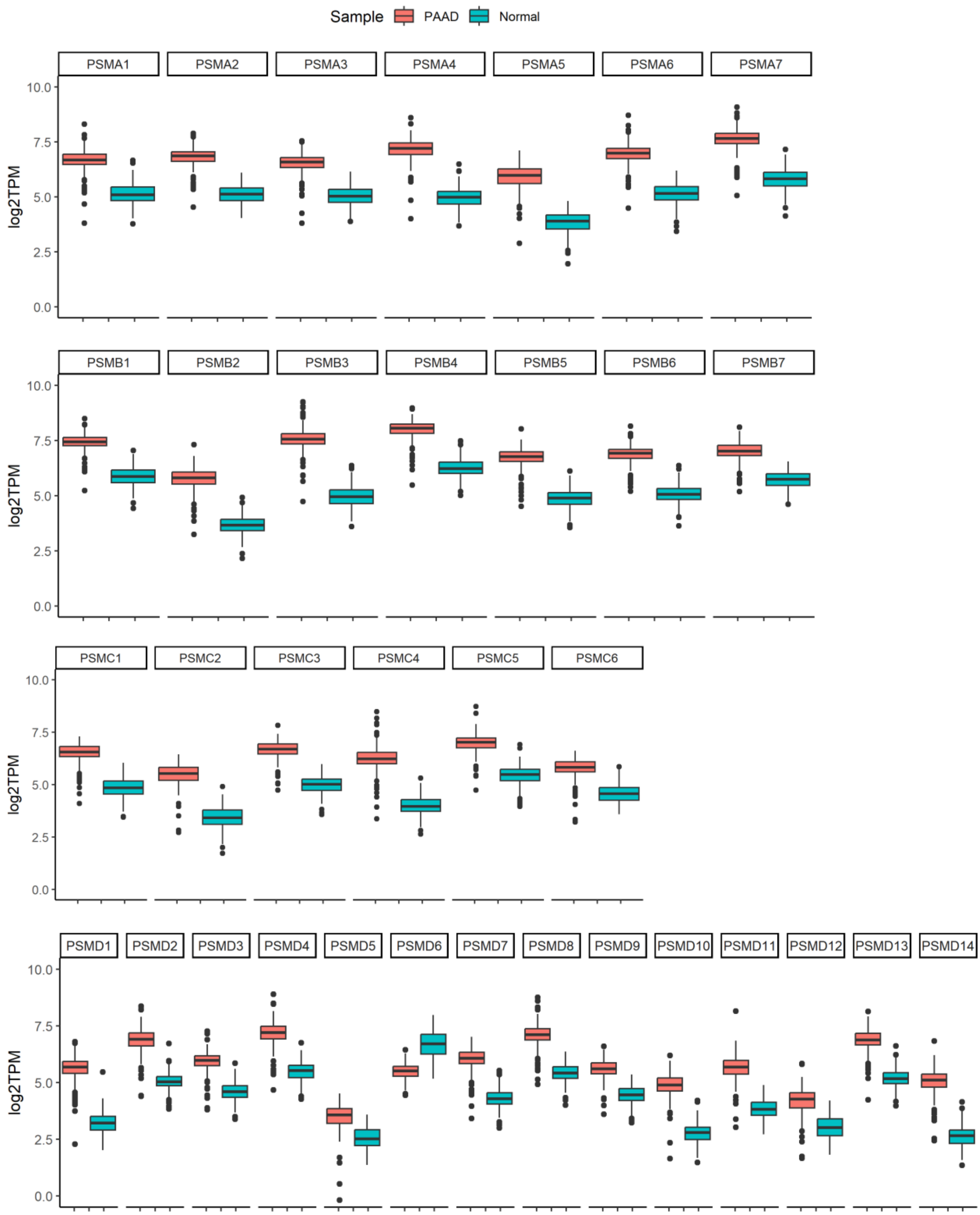

Figure S12. Exploratory survival analyses of proteasome-related features. (A) Overall survival stratified by median within-patient changes in plasma PSM subunits in the discovery cohort. (B) Overall survival stratified by median tumor-tissue PSM expression in TCGA PAAD. P values are from log-rank tests.

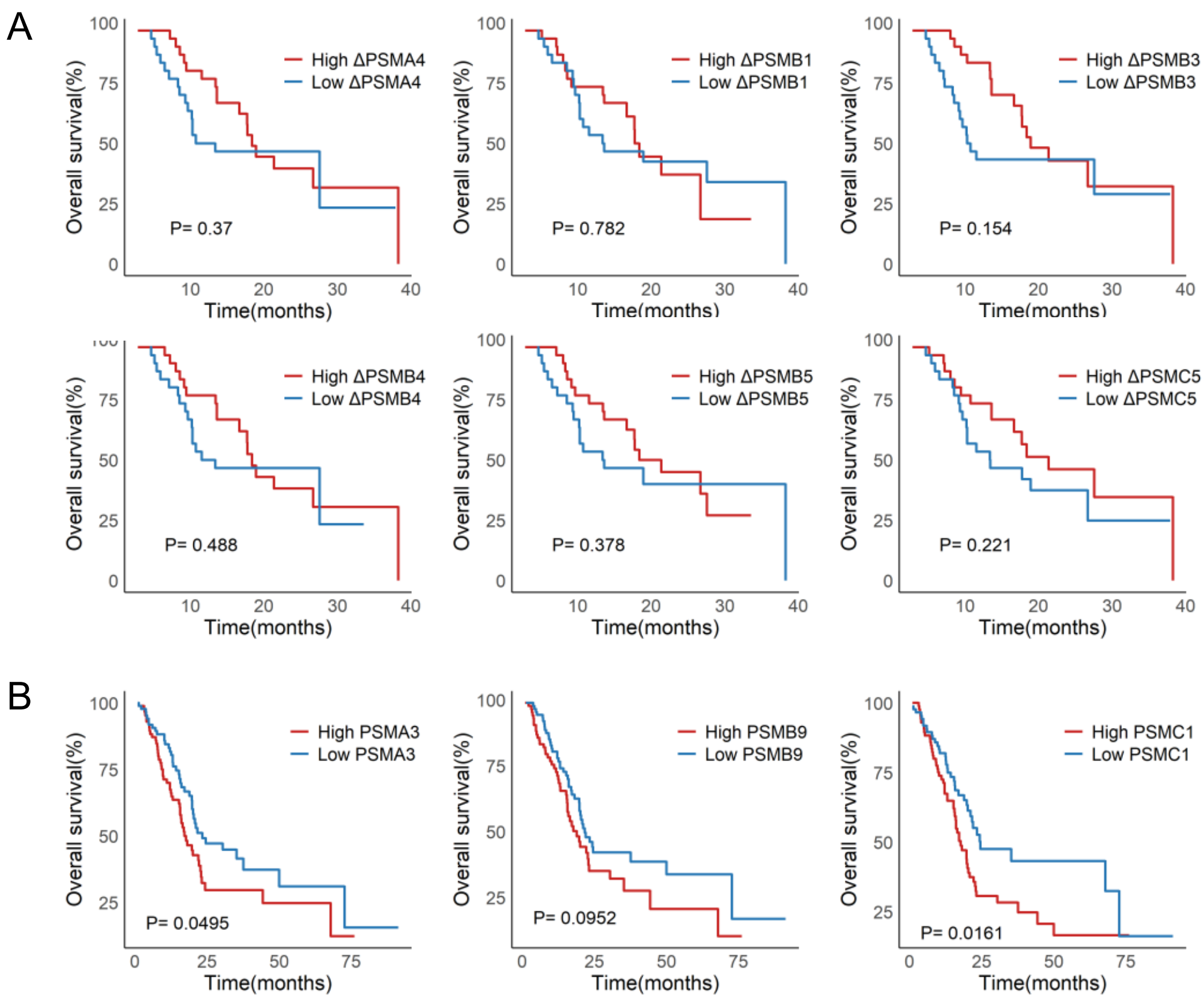

Figure S13. Exploratory WGCNA associations with PR and PD after GnP. (A) Module-eigengene correlations with response and clinical traits; the pink module showed weak correlations in opposite directions with PR and PD. (B) Pink-module eigengene distributions in PD (n = 8) and PR (n = 15); Wilcoxon P = 0.213.

A

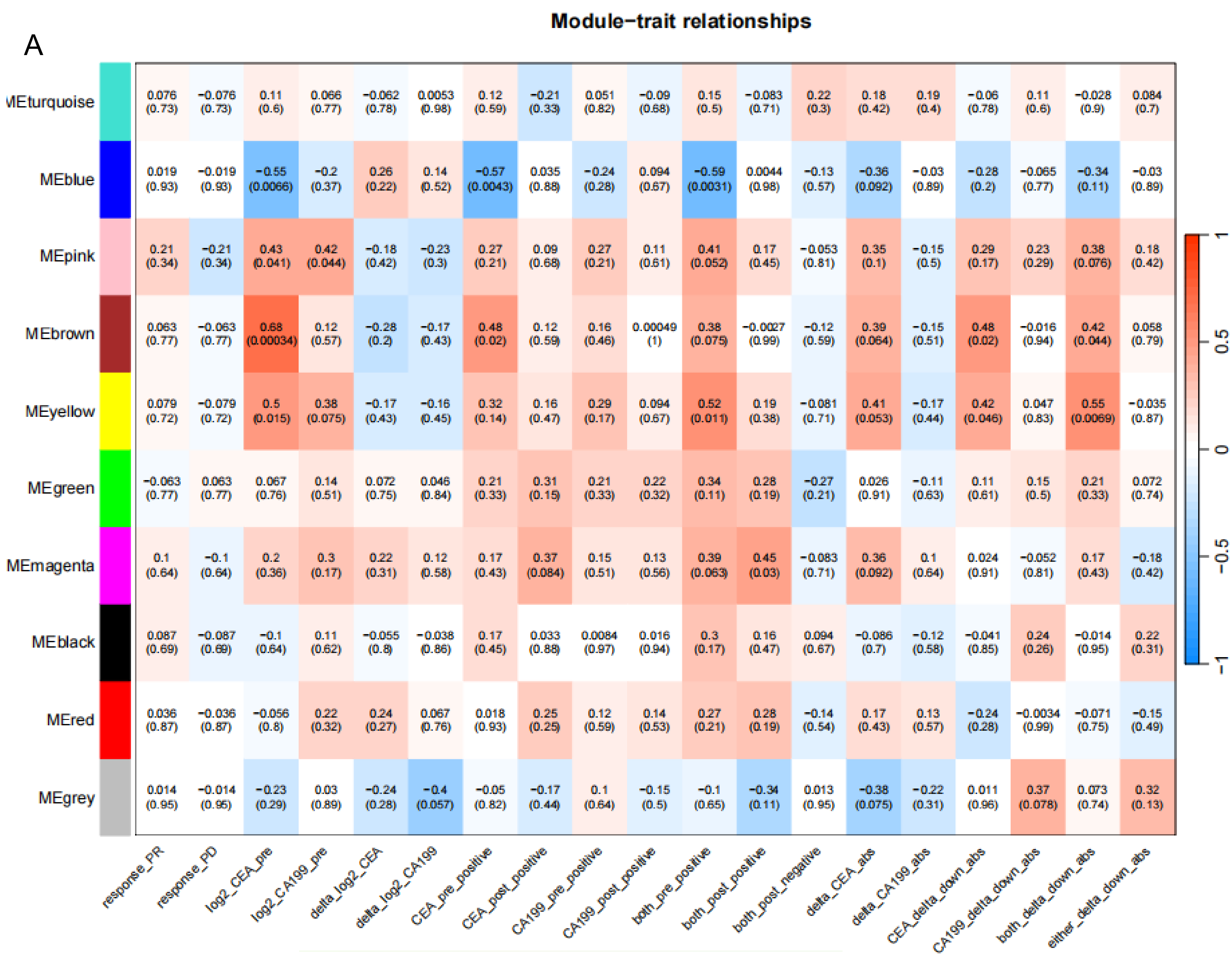

B

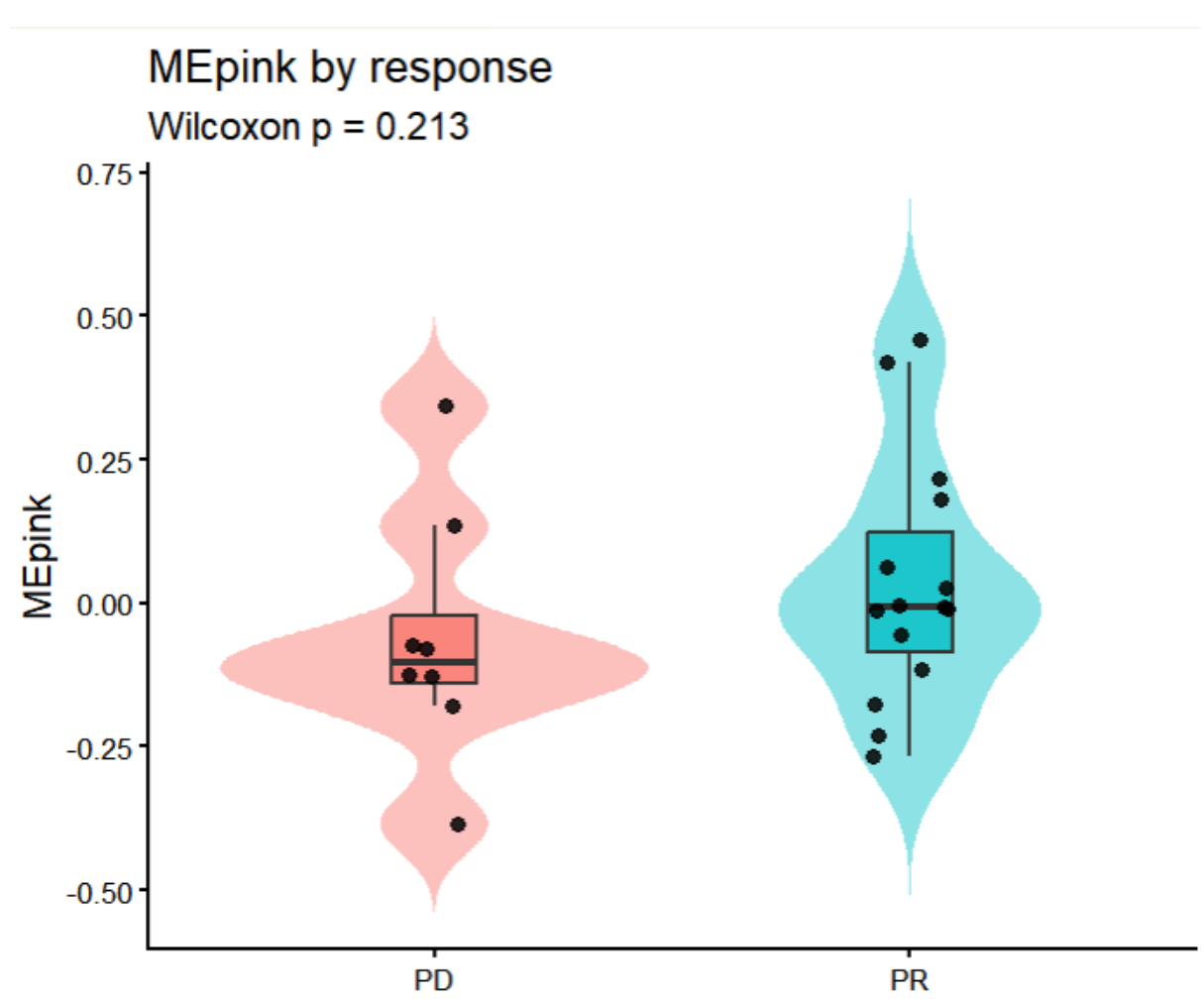

Figure S14. Functional enrichment of proteins assigned to the pink WGCNA module. (A) KEGG pathway and (B) Gene Ontology biological-process enrichment. Point size represents the number of proteins, and color represents the adjusted P value.

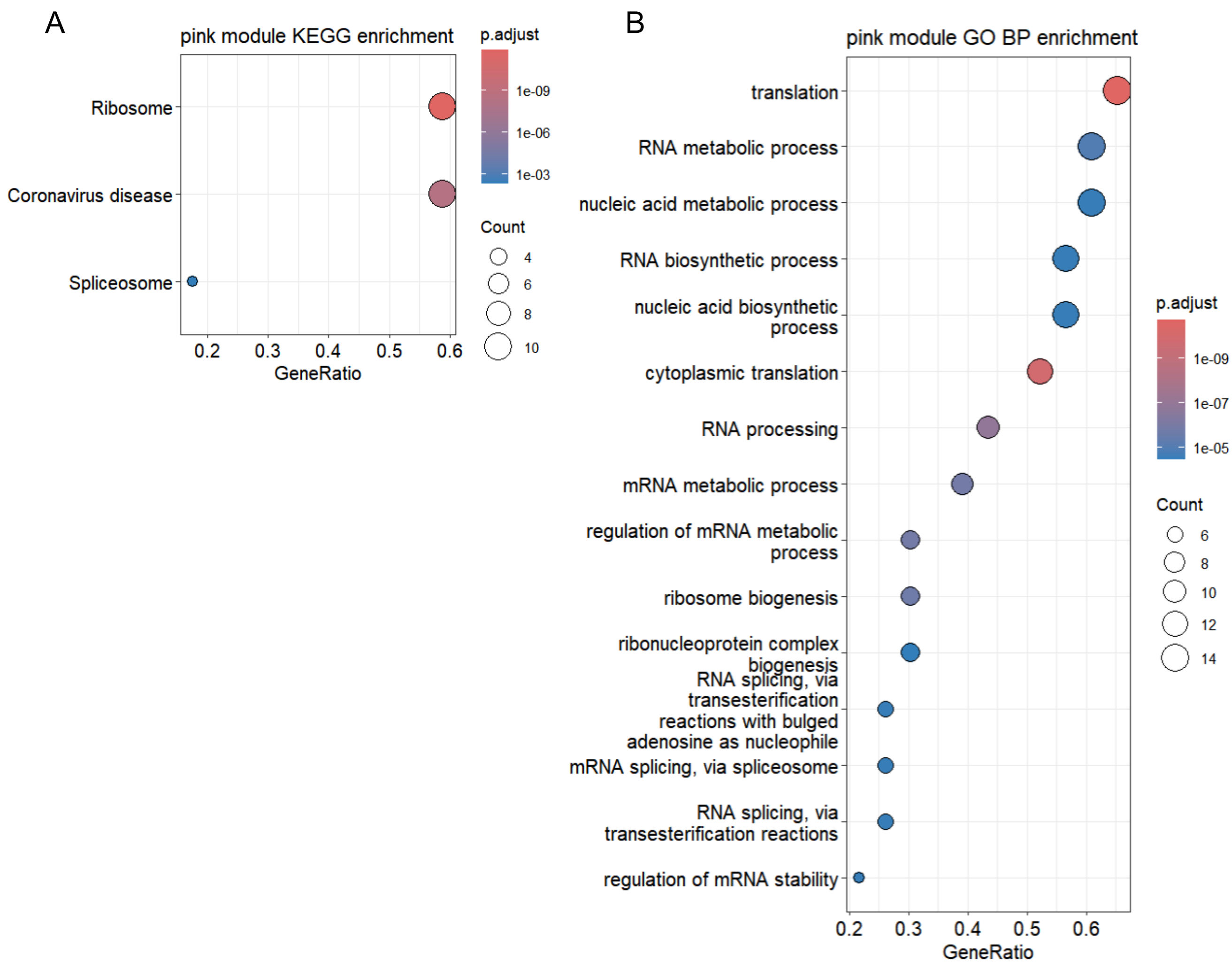

Figure S15. Exploratory overlap of plasma proteins with opposite longitudinal changes after GnP chemotherapy in PR and PD. (A) MANF was the only protein shared between PR Up (n = 344) and PD Down (n = 56). (B) No protein was shared between PR Down (n = 92) and PD Up (n = 53).

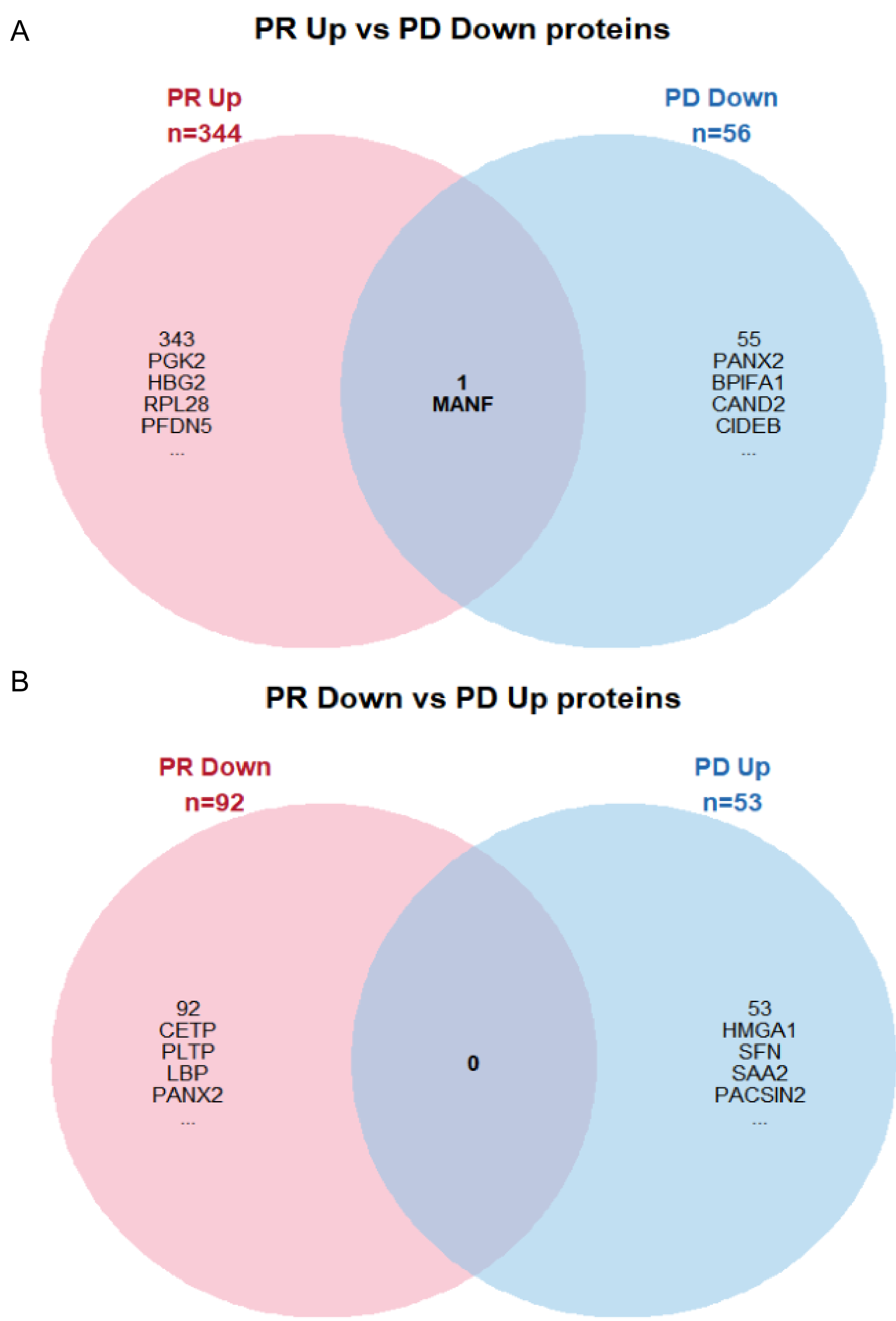

Figure S16. Untargeted plasma metabolomics. (A) Heatmap and (B) enrichment of nominal PR-versus-N changes. Enrichment reflects circulating metabolite annotations.

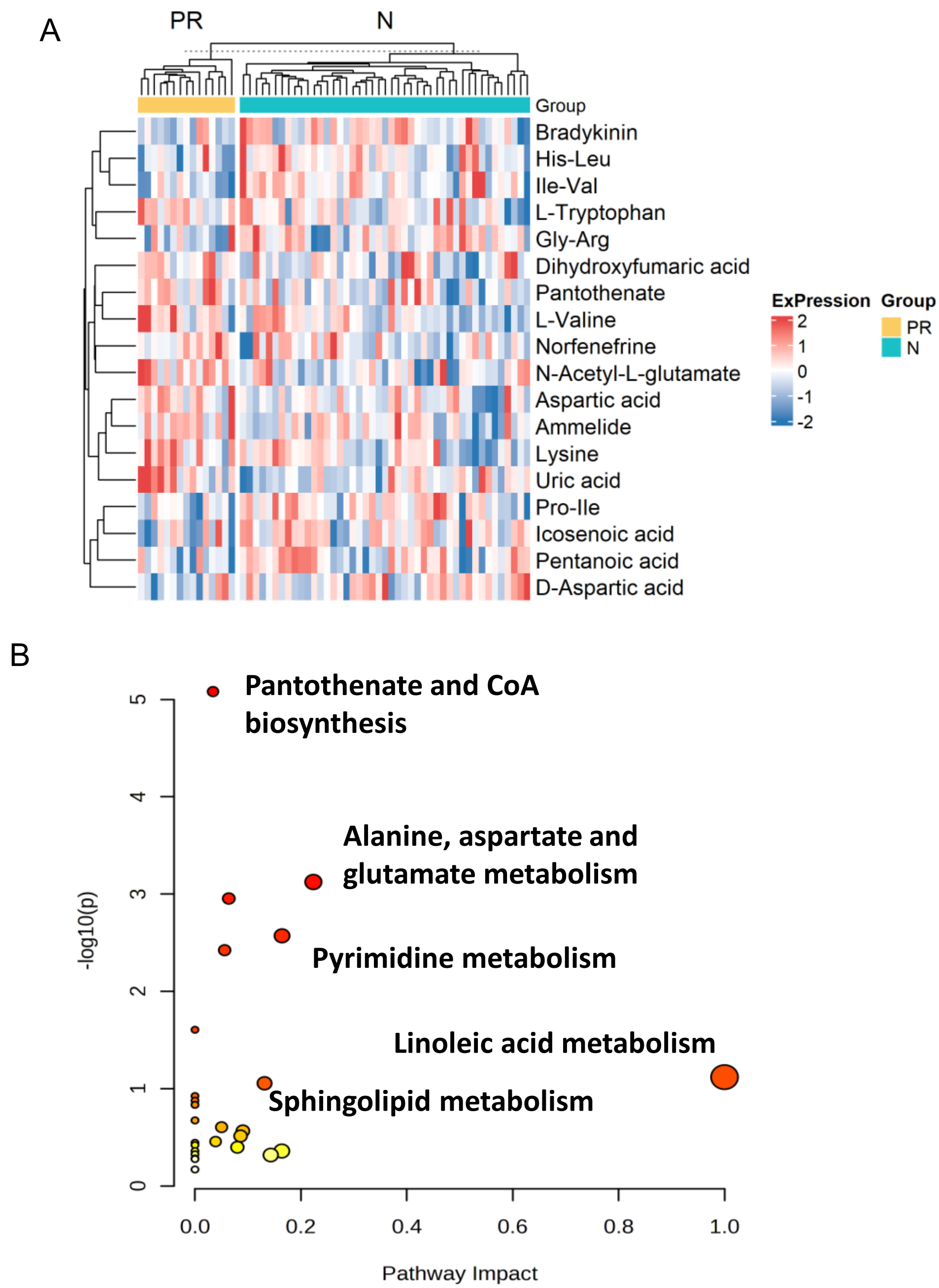

Figure S17. Exploratory assessment of N-glycopeptide candidates and the glyco-score. (A) Abundance of ten of the eleven stable candidates across PR and N groups with ORM2 shown in Figure 7D; nominal P values are shown. (B) Multivariable Cox regression including the median-dichotomized glyco-score and clinical covariates. Points indicate hazard ratios and horizontal lines indicate 95% confidence intervals.

A

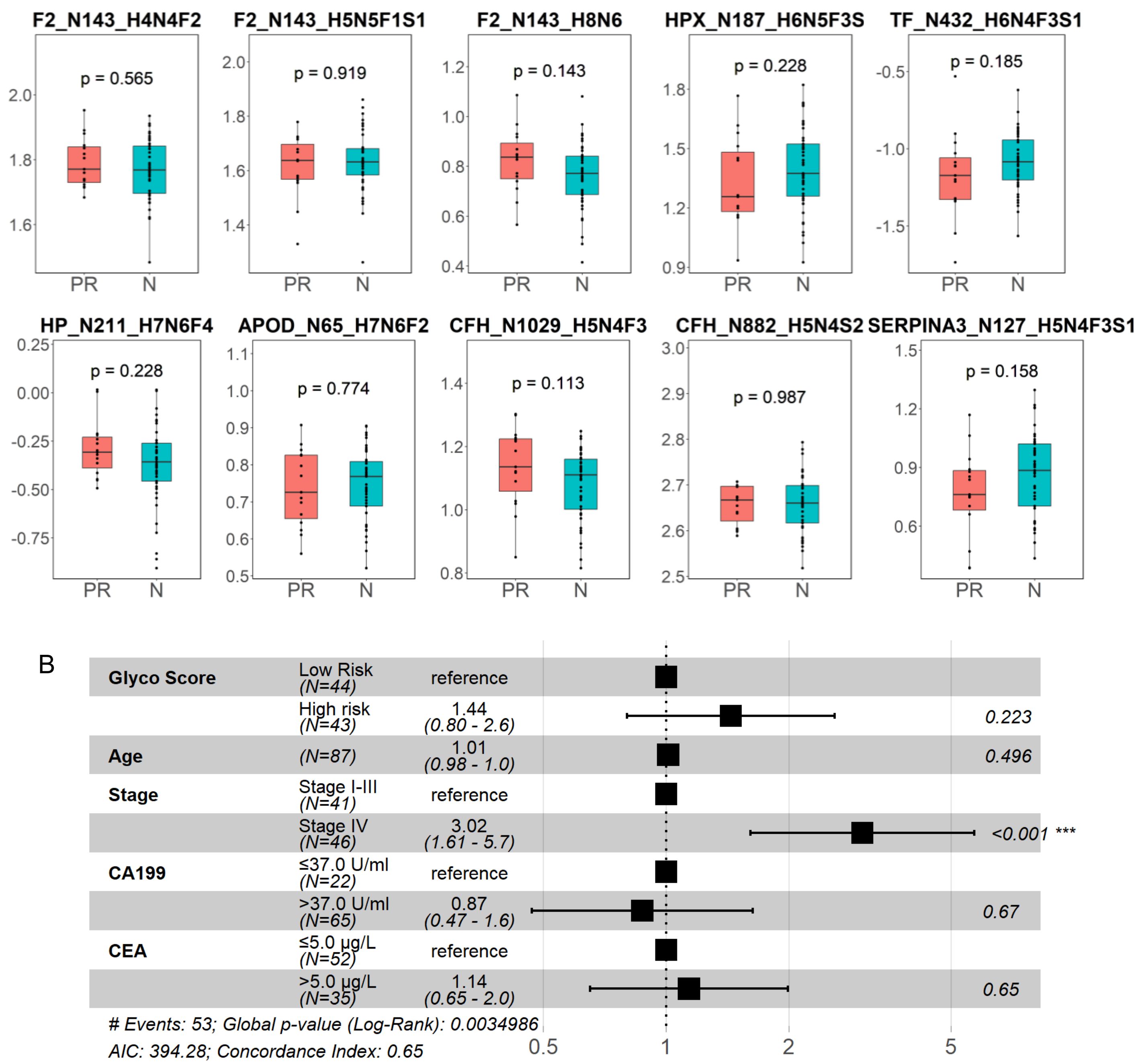

Figure S18. Exploratory performance of the plasma protein risk model. (A) Kaplan-Meier curves based on the cross-validated protein-derived risk score, dichotomized at the median; the log-rank P value is shown. (B) Time-dependent ROC curves for overall survival at 12, 18, and 24 months; AUC values are shown.

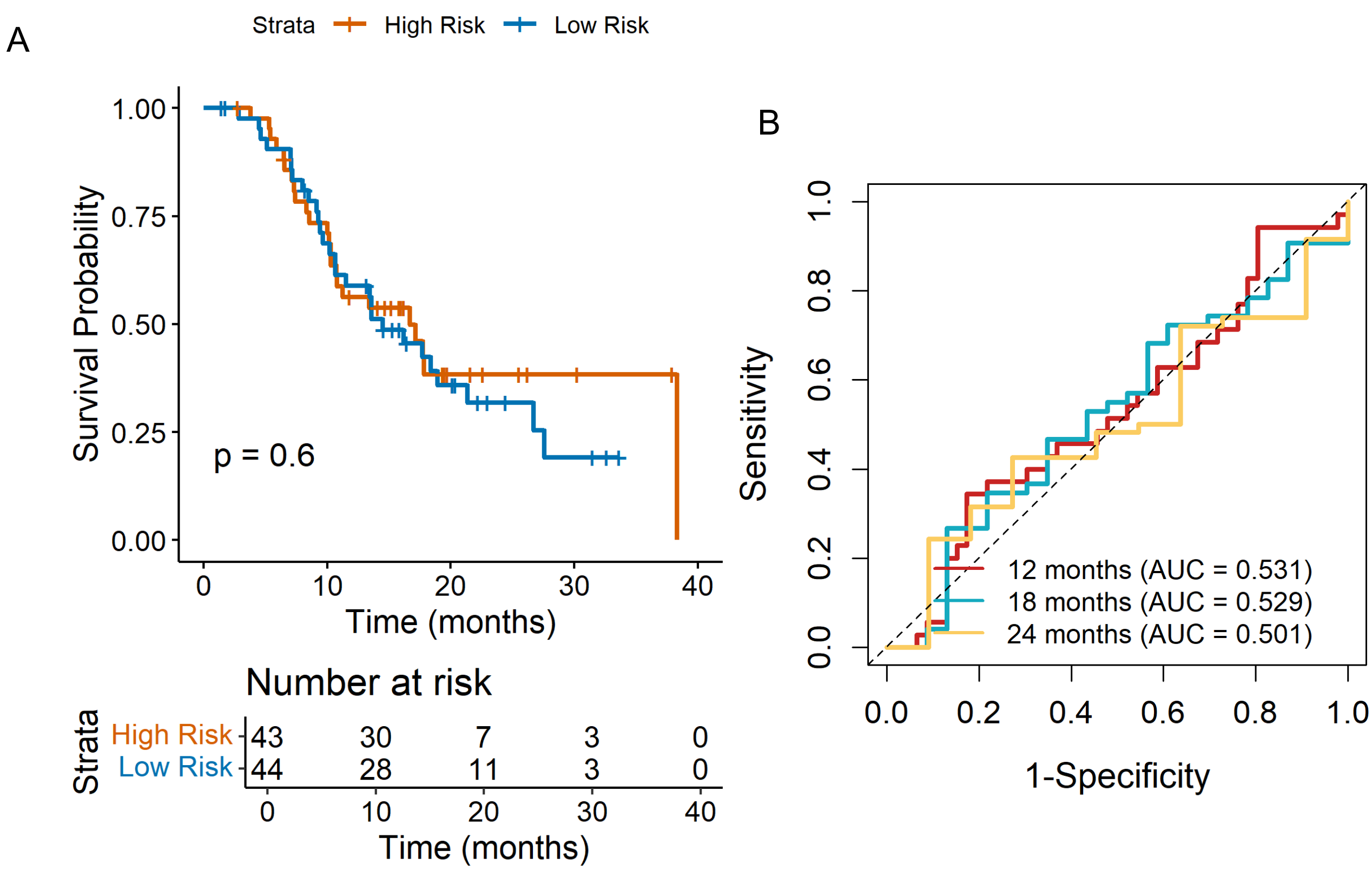

Supplement: Supplementary file 1 [file mmc13.pdf]
