# Supplementary material for: Dynamic Alterations of Site-Specific N-Glycoform and Plasma Proteasome Signatures Stratify Chemotherapy Benefit in Pancreatic Ductal Adenocarcinoma
Source: Mol Cell Proteomics. 2026 Aug 17;25(9):101642. doi: 10.1016/j.mcpro.2026.101642 (PMC13580066; doi:10.1016/j.mcpro.2026.101642)
Supplement: Supplementary file 2 [file mmc14.pdf]

# A0A075B6I9

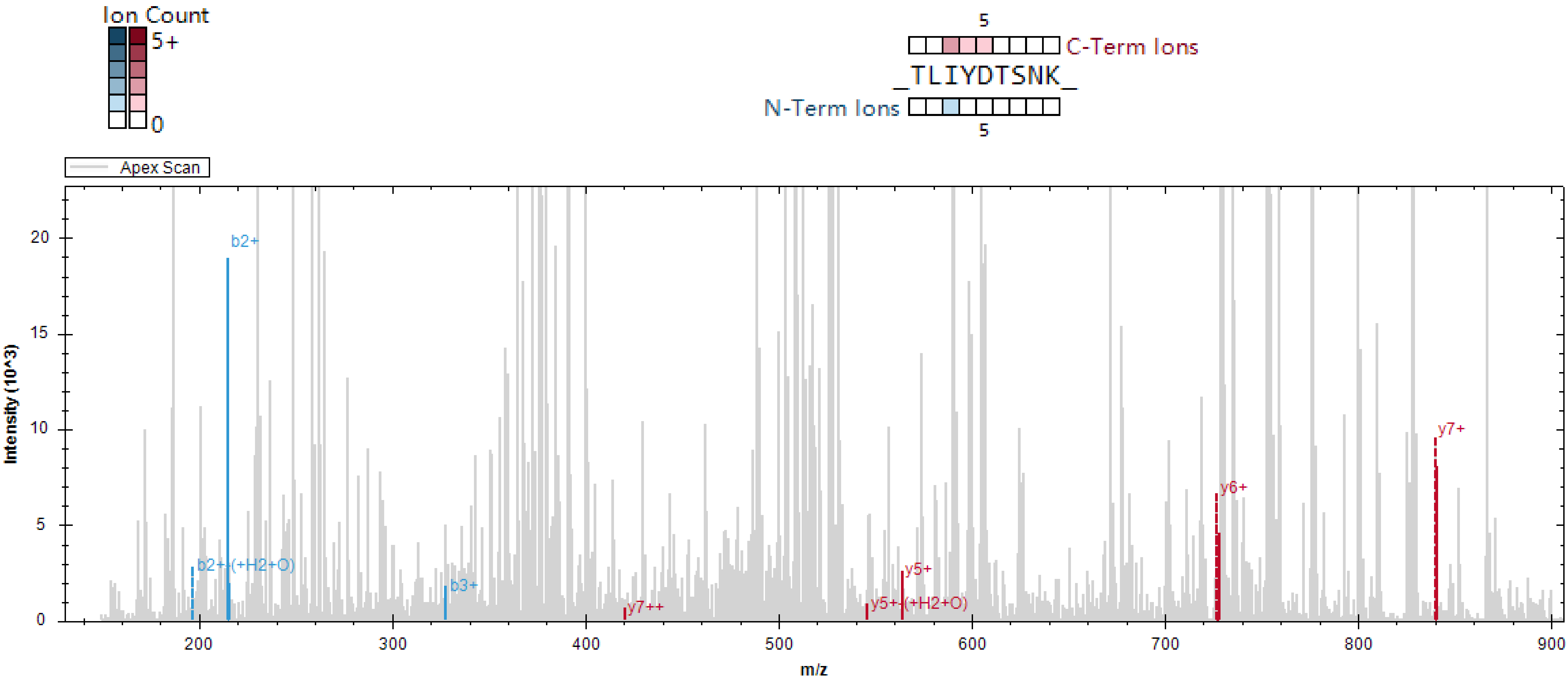

Ion Count
5+
0
5
C-Term Ions
_TLIYDTSNK_
N-Term Ions
5
Apex Scan
b2+
b2+-(+H2+O)
b3+
y7++
y5+-(+H2+O)
y5+
y6+
y7+
Intensity (10^3)
0
5
10
15
20
200
300
400
500
600
700
800
900
m/z

# A0A087WSY6

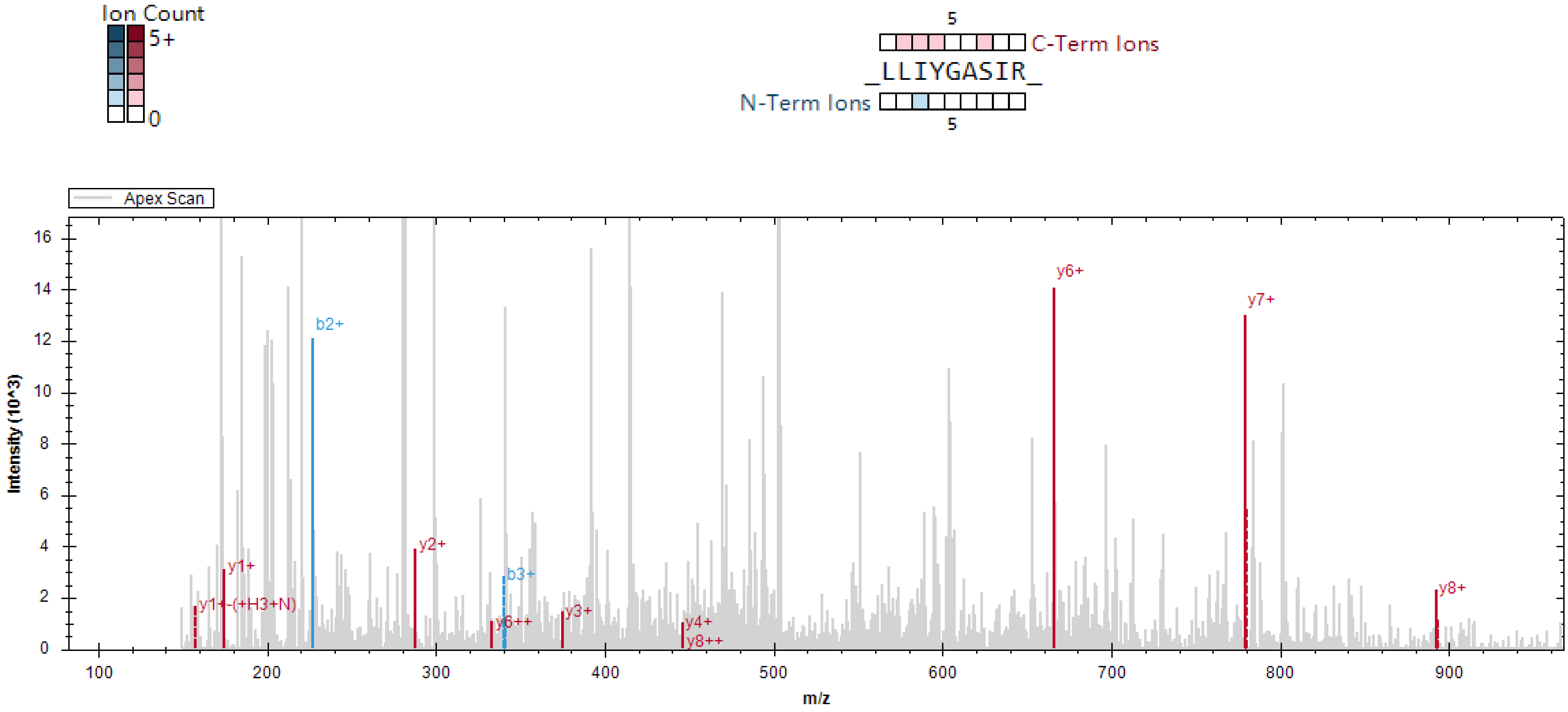

Ion Count
5+
0
5
C-Term Ions
_LLIYGASIR_
N-Term Ions
5
Apex Scan
Intensity (10^3)
m/z
b2+
b3+
y1+
y1+-(+H3+N)
y2+
y3+
y4+
y6+
y6++
y7+
y8+
y8++

# A0A0A0MT89

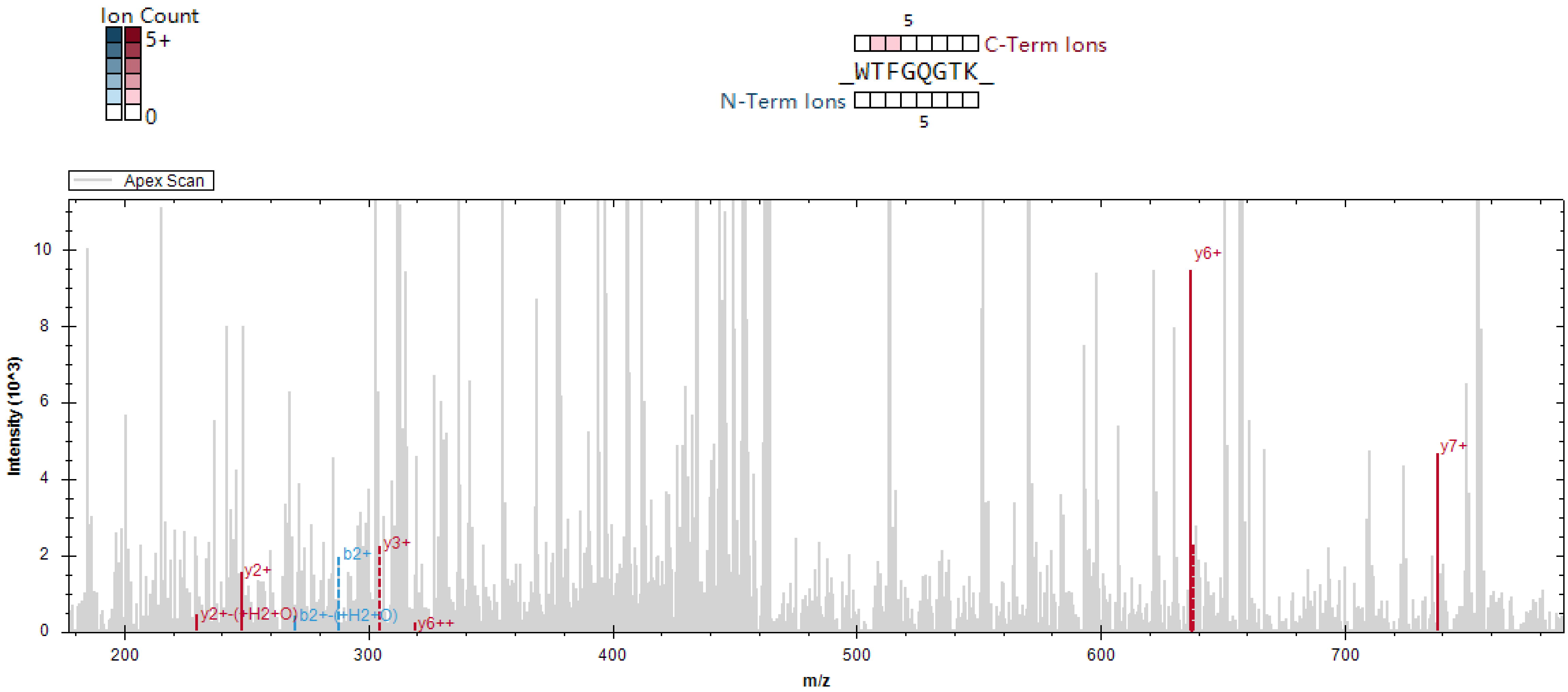

Ion Count
5+
0
5
C-Term Ions
_WTFGQGTK_
N-Term Ions
5
Apex Scan
Intensity (10^3)
m/z
y6+
y7+
y3+
b2+
y2+
y2+-(+H2+O)
b2+-(+H2+O)
y6++

# A0A1B0GTR0

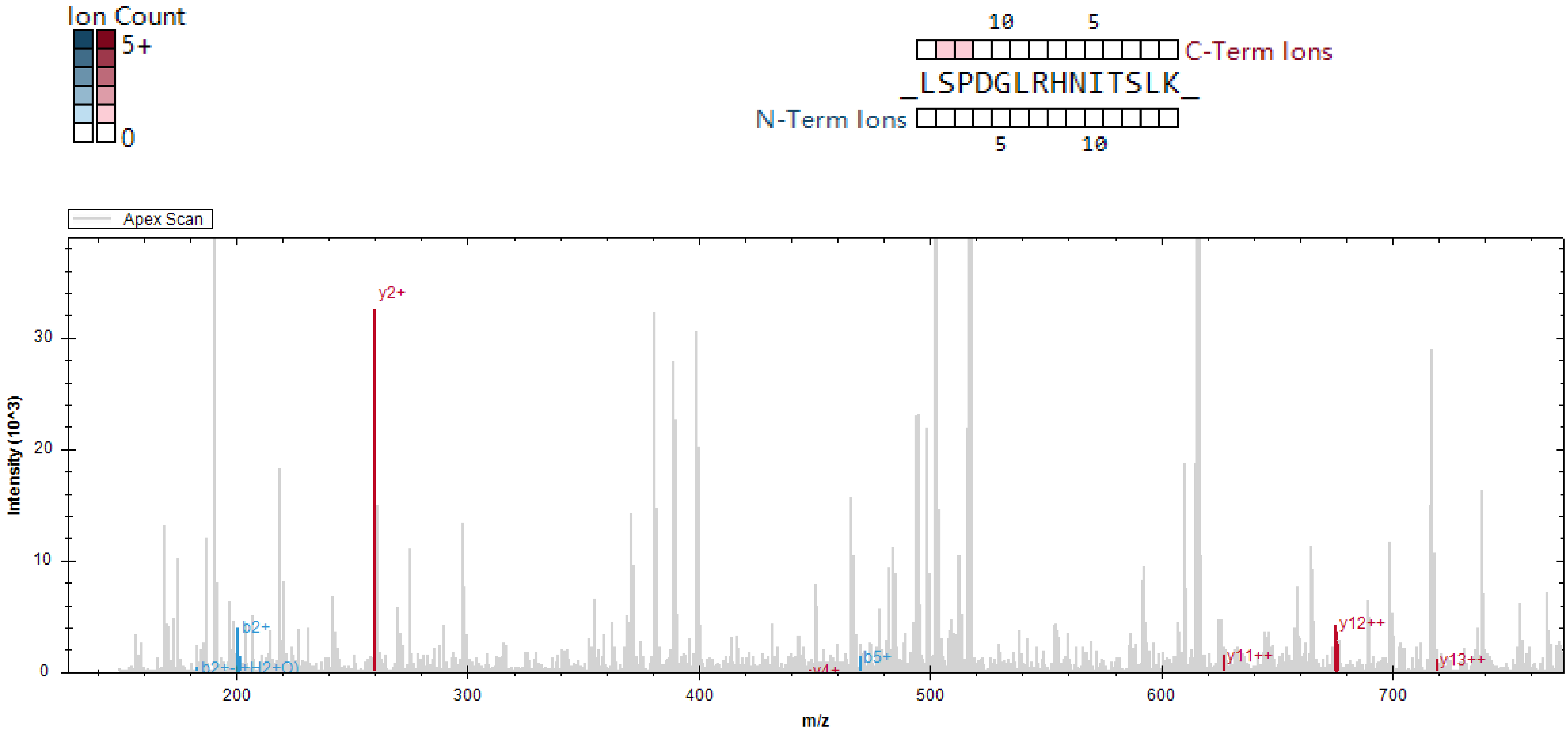

Ion Count
5+
0
10
5
C-Term Ions
_LSPDGLRHNITSLK_
N-Term Ions
5
10
Apex Scan
y2+
b2+
b5+
y11++
y12++
y13++
Intensity (10^3)
0
10
20
30
200
300
400
500
600
700
m/z

# A6NC98

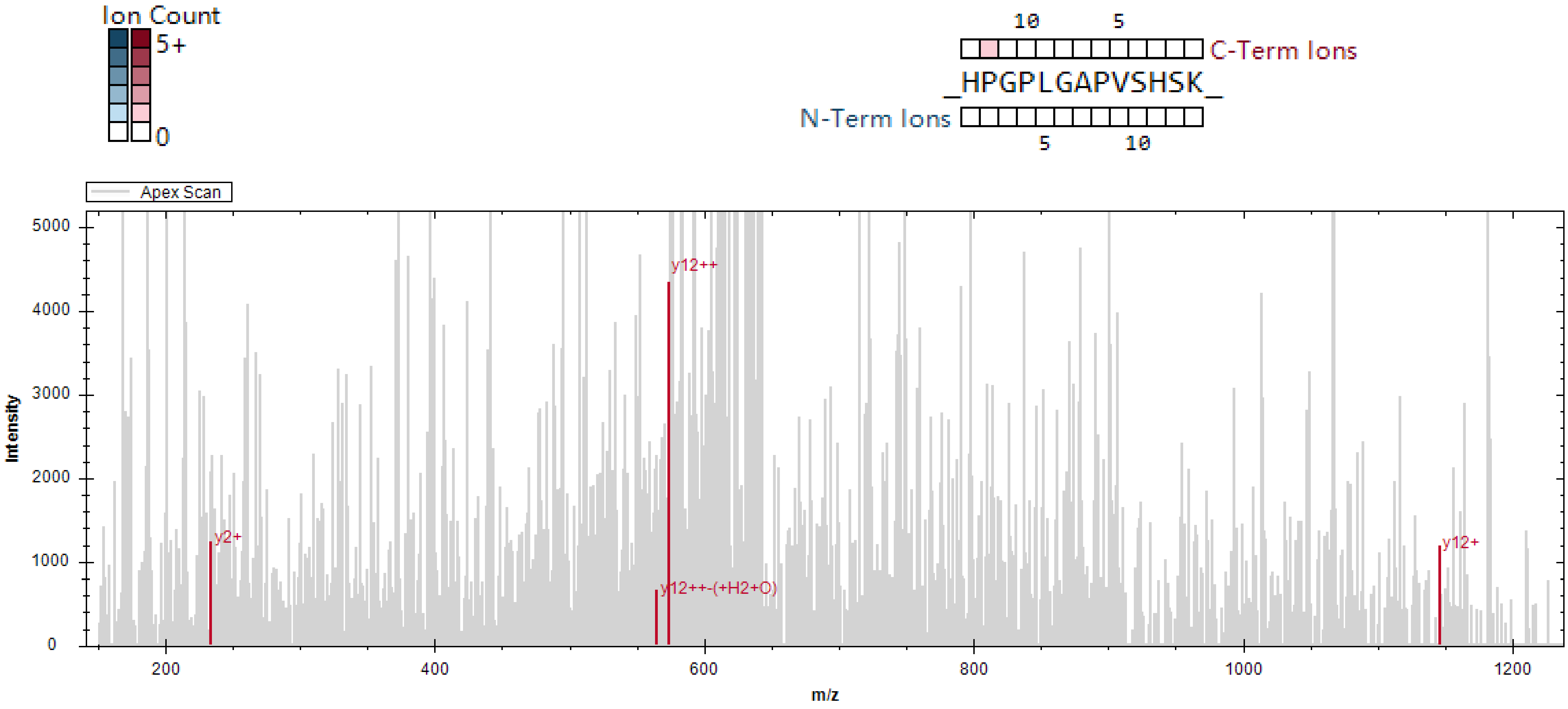

Ion Count
5+
0
10
5
C-Term Ions
_HPGPLGAPVSHSK_
N-Term Ions
5
10
Apex Scan
y12++
y2+
y12++-(+H2+O)
y12+
Intensity
5000
4000
3000
2000
1000
0
200
400
600
800
1000
1200
m/z

# A6NIK2

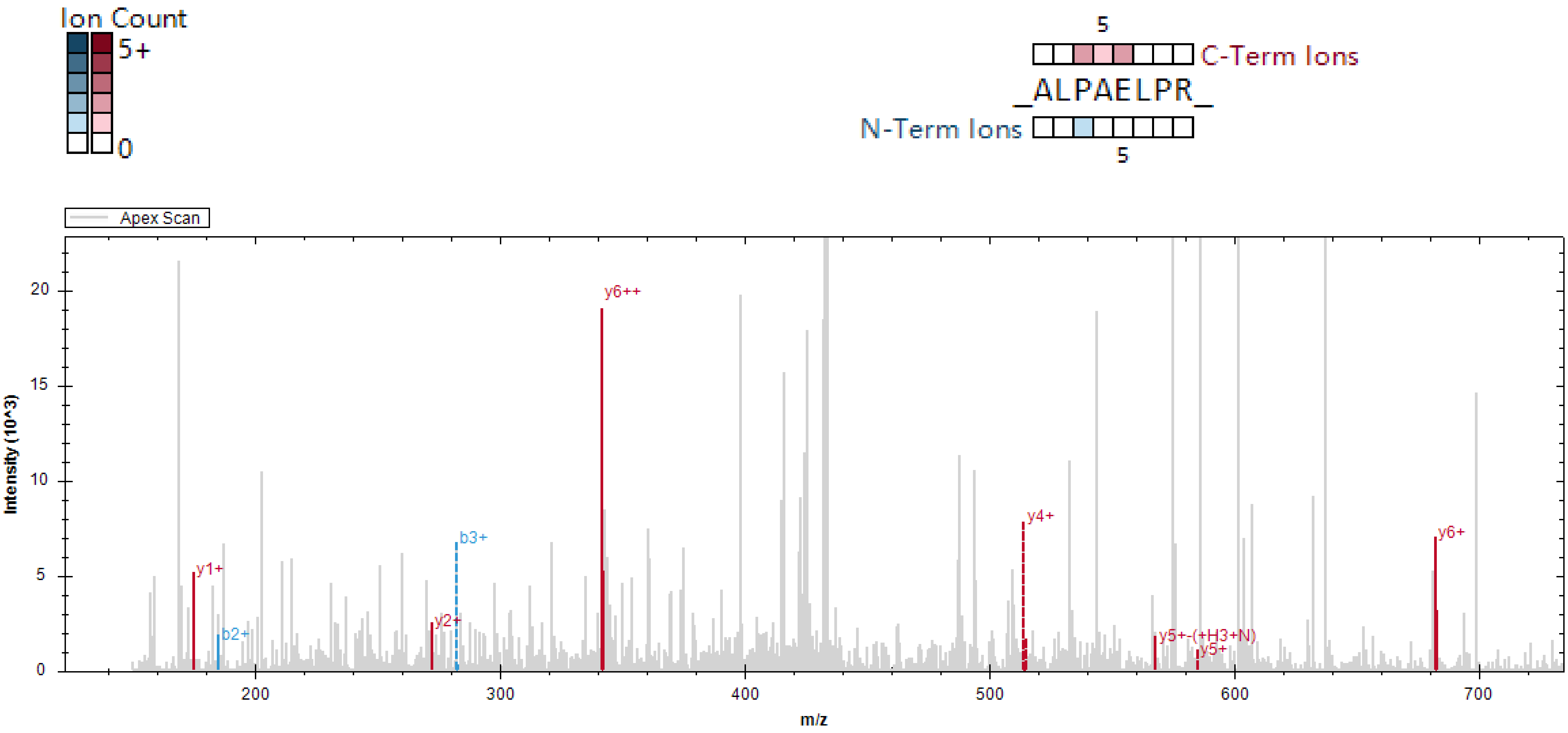

Ion Count
5+
0
5
C-Term Ions
_ALPAELPR_
N-Term Ions
5
Apex Scan
y6++
y1+
b2+
y2+
b3+
y4+
y5+-(+H3+N)
y5+
y6+
Intensity (10^3)
m/z
0
5
10
15
20
200
300
400
500
600
700

# A8MY62

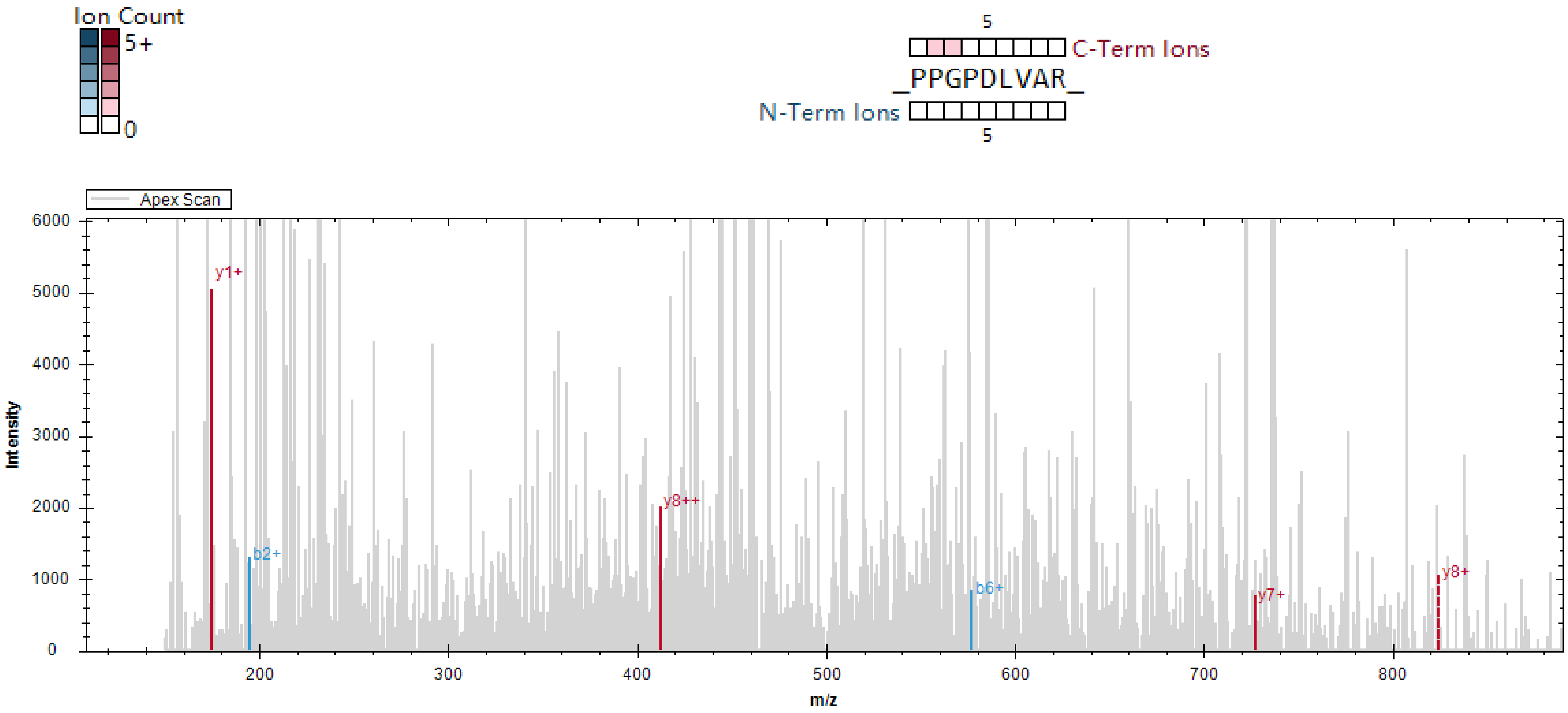

Ion Count
5+
0
5
C-Term Ions
_PPGPDLVAR_
N-Term Ions
5
Apex Scan
6000
5000
4000
3000
2000
1000
0
Intensity
y1+
b2+
y8++
b6+
y7+
y8+
200
300
400
500
600
700
800
m/z

# B1ANS9

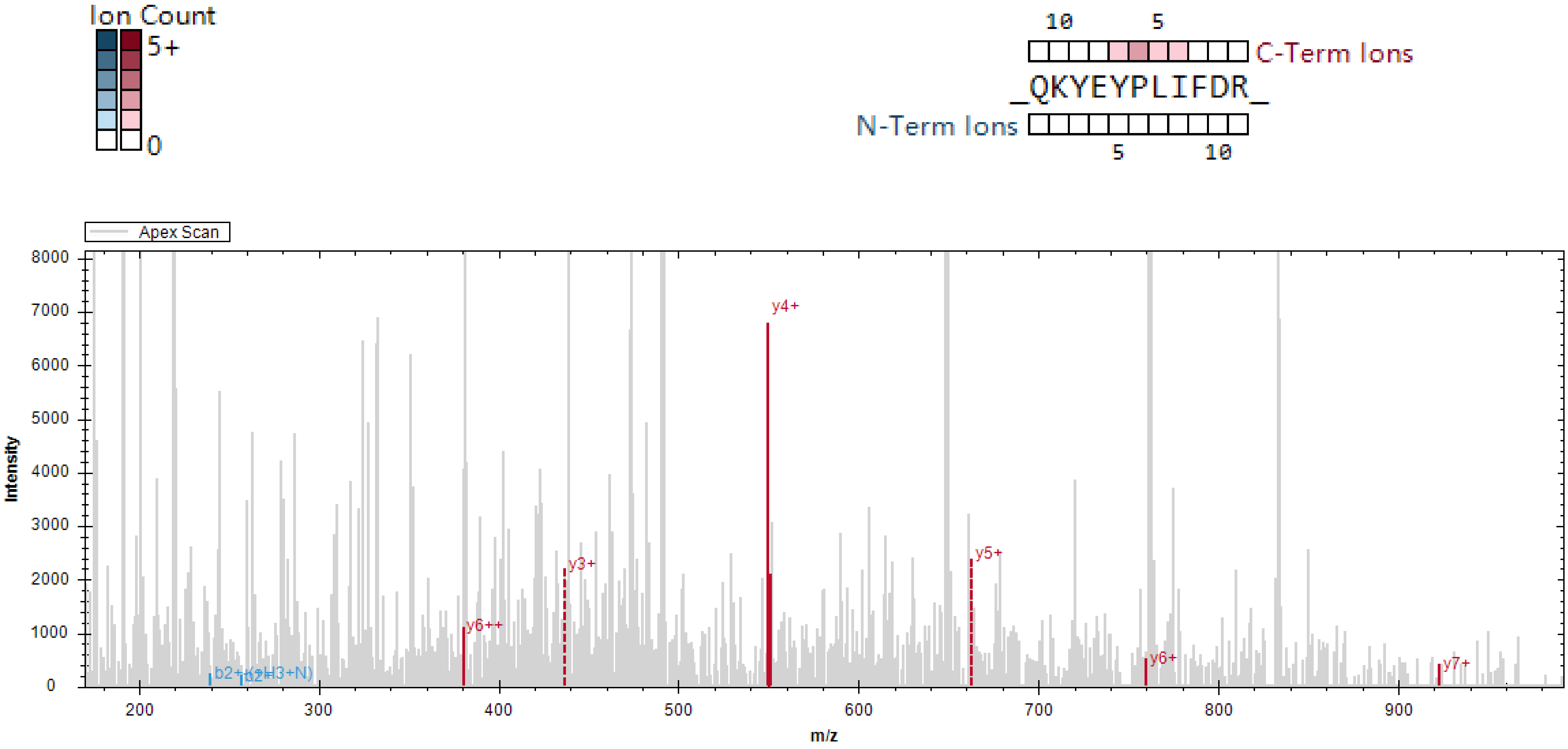

Ion Count
5+
0
10
5
C-Term Ions
_QKYEYPLIFDR_
N-Term Ions
5
10
Apex Scan
Intensity
m/z
y4+
y3+
y5+
y6++
y6+
y7+
b2+

# C9JRZ8

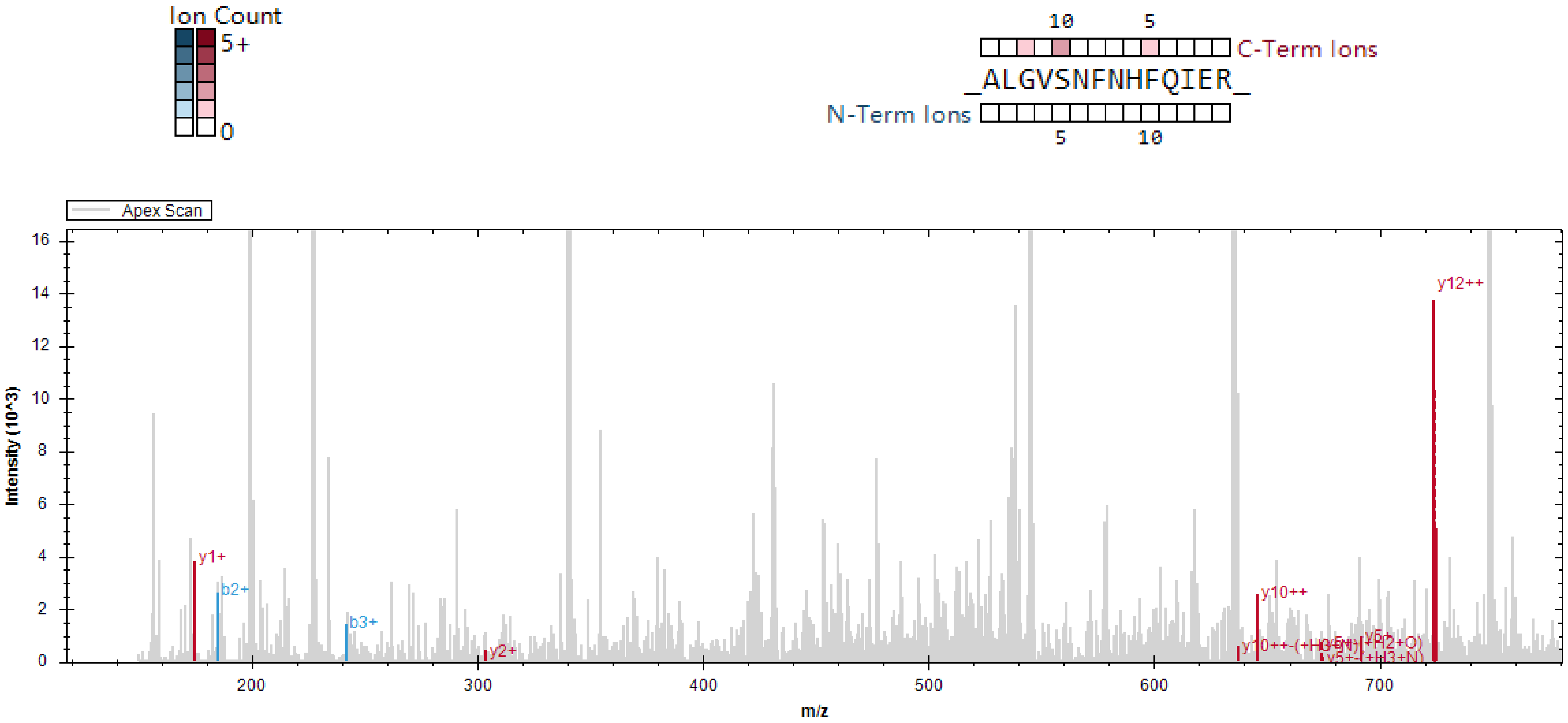

Ion Count
5+
0
10
5
C-Term Ions
_ALGVSNFNHFQIER_
N-Term Ions
5
10
Apex Scan
Intensity (10^3)
m/z
y12++
y1+
b2+
b3+
y2+
y10++

O00425

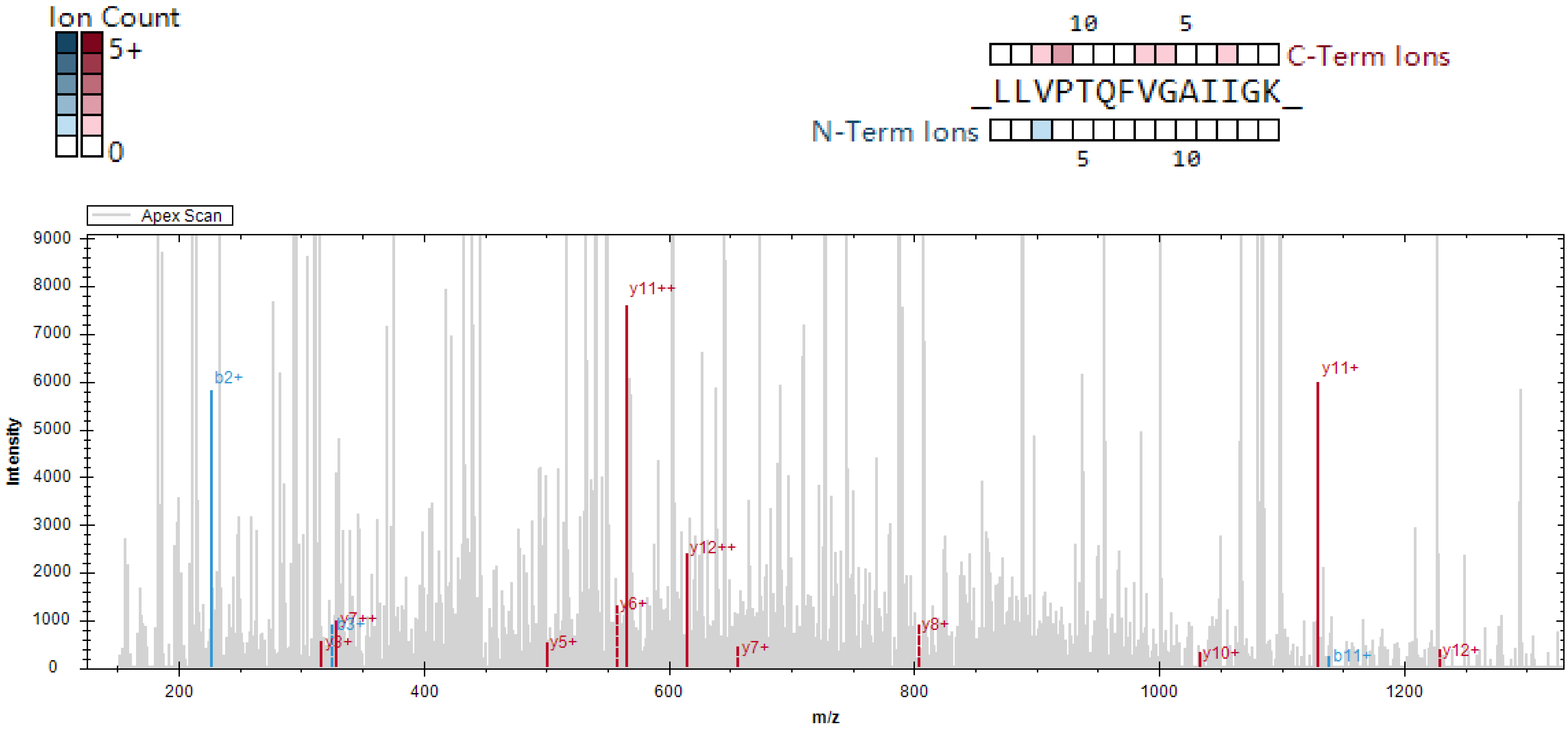

Ion Count
5+
0
10
5
C-Term Ions
_LLVPTQFVGAIIGK_
N-Term Ions
5
10
Apex Scan
Intensity
m/z
b2+
y11++
y11+
y12++
y6+
y7++
b3+
y3+
y5+
y7+
y8+
y10+
b11+
y12+

# O00562

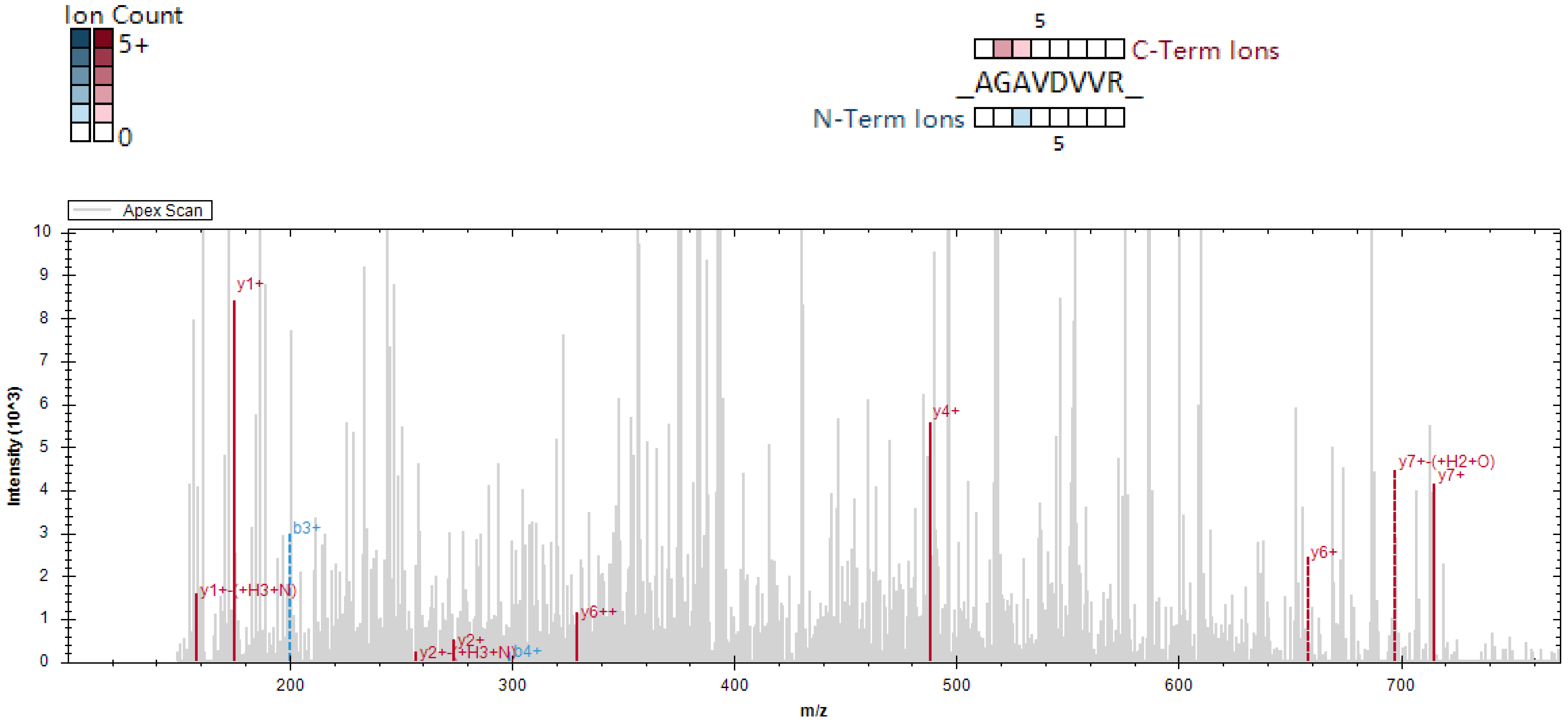

Ion Count
5+
0
5
C-Term Ions
_AGAVDVVR_
N-Term Ions
5
Apex Scan
Intensity (10^3)
m/z
y1+
y1+-(+H3+N)
b3+
y2+-(+H3+N)
y2+
b4+
y6++
y4+
y6+
y7+-(+H2+O)
y7+

# O14569

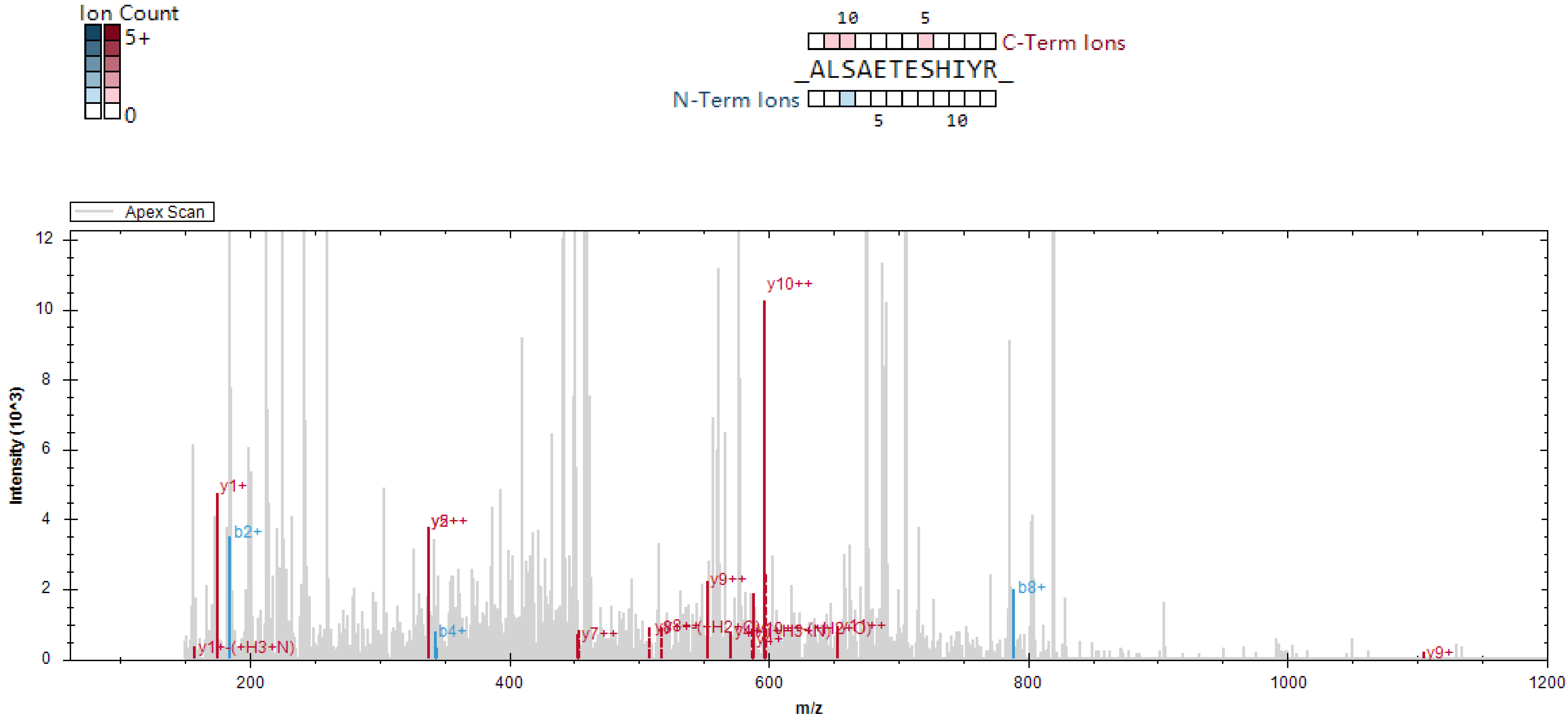
Ion Count
5+
0
10
5
C-Term Ions
_ALSAETESHIYR_
N-Term Ions
5
10
Apex Scan
Intensity (10^3)
m/z
y1+
b2+
y1+-(+H3+N)
y3++
b4+
y7++
y9++
y10++
y4+
b8+
y9+

# O14733

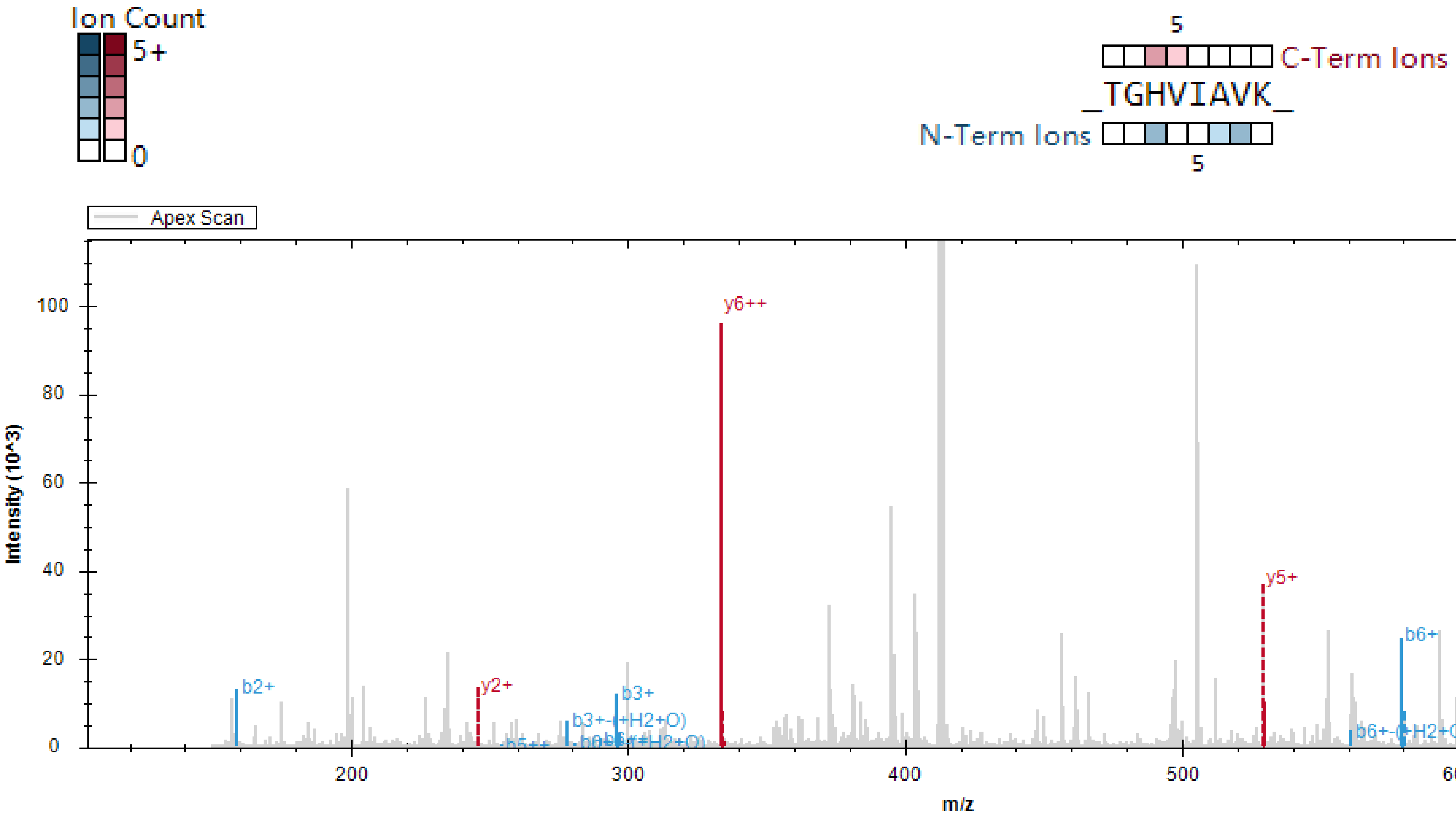

Ion Count
5+
0
5
C-Term Ions
_TGHVIAVK_
N-Term Ions
5
Apex Scan
y6++
y5+
y6+
b6+
b7+
b2+
y2+
b3+
b3+-(+H2+O)
b6+-(+H2+O)
b7+-(+H2+O)
Intensity (10^3)
m/z
0
20
40
60
80
100
200
300
400
500
600
700

# O14782

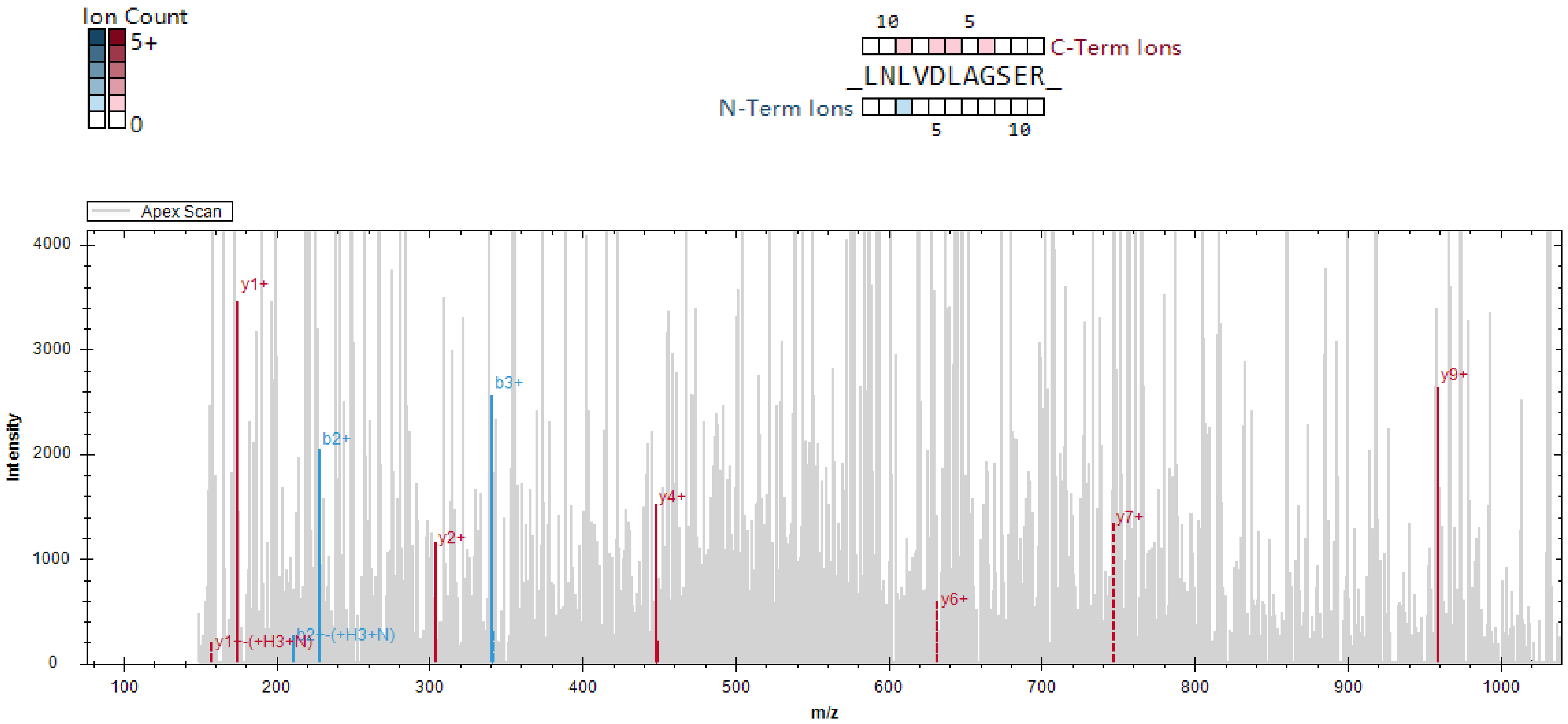
Ion Count
5+
0
10
5
C-Term Ions
_LNLVDLAGSER_
N-Term Ions
5
10
Apex Scan
Intensity
m/z
y1+
b2+
b3+
y2+
y4+
y6+
y7+
y9+
y1+-(+H3+N)
b2+-(+H3+N)

# O14817

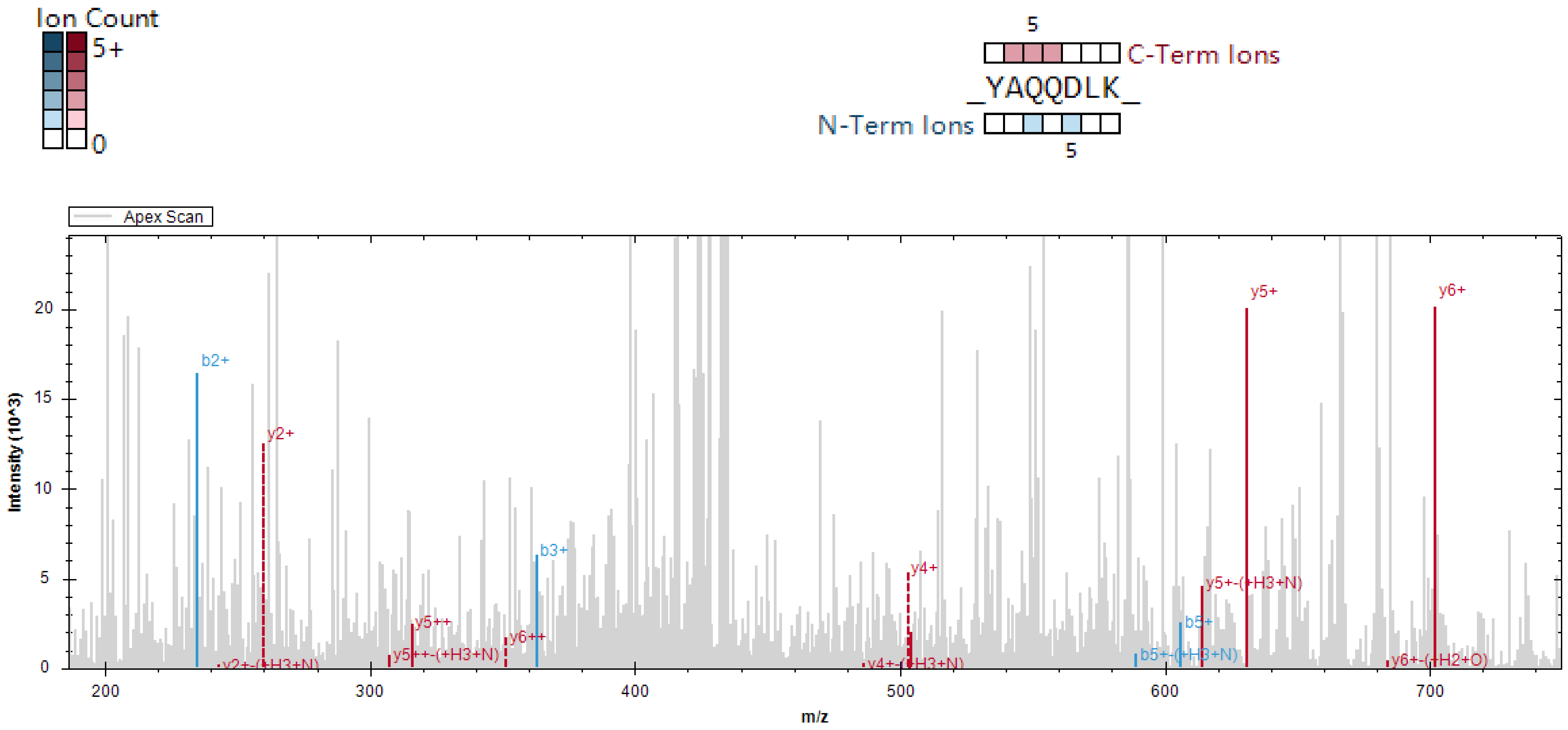

Ion Count
5+
0
5
C-Term Ions
_YAQQDLK_
N-Term Ions
5
Apex Scan
Intensity (10^3)
m/z
b2+
y2+
b3+
y5++
y6++
y4+
y5+
y6+
b5+
y5+-(+H3+N)

O14957

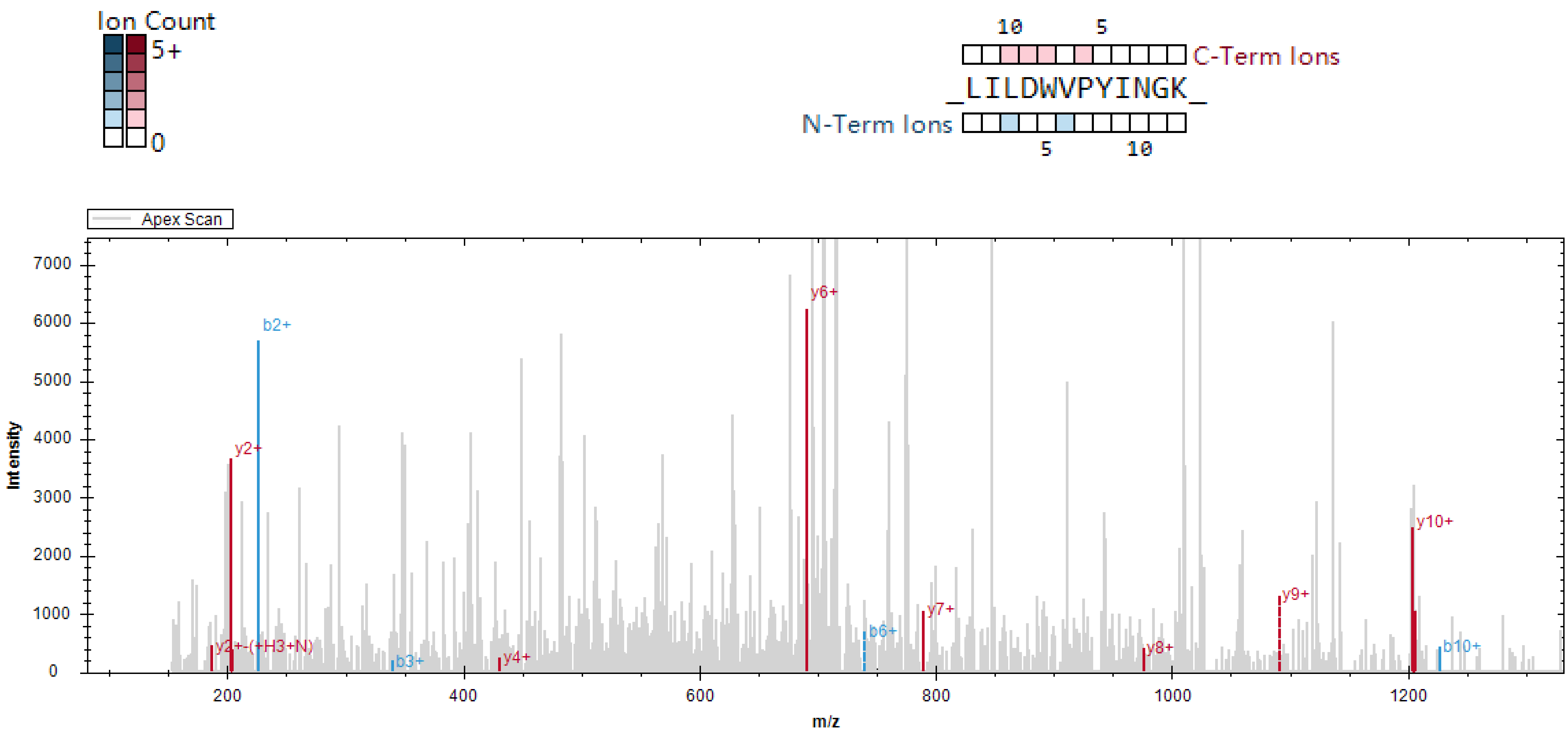

Ion Count
5+
0
10
5
C-Term Ions
_LILDWVPYINGK_
N-Term Ions
5
10
Apex Scan
Intensity
7000
6000
5000
4000
3000
2000
1000
0
200
400
600
800
1000
1200
m/z
b2+
y2+
y2+-(+H3+N)
b3+
y4+
y6+
b6+
y7+
y8+
y9+
y10+
b10+

O14972

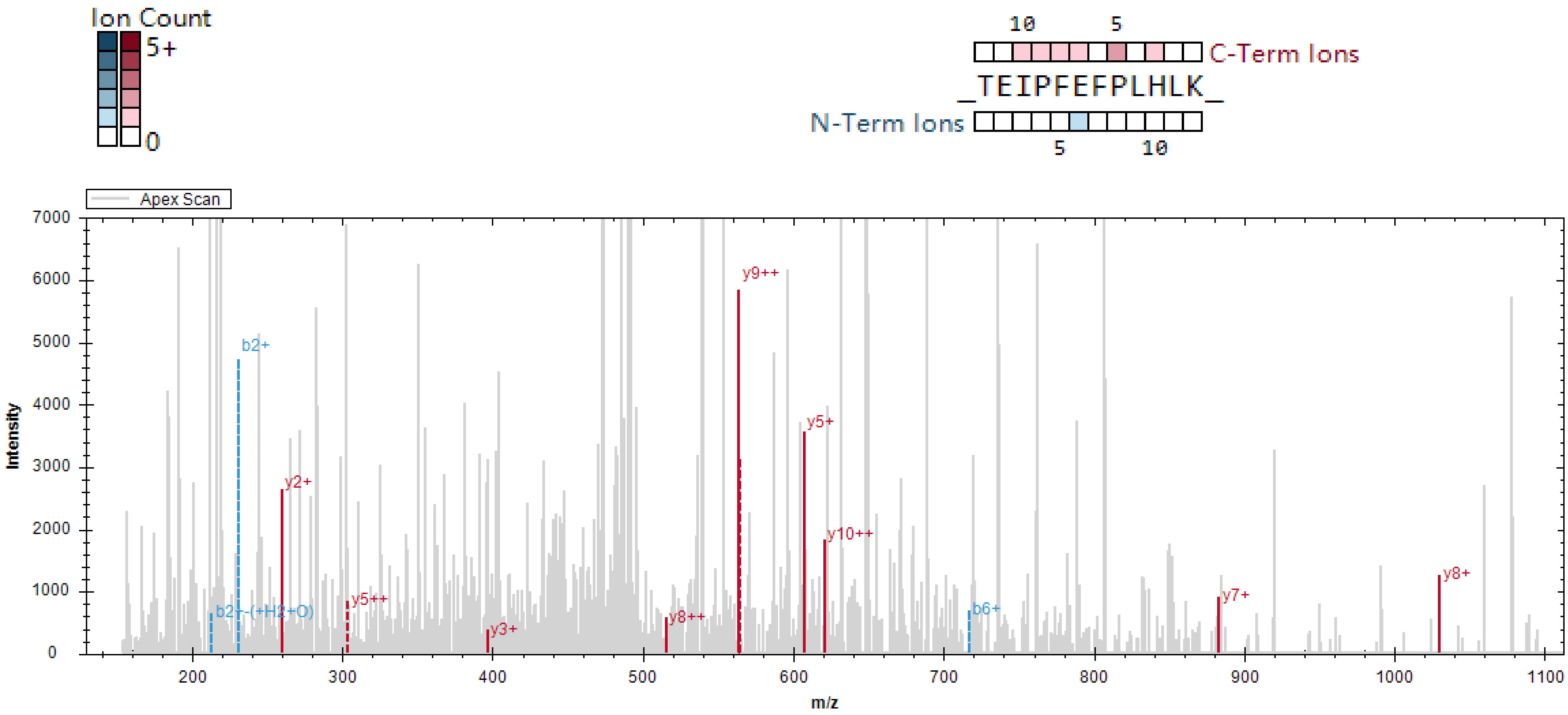

Ion Count
5+
0
10
5
C-Term Ions
_TEIPFEFPLHLK_
N-Term Ions
5
10
Apex Scan
Intensity
m/z
b2+
b2+-(+H2+O)
y2+
y5++
y3+
y8++
y9++
y5+
y10++
b6+
y7+
y8+

# O15389

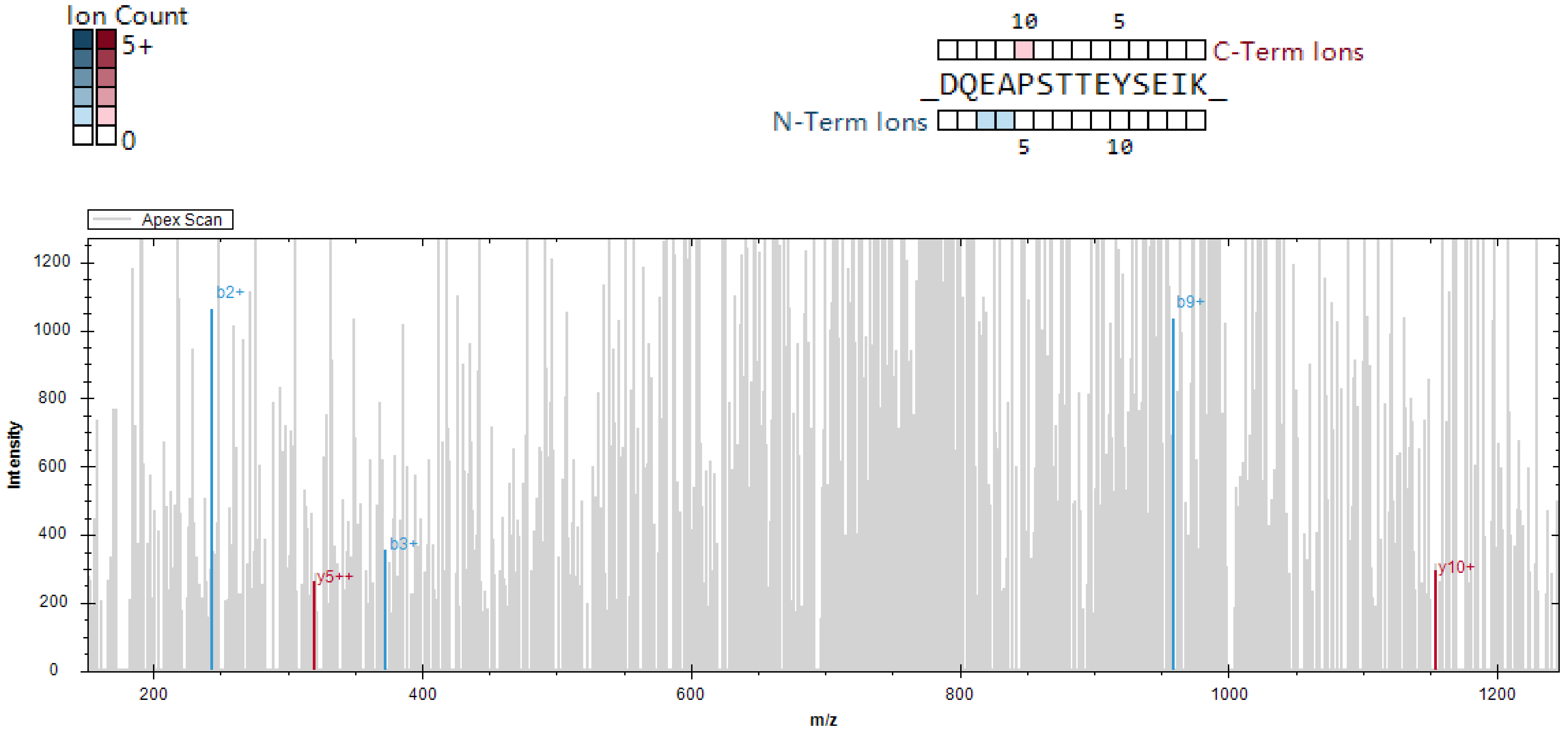
Ion Count
5+
0
10
5
C-Term Ions
_DQEAPSTTEYSEIK_
N-Term Ions
5
10
Apex Scan
Intensity
1200
1000
800
600
400
200
0
b2+
y5++
b3+
b9+
y10+
200
400
600
800
1000
1200
m/z

O15457

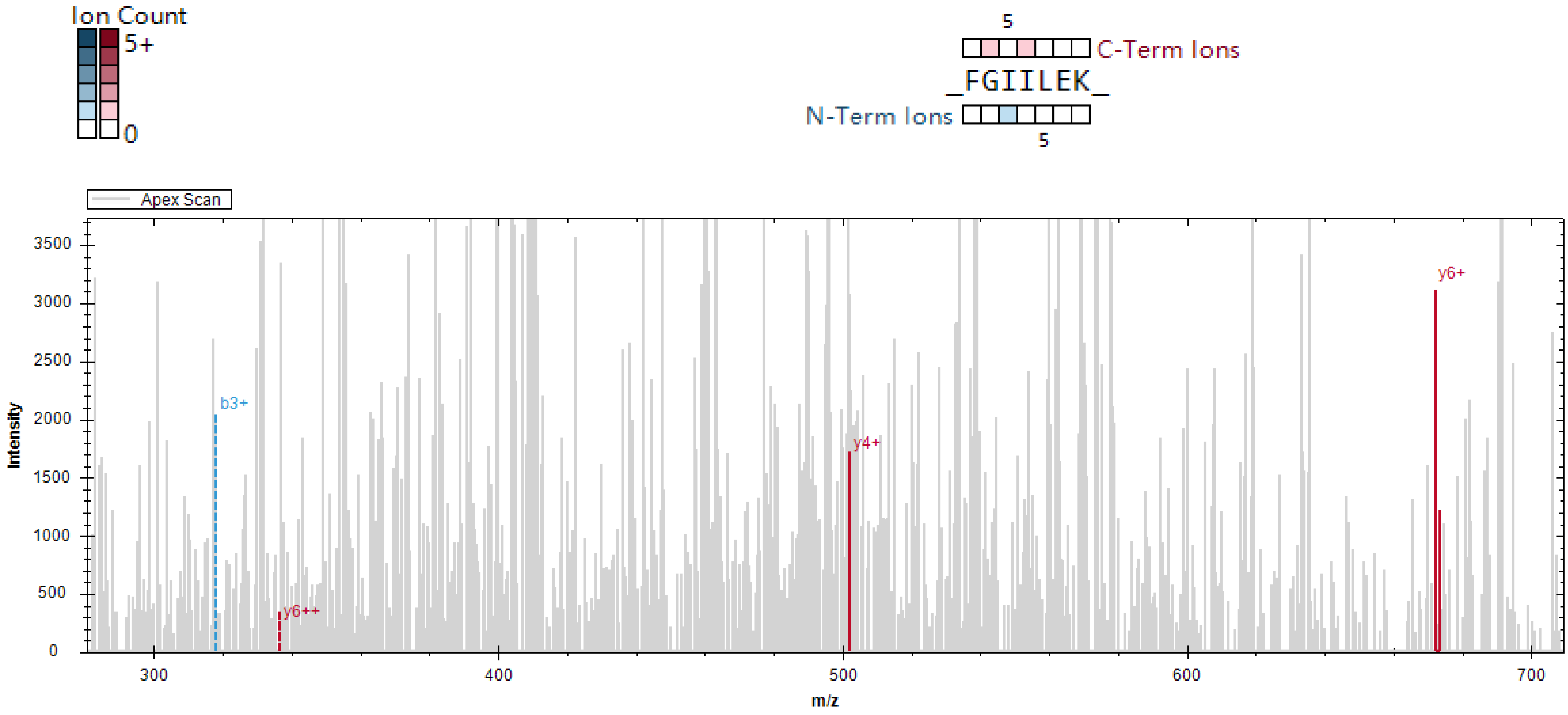

Ion Count
5+
0
5
C-Term Ions
_FGIILEK_
N-Term Ions
5
Apex Scan
Intensity
m/z
b3+
y6++
y4+
y6+
0
500
1000
1500
2000
2500
3000
3500
300
400
500
600
700

O43414

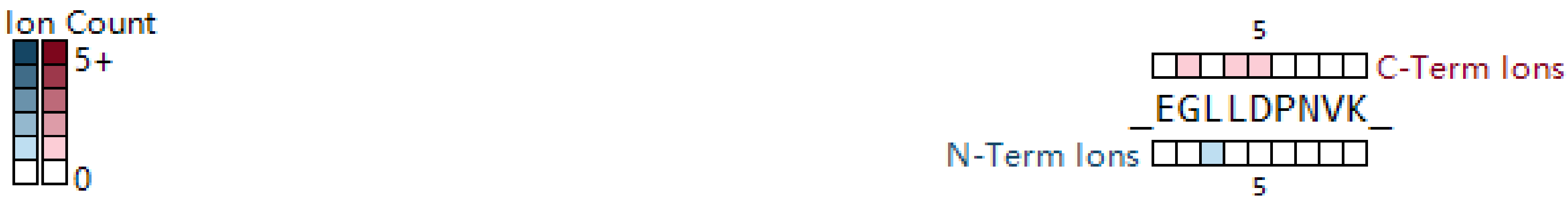

Ion Count
5+
0
5
C-Term Ions
_EGLLDPNVK_
N-Term Ions
5


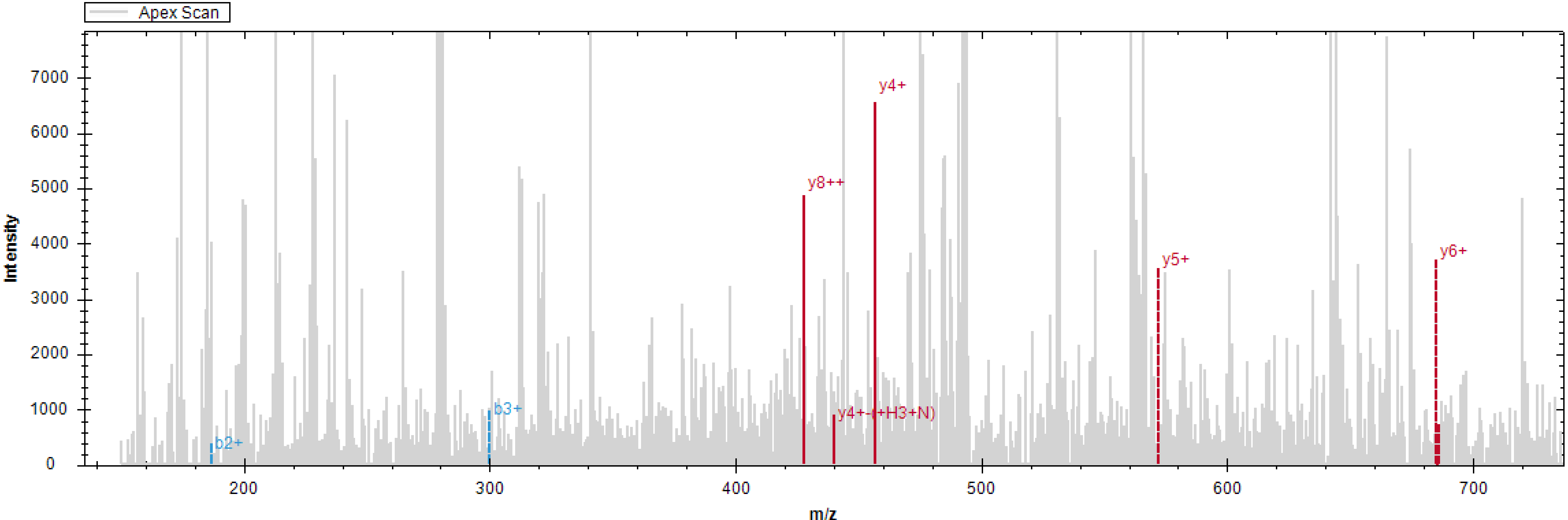

Apex Scan
Intensity
m/z
y4+
y8++
y5+
y6+
b2+
b3+
y4+-(+H3+N)

# O43736

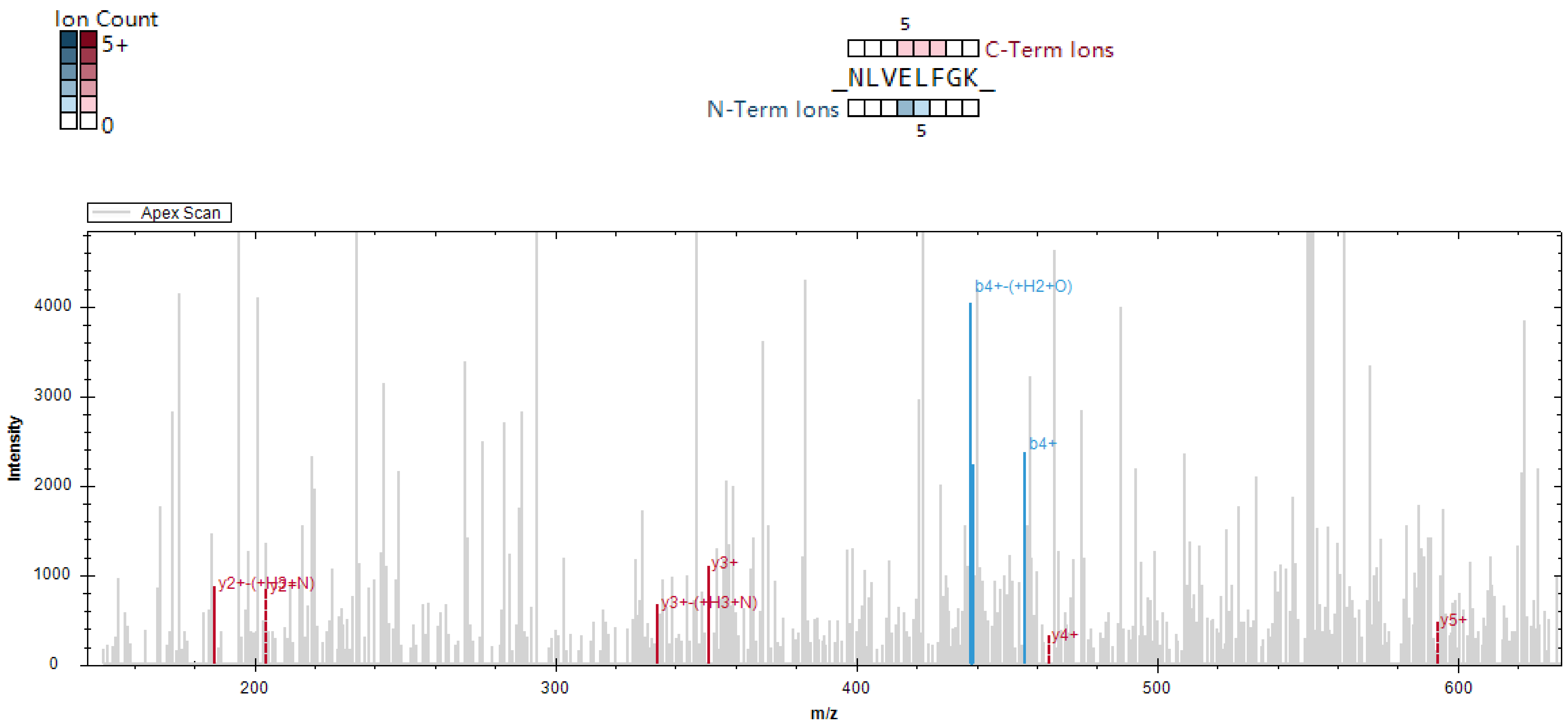

Ion Count
5+
0
5
C-Term Ions
_NLVELFGK_
N-Term Ions
5
Apex Scan
Intensity
m/z
0
1000
2000
3000
4000
200
300
400
500
600
b4+-(+H2+O)
b4+
y2+-(+H3+N)
y2+
y3+
y3+-(+H3+N)
y4+
y5+

O43825

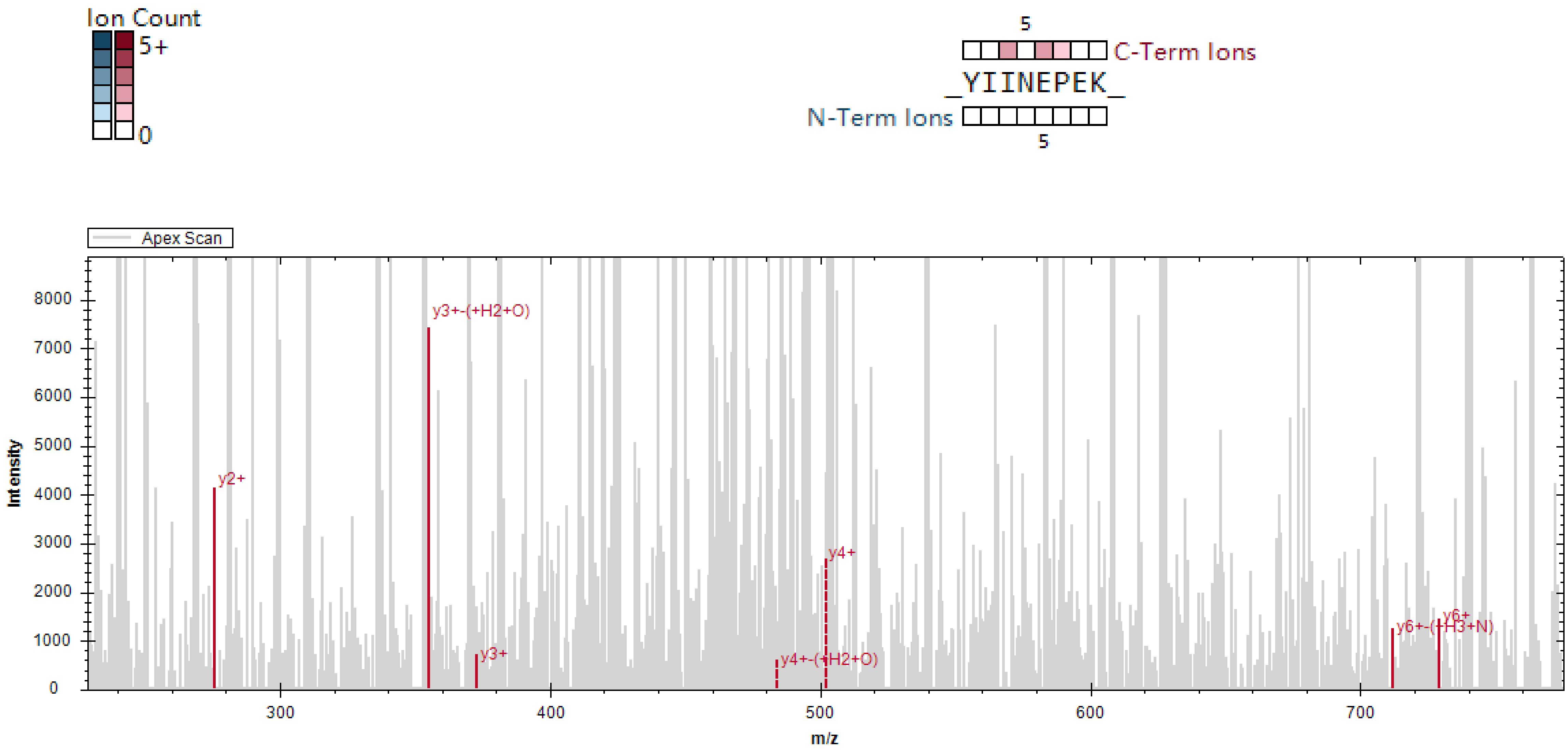

Ion Count
5+
0
5
C-Term Ions
_YIINEPEK_
N-Term Ions
5
Apex Scan
Intensity
m/z
y2+
y3+-(+H2+O)
y3+
y4+-(+H2+O)
y4+
y6+-(+H3+N)
y6+

O43927

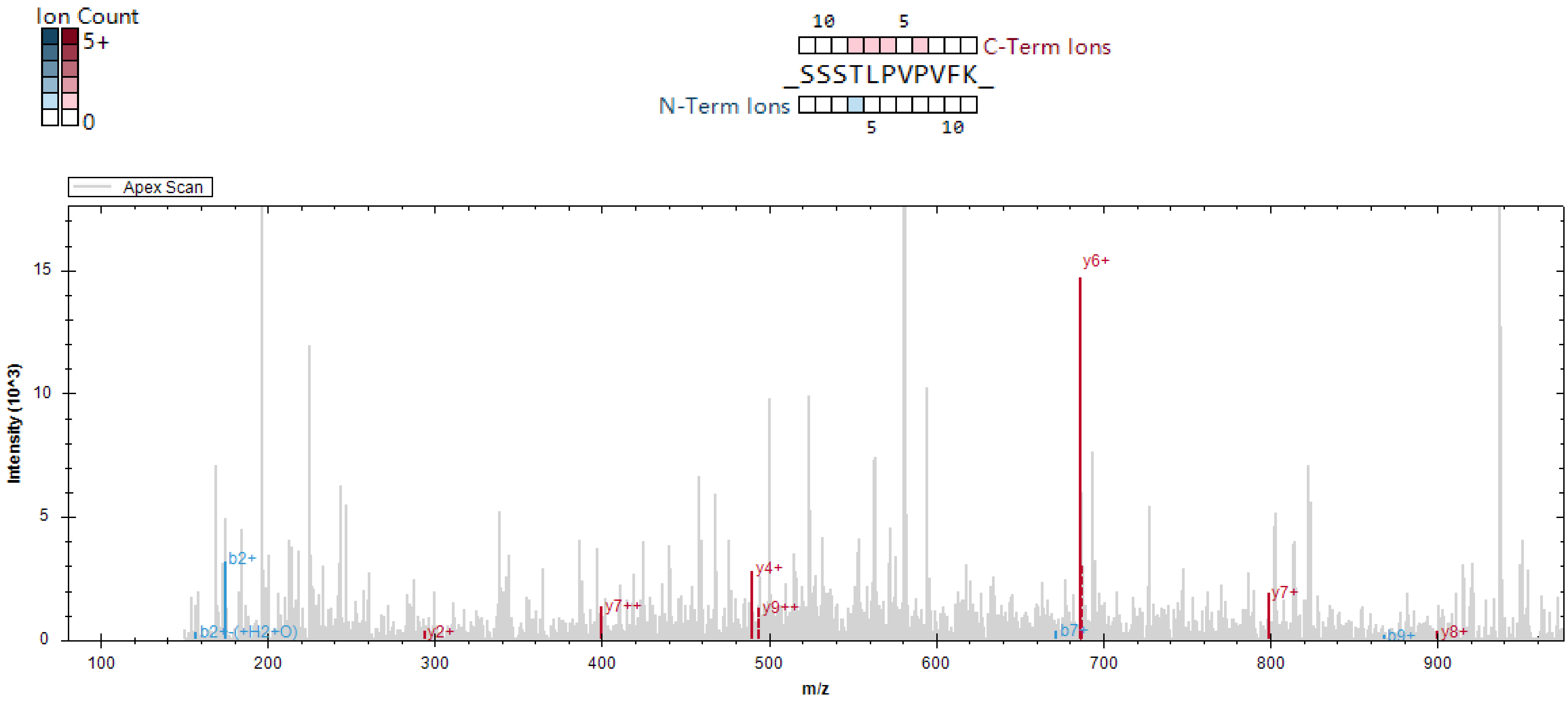
Ion Count
5+
0
10
5
C-Term Ions
_SSSTLPVPVFK_
N-Term Ions
5
10
Apex Scan
y6+
b2+
y4+
y7++
y9++
y7+
b2+-(+H2+O)
y2+
b7+
b9+
y8+
Intensity (10^3)
15
10
5
0
100
200
300
400
500
600
700
800
900
m/z

O43934

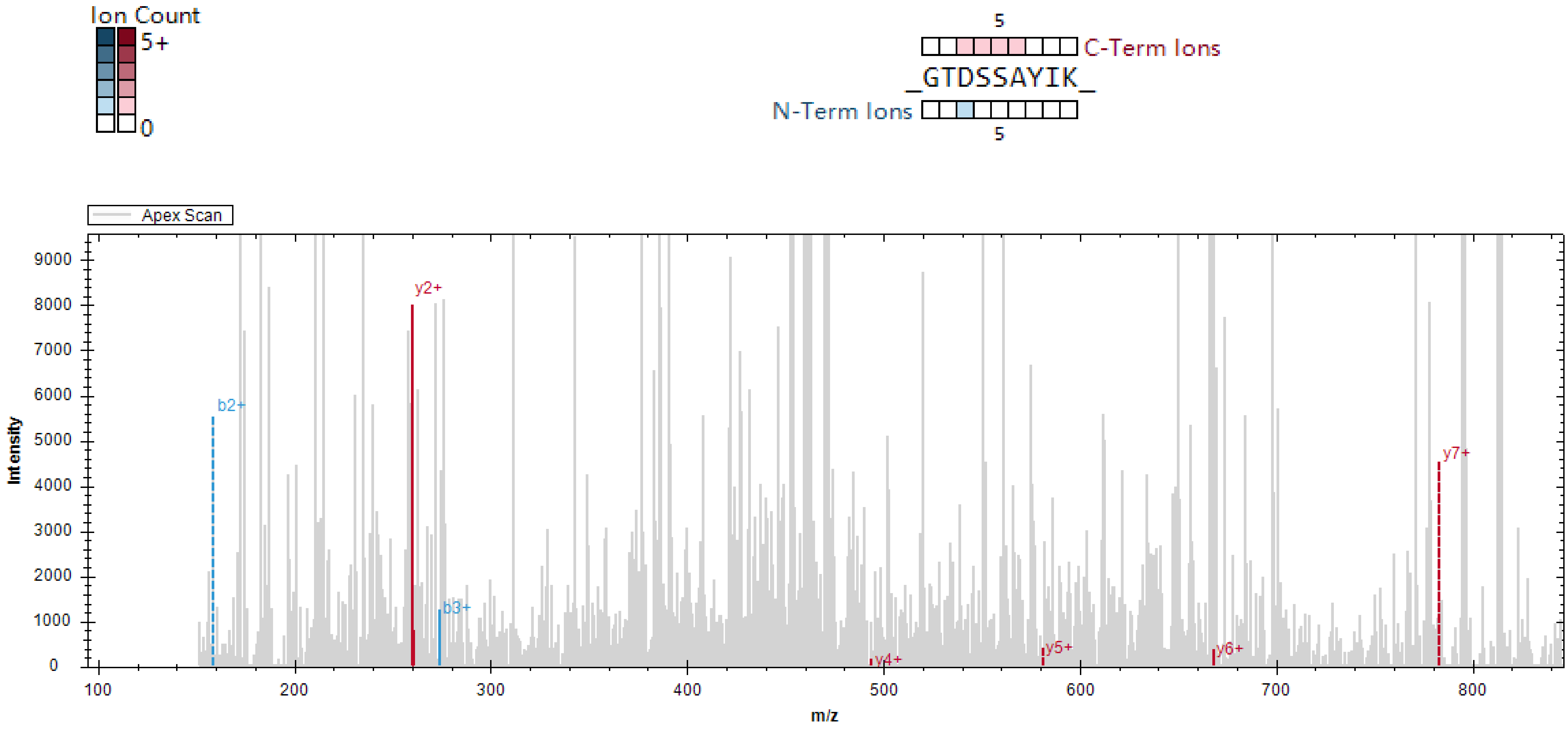

Ion Count
5+
0
5
C-Term Ions
_GTDSSAYIK_
N-Term Ions
5
Apex Scan
Intensity
m/z
b2+
b3+
y2+
y4+
y5+
y6+
y7+

# O60238

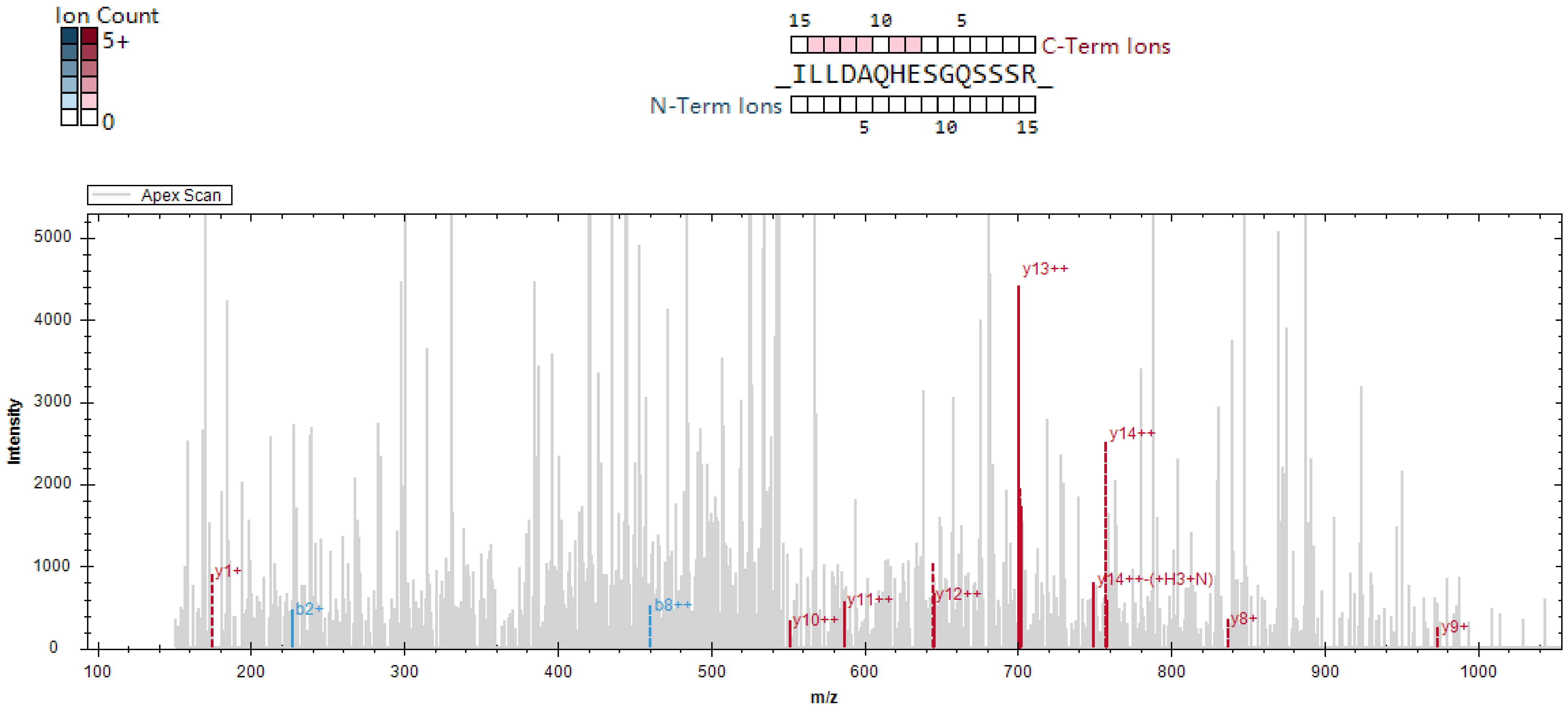

Ion Count
5+
0
15 10 5
C-Term Ions
_ILLDAQHESGQSSSR_
N-Term Ions
5 10 15
Apex Scan
Intensity
m/z
y13++
y14++
y1+
b2+
b8++
y10++
y11++
y12++
y14++-(+H3+N)
y8+
y9+

O60462

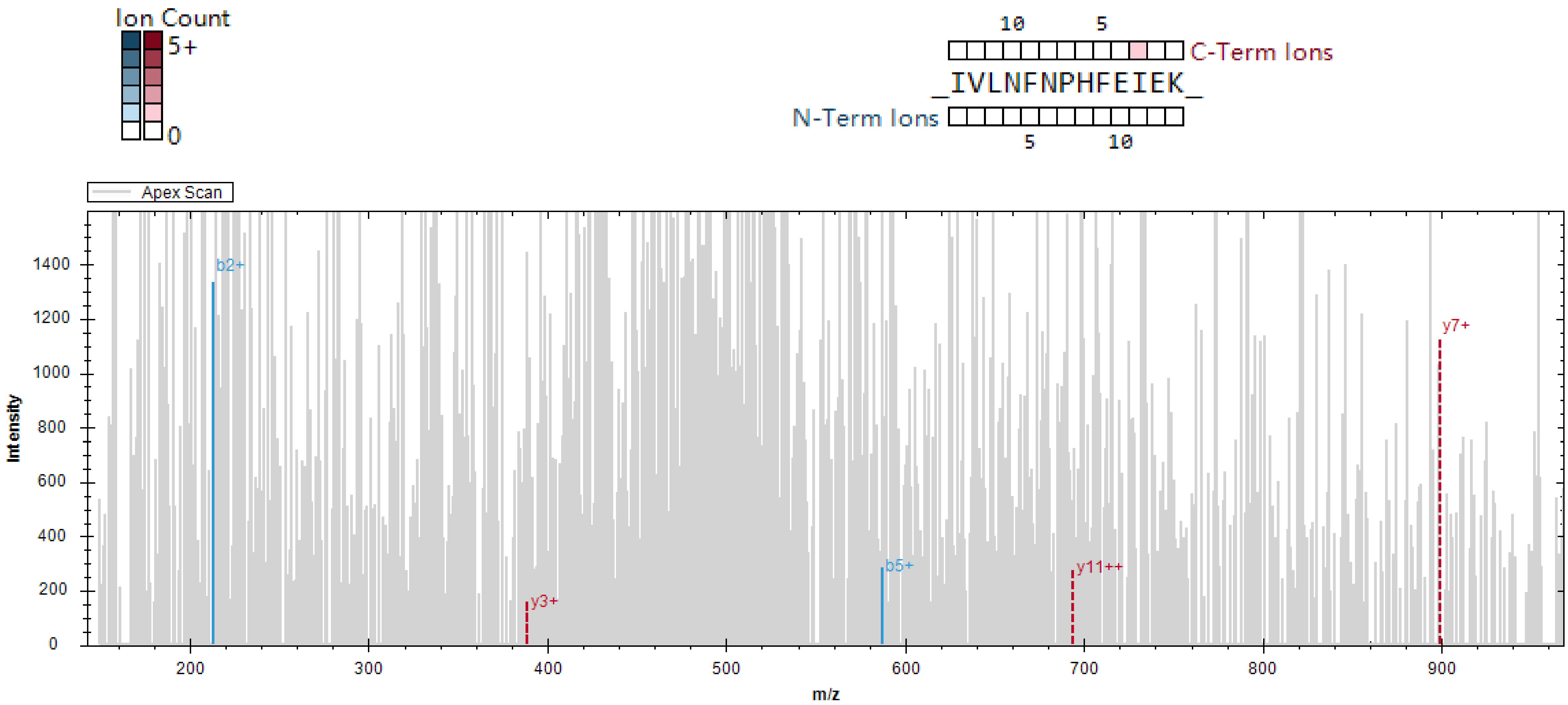

Ion Count
5+
0
10
5
C-Term Ions
_IVLNFNPHFEIEK_
N-Term Ions
5
10
Apex Scan
Intensity
m/z
b2+
b5+
y3+
y11++
y7+

O60499

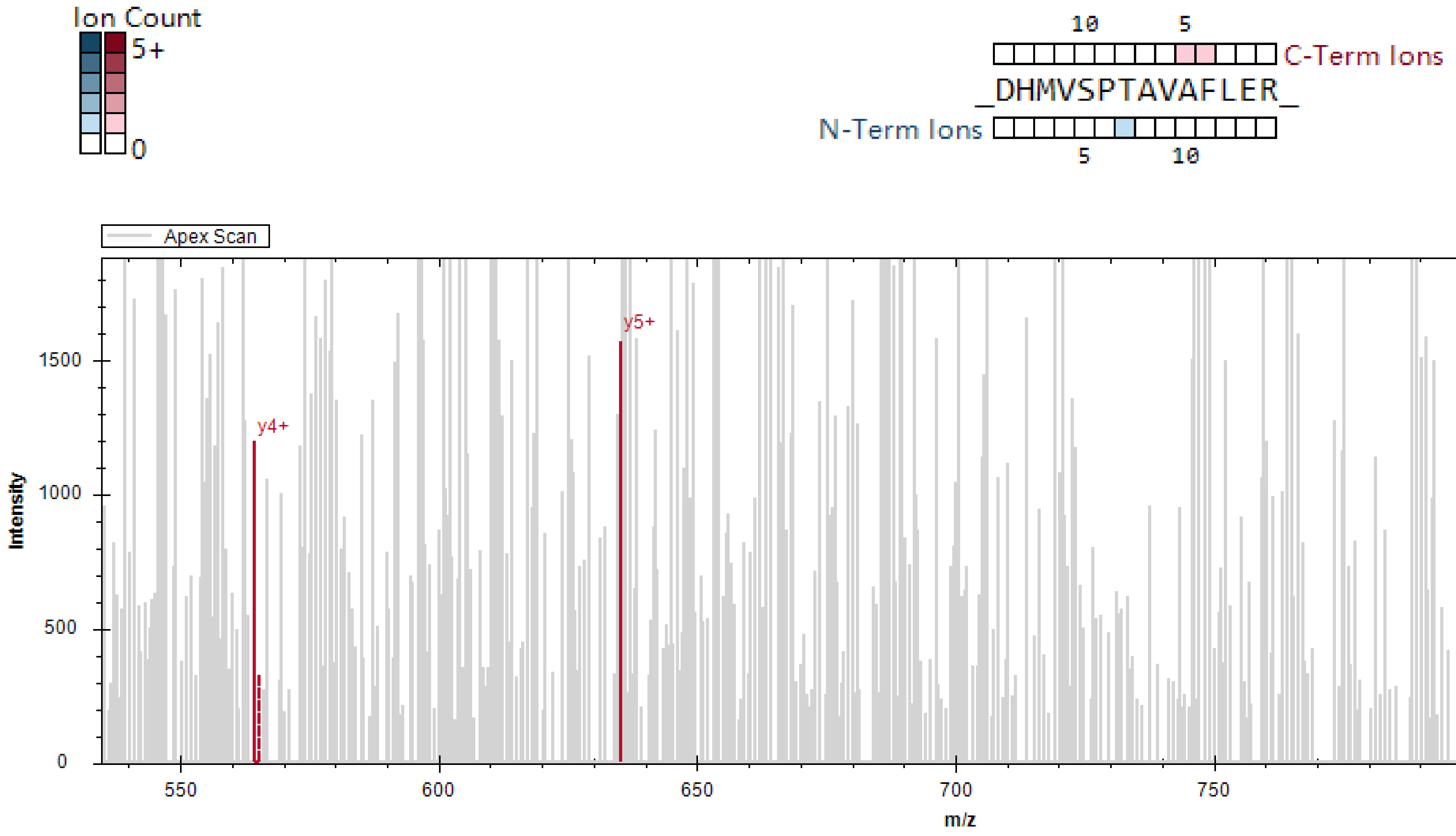

Ion Count
5+
0
10
5
C-Term Ions
_DHMVSPTAVAFLER_
N-Term Ions
5
10
Apex Scan
y4+
y5+
y7+
b8+
Intensity
0
500
1000
1500
550
600
650
700
750
800
850
m/z

O60637

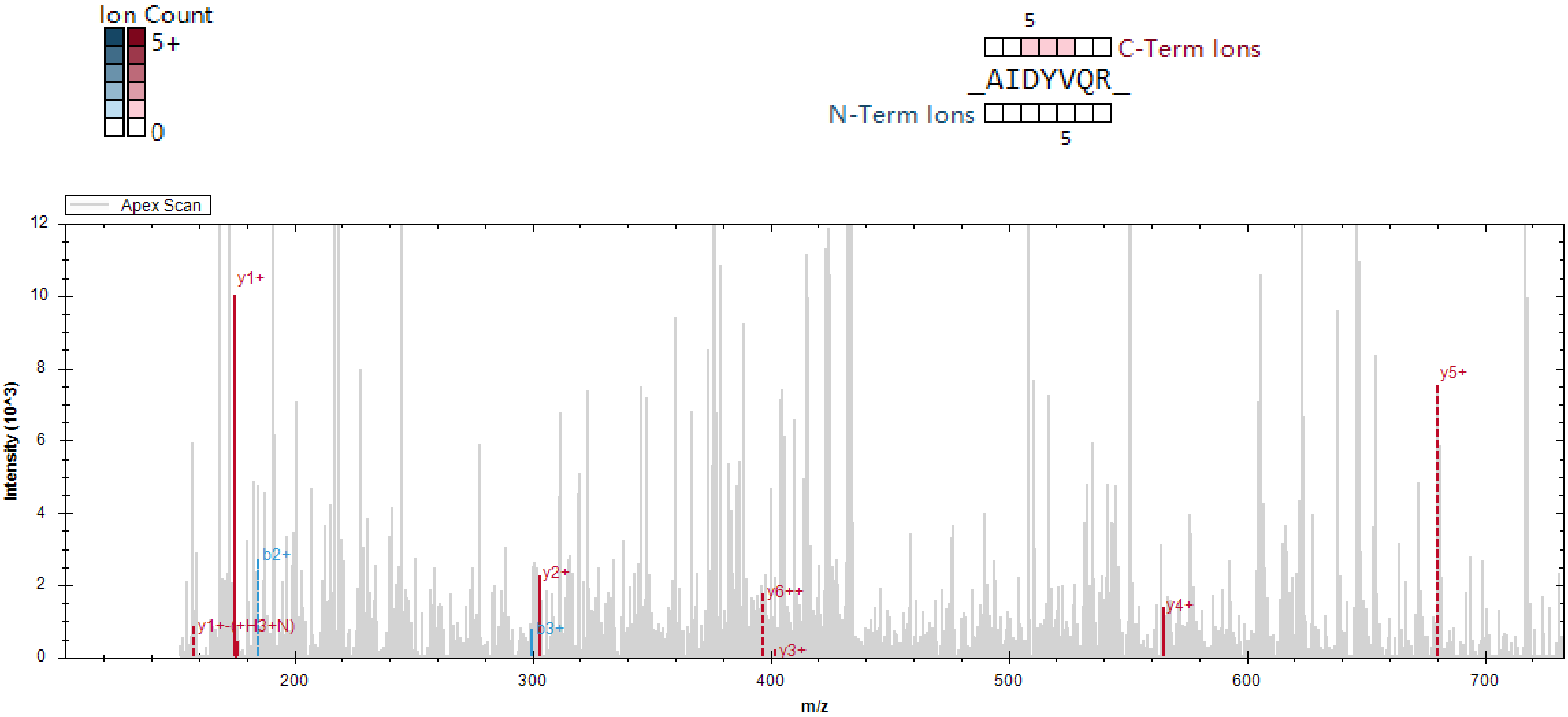

Ion Count
5+
0
5
C-Term Ions
_AIDYVQR_
N-Term Ions
5
Apex Scan
y1+
y1+-(+H3+N)
b2+
y2+
b3+
y6++
y3+
y4+
y5+
Intensity (10^3)
m/z

# O60831

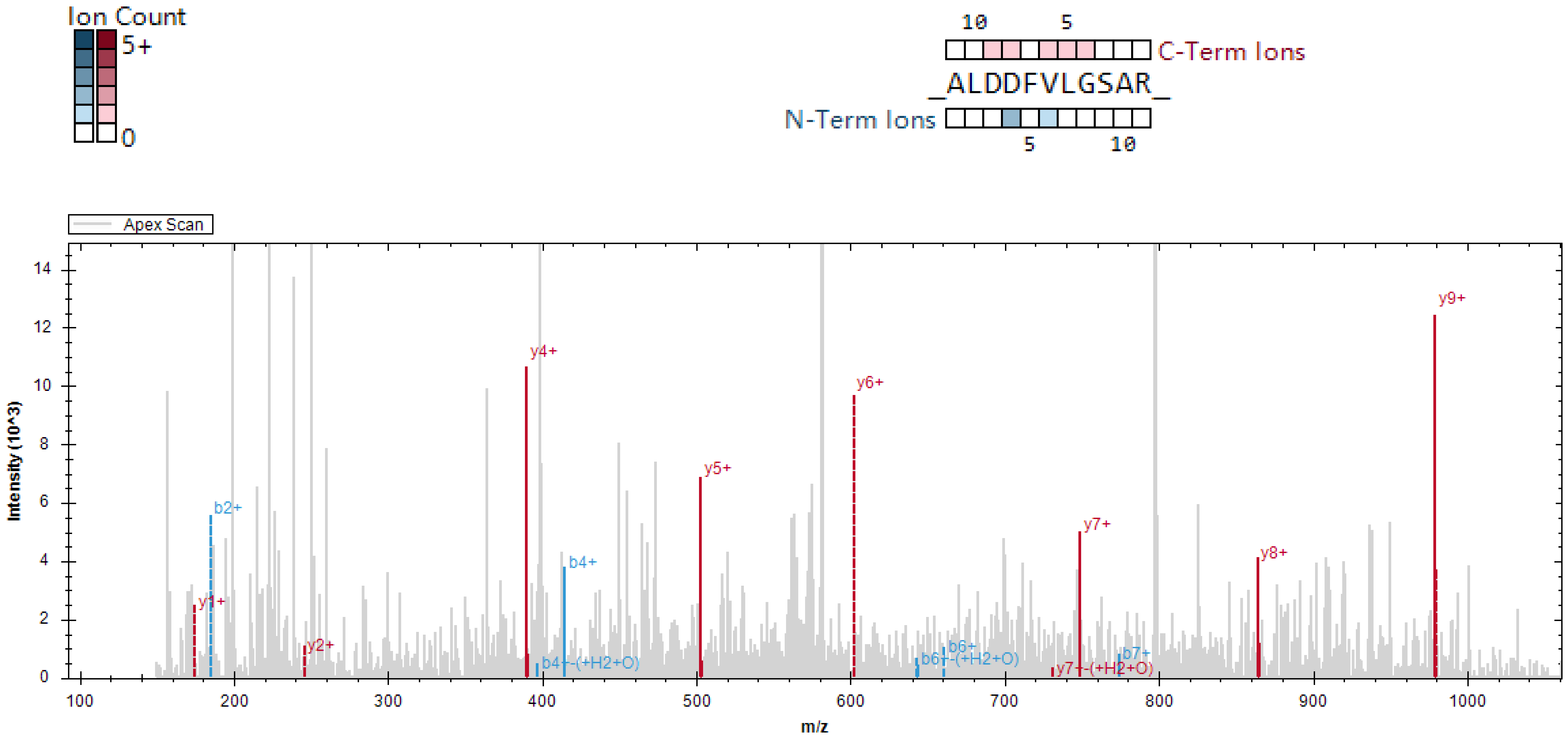

Ion Count
5+
0
10
5
C-Term Ions
_ALDDFVLGSAR_
N-Term Ions
5
10
Apex Scan
Intensity (10^3)
m/z
y1+
b2+
y2+
y4+
b4+
b4+-(+H2+O)
y5+
y6+
b6+
b6+-(+H2+O)
y7+
y7+-(+H2+O)
b7+
y8+
y9+

O75081

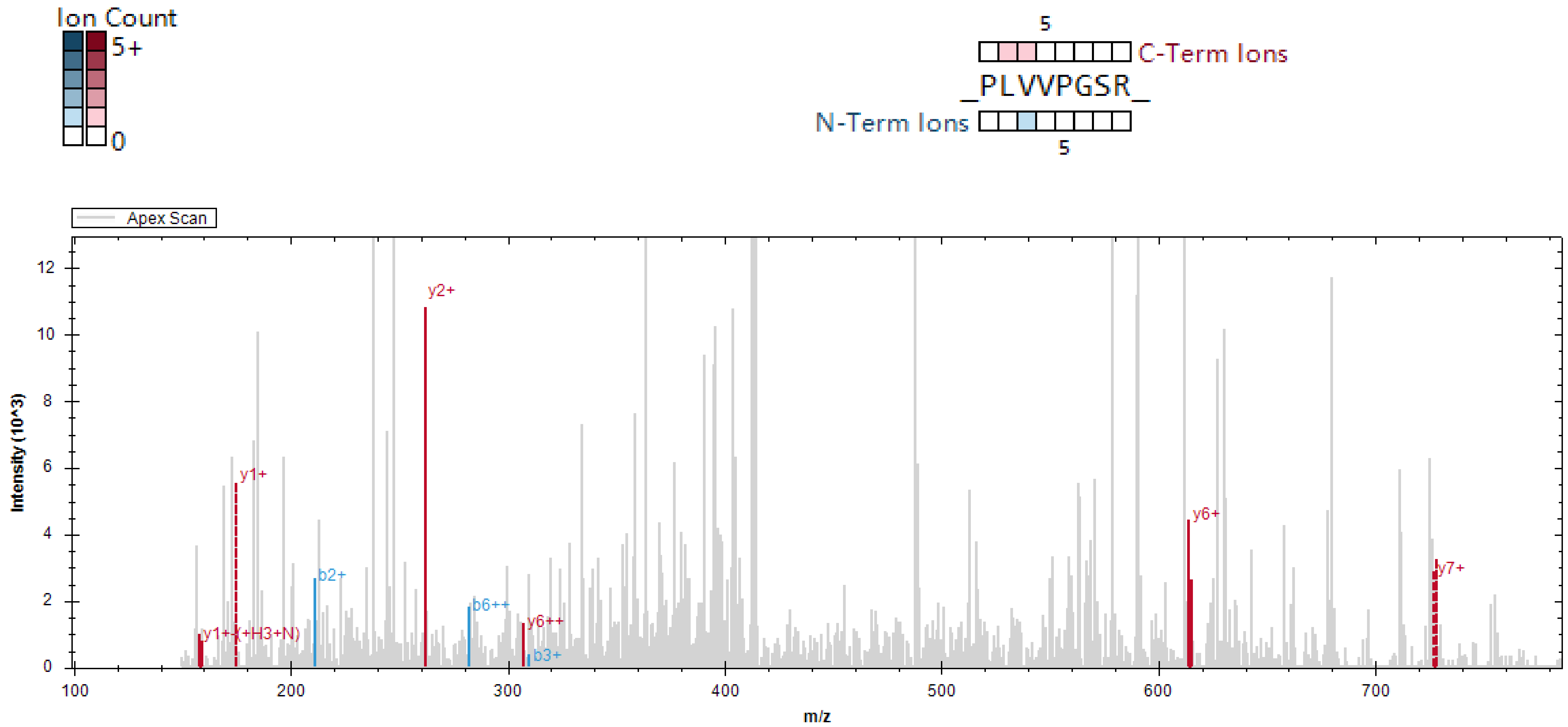
Ion Count
5+
0
5
C-Term Ions
_PLVVPGSR_
N-Term Ions
5
Apex Scan
Intensity (10^3)
m/z
y2+
y1+
b2+
b6++
y6++
b3+
y1+-(+H3+N)
y6+
y7+

O75379

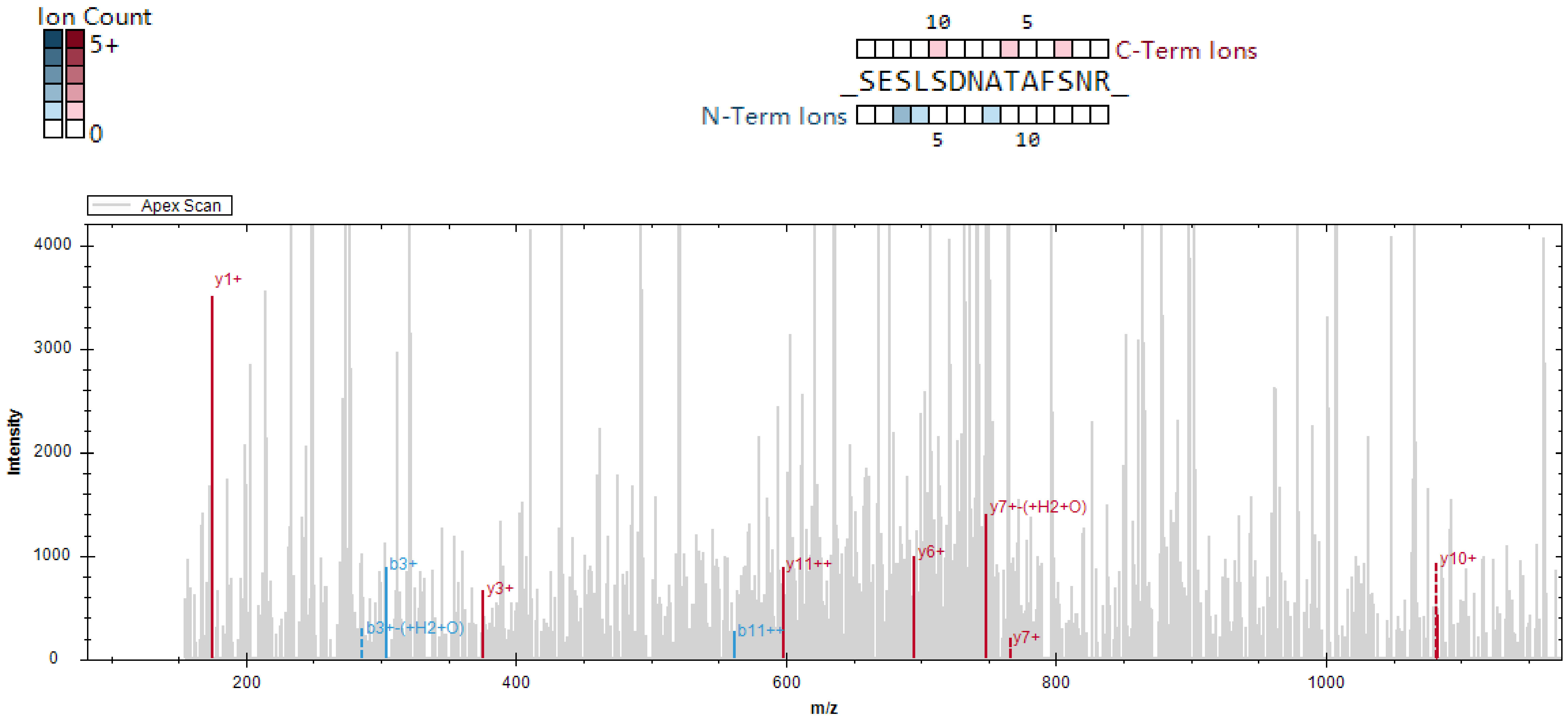

Ion Count
5+
0
10
5
C-Term Ions
_SESLSDNATAFSNR_
N-Term Ions
5
10
Apex Scan
Intensity
m/z
y1+
b3+
b3+-(+H2+O)
y3+
b11++
y11++
y6+
y7+-(+H2+O)
y7+
y10+

O75970

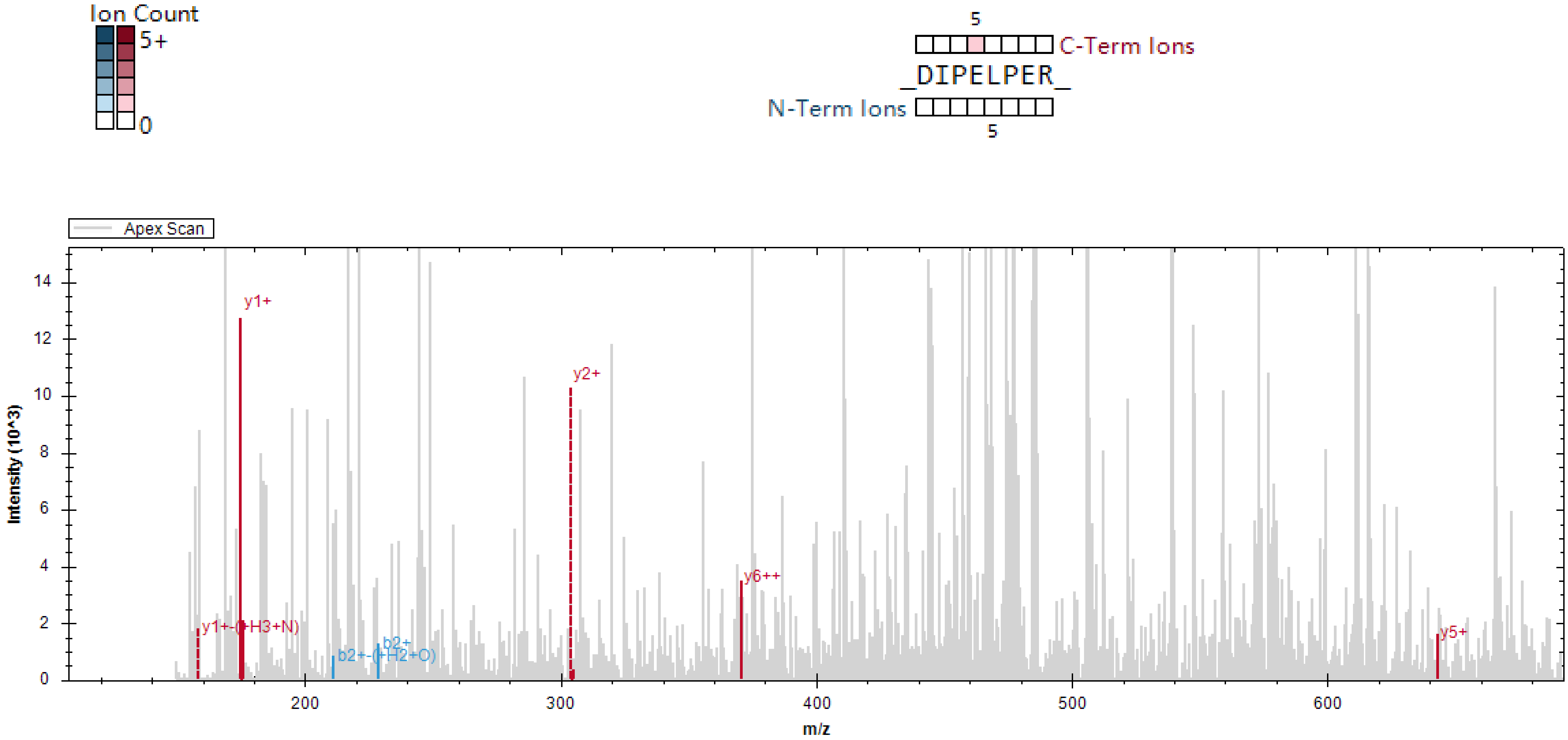

Ion Count
5+
0
5
C-Term Ions
_DIPELPER_
N-Term Ions
5
Apex Scan
Intensity (10^3)
m/z
y1+
y2+
y6++
y5+
y1+-(+H3+N)
b2+
b2+-(+H2+O)

O94819

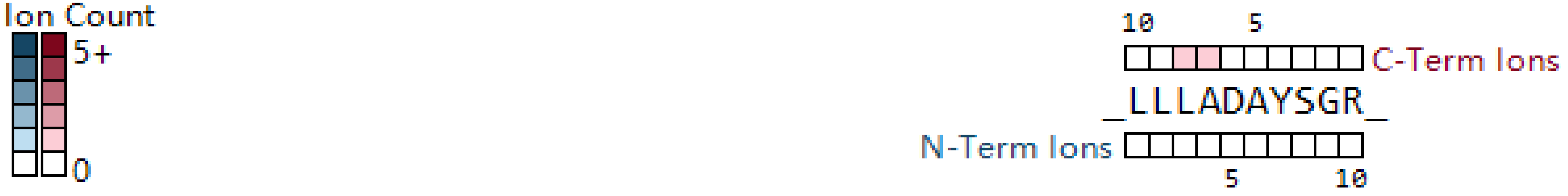
Ion Count
5+
0
10
5
C-Term Ions
_LLLADAYSGR_
N-Term Ions
5
10

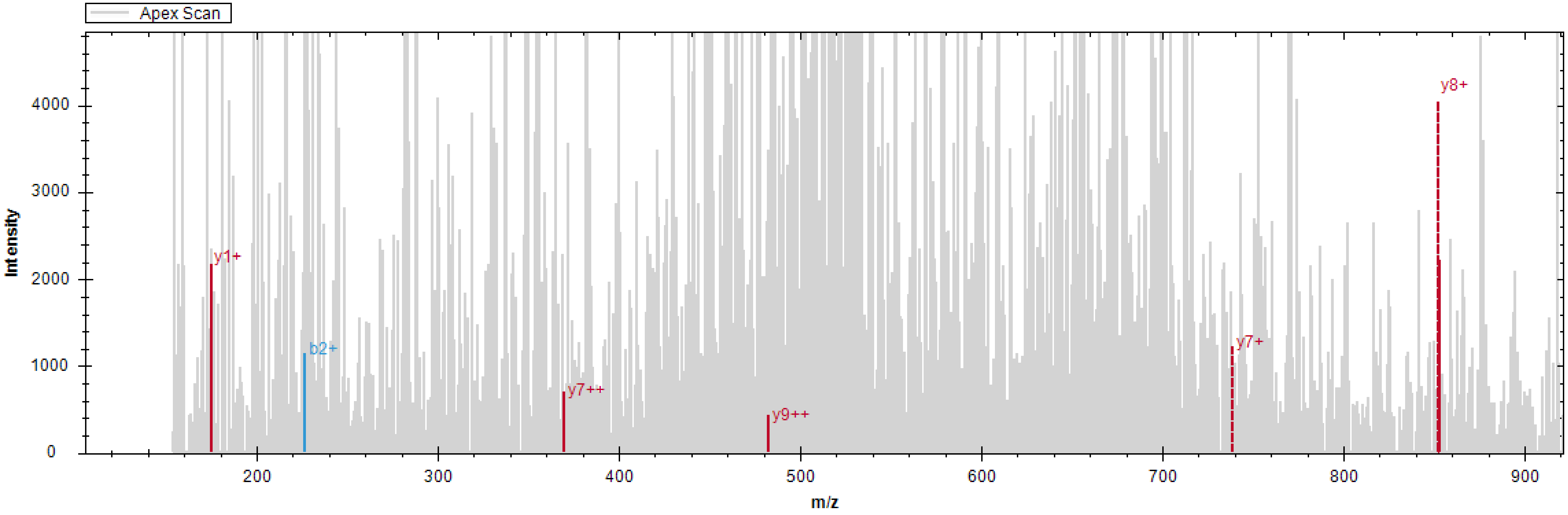
Apex Scan
Intensity
m/z
y1+
b2+
y7++
y9++
y7+
y8+

O95155

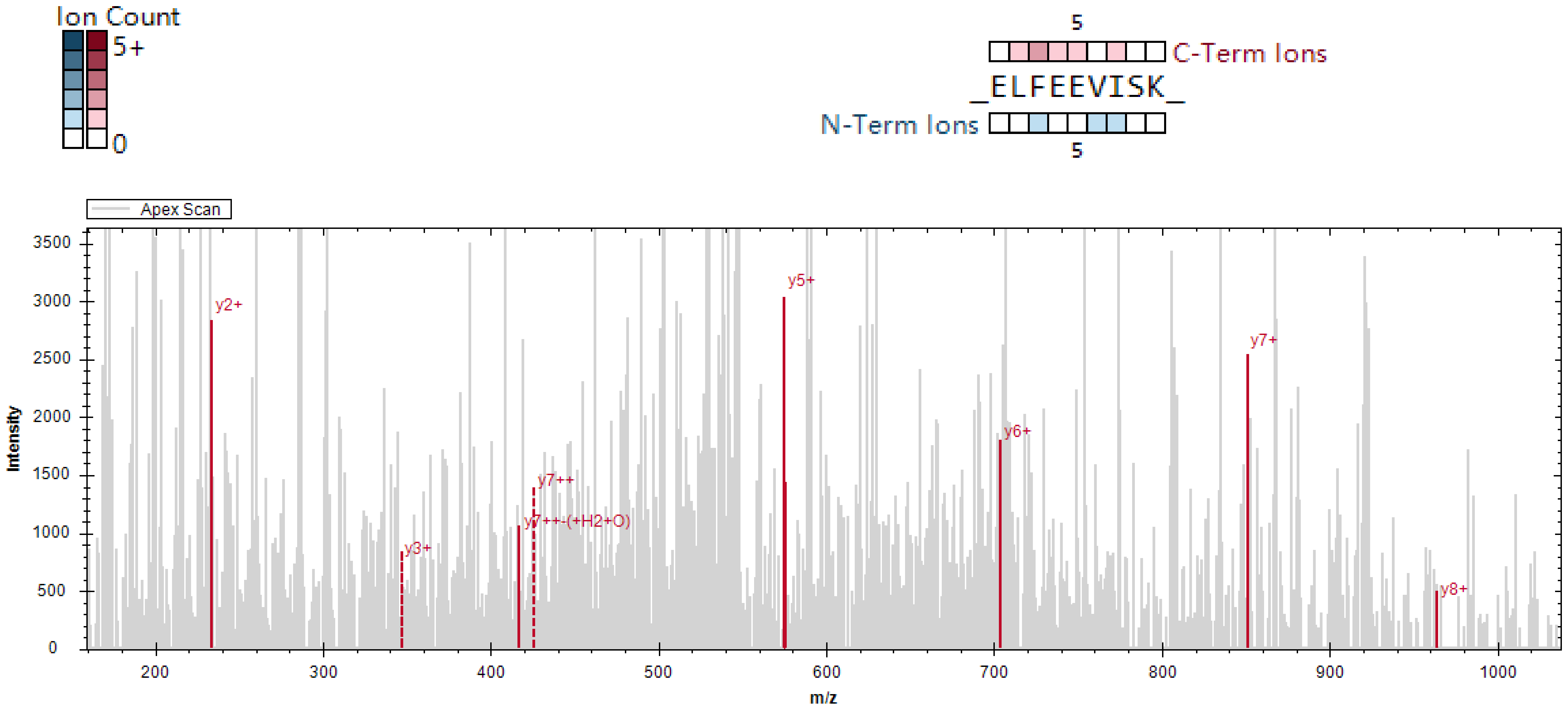
Ion Count
5+
0
5
C-Term Ions
_ELFEEVISK_
N-Term Ions
5
Apex Scan
y2+
y3+
y7++-(+H2+O)
y7++
y5+
y6+
y7+
y8+
Intensity
m/z

O95456

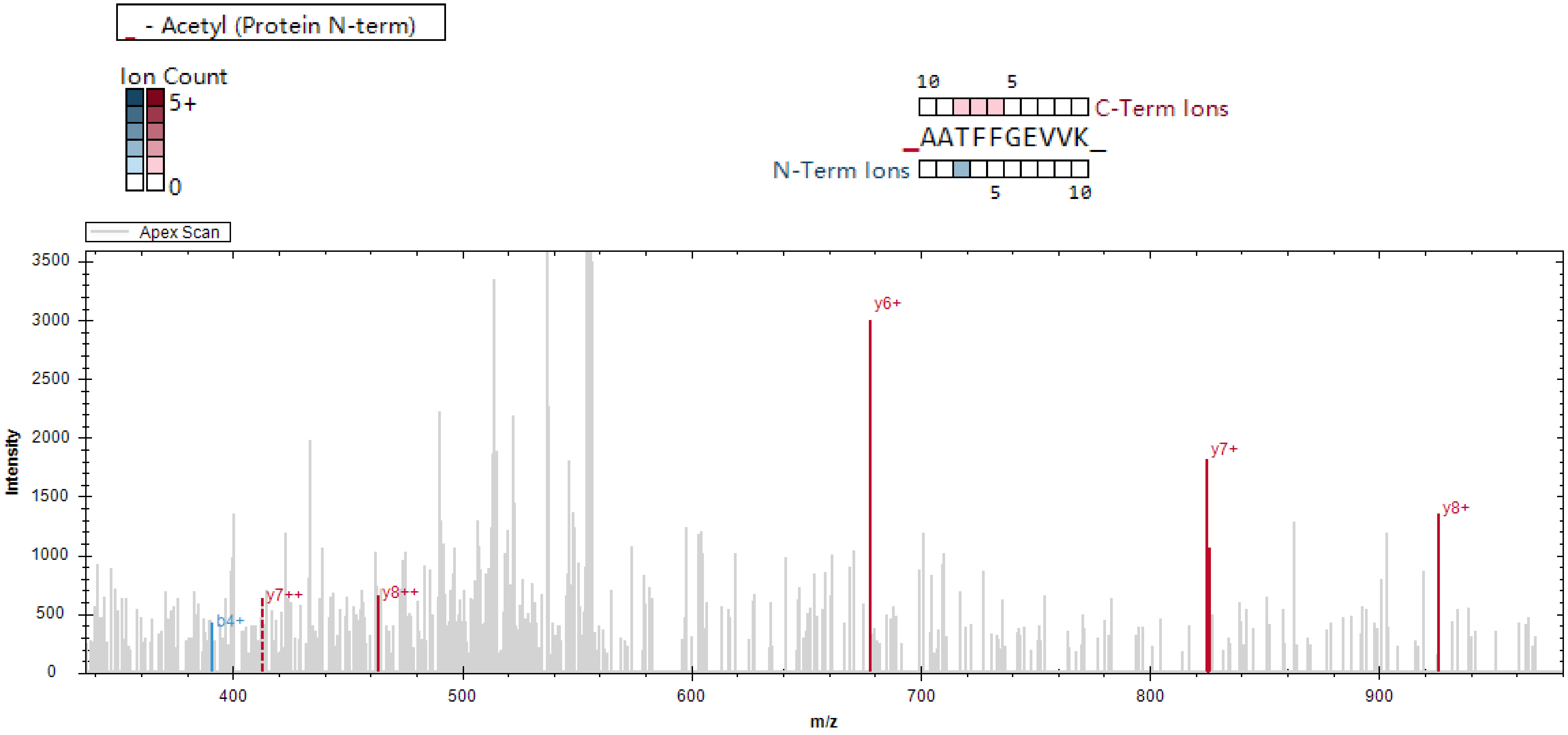
_ - Acetyl (Protein N-term)
Ion Count
5+
0
10
5
C-Term Ions
_AATFFGEVVK_
N-Term Ions
5
10
Apex Scan
3500
3000
2500
2000
1500
1000
500
0
Intensity
y6+
y7+
y8+
y7++
y8++
b4+
400
500
600
700
800
900
m/z

O95490

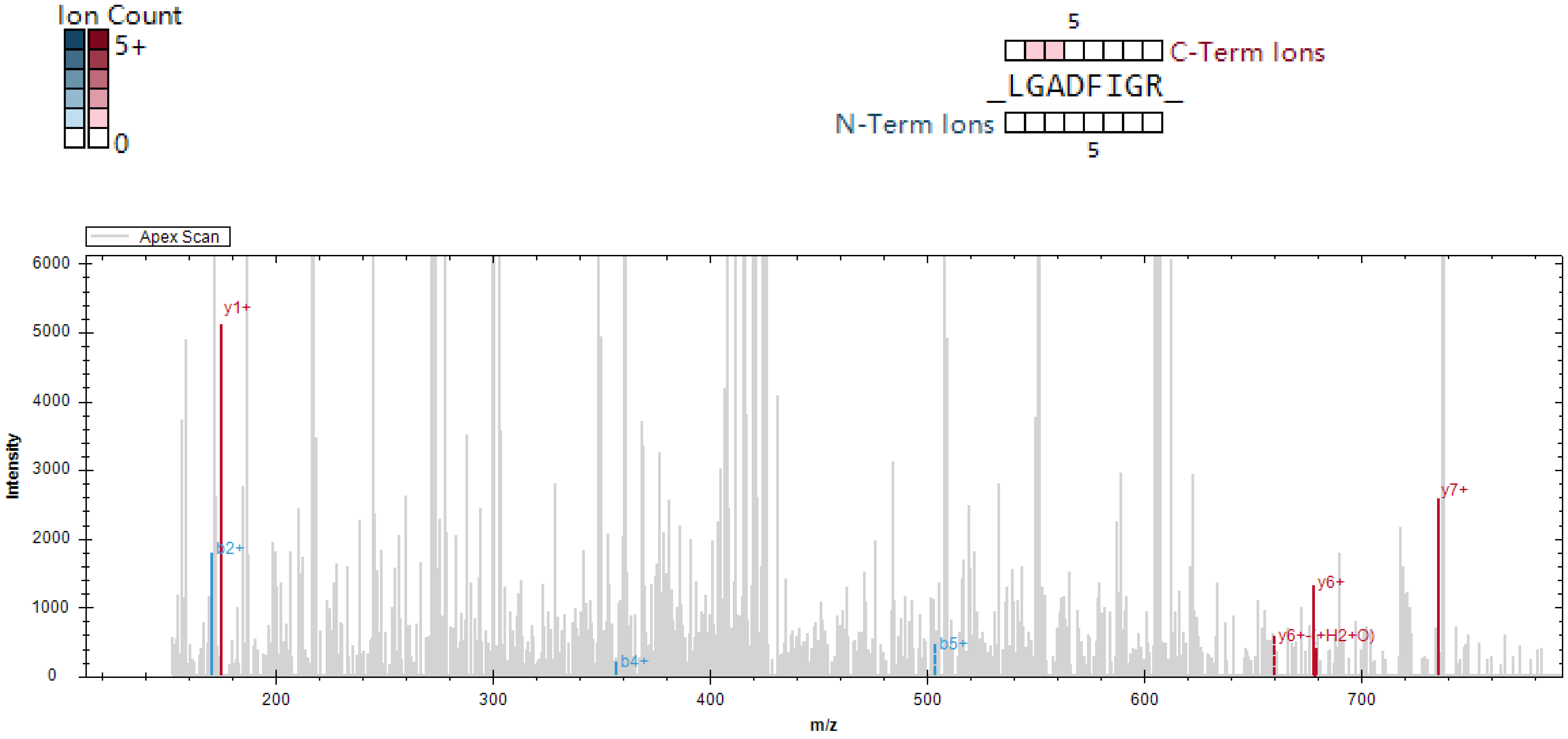

Ion Count
5+
0
5
C-Term Ions
_LGADFIGR_
N-Term Ions
5
Apex Scan
Intensity
m/z
y1+
b2+
b4+
b5+
y6+
y6+-(+H2+O)
y7+

O95989

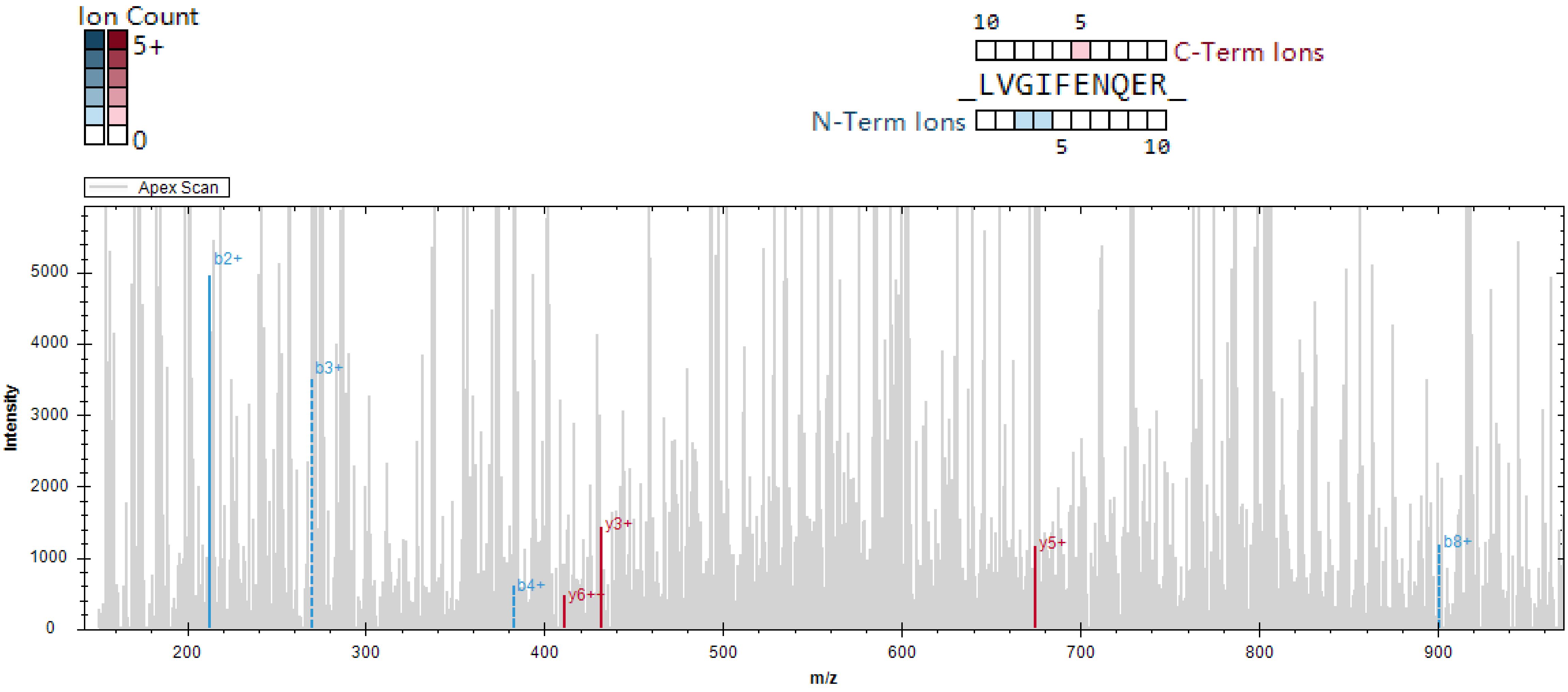

Ion Count
5+
0
10
5
C-Term Ions
_LVGIFENQER_
N-Term Ions
5
10
Apex Scan
b2+
b3+
b4+
y6++
y3+
y5+
b8+
Intensity
5000
4000
3000
2000
1000
0
200
300
400
500
600
700
800
900
m/z

O96019

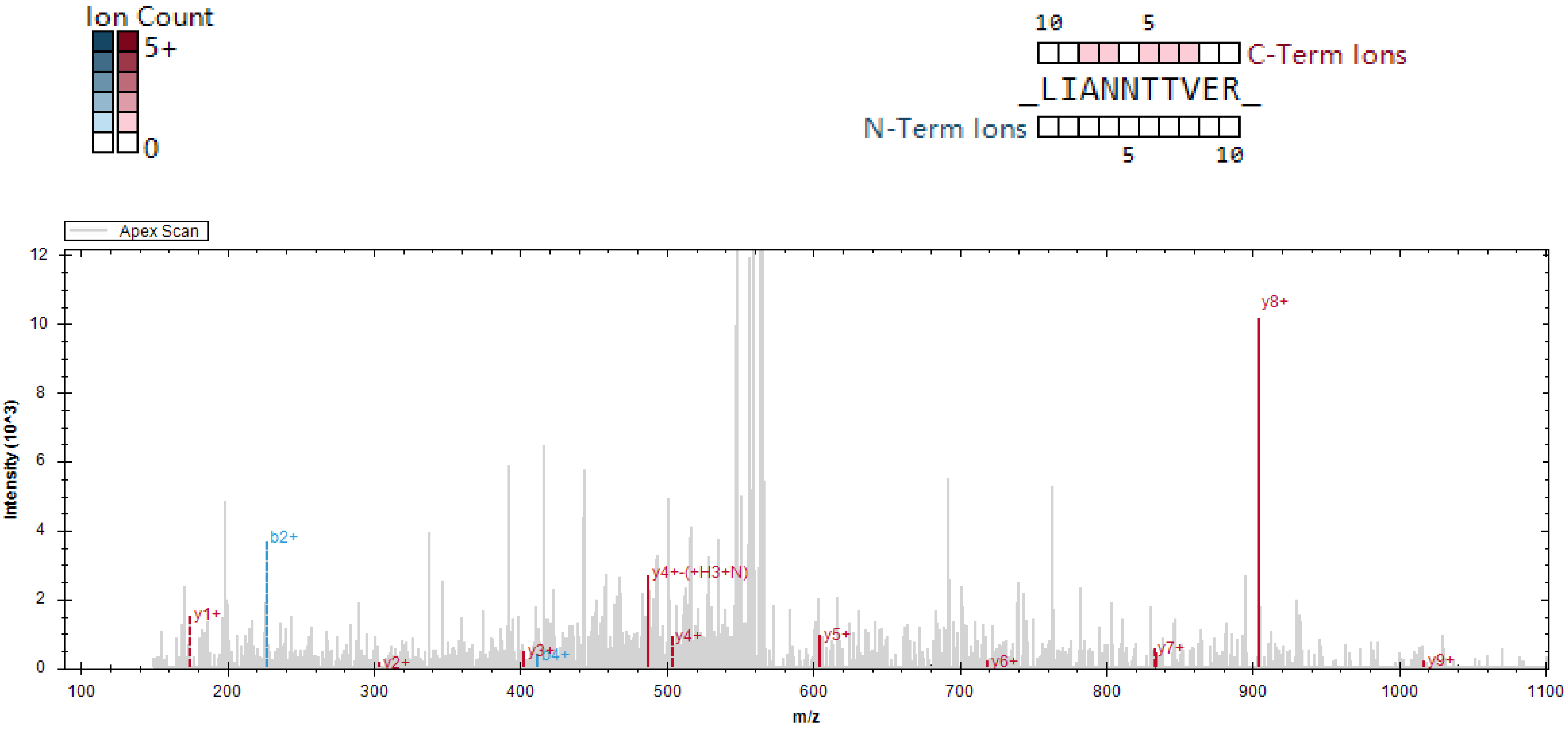
Ion Count
5+
0
10
5
C-Term Ions
_LIANNTTVER_
N-Term Ions
5
10
Apex Scan
y8+
b2+
y4+-(+H3+N)
y1+
y4+
y5+
y3+
b4+
y2+
y6+
y7+
y9+
Intensity (10^3)
m/z
0
2
4
6
8
10
12
100
200
300
400
500
600
700
800
900
1000
1100

P00709

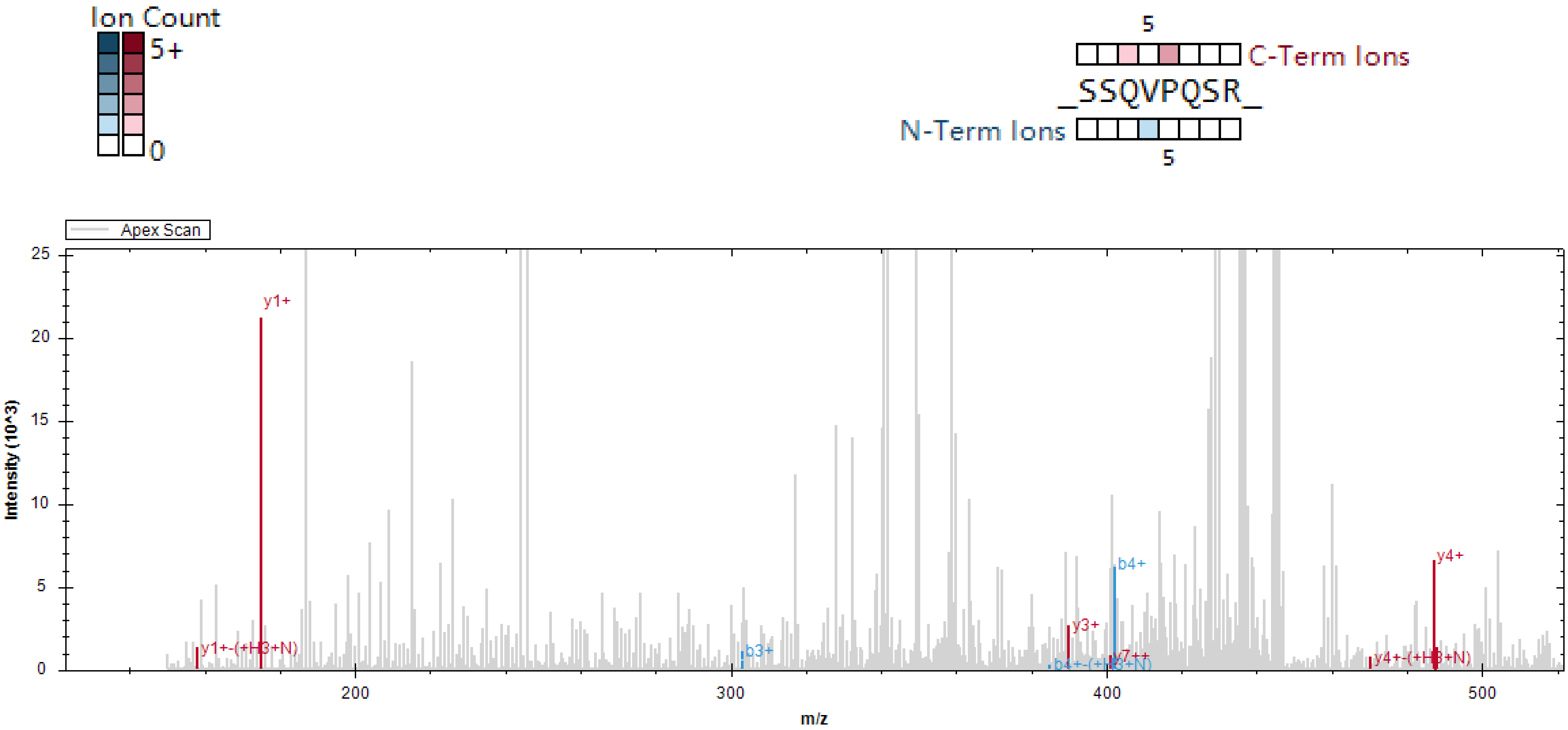

Ion Count
5+
0
5
C-Term Ions
_SSQVPQSR_
N-Term Ions
5
Apex Scan
Intensity (10^3)
m/z
y1+
y1+-(+H3+N)
b3+
b4+
b4+-(+H3+N)
y3+
y7++
y4+
y4+-(+H3+N)

# P01111

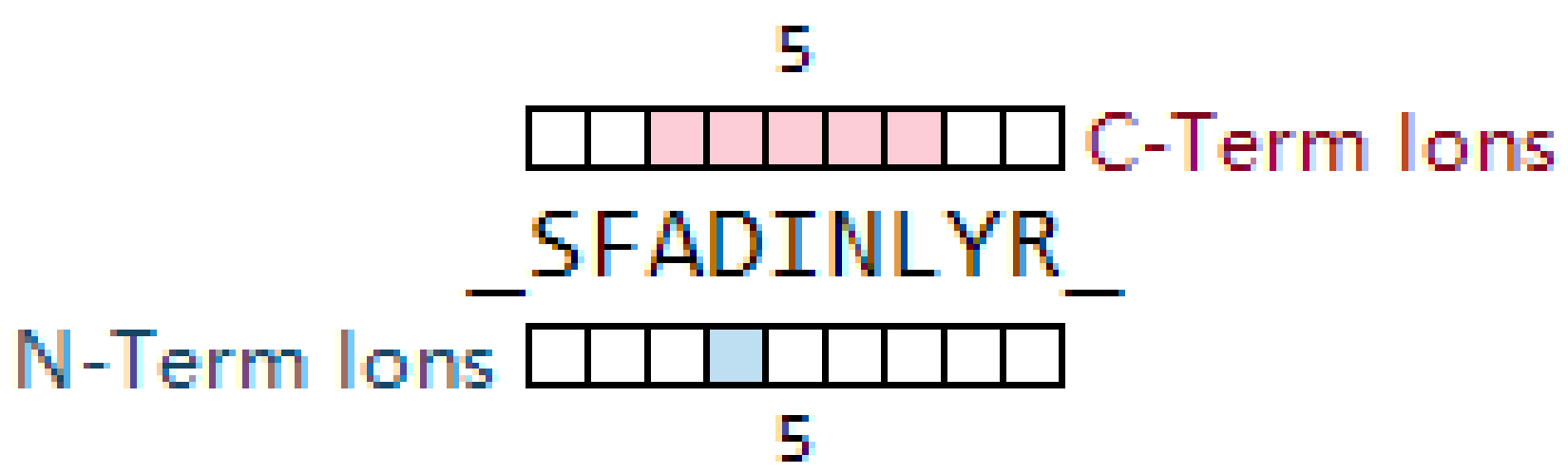
Ion Count
5+
0
5
C-Term Ions
_SFADINLYR_
N-Term Ions
5

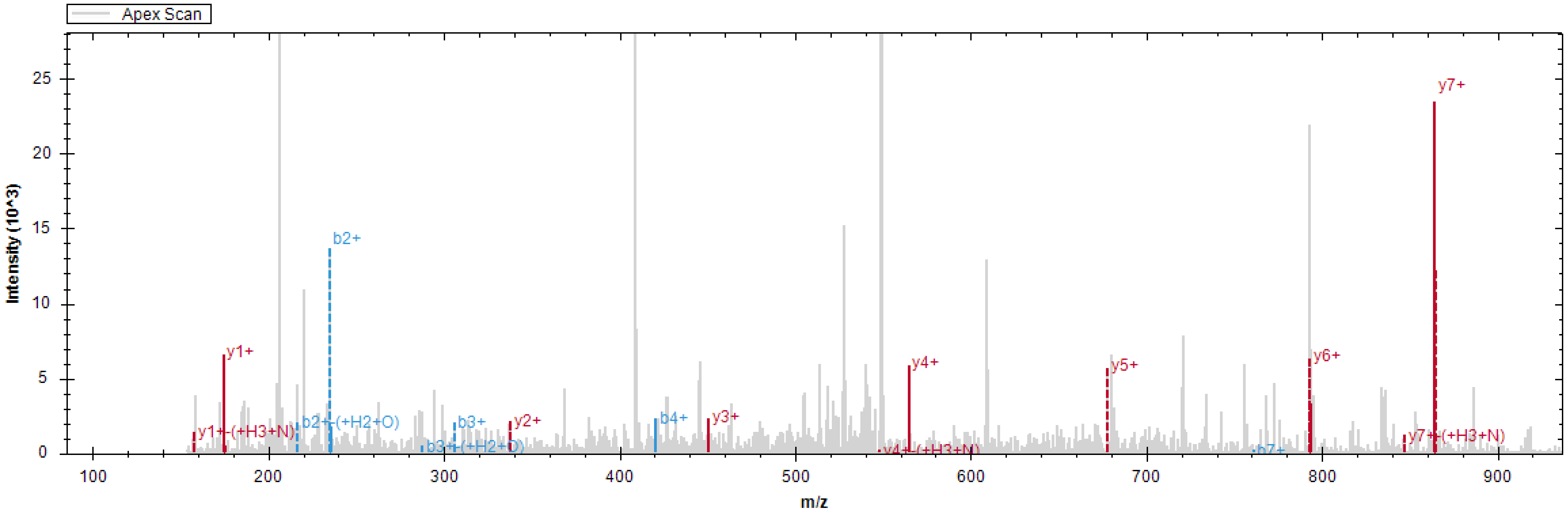
Apex Scan
Intensity (10^3)
m/z
y7+
b2+
y1+
y4+
y5+
y6+
y2+
y3+
b3+
b4+
y1+-(+H3+N)
b2+-(+H2+O)
b3+-(+H2+O)
y7+-(+H3+N)

P01344

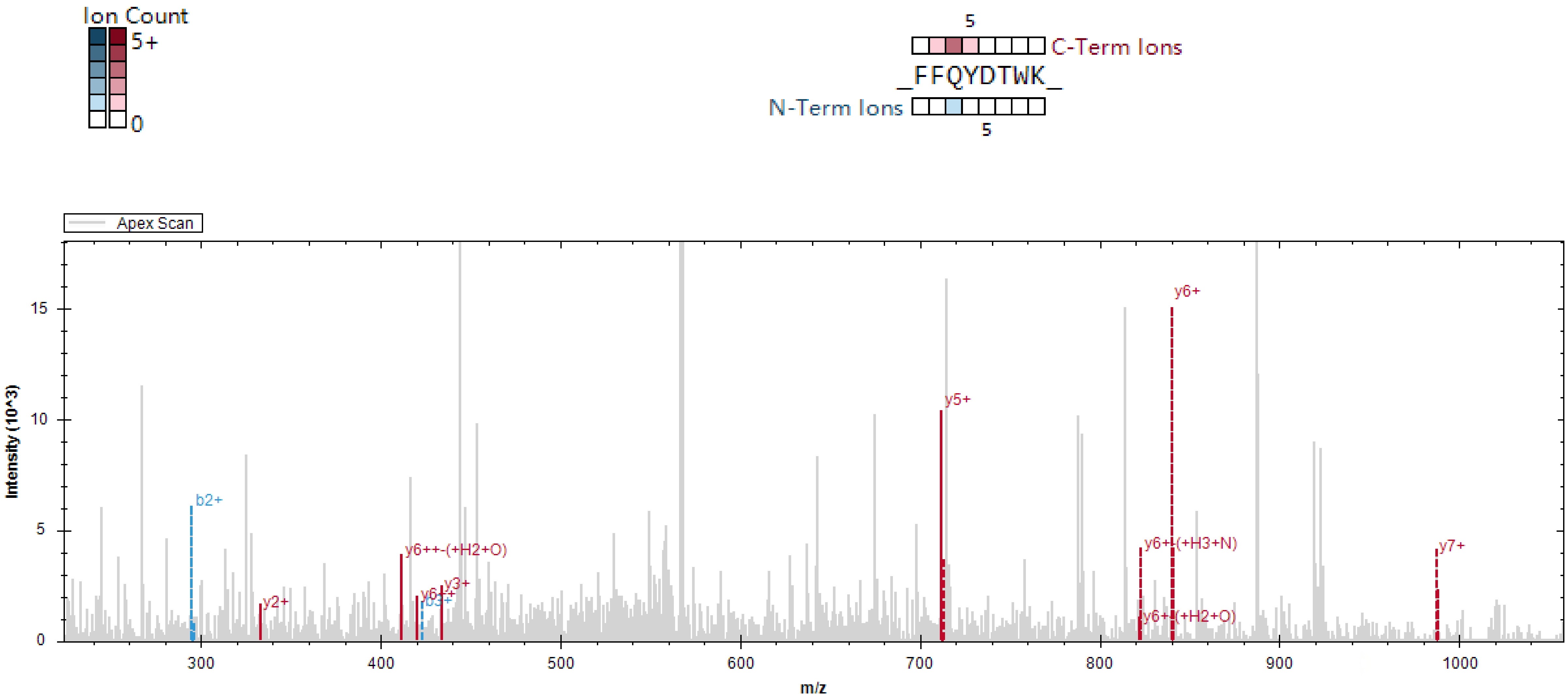

Ion Count
5+
0
5
C-Term Ions
_FFQYDTWK_
N-Term Ions
5
Apex Scan
Intensity (10^3)
m/z
b2+
y2+
y6++-(+H2+O)
y3+
b3+
y5+
y6+
y6+-(+H3+N)
y6+-(+H2+O)
y7+

P01782

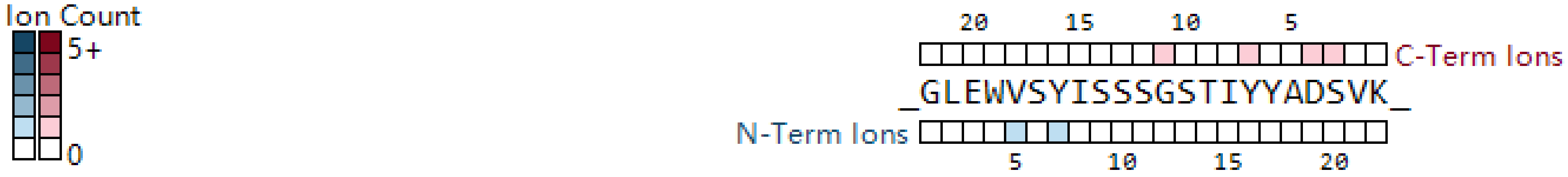

Ion Count
5+
0
20 15 10 5
C-Term Ions
_GLEWVSYISSSGSTIYYADSVK_
N-Term Ions
5 10 15 20


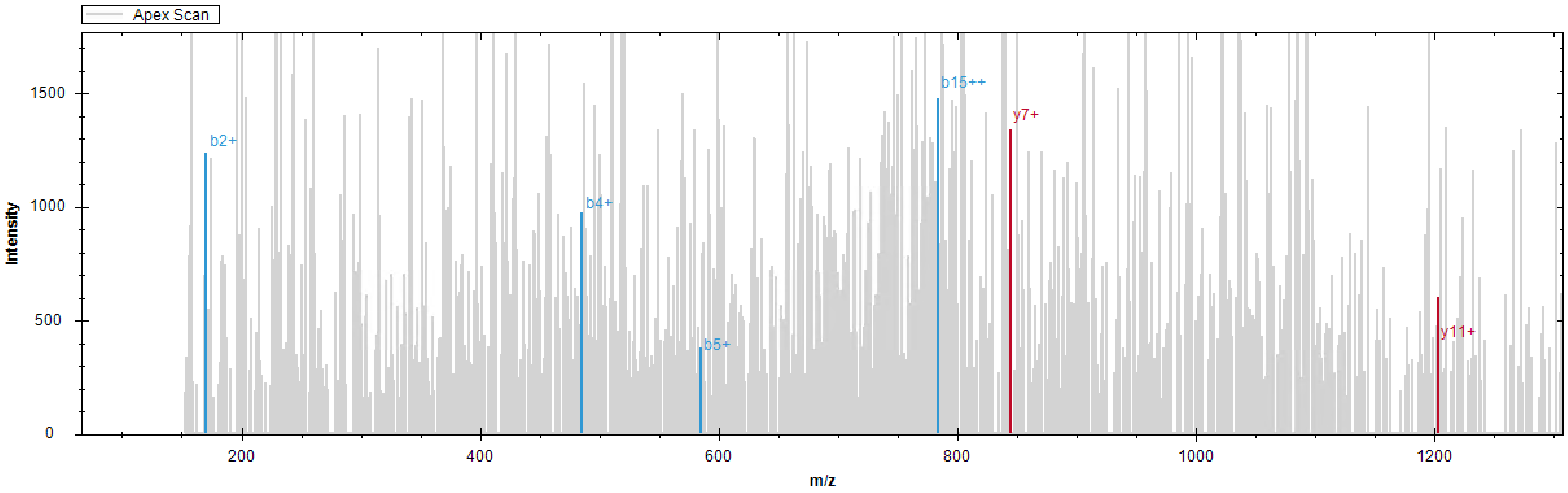

Apex Scan
b2+
b4+
b5+
b15++
y7+
y11+
Intensity
1500
1000
500
0
200
400
600
800
1000
1200
m/z

# P01857

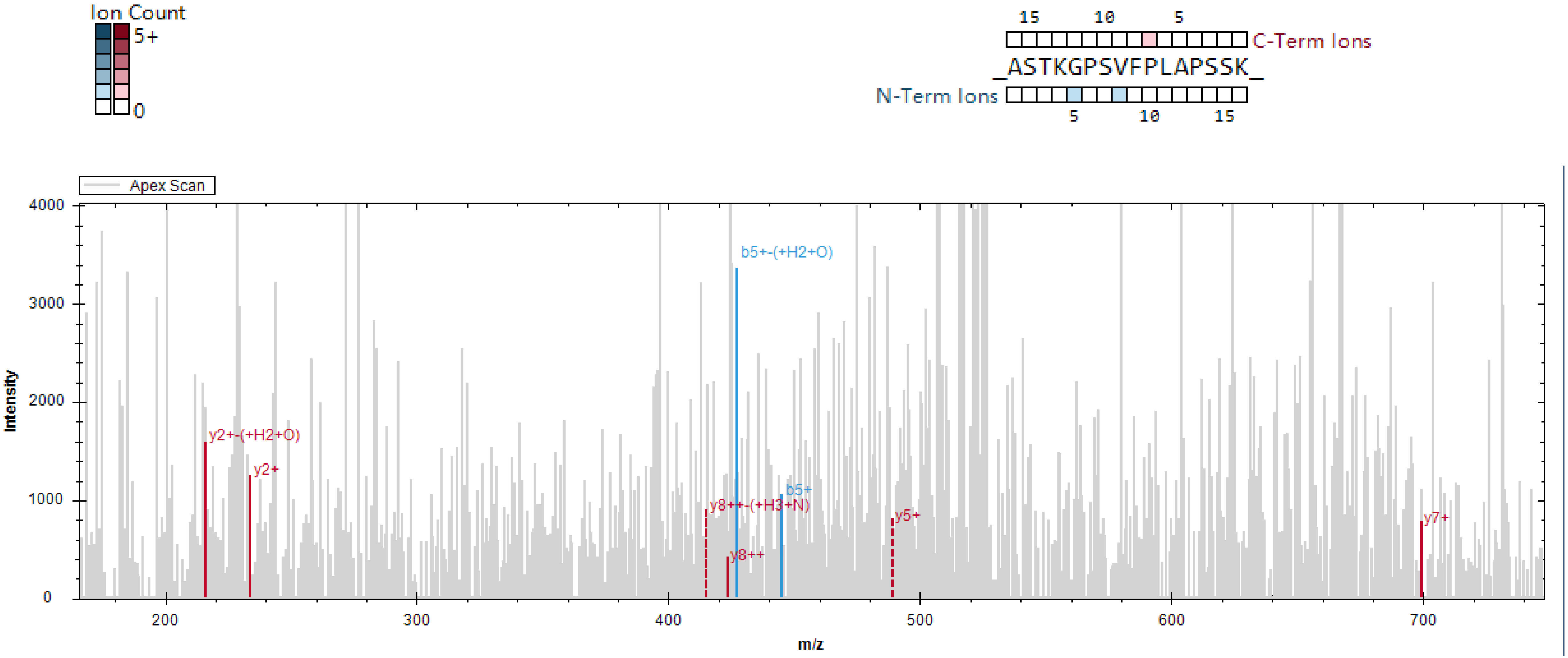

Ion Count
5+
0
15
10
5
C-Term Ions
_ASTKGPSVFPLAPSSK_
N-Term Ions
5
10
15
Apex Scan
4000
3000
2000
1000
0
Intensity
b5+-(+H2+O)
y2+-(+H2+O)
y2+
b5+
y8++-(+H3+N)
y5+
y8++
y7+
200
300
400
500
600
700
m/z

# P01909

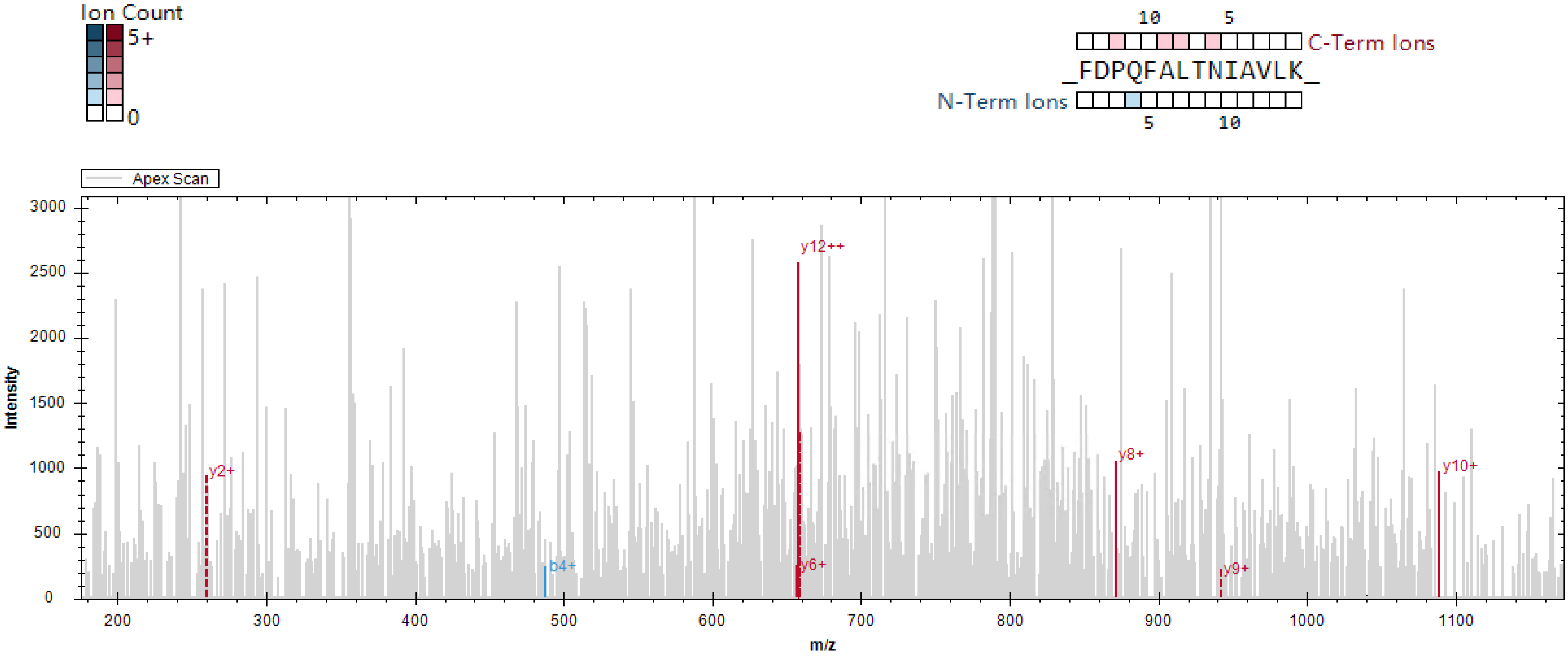

Ion Count
5+
0
10
5
C-Term Ions
_FDPQFALTNIAVLK_
N-Term Ions
5
10
Apex Scan
Intensity
m/z
y2+
b4+
y12++
y6+
y8+
y9+
y10+

P04211

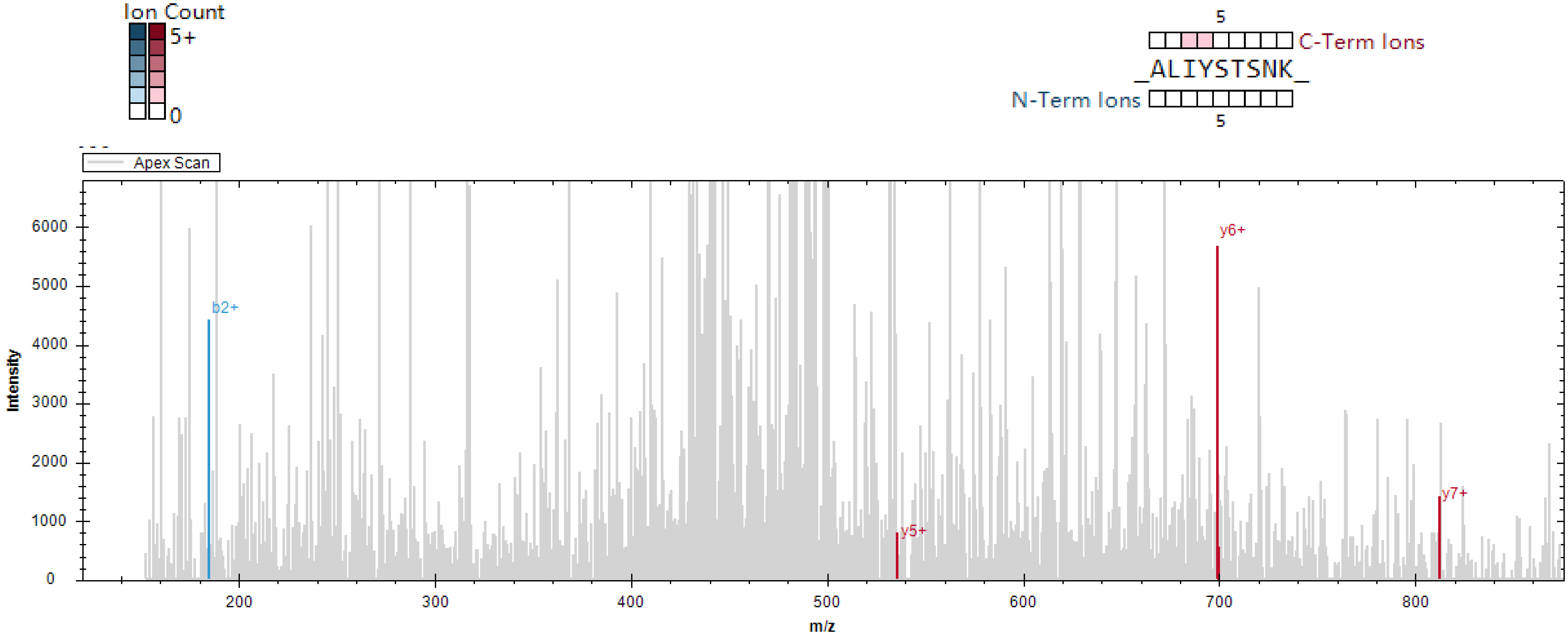

Ion Count
5+
0
5
C-Term Ions
_ALIYSTSNK_
N-Term Ions
5
Apex Scan
Intensity
m/z
b2+
y5+
y6+
y7+

P05386

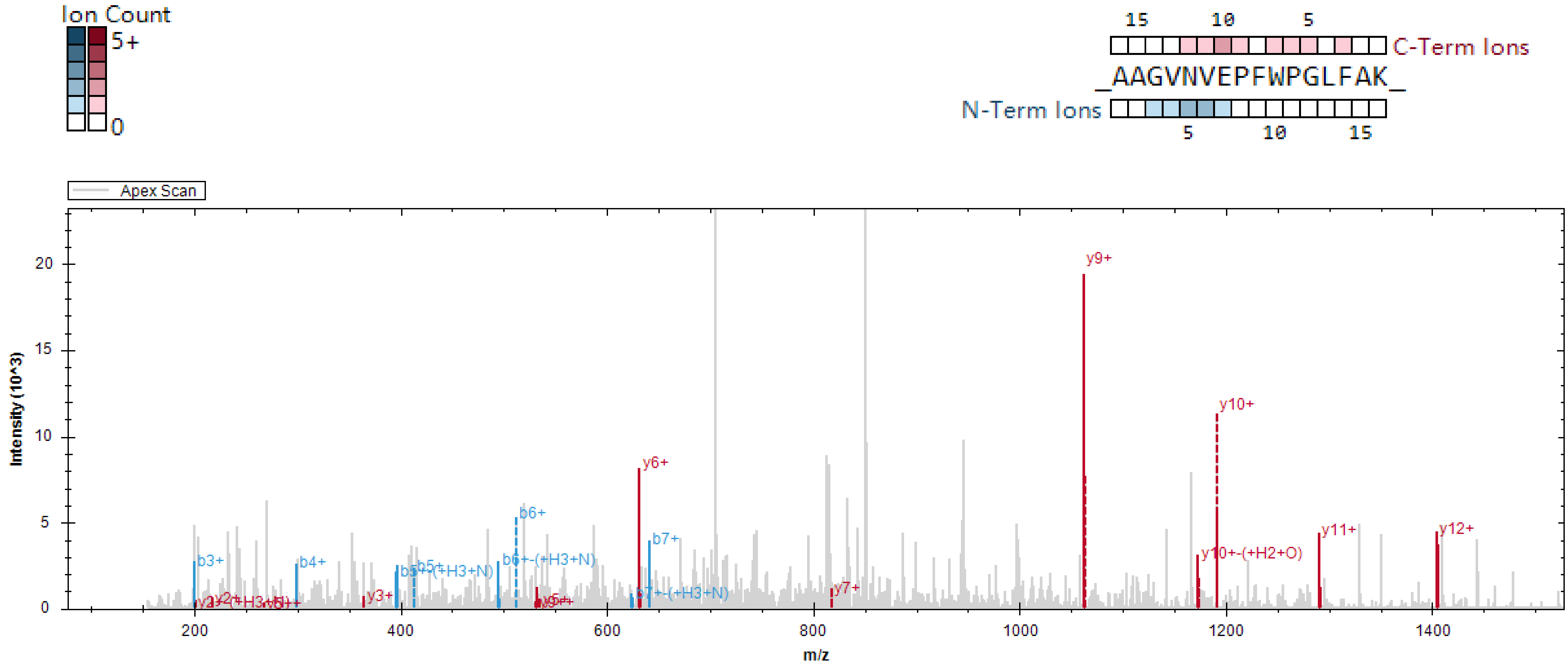

Ion Count
5+
0
15
10
5
C-Term Ions
_AAGVNVEPFWPGLFAK_
N-Term Ions
5
10
15
Apex Scan
Intensity (10^3)
m/z
y9+
y10+
y6+
b6+
b7+
y11+
y12+
b3+
b4+
y10+-(+H2+O)
b6+-(+H3+N)
b7+-(+H3+N)
y3+
y7+

# P06331

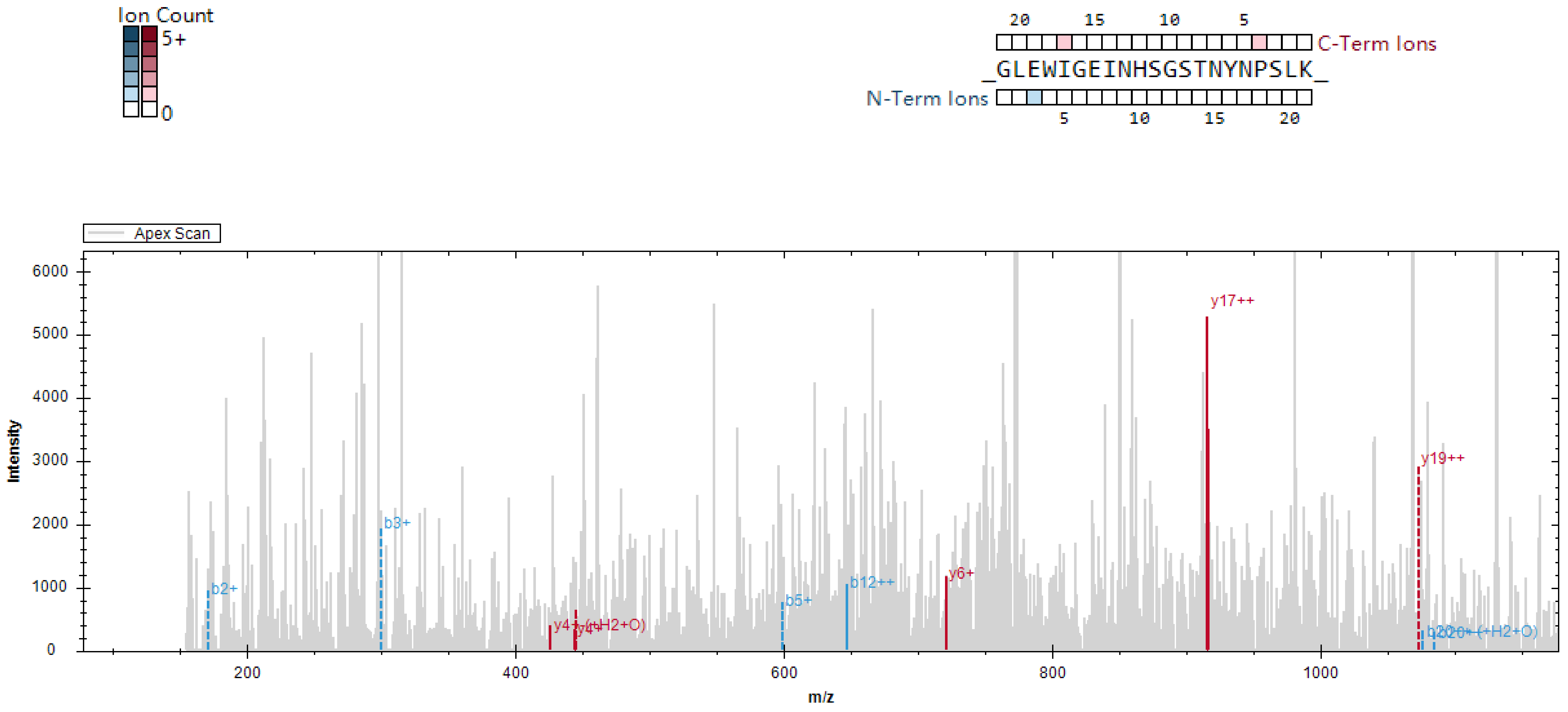

Ion Count
5+
0
20 15 10 5
C-Term Ions
_GLEWIGEINHSGSTNYNPSLK_
N-Term Ions
5 10 15 20
Apex Scan
Intensity
m/z
y17++
y19++
b3+
b2+
y4+-(+H2+O)
y4+
b5+
b12++
y6+
b20+-(+H2+O)

# P07327

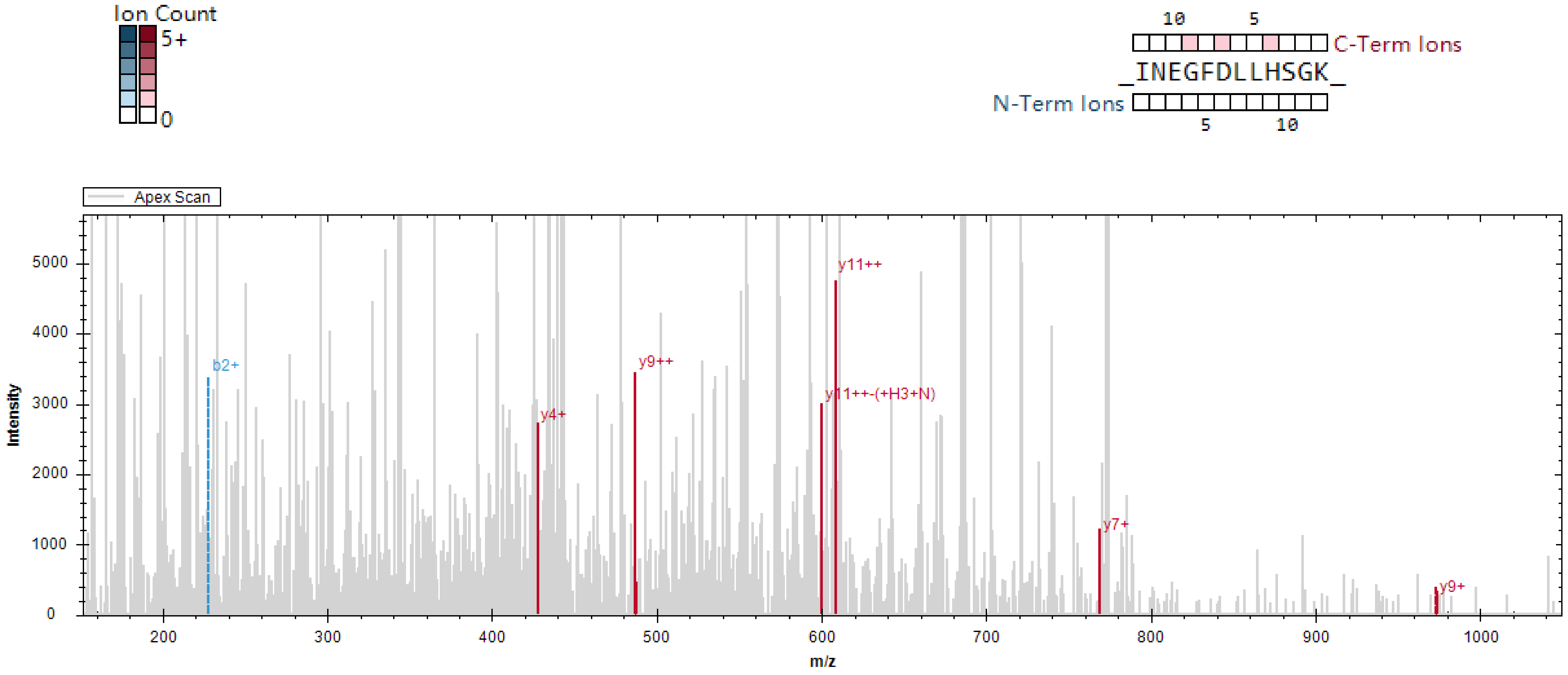
Ion Count
5+
0
10
5
C-Term Ions
_INEGFDLLHSGK_
N-Term Ions
5
10
Apex Scan
Intensity
m/z
b2+
y4+
y9++
y11++-(+H3+N)
y11++
y7+
y9+

P08174

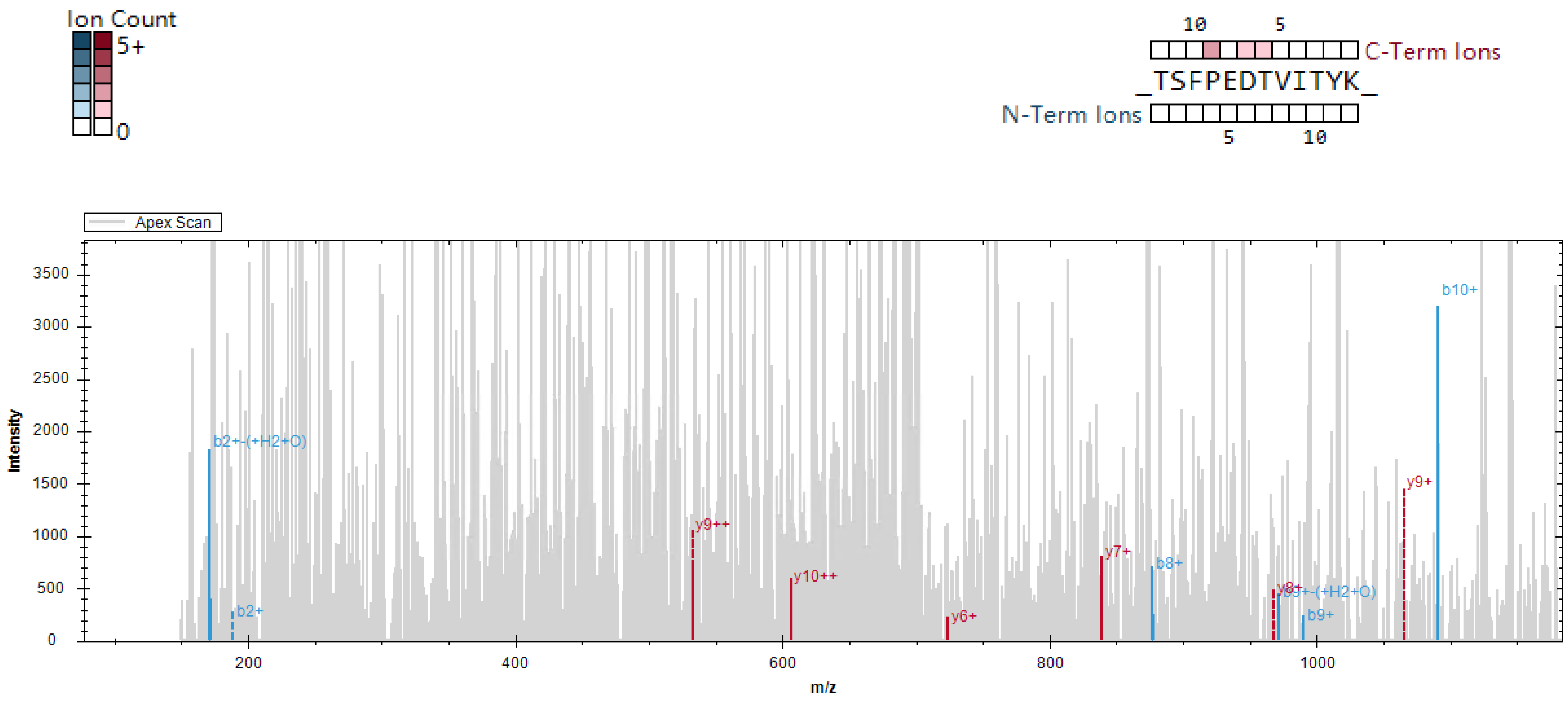

Ion Count
5+
0
10
5
C-Term Ions
_TSFPEDTVITYK_
N-Term Ions
5
10
Apex Scan
Intensity
m/z
b2+-(+H2+O)
b2+
y9++
y10++
y6+
y7+
b8+
y8+
b9+-(+H2+O)
b9+
y9+
b10+

# P08572

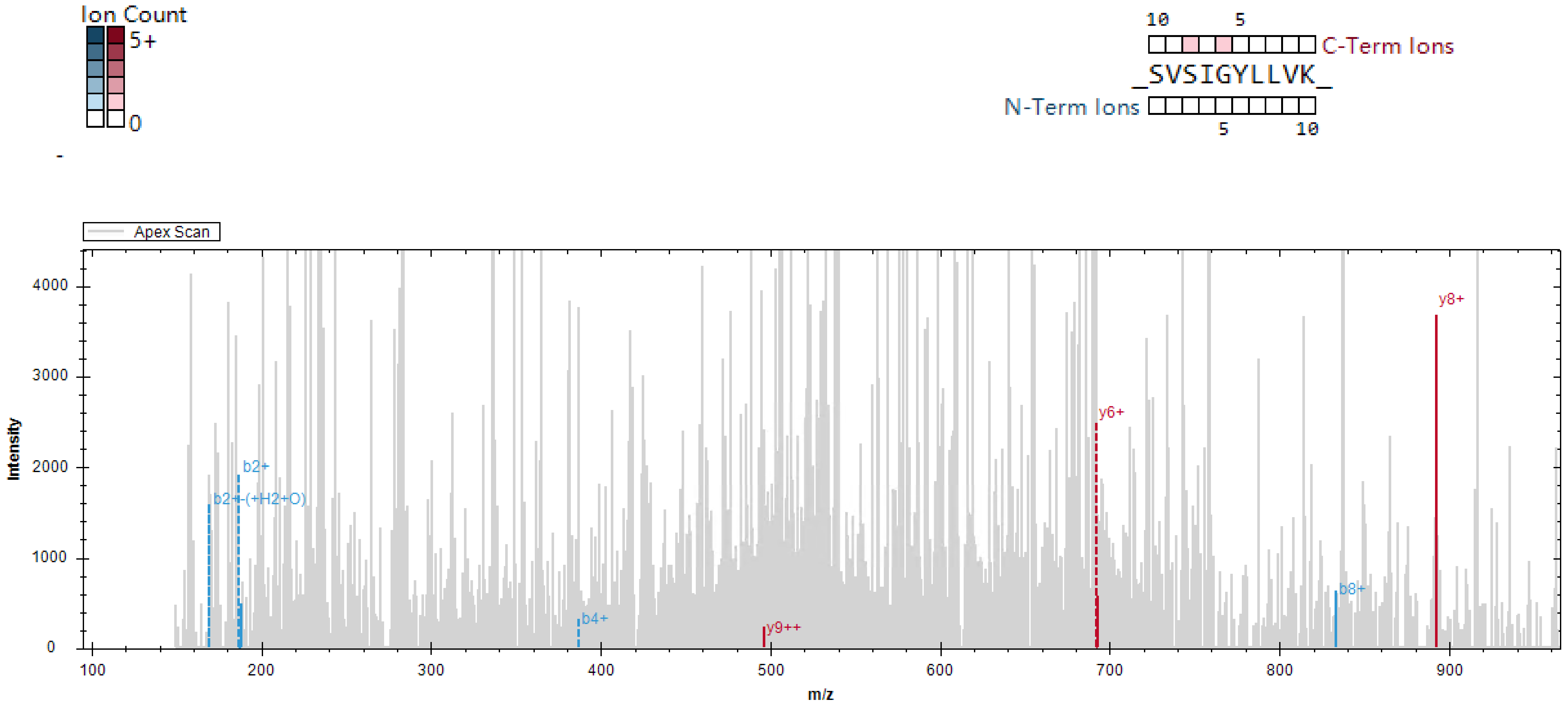

Ion Count
5+
0
10
5
C-Term Ions
_SVSIGYLLVK_
N-Term Ions
5
10
Apex Scan
Intensity
m/z
b2+
b2+-(+H2+O)
b4+
y9++
y6+
b8+
y8+

P08590

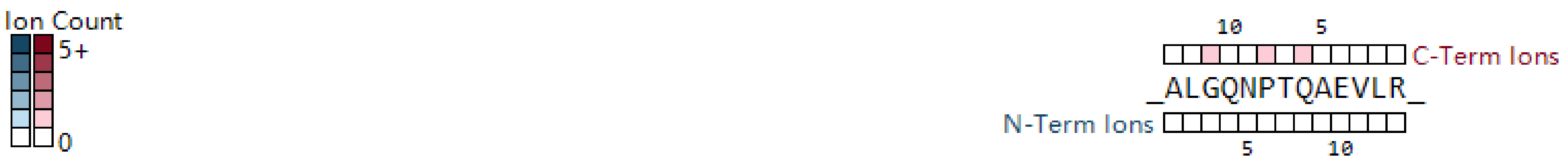
Ion Count
5+
0
10
5
C-Term Ions
_ALGQNPTQAEVLR_
N-Term Ions
5
10

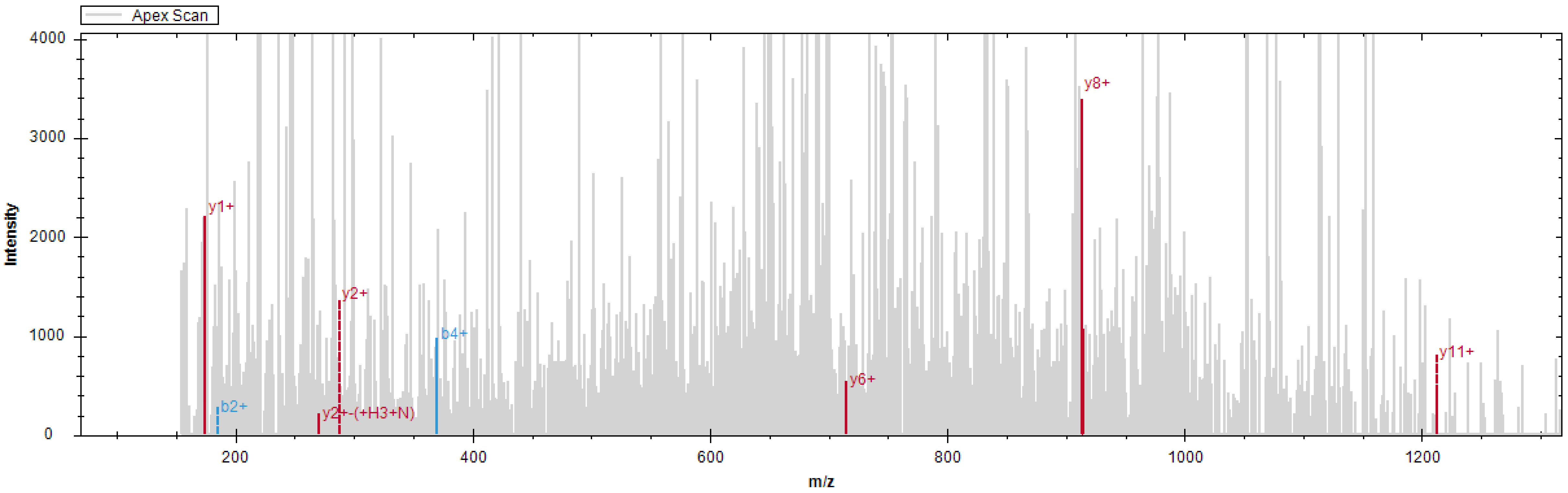
Apex Scan
y1+
b2+
y2+
y2+-(+H3+N)
b4+
y6+
y8+
y11+
4000
3000
2000
1000
0
200
400
600
800
1000
1200
Intensity
m/z

P09758

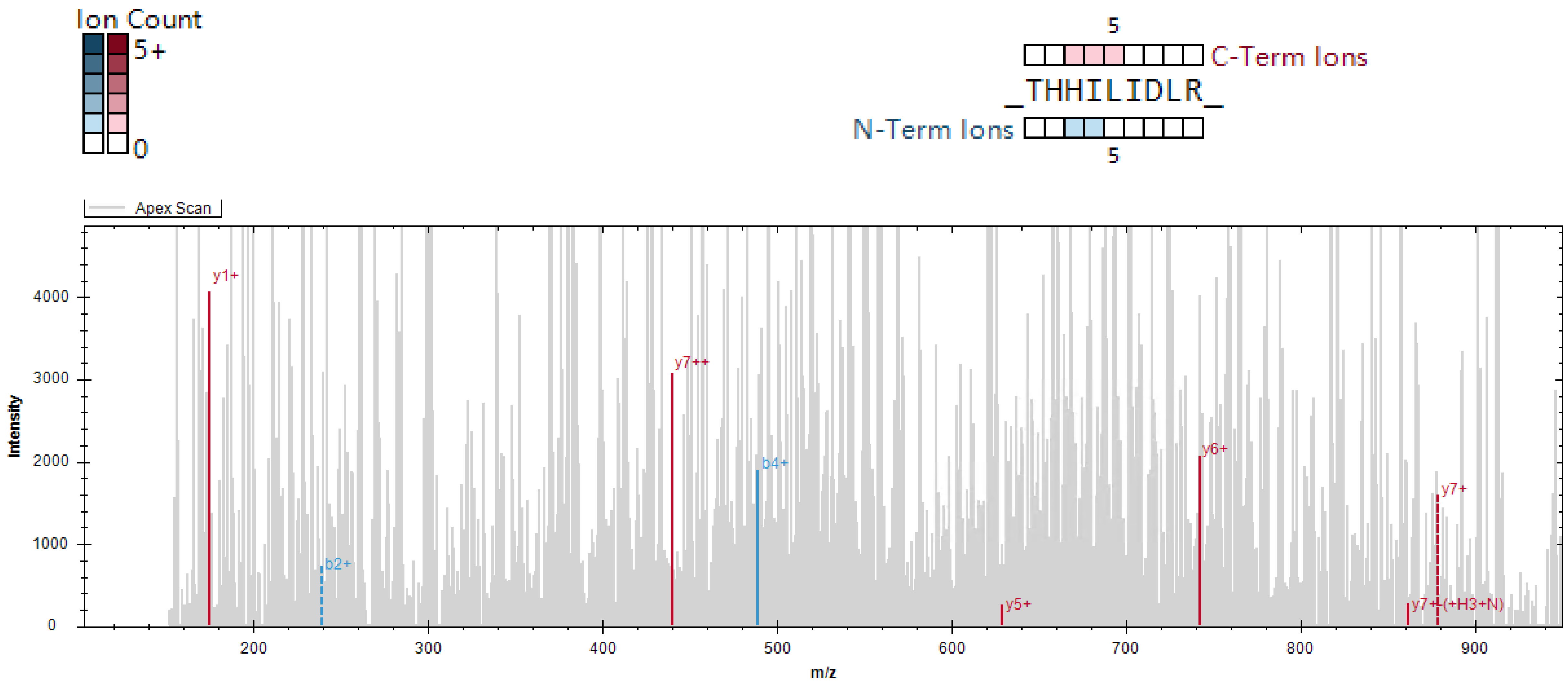

Ion Count
5+
0
5
C-Term Ions
_THHILIDLR_
N-Term Ions
5
Apex Scan
y1+
y7++
b4+
b2+
y5+
y6+
y7+
y7+-(+H3+N)
Intensity
m/z

P0DMQ5

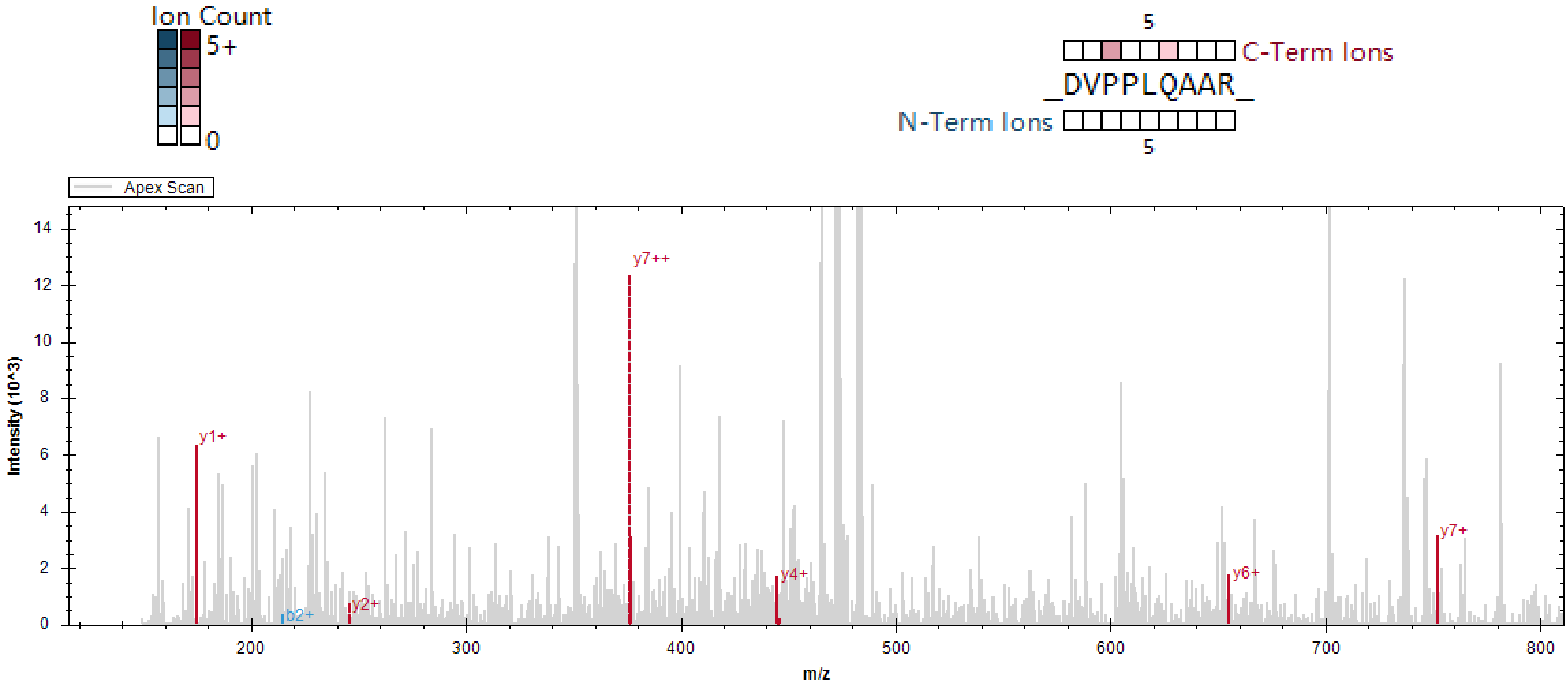
Ion Count
5+
0
5
C-Term Ions
_DVPPLQAAR_
N-Term Ions
5
Apex Scan
y7++
y1+
y4+
y6+
y7+
y2+
b2+
Intensity (10^3)
m/z

P0DOX8

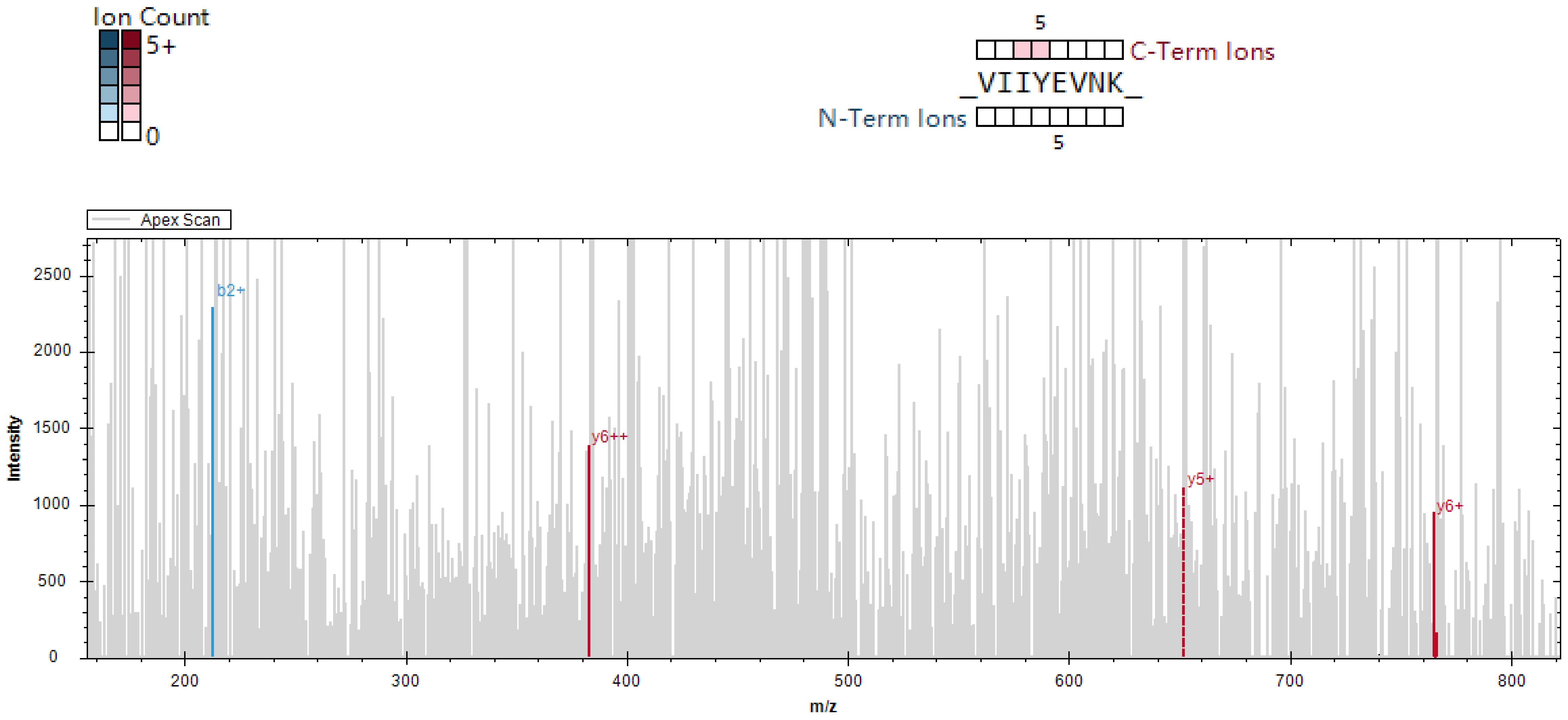

Ion Count
5+
0
5
C-Term Ions
_VIIYEVNK_
N-Term Ions
5
Apex Scan
b2+
y6++
y5+
y6+
Intensity
m/z

P10242

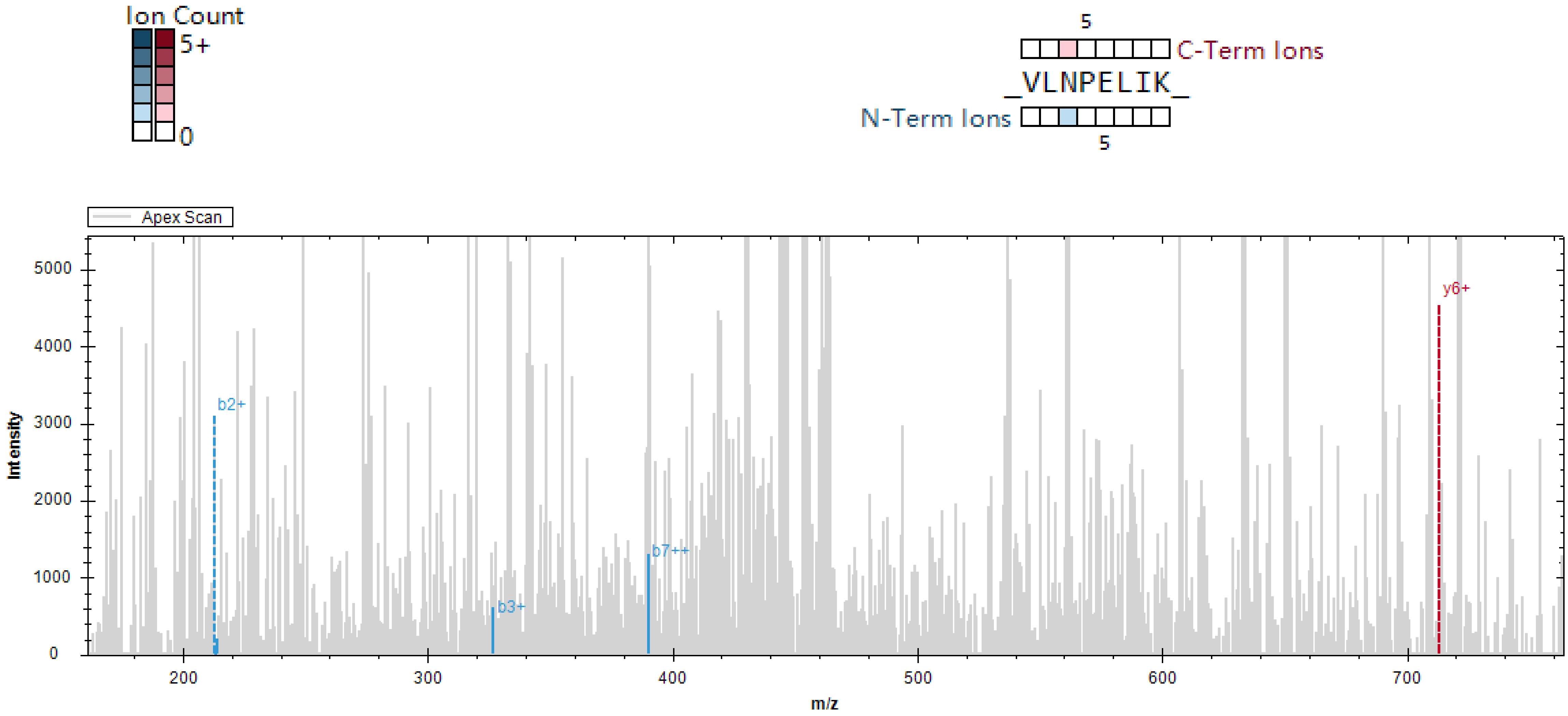

Ion Count
5+
0
5
C-Term Ions
_VLNPELIK_
N-Term Ions
5
Apex Scan
Intensity
m/z
b2+
b3+
b7++
y6+

P11049

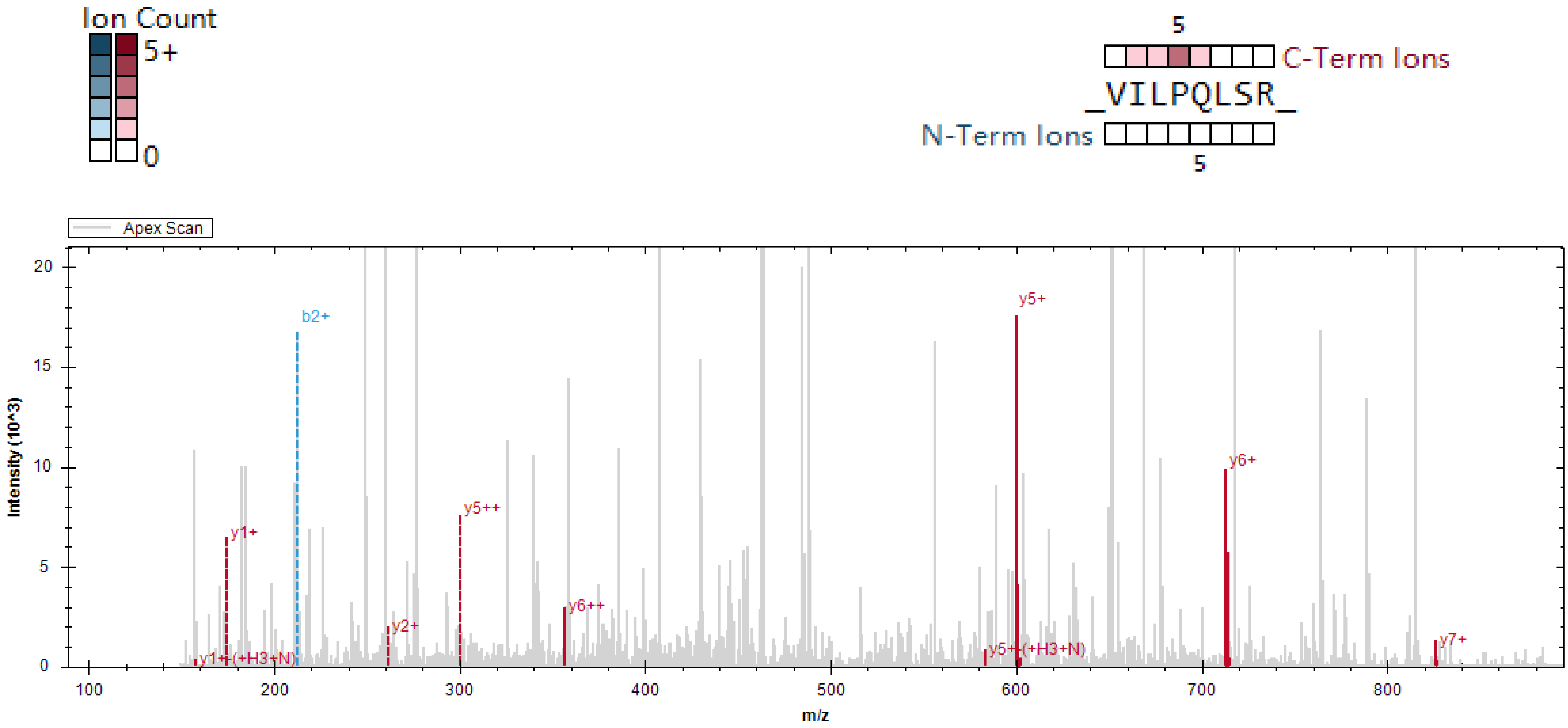

Ion Count
5+
0
5
C-Term Ions
_VILPQLSR_
N-Term Ions
5
Apex Scan
Intensity (10^3)
m/z
b2+
y5+
y6+
y5++
y1+
y6++
y2+
y1+-(+H3+N)
y5+-(+H3+N)
y7+

P13164

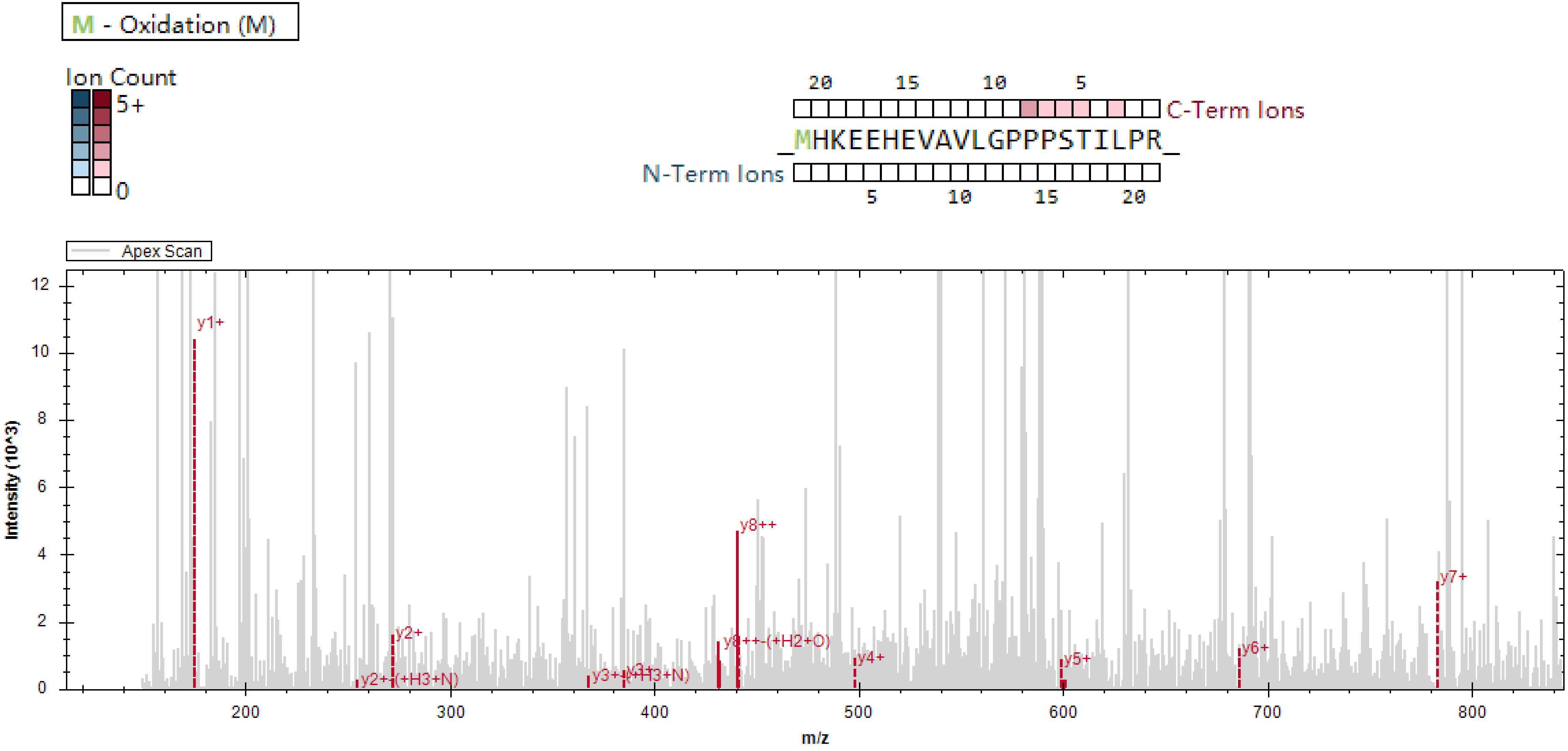

M - Oxidation (M)
Ion Count
5+
0
20 15 10 5
C-Term Ions
_MHKEEHEVAVLGPPPSTILPR_
N-Term Ions
5 10 15 20
Apex Scan
Intensity (10^3)
m/z
y1+
y2+
y2++(+H3+N)
y3++(+H3+N)
y8++
y8++-(+H2+O)
y4+
y5+
y6+
y7+

P13762

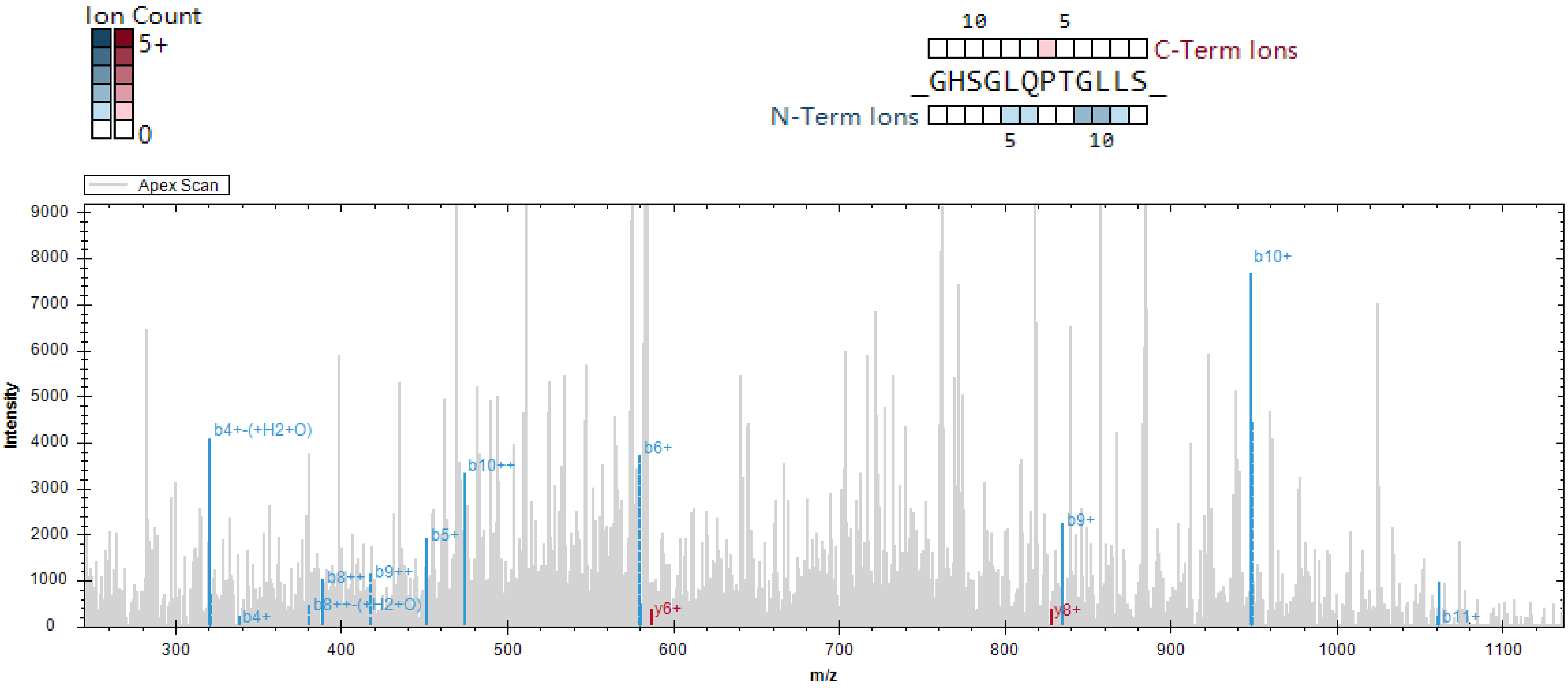

Ion Count
5+
0
10
5
C-Term Ions
_GHSGLQPTGLLS_
N-Term Ions
5
10
Apex Scan
b10+
b4+-(+H2+O)
b6+
b10++
b5+
b9+
b8++
b9++
b8++-(+H2+O)
b4+
y6+
y8+
b11+
Intensity
m/z

P14207

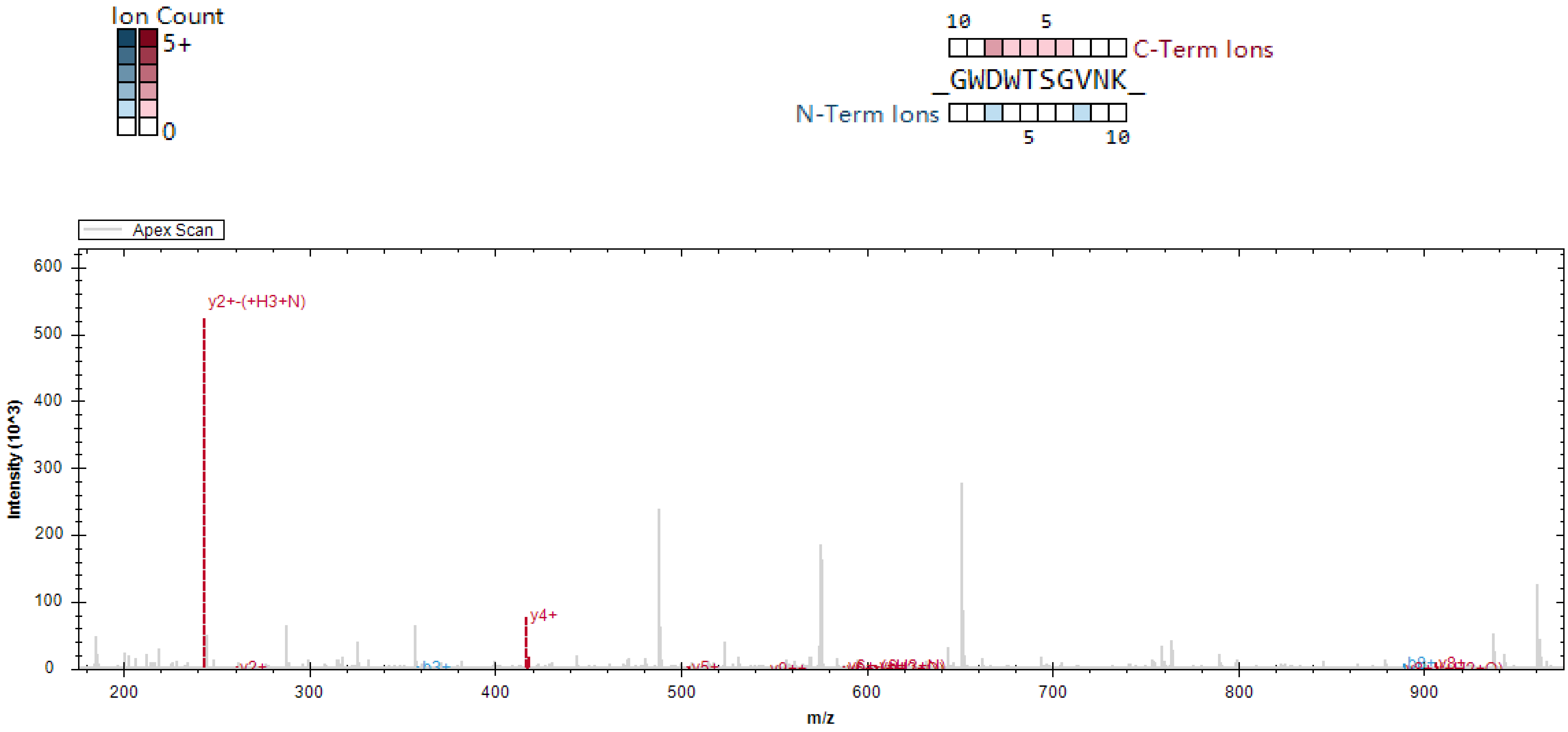
Ion Count
5+
0
10
5
C-Term Ions
_GWDWTSGVNK_
N-Term Ions
5
10
Apex Scan
y2+-(+H3+N)
y4+
Intensity (10^3)
m/z

P15172

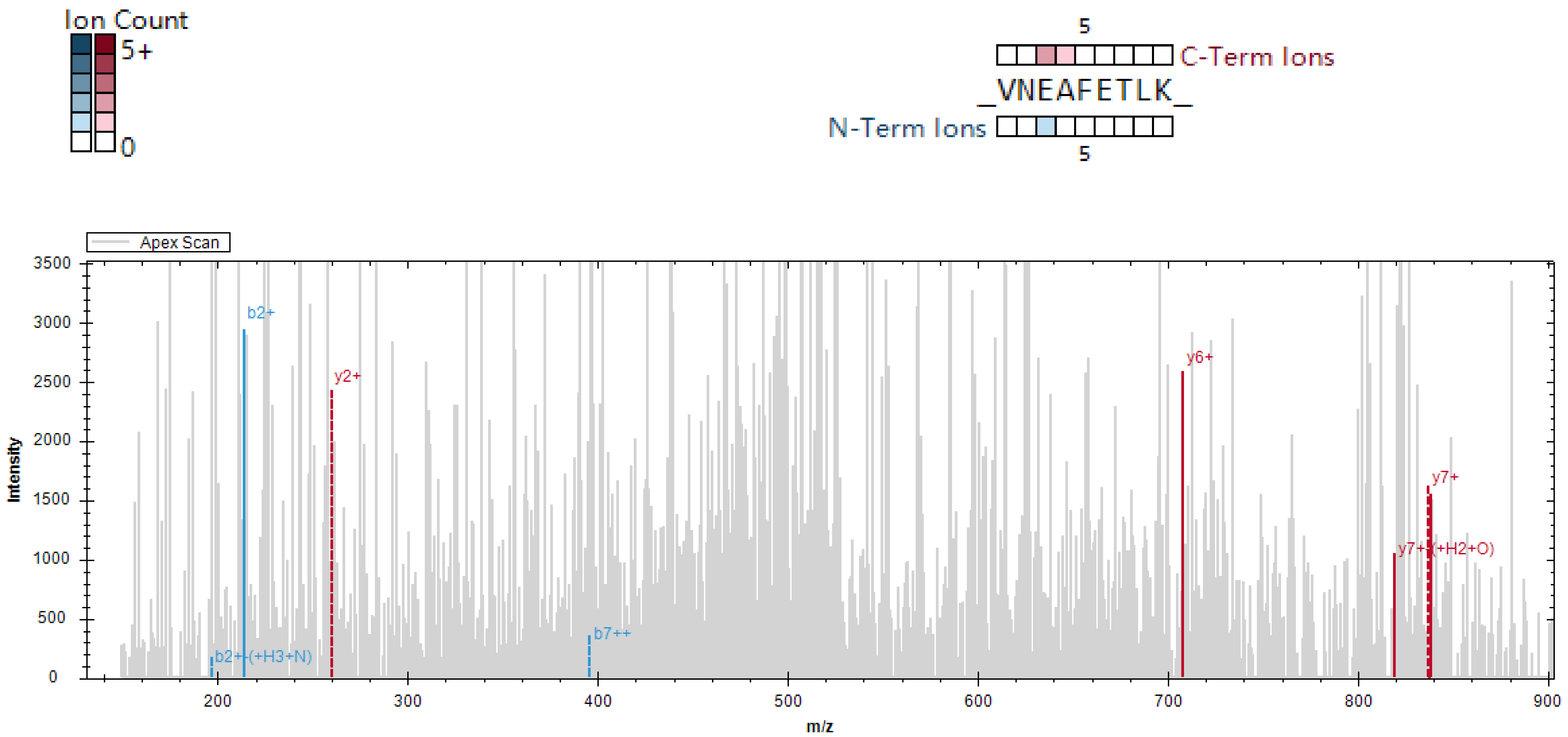

Ion Count
5+
0
5
C-Term Ions
_VNEAFETLK_
N-Term Ions
5
Apex Scan
Intensity
m/z
b2+
y2+
y6+
y7+
y7+-(+H2+O)
b7++
b2+-(+H3+N)

P15516

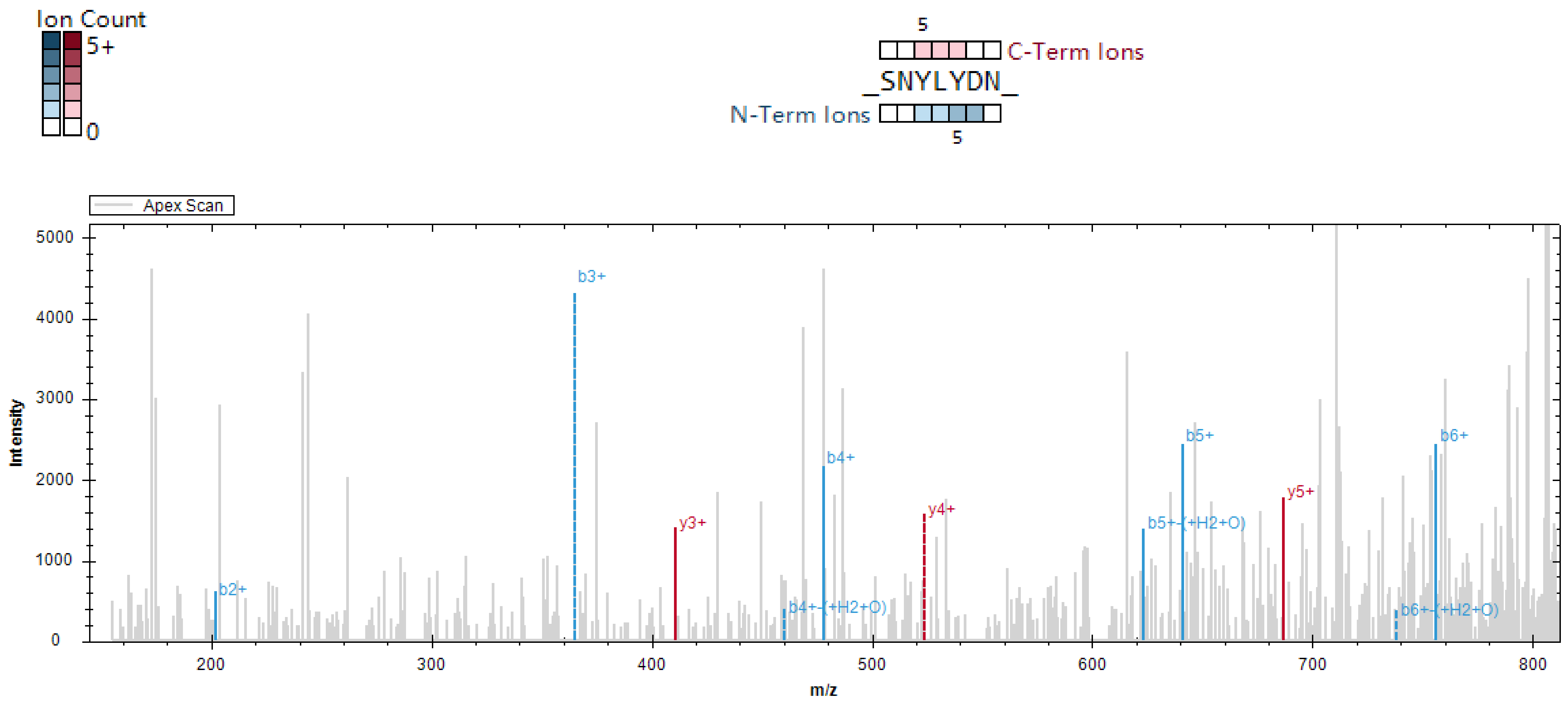

Ion Count
5+
0
5
C-Term Ions
_SNYLYDN_
N-Term Ions
5
Apex Scan
Intensity
m/z
5000
4000
3000
2000
1000
0
200
300
400
500
600
700
800
b2+
b3+
y3+
b4+-(+H2+O)
b4+
y4+
b5+-(+H2+O)
b5+
y5+
b6+-(+H2+O)
b6+

P19075

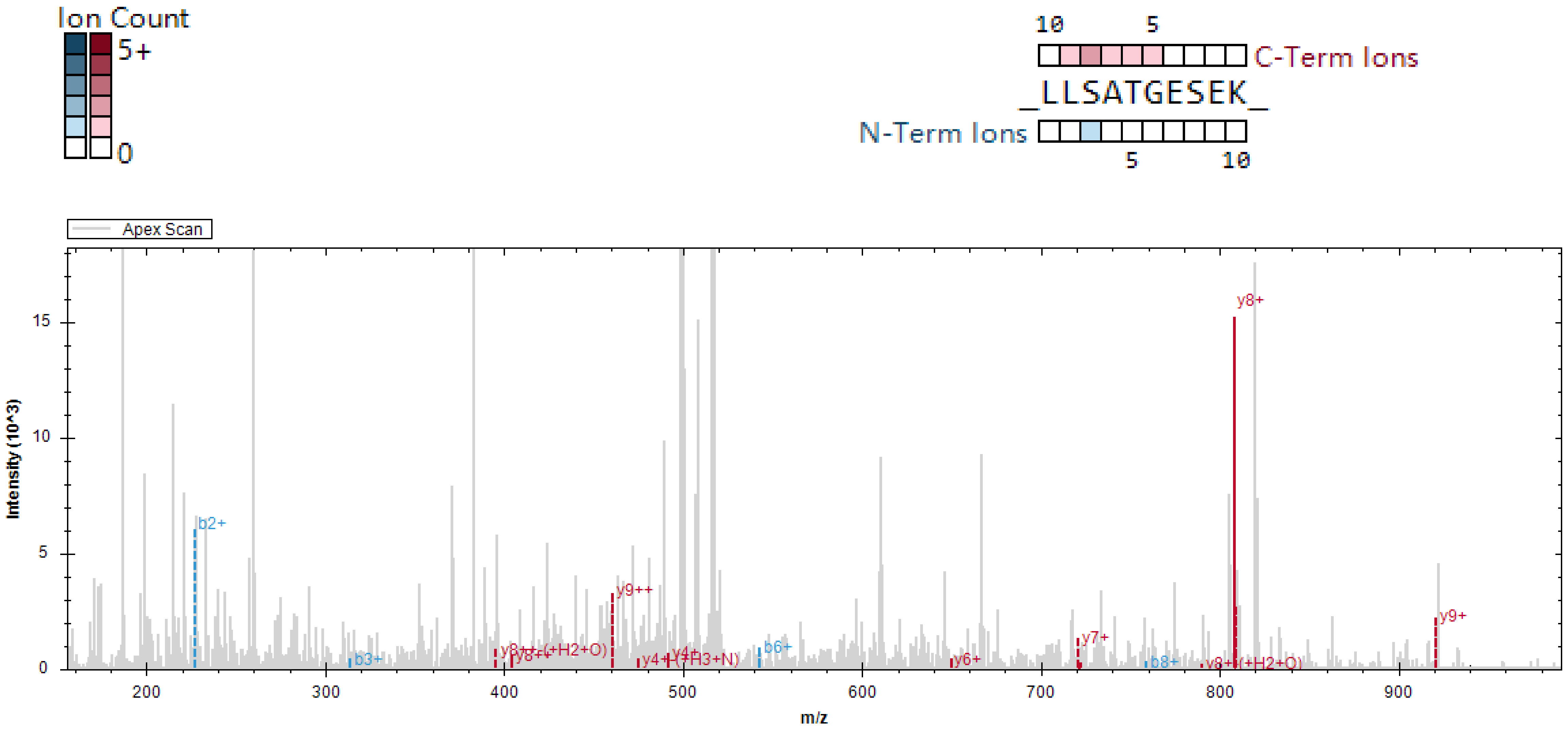

Ion Count
5+
0
10
5
C-Term Ions
_LLSATGESEK_
N-Term Ions
5
10
Apex Scan
y8+
b2+
y9++
b6+
b3+
y8++(+H2O)
y8++
y4+
y4+(+H3+N)
y6+
y7+
b8+
y8+(+H2O)
y9+
Intensity (10^3)
m/z

# P19440

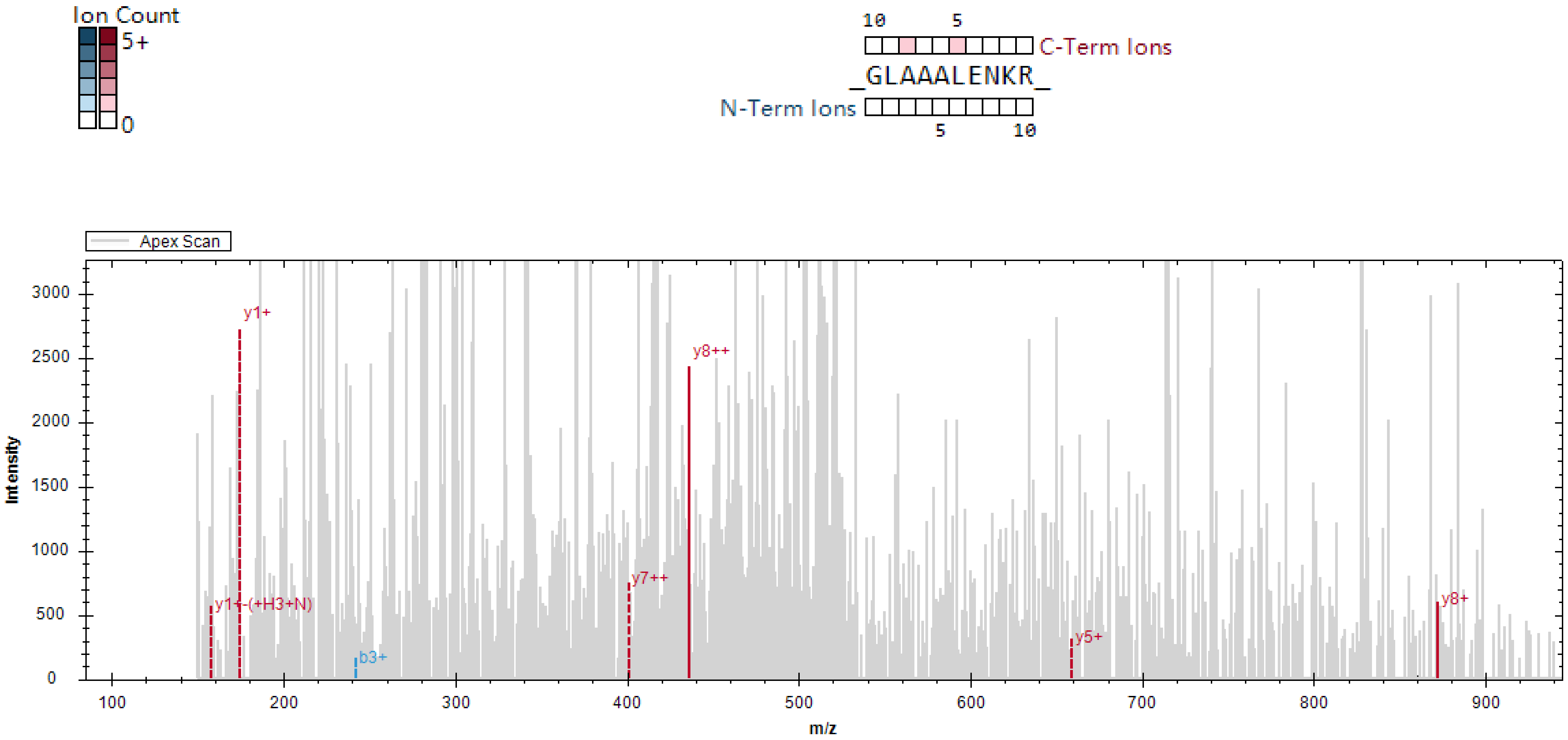

Ion Count
5+
0
10
5
C-Term Ions
_GLAAALENKR_
N-Term Ions
5
10
Apex Scan
y1+
y8++
y1+-(+H3+N)
b3+
y7++
y5+
y8+
Intensity
m/z
3000
2500
2000
1500
1000
500
0
100
200
300
400
500
600
700
800
900

P19447

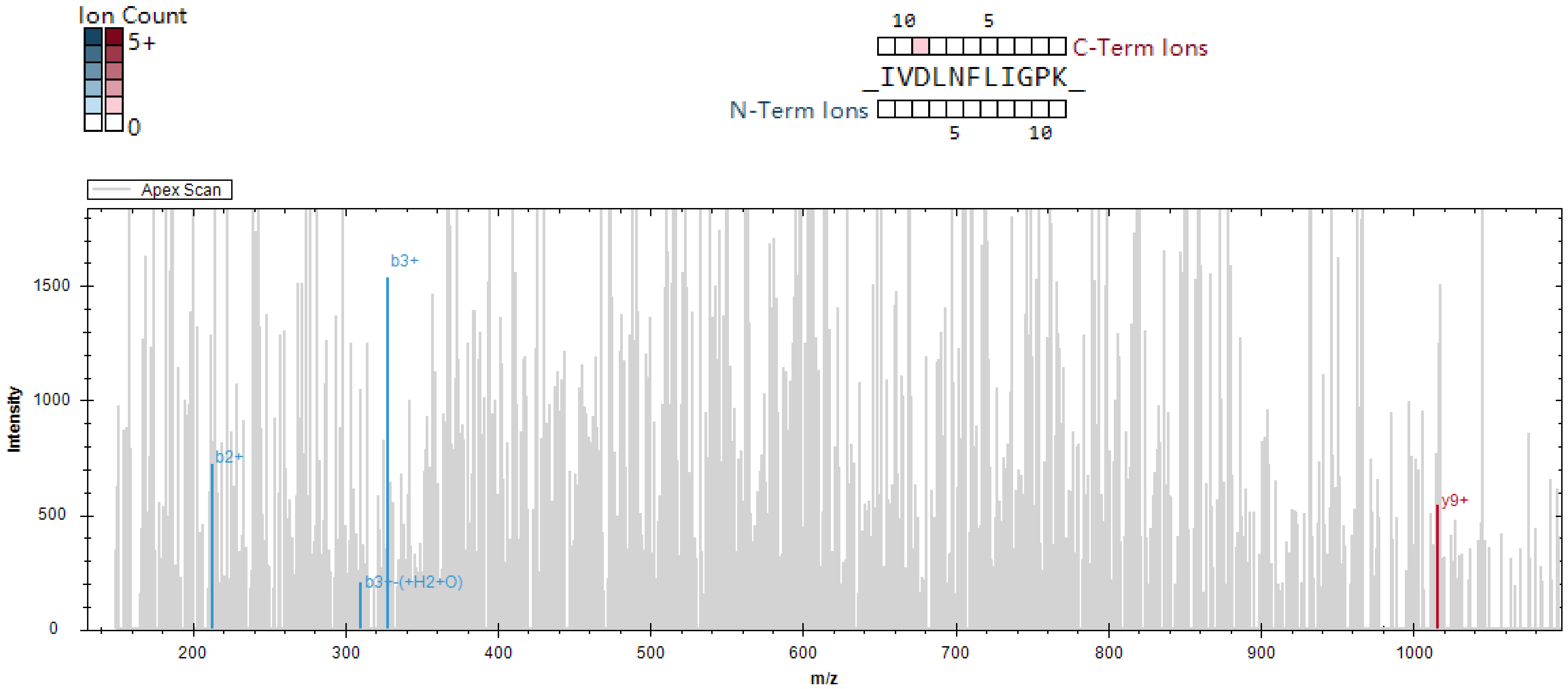
Ion Count
5+
0
10
5
C-Term Ions
_IVDLNFLIGPK_
N-Term Ions
5
10
Apex Scan
b3+
b2+
b3+-(+H2+O)
y9+
Intensity
m/z

P19883

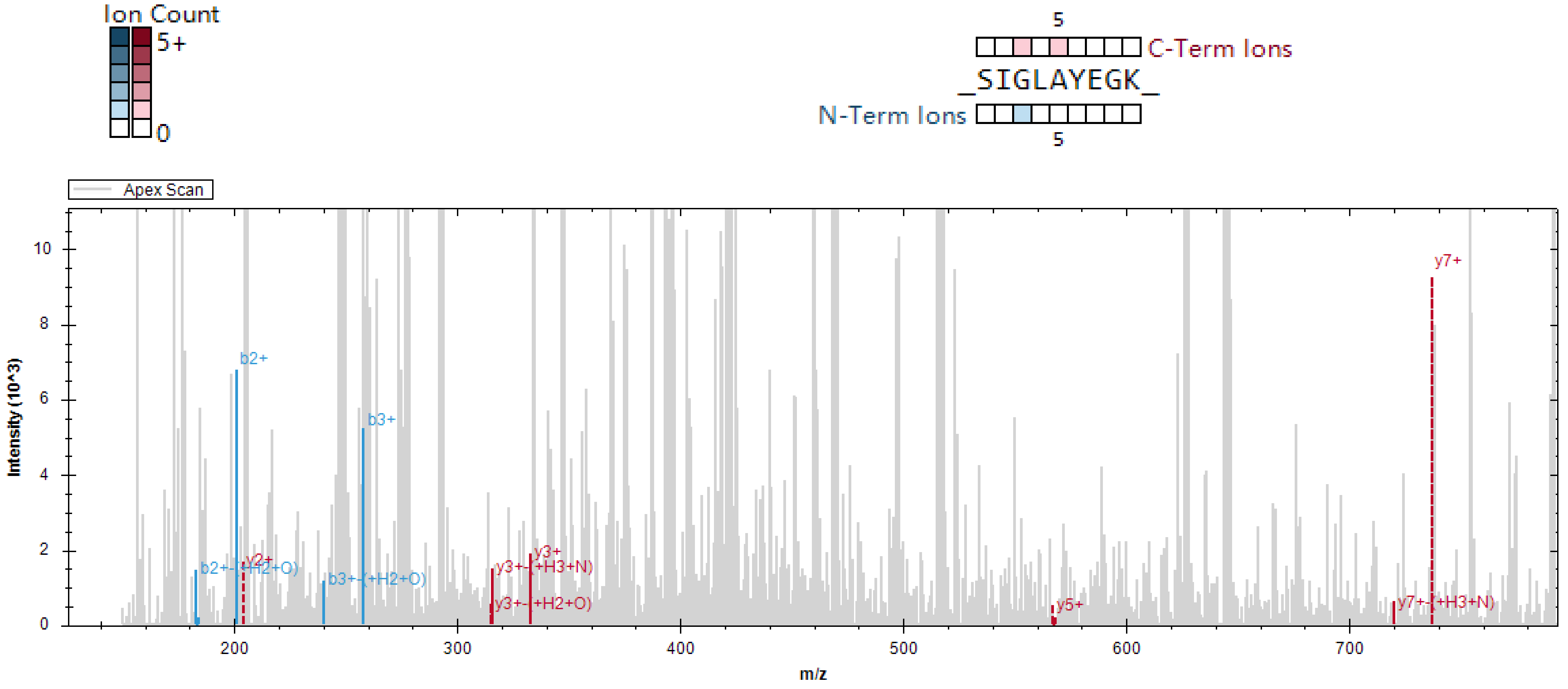
Ion Count
5+
0
5
C-Term Ions
_SIGLAYEGK_
N-Term Ions
5
Apex Scan
Intensity (10^3)
m/z
b2+
b3+
b2+-(+H2+O)
y2+
b3+-(+H2+O)
y3+
y3+-(+H3+N)
y3+-(+H2+O)
y5+
y7+
y7+-(+H3+N)

# P20036

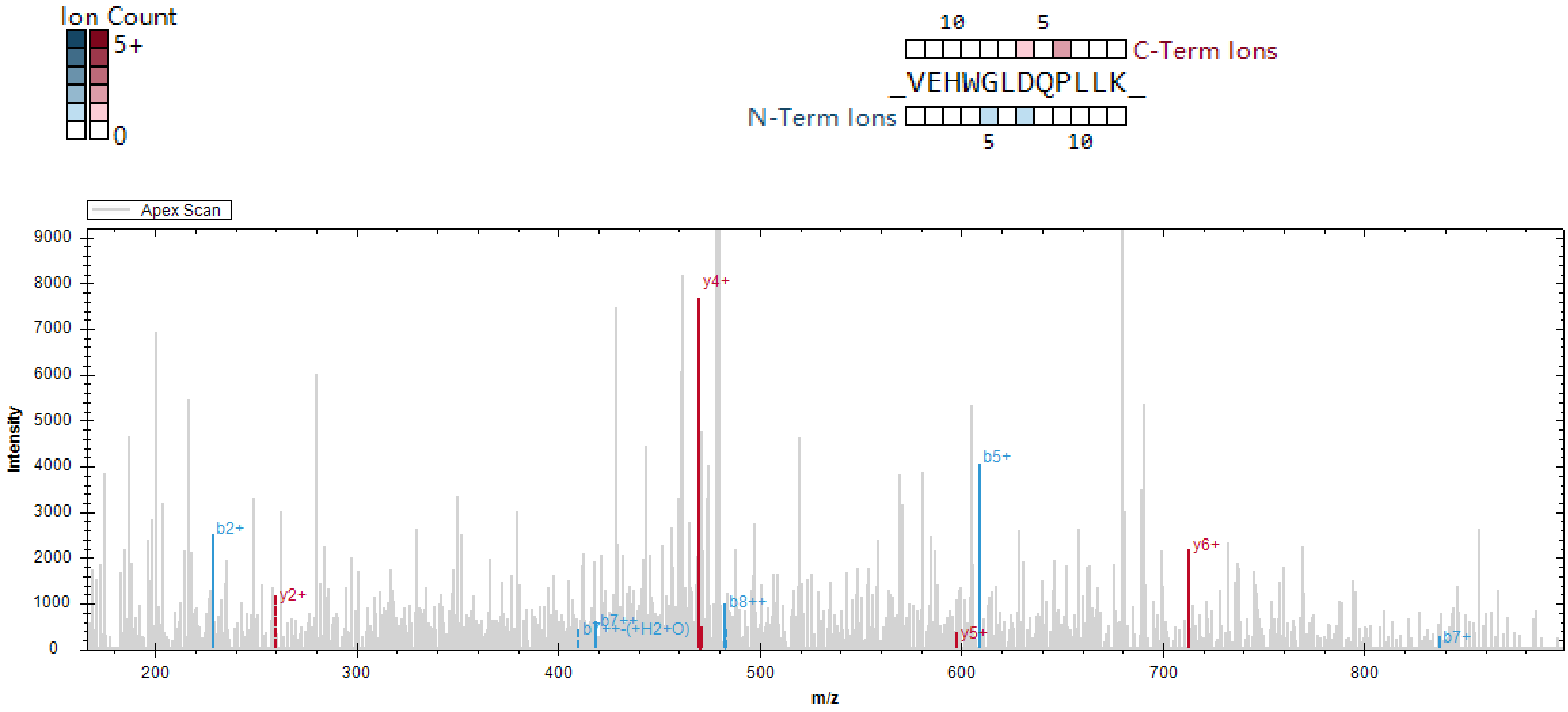

Ion Count
5+
0
10
5
C-Term Ions
_VEHWGLDQPLLK_
N-Term Ions
5
10
Apex Scan
Intensity
m/z
y4+
b5+
b2+
y2+
y6+
b8++
y5+
b7+

P20292

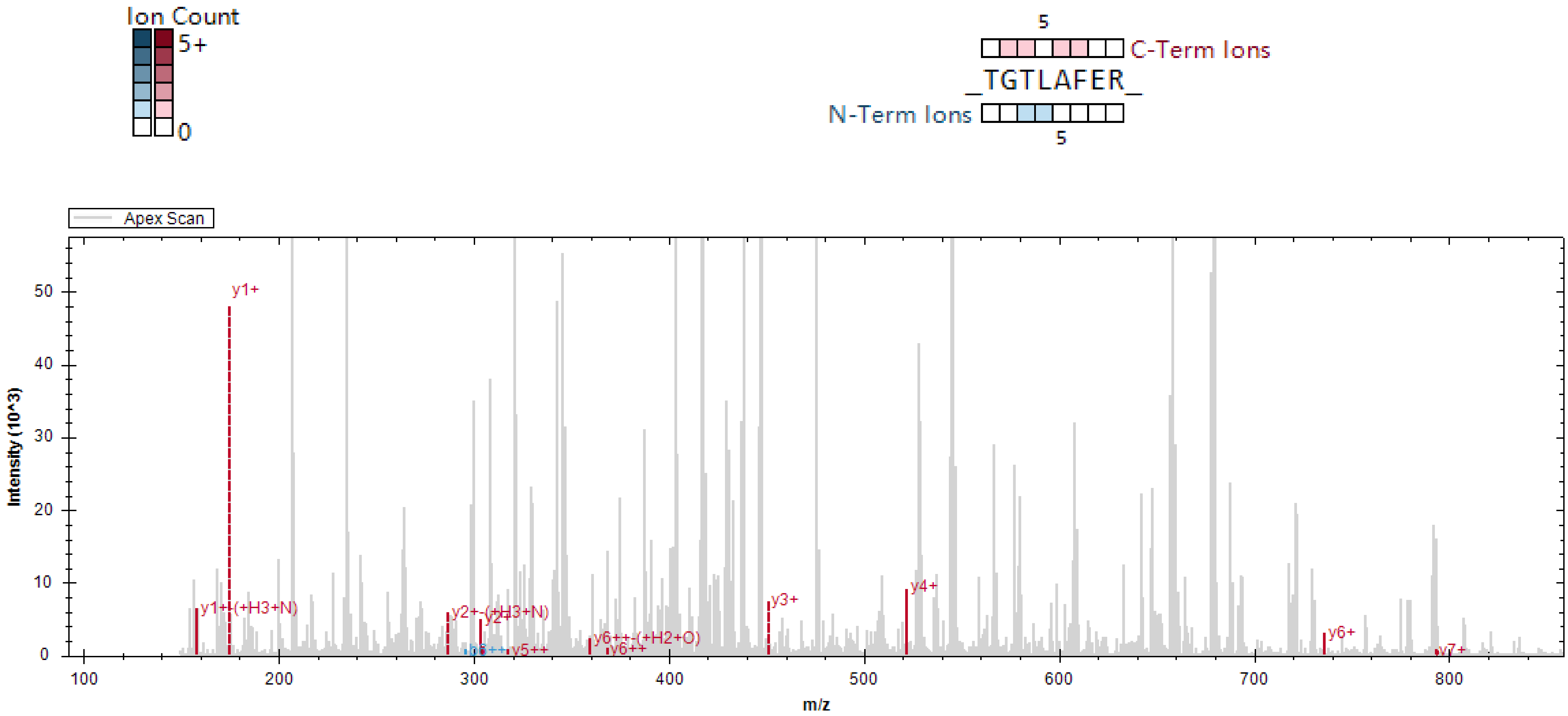

Ion Count
5+
0
5
C-Term Ions
_TGTLAFER_
N-Term Ions
5
Apex Scan
y1+
y1+-(+H3+N)
y2+-(+H3+N)
y2+
y5++
y3+
y4+
y6++-(+H2+O)
y6++
y6+
y7+
Intensity (10^3)
m/z

# P21397

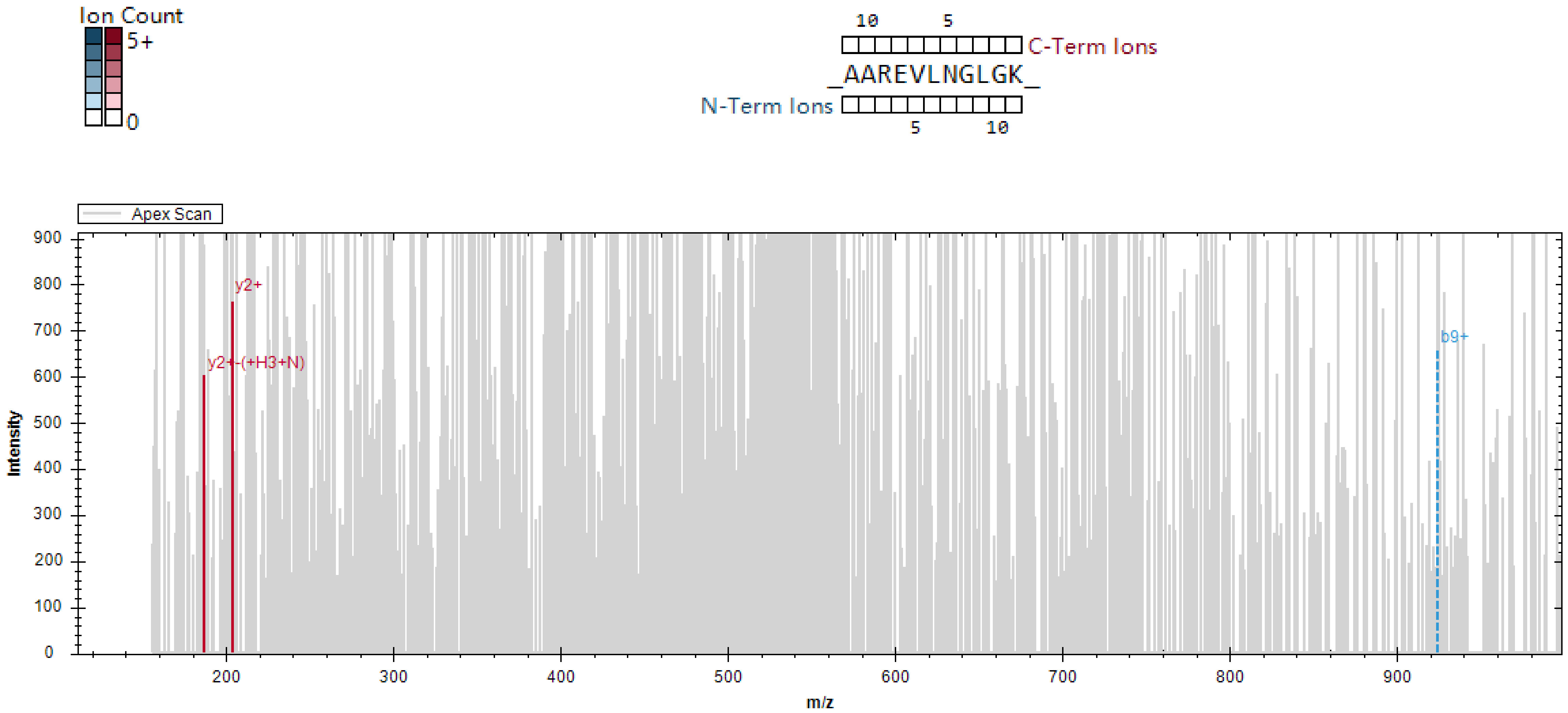

Ion Count
5+
0
10
5
C-Term Ions
_AAREVLNGLGK_
N-Term Ions
5
10
Apex Scan
Intensity
m/z
y2+
y2+-(+H3+N)
b9+

P21462

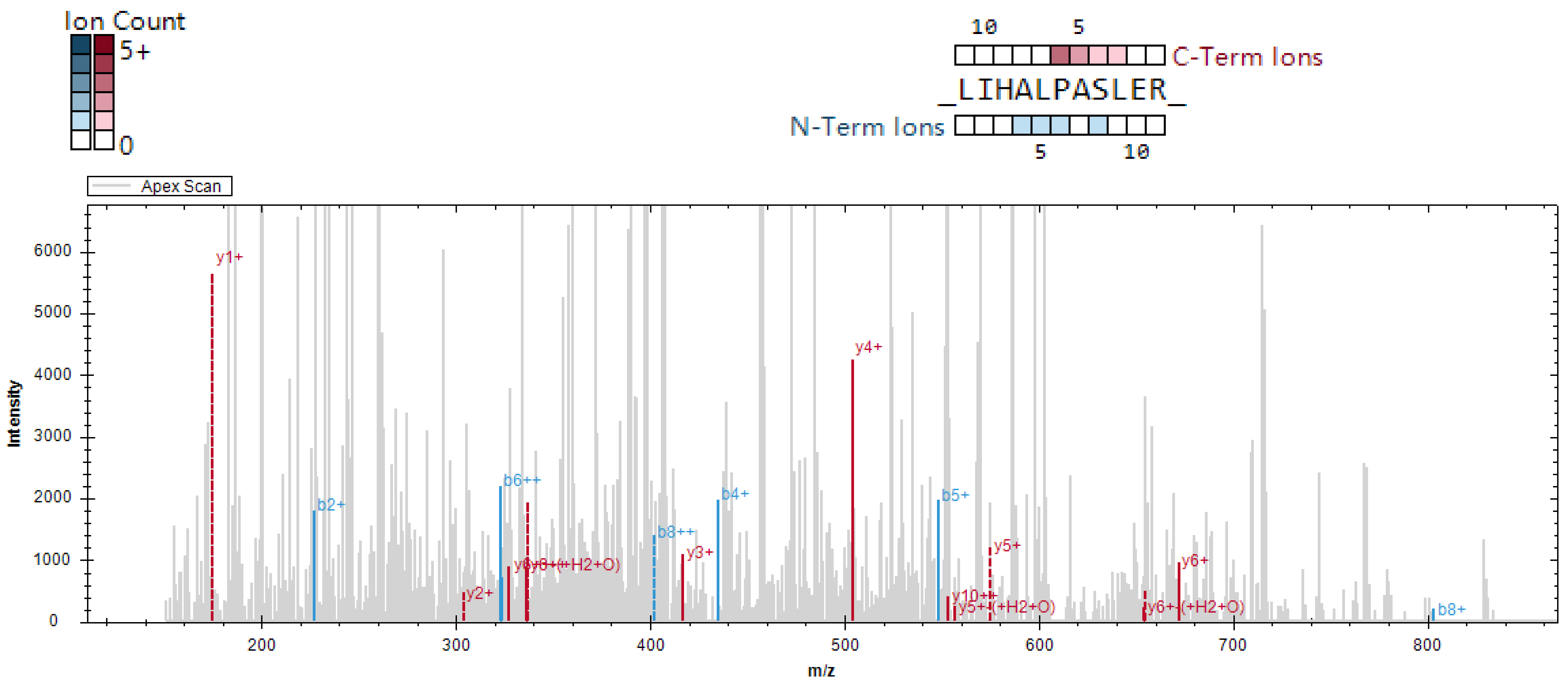

Ion Count
5+
0
10
5
C-Term Ions
_LIHALPASLER_
N-Term Ions
5
10
Apex Scan
Intensity
m/z
y1+
y4+
b6++
b2+
b4+
b5+
b8++
y3+
y5+
y6+
y2+
y10++
b8+

P22692

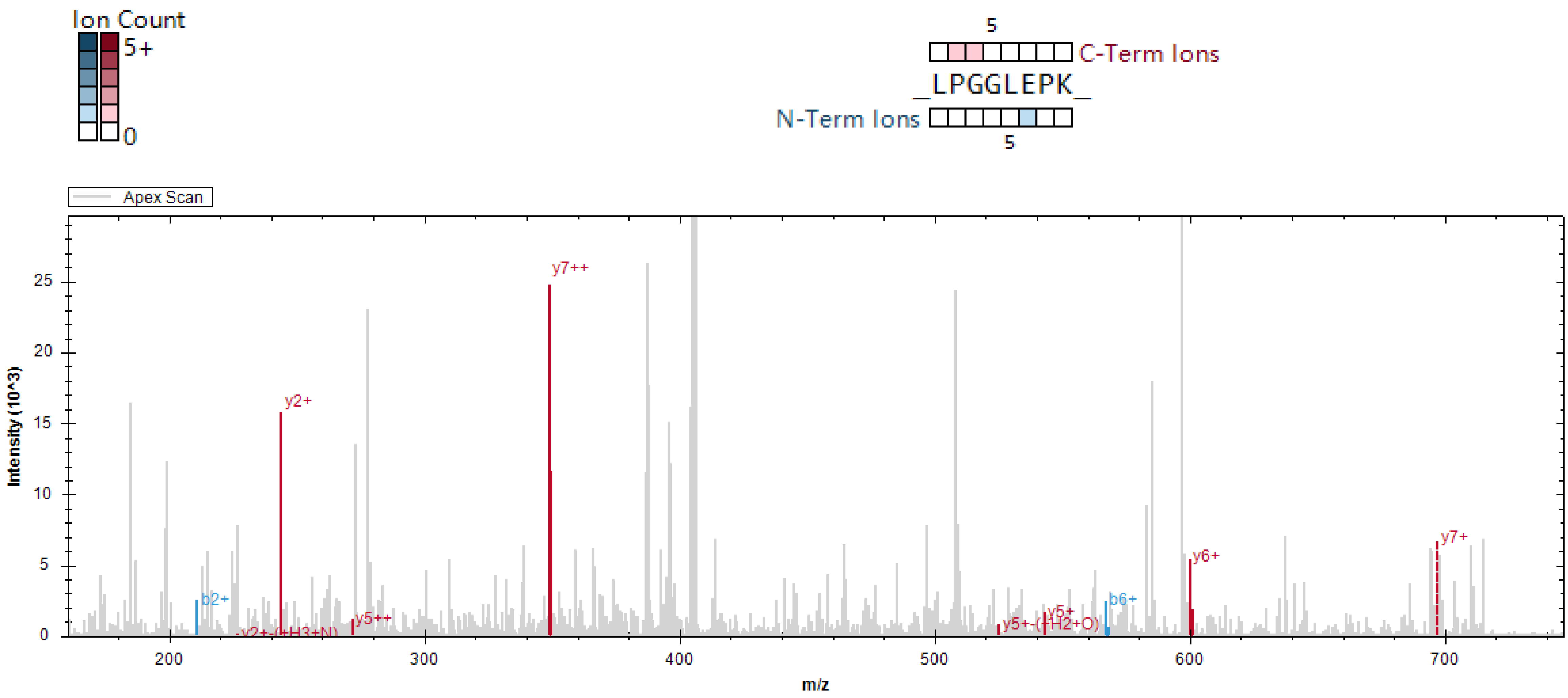

Ion Count
5+
0
5
C-Term Ions
_LPGGLEPK_
N-Term Ions
5
Apex Scan
y7++
y2+
b2+
y2+-(H3+N)
y5++
y5+-(H2+O)
y5+
b6+
y6+
y7+
Intensity (10^3)
m/z
0
5
10
15
20
25
200
300
400
500
600
700

P23435

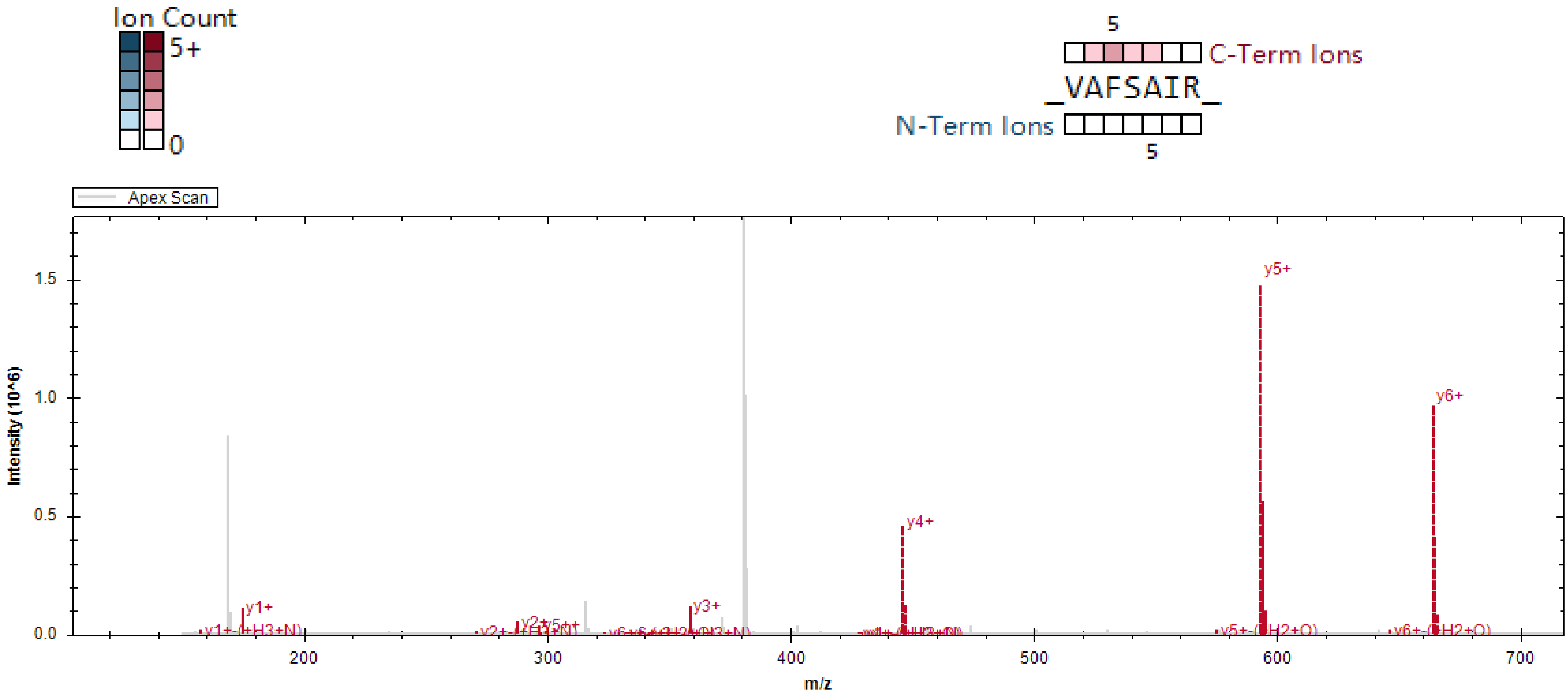

Ion Count
5+
0
5
C-Term Ions
_VAFSAIR_
N-Term Ions
5
Apex Scan
Intensity (10^6)
m/z
y5+
y6+
y4+
y3+
y1+

P24071

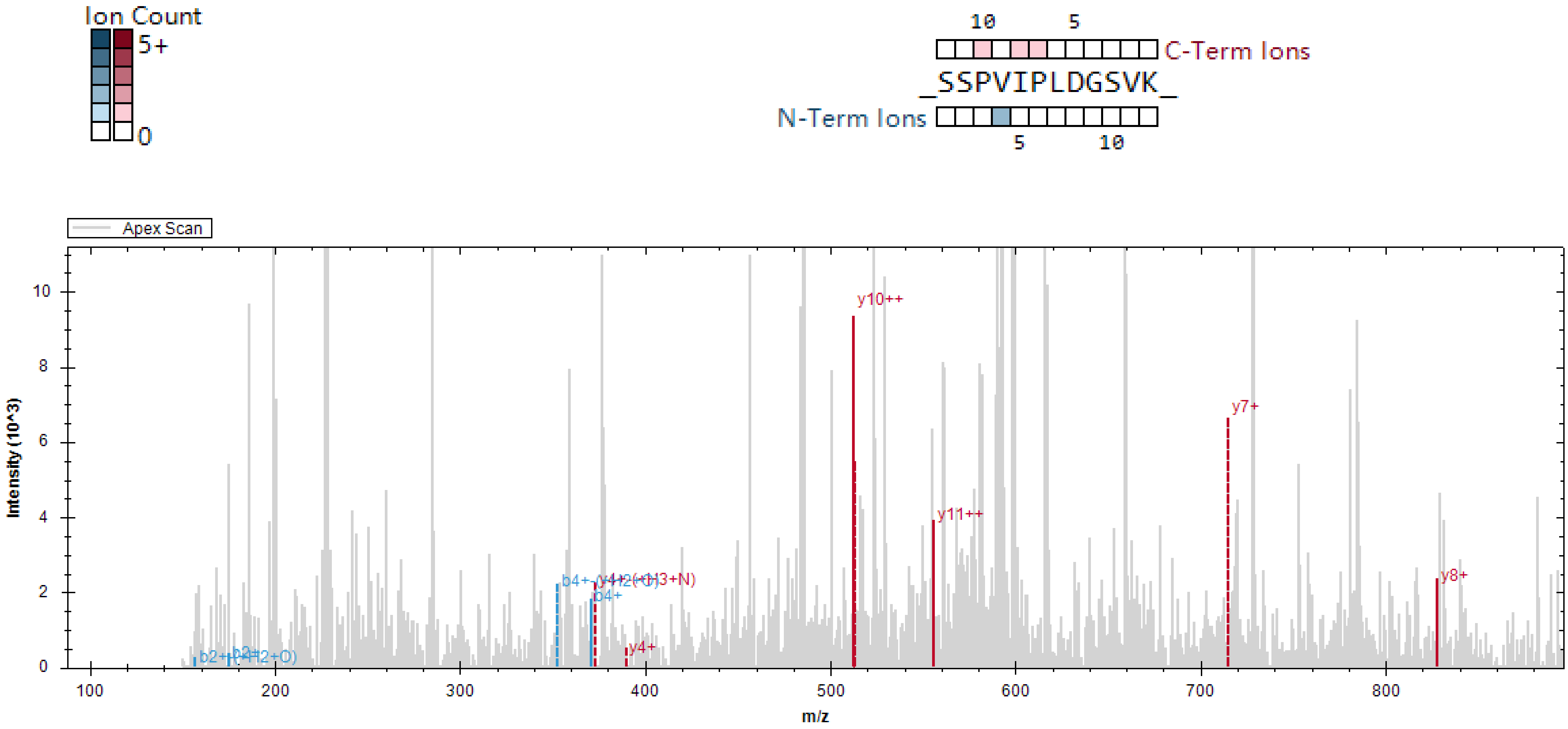
Ion Count
5+
0
10
5
C-Term Ions
_SSPVIPLDGSVK_
N-Term Ions
5
10
Apex Scan
y10++
y7+
y11++
b4+
y8+
b4+
y4+
b2+
Intensity (10^3)
m/z
0
2
4
6
8
10
100
200
300
400
500
600
700
800

P24385

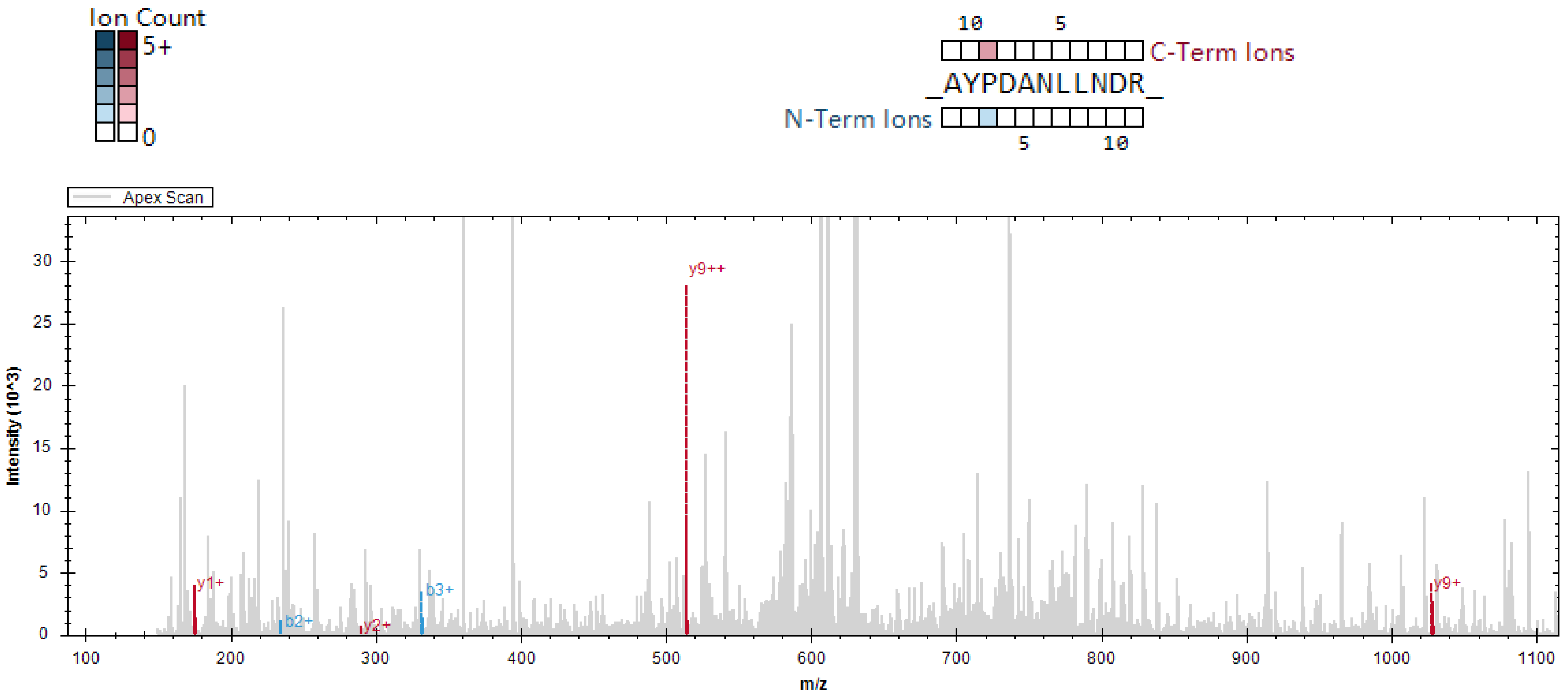

Ion Count
5+
0
10
5
C-Term Ions
_AYPDANLLNDR_
N-Term Ions
5
10
Apex Scan
y9++
y1+
b2+
y2+
b3+
y9+
Intensity (10^3)
m/z

P24592

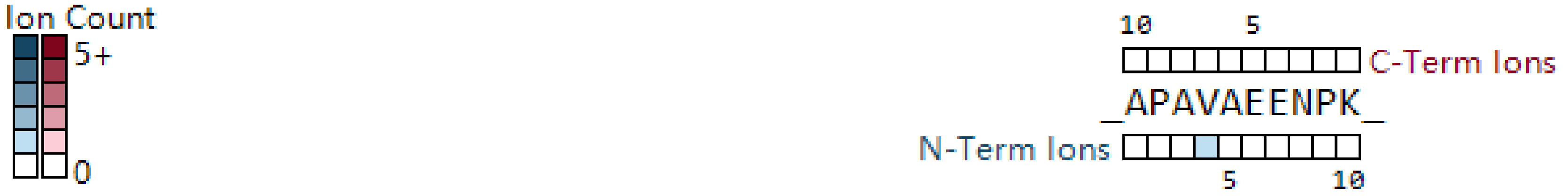

Ion Count
5+
0
10
5
C-Term Ions
_APAVAEENPK_
N-Term Ions
5
10


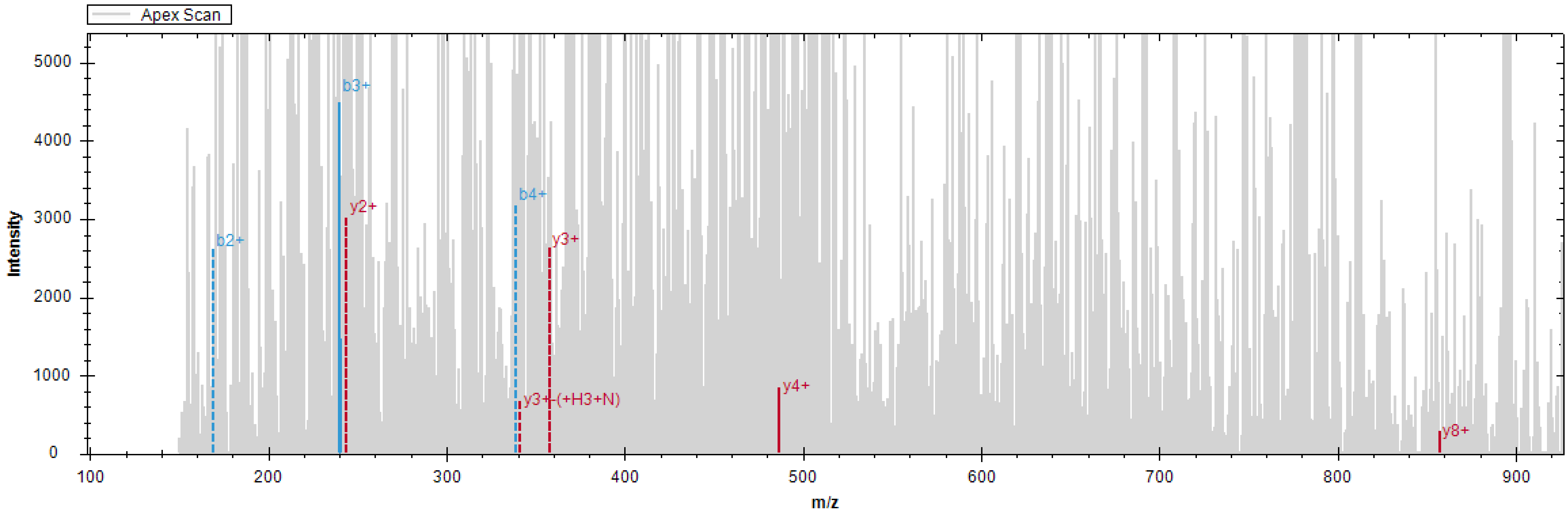

Apex Scan
Intensity
5000
4000
3000
2000
1000
0
b3+
b4+
y2+
b2+
y3+
y3+-(+H3+N)
y4+
y8+
100
200
300
400
500
600
700
800
900
m/z

P26006

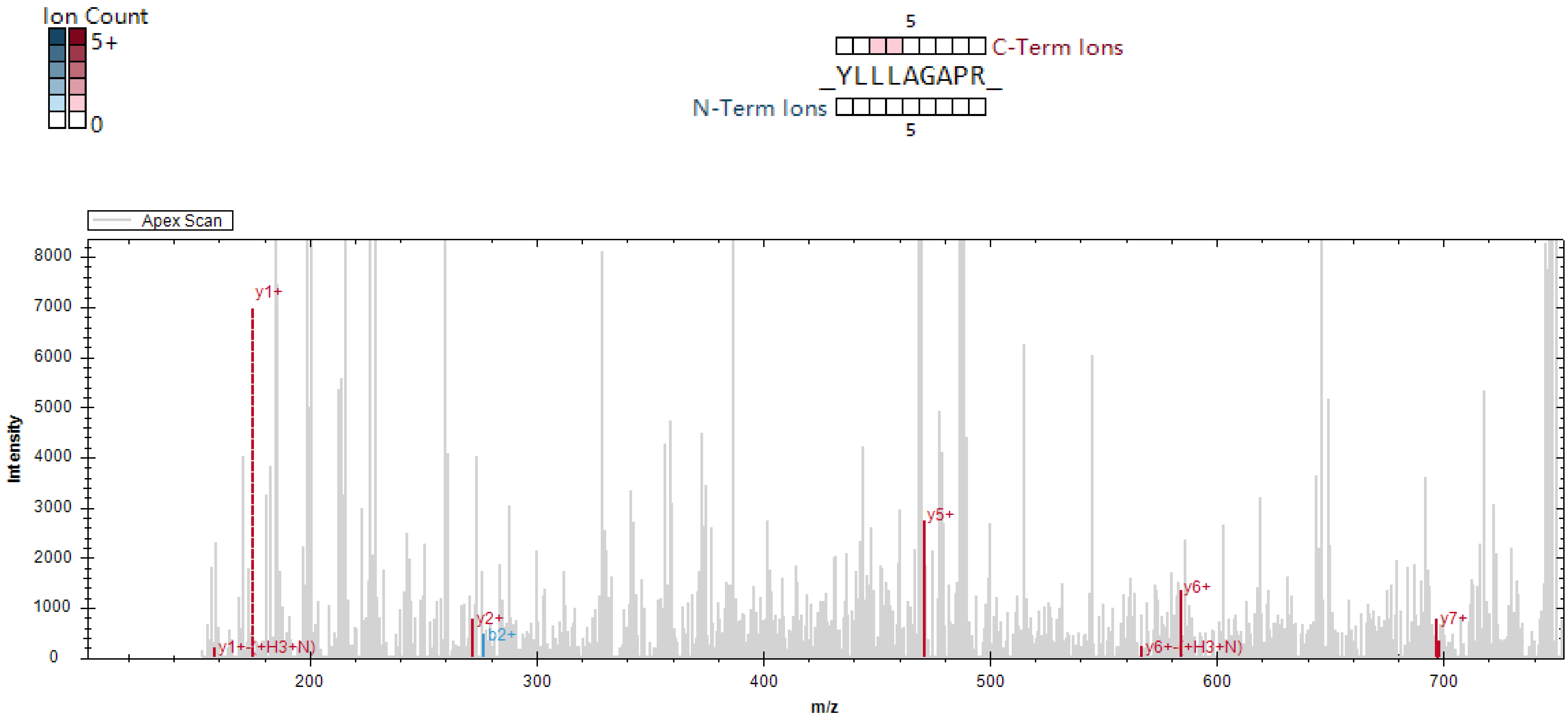

Ion Count
5+
0
5
C-Term Ions
_YLLLAGAPR_
N-Term Ions
5
Apex Scan
Intensity
m/z
y1+
y1+-(+H3+N)
y2+
b2+
y5+
y6+
y6+-(+H3+N)
y7+

P29317

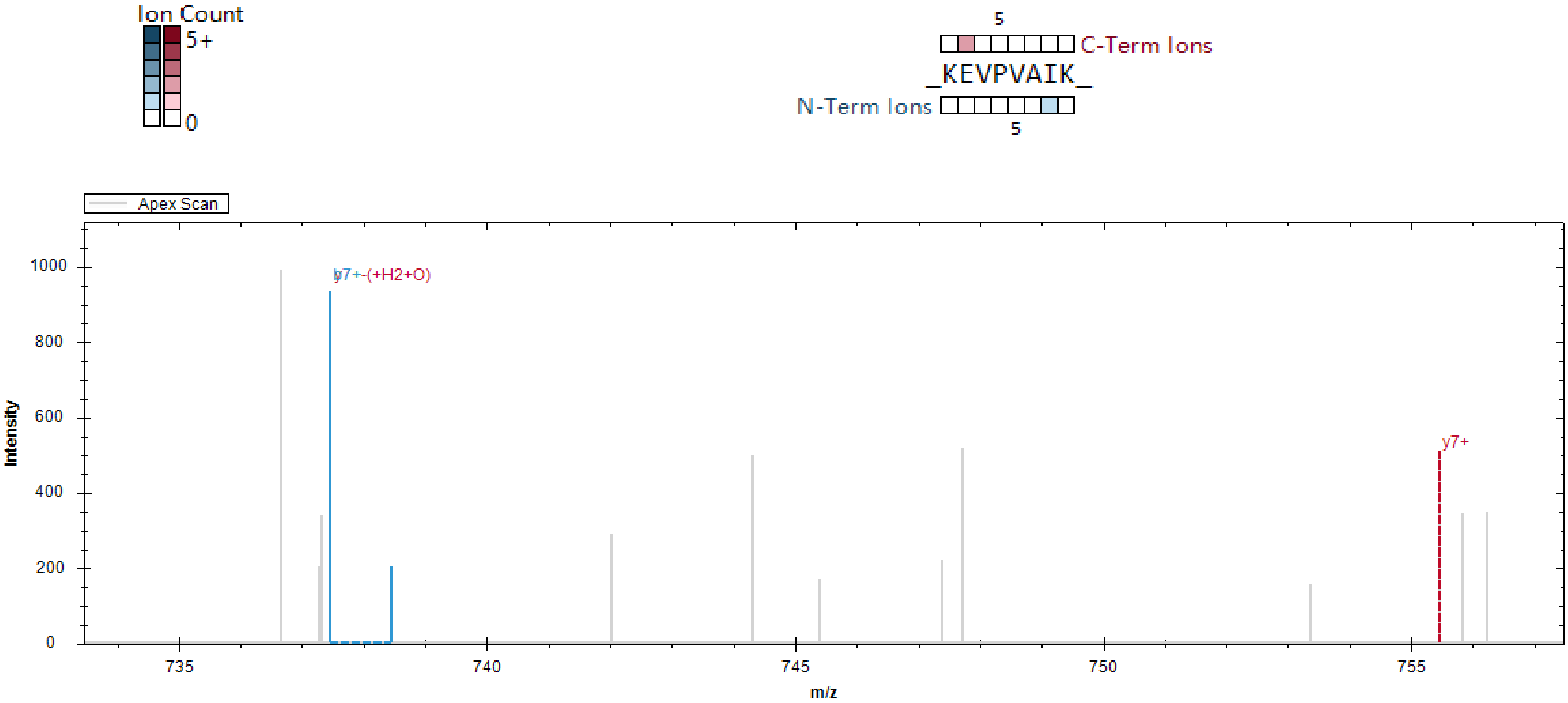

Ion Count
5+
0
5
C-Term Ions
_KEVPVAIK_
N-Term Ions
5
Apex Scan
b7+-(+H2+O)
y7+
Intensity
m/z

P30046

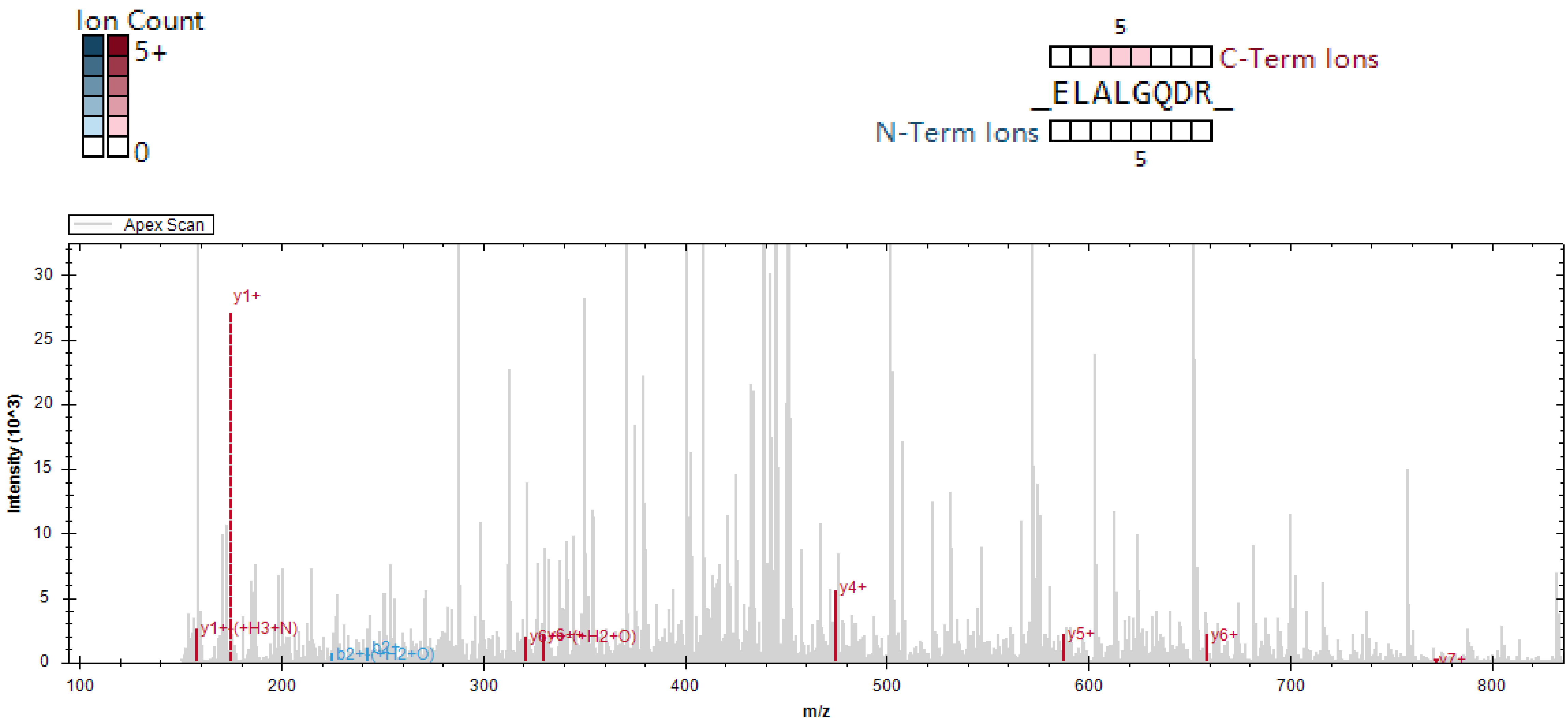

Ion Count
5+
0
5
C-Term Ions
_ELALGQDR_
N-Term Ions
5
Apex Scan
Intensity (10^3)
m/z
y1+
y4+
y5+
y6+

P31483

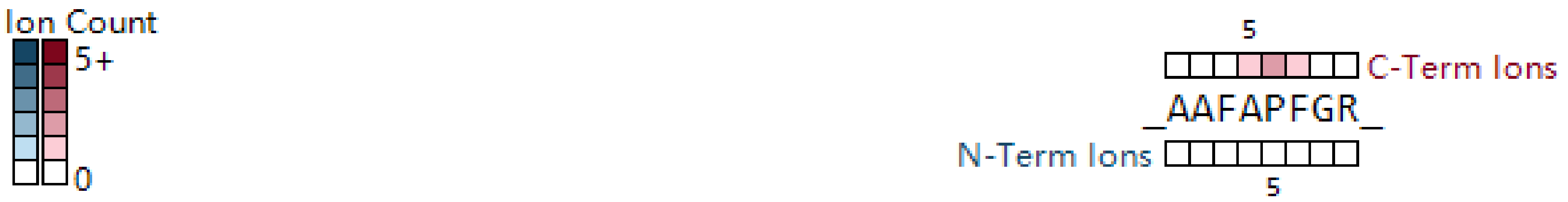

Ion Count
5+
0
5
C-Term Ions
_AAFAPFGR_
N-Term Ions
5


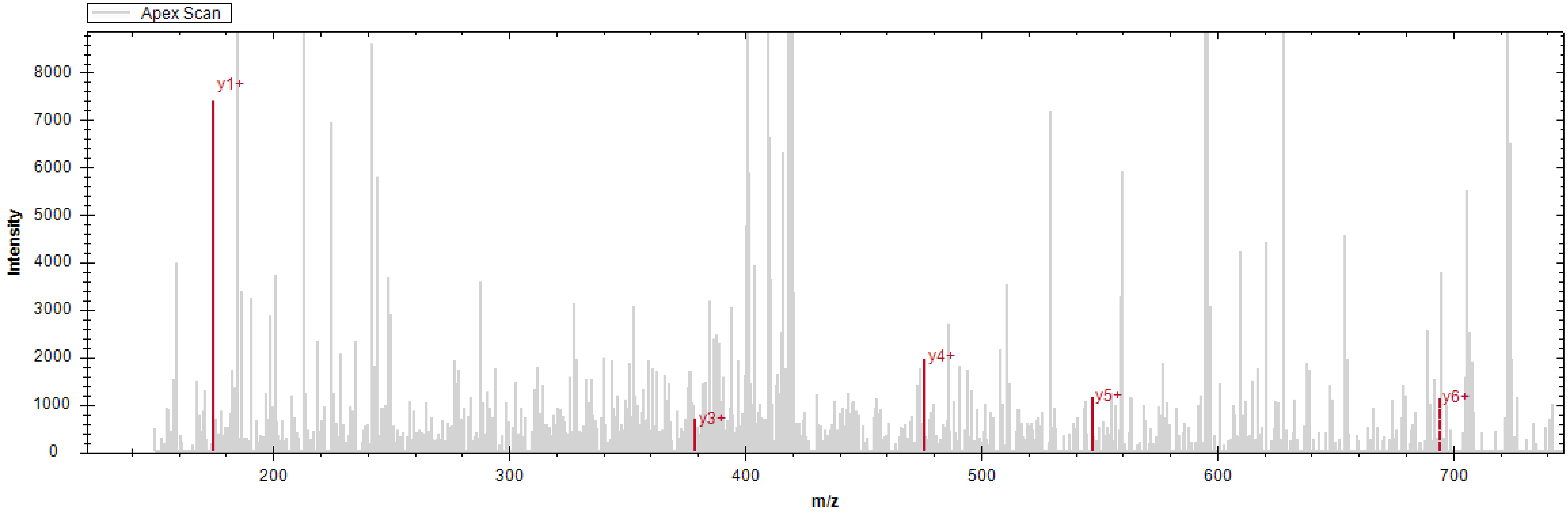

Apex Scan
Intensity
m/z
y1+
y3+
y4+
y5+
y6+

P31997

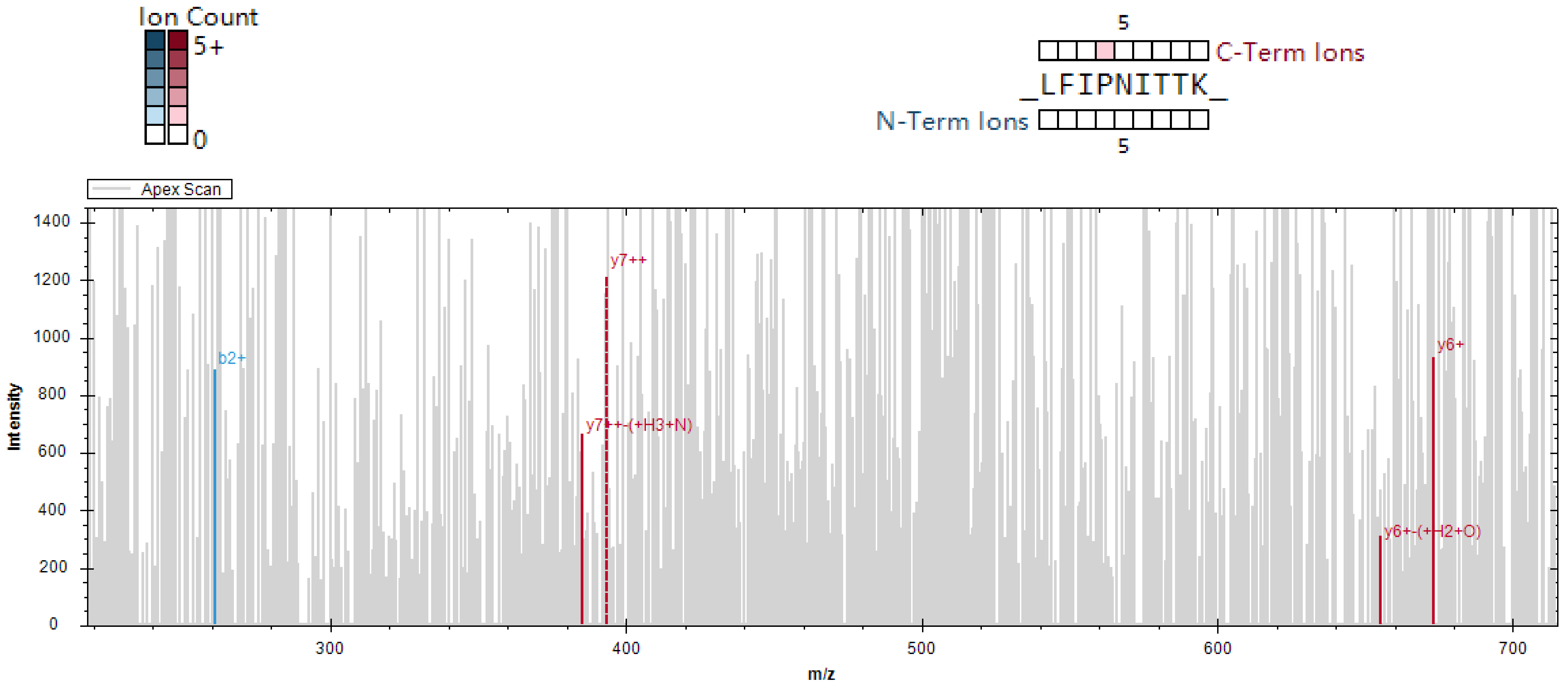

Ion Count
5+
0
5
C-Term Ions
_LFIPNITTK_
N-Term Ions
5
Apex Scan
Intensity
m/z
b2+
y7++
y7++-(+H3+N)
y6+
y6+-(+H2+O)

# P32189

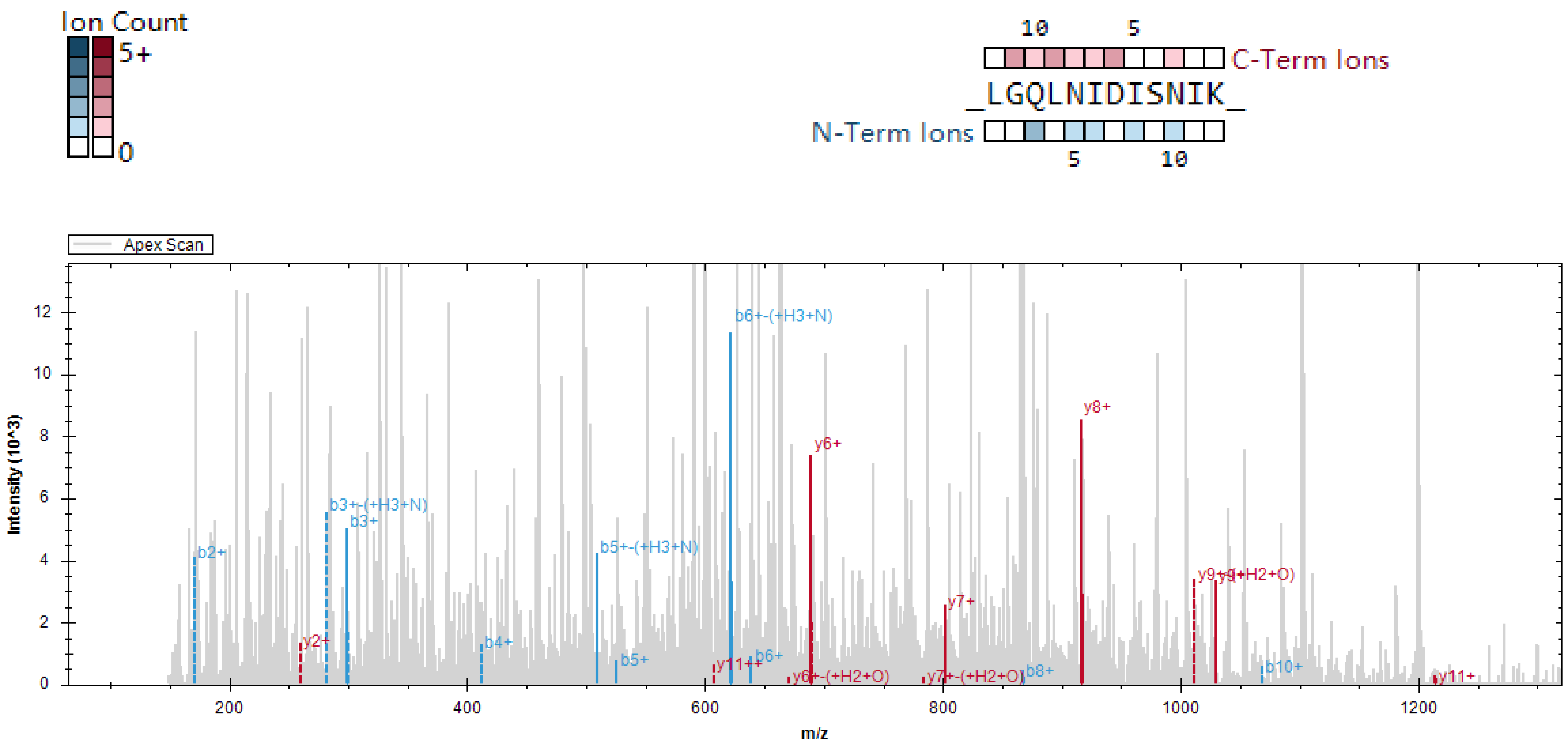

Ion Count
5+
0
10
5
C-Term Ions
_LGQLNIDISNIK_
N-Term Ions
5
10
Apex Scan
Intensity (10^3)
m/z
b2+
b3+-(+H3+N)
b3+
b4+
b5+-(+H3+N)
b5+
b6+-(+H3+N)
b6+
b8+
b10+
y2+
y6+
y6+-(+H2+O)
y7+
y7+-(+H2+O)
y8+
y9+
y11++
y11+

P32246

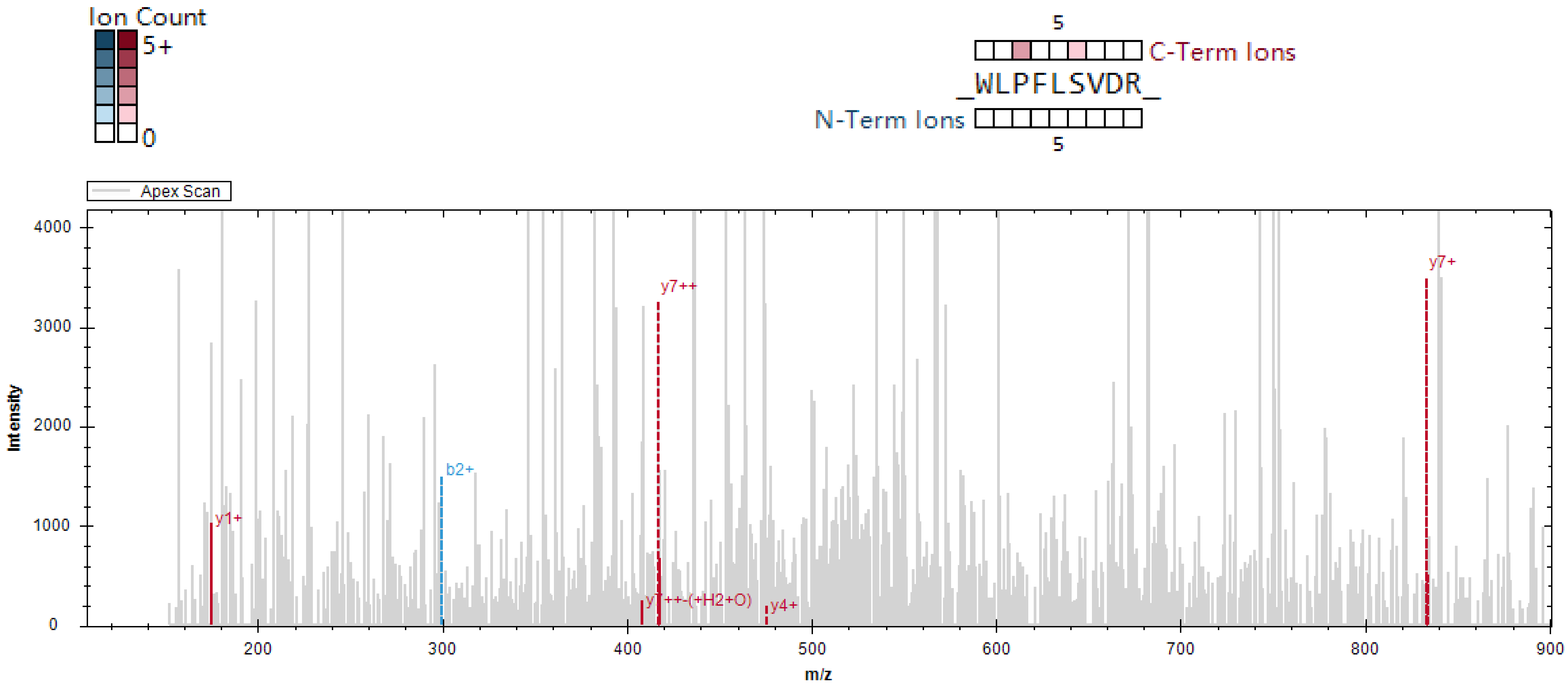

Ion Count
5+
0
5
C-Term Ions
_WLPFLSVDR_
N-Term Ions
5
Apex Scan
y7+
y7++
b2+
y1+
y7++-(+H2+O)
y4+
Intensity
m/z
4000
3000
2000
1000
0
200
300
400
500
600
700
800
900

P34810

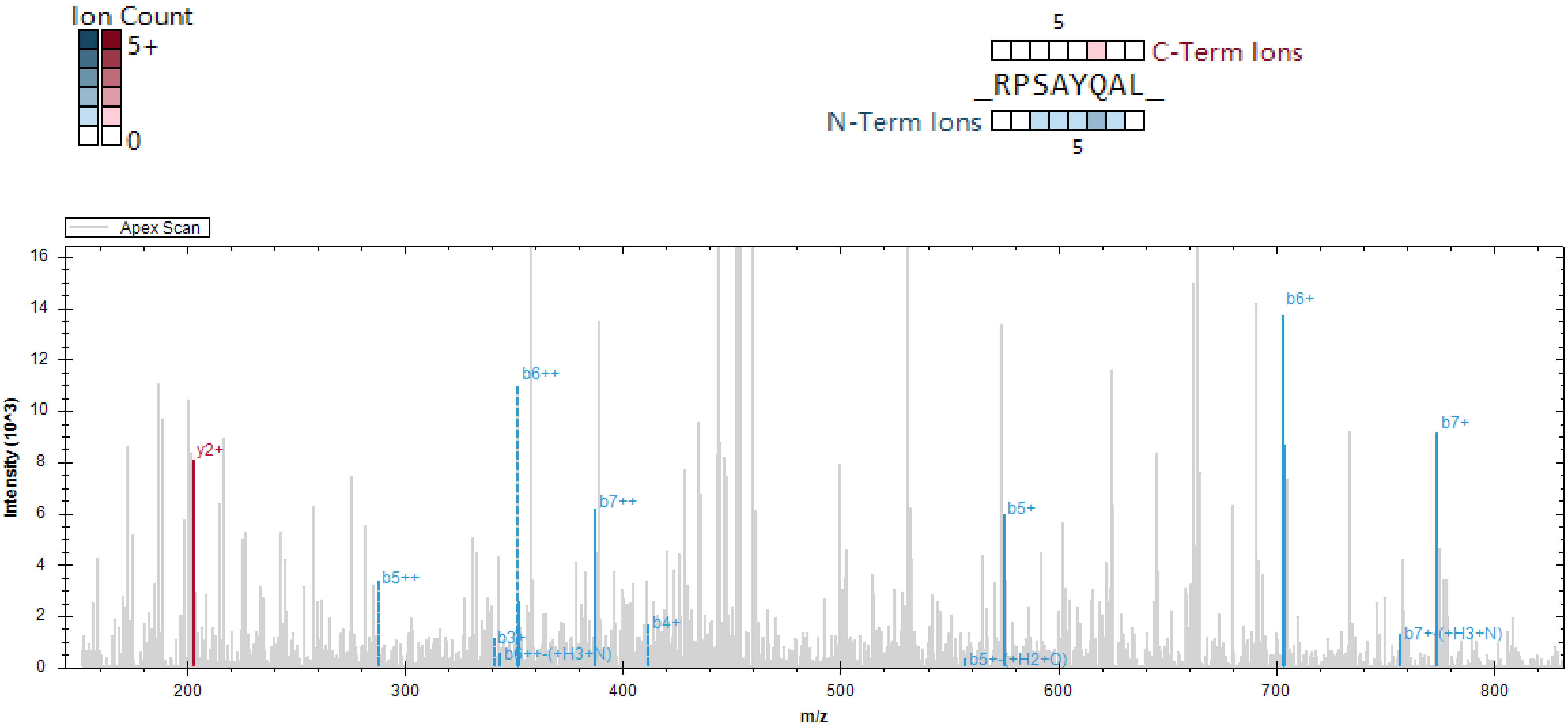

Ion Count
5+
0
5
C-Term Ions
_RPSAYQAL_
N-Term Ions
5
Apex Scan
y2+
b5++
b3+
b6++
b7++
b4+
b5+
b6+
b7+
b7+-(+H3+N)
b5+-(+H2+O)
Intensity (10^3)
m/z
0
2
4
6
8
10
12
14
16
200
300
400
500
600
700
800

P35240

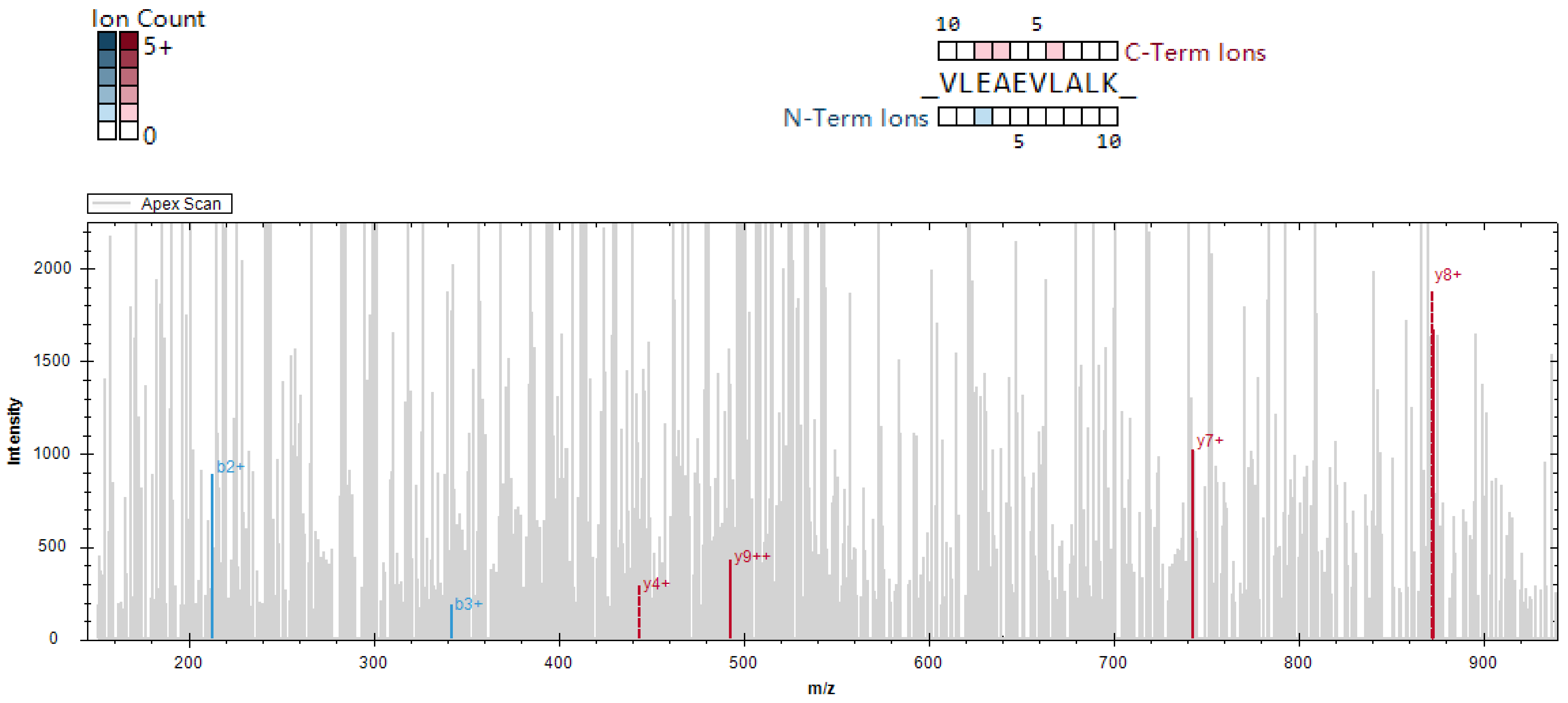

Ion Count
5+
0
10
5
C-Term Ions
_VLEAEVLALK_
N-Term Ions
5
10
Apex Scan
y8+
y7+
b2+
y9++
y4+
b3+
Intensity
m/z

P35670

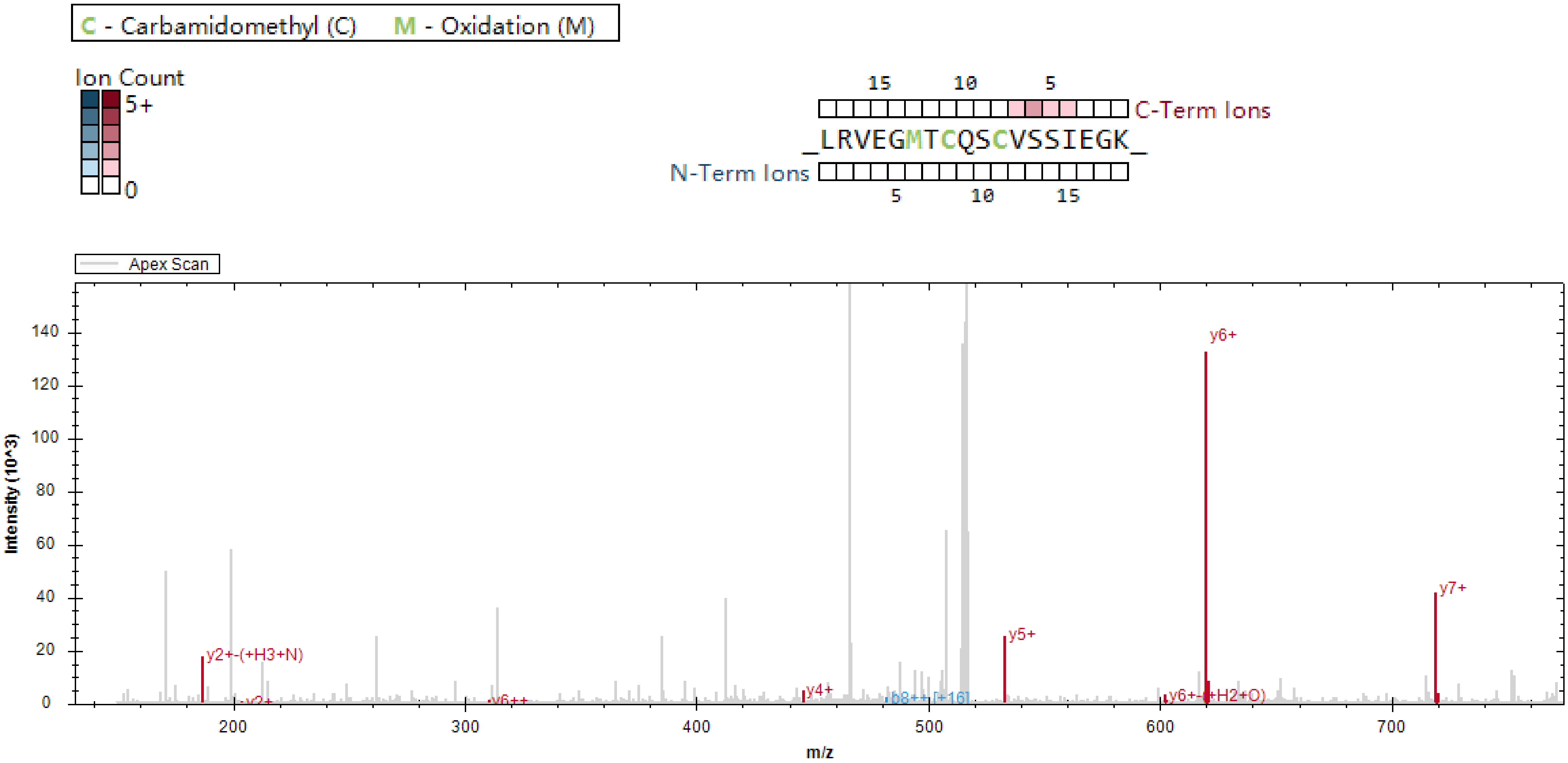

C - Carbamidomethyl (C)
M - Oxidation (M)
Ion Count
5+
0
15 10 5
C-Term Ions
_LRVEGMTCQSCVSSIEGK_
N-Term Ions
5 10 15
Apex Scan
Intensity (10^3)
m/z
y6+
y7+
y5+
y2+-(+H3+N)
y2+
y6++
y4+
b8++[+16]
y6+-(+H2+O)

# P35900

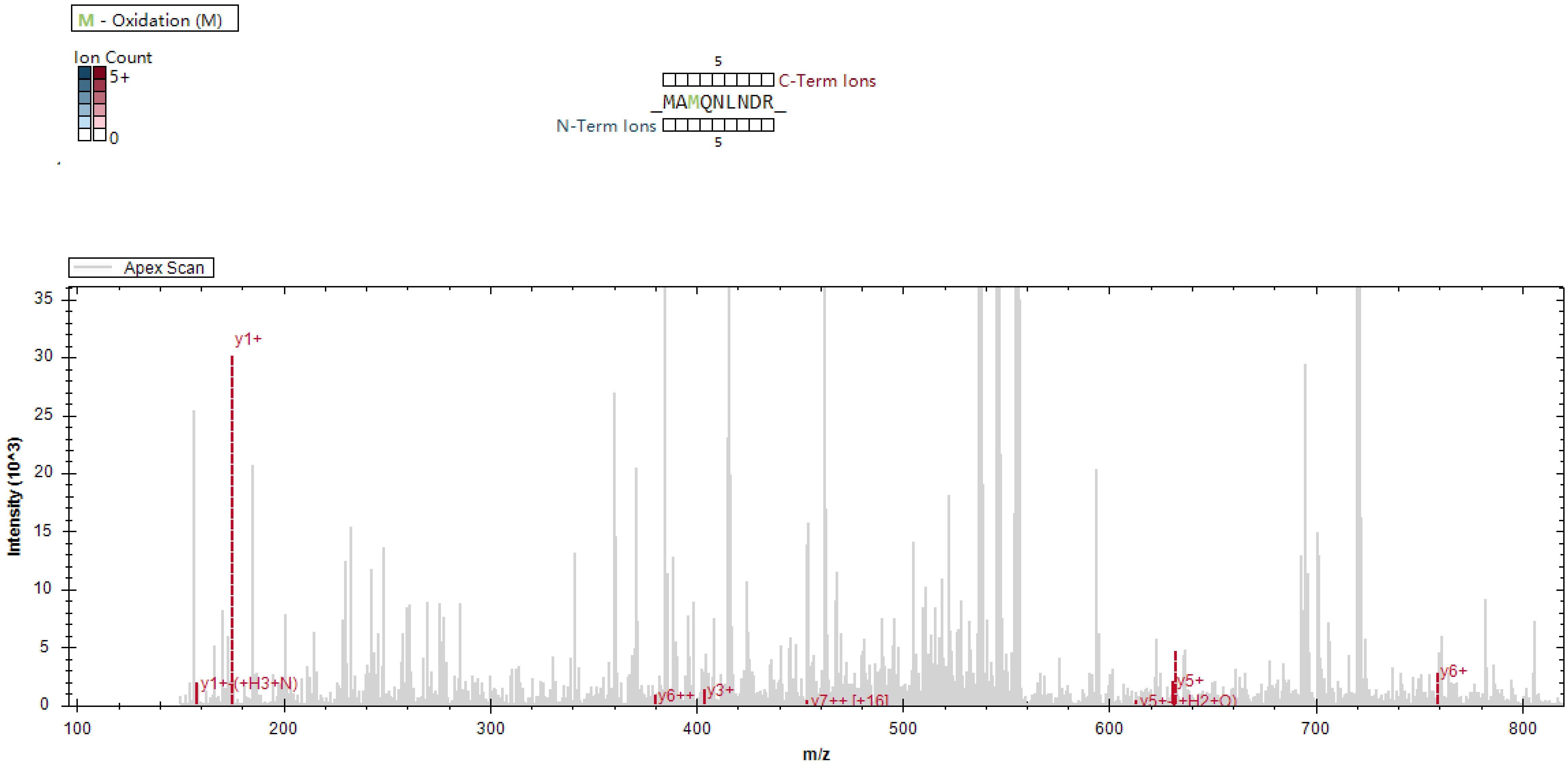
M - Oxidation (M)
Ion Count
5+
0
5
C-Term Ions
_MAMQNLNDR_
N-Term Ions
5
Apex Scan
y1+
y1+ (+H3+N)
y6++
y3+
y7++ [+16]
y5++ (+H2+O)
y5+
y6+
Intensity (10^3)
m/z
0
5
10
15
20
25
30
35
100
200
300
400
500
600
700
800

P39687

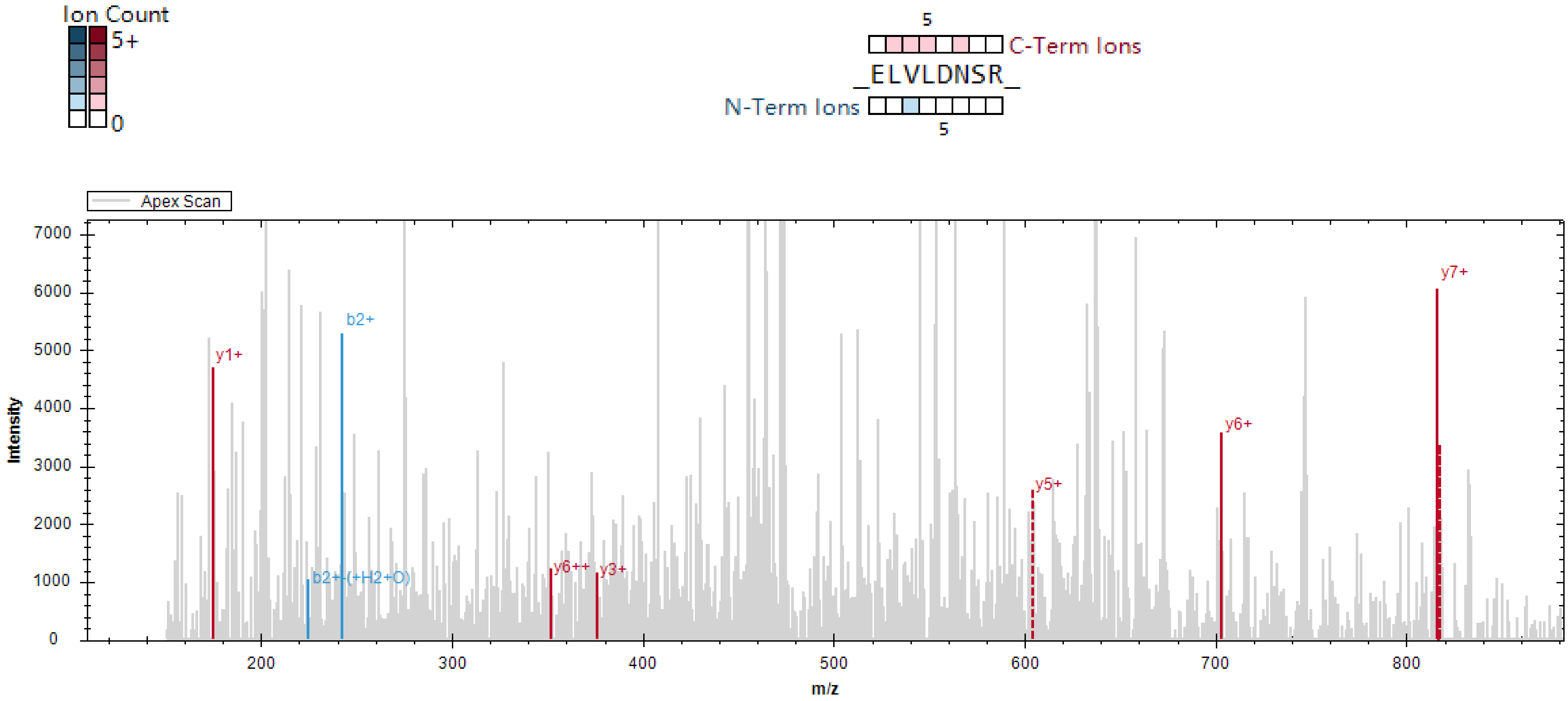
Ion Count
5+
0
5
C-Term Ions
_ELVLDNSR_
N-Term Ions
5
Apex Scan
Intensity
m/z
y1+
b2+
b2+-(+H2+O)
y6++
y3+
y5+
y6+
y7+

P41236

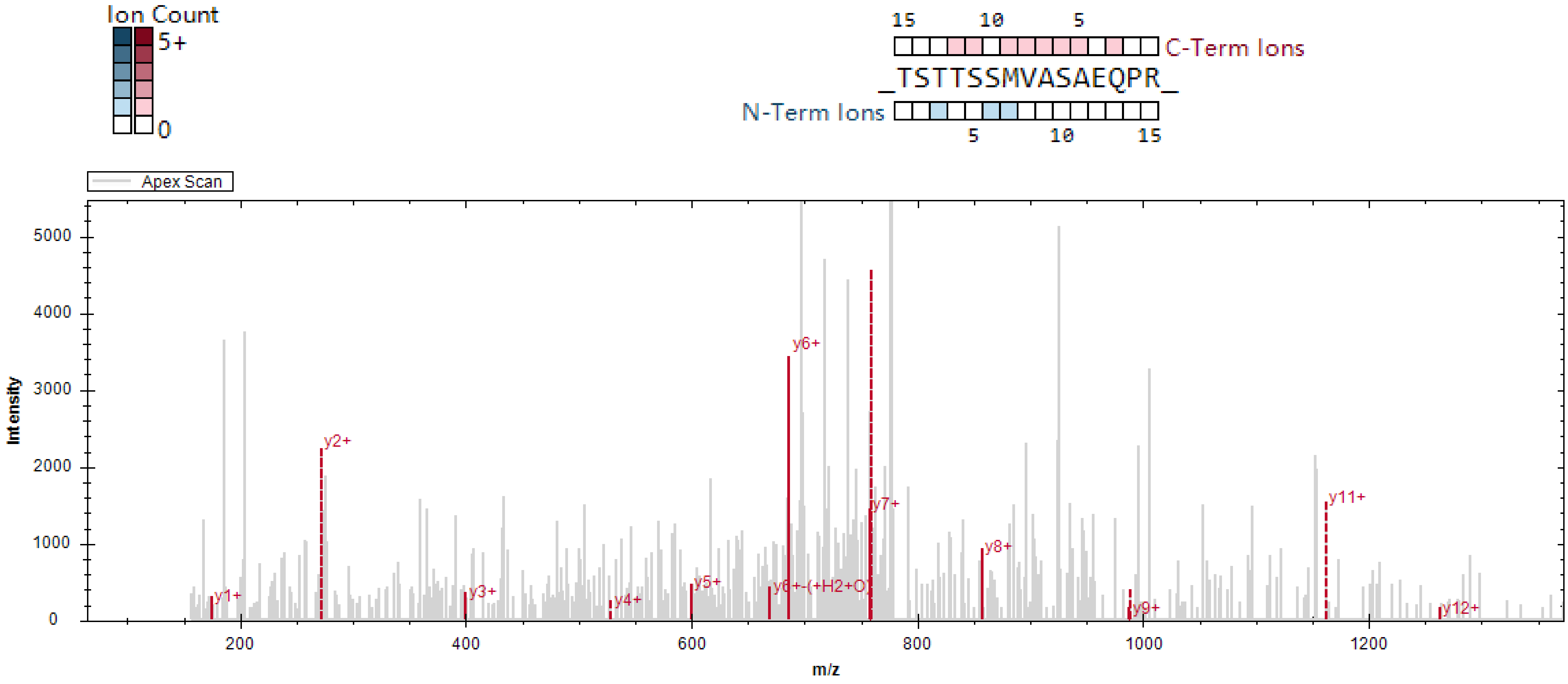
Ion Count
5+
0
15 10 5
C-Term Ions
_TSTTSSMVASAEQPR_
N-Term Ions
5 10 15
Apex Scan
5000
4000
3000
2000
1000
0
Intensity
200
400
600
800
1000
1200
m/z
y1+
y2+
y3+
y4+
y5+
y6+
y6+-(+H2+O)
y7+
y8+
y9+
y11+
y12+

P43366

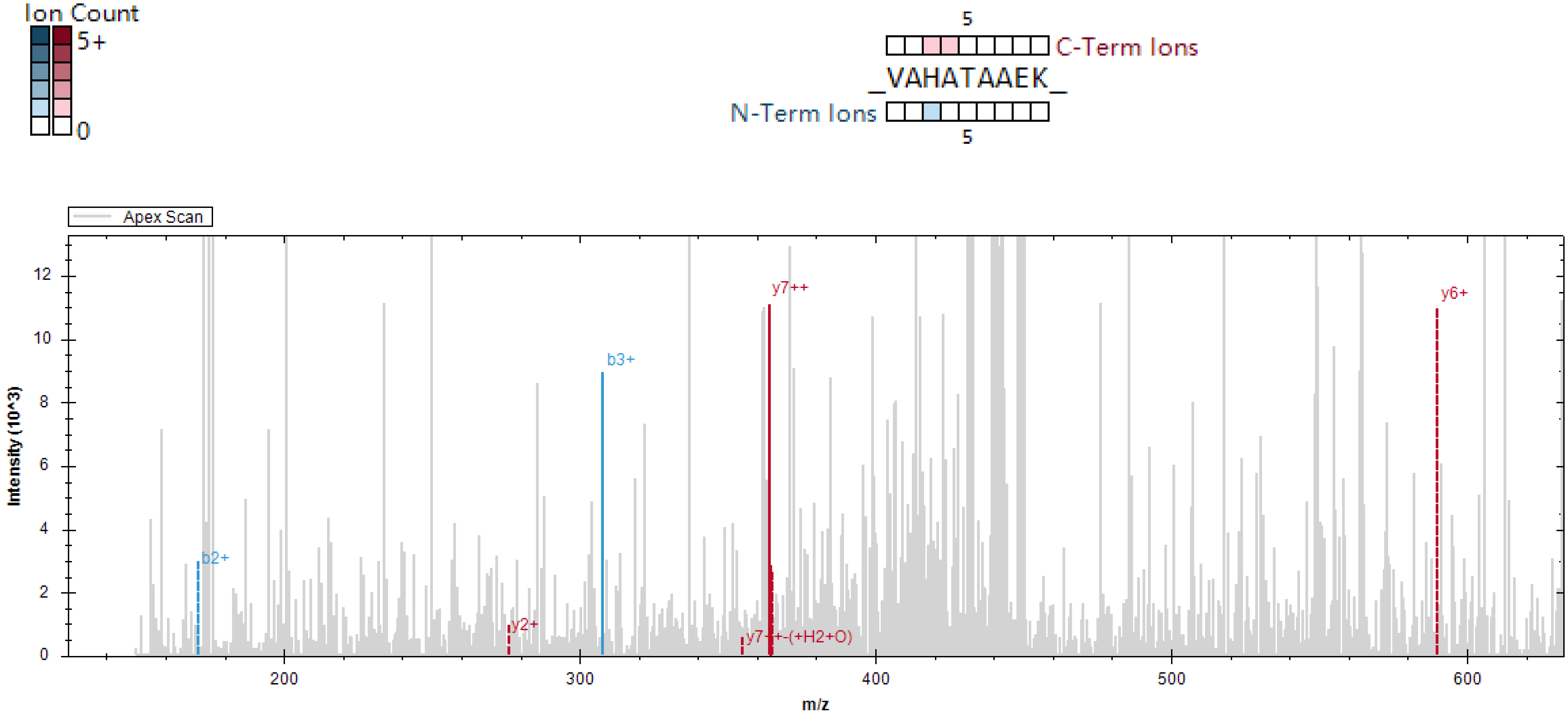

Ion Count
5+
0
5
C-Term Ions
_VAHATAAEK_
N-Term Ions
5
Apex Scan
y7++
y6+
b3+
b2+
y2+
y7++-(+H2+O)
Intensity (10^3)
m/z
0
2
4
6
8
10
12
200
300
400
500
600

P48681

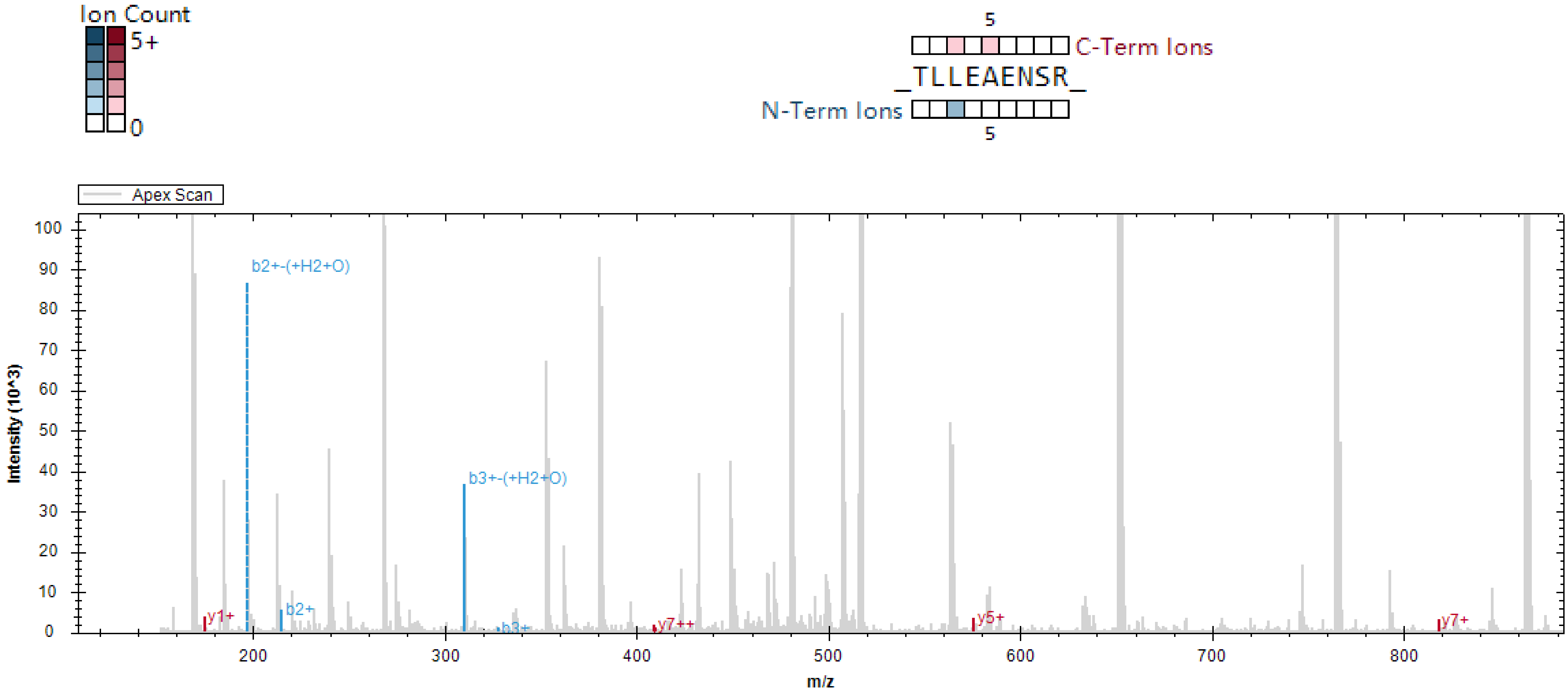

Ion Count
5+
0
5
C-Term Ions
_TLLEAENSR_
N-Term Ions
5
Apex Scan
Intensity (10^3)
m/z
b2+-(+H2+O)
b3+-(+H2+O)
b2+
b3+
y1+
y7++
y5+
y7+

P49441

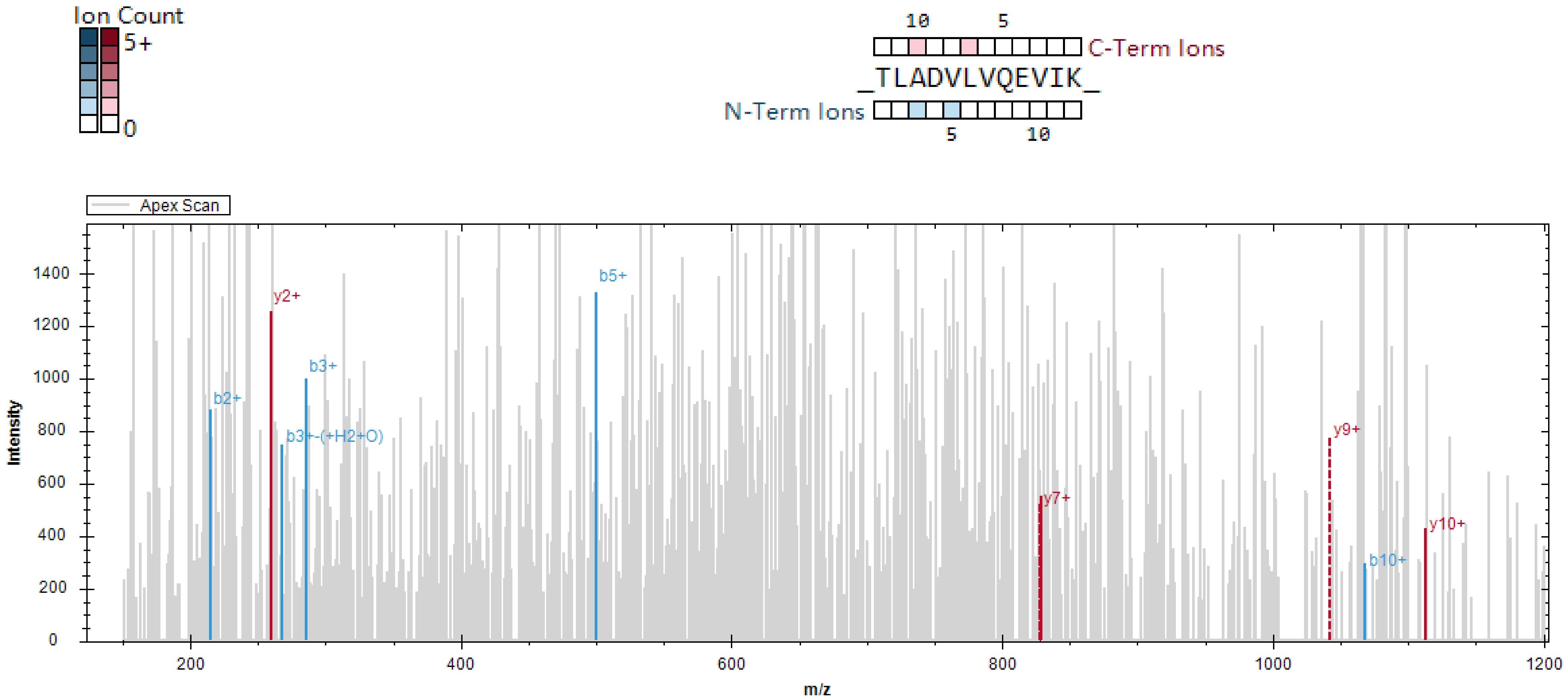
Ion Count
5+
0
10
5
C-Term Ions
_TLADVLVQEVIK_
N-Term Ions
5
10
Apex Scan
y2+
b2+
b3+
b3+-(+H2+O)
b5+
y7+
y9+
b10+
y10+
Intensity
m/z
0
200
400
600
800
1000
1200
1400
200
400
600
800
1000
1200

# P49790

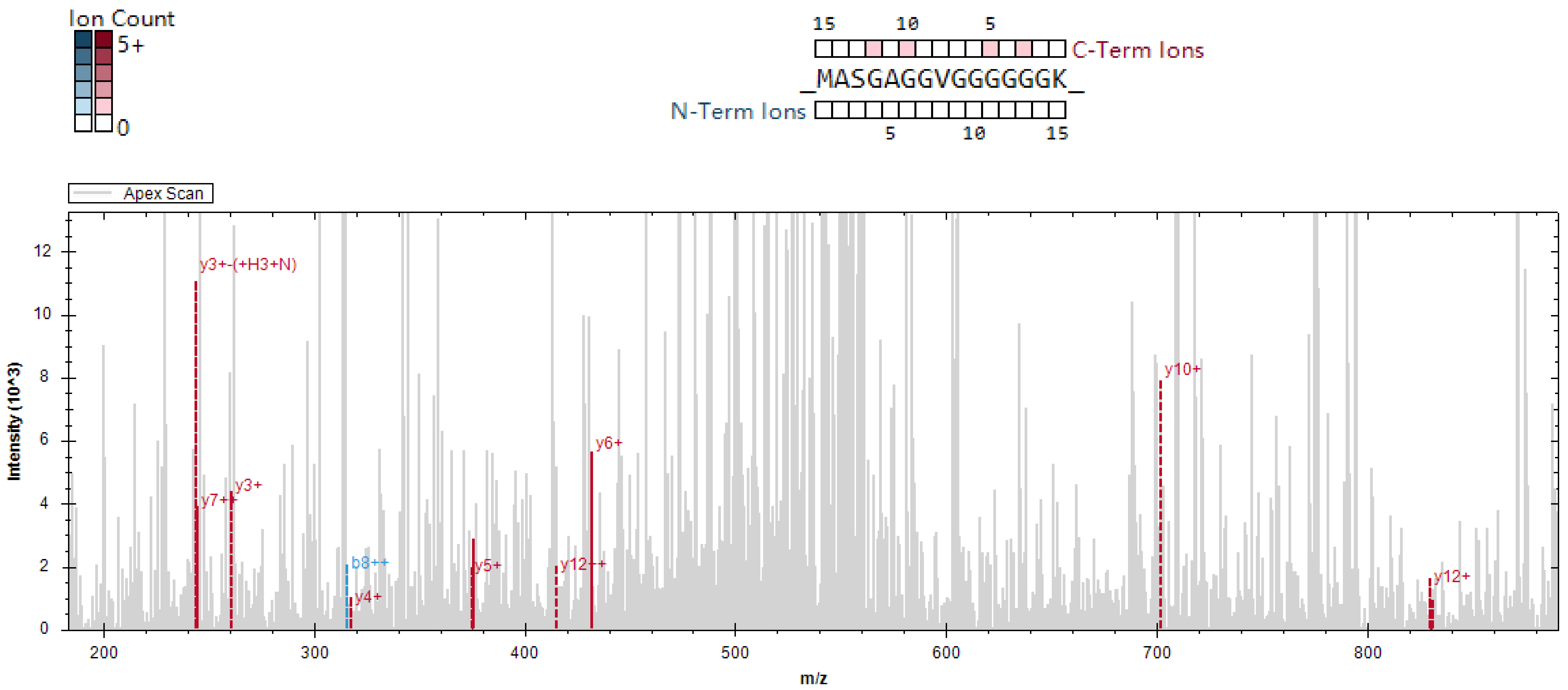

Ion Count
5+
0
15
10
5
C-Term Ions
_MASGAGGVGGGGGK_
N-Term Ions
5
10
15
Apex Scan
y3+-(+H3+N)
y7++
y3+
b8++
y4+
y5+
y12++
y6+
y10+
y12+
Intensity (10^3)
m/z

# P50238

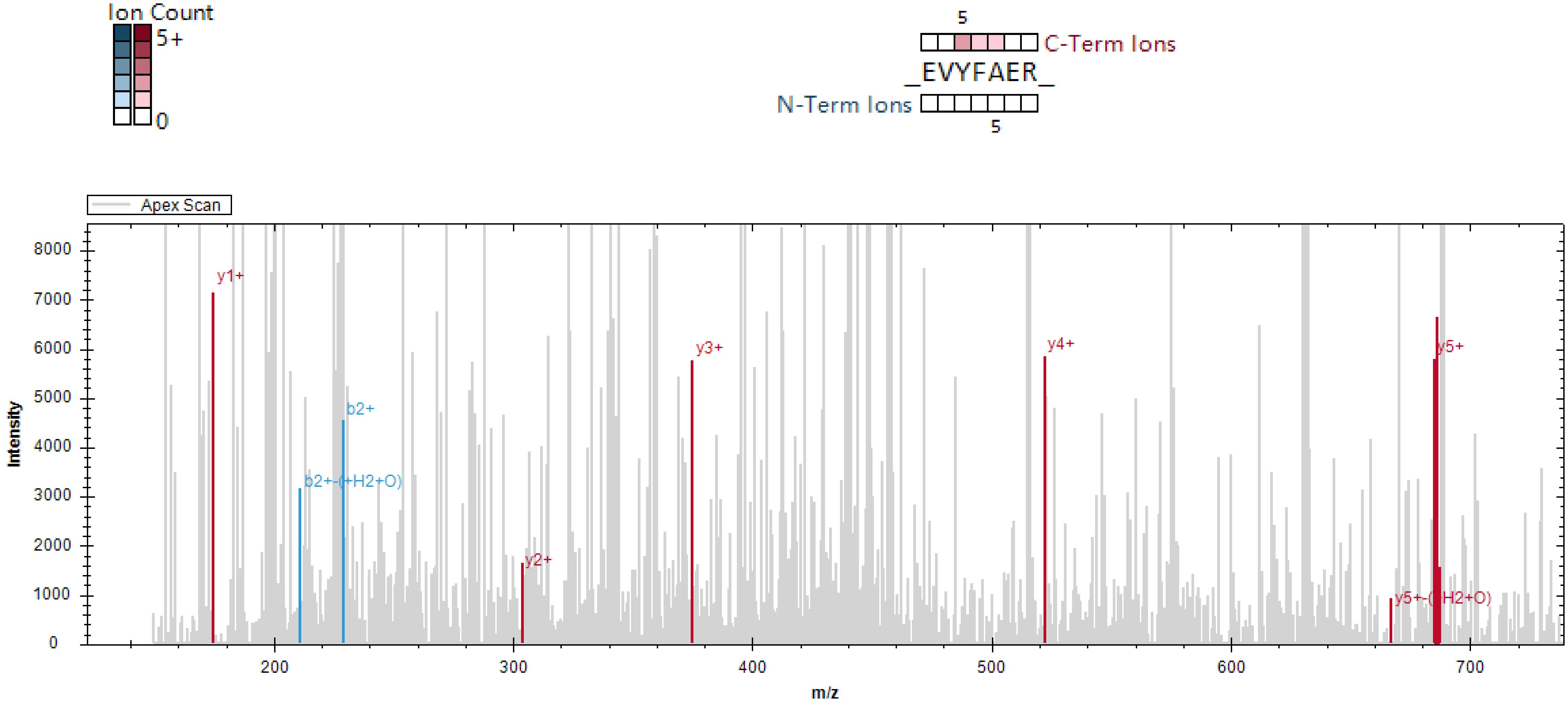

Ion Count
5+
0
5
C-Term Ions
_EVYFAER_
N-Term Ions
5
Apex Scan
y1+
b2+
b2+-(H2+O)
y2+
y3+
y4+
y5+
y5+-(H2+O)
Intensity
m/z

# P50281

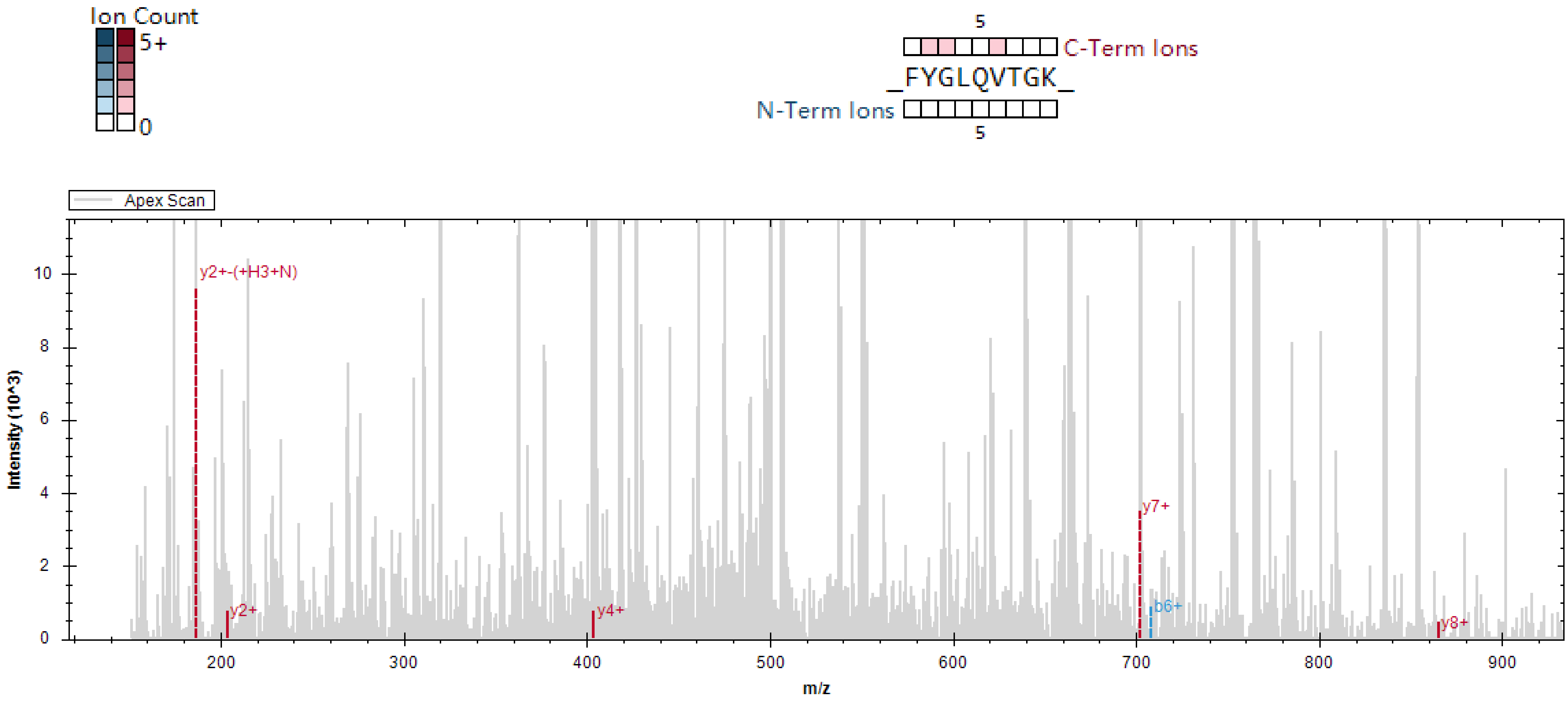

Ion Count
5+
0
5
C-Term Ions
_FYGLQVTGK_
N-Term Ions
5
Apex Scan
y2+-(+H3+N)
y2+
y4+
y7+
b6+
y8+
Intensity (10^3)
m/z
0
2
4
6
8
10
200
300
400
500
600
700
800
900

P50583

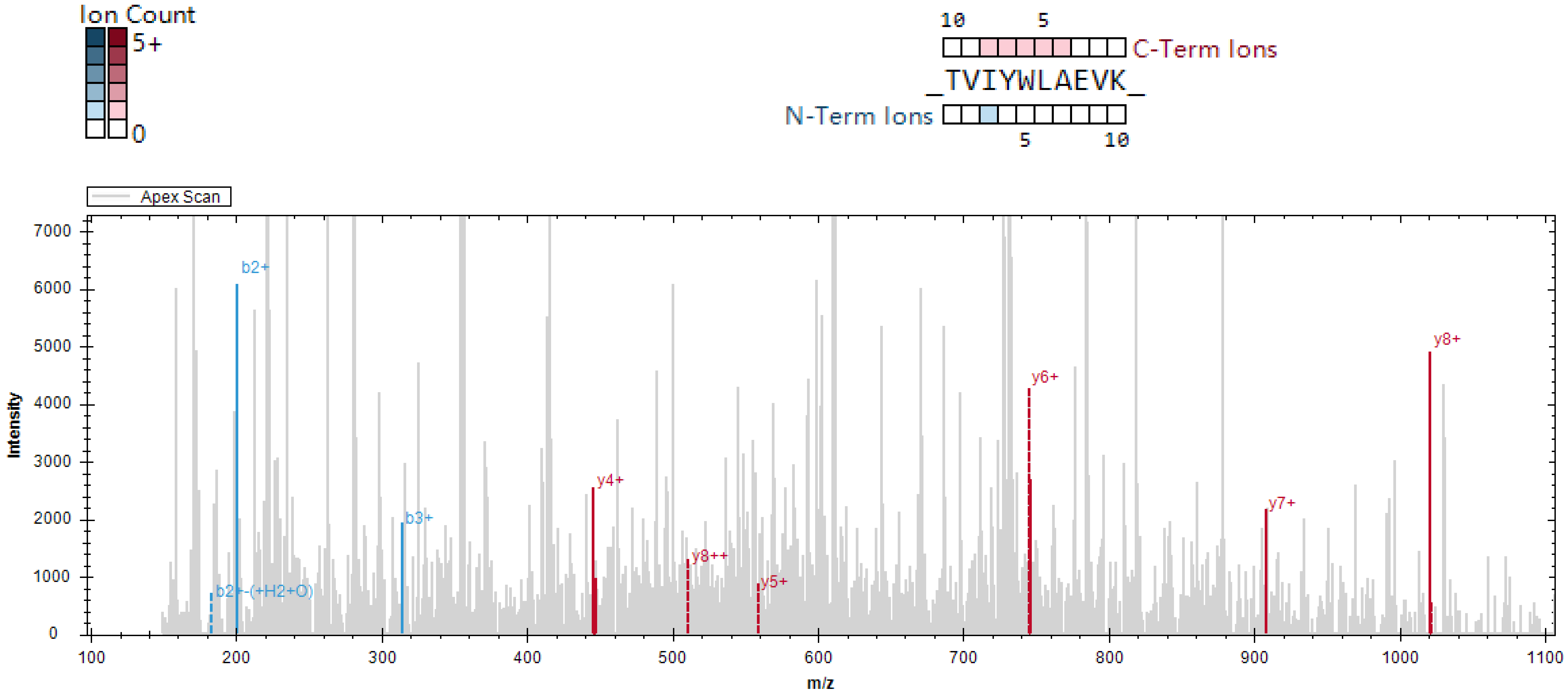

Ion Count
5+
0
10
5
C-Term Ions
_TVIYWLAEVK_
N-Term Ions
5
10
Apex Scan
b2+
b2+-(+H2+O)
b3+
y4+
y8++
y5+
y6+
y7+
y8+
Intensity
m/z

P51674

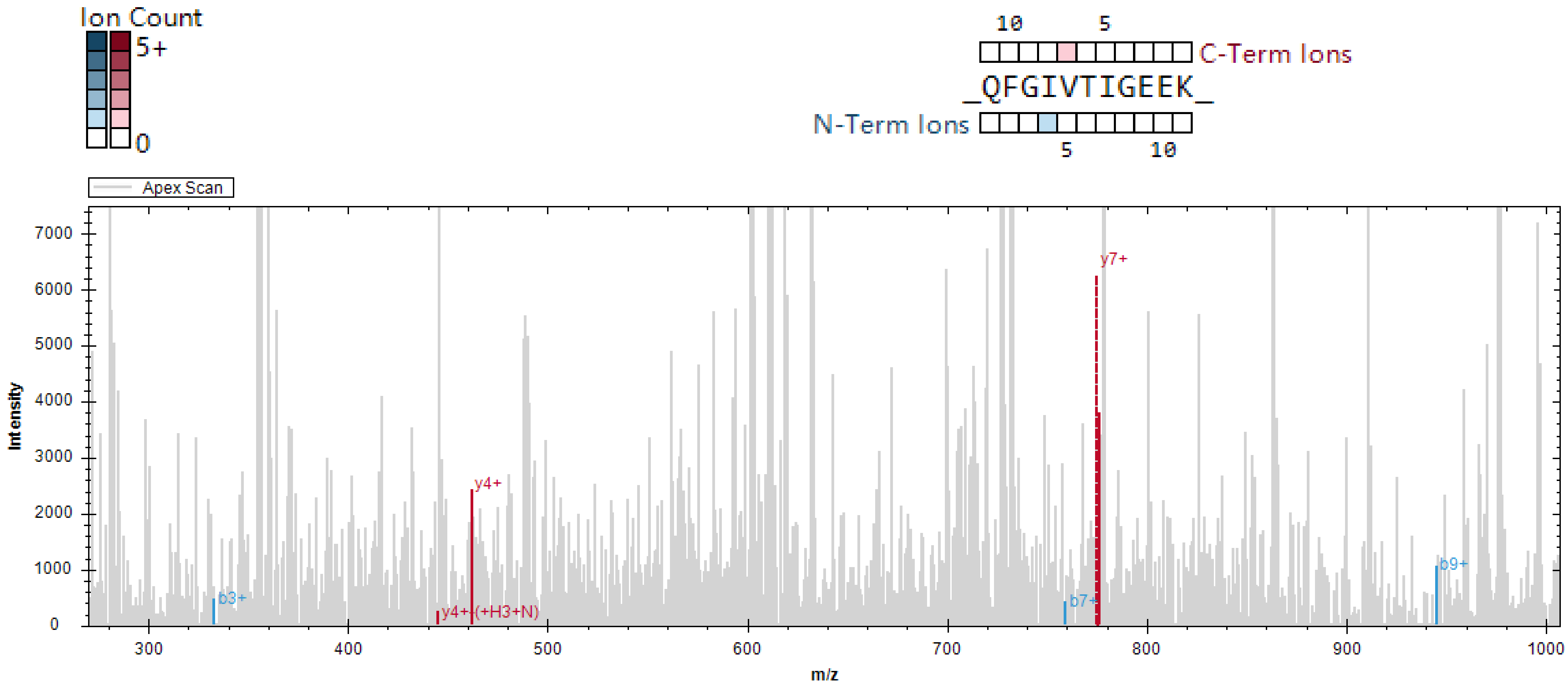

Ion Count
5+
0
10
5
C-Term Ions
_QFGIVTIGEEK_
N-Term Ions
5
10
Apex Scan
Intensity
m/z
y7+
y4+
y4+-(+H3+N)
b3+
b7+
b9+
0
1000
2000
3000
4000
5000
6000
7000
300
400
500
600
700
800
900
1000

# P51679

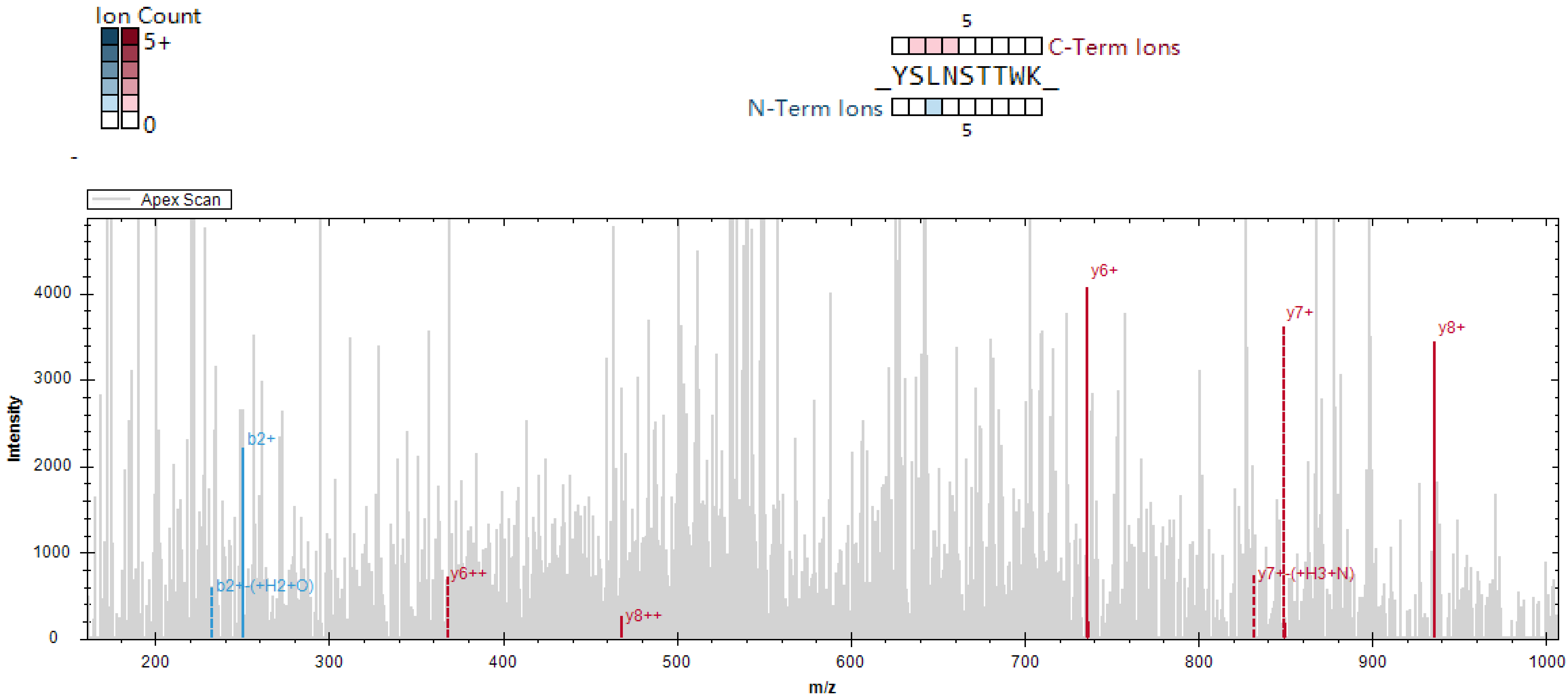

Ion Count
5+
0
5
C-Term Ions
_YSLNSTTWK_
N-Term Ions
5
Apex Scan
Intensity
m/z
y6+
y7+
y8+
b2+
b2+-(+H2+O)
y6++
y8++
y7+-(+H3+N)

P52895

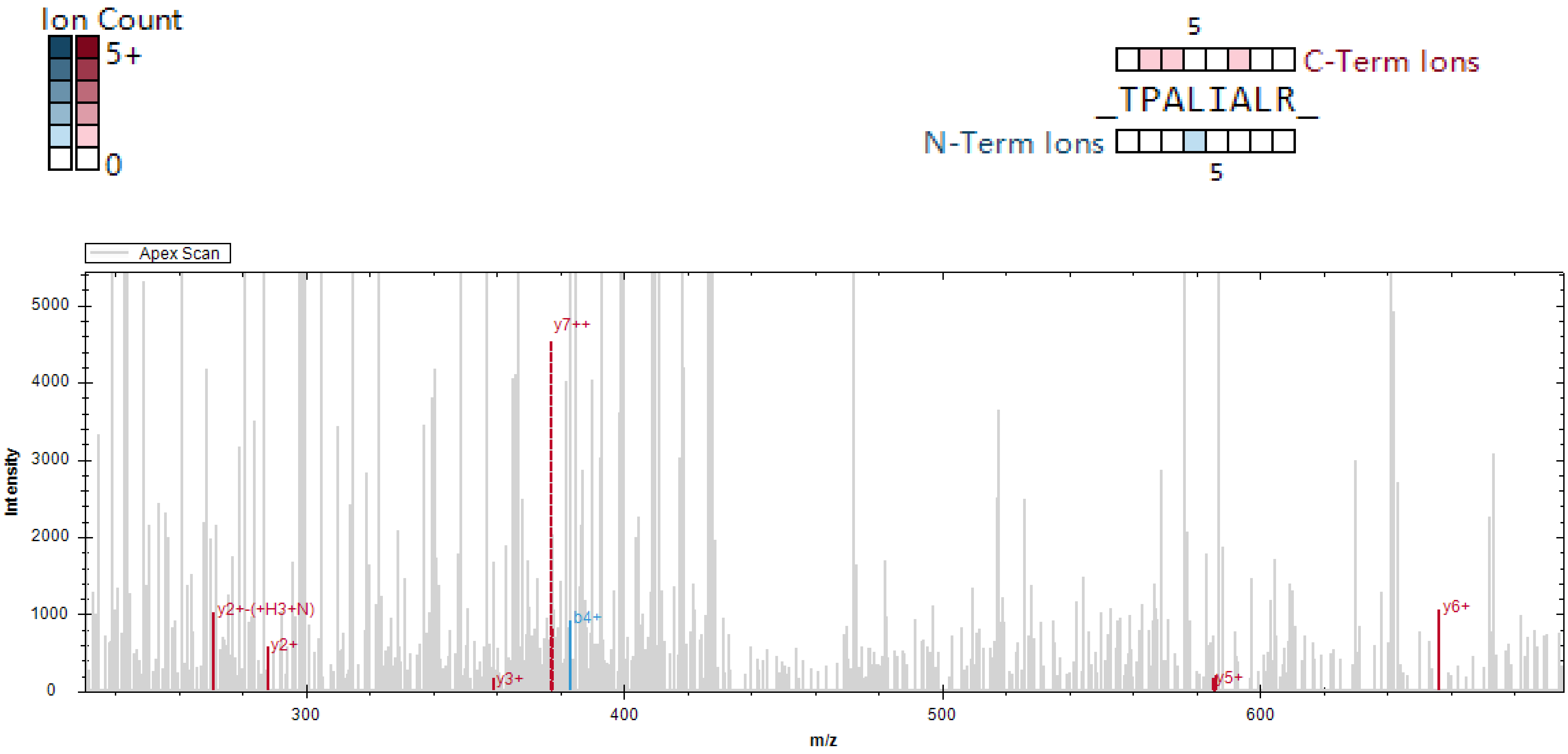

Ion Count
5+
0
5
C-Term Ions
_TPALIALR_
N-Term Ions
5
Apex Scan
y7++
y2+-(+H3+N)
y2+
y3+
b4+
y5+
y6+
Intensity
m/z

# P53384

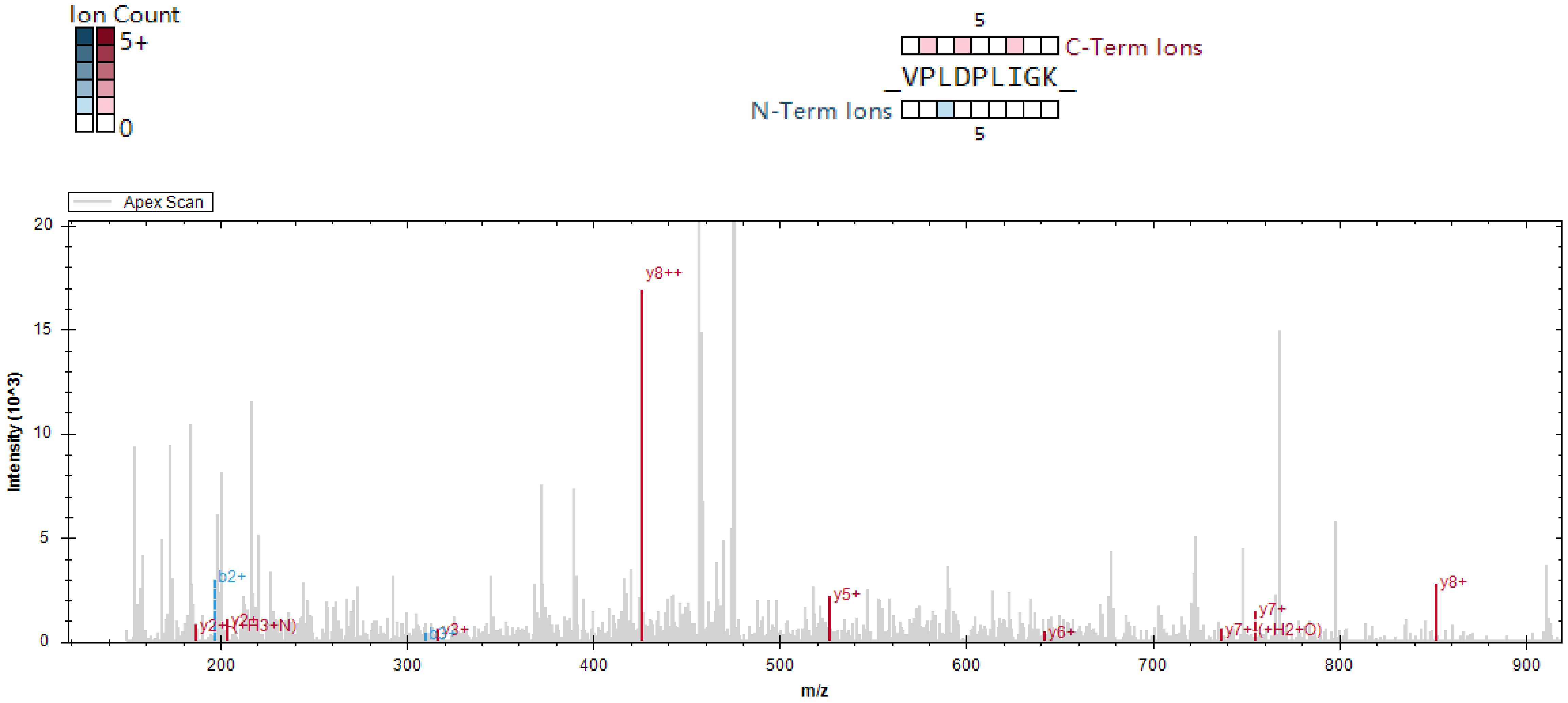

Ion Count
5+
0
5
C-Term Ions
_VPLDPLIGK_
N-Term Ions
5
Apex Scan
Intensity (10^3)
m/z
y8++
b2+
y2+
y5+
y6+
y7+
y8+

P53420

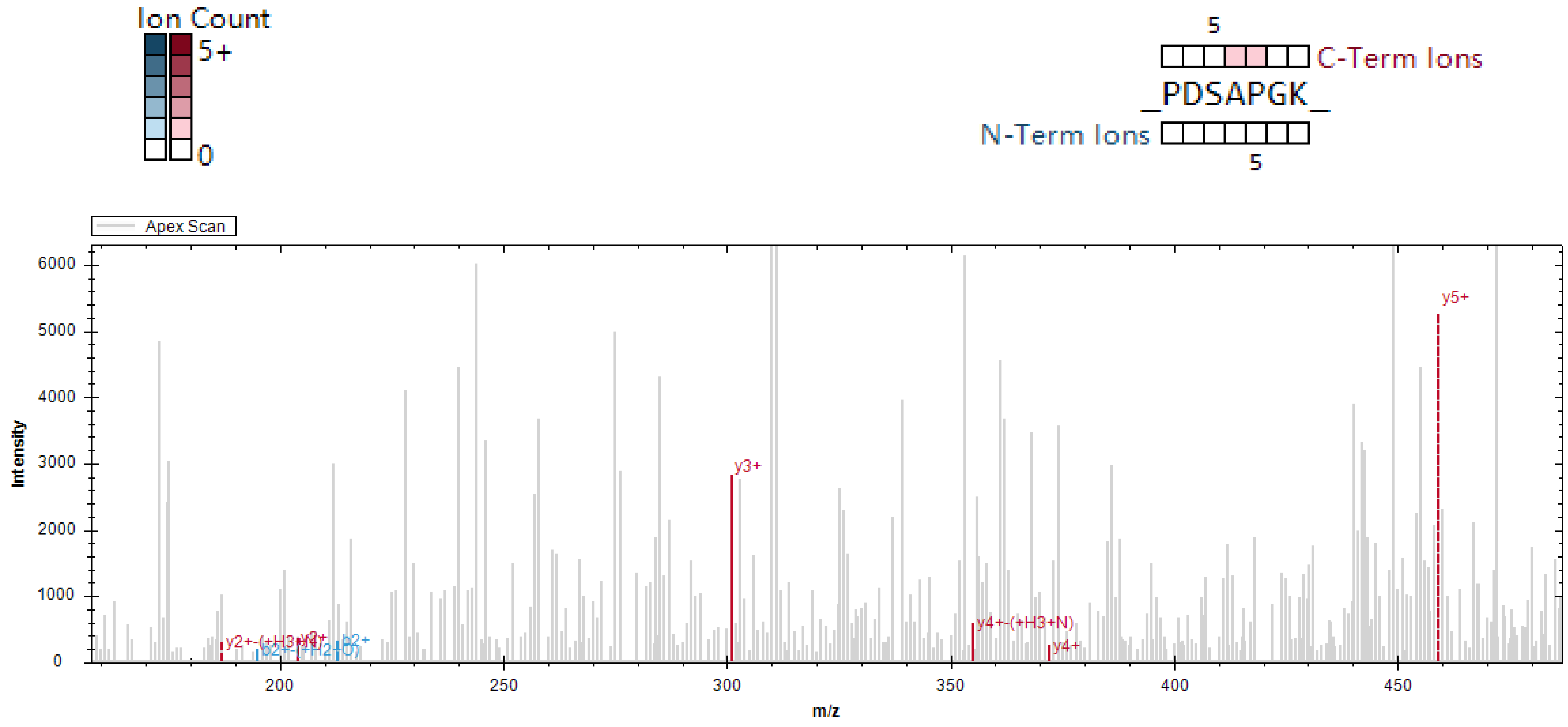

Ion Count
5+
0
5
C-Term Ions
_PDSAPGK_
N-Term Ions
5
Apex Scan
Intensity
m/z
y5+
y3+
y4+-(+H3+N)
y4+
b2+

P53708

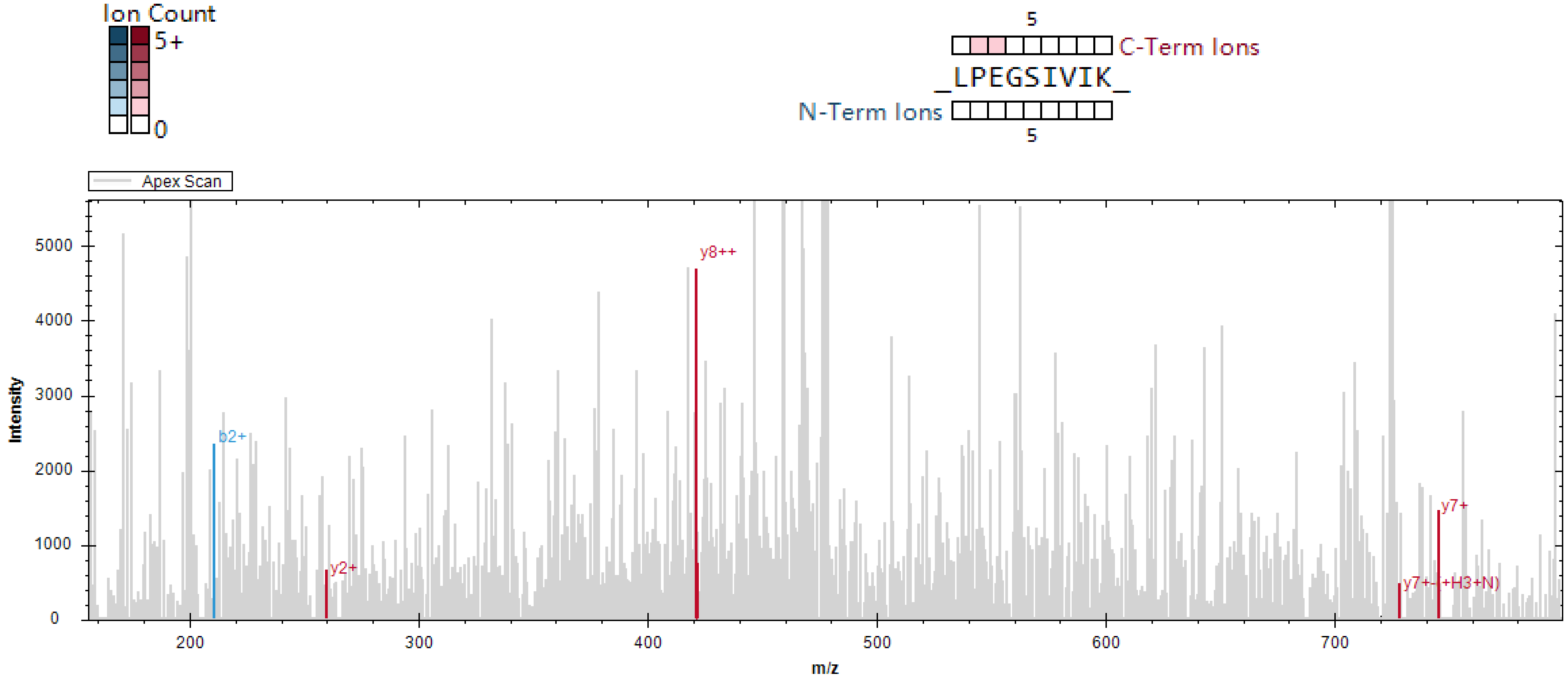

Ion Count
5+
0
5
C-Term Ions
_LPEGSIVIK_
N-Term Ions
5
Apex Scan
Intensity
m/z
b2+
y2+
y8++
y7+
y7+-(+H3+N)

# P54277

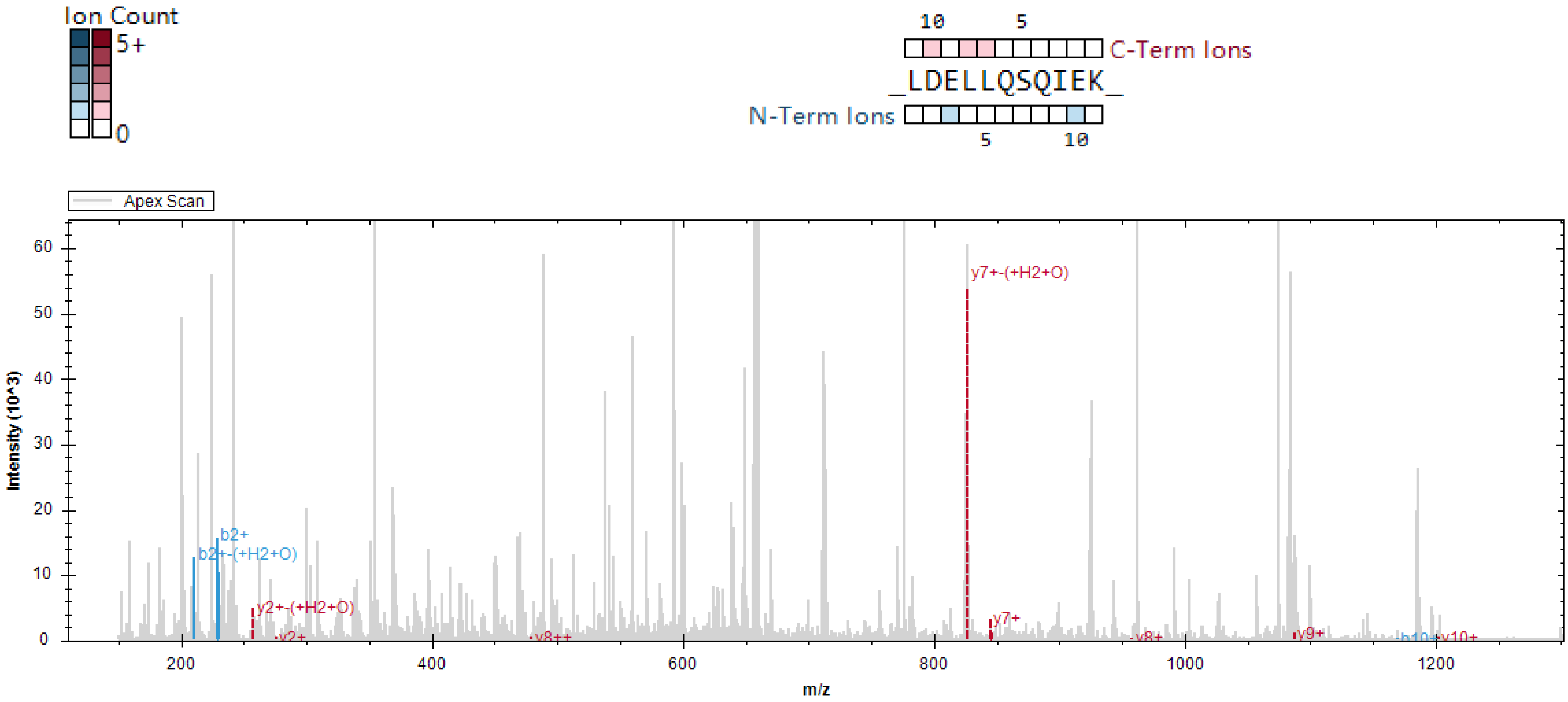

Ion Count
5+
0
10
5
C-Term Ions
_LDELLQSQIEK_
N-Term Ions
5
10
Apex Scan
Intensity (10^3)
m/z
b2+
b2+-(+H2+O)
y2+-(+H2+O)
y7+-(+H2+O)
y7+
y9+

P54852

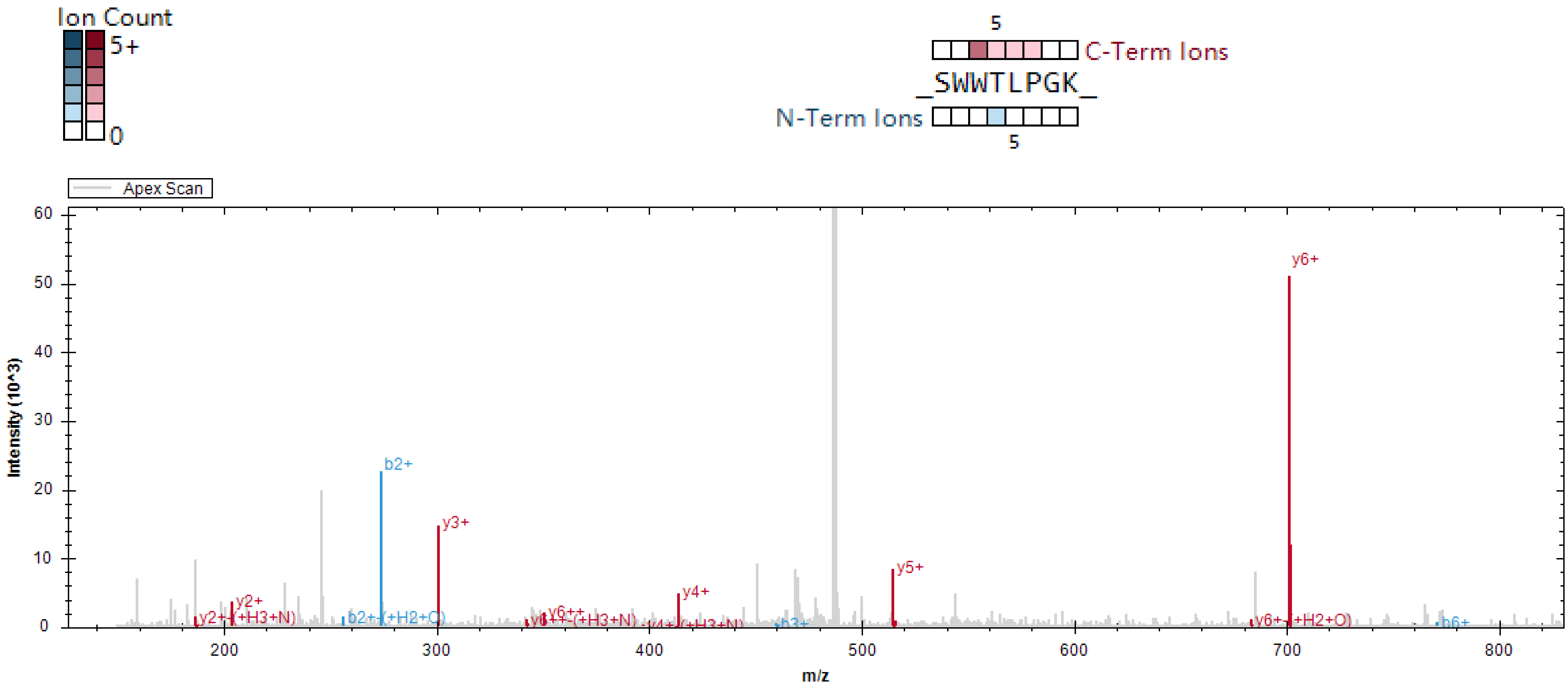

Ion Count
5+
0
5
C-Term Ions
_SWWTLPGK_
N-Term Ions
5
Apex Scan
Intensity (10^3)
m/z
y6+
b2+
y3+
y5+
y4+
y2+
y2+-(+H3+N)
b2+-(+H2+O)
y6++
y6+-(+H2+O)
b6+

P56181

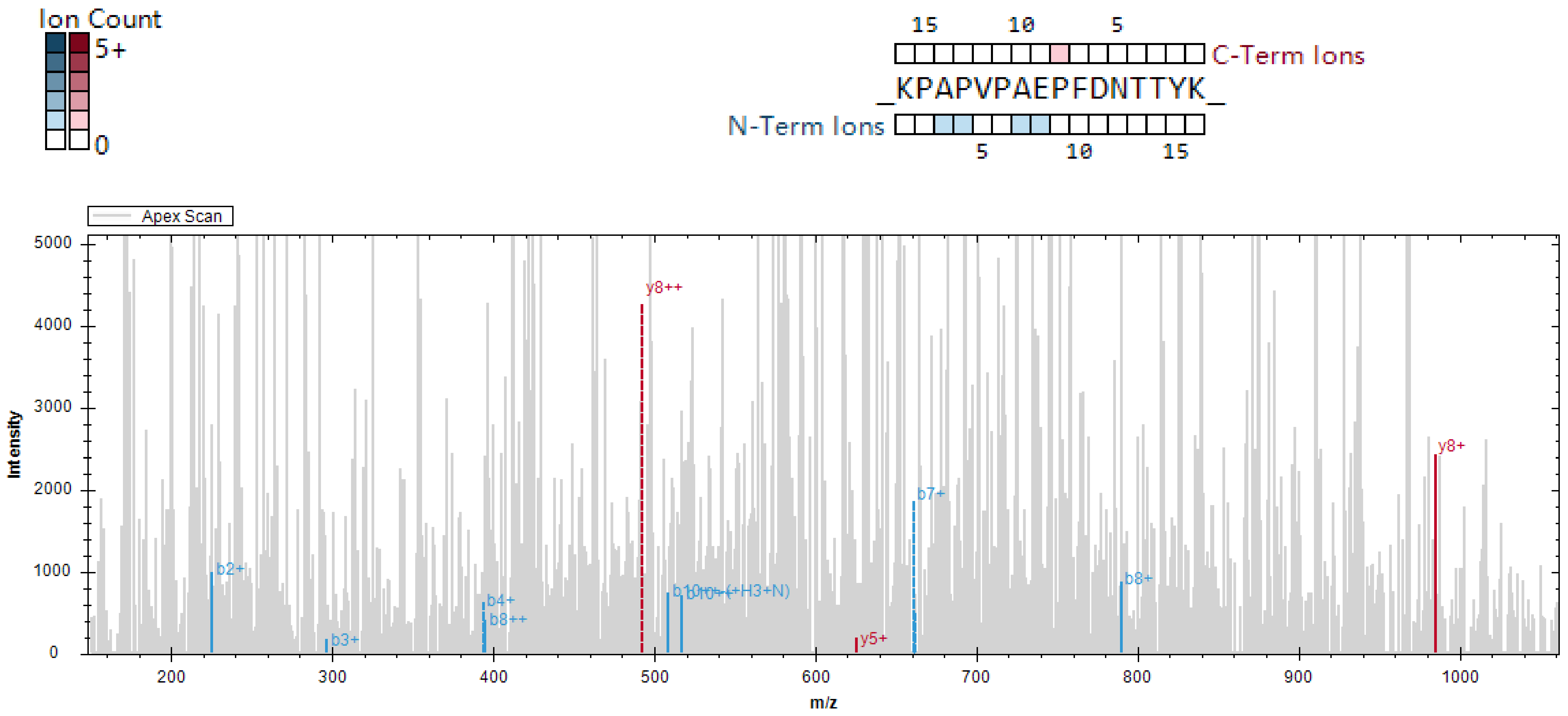

Ion Count
5+
0
15 10 5
C-Term Ions
_KPAPVPAEPFDNTTYK_
N-Term Ions
5 10 15
Apex Scan
Intensity
m/z
y8++
y8+
b7+
b2+
b8+
b4+
b8++
b3+
y5+
b10++ (+H3+N)
b10++

P57075

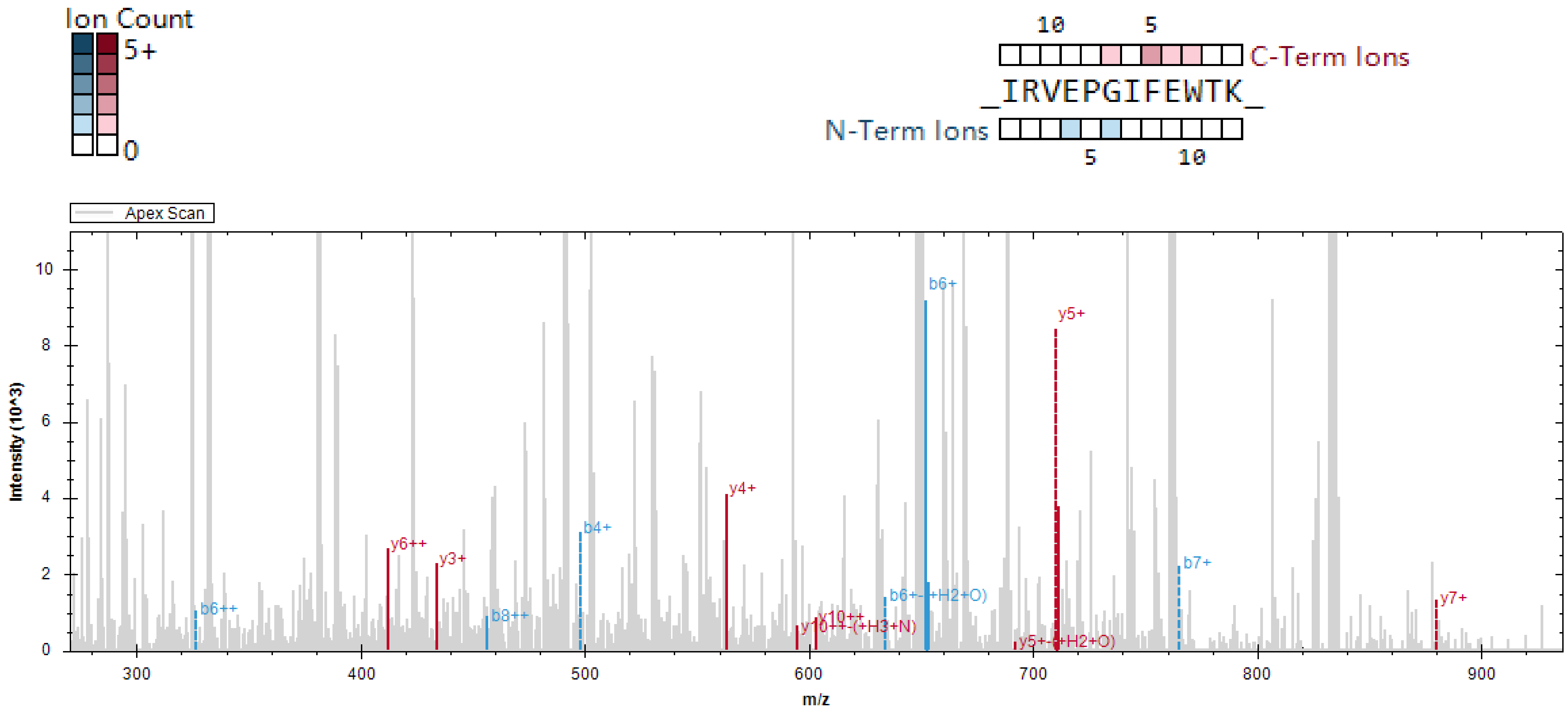

Ion Count
5+
0
10
5
C-Term Ions
_IRVEPGIFEWTK_
N-Term Ions
5
10
Apex Scan
Intensity (10^3)
m/z
b6+
y5+
y4+
b4+
y6++
y3+
b7+
y7+
b6++
b8++
b6+-(+H2+O)
y10++
y5+-(+H2+O)

P60673

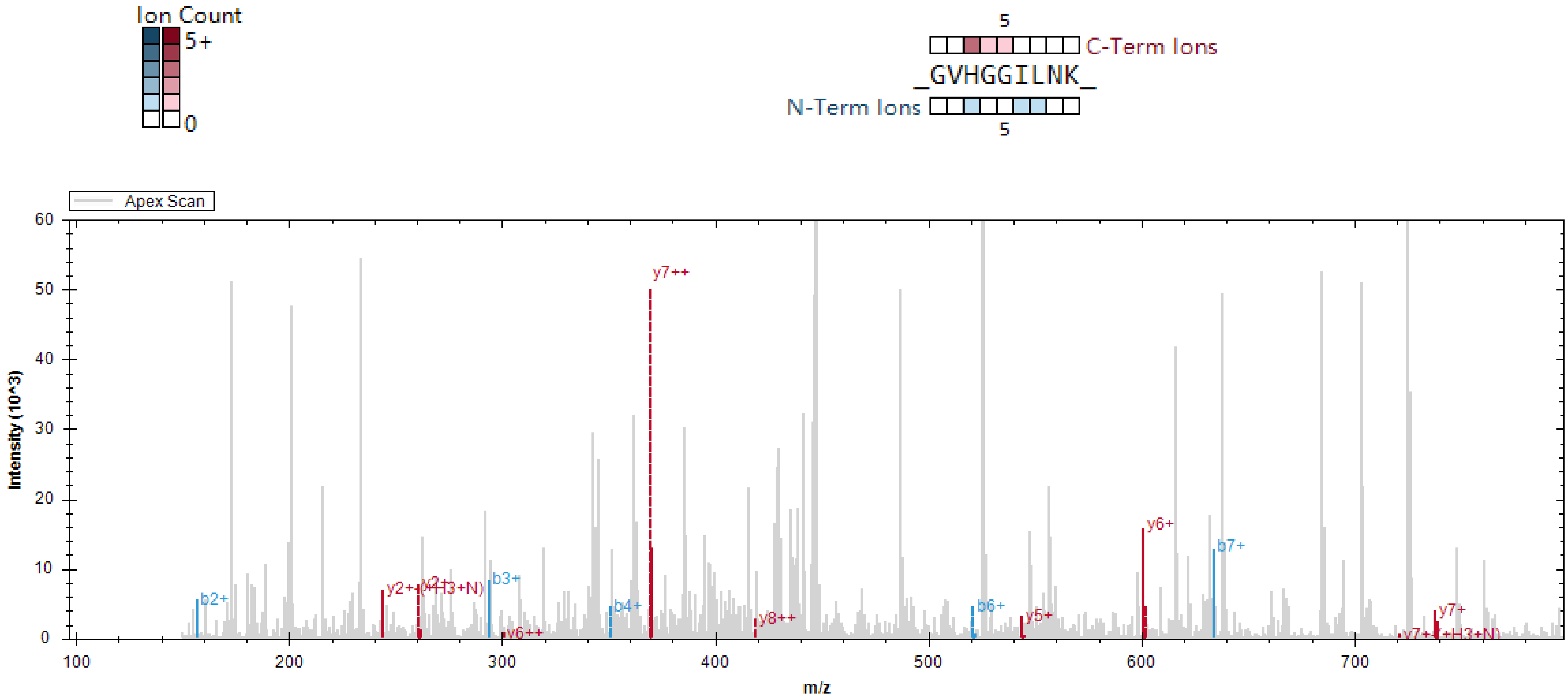

Ion Count
5+
0
5
C-Term Ions
_GVHGGILNK_
N-Term Ions
5
Apex Scan
Intensity (10^3)
m/z
y7++
y6+
b7+
b2+
y2+
b3+
b4+
b6+
y8++
y5+
y6++
y7+

P61077

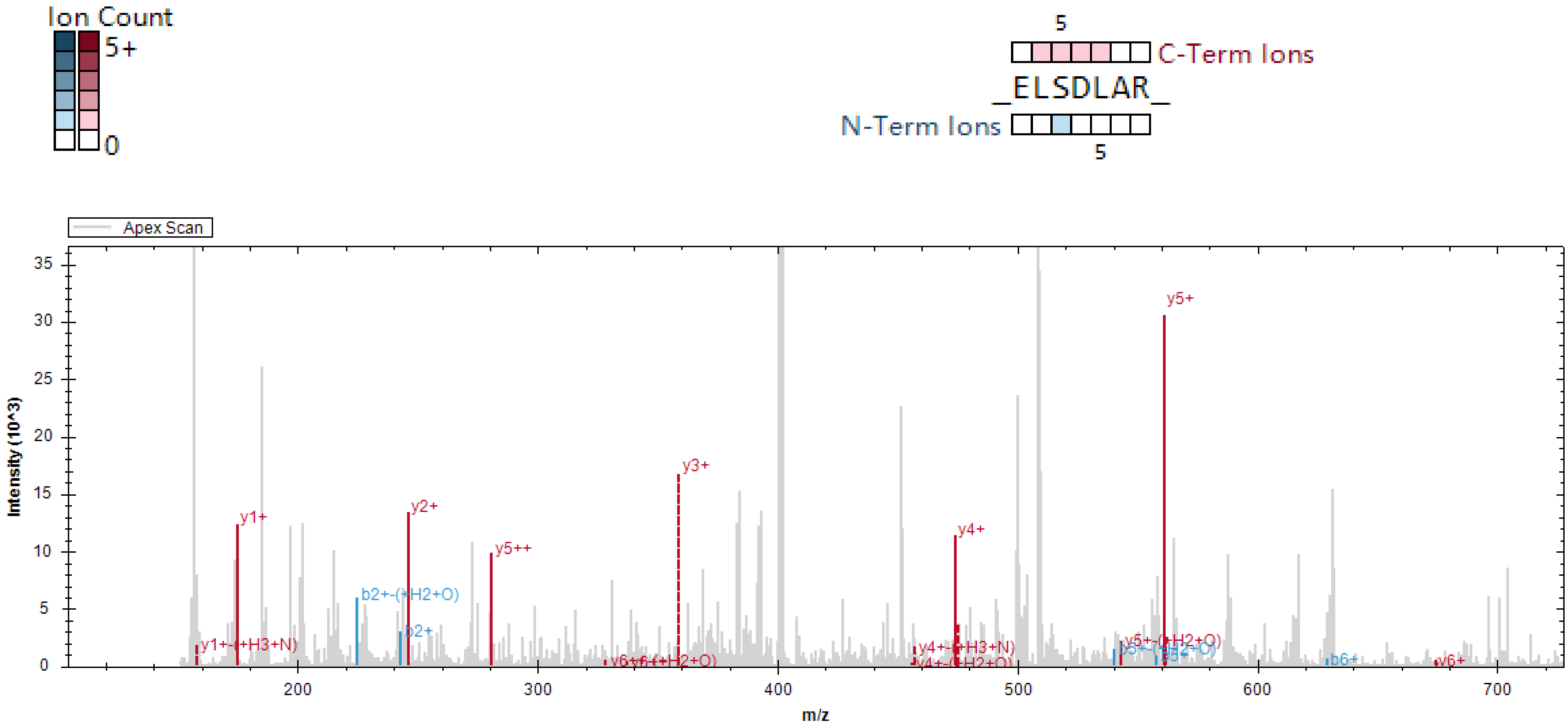

Ion Count
5+
0
5
C-Term Ions
_ELSDLAR_
N-Term Ions
5
Apex Scan
Intensity (10^3)
m/z
y1+
y1+-(+H3+N)
b2+-(+H2+O)
b2+
y2+
y5++
y3+
y4+
y5+
b6+
y6+

P63313

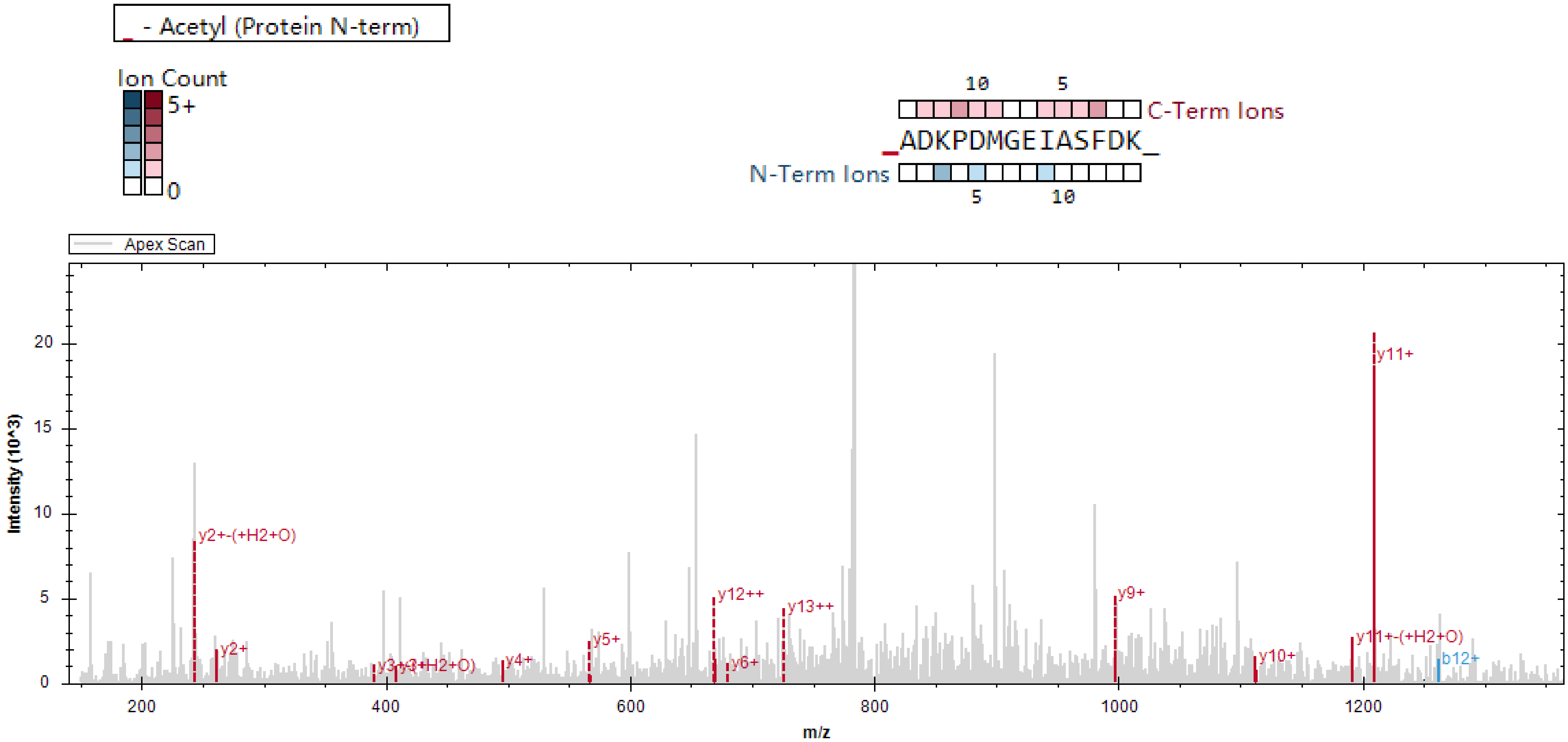

_ - Acetyl (Protein N-term)
Ion Count
5+
0
10
5
C-Term Ions
_ADKPDMGEIASFDK_
N-Term Ions
5
10
Apex Scan
Intensity (10^3)
m/z
y2+-(+H2+O)
y2+
y3+
y3+-(+H2+O)
y4+
y5+
y12++
y6+
y13++
y9+
y10+
y11+
y11+-(+H2+O)
b12+

# P68104

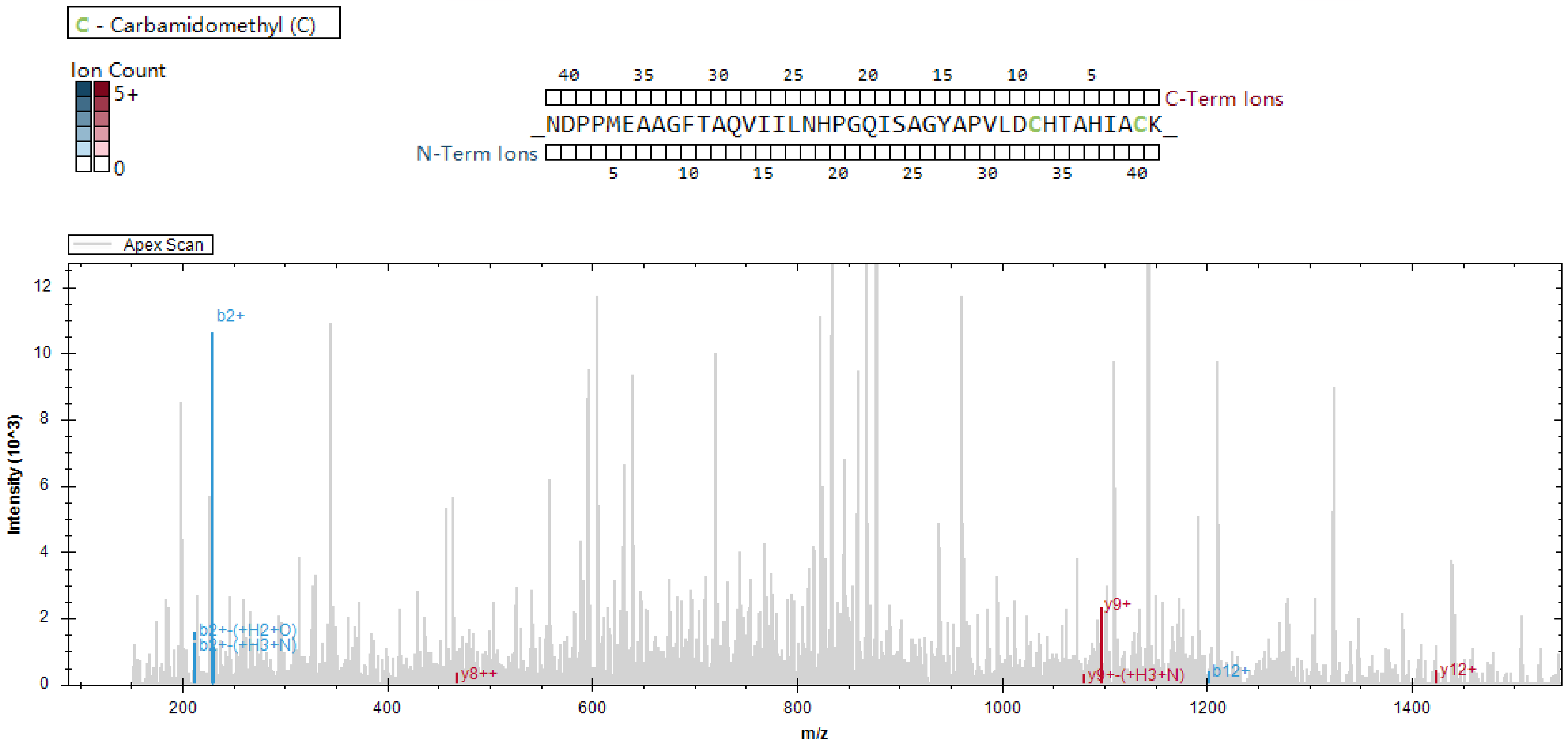

C - Carbamidomethyl (C)
Ion Count
5+
0
C-Term Ions
_NDPPMEAAGFTAQVIILNHPGQISAGYAPVLDCHTAHIACK_
N-Term Ions
Apex Scan
b2+
b2+-(+H2+O)
b2+-(+H3+N)
y8++
y9+
y9+-(+H3+N)
b12+
y12+
Intensity (10^3)
m/z

# P78368

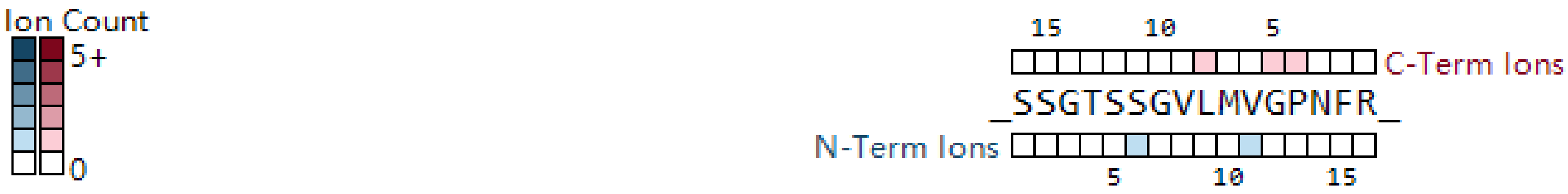

Ion Count
5+
0
15
10
5
C-Term Ions
_SSGTSSGVLMVGPNFR_
N-Term Ions
5
10
15


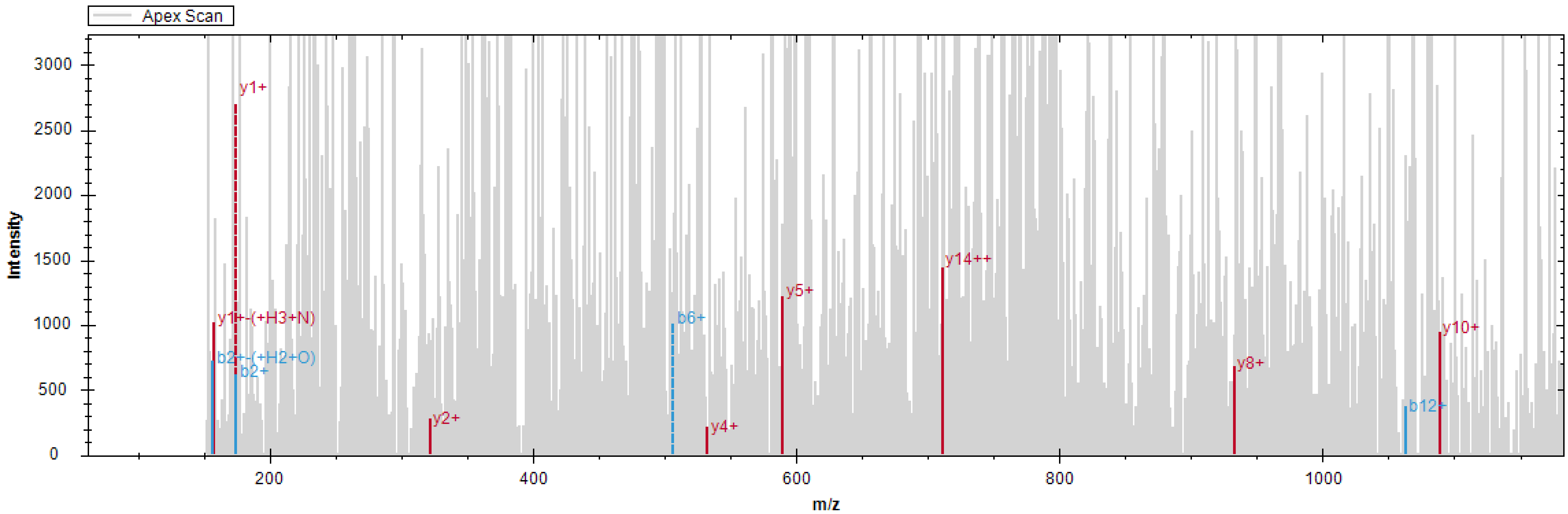

Apex Scan
Intensity
m/z
y1+
y1+-(+H3+N)
b2+-(+H2+O)
b2+
y2+
b6+
y4+
y5+
y14++
y8+
b12+
y10+

# P78385

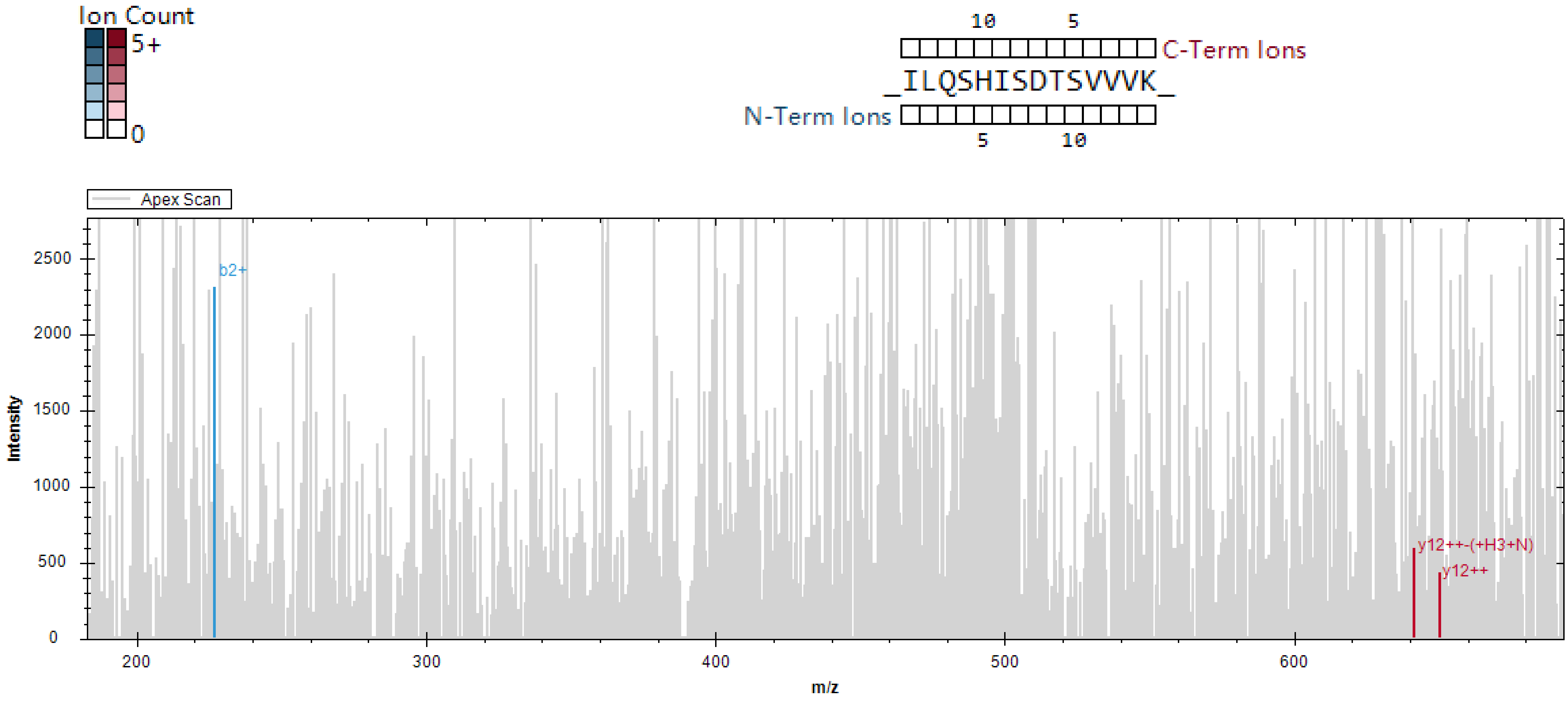

Ion Count
5+
0
10
5
C-Term Ions
_ILQSHISDTSVVVK_
N-Term Ions
5
10
Apex Scan
b2+
y12++-(+H3+N)
y12++
Intensity
m/z
0
500
1000
1500
2000
2500
200
300
400
500
600

P78556

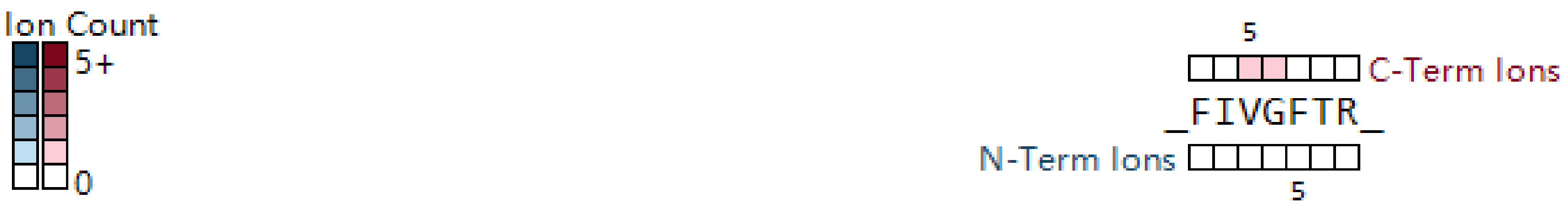
Ion Count
5+
0
5
C-Term Ions
_FIVGFTR_
N-Term Ions
5

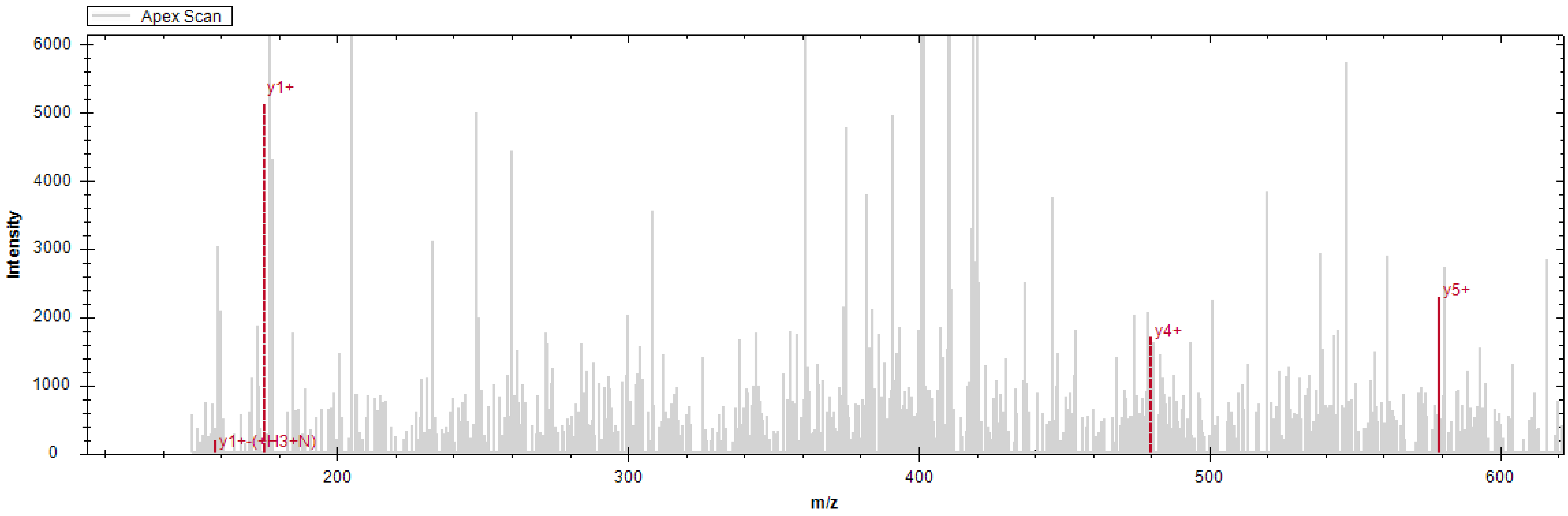
Apex Scan
Intensity
m/z
y1+
y1+-(H3+N)
y4+
y5+
6000
5000
4000
3000
2000
1000
0
200
300
400
500
600

P83110

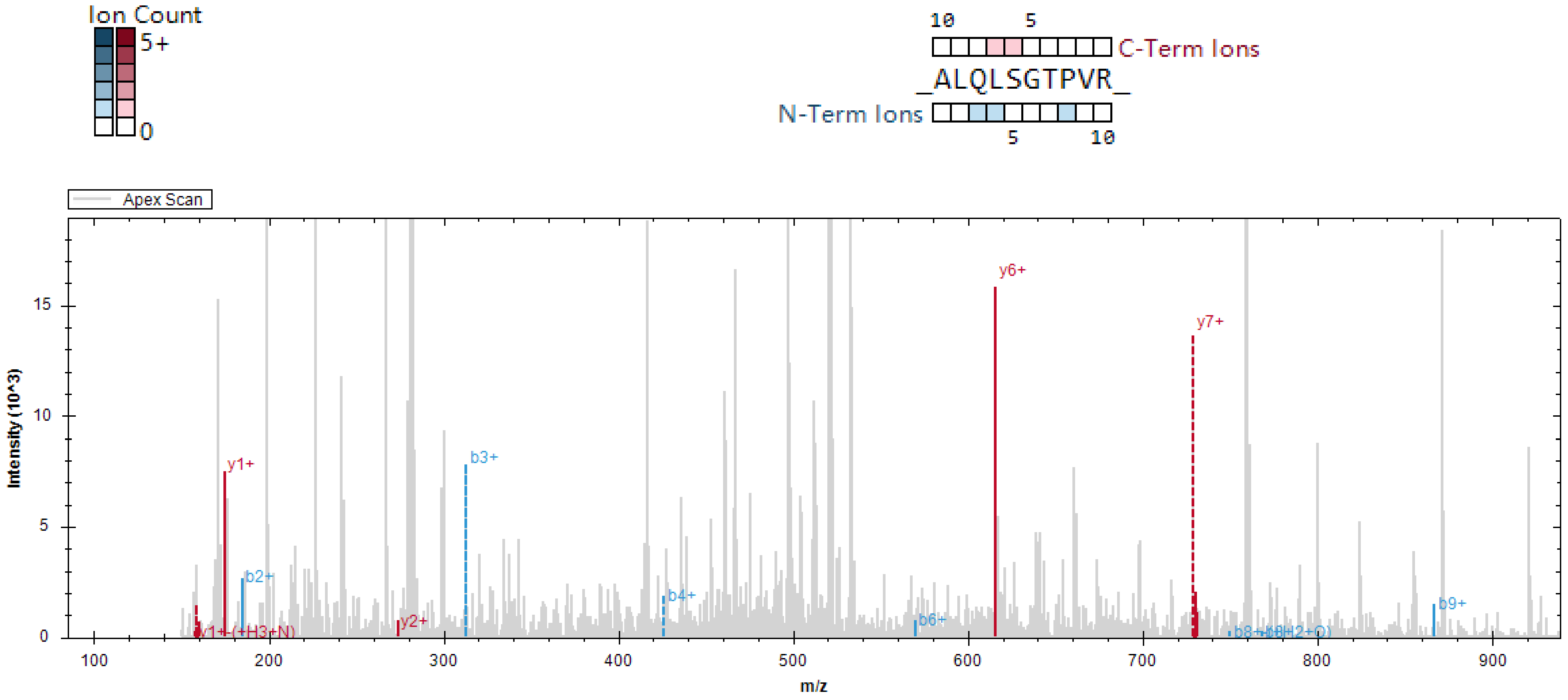

Ion Count
5+
0
10
5
C-Term Ions
_ALQLSGTPVR_
N-Term Ions
5
10
Apex Scan
Intensity (10^3)
m/z
y1+
y1+-(+H3+N)
b2+
y2+
b3+
b4+
b6+
y6+
y7+
b8+-(+H2+O)
b9+

P98164

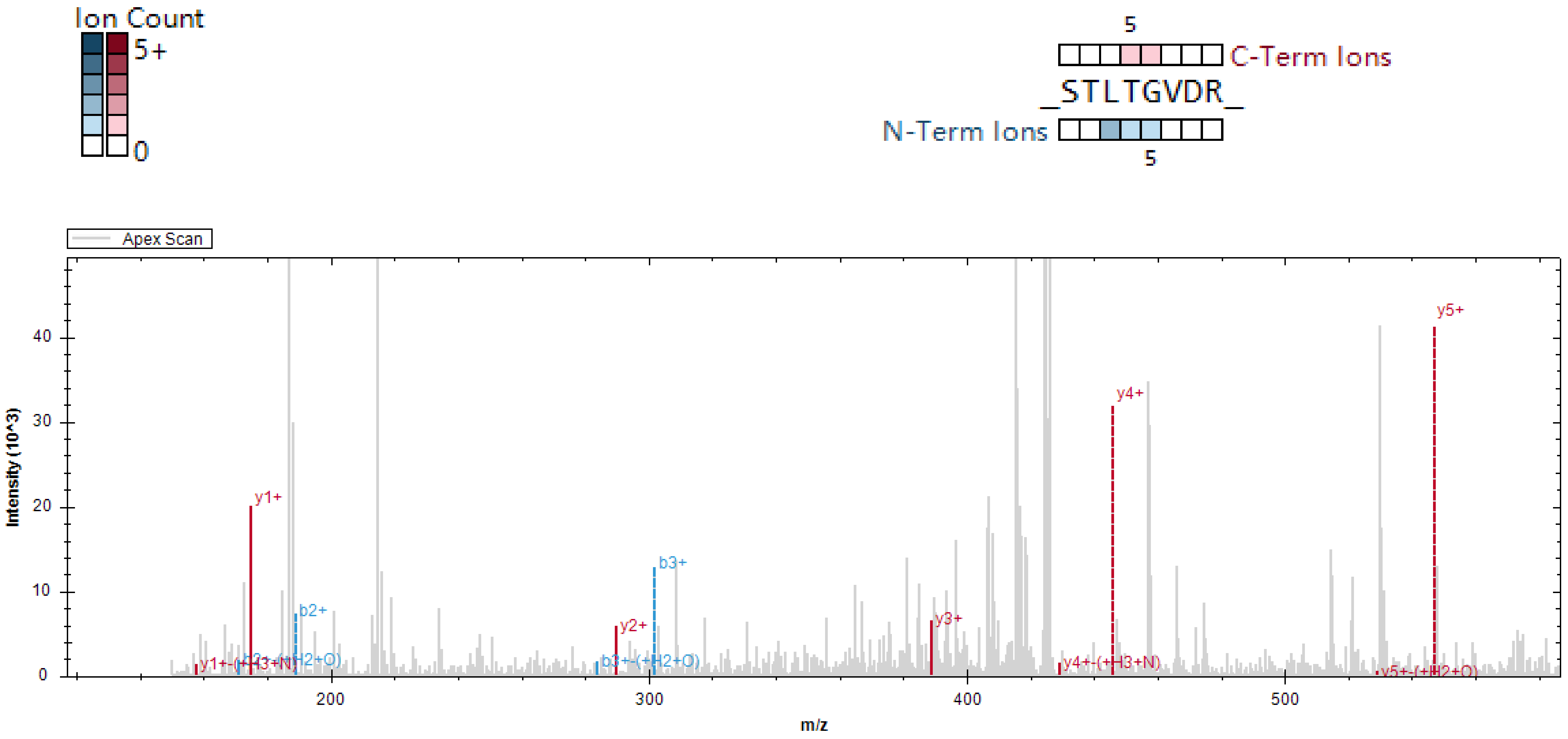

Ion Count
5+
0
5
C-Term Ions
_STLTGVDR_
N-Term Ions
5
Apex Scan
Intensity (10^3)
0
10
20
30
40
y1+
b2+
b3+
y2+
y3+
y4+
y5+
y1+-(+H3+N)
b2+-(+H2+O)
b3+-(+H2+O)
y4+-(+H3+N)
y5+-(+H2+O)
200
300
400
500
m/z

Q01130

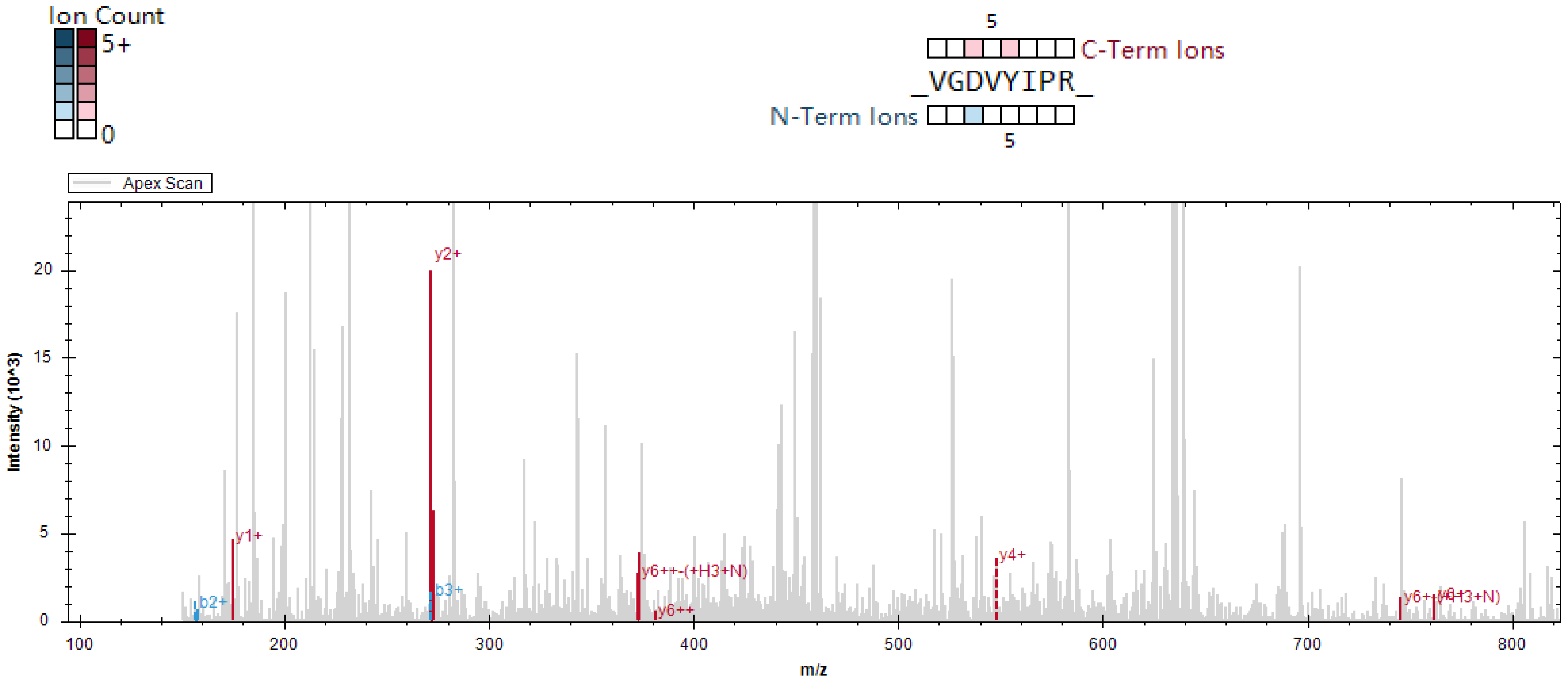
Ion Count
5+
0
5
C-Term Ions
_VGDVYIPR_
N-Term Ions
5
Apex Scan
y2+
y1+
b2+
b3+
y6++-(+H3+N)
y6++
y4+
y6+
Intensity (10^3)
m/z
0
5
10
15
20
100
200
300
400
500
600
700
800

Q02509

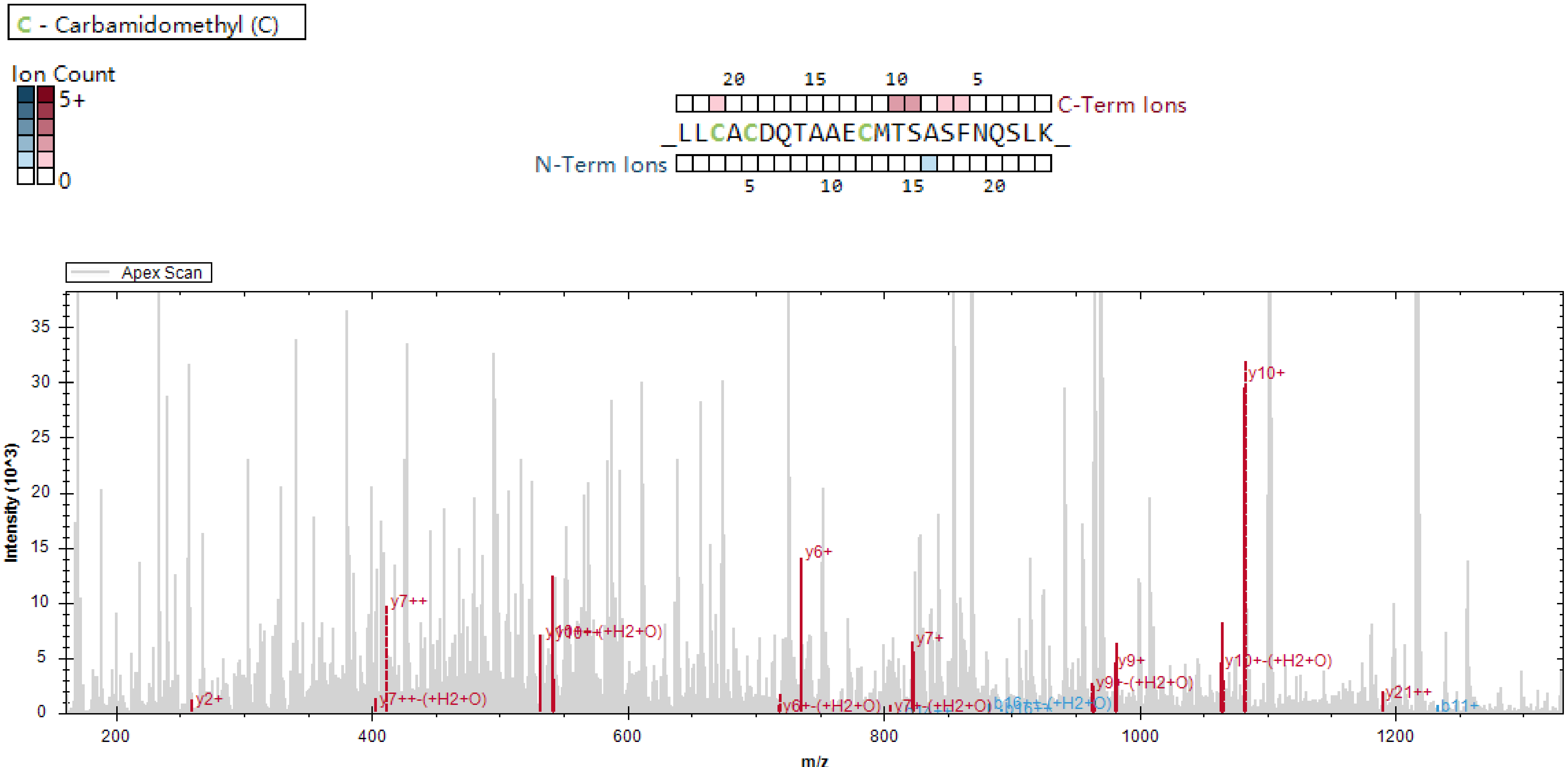

C - Carbamidomethyl (C)
Ion Count
5+
0
20 15 10 5
C-Term Ions
_LLCACDQTAAECMTSASFNQSLK_
N-Term Ions
5 10 15 20
Apex Scan
Intensity (10^3)
m/z
y10+
y6+
y7++
y7+
y9+
y2+
y7++-(+H2+O)
y6+-(+H2+O)
y9+-(+H2+O)
y21++
b11+

Q04721

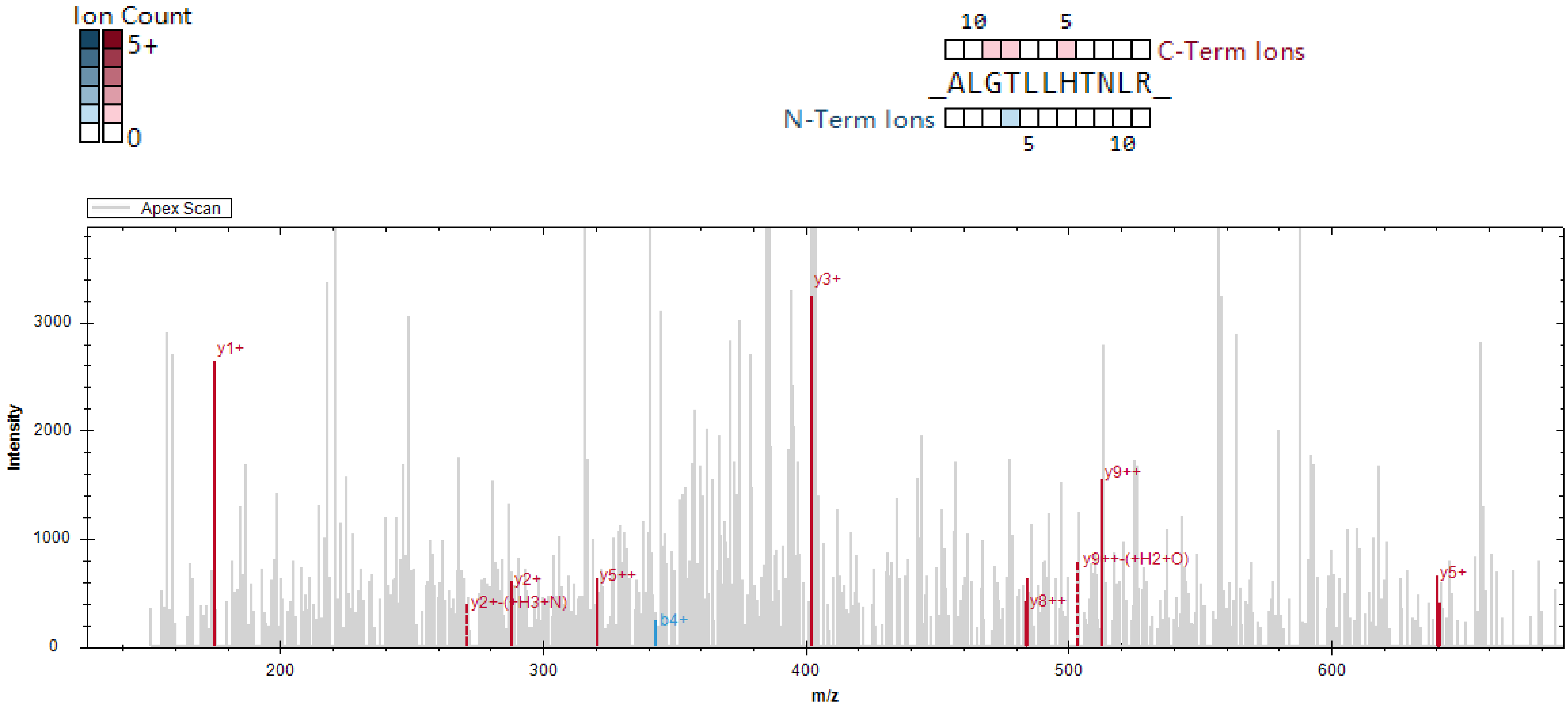

Ion Count
5+
0
10
5
C-Term Ions
_ALGTLLHTNLR_
N-Term Ions
5
10
Apex Scan
Intensity
m/z
y1+
y2+-(+H3+N)
y2+
y5++
b4+
y3+
y8++
y9++-(+H2+O)
y9++
y5+

Q04941

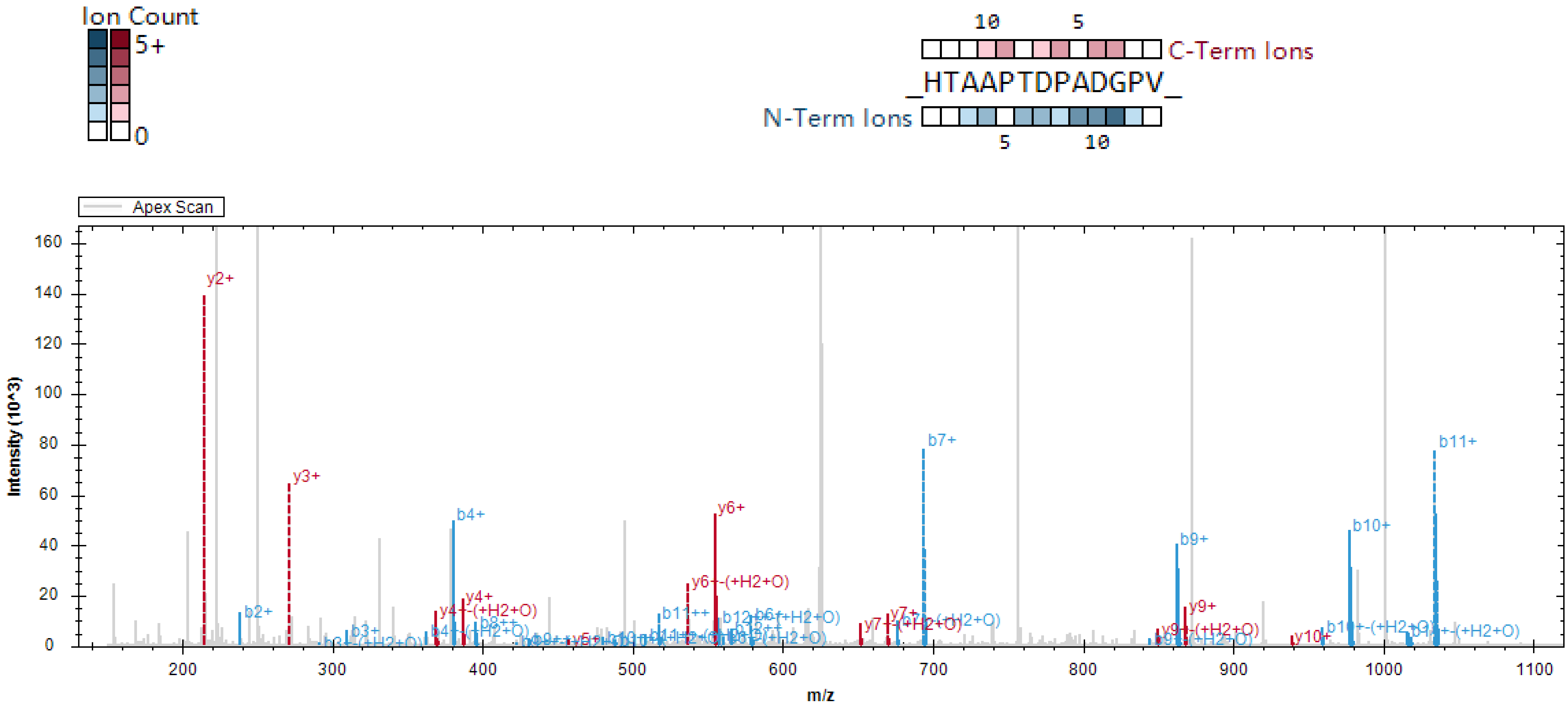

Ion Count
5+
0
10
5
C-Term Ions
_HTAAPTDPADGPV_
N-Term Ions
5
10
Apex Scan
Intensity (10^3)
m/z
y2+
y3+
b4+
y6+
y6+-(+H2+O)
b7+
b9+
b10+
b11+
y9+
y10+

Q07325

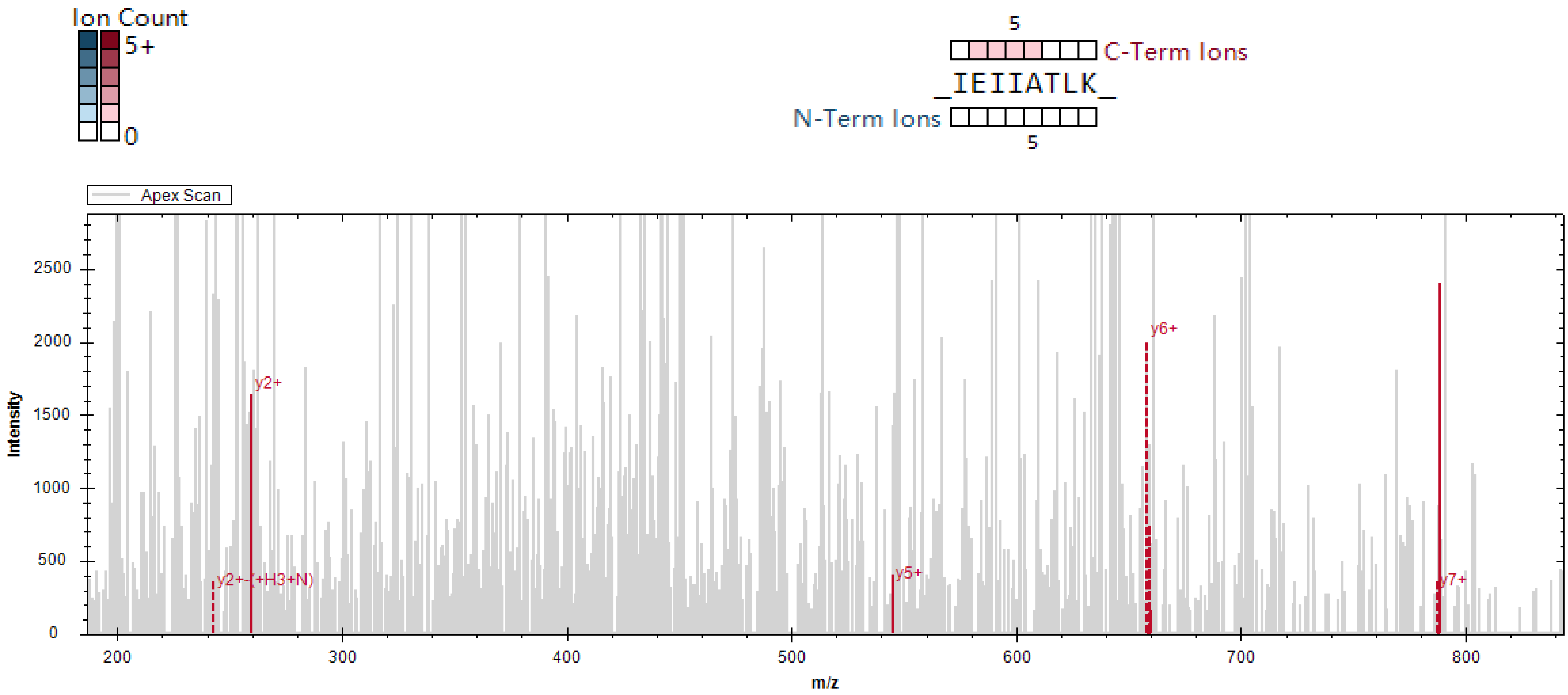
Ion Count
5+
0
5
C-Term Ions
_IEIIATLK_
N-Term Ions
5
Apex Scan
y2+
y2+-(+H3+N)
y5+
y6+
y7+
Intensity
m/z
0
500
1000
1500
2000
2500
200
300
400
500
600
700
800

Q08493

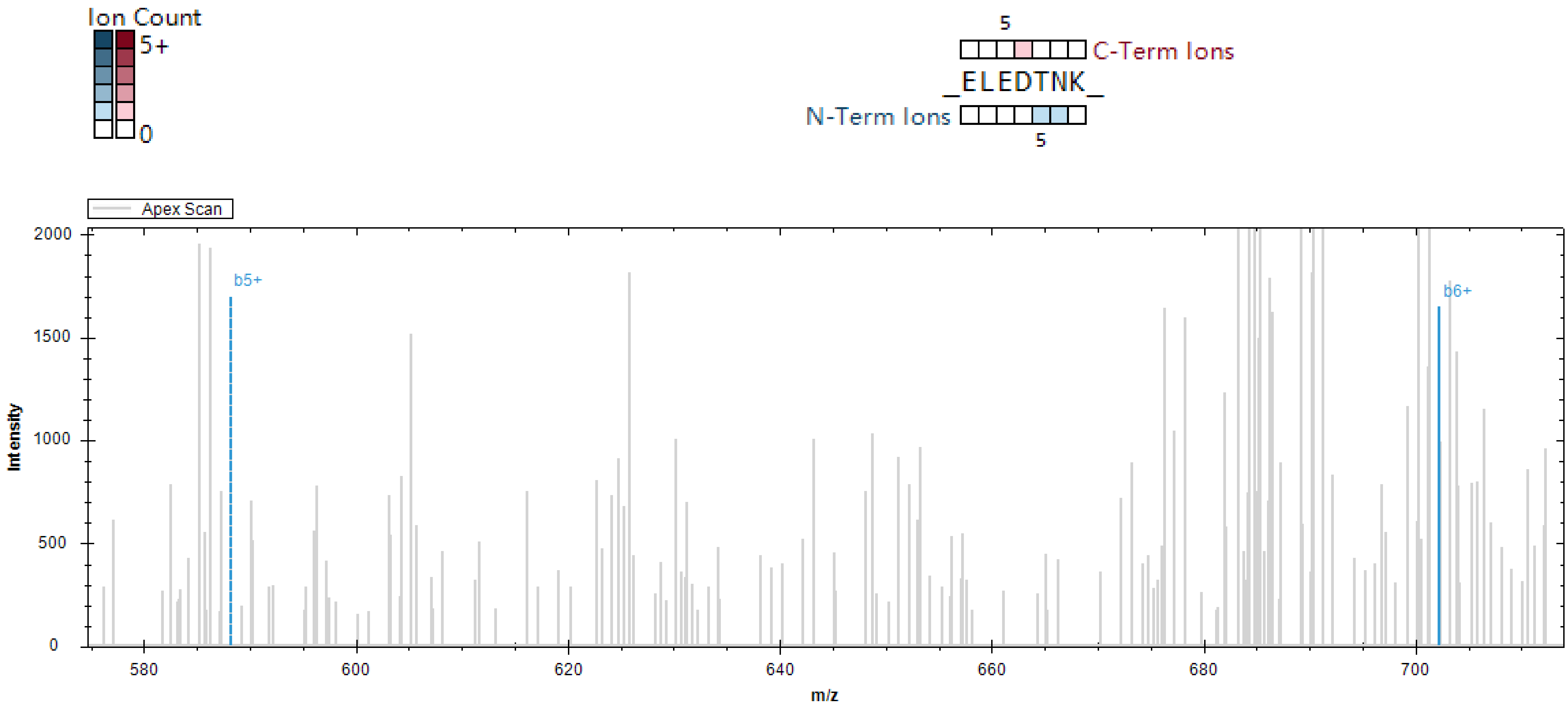

Ion Count
5+
0
5
C-Term Ions
_ELEDTNK_
N-Term Ions
5
Apex Scan
b5+
b6+
2000
1500
1000
500
0
Intensity
580
600
620
640
660
680
700
m/z

Q13258

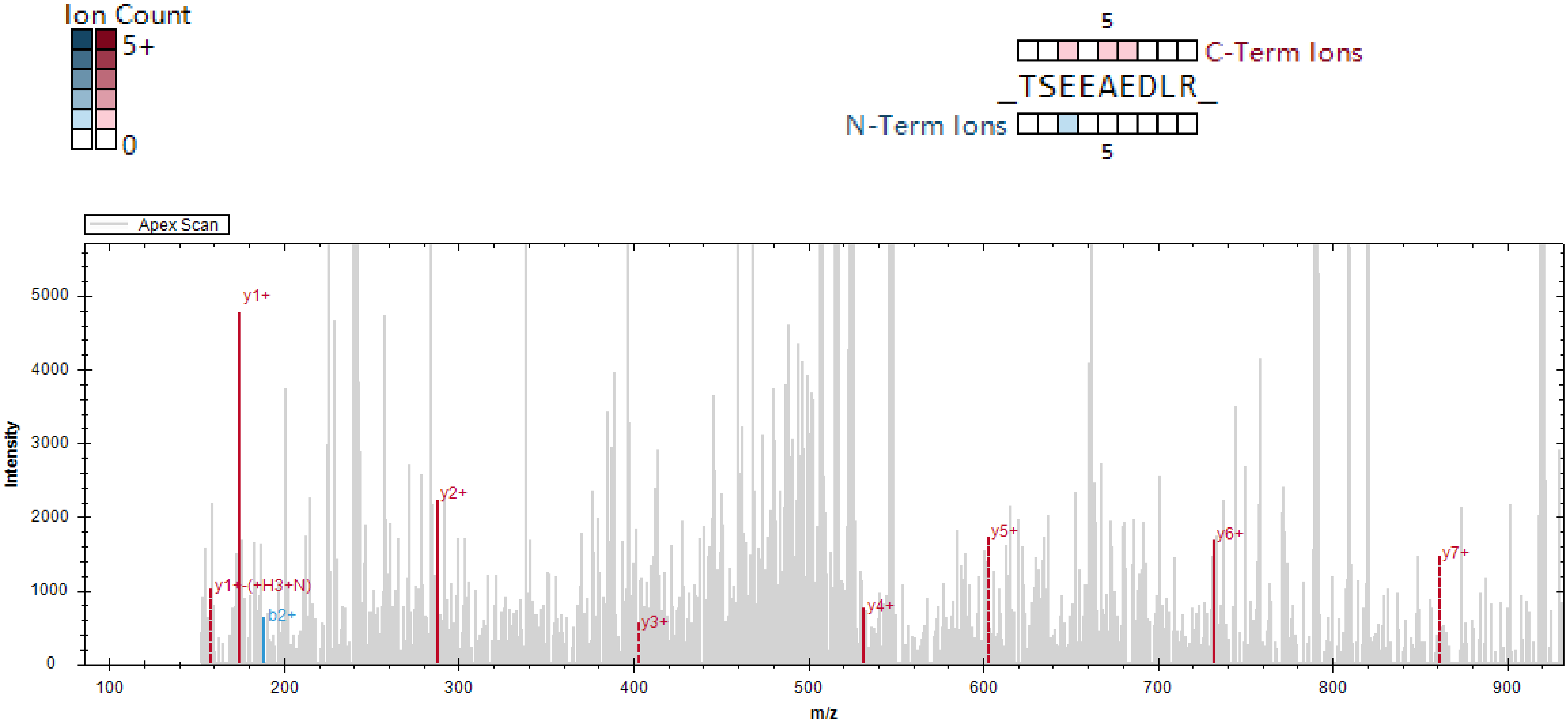

Ion Count
5+
0
5
C-Term Ions
_TSEEAEDLR_
N-Term Ions
5
Apex Scan
y1+
y1+-(+H3+N)
b2+
y2+
y3+
y4+
y5+
y6+
y7+
Intensity
m/z

# Q13325

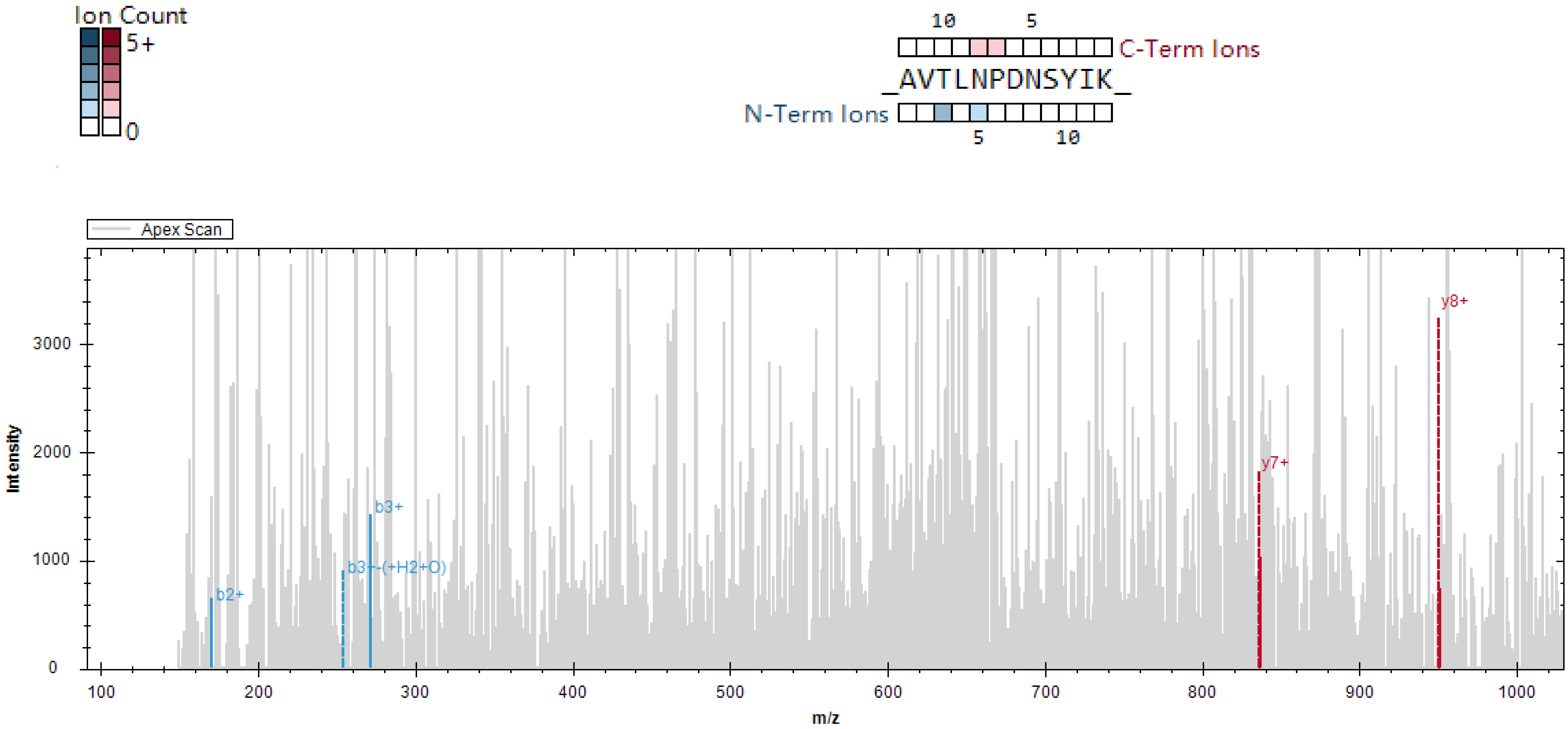

Ion Count
5+
0
10
5
C-Term Ions
_AVTLNPDNSYIK_
N-Term Ions
5
10
Apex Scan
Intensity
3000
2000
1000
0
100
200
300
400
500
600
700
800
900
1000
m/z
b2+
b3+-(+H2+O)
b3+
y7+
y8+

Q13393

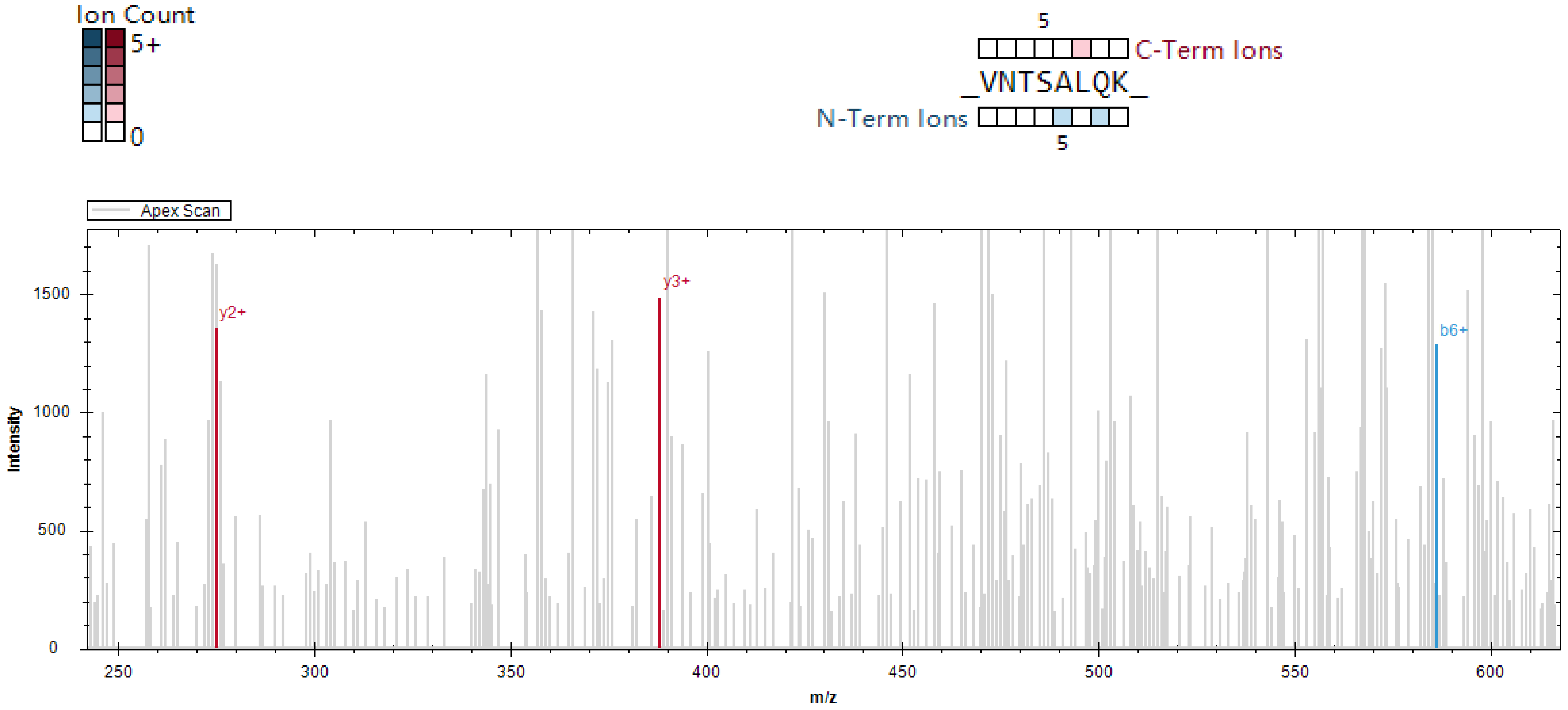

Ion Count
5+
0
5
C-Term Ions
_VNTSALQK_
N-Term Ions
5
Apex Scan
y2+
y3+
b6+
Intensity
m/z

Q13939

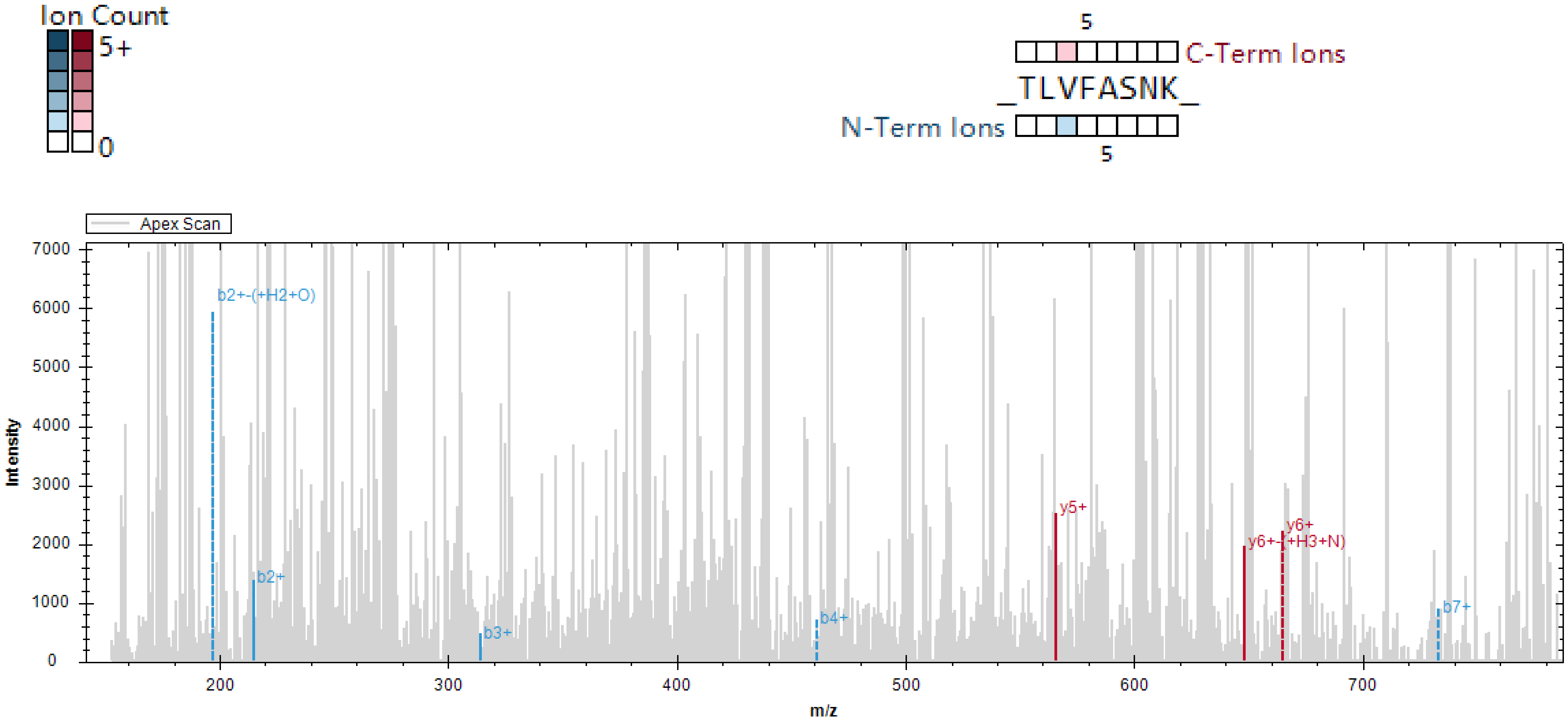

Ion Count
5+
0
5
C-Term Ions
_TLVFASNK_
N-Term Ions
5
Apex Scan
Intensity
m/z
b2+-(+H2+O)
b2+
b3+
b4+
y5+
y6+-(+H3+N)
y6+
b7+

Q14055

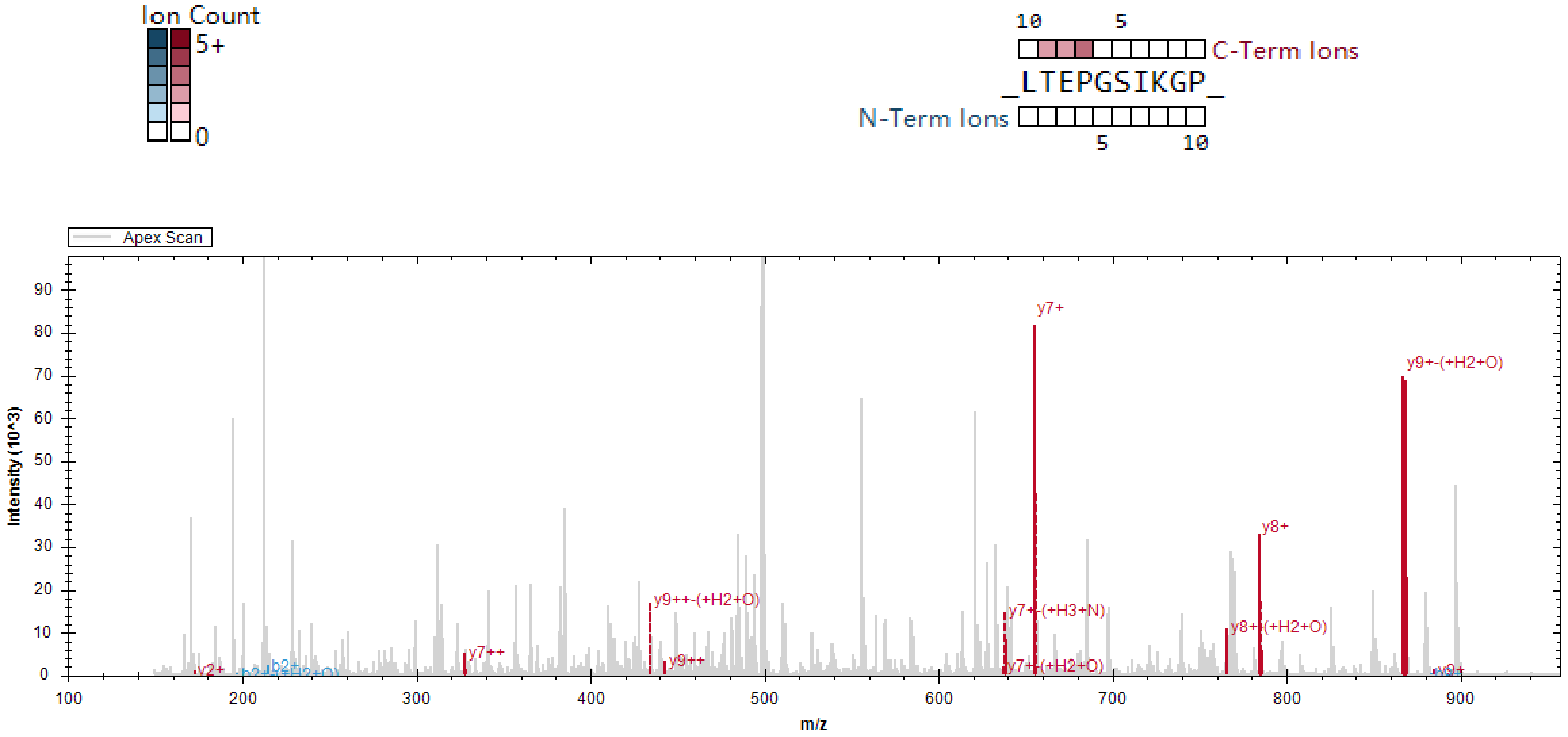

Ion Count
5+
0
10
5
C-Term Ions
_LTEPGSIKGP_
N-Term Ions
5
10
Apex Scan
Intensity (10^3)
m/z
y7+
y9+-(+H2+O)
y8+
y9++-(+H2+O)
y7+-(+H3+N)
y8+-(+H2+O)
y7++
y9++
y7+-(+H2+O)
y2+
b2+

Q14146

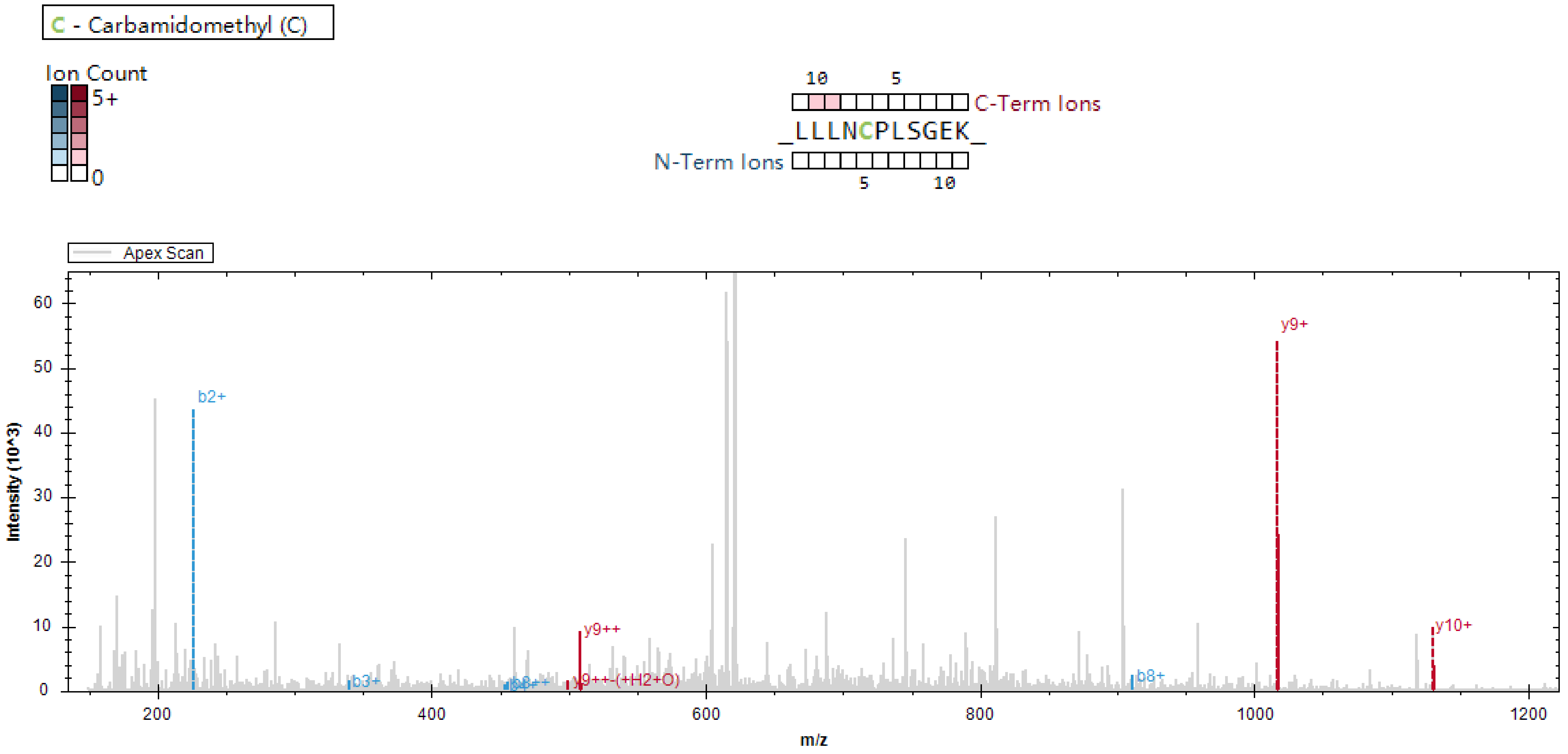

C - Carbamidomethyl (C)
Ion Count
5+
0
10
5
C-Term Ions
_LLLNCPLSGEK_
N-Term Ions
5
10
Apex Scan
y9+
b2+
y9++
y10+
b3+
y9++-(+H2+O)
b8+
Intensity (10^3)
m/z

Q14242

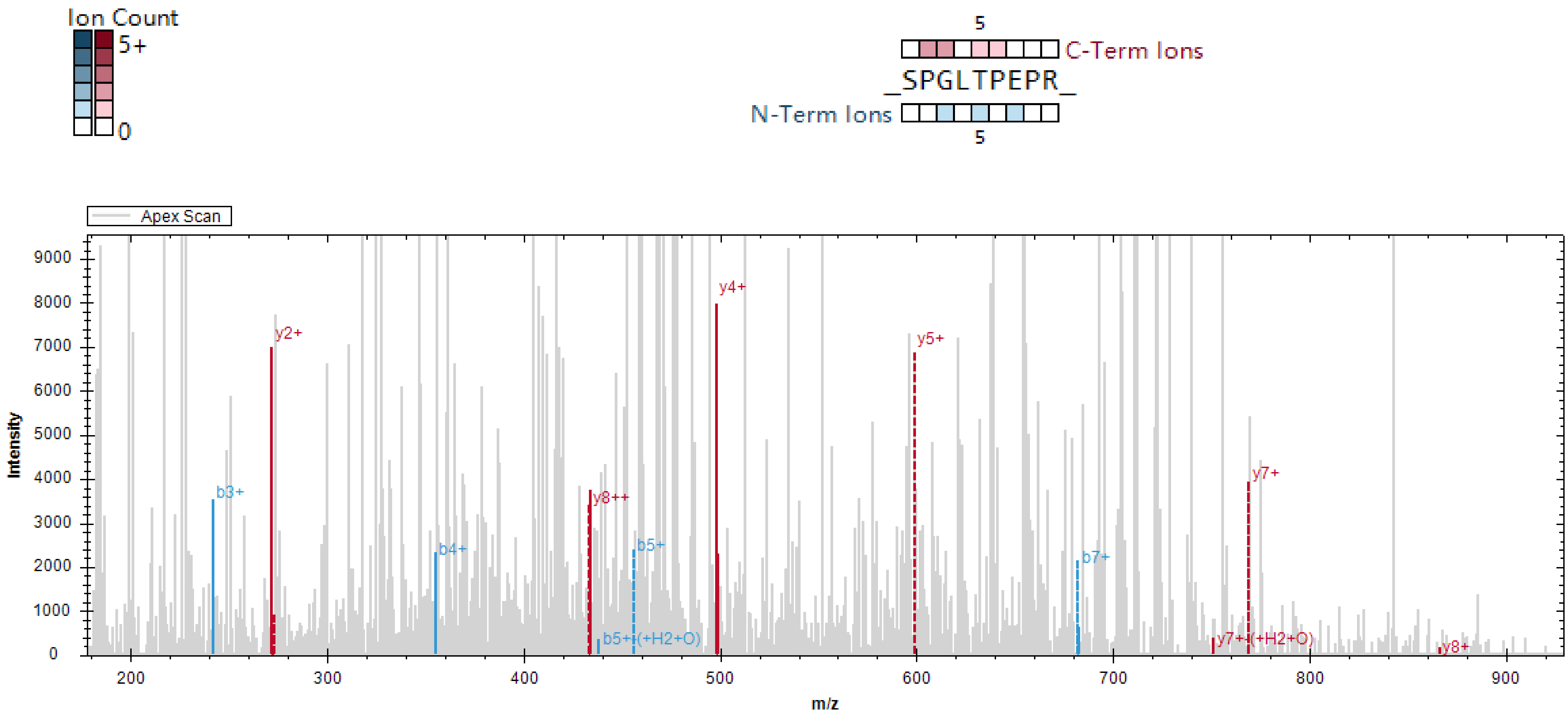

Ion Count
5+
0
5
C-Term Ions
_SPGLTPEPR_
N-Term Ions
5
Apex Scan
Intensity
m/z
y2+
b3+
b4+
y8++
b5+
b5++(+H2+O)
y4+
y5+
b7+
y7+
y7++(+H2+O)
y8+

Q14526

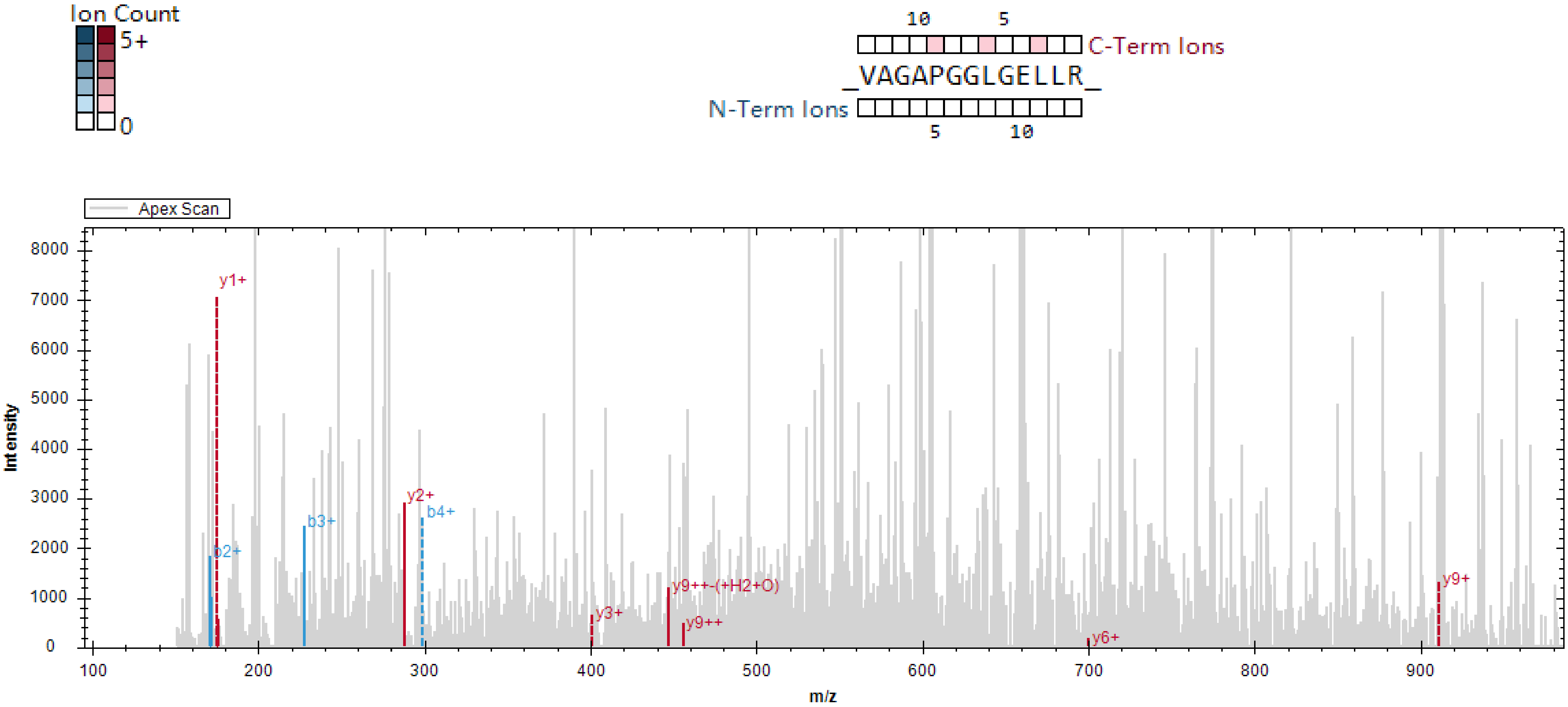

Ion Count
5+
0
10
5
C-Term Ions
_VAGAPGGLGELLR_
N-Term Ions
5
10
Apex Scan
Intensity
m/z
y1+
b2+
b3+
y2+
b4+
y3+
y9++-(+H2+O)
y9++
y6+
y9+

Q14919

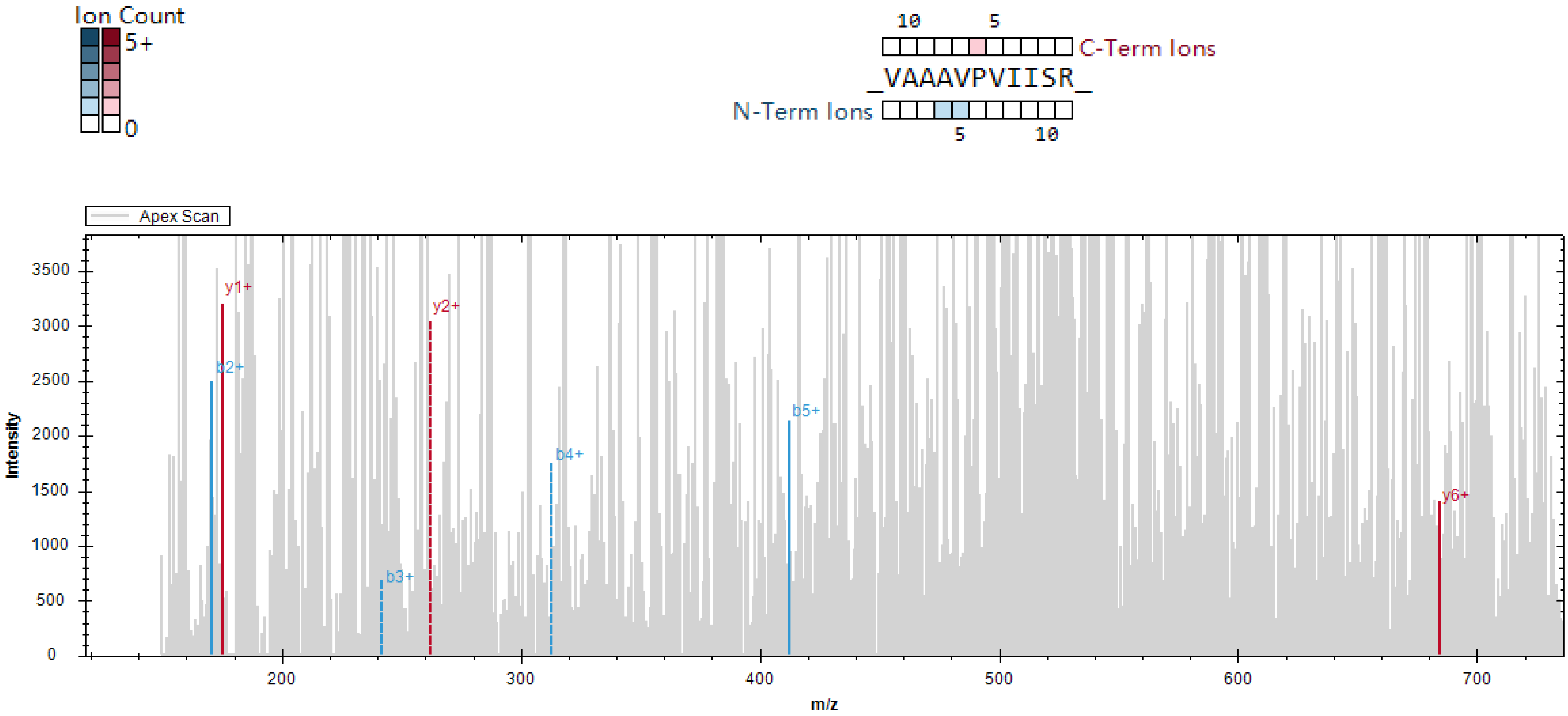

Ion Count
5+
0
10
5
C-Term Ions
_VAAAVPVIISR_
N-Term Ions
5
10
Apex Scan
Intensity
m/z
y1+
y2+
y6+
b2+
b3+
b4+
b5+

Q149N8

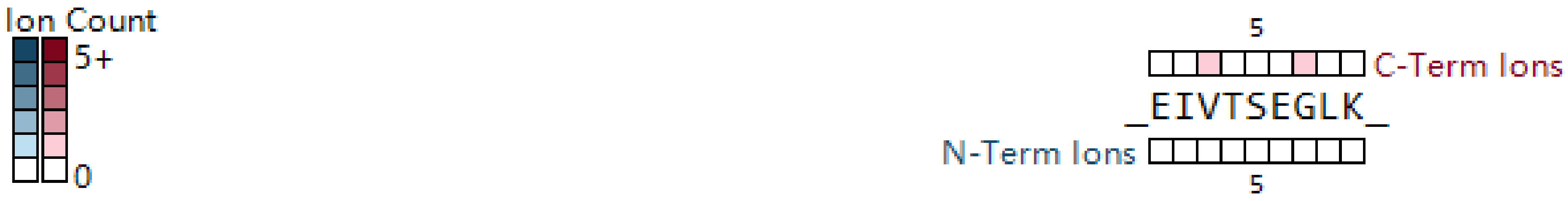
Ion Count
5+
0
5
C-Term Ions
_EIVTSEGLK_
N-Term Ions
5

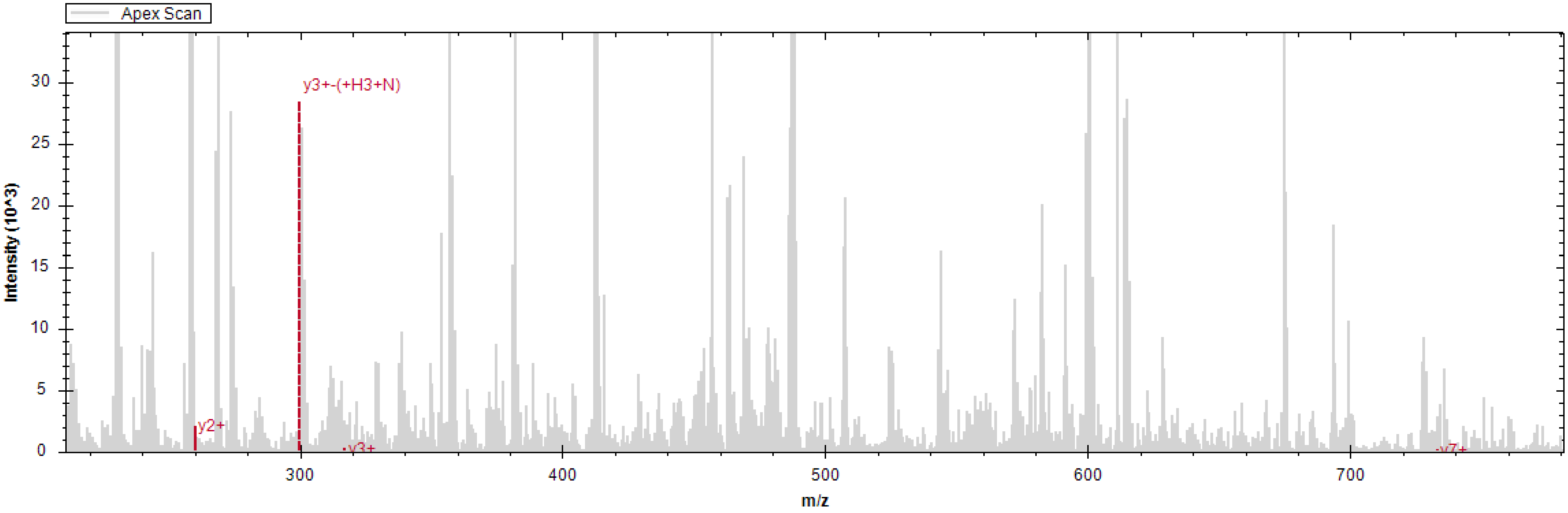
Apex Scan
y3+-(+H3+N)
y2+
y3+
Intensity (10^3)
30
25
20
15
10
5
0
300
400
500
600
700
m/z

Q15027

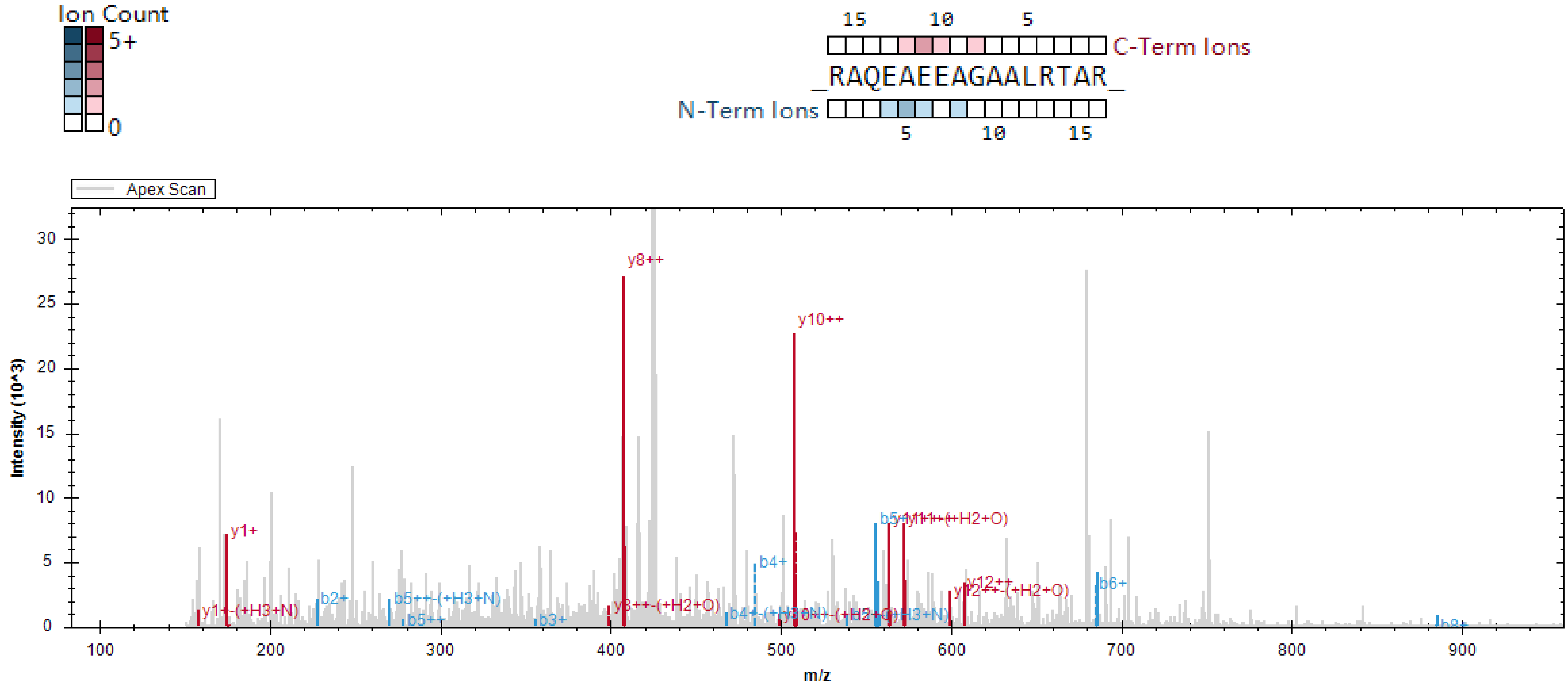
Ion Count
5+
0
15
10
5
C-Term Ions
_RAQEAEEAGAALRTAR_
N-Term Ions
5
10
15
Apex Scan
Intensity (10^3)
m/z
y8++
y10++
y1+
y1+-(+H3+N)
b2+
b5++-(+H3+N)
b5++
b3+
y3++-(+H2+O)
b4+
b6+
y12++
b8+

Q15131

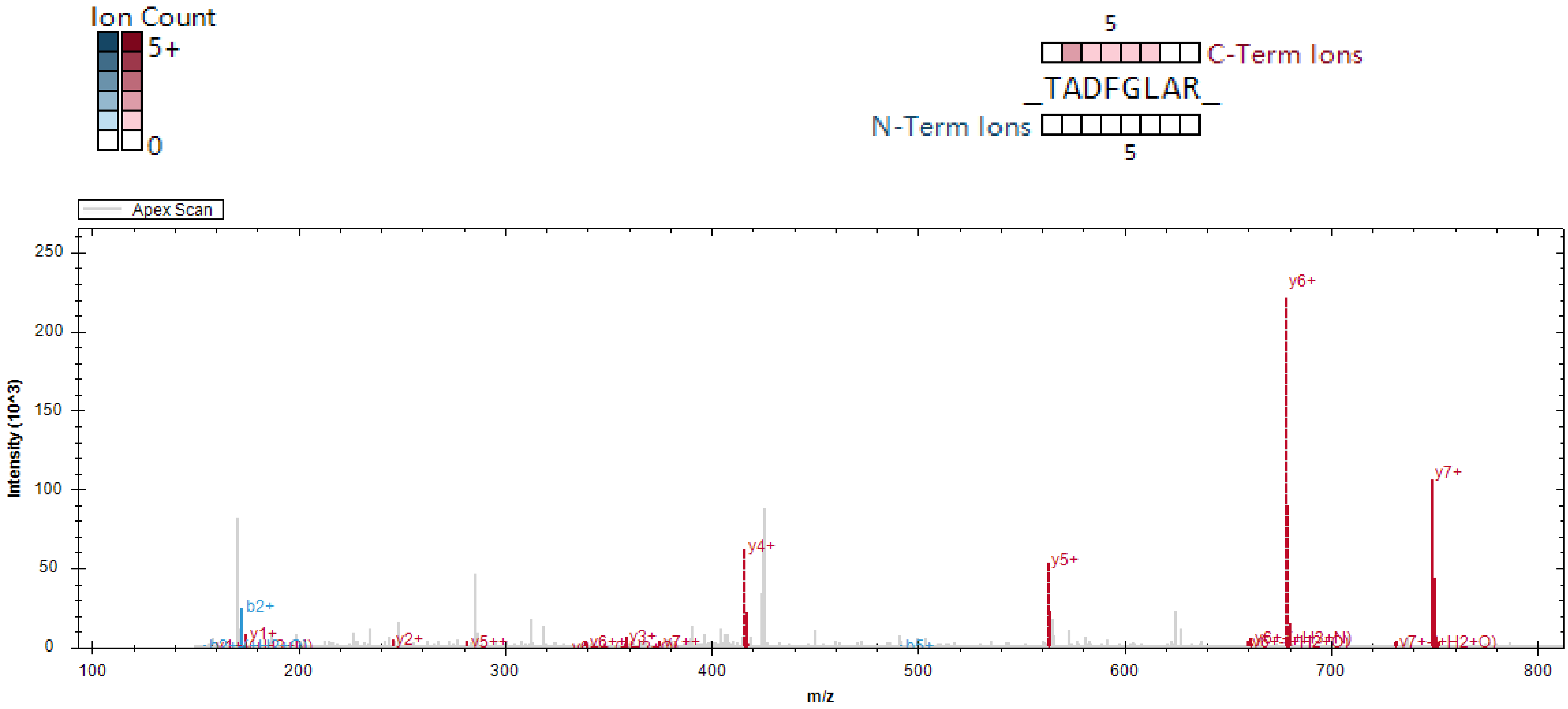

Ion Count
5+
0
5
C-Term Ions
_TADFGLAR_
N-Term Ions
5
Apex Scan
Intensity (10^3)
m/z
y6+
y7+
y4+
y5+
b2+
y1+
y2+

Q16222

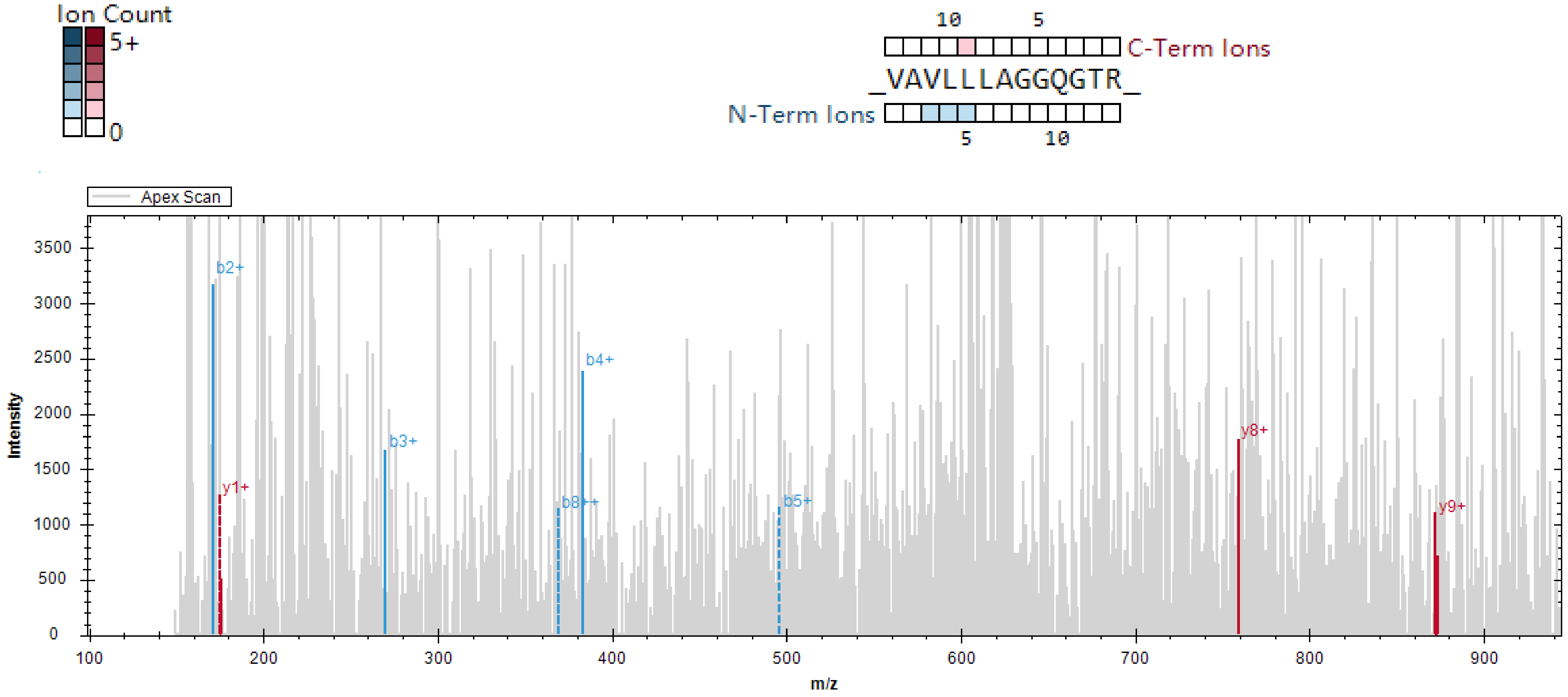

Ion Count
5+
0
10
5
C-Term Ions
_VAVLLLAGGQGTR_
N-Term Ions
5
10
Apex Scan
b2+
y1+
b3+
b8++
b4+
b5+
y8+
y9+
Intensity
0
500
1000
1500
2000
2500
3000
3500
100
200
300
400
500
600
700
800
900
m/z

Q16378

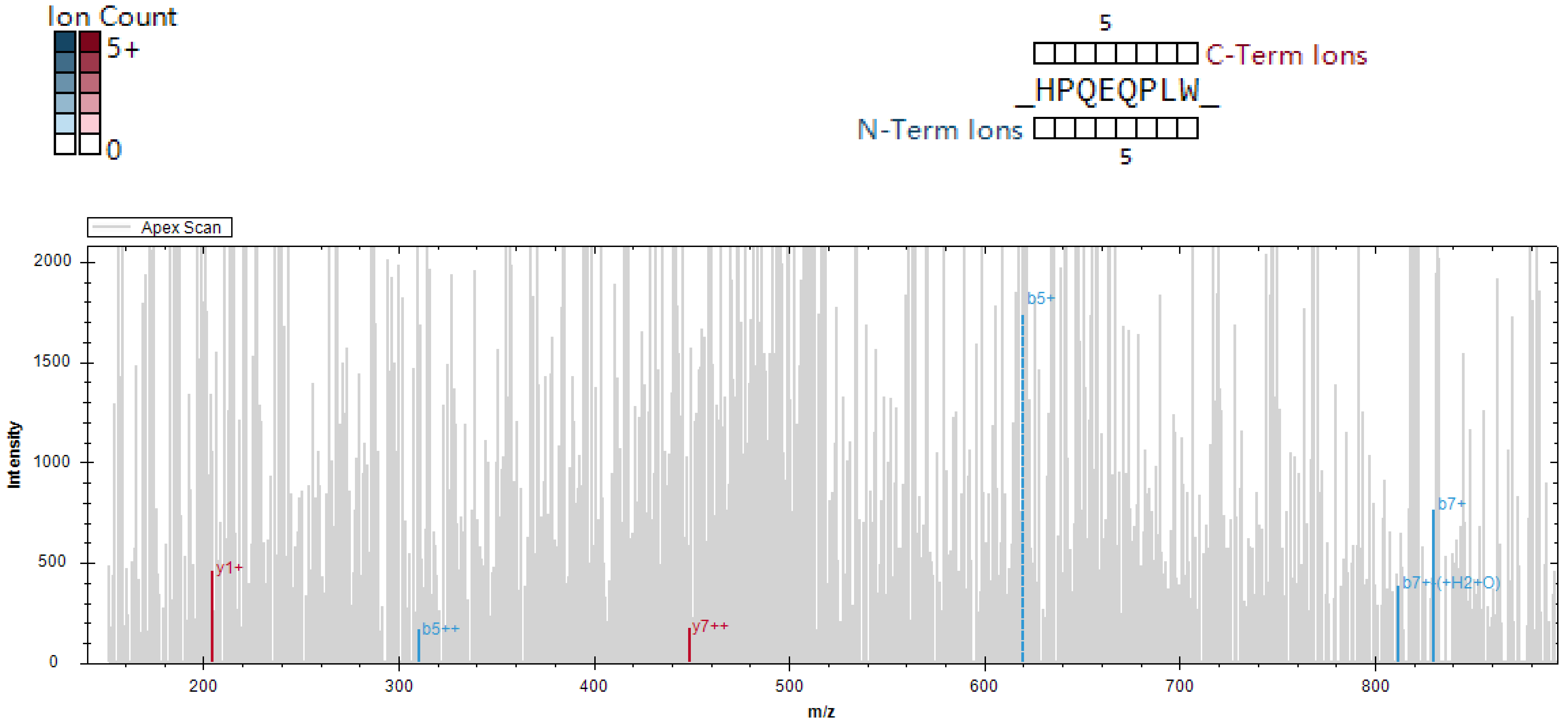

Ion Count
5+
0
5
C-Term Ions
_HPQEQPLW_
N-Term Ions
5
Apex Scan
Intensity
m/z
y1+
b5++
y7++
b5+
b7++(+H2+O)
b7+

Q16566

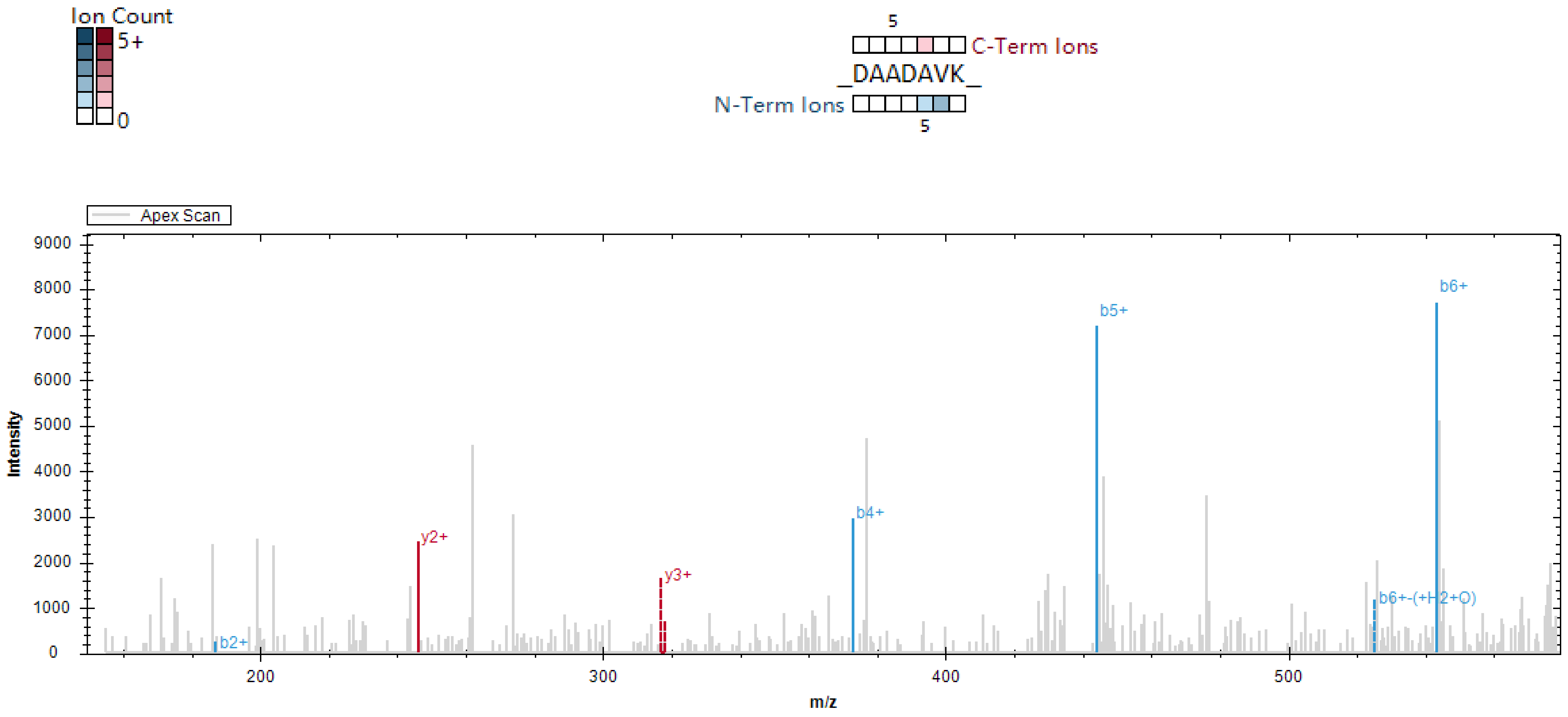

Ion Count
5+
0
5
C-Term Ions
_DAADAVK_
N-Term Ions
5
Apex Scan
Intensity
m/z
9000
8000
7000
6000
5000
4000
3000
2000
1000
0
200
300
400
500
b2+
y2+
y3+
b4+
b5+
b6+
b6+-(+H2+O)

# Q16570

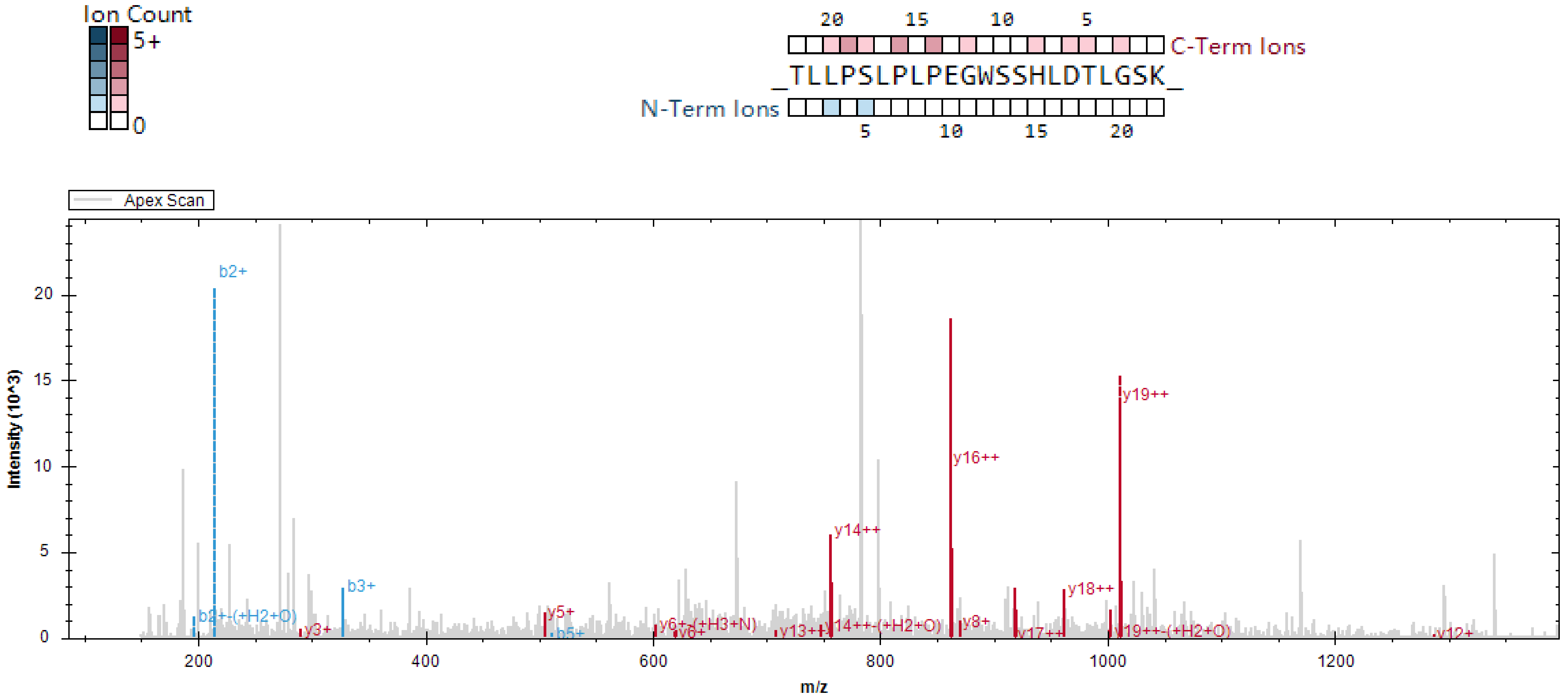
Ion Count
5+
0
20 15 10 5
C-Term Ions
_TLLPSLPLPEGWSSHLDTLGSK_
N-Term Ions
5 10 15 20
Apex Scan
b2+
b2+-(+H2+O)
b3+
b5+
y3+
y5+
y6+-(+H3+N)
y6+
y13++
y14++
y14++-(+H2+O)
y16++
y8+
y17++
y18++
y19++
y19++-(+H2+O)
y12+
Intensity (10^3)
m/z
0
5
10
15
20
200
400
600
800
1000
1200

Q16609

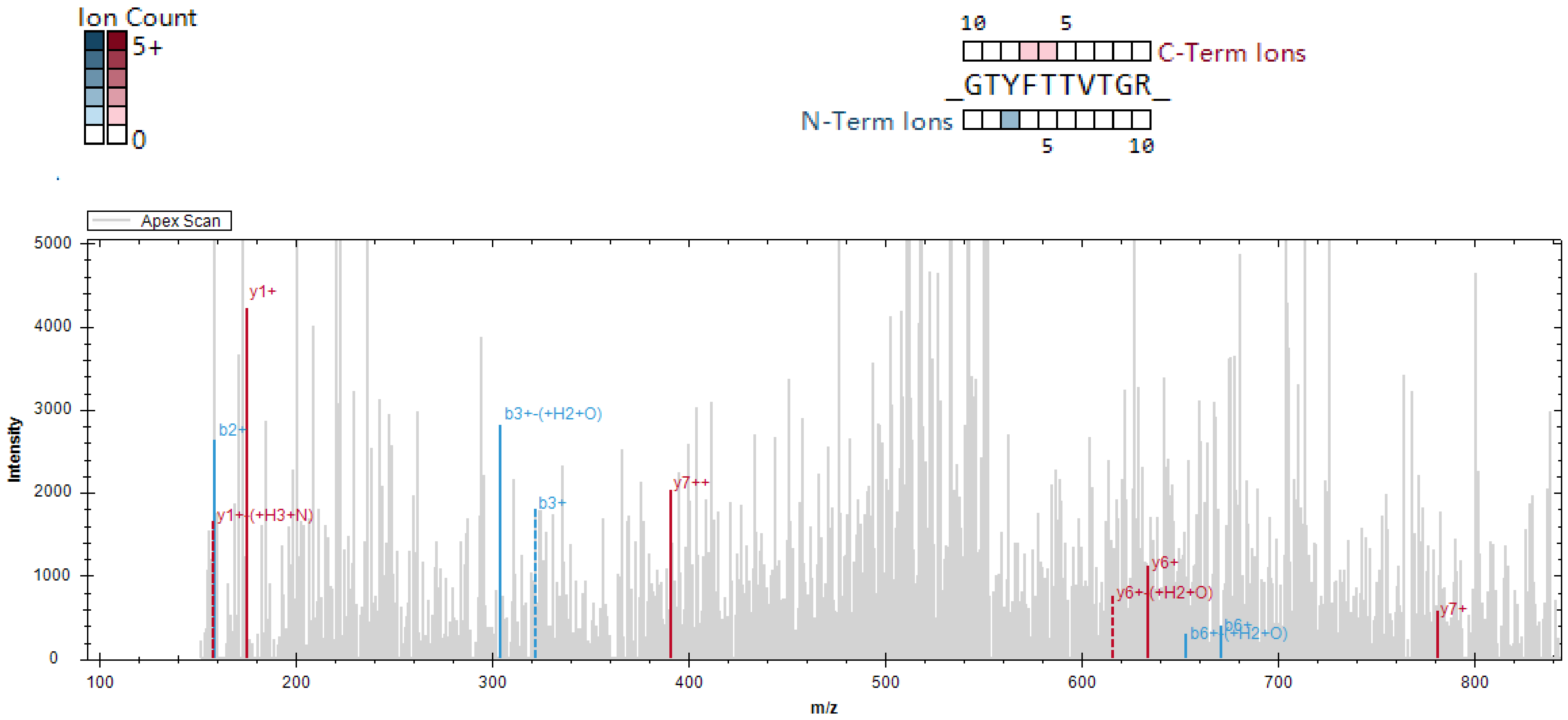

Ion Count
5+
0
10
5
C-Term Ions
_GTYFTTVTGR_
N-Term Ions
5
10
Apex Scan
Intensity
m/z
y1+
b2+
y1+-(+H3+N)
b3+-(+H2+O)
b3+
y7++
y6+
y6+-(+H2+O)
b6+
b6+-(+H2+O)
y7+

Q16825

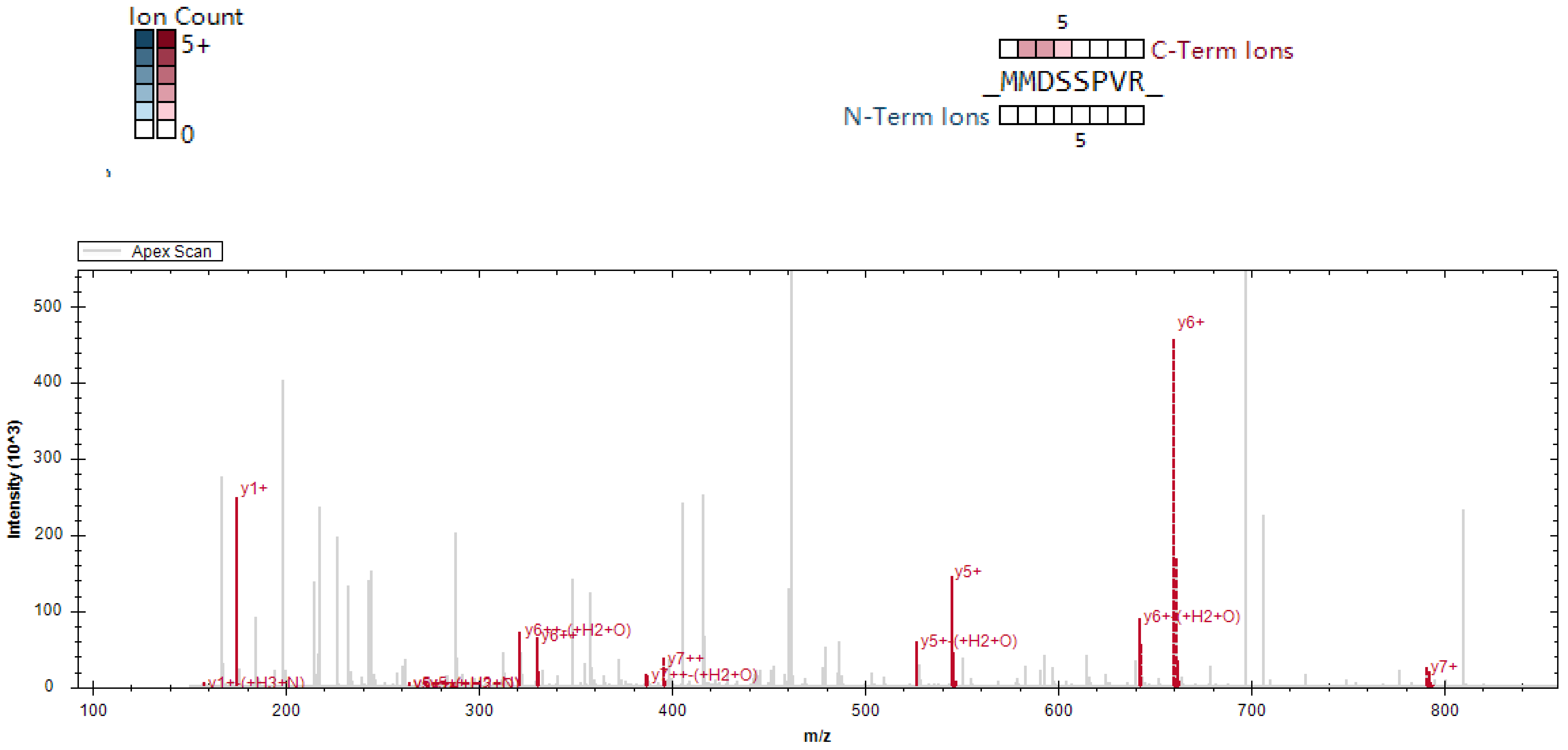

Ion Count
5+
0
5
C-Term Ions
_MMDSSPVR_
N-Term Ions
5
Apex Scan
Intensity (10^3)
m/z
y1+
y5+
y6+
y7+
y5+-(+H2+O)
y6+-(+H2+O)
y6++-(+H2+O)
y7++
y7++-(+H2+O)

Q2KHR3

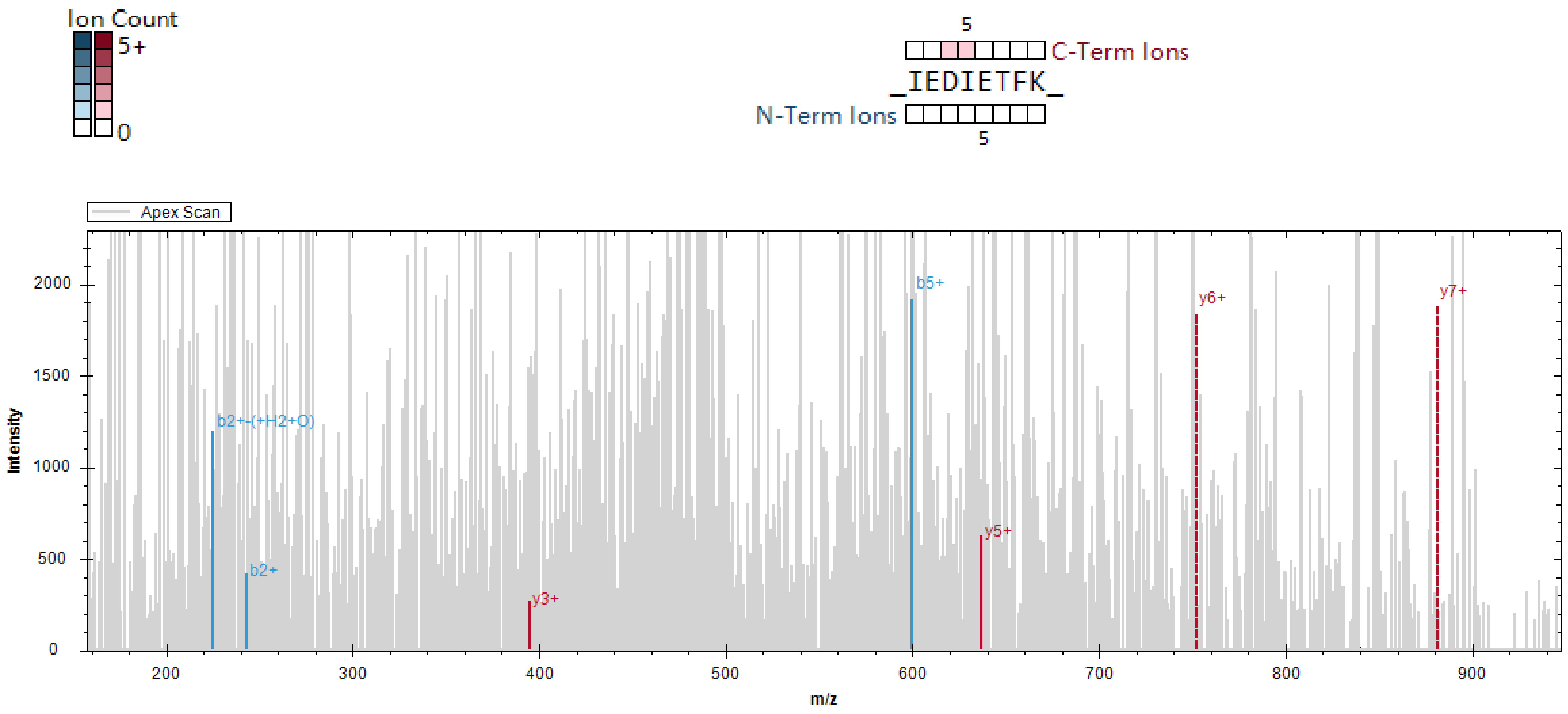

Ion Count
5+
0
5
C-Term Ions
_IEDIETFK_
N-Term Ions
5
Apex Scan
Intensity
m/z
b2+-(+H2+O)
b2+
y3+
b5+
y5+
y6+
y7+
0
500
1000
1500
2000
200
300
400
500
600
700
800
900

# Q2NL67

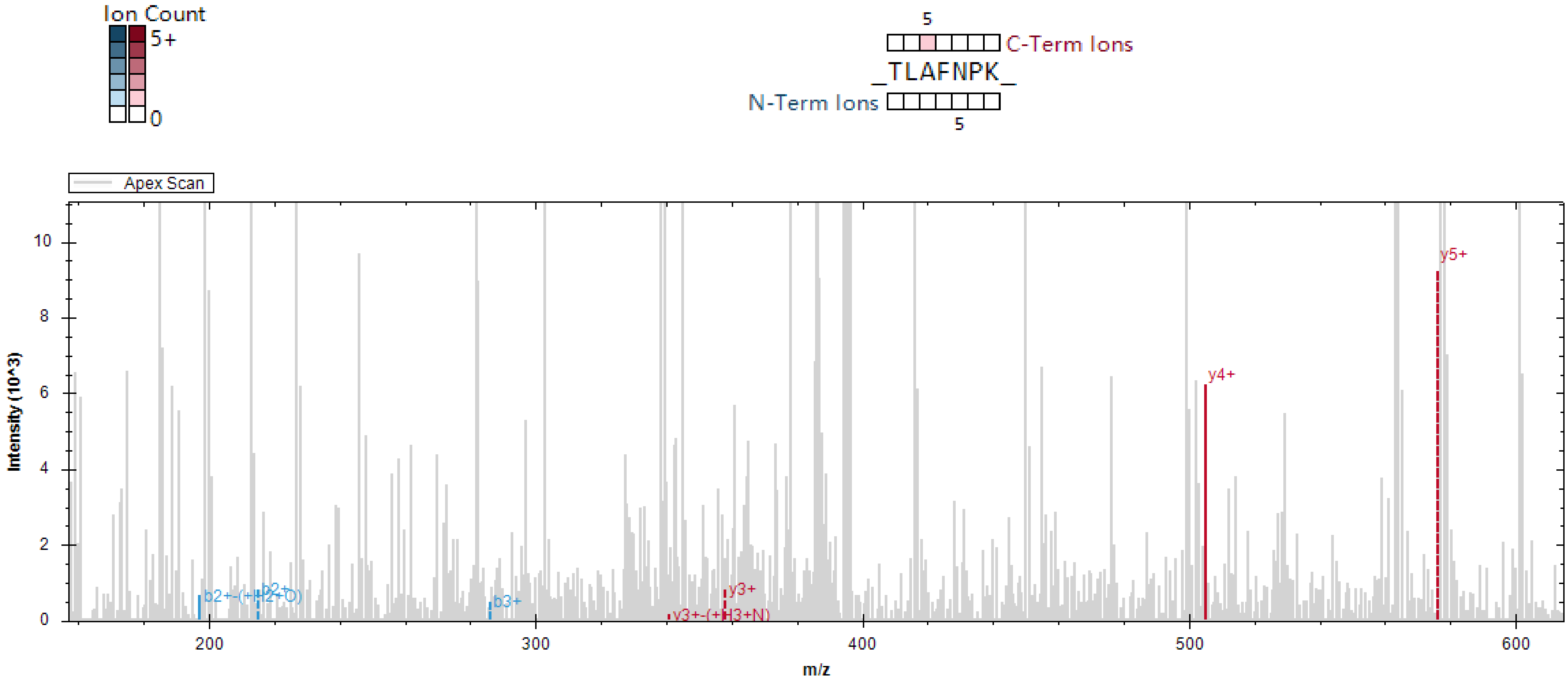

Ion Count
5+
0
5
C-Term Ions
_TLAFNPK_
N-Term Ions
5
Apex Scan
Intensity (10^3)
m/z
y5+
y4+
y3+
b3+

# Q4KWH8

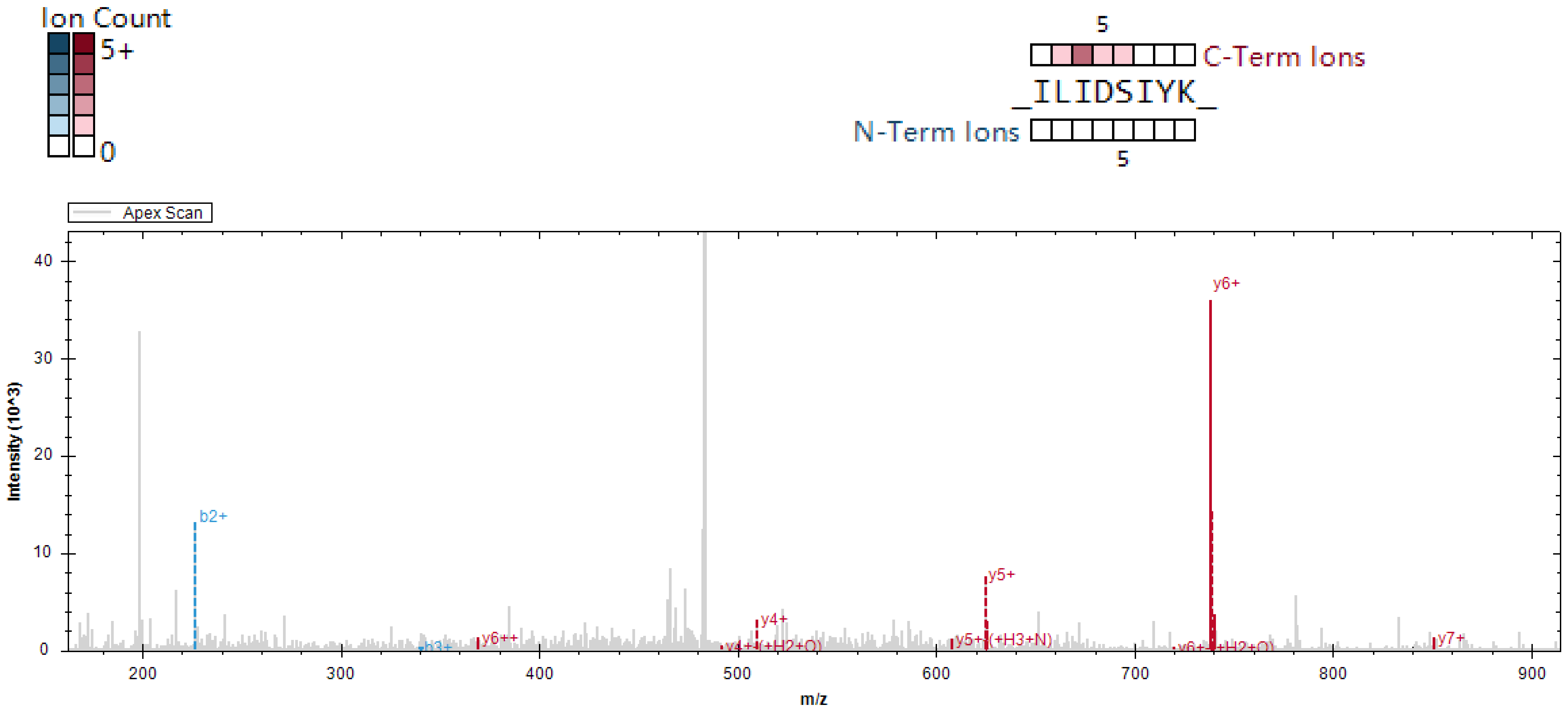

Ion Count
5+
0
5
C-Term Ions
_ILIDSIYK_
N-Term Ions
5
Apex Scan
Intensity (10^3)
m/z
y6+
b2+
y5+
y4+
y6++
y7+

Q5HYI8

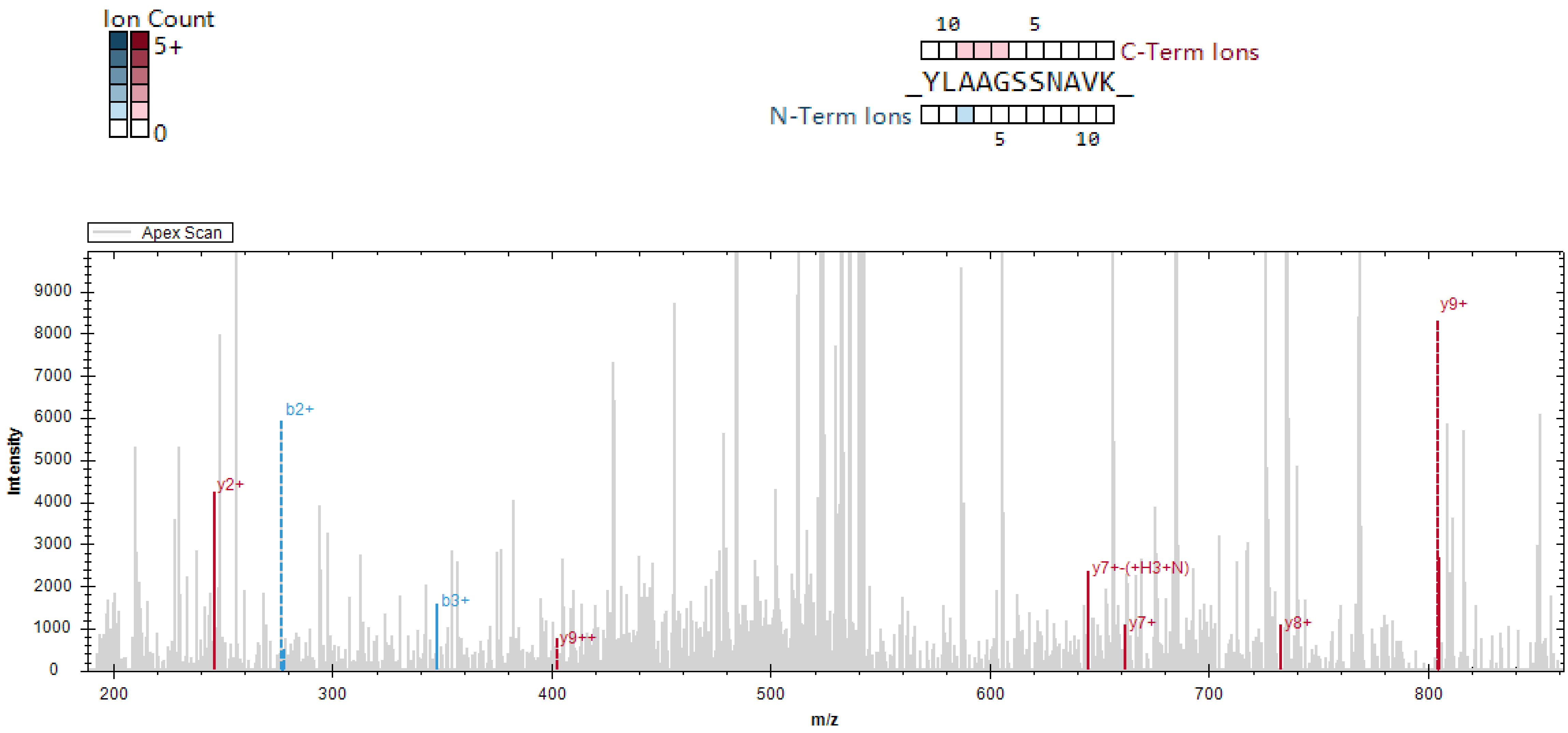

Ion Count
5+
0
10
5
C-Term Ions
_YLAAGSSNAVK_
N-Term Ions
5
10
Apex Scan
Intensity
m/z
y2+
b2+
b3+
y9++
y7+-(+H3+N)
y7+
y8+
y9+

# Q5JPF3

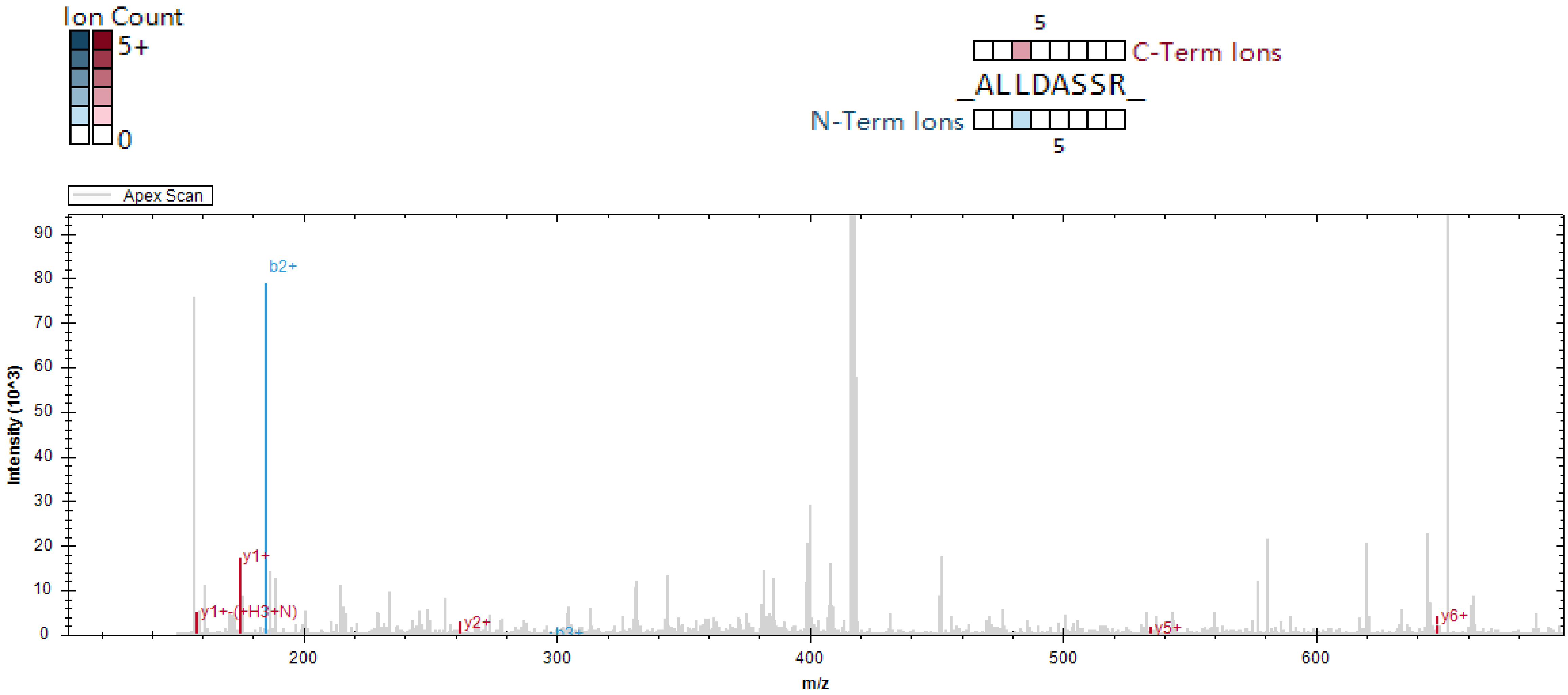

Ion Count
5+
0
5
C-Term Ions
_ALLDASSR_
N-Term Ions
5
Apex Scan
Intensity (10^3)
m/z
b2+
y1+
y1+-(+H3+N)
y2+
y5+
y6+

Q5T3U5

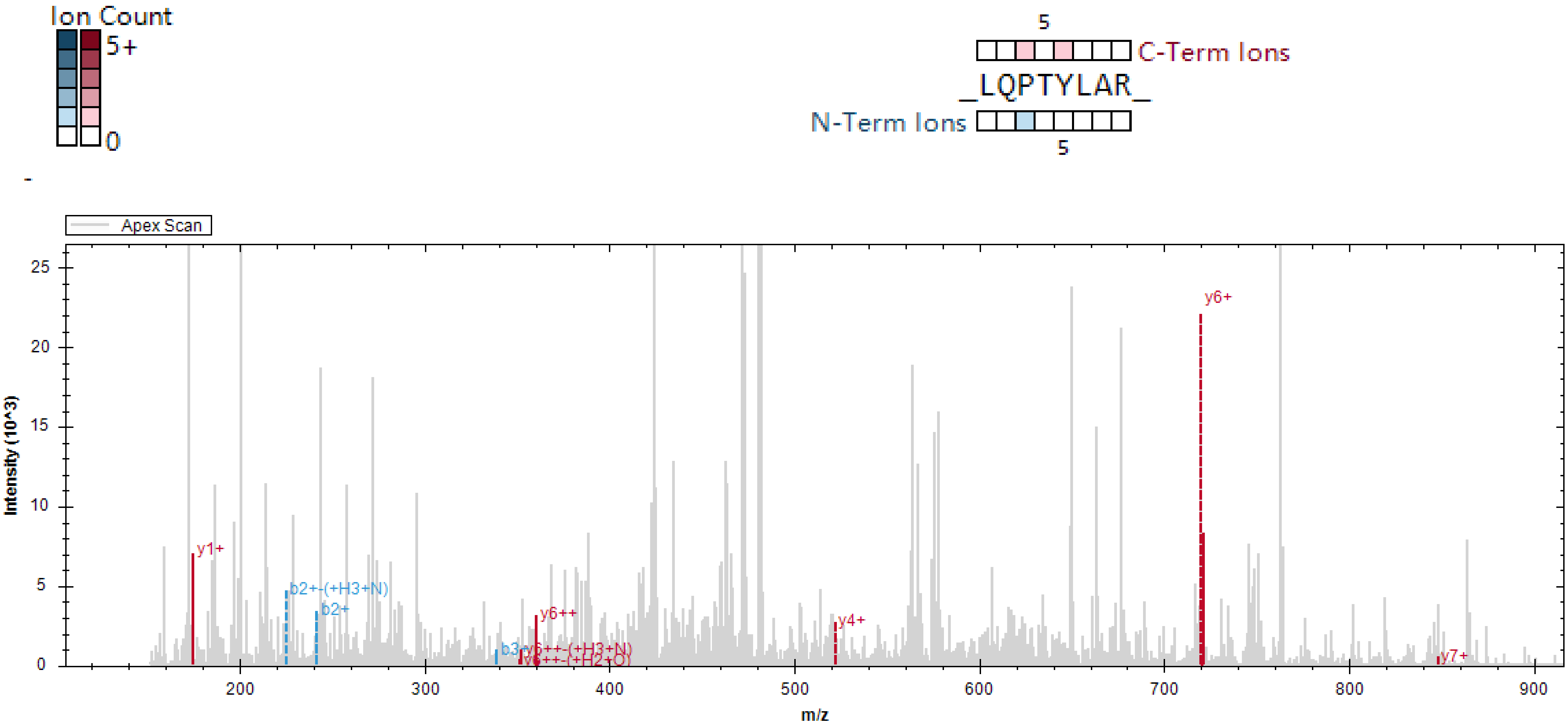

Ion Count
5+
0
5
C-Term Ions
_LQPTYLAR_
N-Term Ions
5
Apex Scan
Intensity (10^3)
m/z
y1+
b2+-(+H3+N)
b2+
b3+
y6++
y6++-(+H3+N)
y6++-(+H2+O)
y4+
y6+
y7+

Q5T4F4

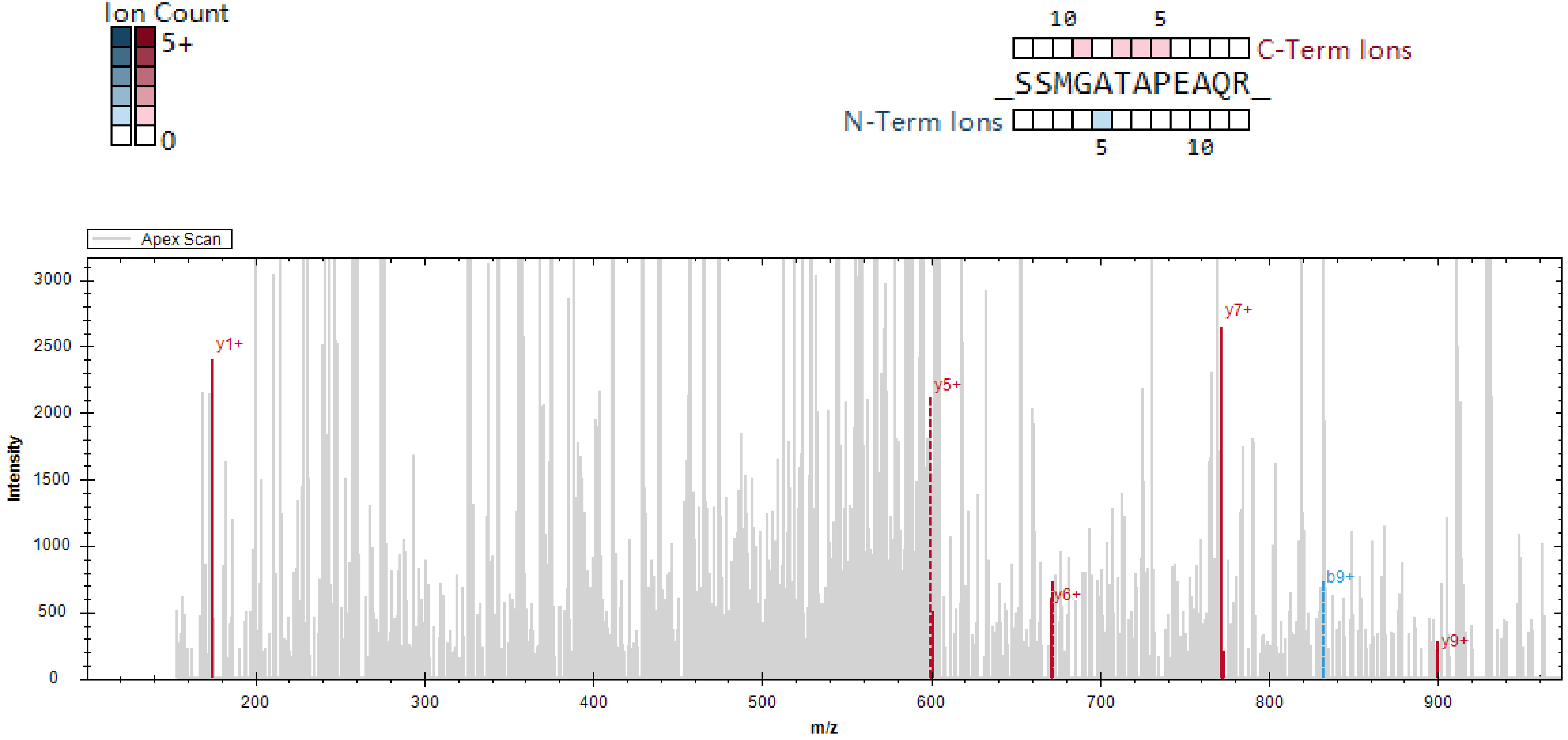
Ion Count
5+
0
10
5
C-Term Ions
_SSMGATAPEAQR_
N-Term Ions
5
10
Apex Scan
y1+
y5+
y6+
y7+
b9+
y9+
Intensity
m/z
0
500
1000
1500
2000
2500
3000
200
300
400
500
600
700
800
900

Q5T5S1

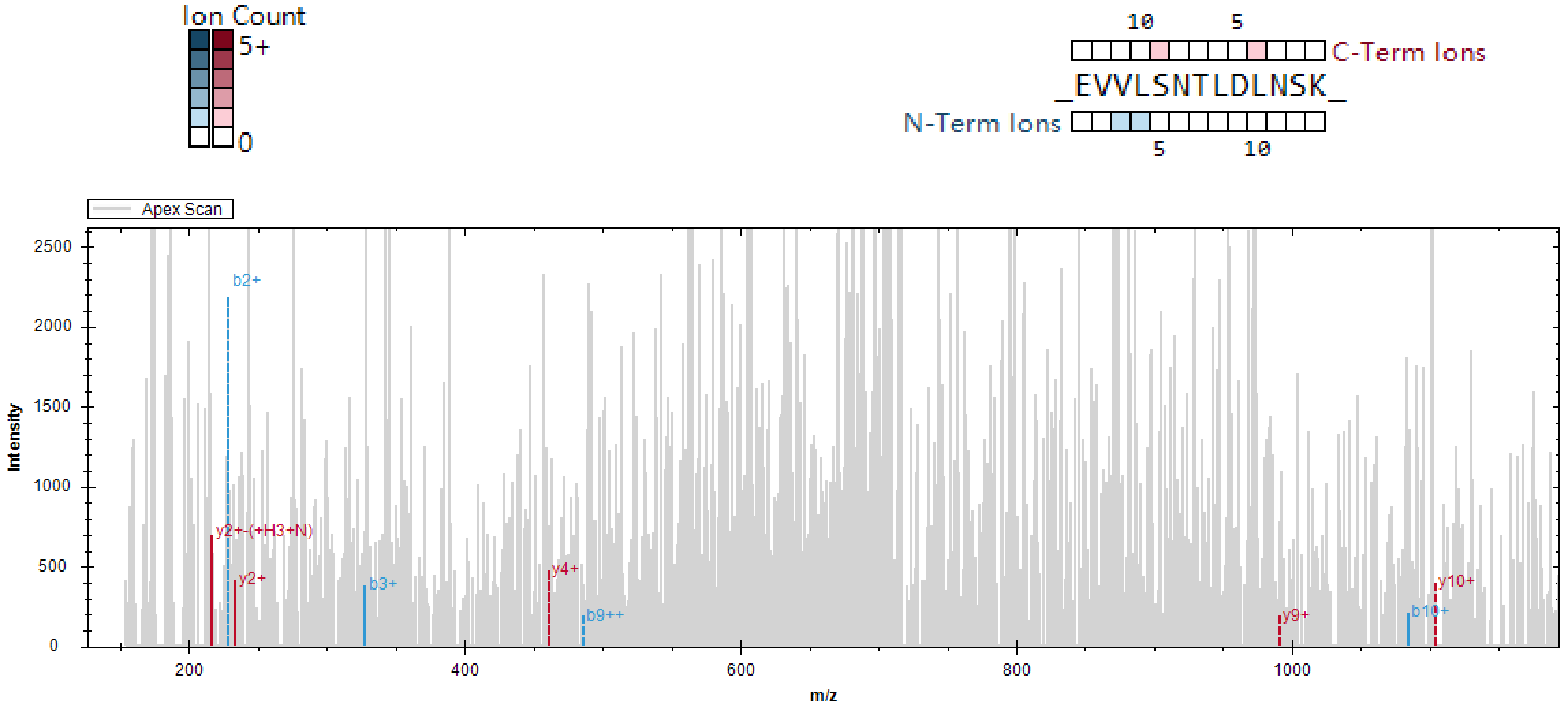

Ion Count
5+
0
10
5
C-Term Ions
_EVVLSNTLDLNSK_
N-Term Ions
5
10
Apex Scan
Intensity
m/z
b2+
y2+-(+H3+N)
y2+
b3+
y4+
b9++
y9+
b10+
y10+

Q5T9A4

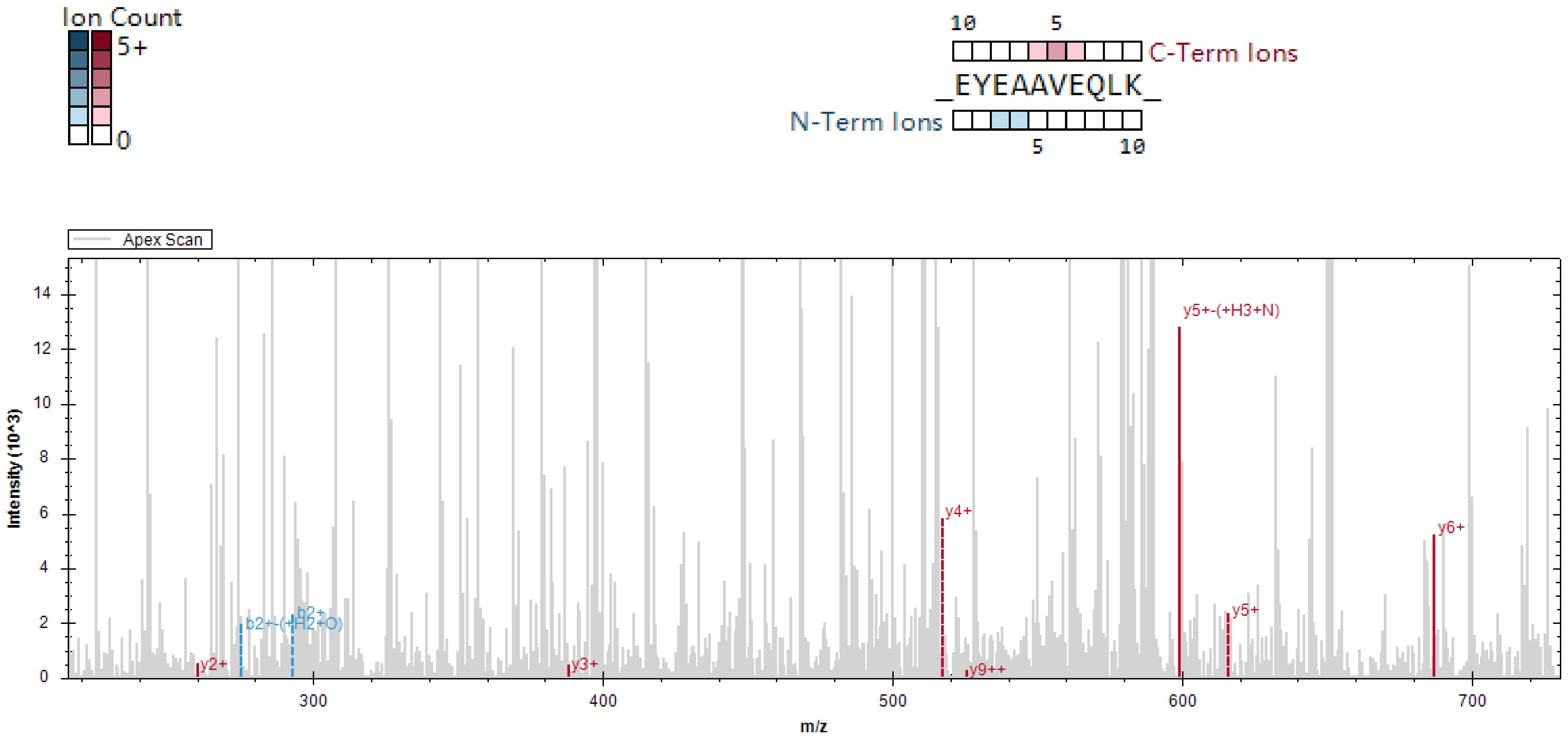

Ion Count
5+
0
10
5
C-Term Ions
_EYEAAVEQLK_
N-Term Ions
5
10
Apex Scan
Intensity (10^3)
m/z
y5+-(+H3+N)
y4+
y6+
y5+
b2+-(+H2+O)
b2+
y2+
y3+
y9++

# Q5TFQ8

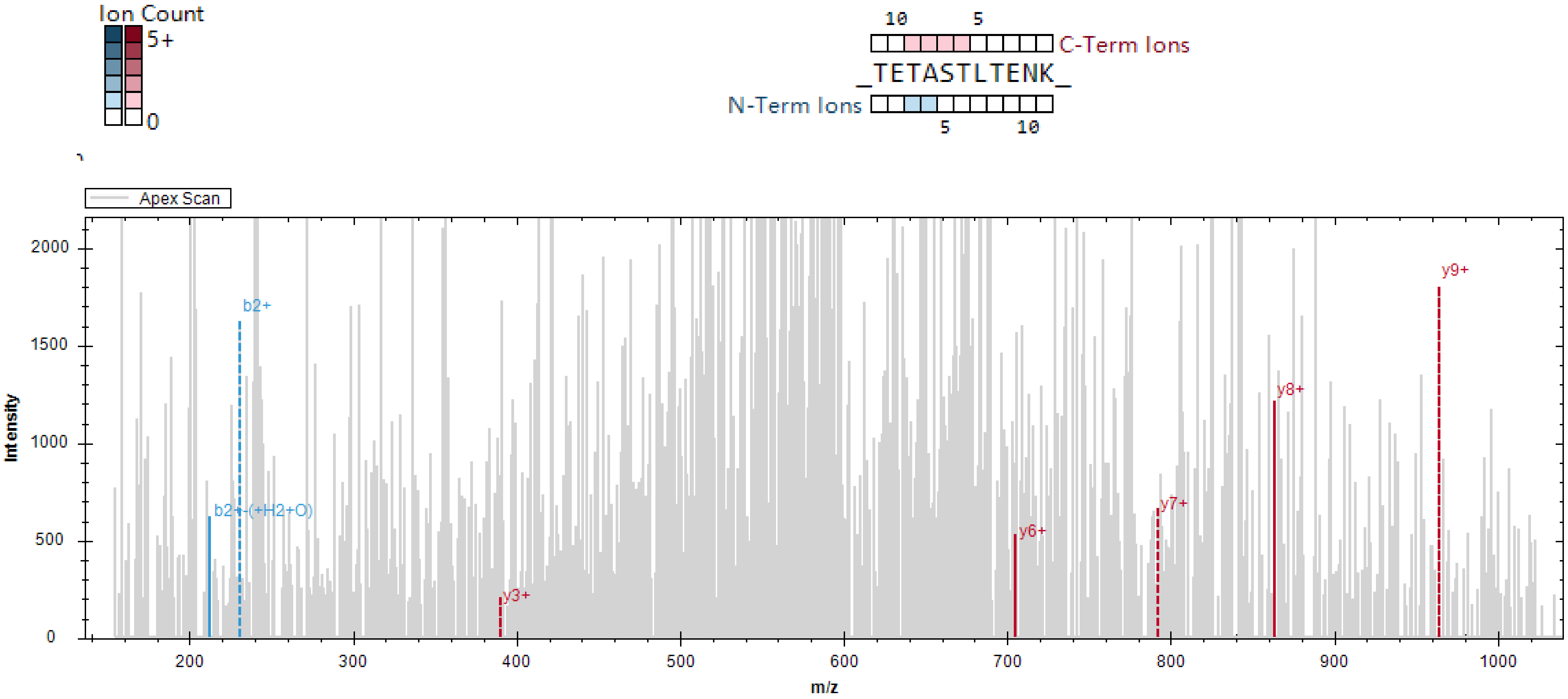

Ion Count
5+
0
10
5
C-Term Ions
_TETASTLTENK_
N-Term Ions
5
10
Apex Scan
Intensity
m/z
2000
1500
1000
500
0
200
300
400
500
600
700
800
900
1000
b2+
b2+-(+H2+O)
y3+
y6+
y7+
y8+
y9+

Q5VU43

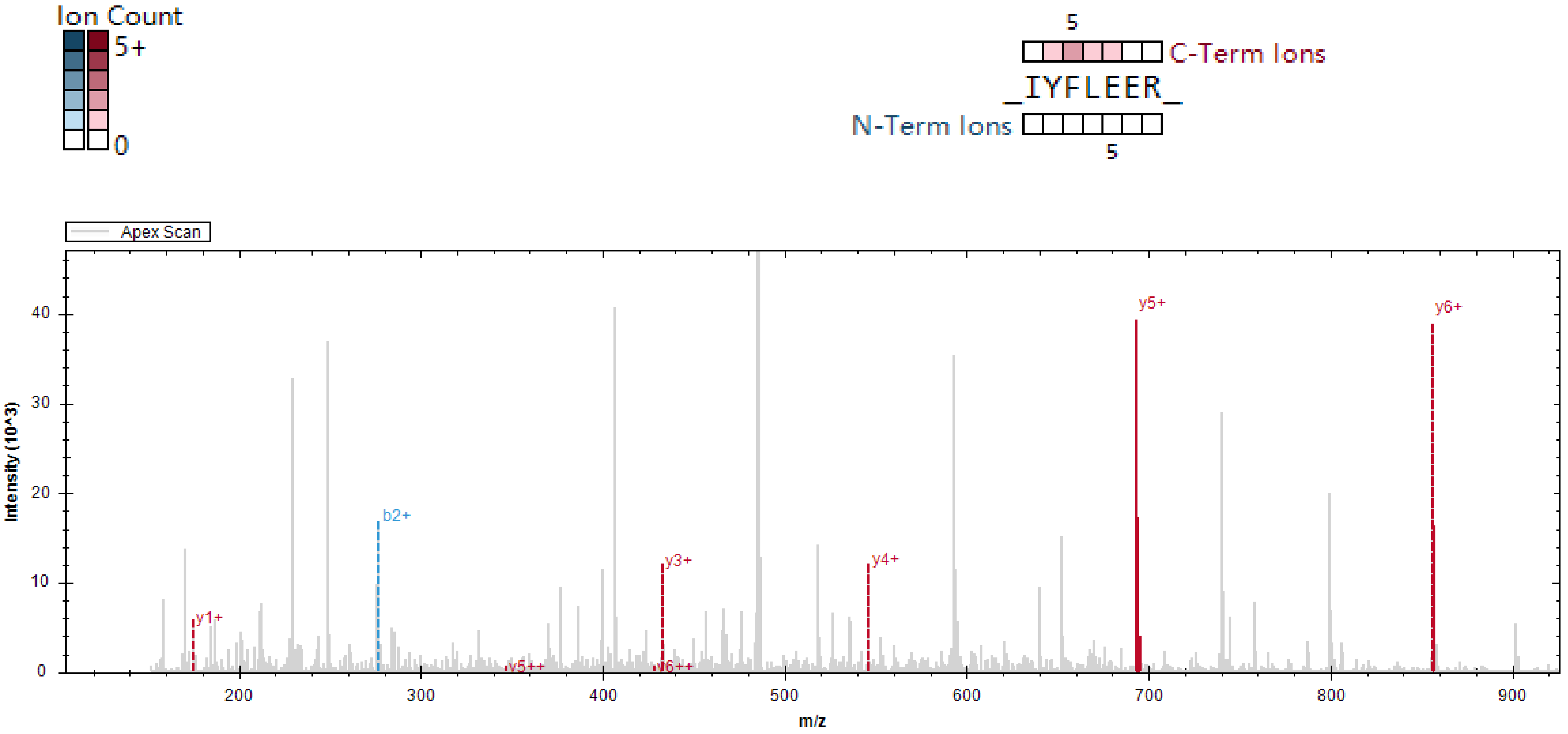
Ion Count
5+
0
5
C-Term Ions
_IYFLEER_
N-Term Ions
5
Apex Scan
Intensity (10^3)
m/z
y1+
b2+
y5++
y6++
y3+
y4+
y5+
y6+
0
10
20
30
40
200
300
400
500
600
700
800
900

Q60I27

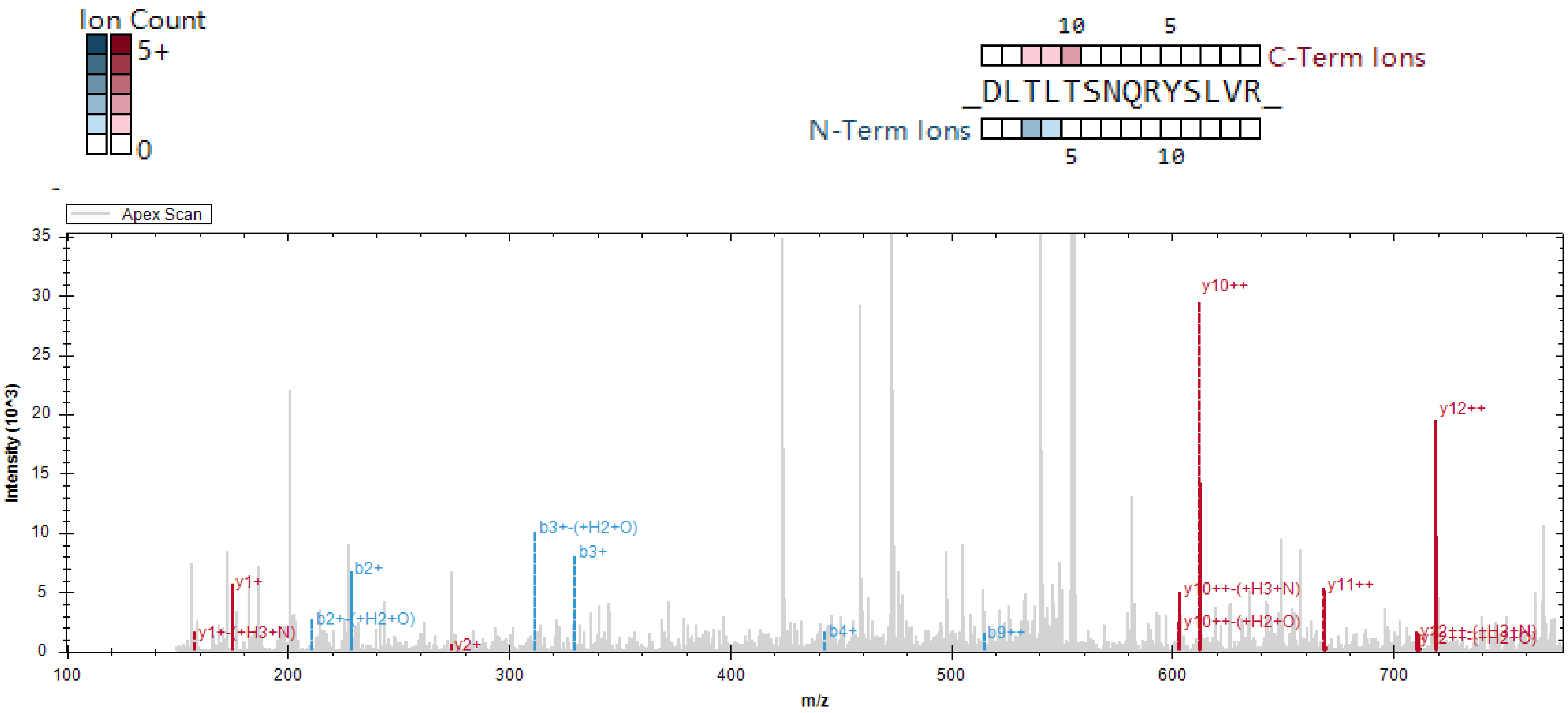

Ion Count
5+
0
10
5
C-Term Ions
_DLTLTSNQRYSLVR_
N-Term Ions
5
10
Apex Scan
Intensity (10^3)
m/z
y10++
y12++
b3+-(+H2+O)
b3+
b2+
y1+
b2+-(+H2+O)
y1+-(+H3+N)
y2+
b4+
b9++
y10++-(+H3+N)
y10++-(+H2+O)
y11++

Q629K1

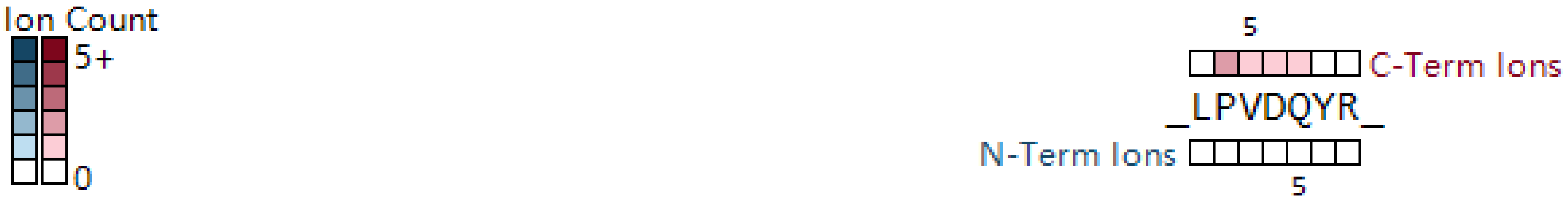

Ion Count
5+
0
5
C-Term Ions
_LPVDQYR_
N-Term Ions
5


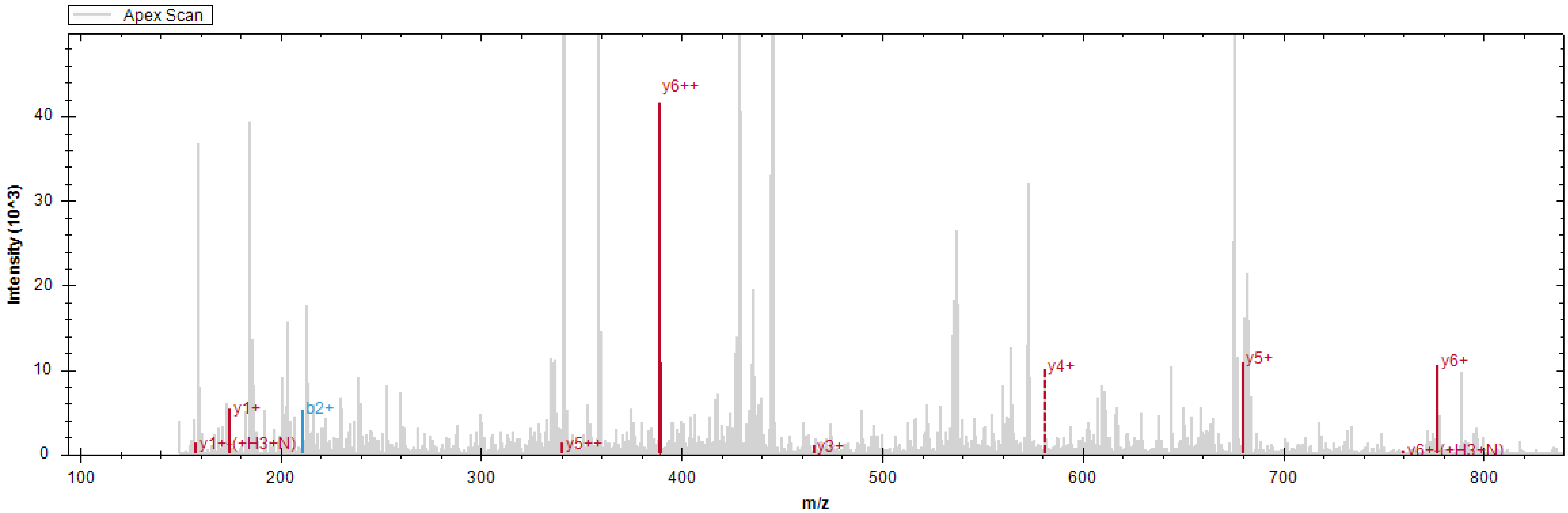

Apex Scan
Intensity (10^3)
0
10
20
30
40
y6++
y1+
b2+
y1+-(+H3+N)
y5++
y3+
y4+
y5+
y6+
y6+-(+H3+N)
100
200
300
400
500
600
700
800
m/z

Q687X5

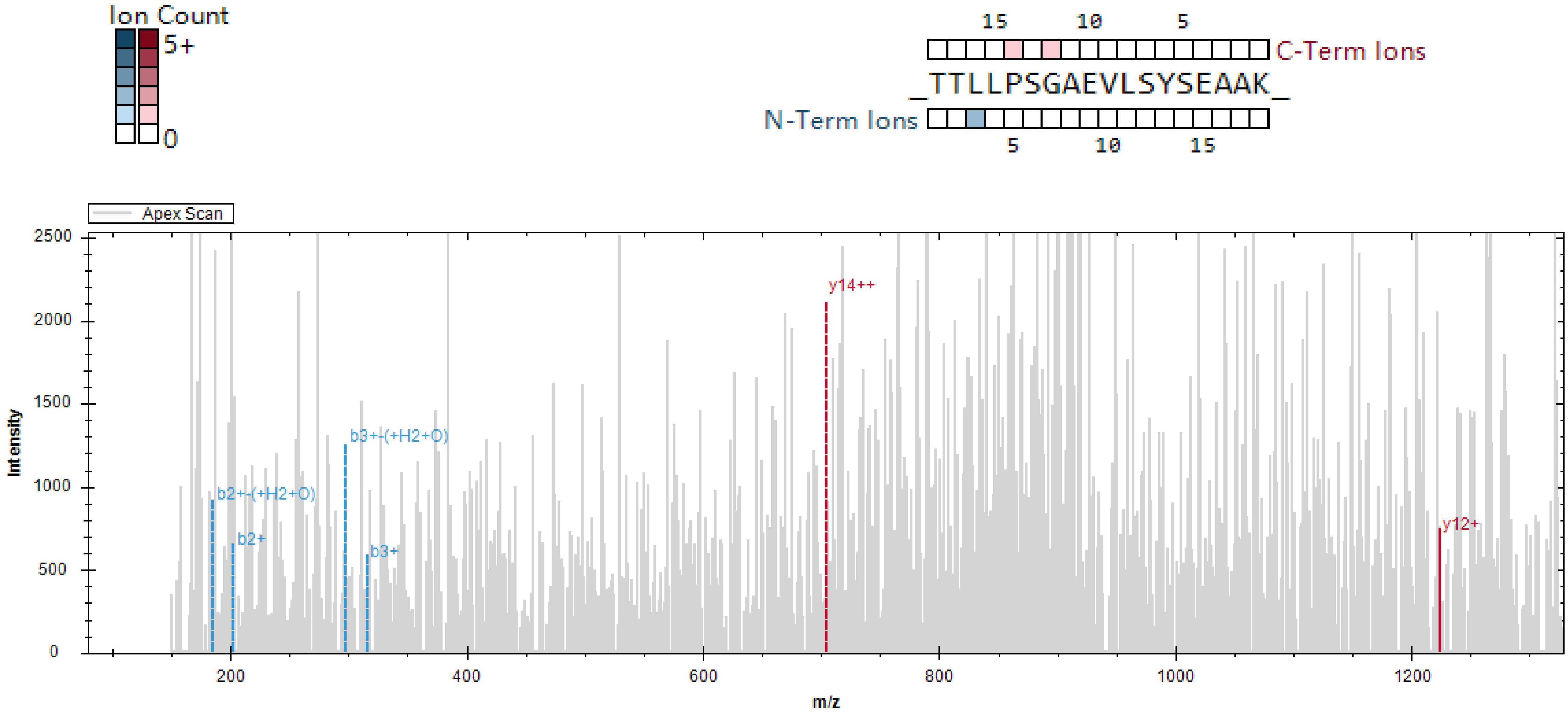

Ion Count
5+
0
15
10
5
C-Term Ions
_TTLLPSGAEVLSYSEAAK_
N-Term Ions
5
10
15
Apex Scan
2500
2000
1500
1000
500
0
Intensity
b2+-(+H2+O)
b2+
b3+-(+H2+O)
b3+
y14++
y12+
200
400
600
800
1000
1200
m/z

Q6DHV5

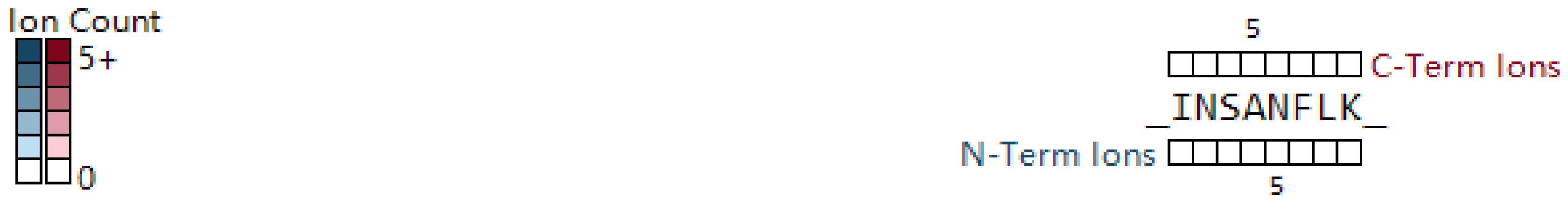

Ion Count
5+
0
5
C-Term Ions
_INSANFLK_
N-Term Ions
5


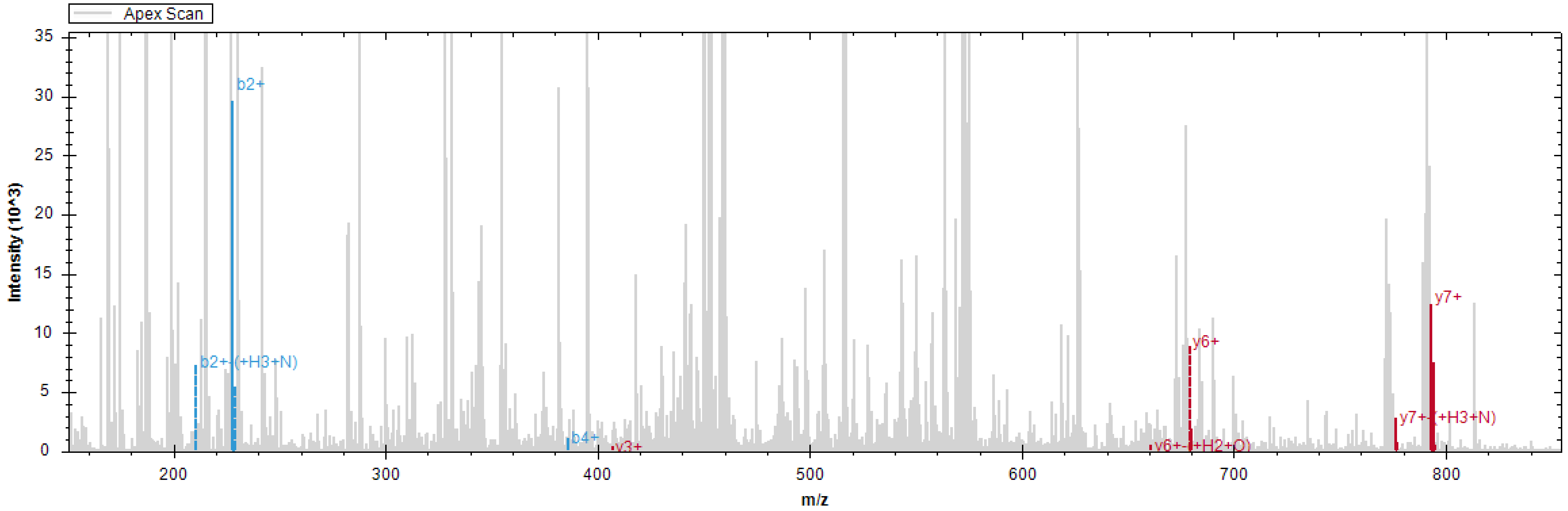

Apex Scan
Intensity (10^3)
m/z
b2+
b2+-(+H3+N)
b4+
y3+
y6+
y6+-(+H2+O)
y7+
y7+-(+H3+N)

# Q6I9Y2

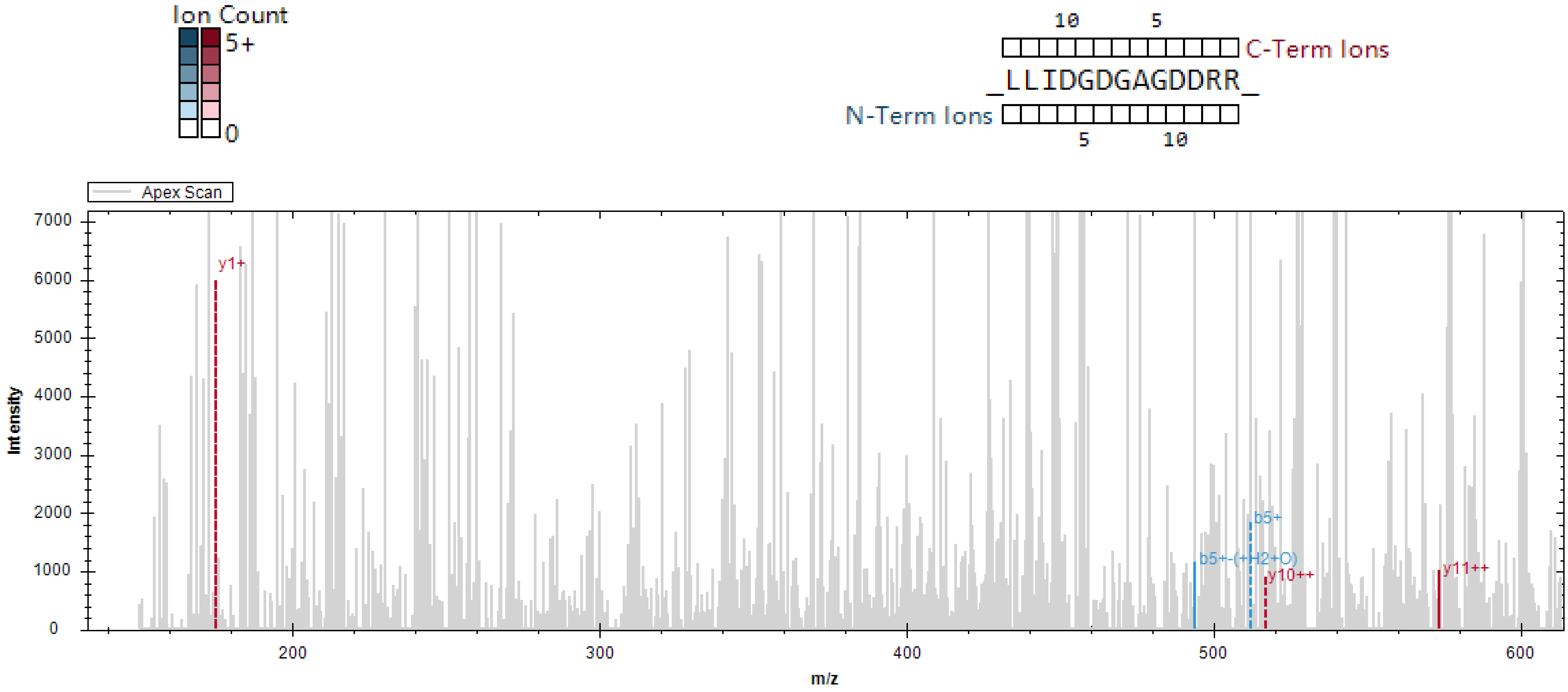

Ion Count
5+
0
10
5
C-Term Ions
_LLIDGDGAGDDRR_
N-Term Ions
5
10
Apex Scan
y1+
b5+
b5+-(+H2+O)
y10++
y11++
Intensity
m/z
0
1000
2000
3000
4000
5000
6000
7000
200
300
400
500
600

Q6NUM9

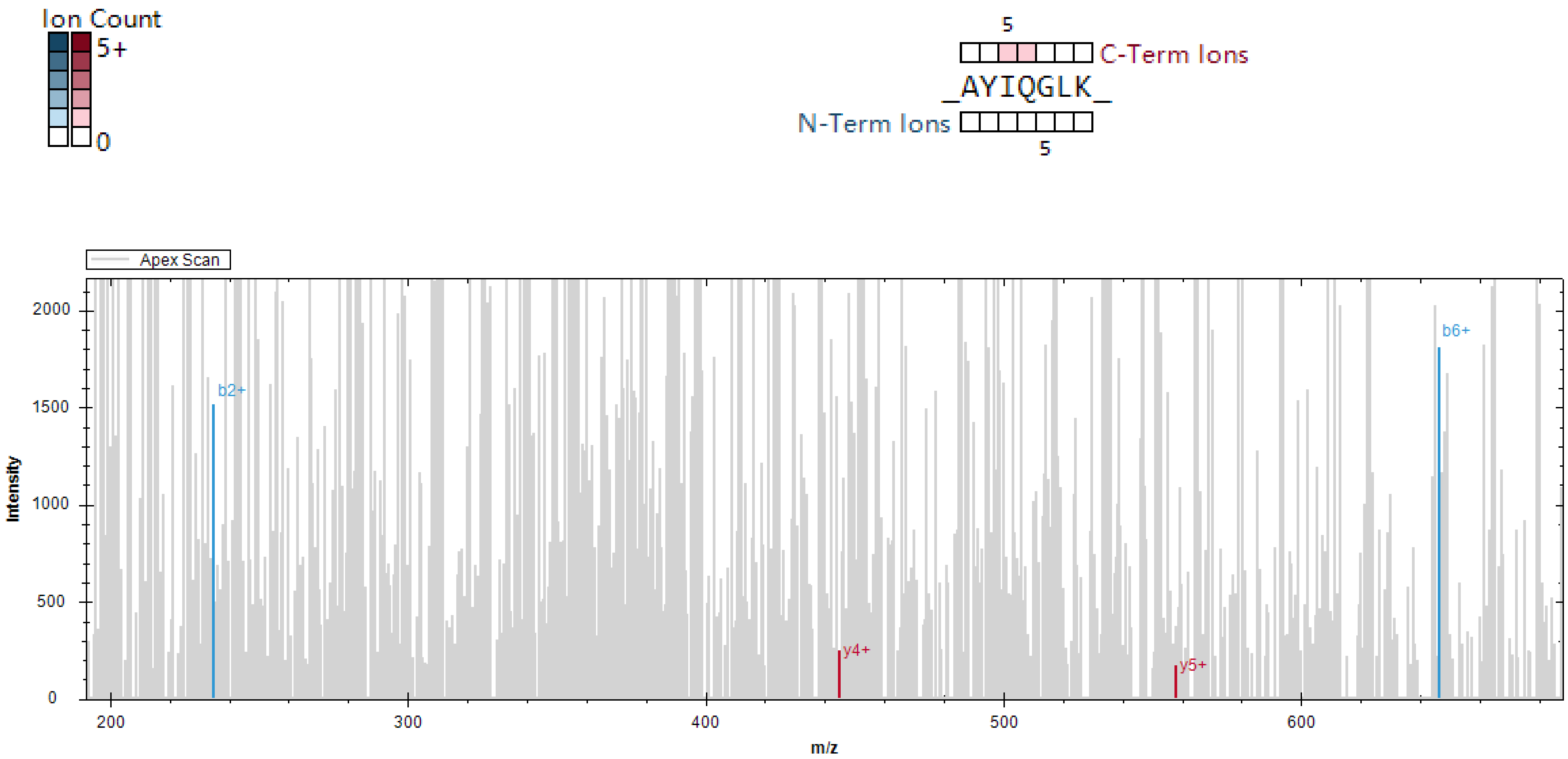
Ion Count
5+
0
5
C-Term Ions
_AYIQGLK_
N-Term Ions
5
Apex Scan
Intensity
2000
1500
1000
500
0
b2+
b6+
y4+
y5+
200
300
400
500
600
m/z

Q6QNY0

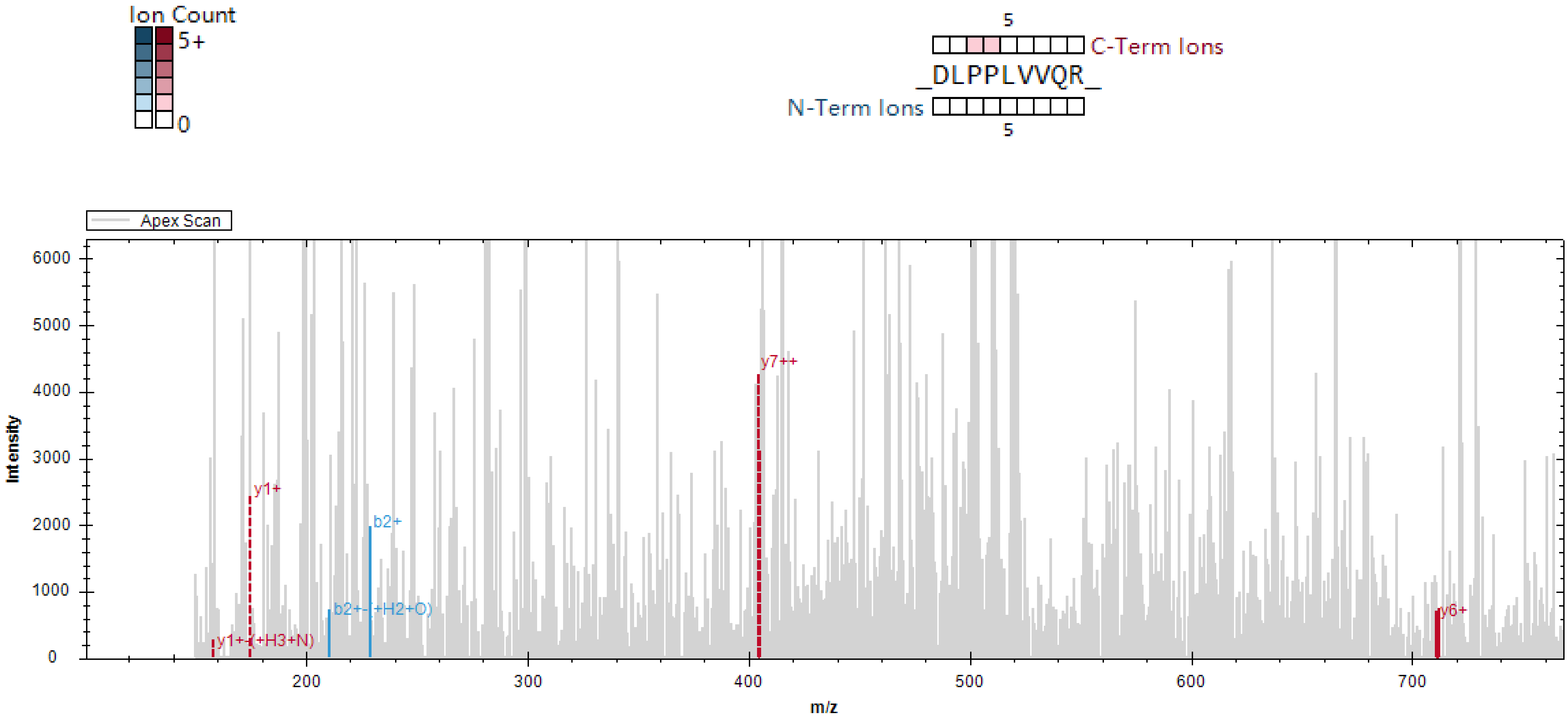
Ion Count
5+
0
5
C-Term Ions
_DLPPLVVQR_
N-Term Ions
5
Apex Scan
Intensity
m/z
y1+
y1+-(+H3+N)
b2+
b2+-(+H2+O)
y7++
y6+

Q6UXH1

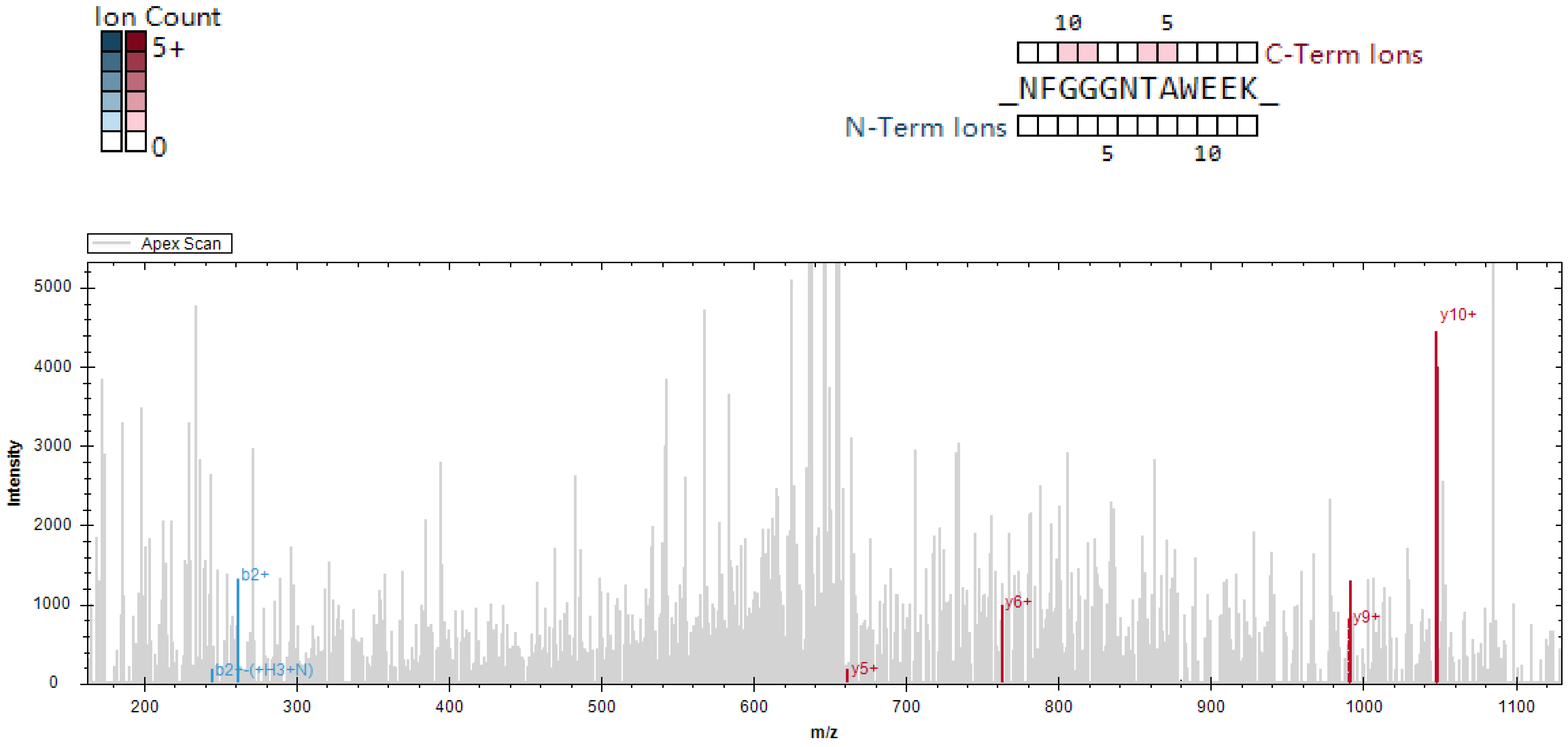

Ion Count
5+
0
10
5
C-Term Ions
_NFGGGNTAWEEK_
N-Term Ions
5
10
Apex Scan
Intensity
m/z
b2+
b2+-(+H3+N)
y5+
y6+
y9+
y10+

Q6ZN30

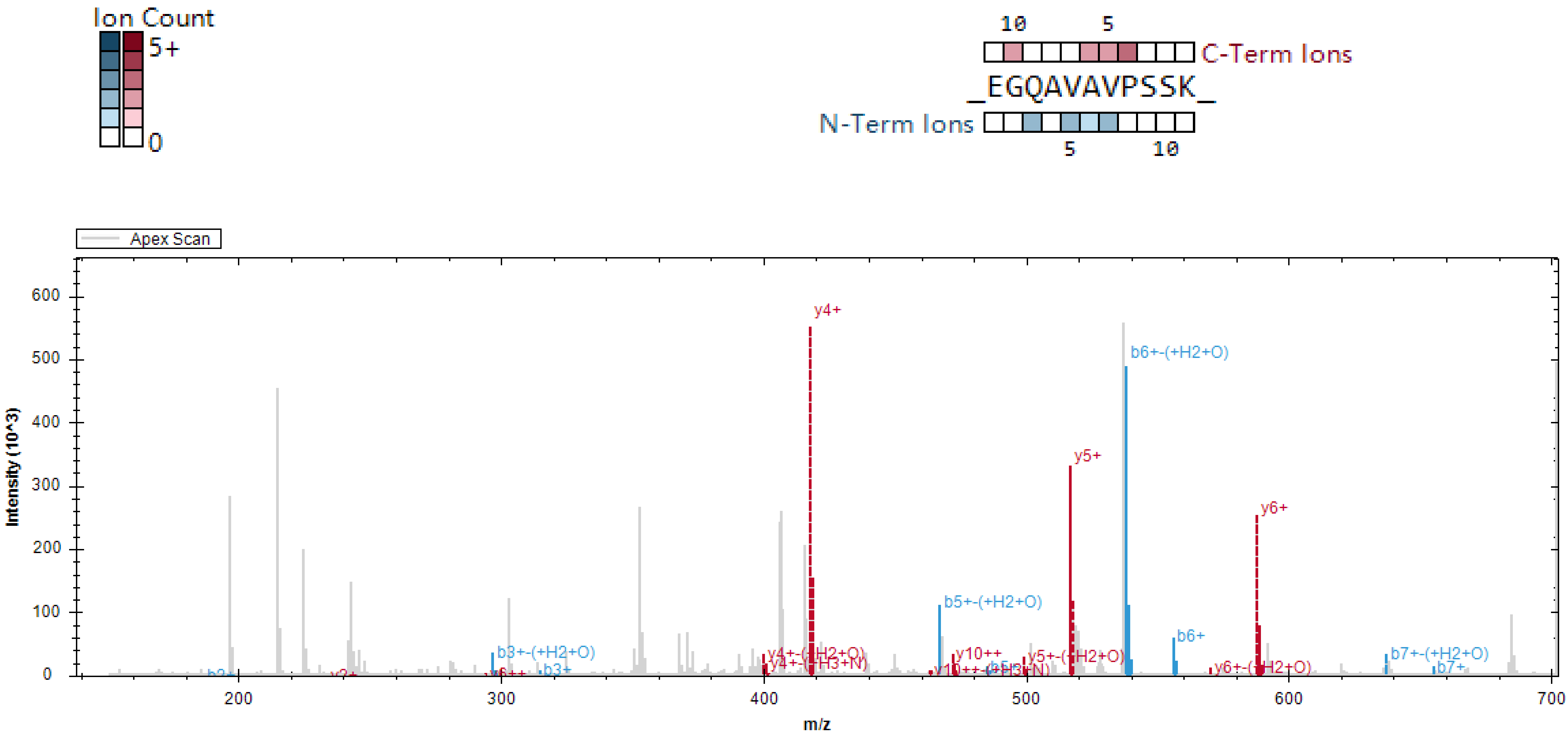

Ion Count
5+
0
10
5
C-Term Ions
_EGQAVAVPSSK_
N-Term Ions
5
10
Apex Scan
y4+
b6+-(+H2+O)
y5+
y6+
b5+-(+H2+O)
b6+
b3+-(+H2+O)
b3+
y10++
y5+-(+H2+O)
y6+-(+H2+O)
b7+-(+H2+O)
b7+
Intensity (10^3)
m/z

Q6ZTR7

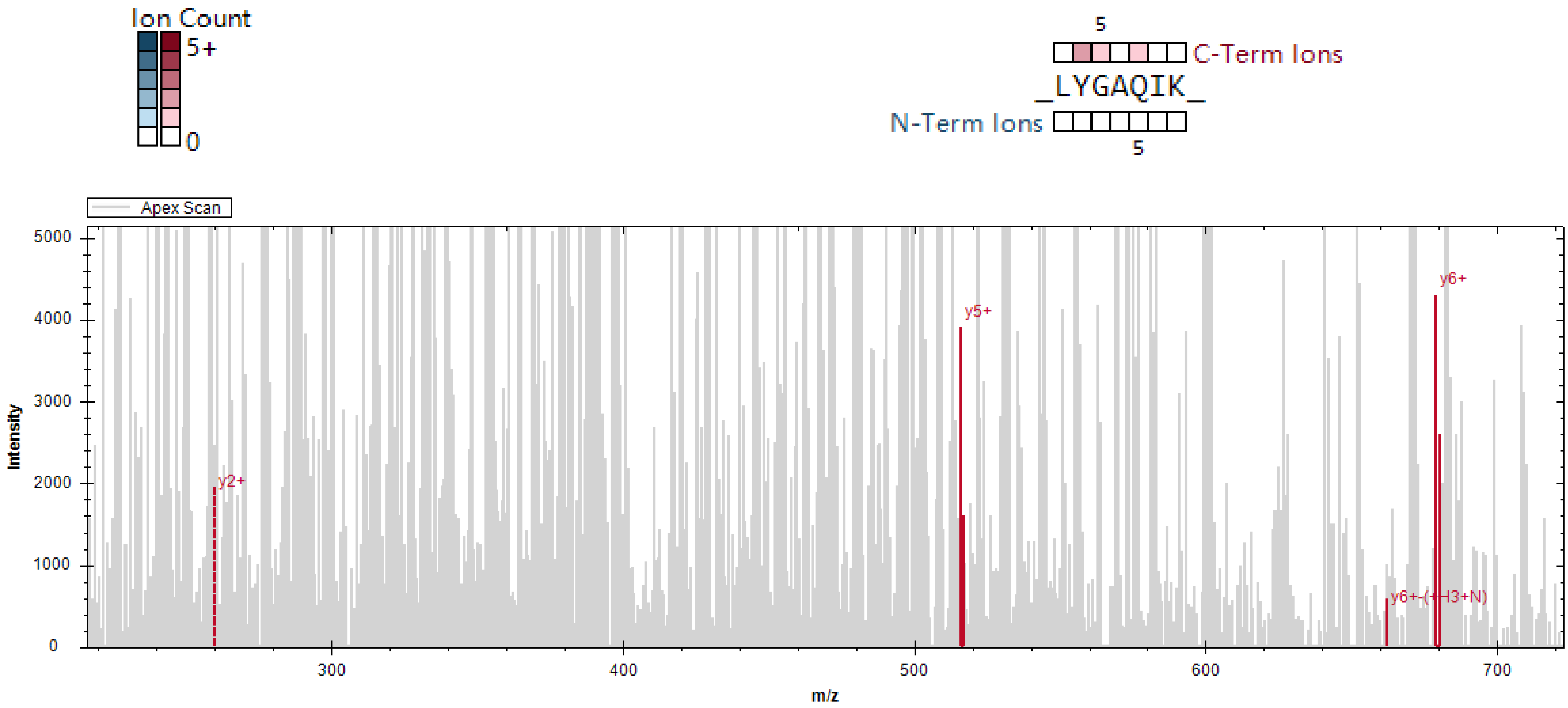
Ion Count
5+
0
5
C-Term Ions
_LYGAQIK_
N-Term Ions
5
Apex Scan
Intensity
m/z
y2+
y5+
y6+
y6+-(H3+N)

# Q6ZVW7

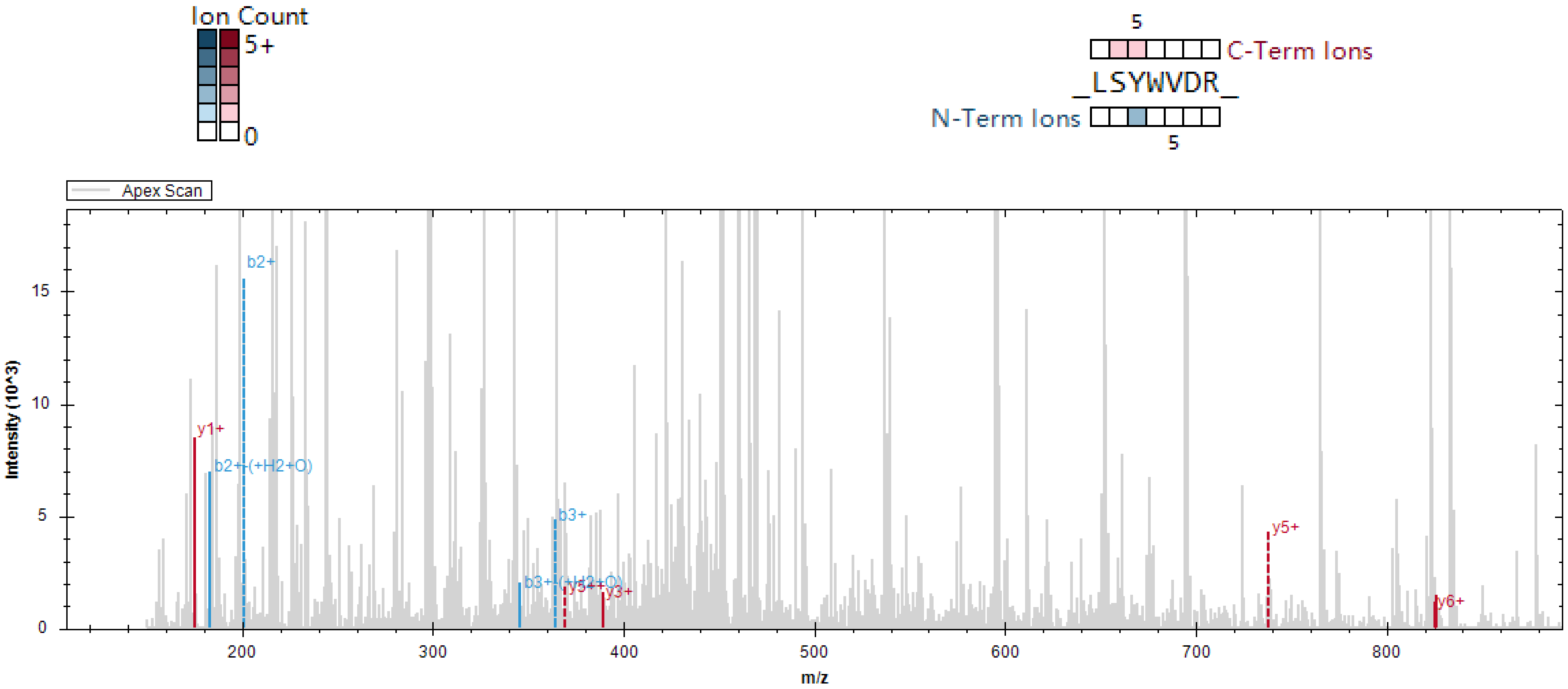

Ion Count
5+
0
5
C-Term Ions
_LSYWVDR_
N-Term Ions
5
Apex Scan
Intensity (10^3)
m/z
y1+
b2+
b2+-(+H2+O)
b3+
b3+-(+H2+O)
y5++
y3+
y5+
y6+

Q712K3

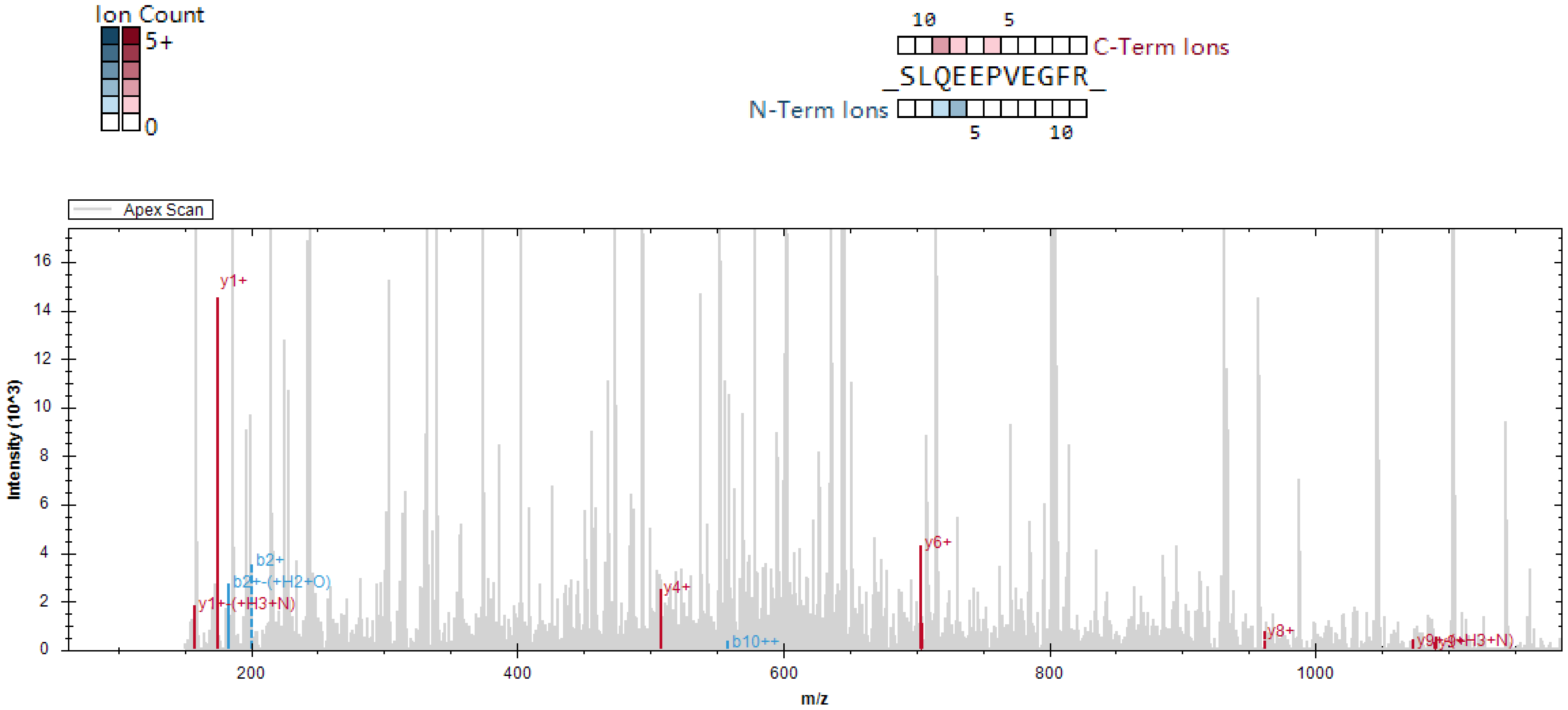

Ion Count
5+
0
10
5
C-Term Ions
_SLQEEPVEGFR_
N-Term Ions
5
10
Apex Scan
y1+
y1+-(+H3+N)
b2+
b2+-(+H2+O)
y4+
b10++
y6+
y8+
Intensity (10^3)
m/z

Q7L1S5

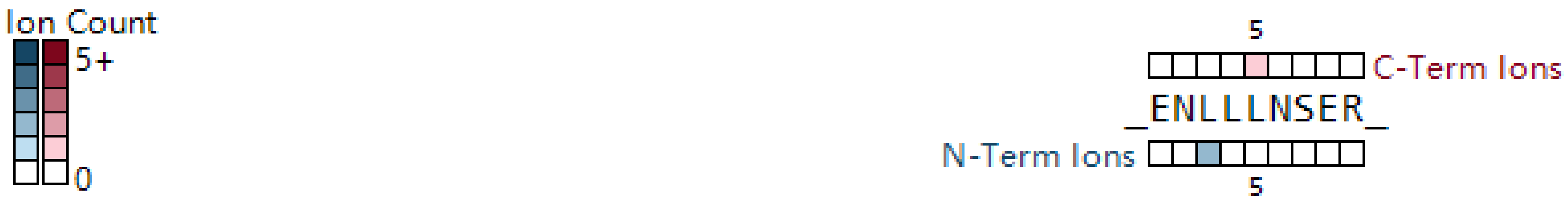

Ion Count
5+
0
5
C-Term Ions
_ENLLLNSER_
N-Term Ions
5


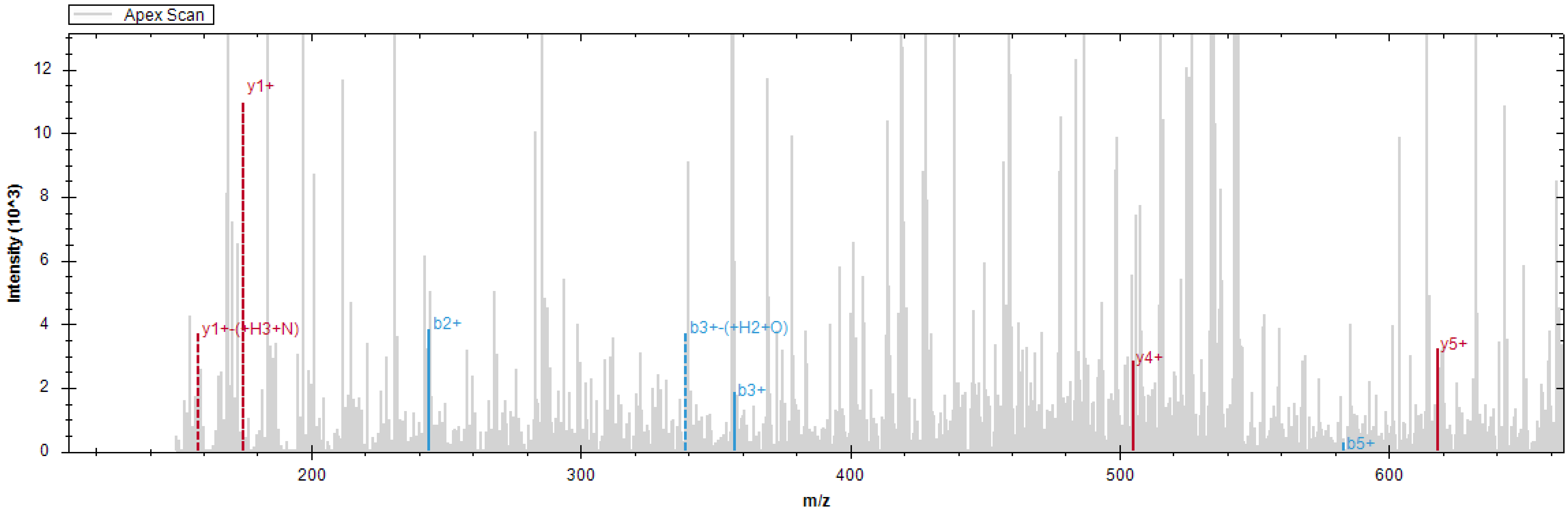

Apex Scan
Intensity (10^3)
m/z
y1+
y1+-(+H3+N)
b2+
b3+-(+H2+O)
b3+
y4+
y5+
b5+
0
2
4
6
8
10
12
200
300
400
500
600

Q7RTP0

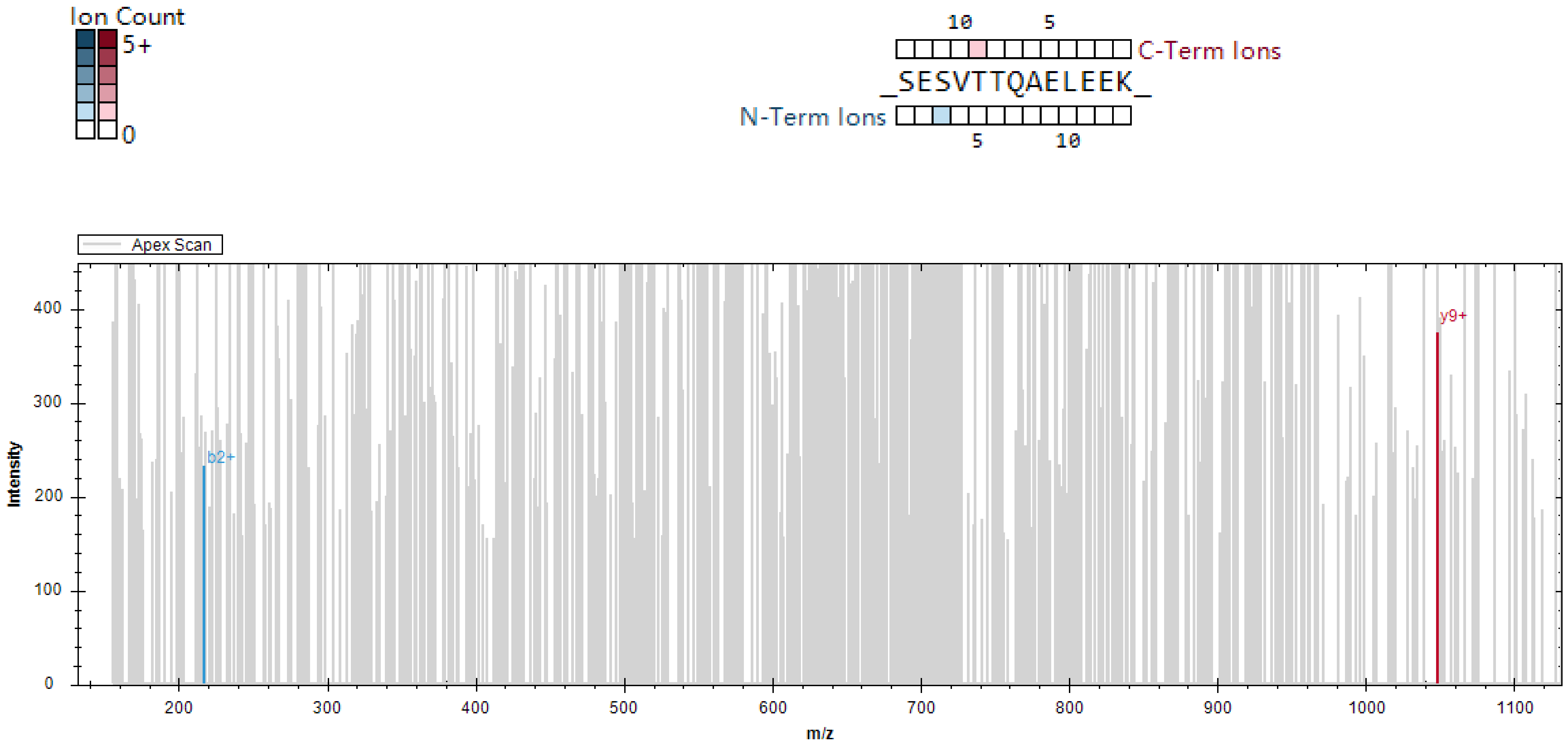

Ion Count
5+
0
10
5
C-Term Ions
_SESVTTQAELEEK_
N-Term Ions
5
10
Apex Scan
y9+
b2+
Intensity
m/z

Q7RTV2

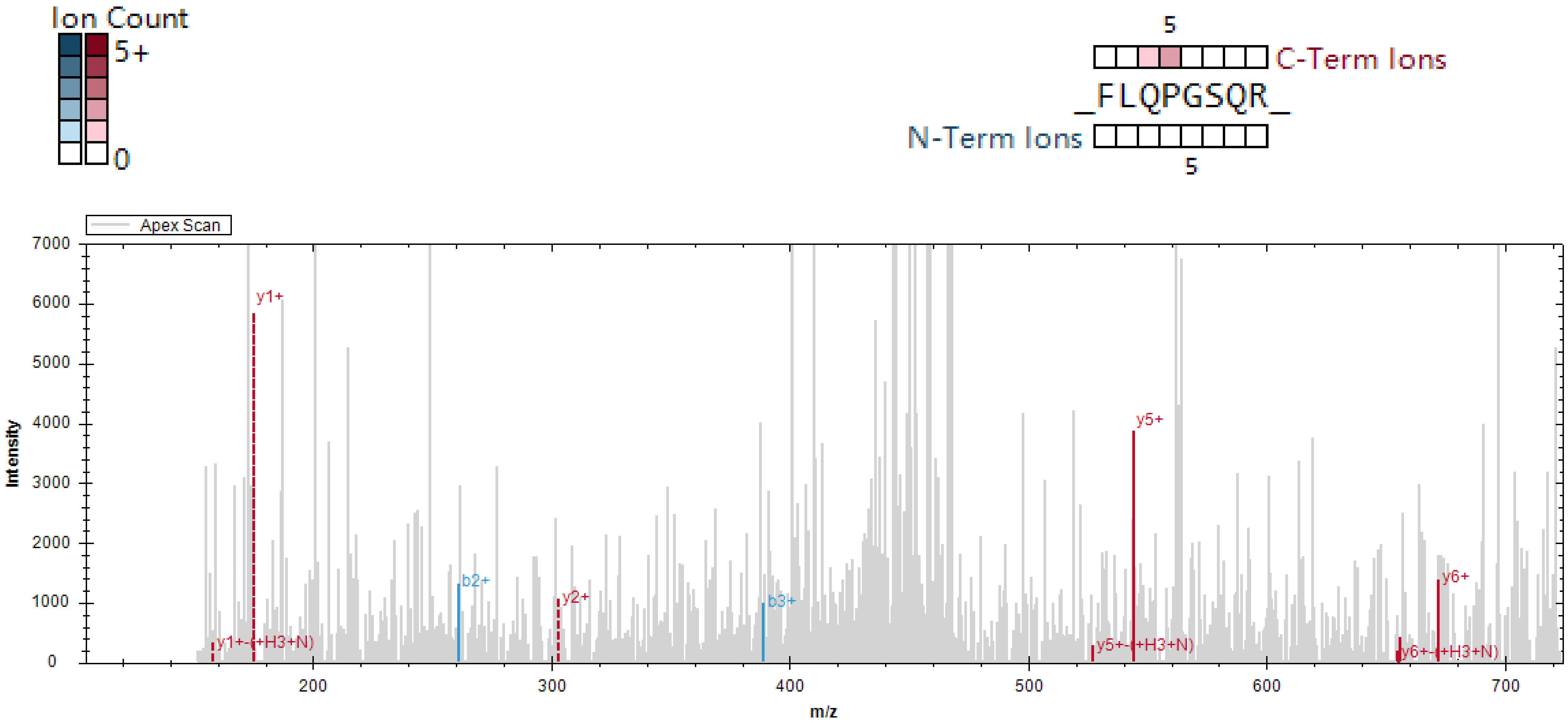

Ion Count
5+
0
5
C-Term Ions
_FLQPGSQR_
N-Term Ions
5
Apex Scan
Intensity
m/z
y1+
y1+-(+H3+N)
b2+
y2+
b3+
y5+
y5+-(+H3+N)
y6+
y6+-(+H3+N)

# Q7Z2Q7

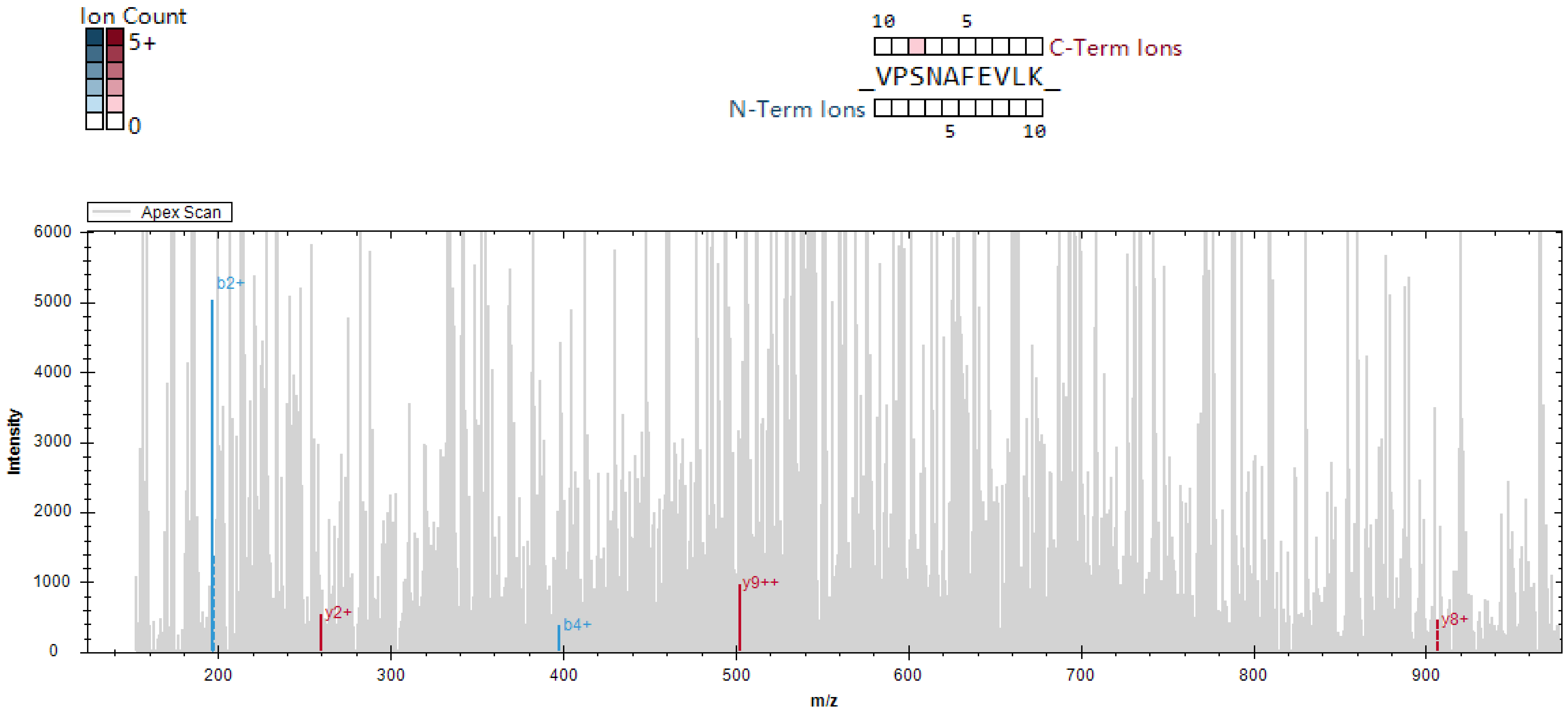

Ion Count
5+
0
10
5
C-Term Ions
_VPSNAFEVLK_
N-Term Ions
5
10
Apex Scan
6000
5000
4000
3000
2000
1000
0
Intensity
b2+
y2+
b4+
y9++
y8+
200
300
400
500
600
700
800
900
m/z

Q7Z3B0

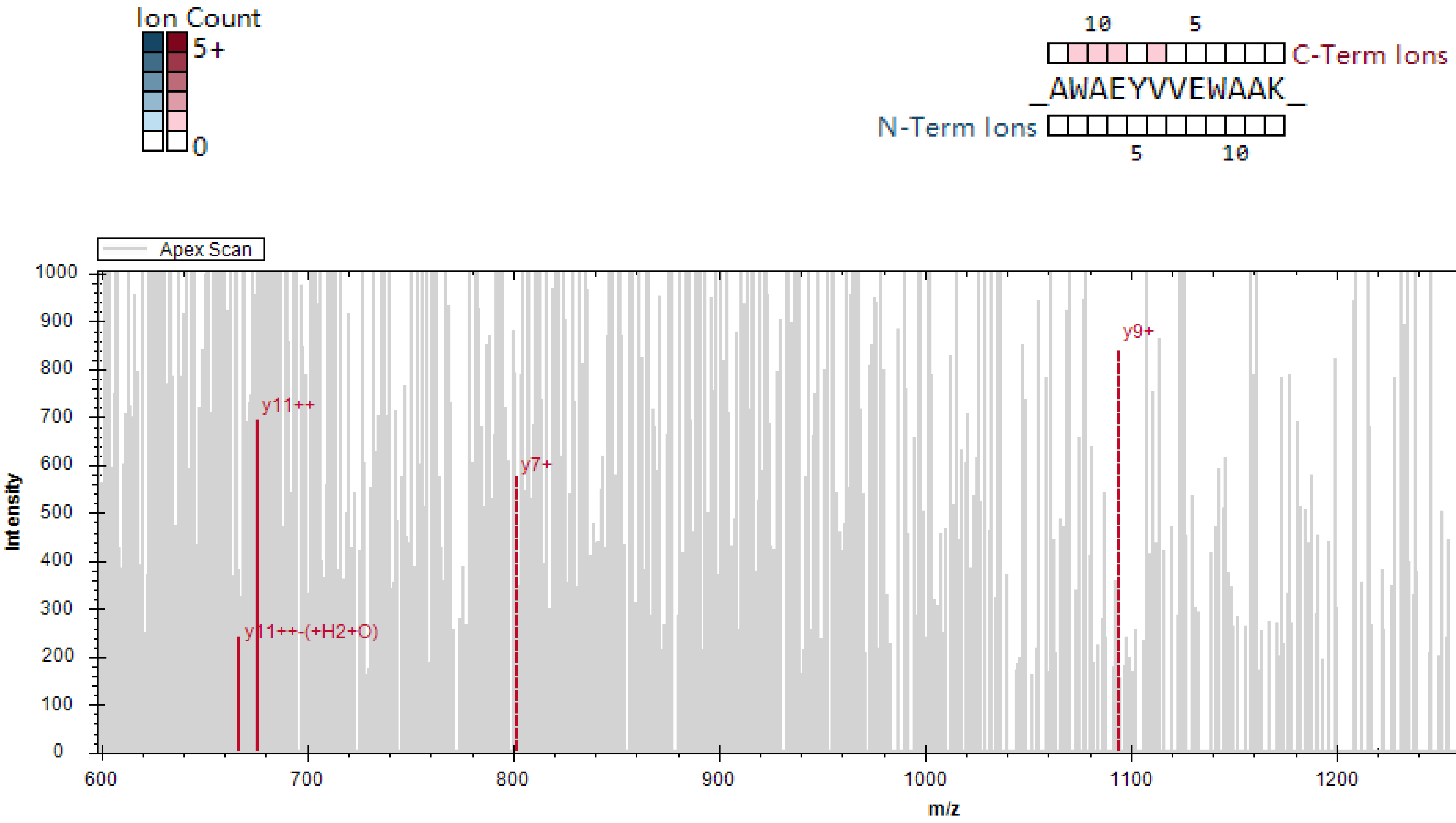
Ion Count
5+
0
10
5
C-Term Ions
_AWAEYVVEWAAK_
N-Term Ions
5
10
Apex Scan
Intensity
m/z
y11++
y11++-(+H2+O)
y7+
y9+
y11+

# Q7Z4L9

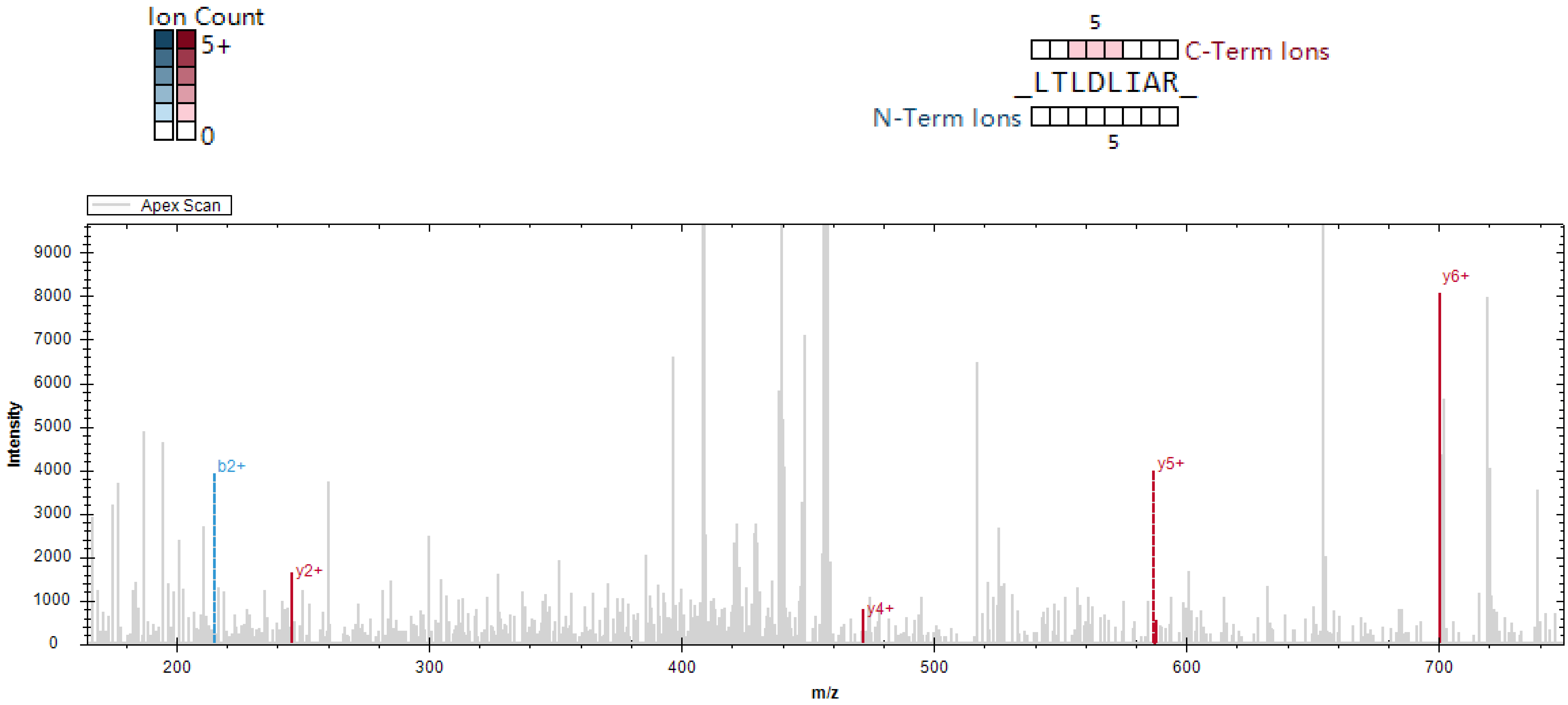

Ion Count
5+
0
5
C-Term Ions
_LTLDLIAR_
N-Term Ions
5
Apex Scan
Intensity
m/z
b2+
y2+
y4+
y5+
y6+

# Q7Z4R8

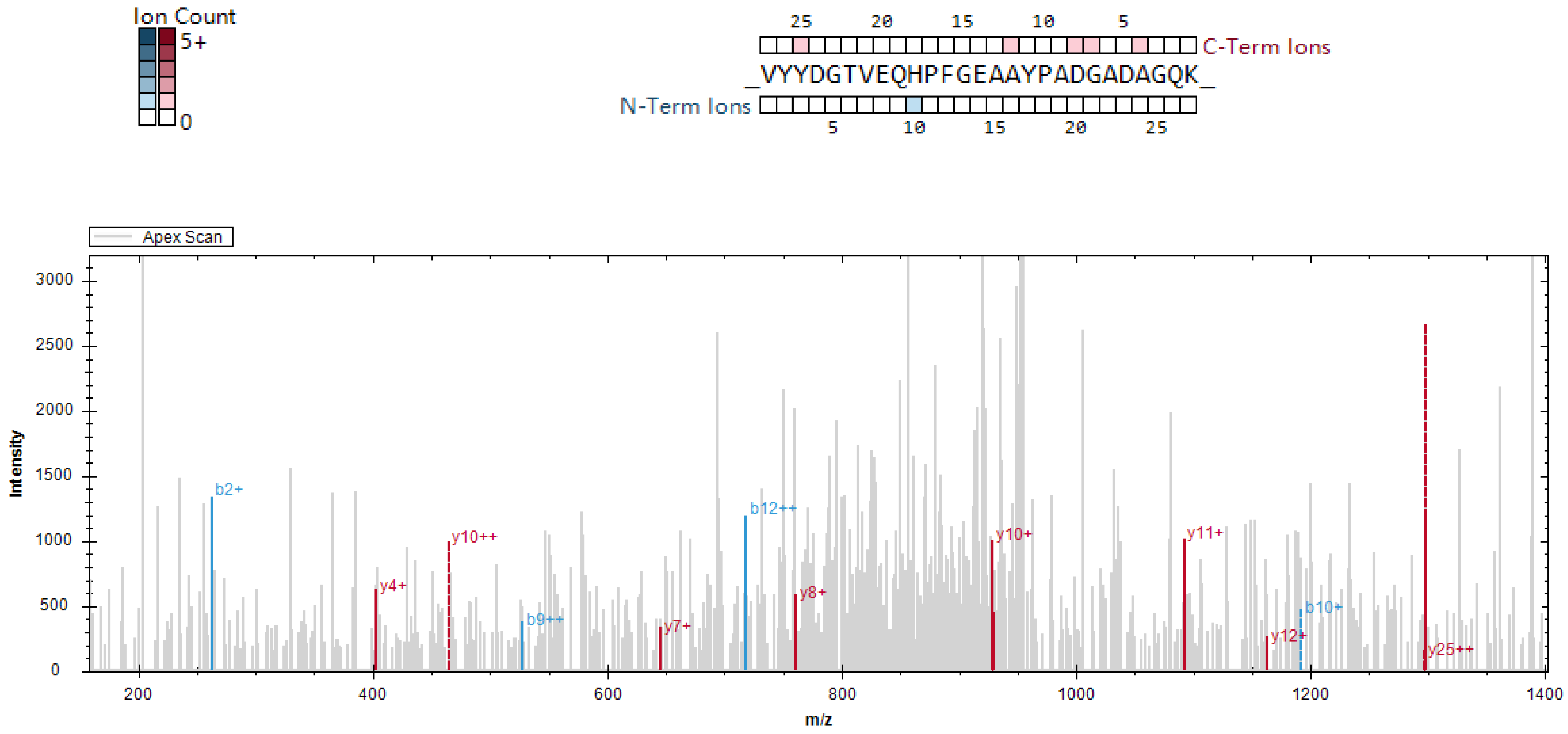

Ion Count
5+
0
25 20 15 10 5
C-Term Ions
_VYYDGTVEQHPFGEAAYPADGADAGQK_
N-Term Ions
5 10 15 20 25
Apex Scan
b2+
y4+
y10++
b9++
y7+
b12++
y8+
y10+
y11+
y12+
b10+
y25++
Intensity
m/z
0
500
1000
1500
2000
2500
3000
200
400
600
800
1000
1200
1400

Q7Z7G1

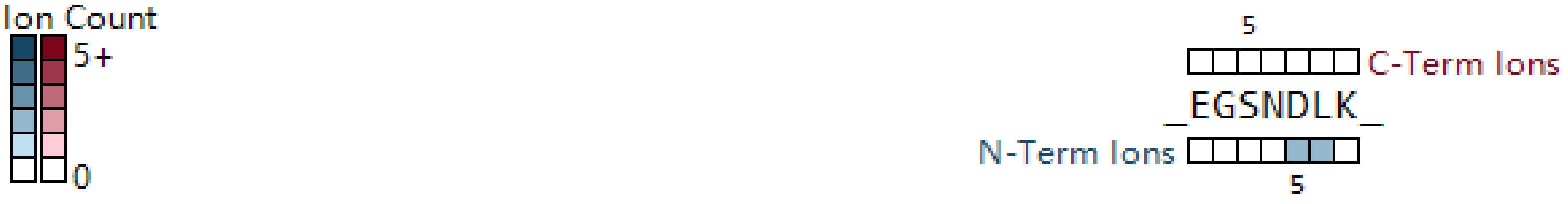

Ion Count
5+
0
5
C-Term Ions
_EGSNDLK_
N-Term Ions
5


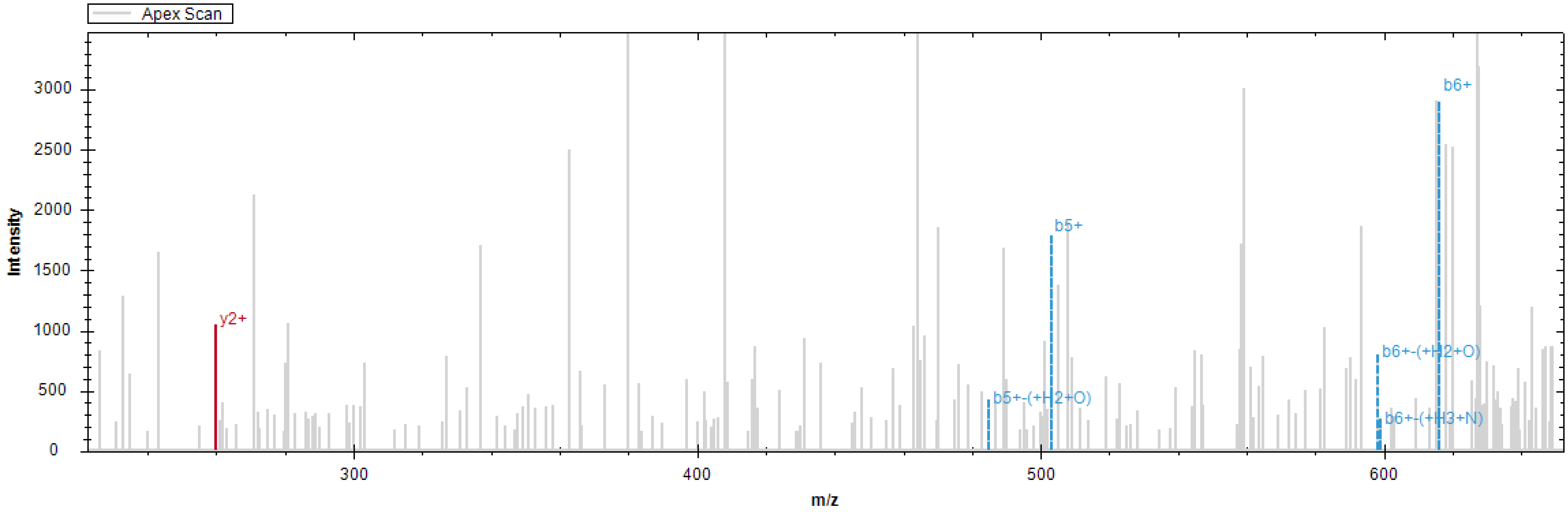

Apex Scan
Intensity
m/z
y2+
b5+
b5+-(+H2+O)
b6+
b6+-(+H2+O)
b6+-(+H3+N)

Q7Z7M9

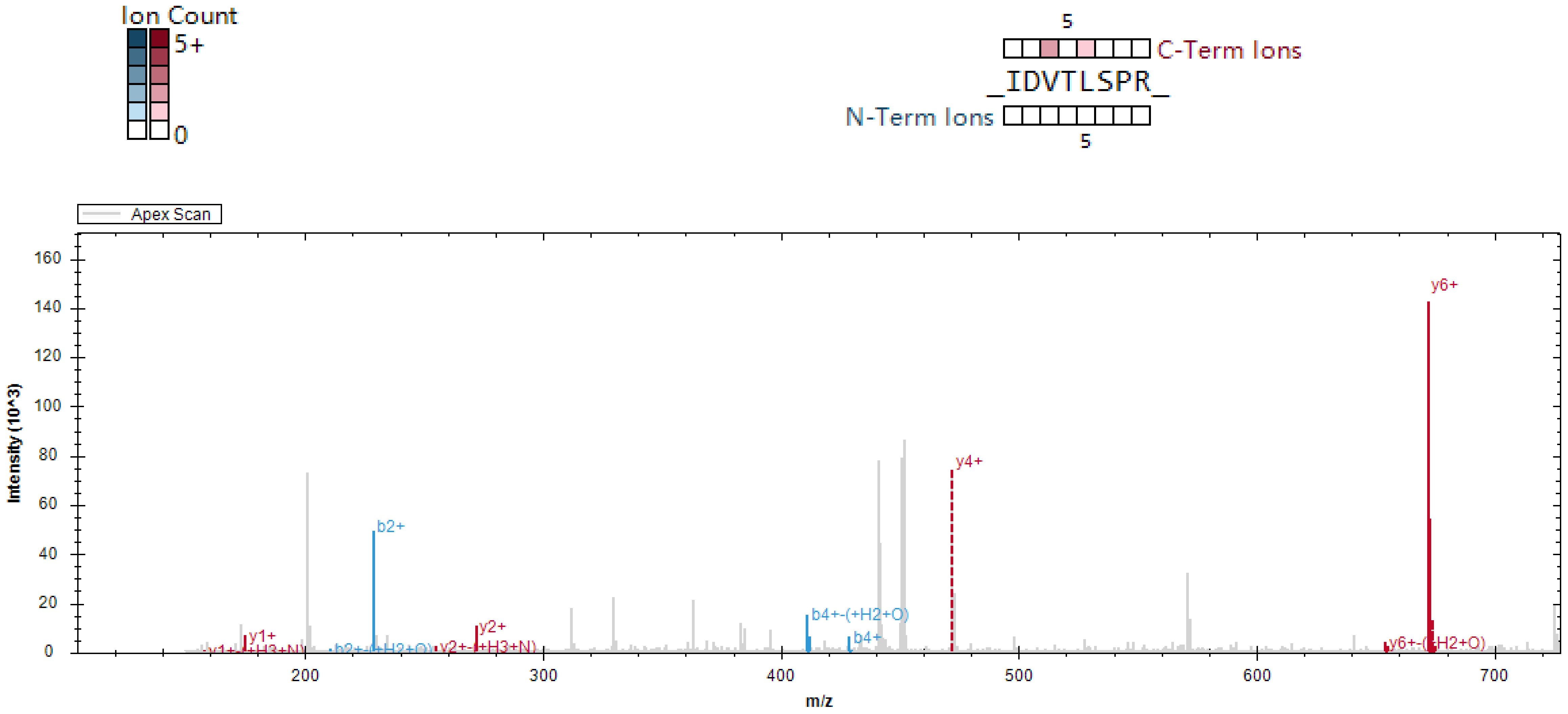

Ion Count
5+
0
5
C-Term Ions
_IDVTLSPR_
N-Term Ions
5
Apex Scan
y6+
y4+
b2+
b4+-(+H2+O)
b4+
y2+
y2+-(+H3+N)
y1+
y6+-(+H2+O)
Intensity (10^3)
m/z
0
20
40
60
80
100
120
140
160
200
300
400
500
600
700

Q86TH1

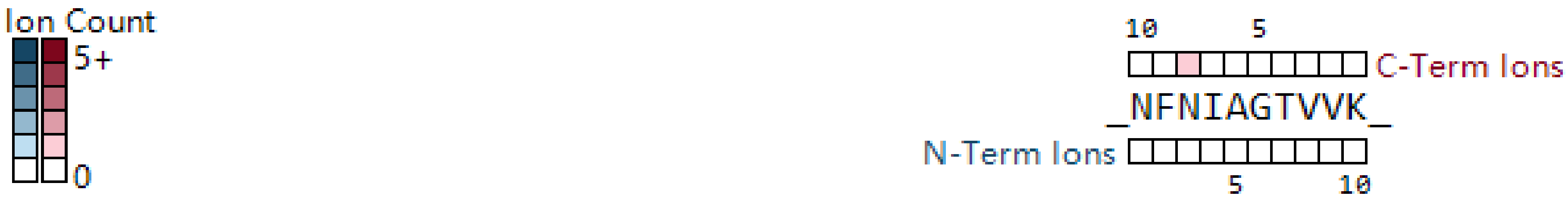

Ion Count
5+
0
10
5
C-Term Ions
_NFNIAGTVVK_
N-Term Ions
5
10


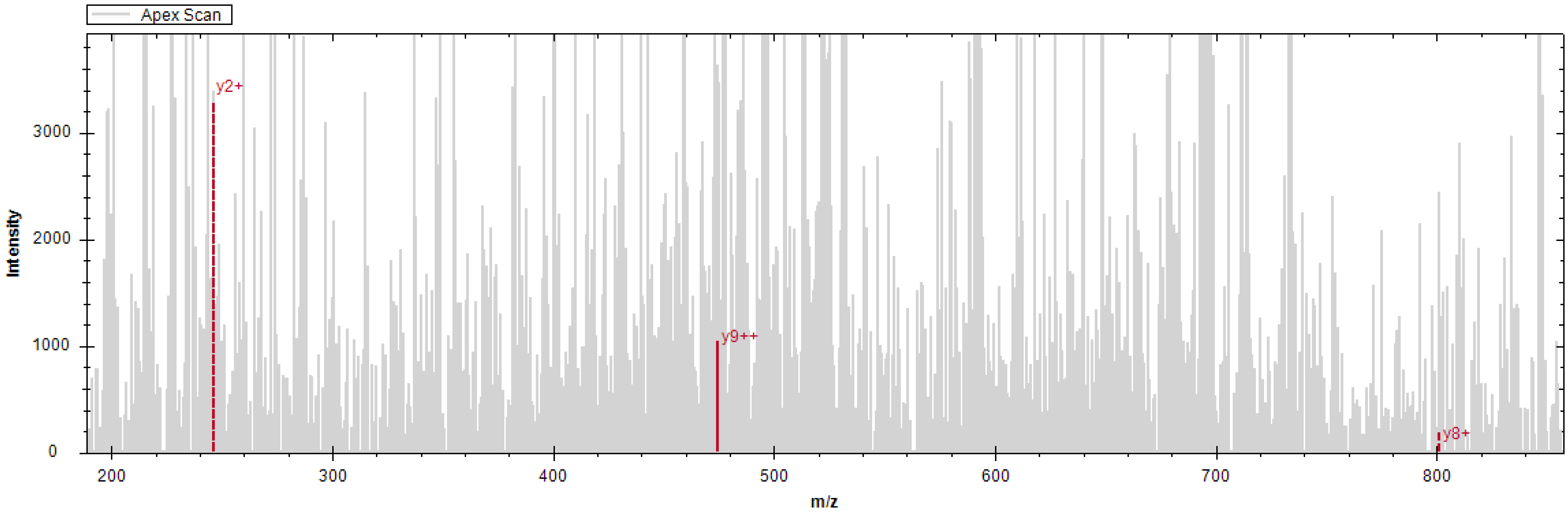

Apex Scan
y2+
y9++
y8+
Intensity
3000
2000
1000
0
200
300
400
500
600
700
800
m/z

Q86UQ4

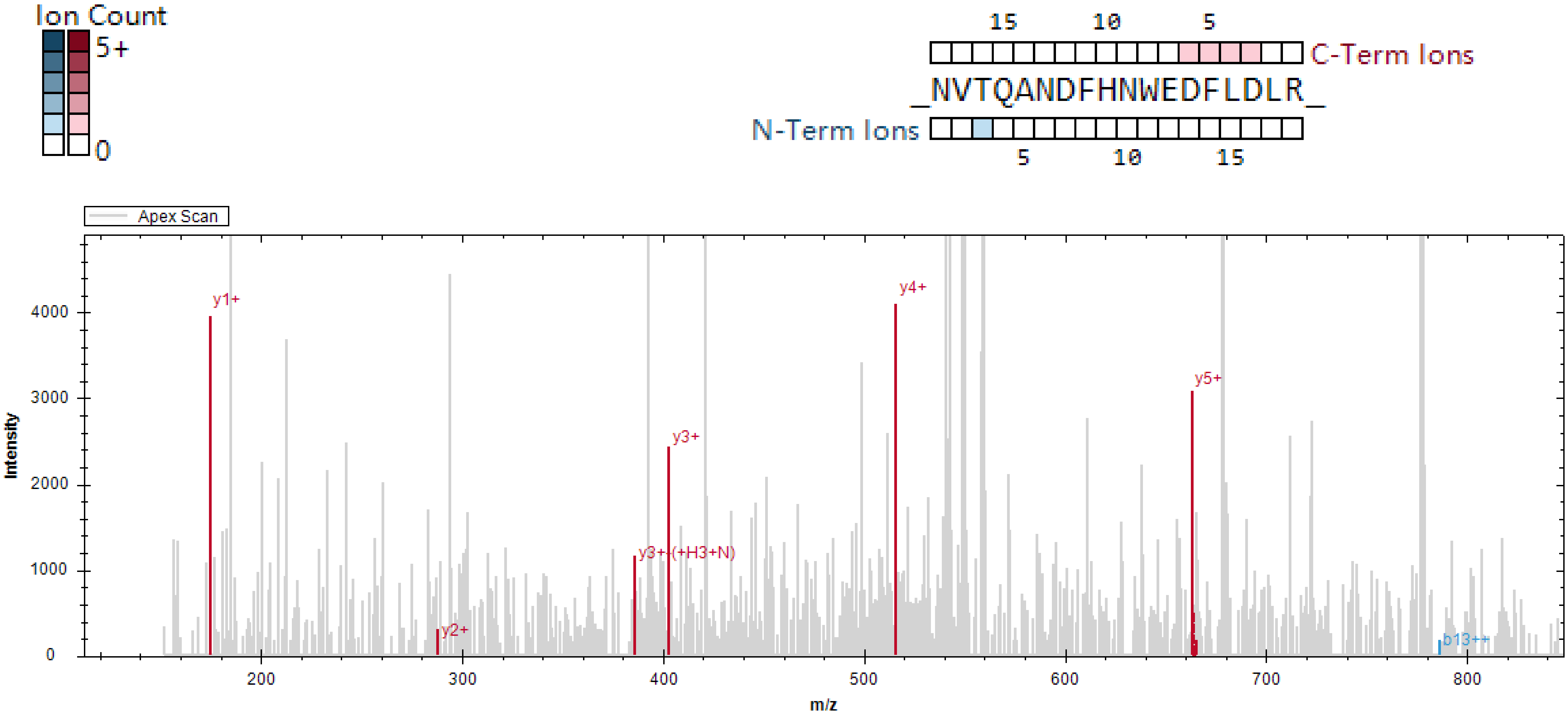

Ion Count
5+
0
15 10 5
C-Term Ions
_NVTQANDFHNWEDFLDLR_
N-Term Ions
5 10 15
Apex Scan
Intensity
m/z
y1+
y2+
y3+
y3++(+H3+N)
y4+
y5+
b13++

Q86VZ1

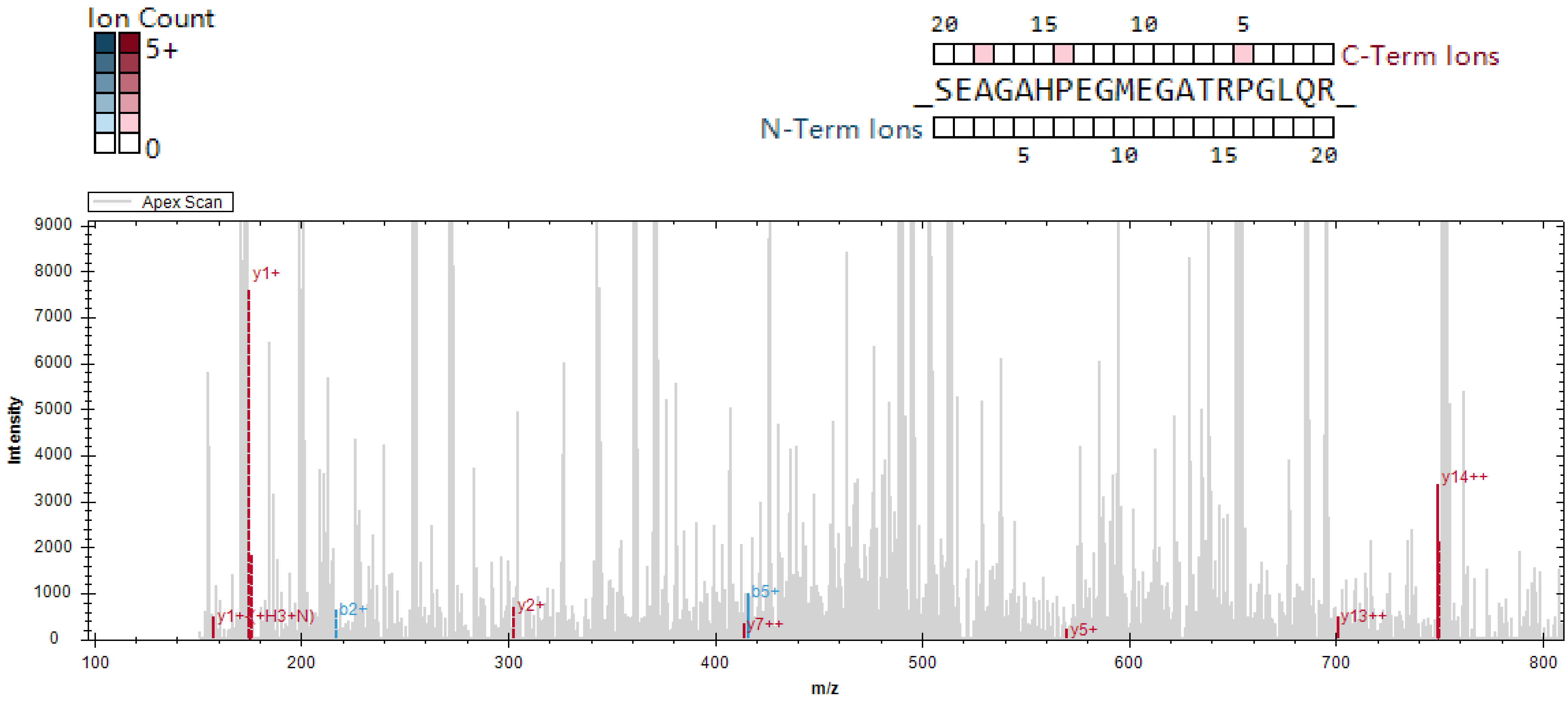

Ion Count
5+
0
20
15
10
5
C-Term Ions
_SEAGAHPEGMEGATRPGLQR_
N-Term Ions
5
10
15
20
Apex Scan
Intensity
m/z
y1+
y1+-(+H3+N)
b2+
y2+
b5+
y7++
y5+
y13++
y14++

Q86W25

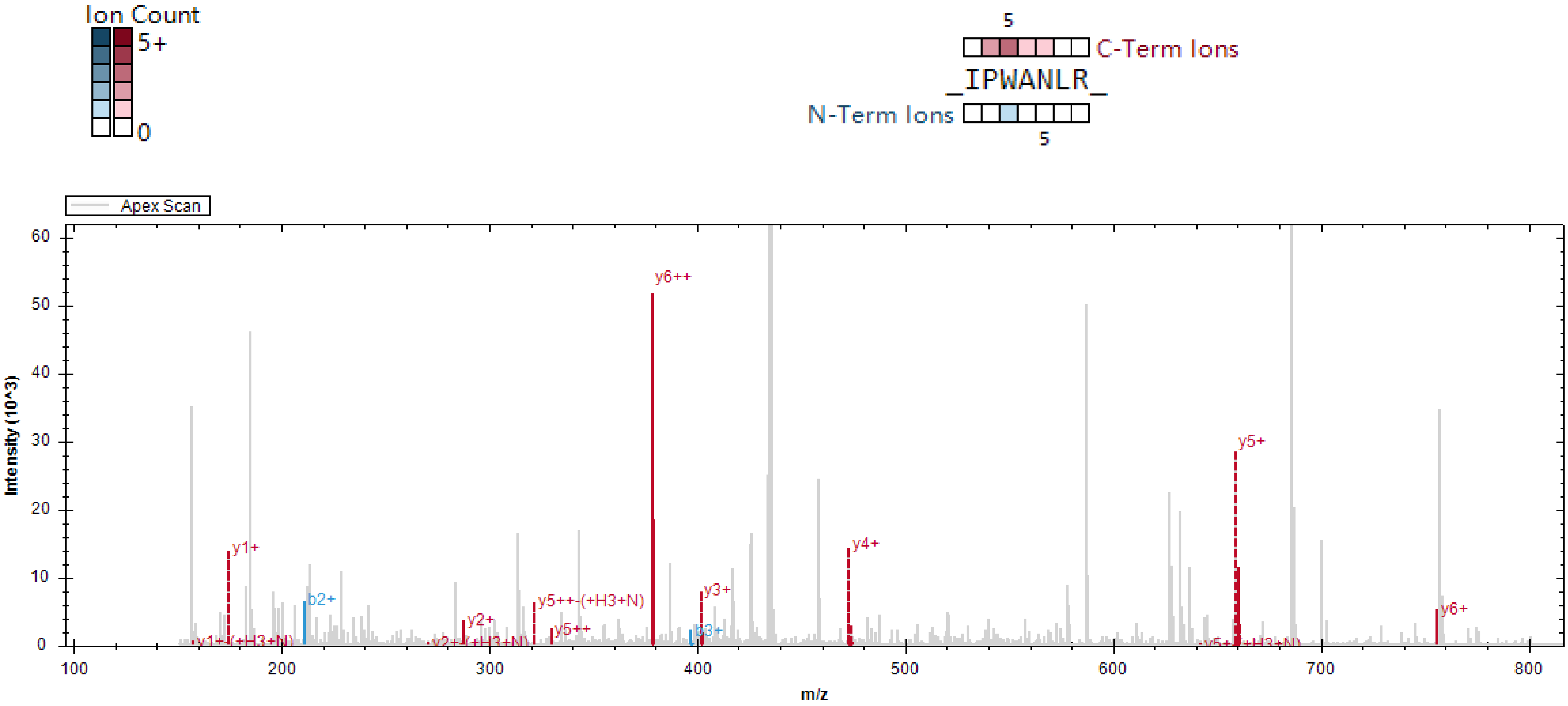

Ion Count
5+
0
5
C-Term Ions
_IPWANLR_
N-Term Ions
5
Apex Scan
y6++
y5+
y1+
y4+
b2+
y5++-(+H3+N)
y3+
y2+
y5++
b3+
y6+
Intensity (10^3)
m/z

Q86X83

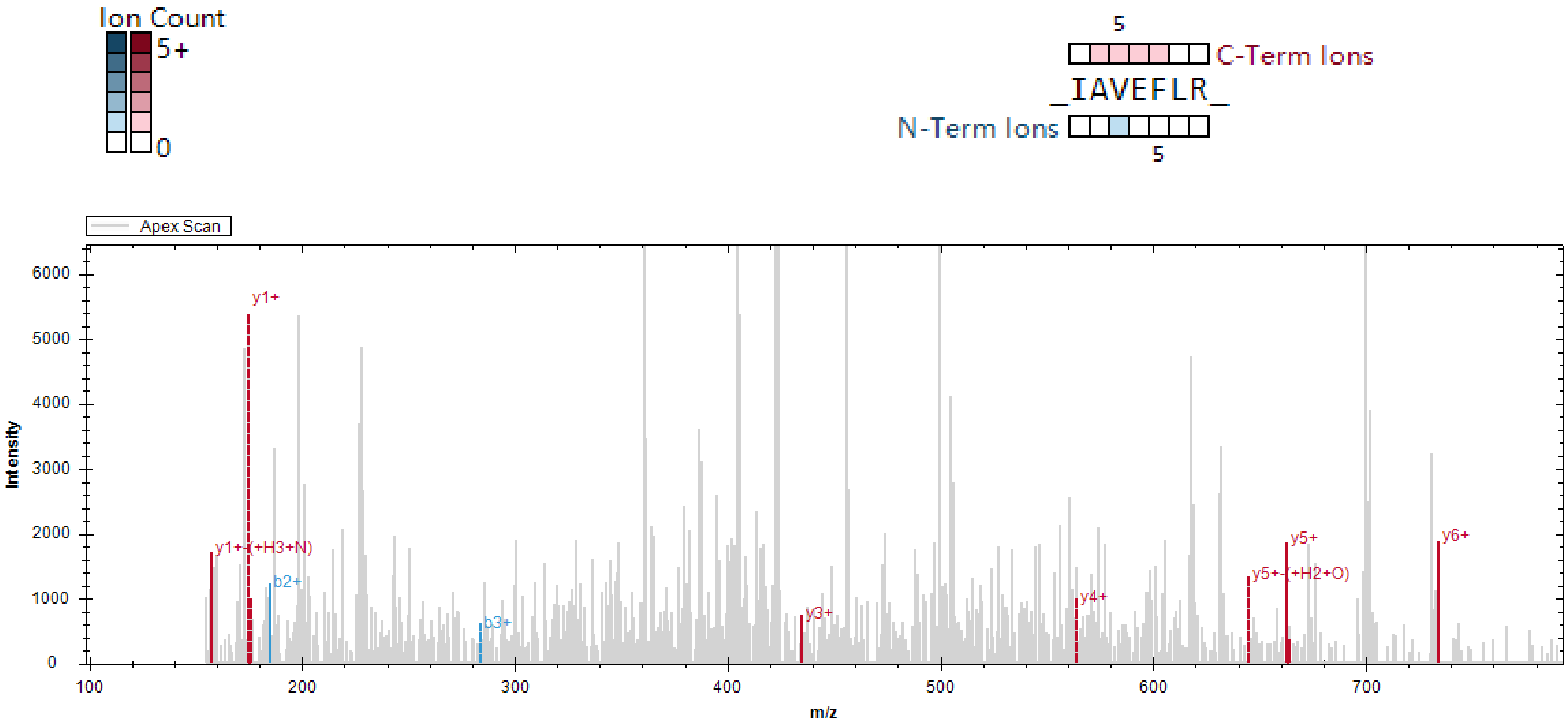
Ion Count
5+
0
5
C-Term Ions
_IAVEFLR_
N-Term Ions
5
Apex Scan
Intensity
m/z
y1+
y1+-(+H3+N)
b2+
b3+
y3+
y4+
y5+-(+H2+O)
y5+
y6+

Q86XI8

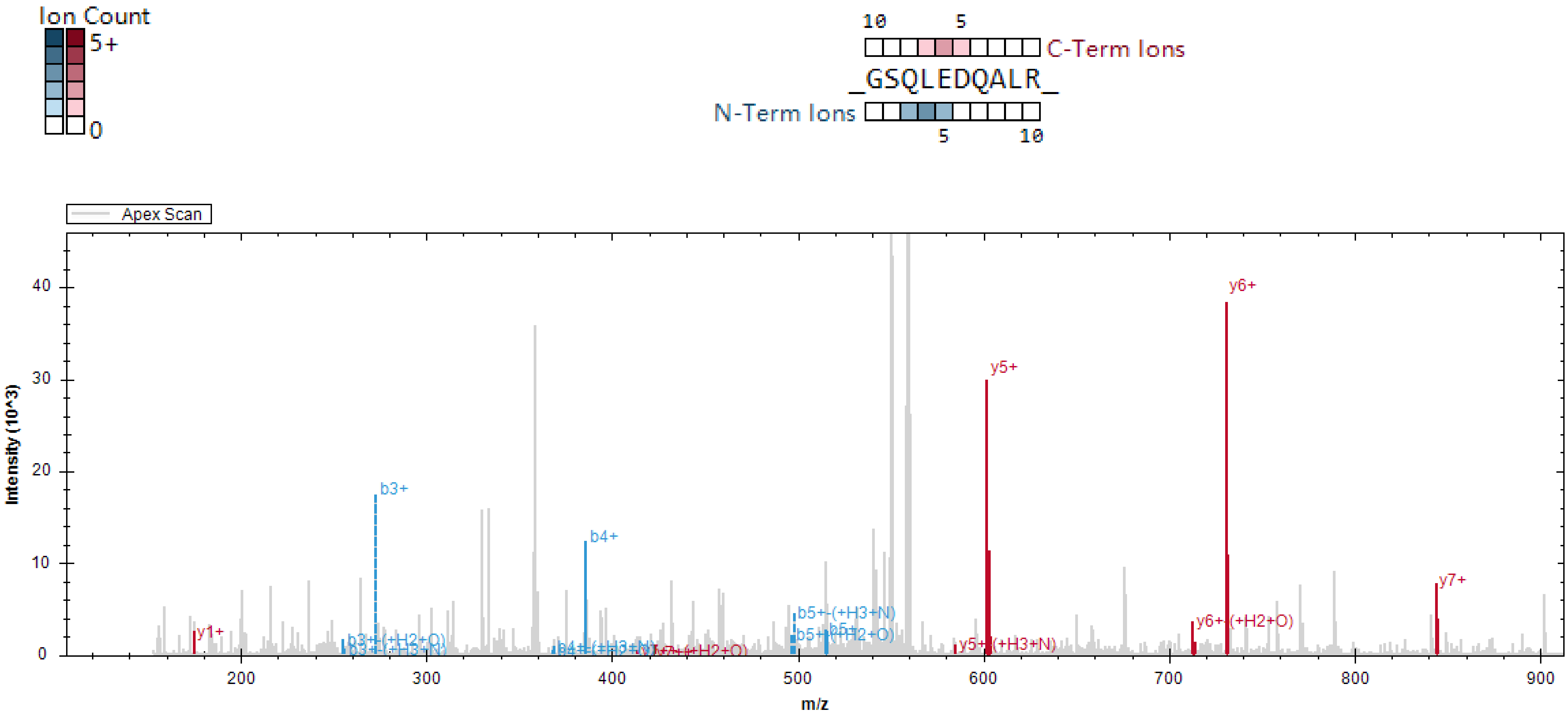
Ion Count
5+
0
10
5
C-Term Ions
_GSQLEDQALR_
N-Term Ions
5
10
Apex Scan
Intensity (10^3)
m/z
y6+
y5+
b3+
b4+
y7+
y1+
y6+-(+H2+O)
b5+-(+H3+N)
y5+-(+H3+N)

Q86XP6

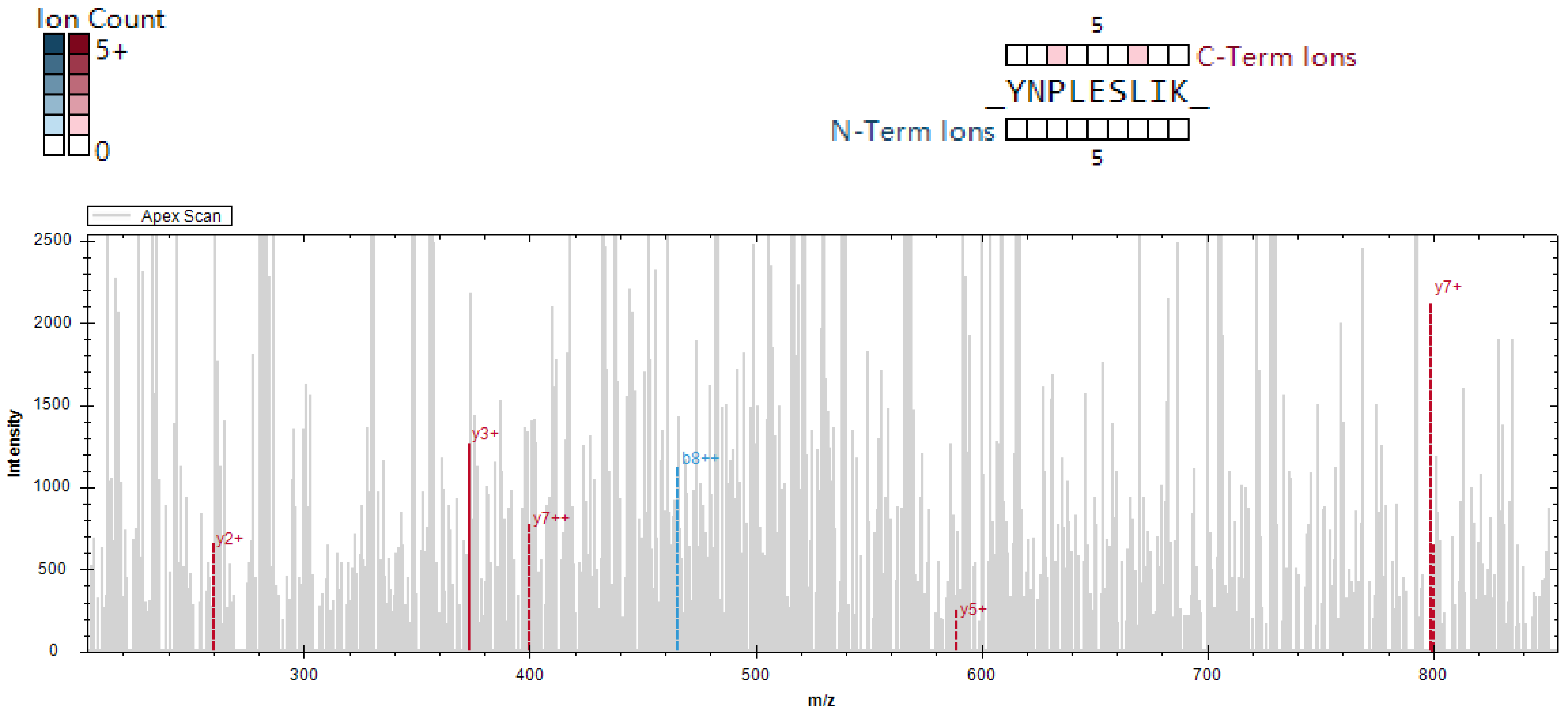

Ion Count
5+
0
5
C-Term Ions
_YNPLESLIK_
N-Term Ions
5
Apex Scan
Intensity
m/z
y7+
y3+
b8++
y7++
y2+
y5+

Q86XR2

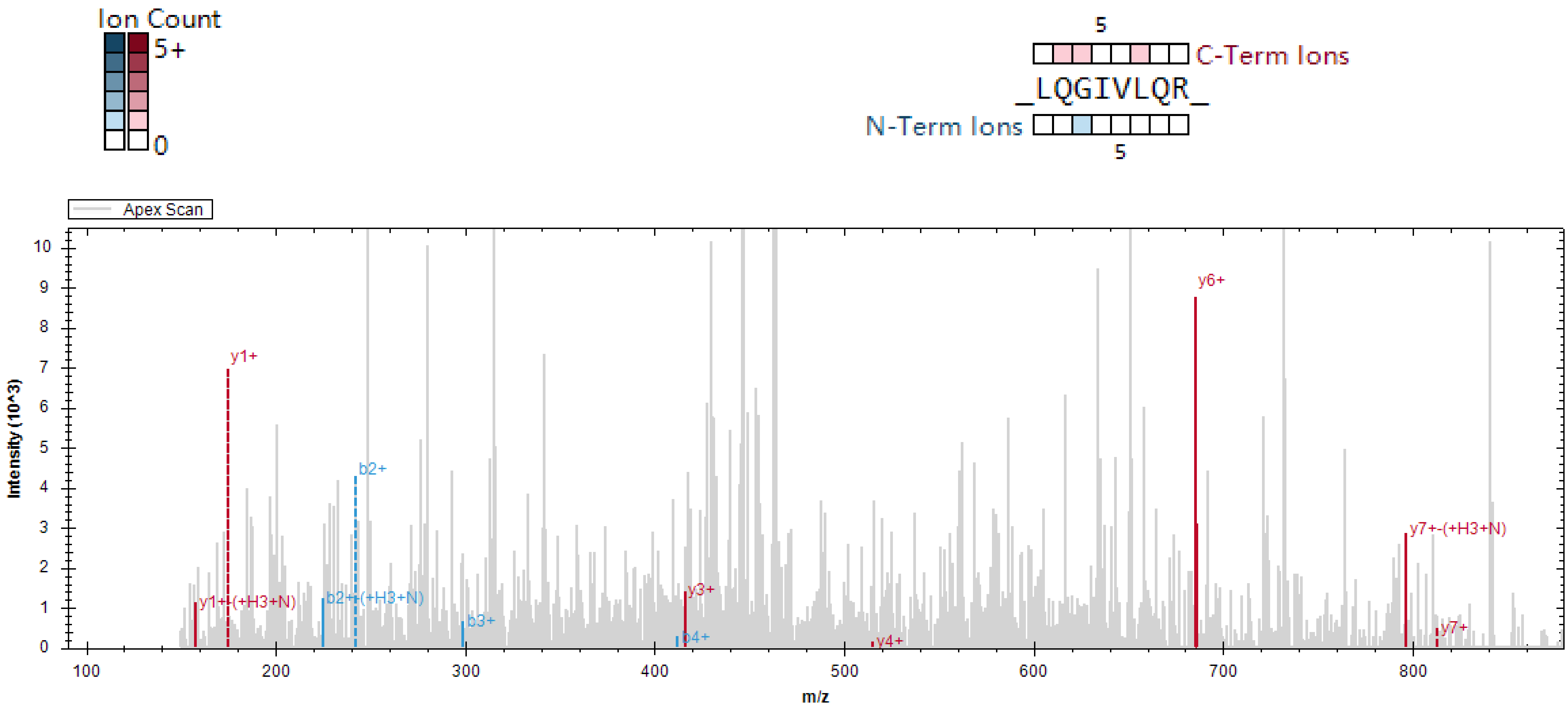

Ion Count
5+
0
5
C-Term Ions
_LQGIVLQR_
N-Term Ions
5
Apex Scan
Intensity (10^3)
m/z
y1+
y1+-(+H3+N)
b2+
b2+-(+H3+N)
b3+
b4+
y3+
y4+
y6+
y7+-(+H3+N)
y7+

Q86YJ7

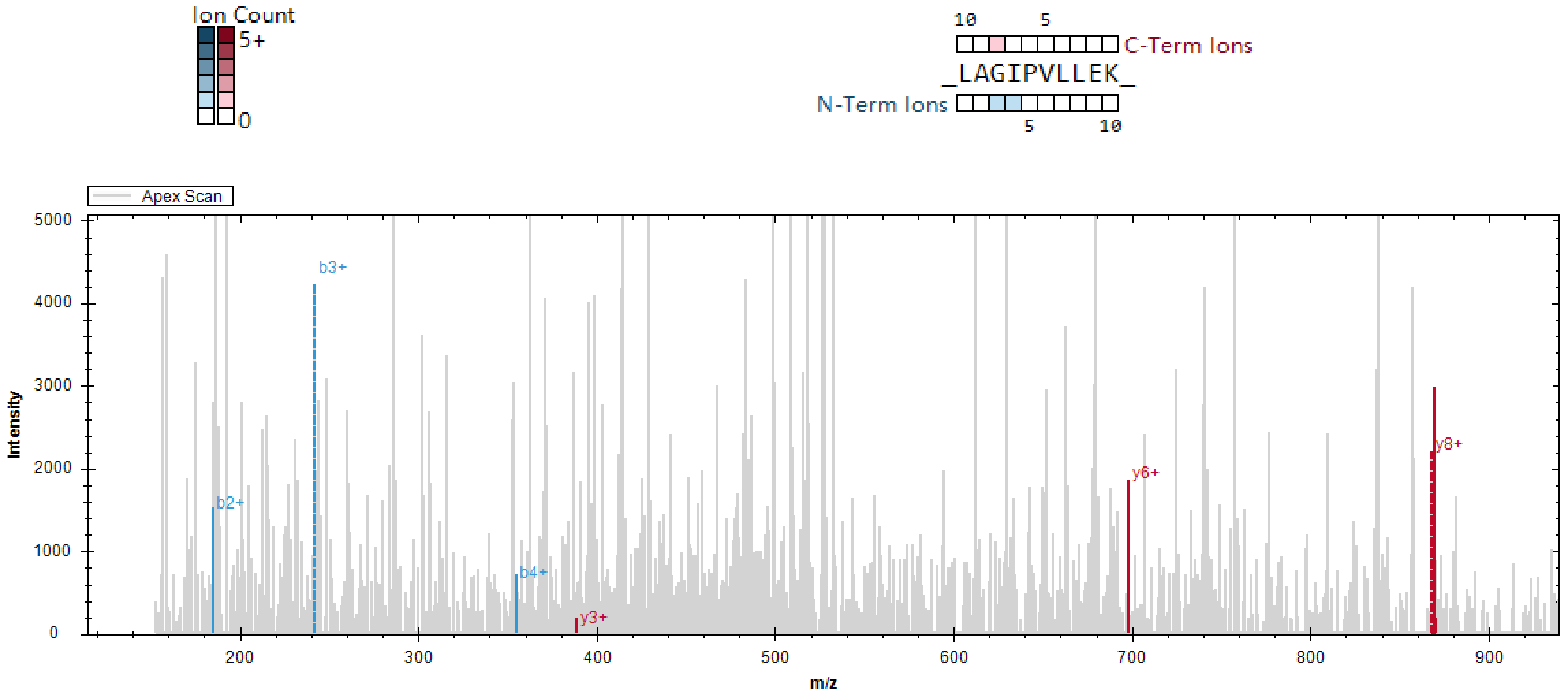

Ion Count
5+
0
10
5
C-Term Ions
_LAGIPVLLEK_
N-Term Ions
5
10
Apex Scan
Intensity
m/z
b2+
b3+
b4+
y3+
y6+
y8+

Q8IUE6

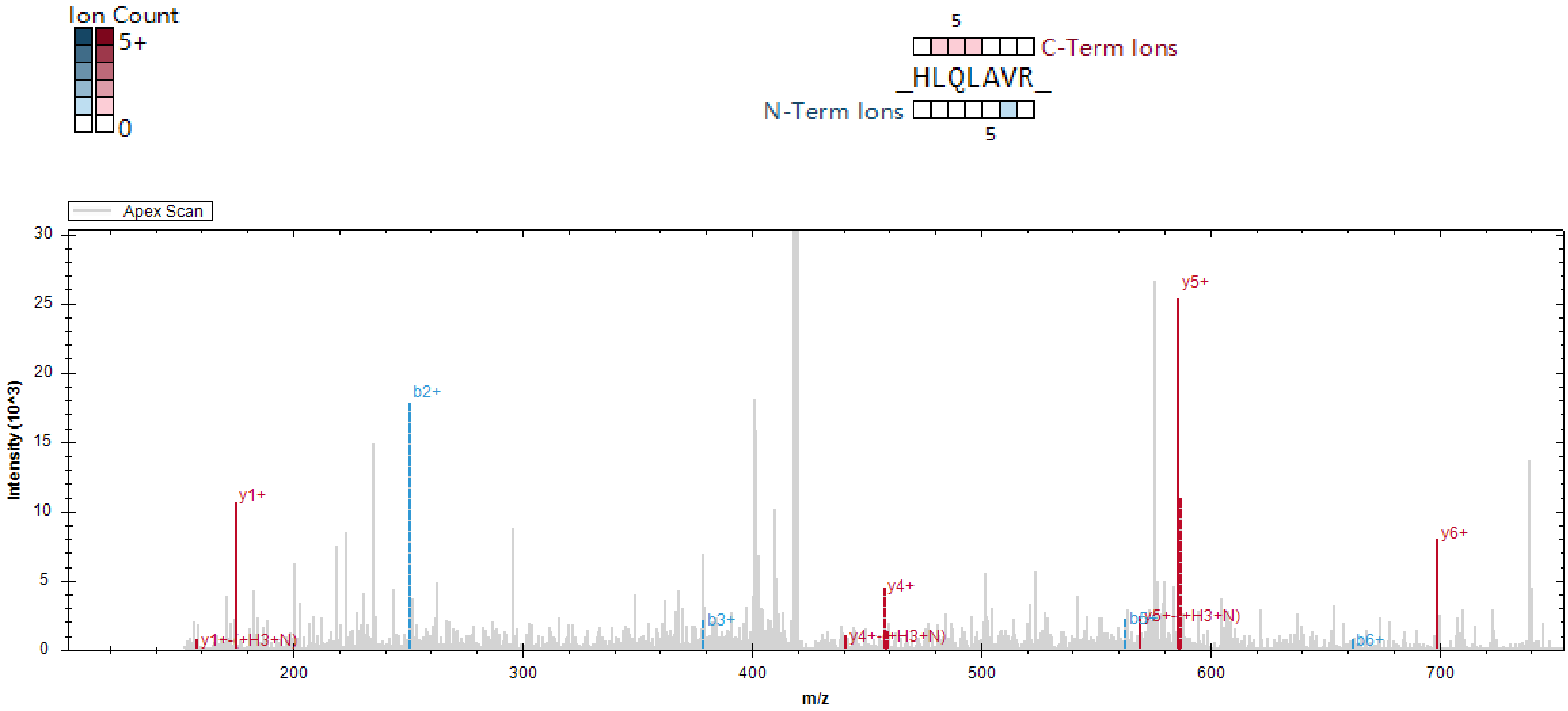

Ion Count
5+
0
5
C-Term Ions
_HLQLAVR_
N-Term Ions
5
Apex Scan
y1+
y1+-(+H3+N)
b2+
b3+
y4+
y4+-(+H3+N)
b5+
y5+
y5+-(+H3+N)
b6+
y6+
Intensity (10^3)
m/z

# Q8IUX4

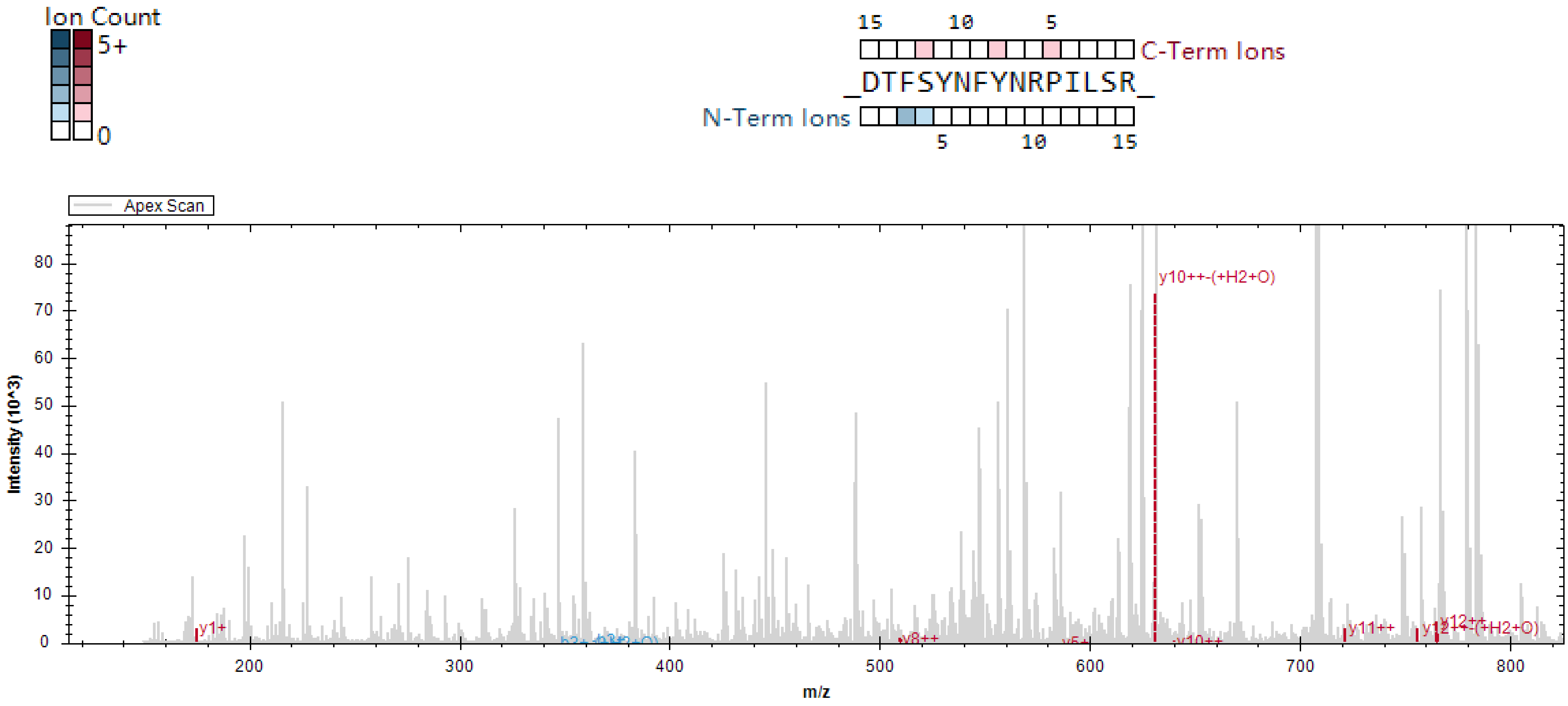
Ion Count
5+
0
15
10
5
C-Term Ions
_DTFSYNFYNRPILSR_
N-Term Ions
5
10
15
Apex Scan
Intensity (10^3)
m/z
y10++-(+H2+O)
y1+
y8++
y5+
y10++
y11++
y12++
y12++-(+H2+O)

Q8IV03

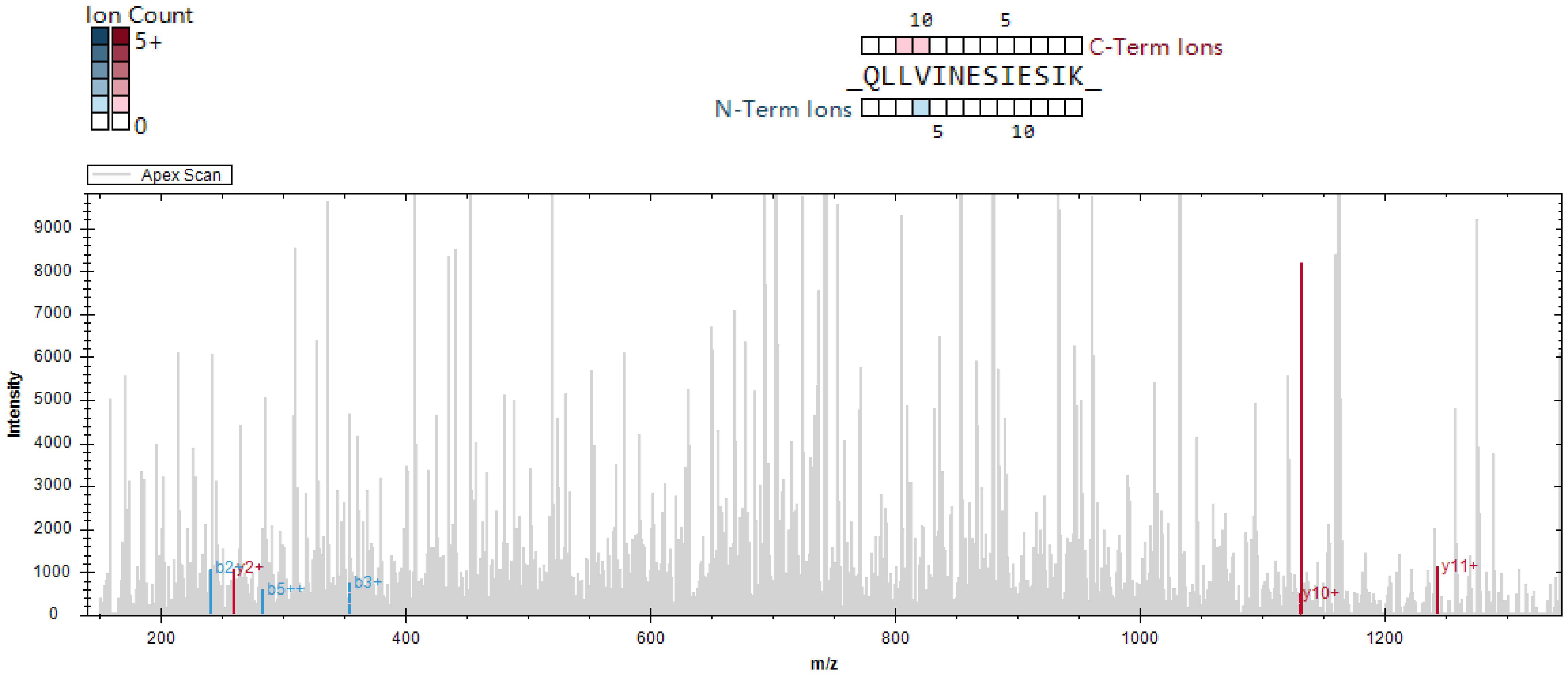

Ion Count
5+
0
10
5
C-Term Ions
_QLLVINESIESIK_
N-Term Ions
5
10
Apex Scan
Intensity
m/z
b2+
y2+
b5++
b3+
y10+
y11+

# Q8IVW1

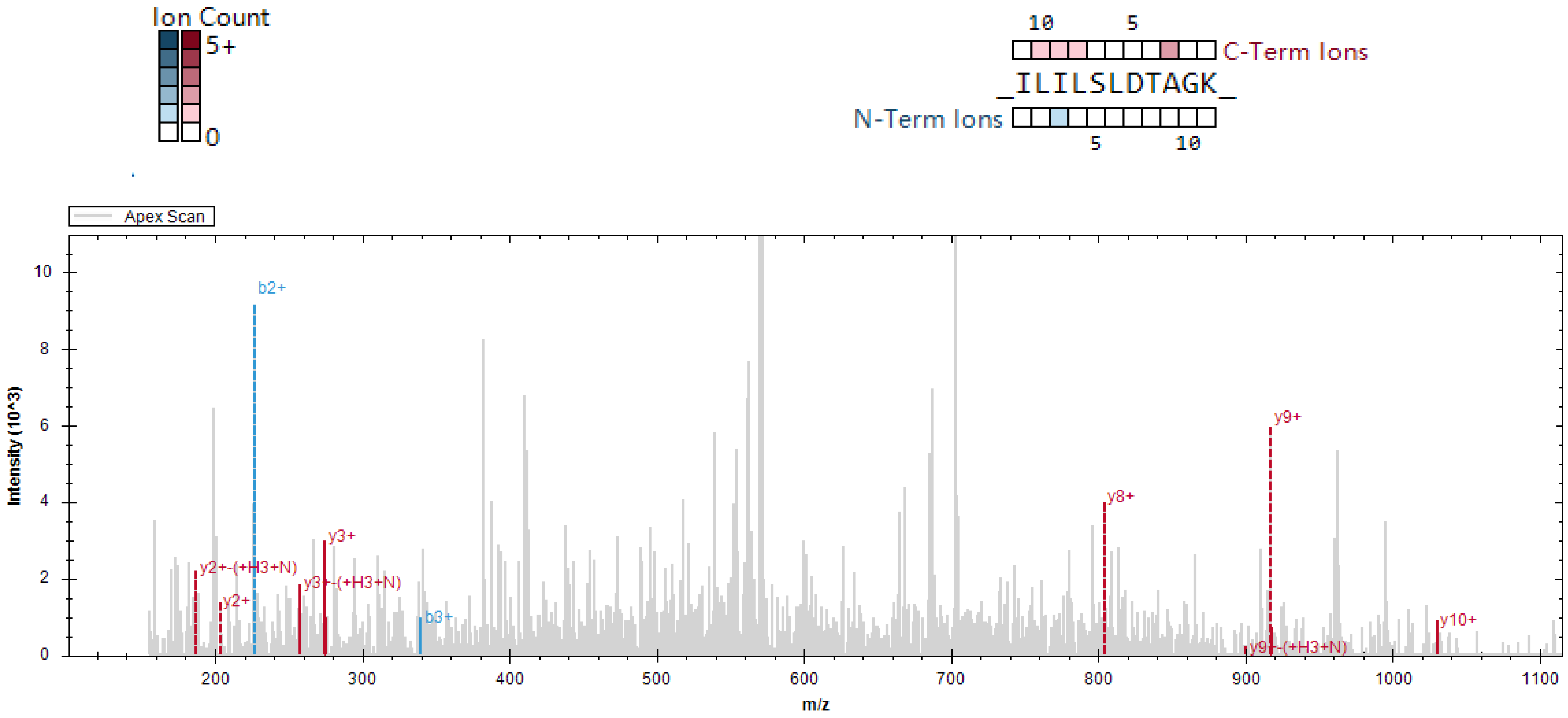

Ion Count
5+
0
10
5
C-Term Ions
_ILILSLDTAGK_
N-Term Ions
5
10
Apex Scan
Intensity (10^3)
m/z
b2+
b3+
y2+-(+H3+N)
y2+
y3+
y3+-(+H3+N)
y8+
y9+
y9+-(+H3+N)
y10+

Q8IWF9

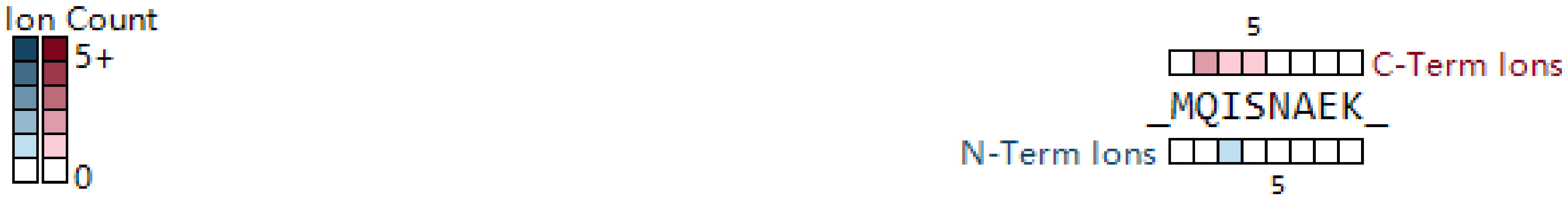
Ion Count
5+
0
5
C-Term Ions
_MQISNAEK_
N-Term Ions
5

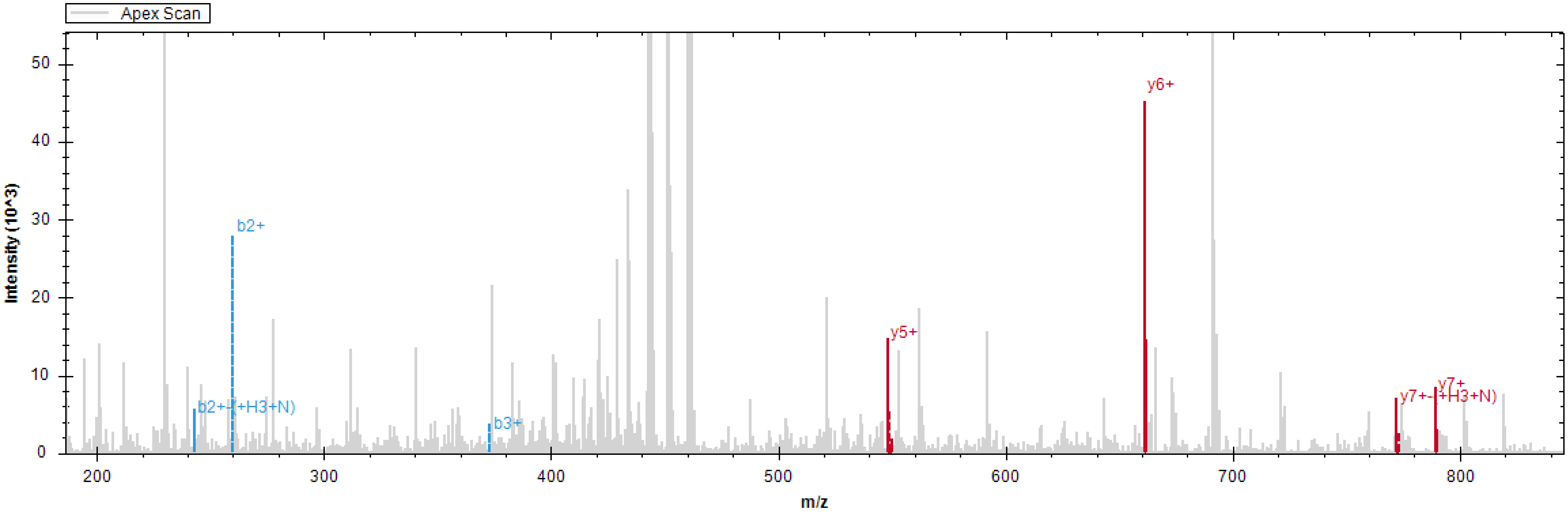
Apex Scan
Intensity (10^3)
m/z
b2+
b2+-(+H3+N)
b3+
y5+
y6+
y7+-(+H3+N)
y7+

Q8IWU2

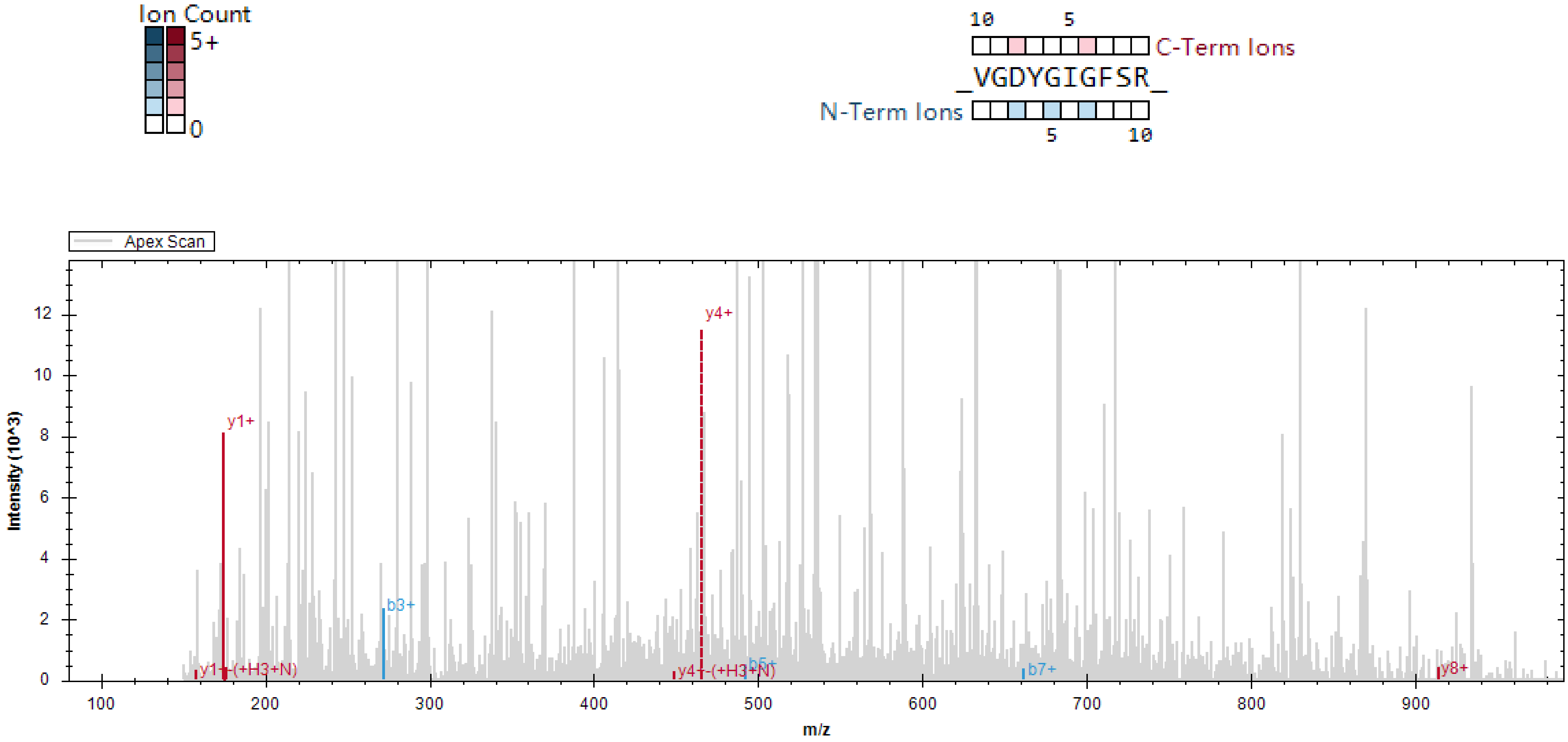

Ion Count
5+
0
10
5
C-Term Ions
_VGDYGIGFSR_
N-Term Ions
5
10
Apex Scan
Intensity (10^3)
m/z
y1+
y4+
b3+
y1+-(+H3+N)
y4+-(+H3+N)
b5+
b7+
y8+

# Q8IWY4

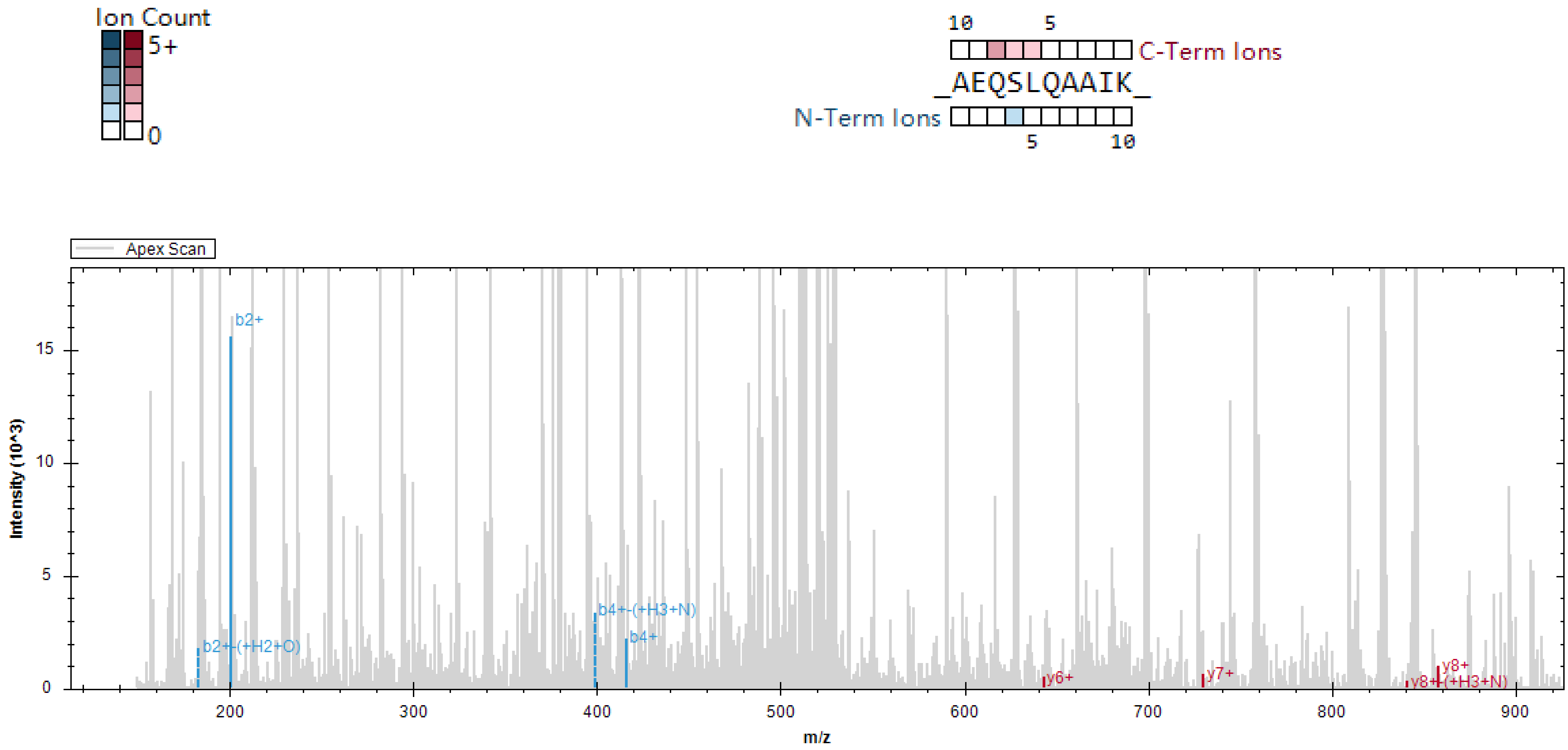

Ion Count
5+
0
10
5
C-Term Ions
_AEQSLQAAIK_
N-Term Ions
5
10
Apex Scan
Intensity (10^3)
m/z
b2+
b2+-(+H2+O)
b4+-(+H3+N)
b4+
y6+
y7+
y8+
y8+-(+H3+N)

Q8IWZ5

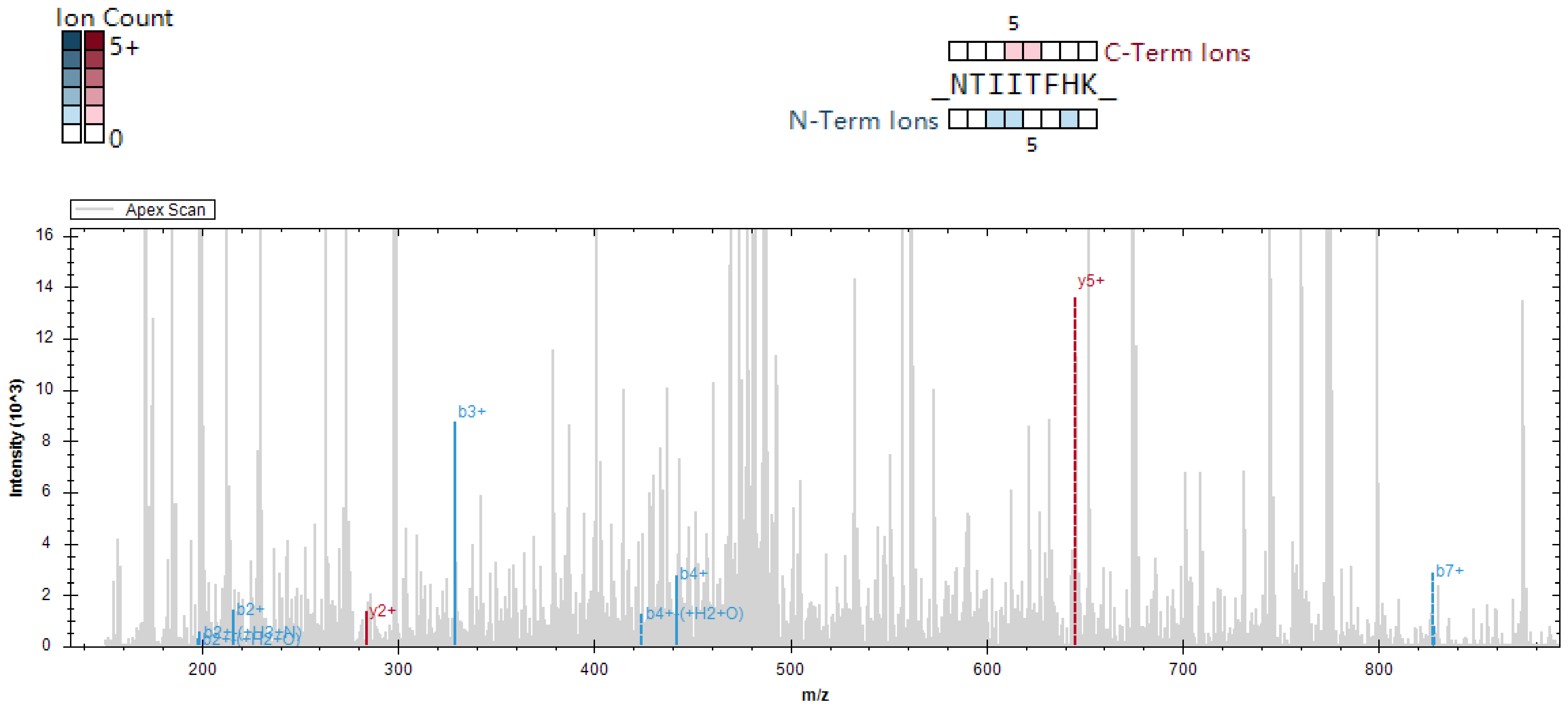

Ion Count
5+
0
5
C-Term Ions
_NTIITFHK_
N-Term Ions
5
Apex Scan
Intensity (10^3)
m/z
y5+
b3+
b4+
b4+-(+H2+O)
b2+
y2+
b7+

Q8IY26

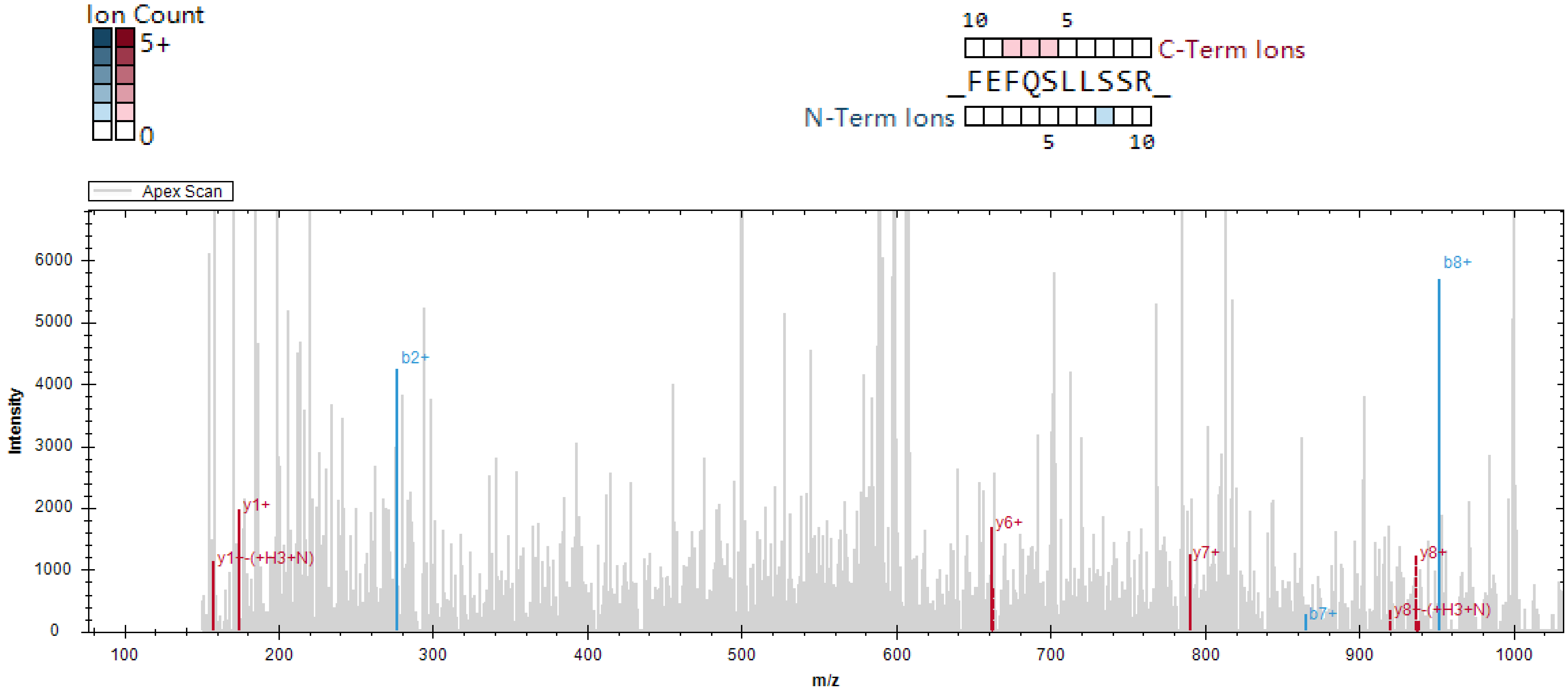

Ion Count
5+
0
10
5
C-Term Ions
_FEFQSLLSSR_
N-Term Ions
5
10
Apex Scan
Intensity
m/z
b2+
b8+
b7+
y1+
y1+-(+H3+N)
y6+
y7+
y8+
y8+-(+H3+N)

Q8IYB7

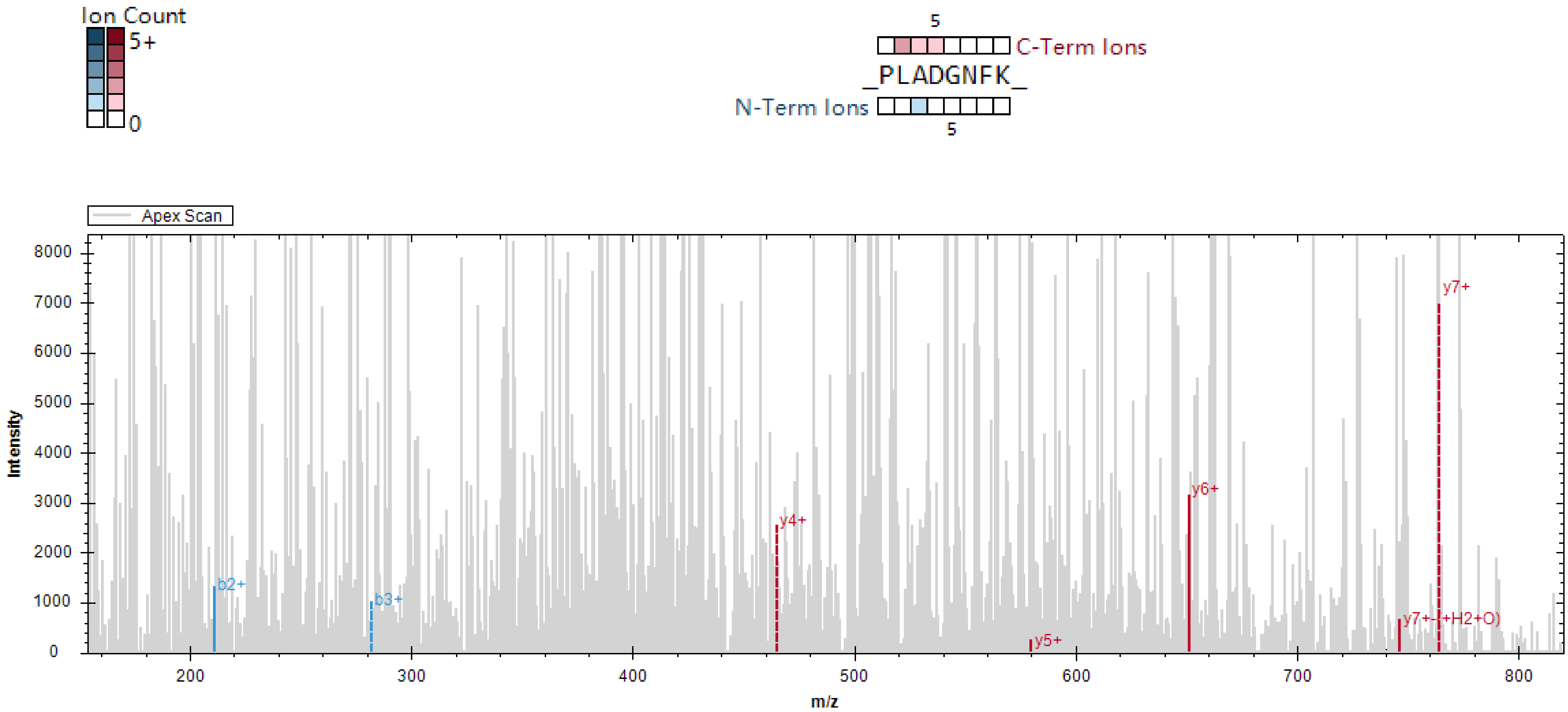

Ion Count
5+
0
5
C-Term Ions
_PLADGNFK_
N-Term Ions
5
Apex Scan
Intensity
m/z
b2+
b3+
y4+
y5+
y6+
y7+
y7+-(+H2+O)

Q8IYJ2

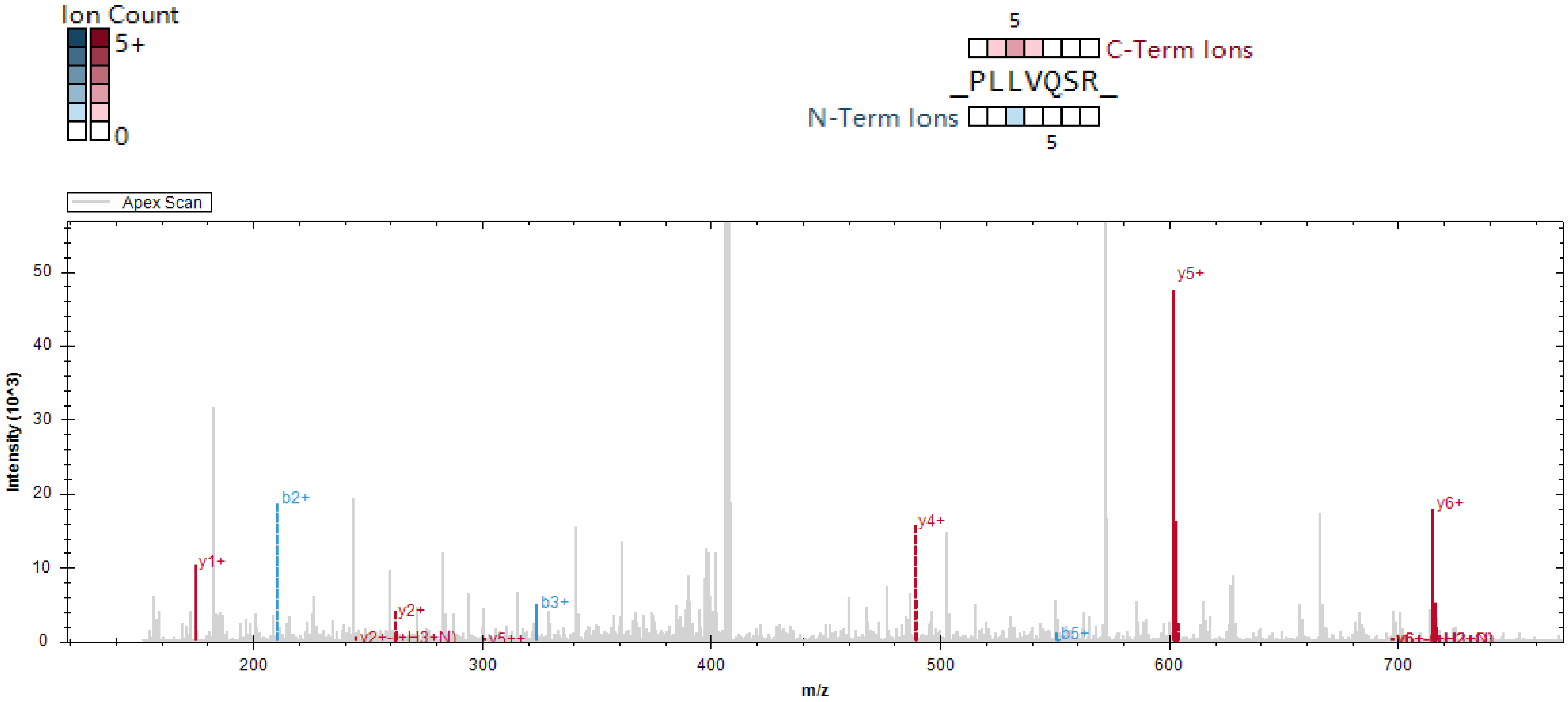

Ion Count
5+
0
5
C-Term Ions
_PLLVQSR_
N-Term Ions
5
Apex Scan
y5+
b2+
y4+
y6+
y1+
y2+
b3+
b5+
Intensity (10^3)
m/z
0
10
20
30
40
50
200
300
400
500
600
700

Q8IZF6

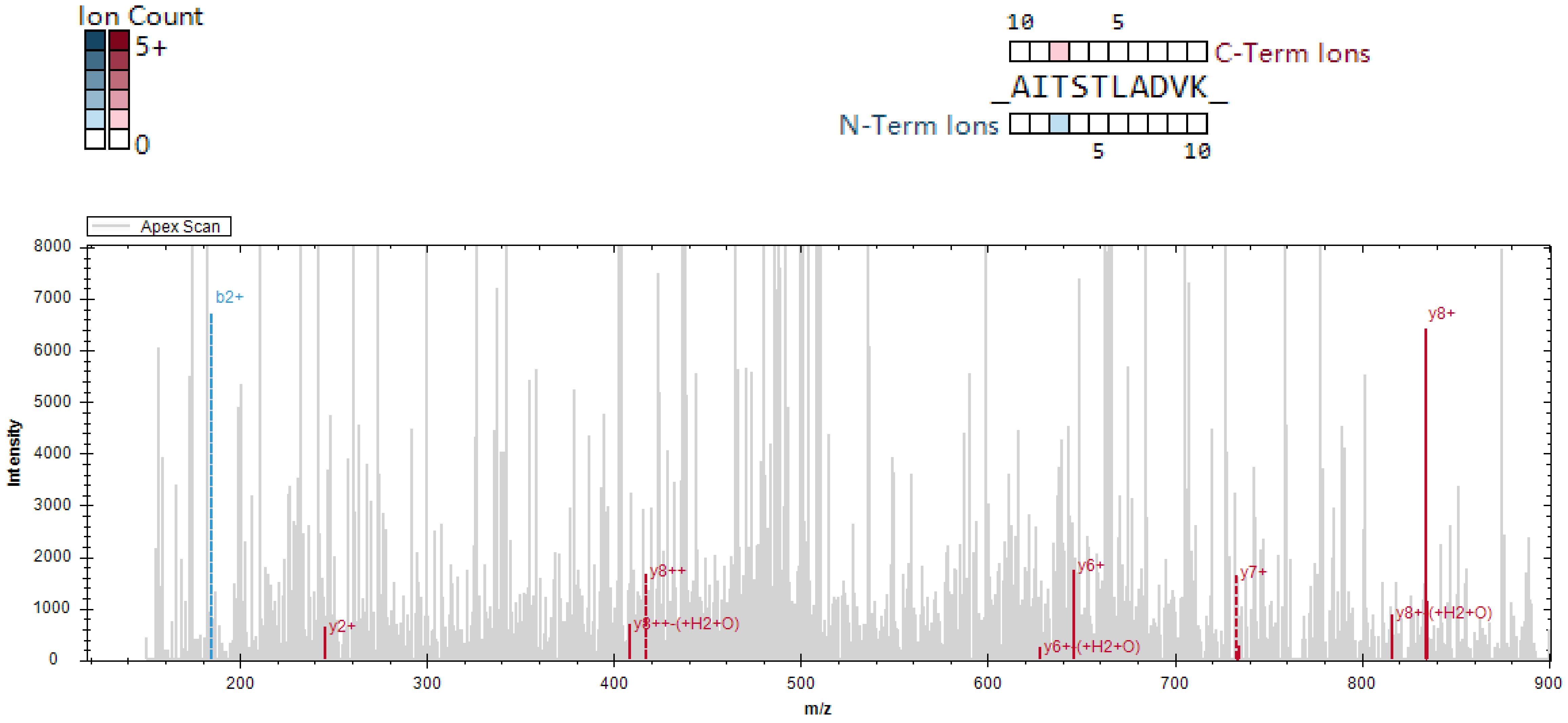
Ion Count
5+
0
10
5
C-Term Ions
_AITSTLADVK_
N-Term Ions
5
10
Apex Scan
b2+
y2+
y8++
y8++-(+H2+O)
y6+
y6+-(+H2+O)
y7+
y8+
y8+-(+H2+O)
Intensity
m/z
0
1000
2000
3000
4000
5000
6000
7000
8000
200
300
400
500
600
700
800
900

Q8N131

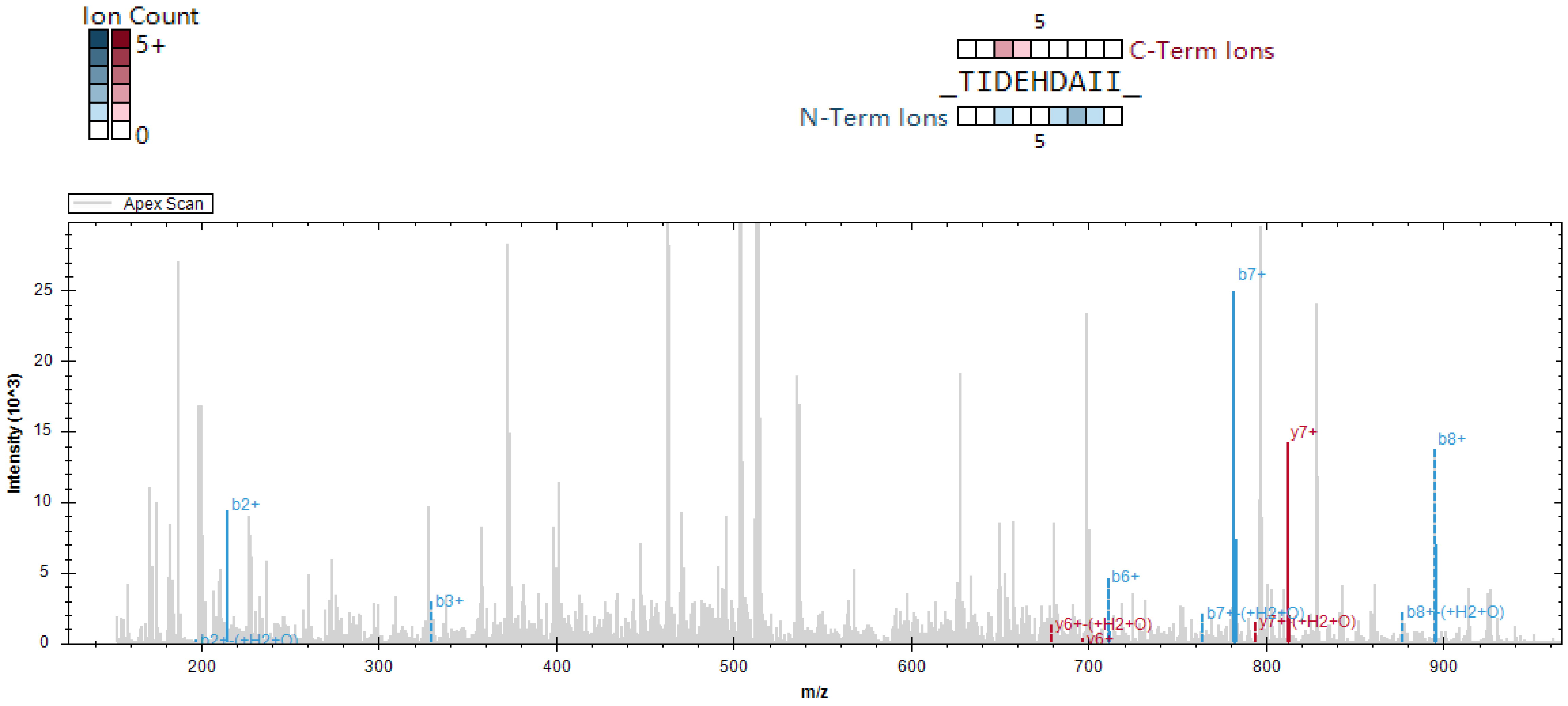

Ion Count
5+
0
5
C-Term Ions
_TIDEHDAII_
N-Term Ions
5
Apex Scan
Intensity (10^3)
m/z
b2+
b2+-(+H2+O)
b3+
b6+
b7+
b7+-(+H2+O)
y6+-(+H2+O)
y6+
y7+
y7+-(+H2+O)
b8+
b8+-(+H2+O)

# Q8N149

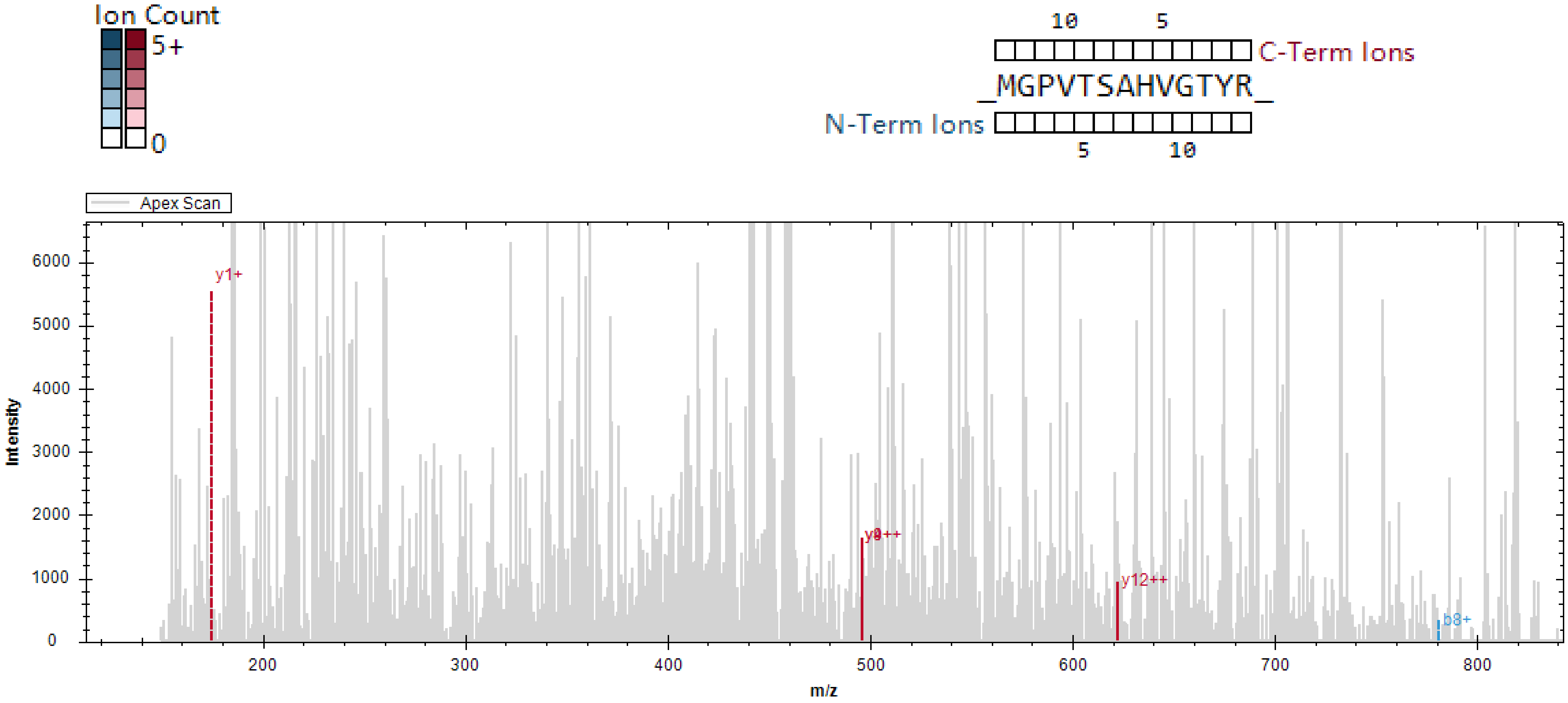

Ion Count
5+
0
10
5
C-Term Ions
_MGPVTSAHVGTYR_
N-Term Ions
5
10
Apex Scan
y1+
y8++
y12++
b8+
Intensity
m/z
0
1000
2000
3000
4000
5000
6000
200
300
400
500
600
700
800

Q8N4H5

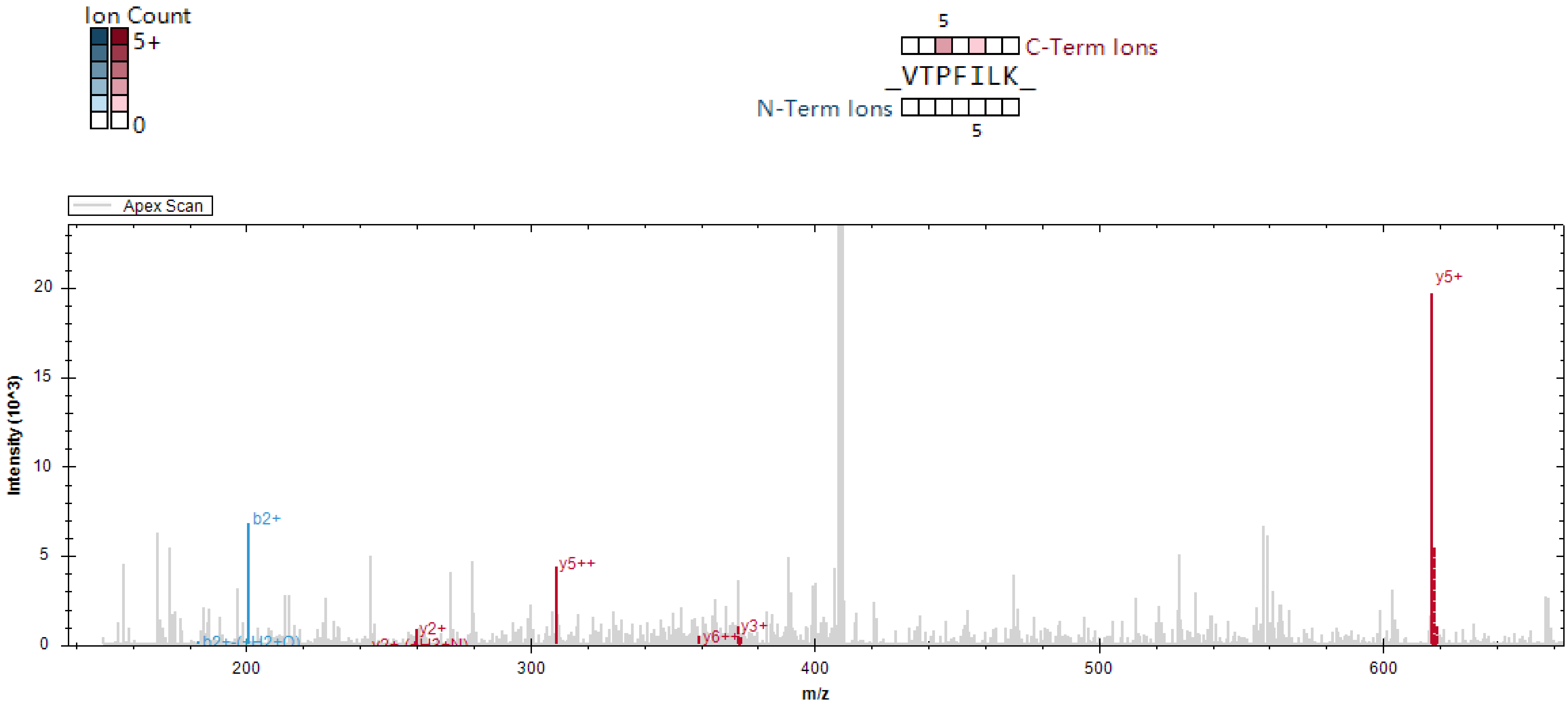

Ion Count
5+
0
5
C-Term Ions
_VTPFILK_
N-Term Ions
5
Apex Scan
Intensity (10^3)
m/z
y5+
b2+
y5++
y2+
y6++
y3+

Q8N4Q0

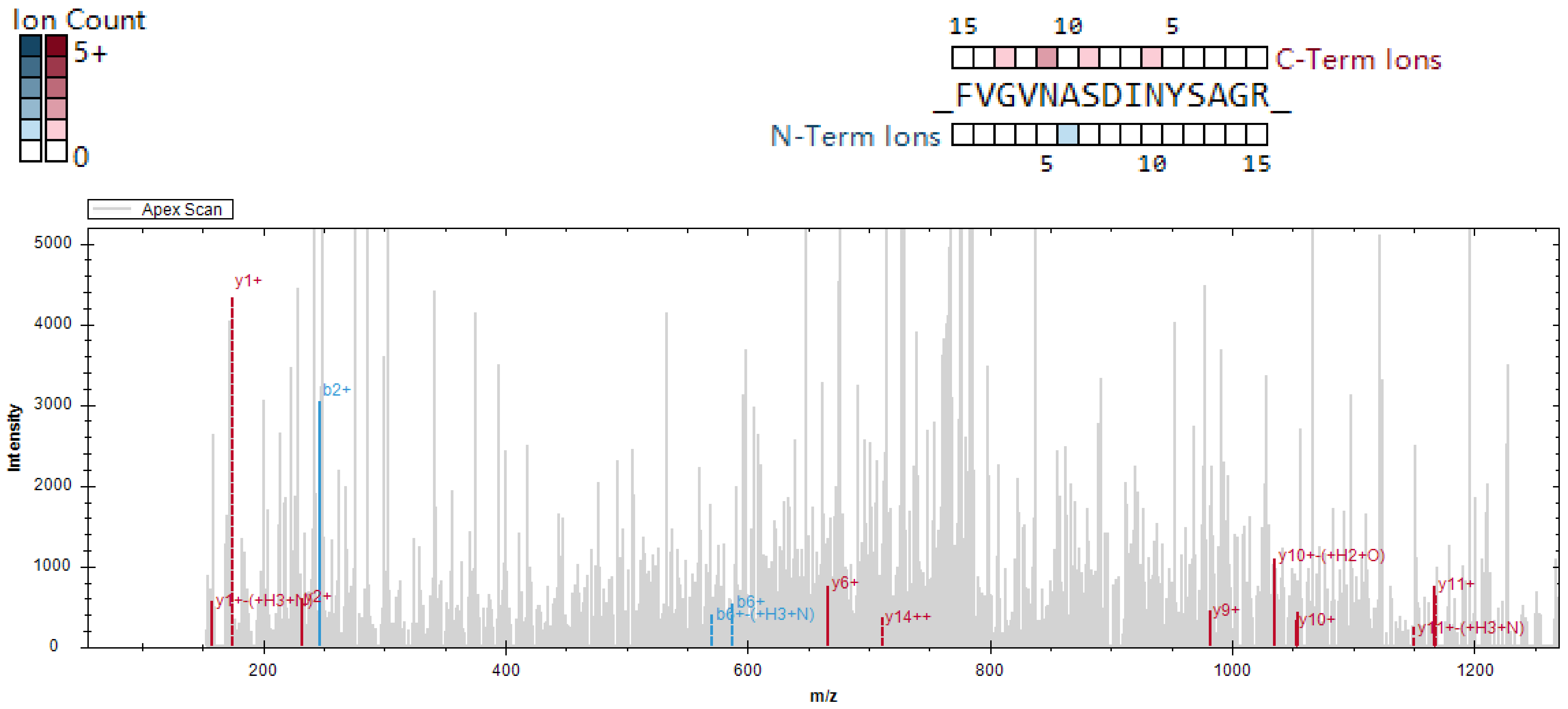

Ion Count
5+
0
15
10
5
C-Term Ions
_FVGVNASDINYSAGR_
N-Term Ions
5
10
15
Apex Scan
Intensity
m/z
y1+
b2+
y1+-(+H3+N)
y2+
b6+
b6+-(+H3+N)
y6+
y14++
y9+
y10+-(+H2+O)
y10+
y11+
y11+-(+H3+N)

Q8N4Q1

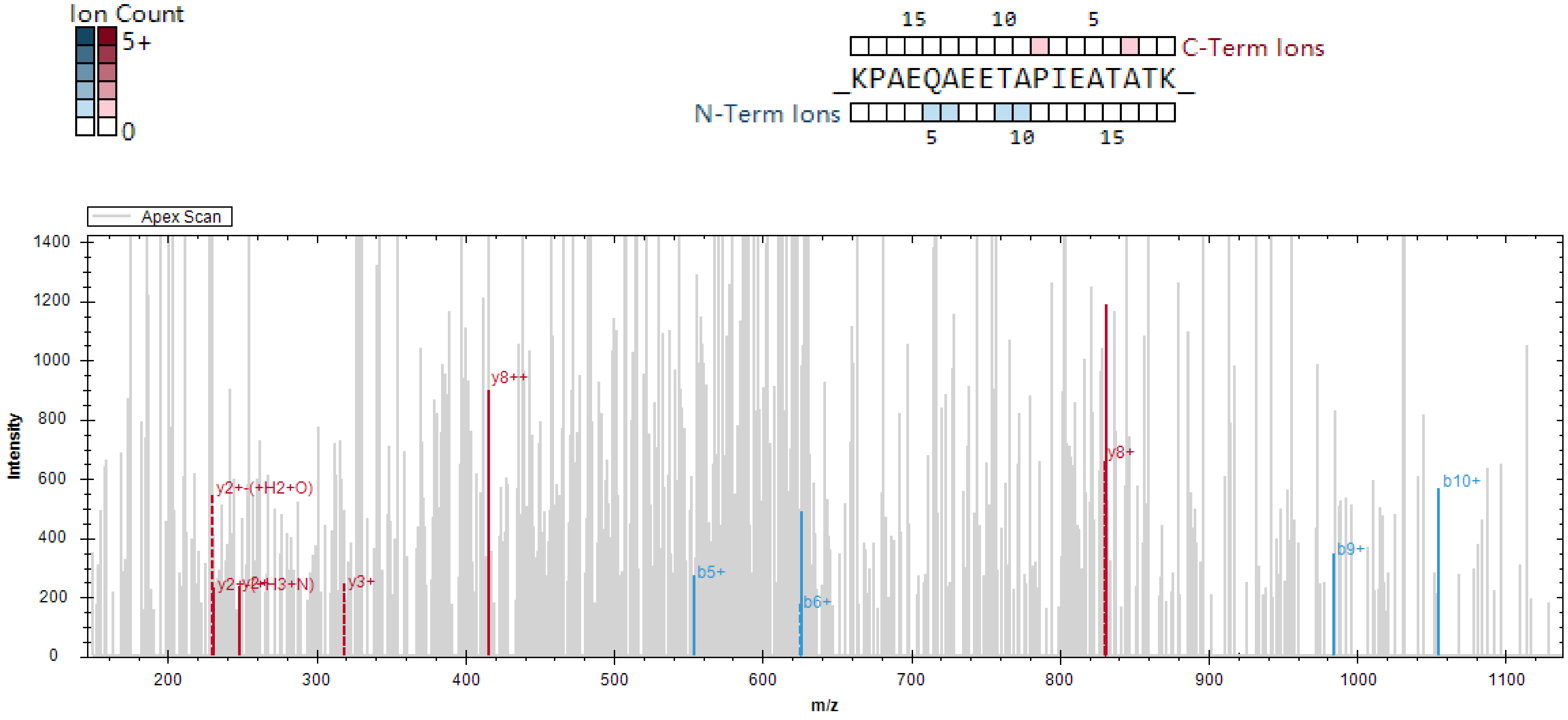

Ion Count
5+
0
15
10
5
C-Term Ions
_KPAEQAEETAPIEATATK_
N-Term Ions
5
10
15
Apex Scan
Intensity
m/z
y2+-(+H2+O)
y2+
y2+-(+H3+N)
y3+
y8++
y8+
b5+
b6+
b9+
b10+

Q8N5M1

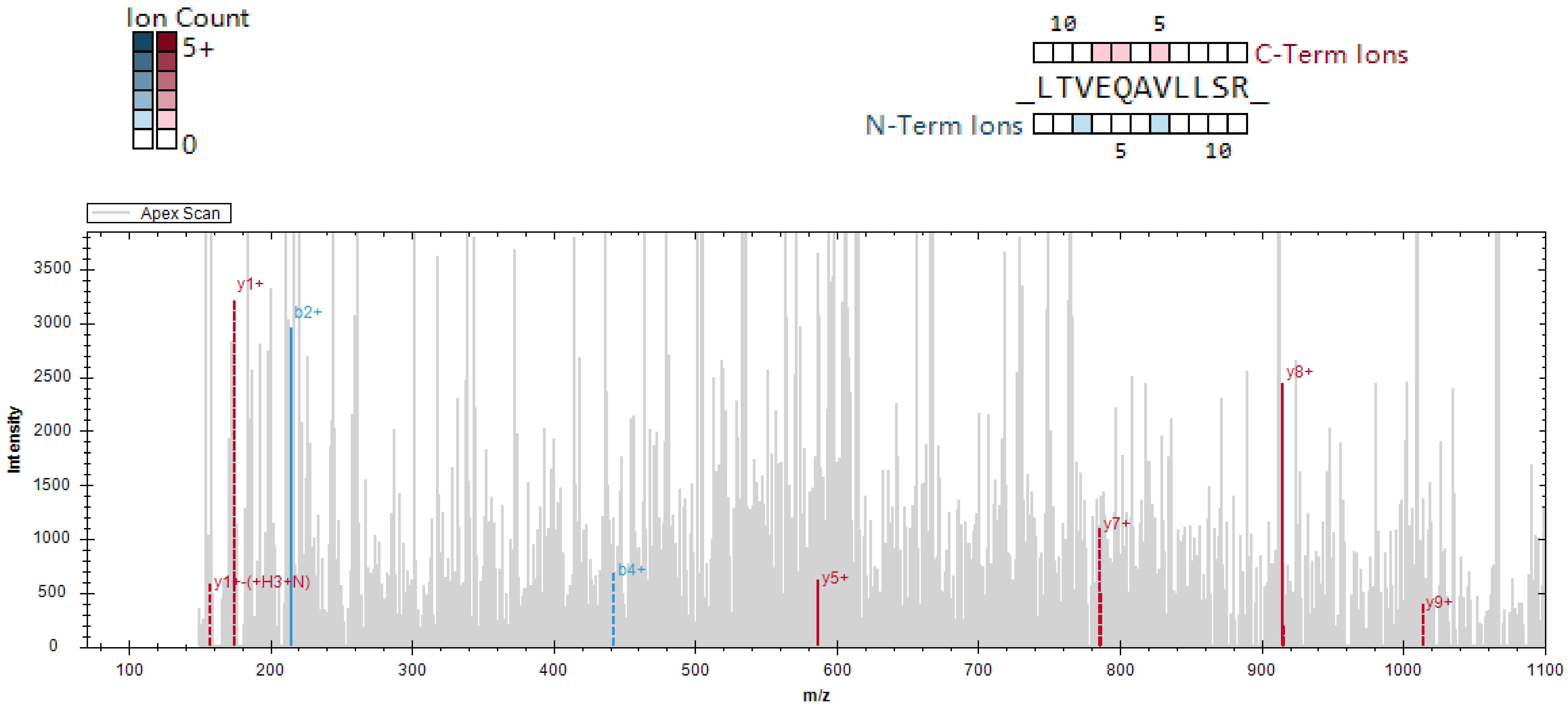

Ion Count
5+
0
10
5
C-Term Ions
_LTVEQAVLLSR_
N-Term Ions
5
10
Apex Scan
Intensity
m/z
y1+
b2+
y8+
y7+
y1+-(+H3+N)
b4+
y5+
y9+

Q8N5N7

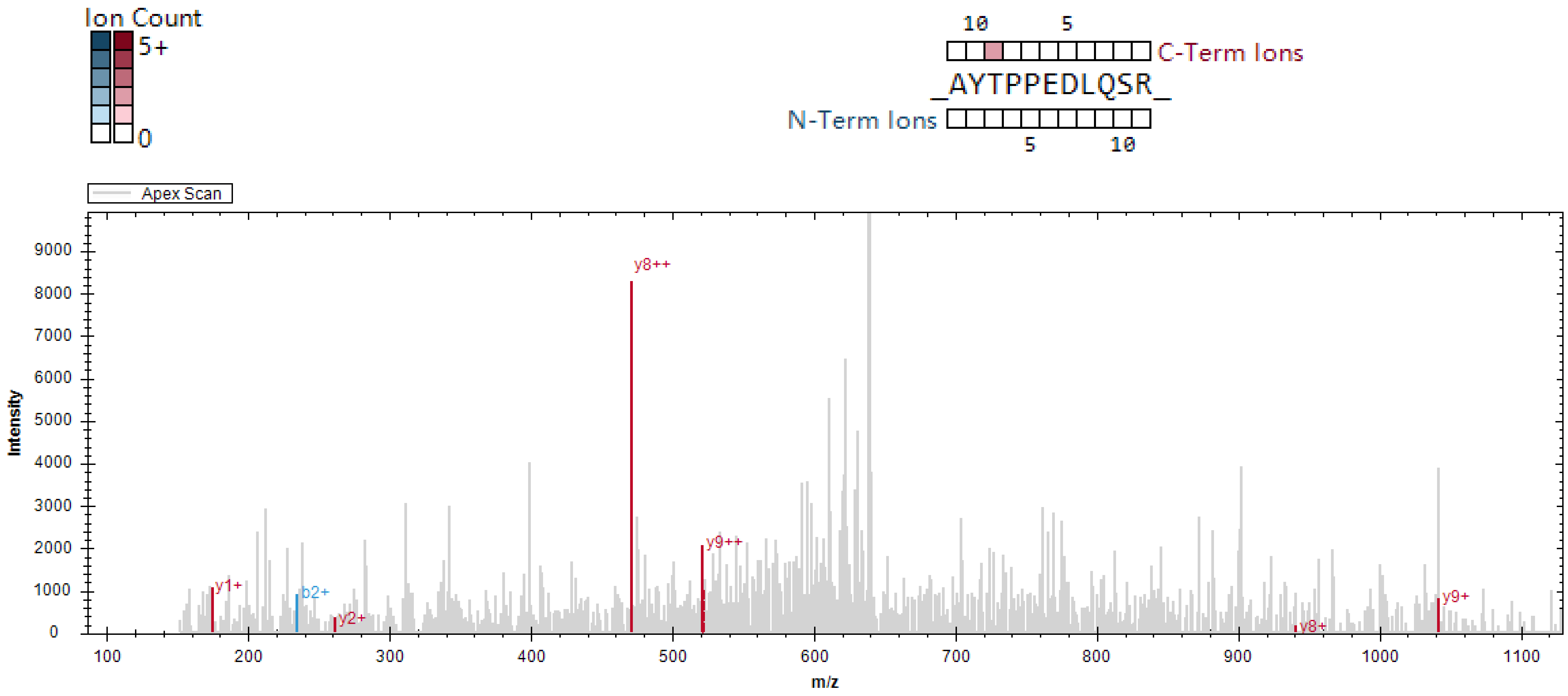

Ion Count
5+
0
10
5
C-Term Ions
_AYTPPEDLQSR_
N-Term Ions
5
10
Apex Scan
Intensity
m/z
y8++
y9++
y1+
b2+
y2+
y8+
y9+

Q8N7P1

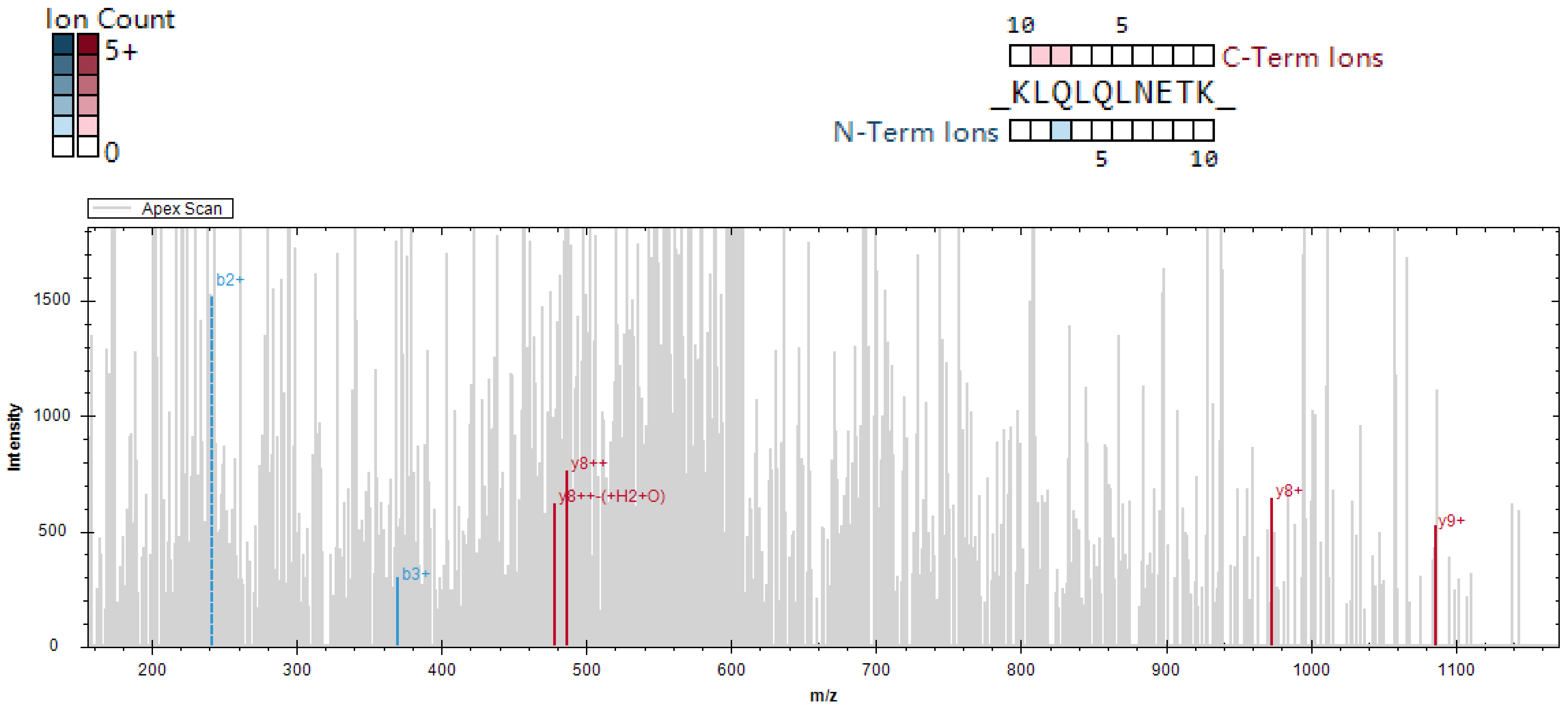

Ion Count
5+
0
10
5
C-Term Ions
_KLQLQLNETK_
N-Term Ions
5
10
Apex Scan
b2+
b3+
y8++
y8++-(+H2+O)
y8+
y9+
Intensity
1500
1000
500
0
200
300
400
500
600
700
800
900
1000
1100
m/z

Q8NAQ8

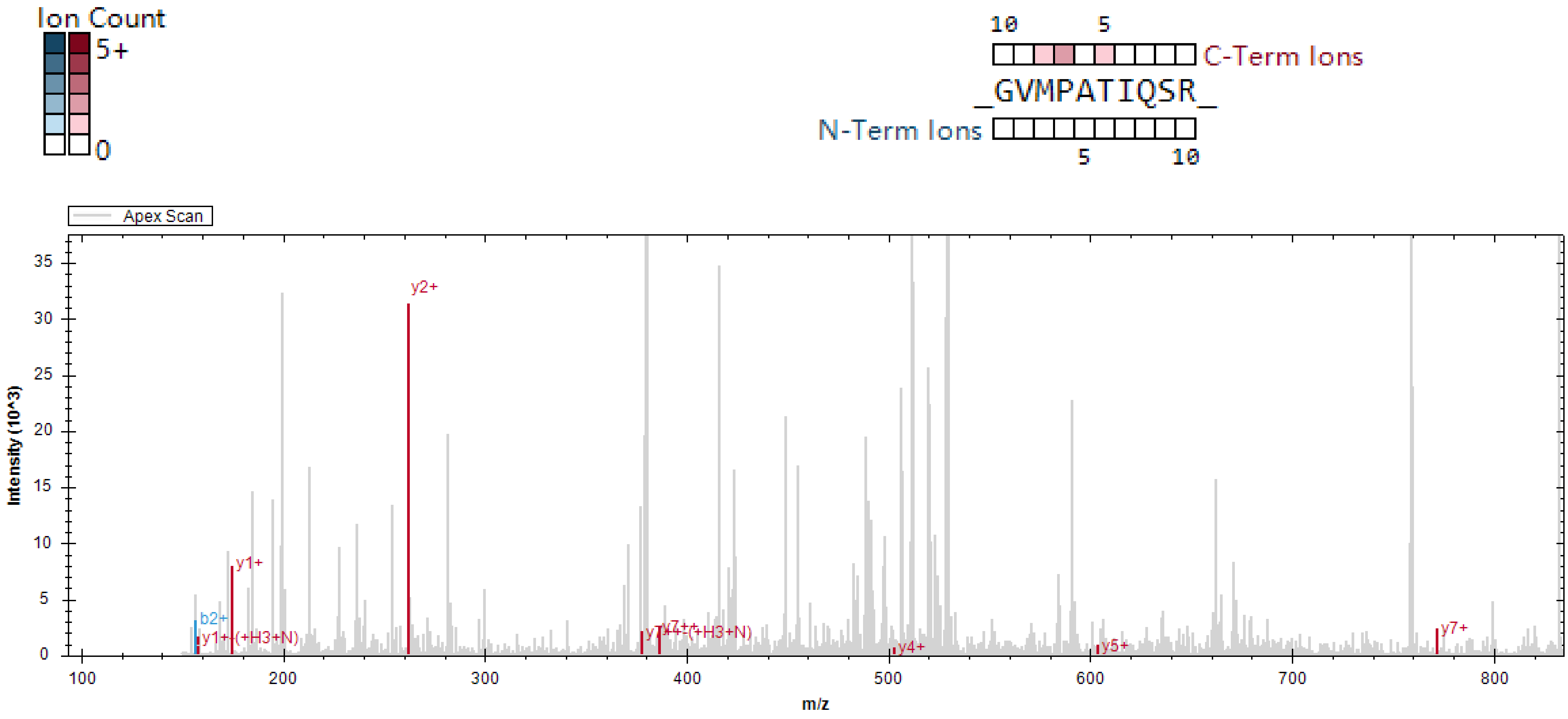
Ion Count
5+
0
10
5
C-Term Ions
_GVMPATIQSR_
N-Term Ions
5
10
Apex Scan
Intensity (10^3)
m/z
y2+
y1+
b2+
y1+-(+H3+N)
y7+-(+H3+N)
y4+
y5+
y7+

# Q8NAT1

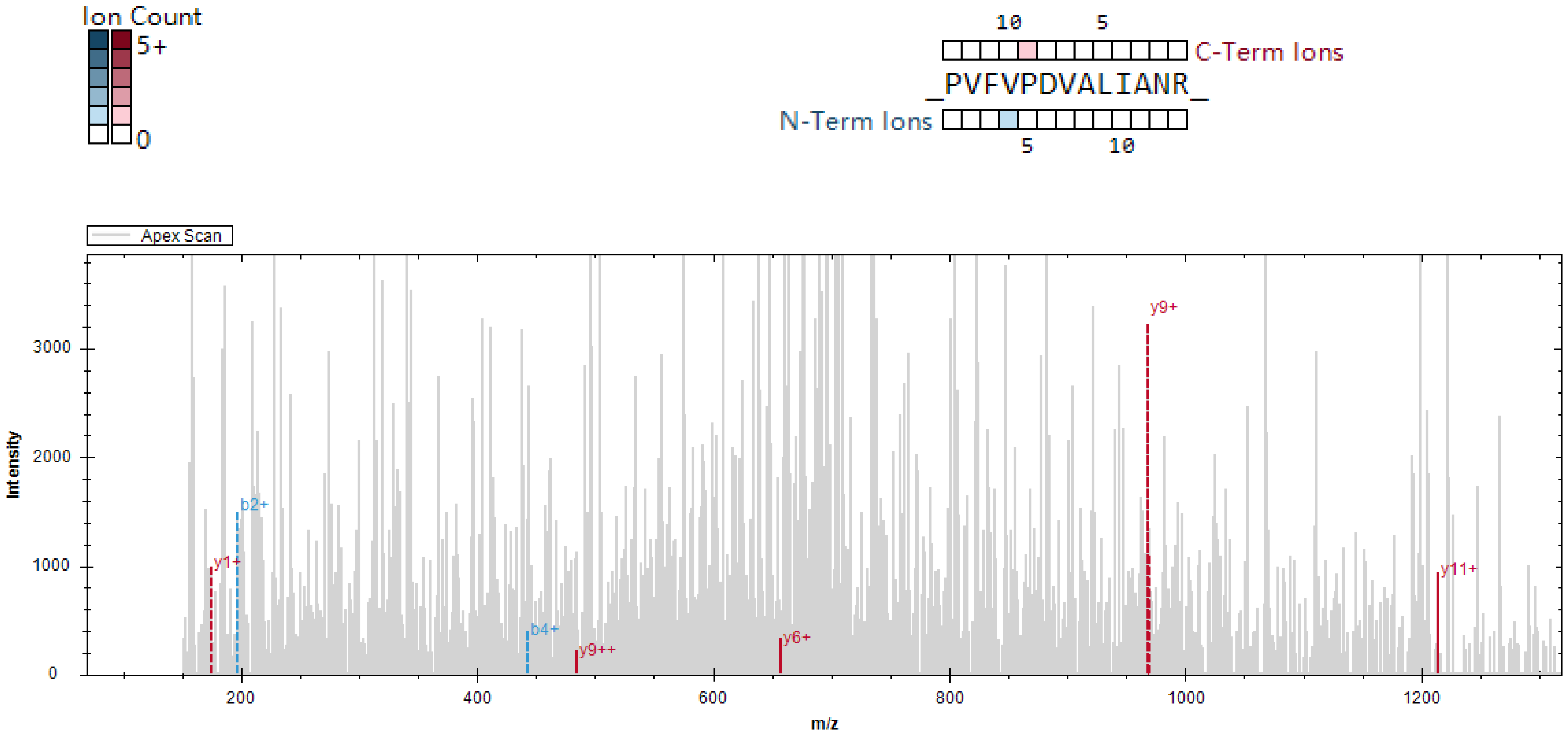

Ion Count
5+
0
10
5
C-Term Ions
_PVFVPDVALIANR_
N-Term Ions
5
10
Apex Scan
y9+
b2+
y1+
y11+
b4+
y9++
y6+
3000
2000
1000
0
Intensity
200
400
600
800
1000
1200
m/z

Q8NBF6

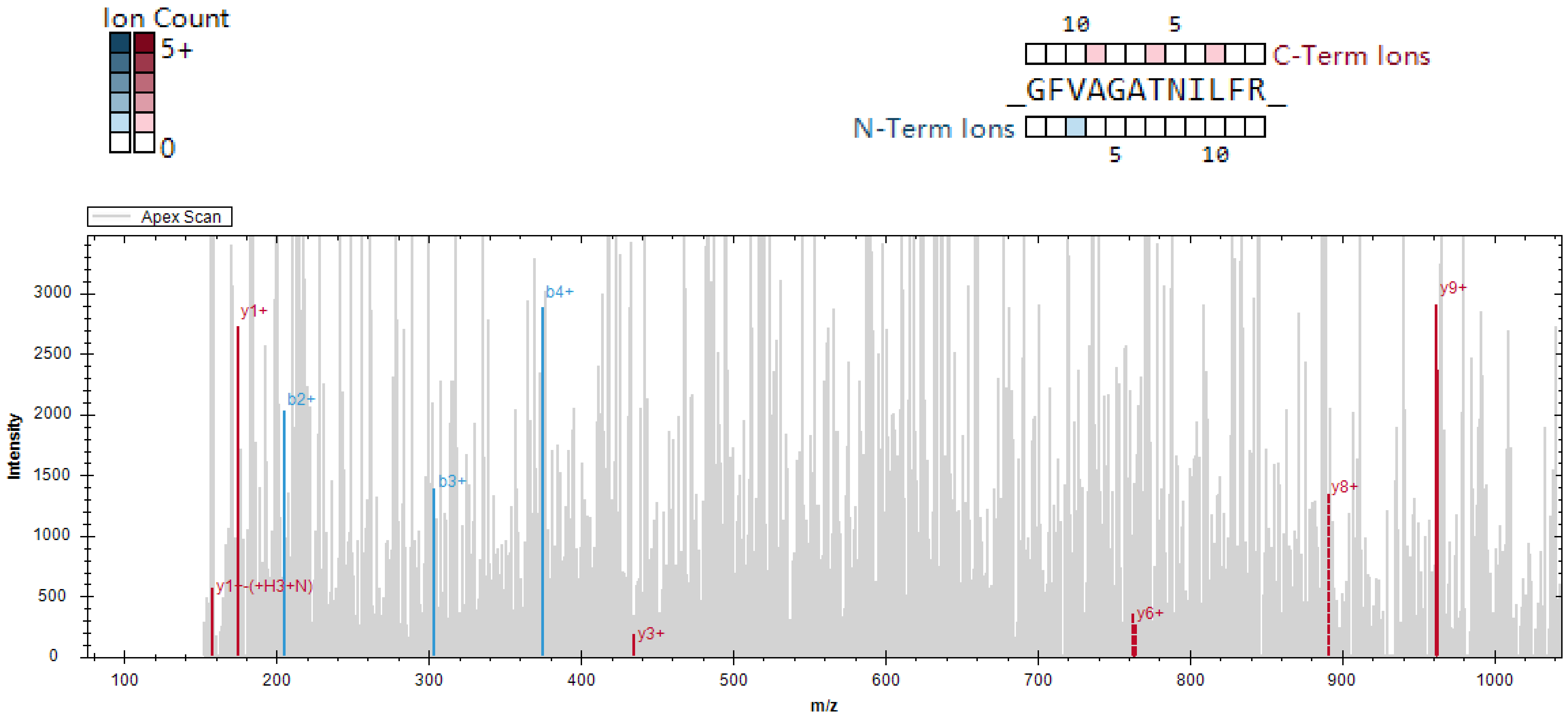

Ion Count
5+
0
10
5
C-Term Ions
_GFVAGATNILFR_
N-Term Ions
5
10
Apex Scan
y1+
y1+-(+H3+N)
b2+
b3+
b4+
y3+
y6+
y8+
y9+
Intensity
m/z

Q8NCD3

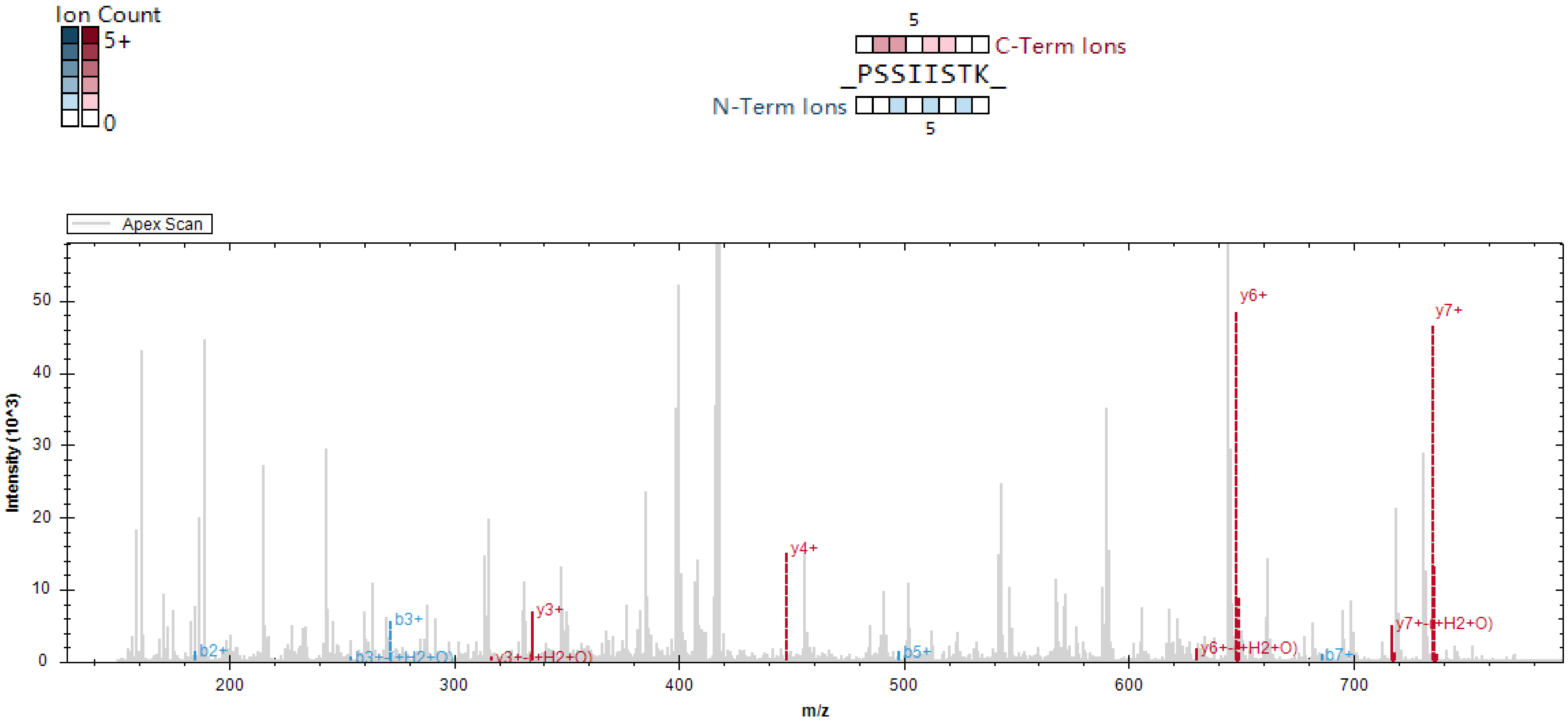

Ion Count
5+
0
5
C-Term Ions
_PSSIISTK_
N-Term Ions
5
Apex Scan
Intensity (10^3)
m/z
y6+
y7+
y4+
y3+
b3+
b2+
b5+
b7+
y7+-(+H2+O)
y6+-(+H2+O)

Q8NCU1

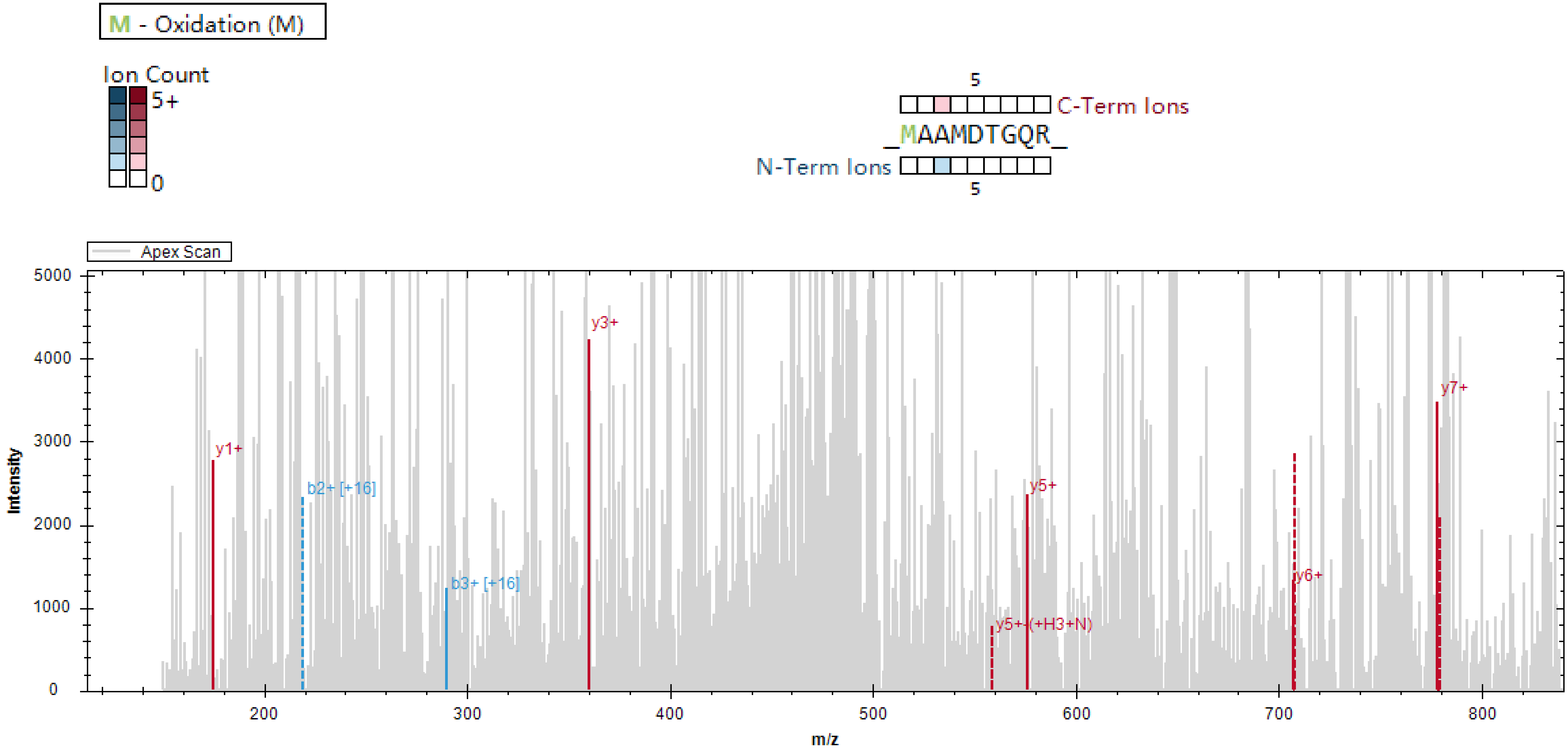

M - Oxidation (M)
Ion Count
5+
0
5
C-Term Ions
_MAAMDTGQR_
N-Term Ions
5
Apex Scan
Intensity
m/z
y1+
b2+ [+16]
b3+ [+16]
y3+
y5+-(+H3+N)
y5+
y6+
y7+

Q8NDC0

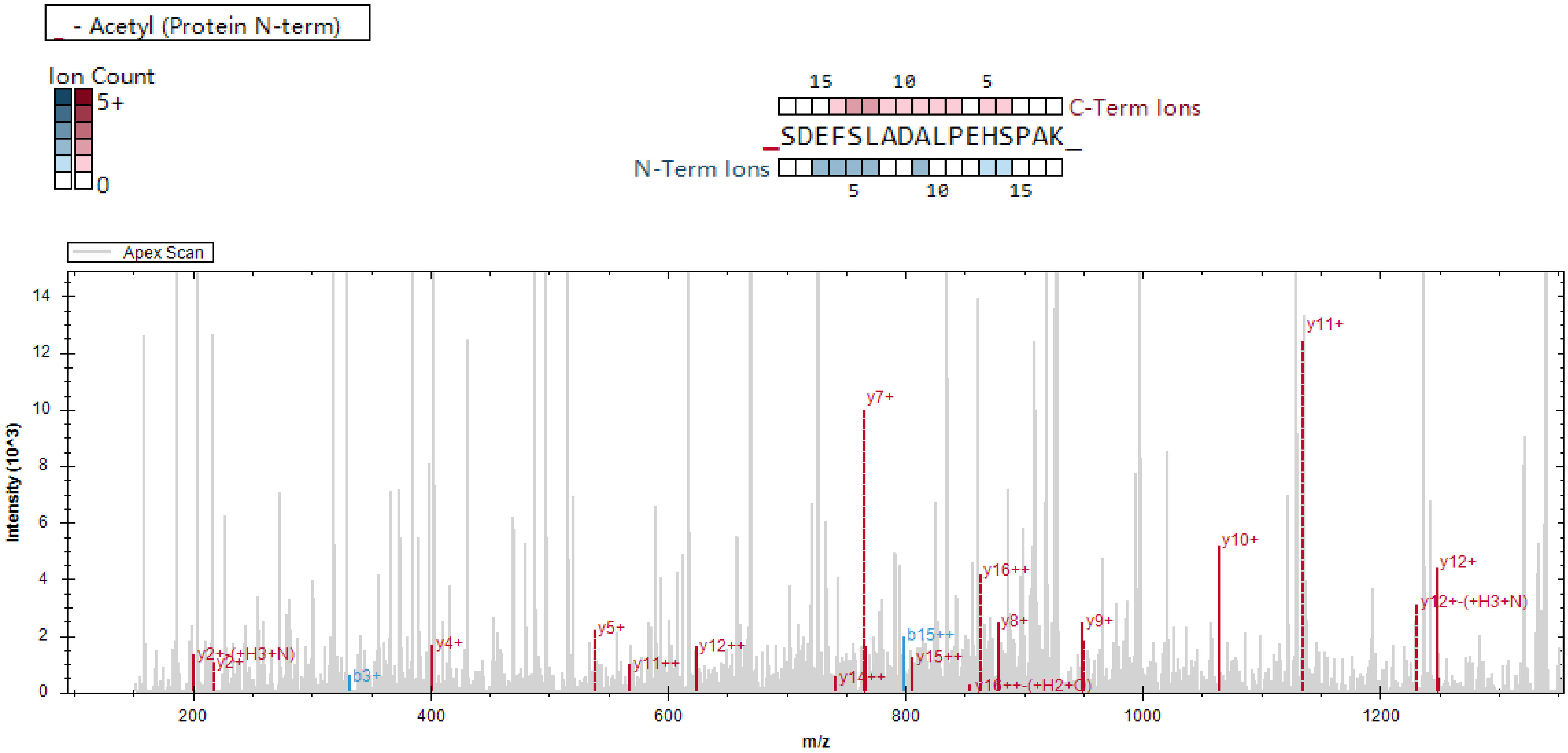

_ - Acetyl (Protein N-term)
Ion Count
5+
0
15 10 5
C-Term Ions
_SDEFSLADALPEHSPAK_
N-Term Ions
5 10 15
Apex Scan
Intensity (10^3)
m/z
y11+
y7+
y10+
y16++
y12+
y12+-(+H3+N)
y8+
y9+
b15++
y15++
y5+
y12++
y11++
y4+
y2+-(+H3+N)
y2+
b3+
y14++
y16++-(+H2+O)

Q8NES3

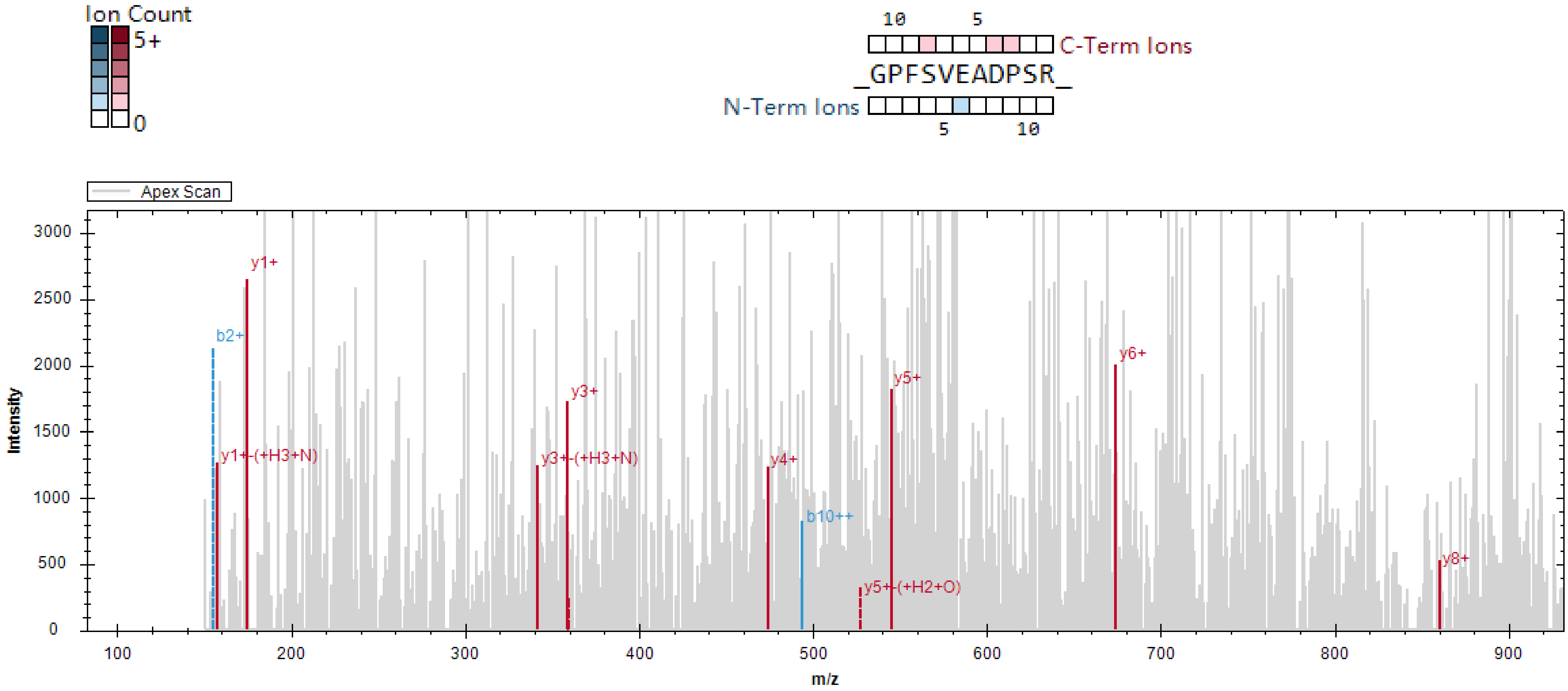

Ion Count
5+
0
10
5
C-Term Ions
_GPFSVEADPSR_
N-Term Ions
5
10
Apex Scan
y1+
b2+
y1+-(+H3+N)
y3+
y3+-(+H3+N)
y4+
b10++
y5+
y5+-(+H2+O)
y6+
y8+
Intensity
m/z
3000
2500
2000
1500
1000
500
0
100
200
300
400
500
600
700
800
900

Q8NFJ9

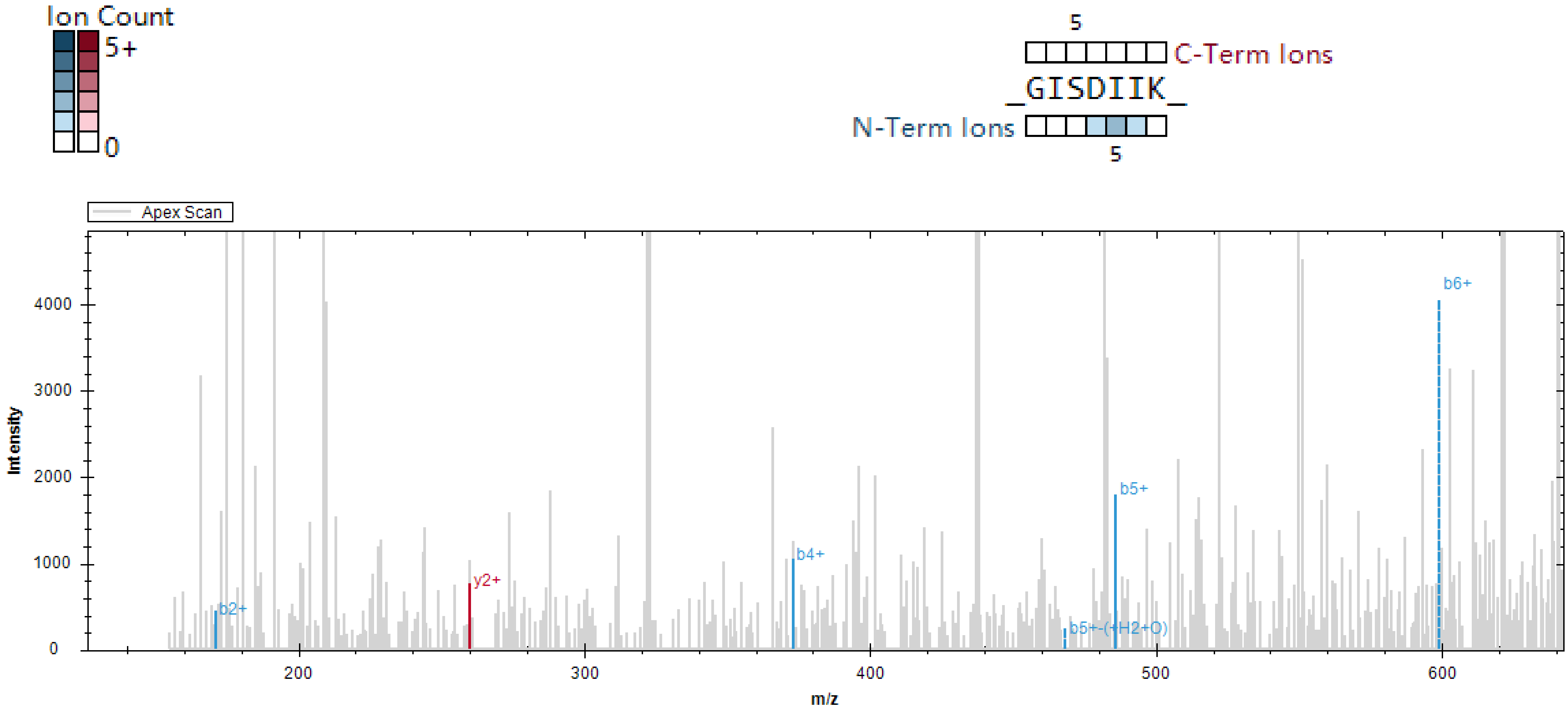

Ion Count
5+
0
5
C-Term Ions
_GISDIIK_
N-Term Ions
5
Apex Scan
Intensity
m/z
b2+
y2+
b4+
b5+-(+H2+O)
b5+
b6+

# Q8NFU3

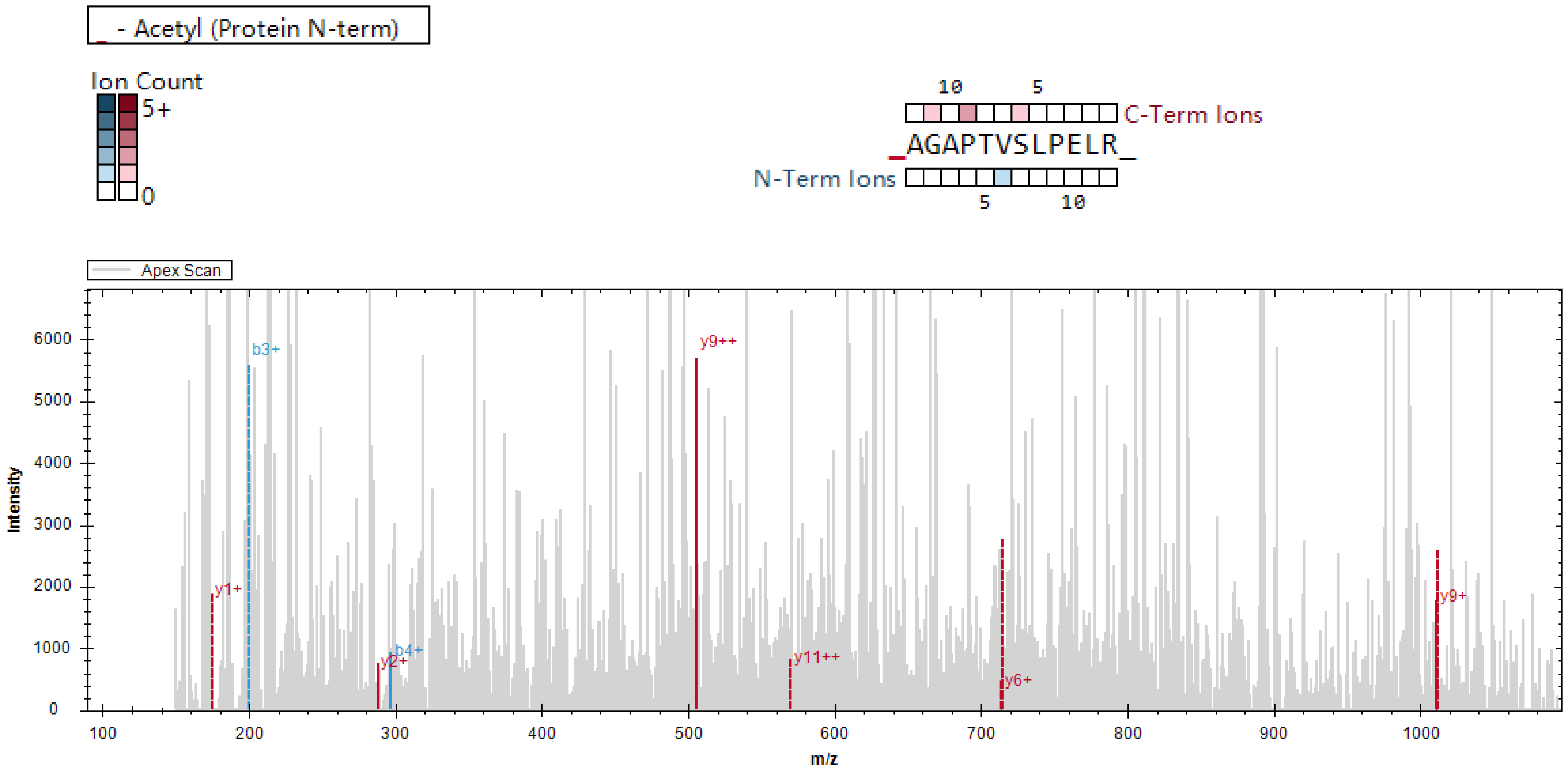
_ - Acetyl (Protein N-term)
Ion Count
5+
0
10
5
C-Term Ions
_AGAPTVSLPELR_
N-Term Ions
5
10
Apex Scan
Intensity
6000
5000
4000
3000
2000
1000
0
b3+
y9++
y1+
b4+
y2+
y11++
y6+
y9+
100
200
300
400
500
600
700
800
900
1000
m/z

# Q8TC44

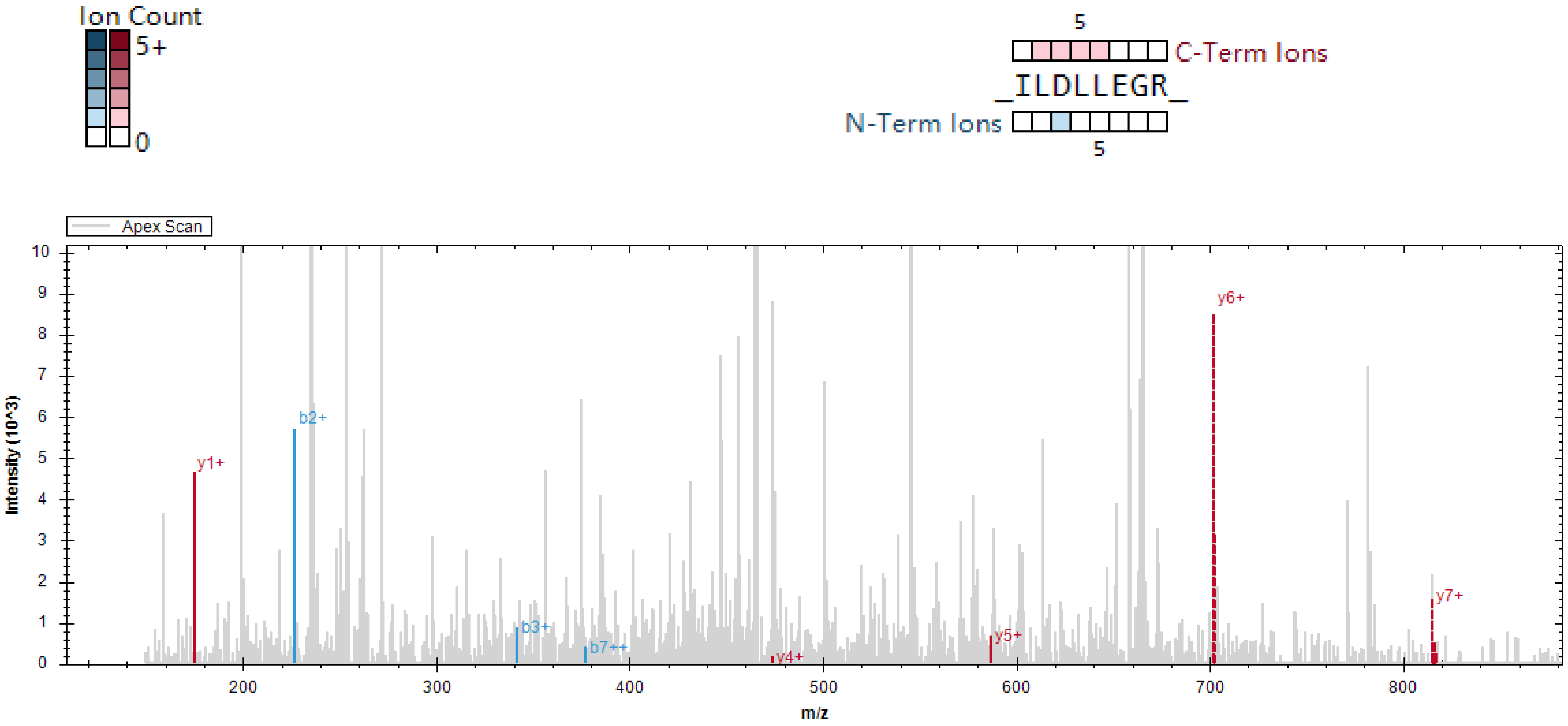

Ion Count
5+
0
5
C-Term Ions
_ILDLLEGR_
N-Term Ions
5
Apex Scan
Intensity (10^3)
m/z
y1+
b2+
b3+
b7++
y4+
y5+
y6+
y7+

Q8TDB8

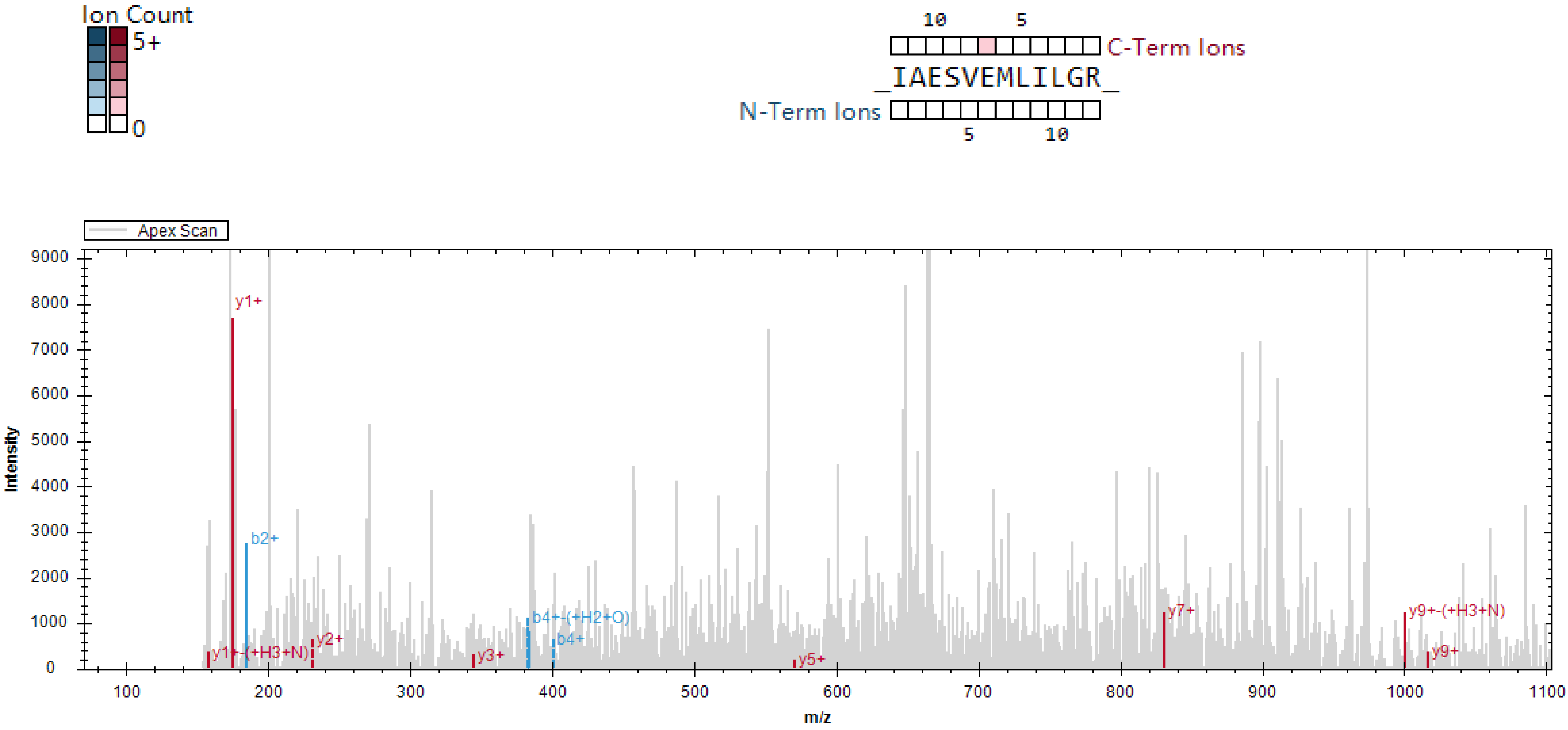

Ion Count
5+
0
10
5
C-Term Ions
_IAESVEMLILGR_
N-Term Ions
5
10
Apex Scan
Intensity
m/z
y1+
b2+
y1+-(+H3+N)
y2+
y3+
b4+-(+H2+O)
b4+
y5+
y7+
y9+-(+H3+N)
y9+

Q8WV19

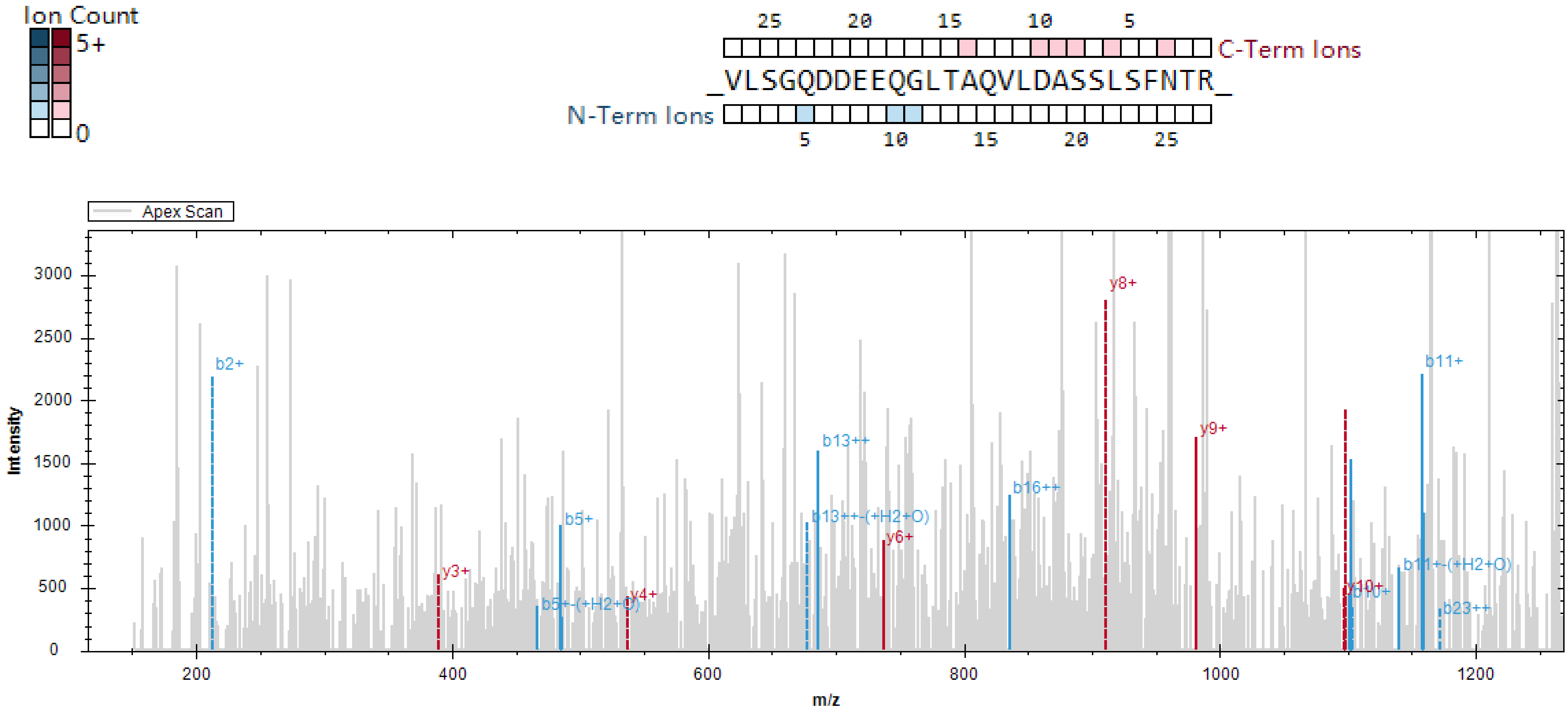

Ion Count
5+
0
C-Term Ions
_VLSGQDDEEQGLTAQVLDASSLSFNTR_
N-Term Ions
Apex Scan
Intensity
m/z
b2+
b5+
b5+-(+H2+O)
b13++
b13++-(+H2+O)
b16++
b11+
b11+-(+H2+O)
b23++
y3+
y4+
y6+
y8+
y9+
y10+

Q8WVX3

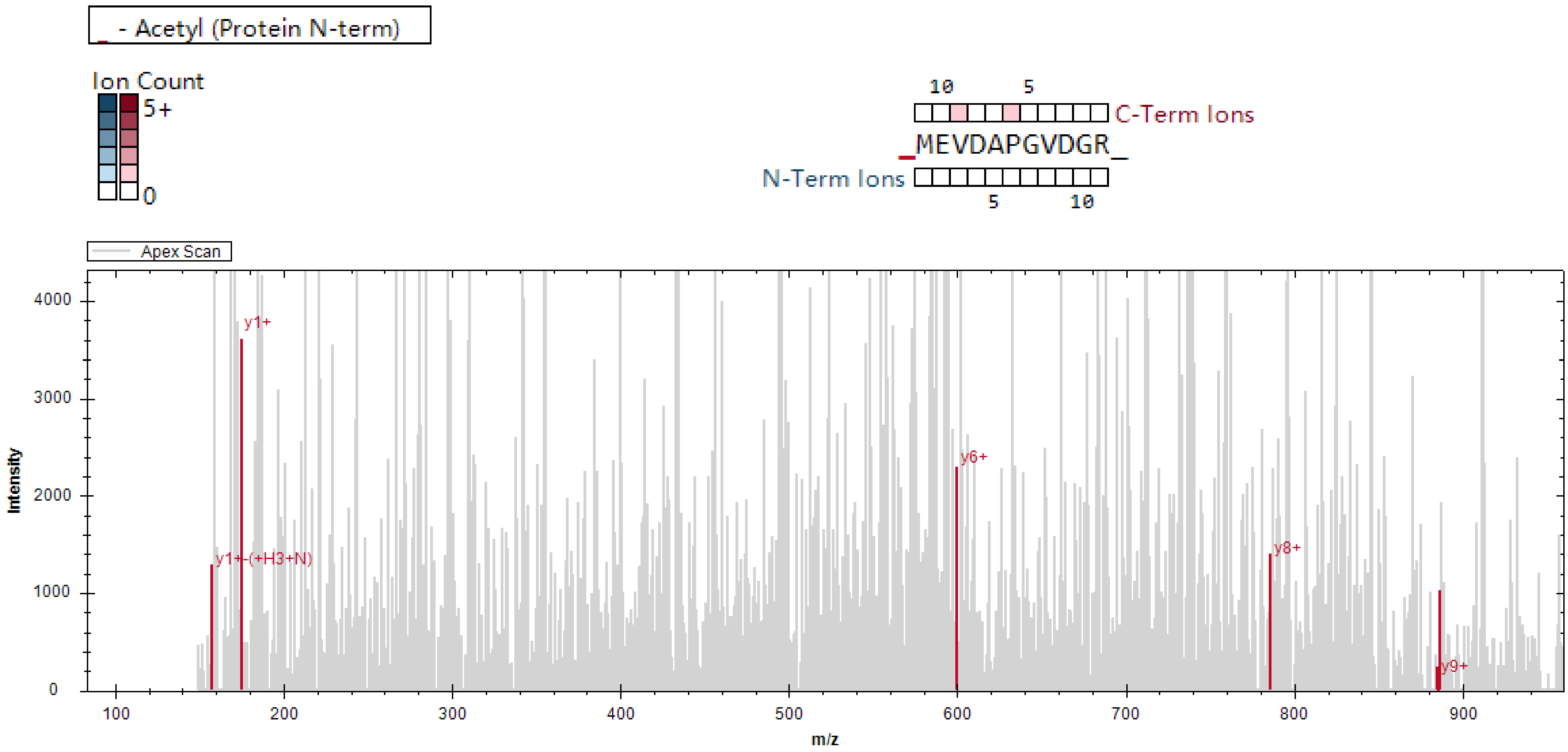
_ - Acetyl (Protein N-term)
Ion Count
5+
0
10 5
C-Term Ions
_MEVDAPGVDGR_
N-Term Ions
5 10
Apex Scan
y1+
y1+-(+H3+N)
y6+
y8+
y9+
Intensity
m/z
0
1000
2000
3000
4000
100
200
300
400
500
600
700
800
900

Q92526

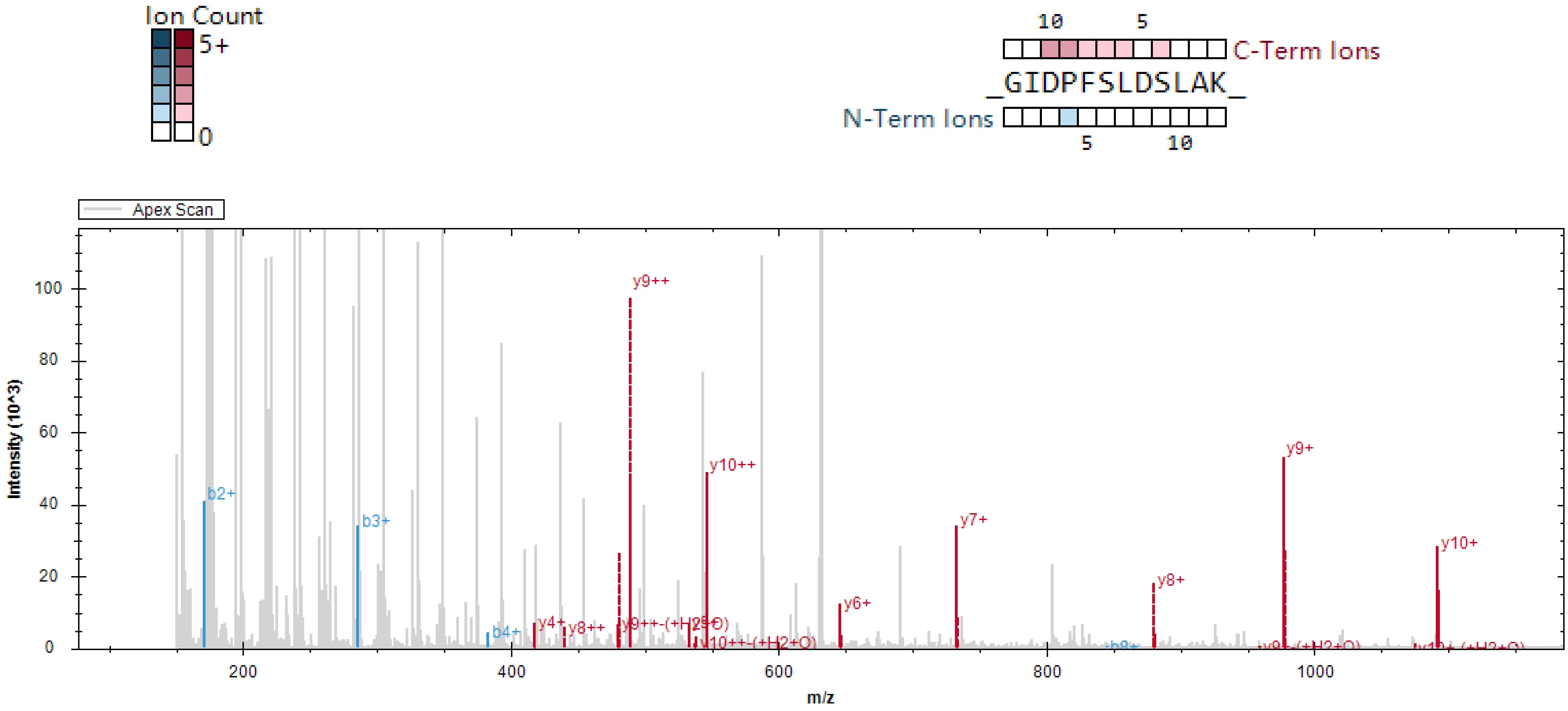

Ion Count
5+
0
10
5
C-Term Ions
_GIDPFSLDSLAK_
N-Term Ions
5
10
Apex Scan
Intensity (10^3)
m/z
b2+
b3+
b4+
y4+
y8++
y9++
y9++-(+H2O)
y10++
y10++-(+H2O)
y6+
y7+
y8+
y9+
y10+

Q92839

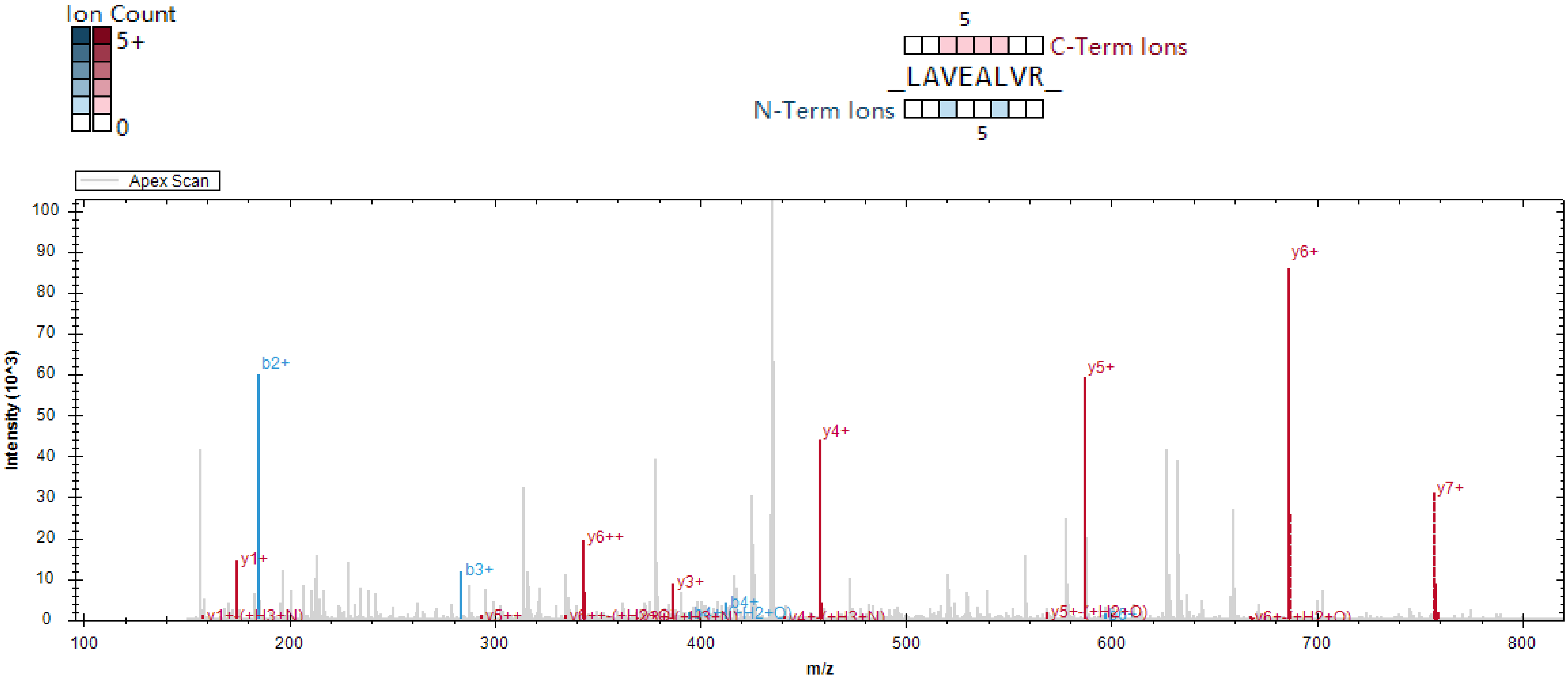

Ion Count
5+
0
5
C-Term Ions
_LAVEALVR_
N-Term Ions
5
Apex Scan
Intensity (10^3)
m/z
b2+
b3+
y1+
y6++
y3+
y4+
y5+
y6+
y7+

Q92928

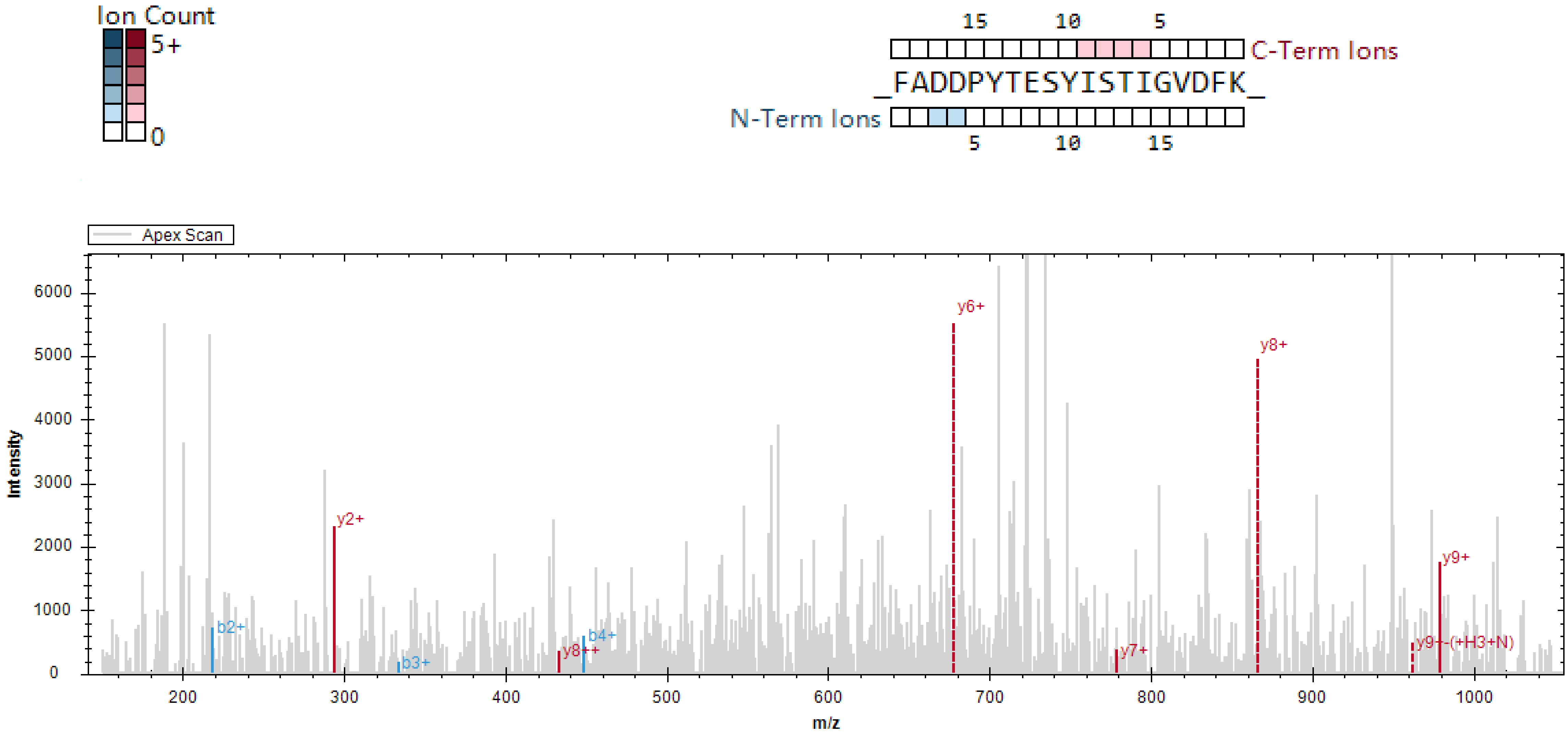

Ion Count
5+
0
15 10 5
C-Term Ions
_FADDPYTESYISTIGVDFK_
N-Term Ions
5 10 15
Apex Scan
Intensity
m/z
y6+
y8+
y2+
y9+
b2+
b3+
b4+
y8++
y7+
y9+-(+H3+N)

Q969S0

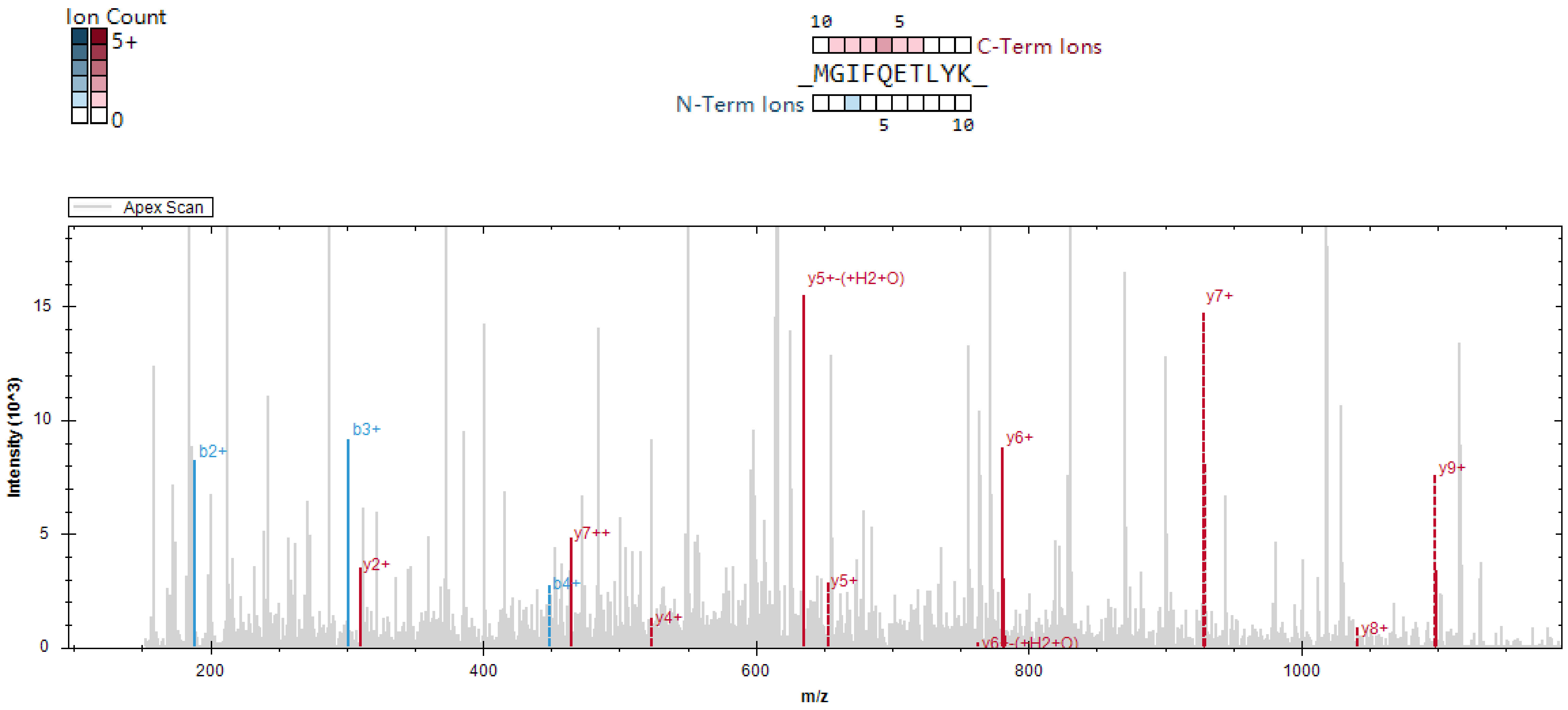

Ion Count
5+
0
10
5
C-Term Ions
_MGIFQETLYK_
N-Term Ions
5
10
Apex Scan
y5+-(+H2+O)
y7+
b3+
b2+
y6+
y9+
y7++
y2+
b4+
y5+
y4+
y8+
y6+-(+H2+O)
Intensity (10^3)
m/z

Q96AQ7

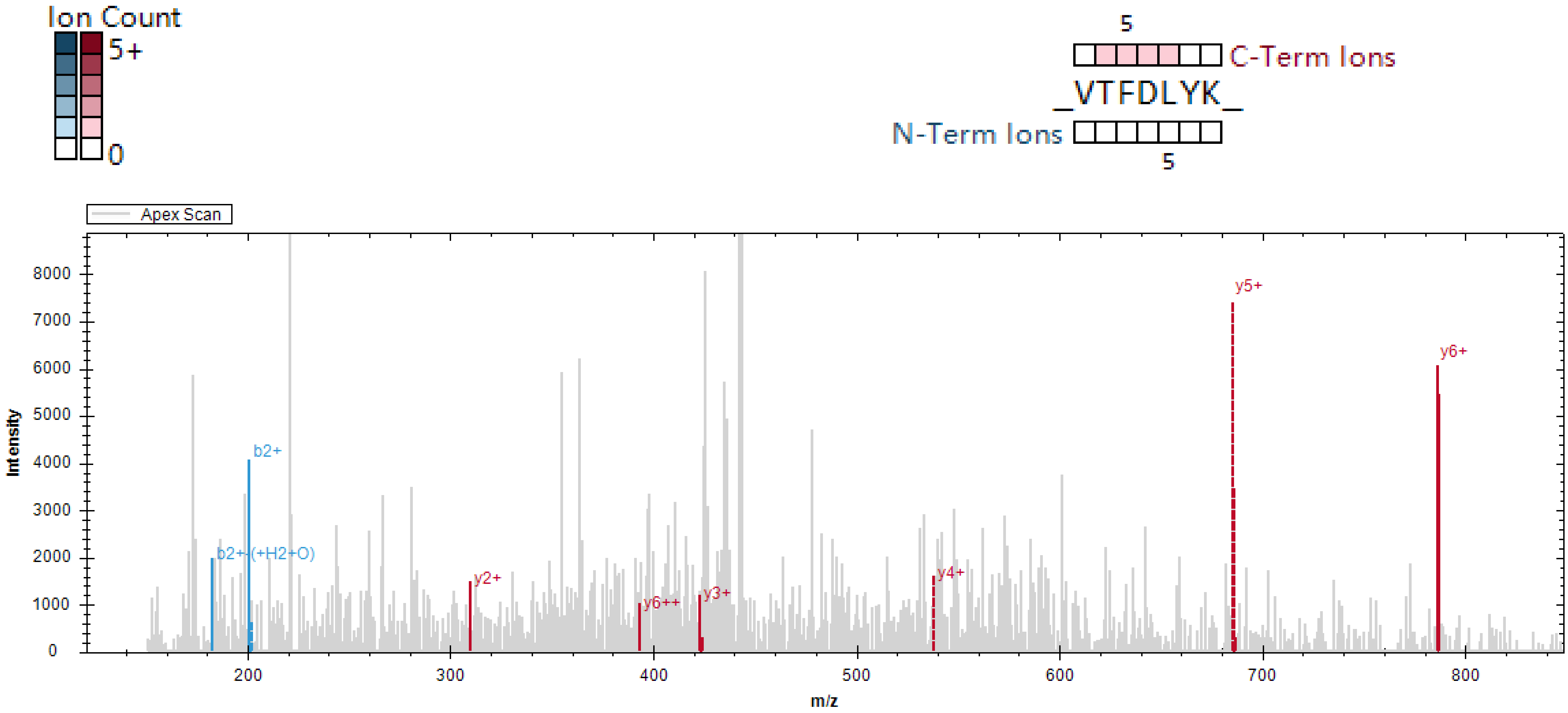

Ion Count
5+
0
5
C-Term Ions
_VTFDLYK_
N-Term Ions
5
Apex Scan
Intensity
m/z
b2+
b2+(+H2+O)
y2+
y6++
y3+
y4+
y5+
y6+

Q96AT9

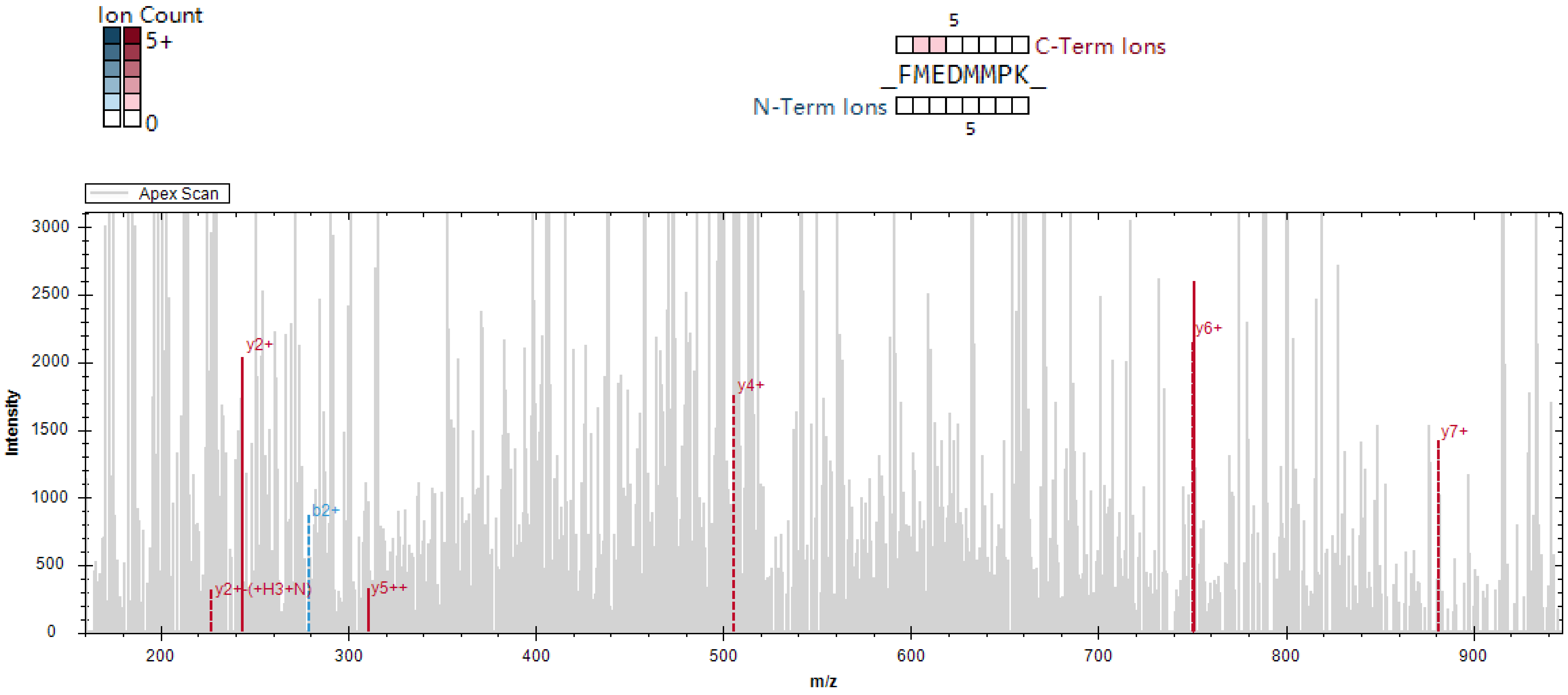
Ion Count
5+
0
5
C-Term Ions
_FMEDMMPK_
N-Term Ions
5
Apex Scan
Intensity
m/z
y2+
y2++(+H3+N)
b2+
y5++
y4+
y6+
y7+
0
500
1000
1500
2000
2500
3000
200
300
400
500
600
700
800
900

Q96D96

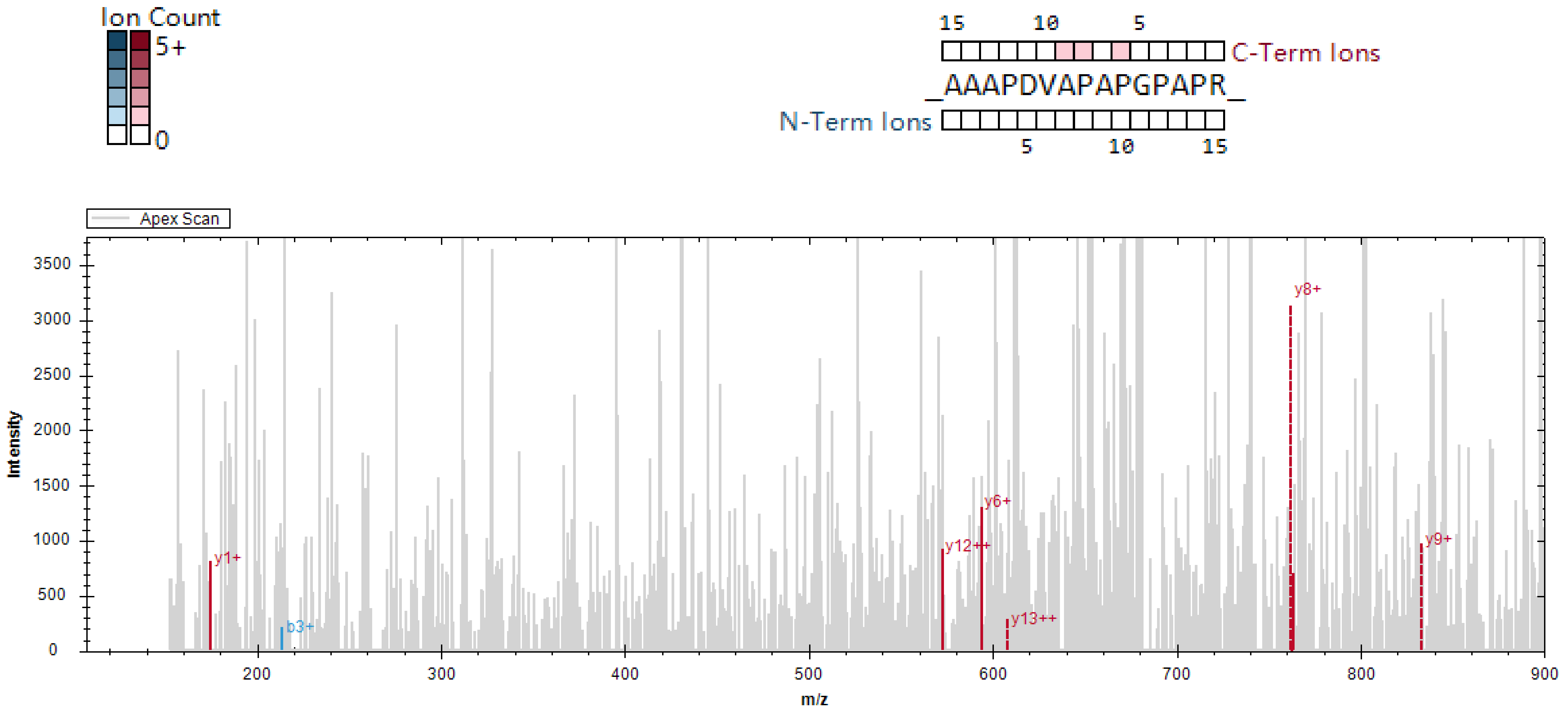
Ion Count
5+
0
15 10 5
C-Term Ions
_AAAPDVAPAPGPAPR_
N-Term Ions
5 10 15
Apex Scan
Intensity
m/z
y1+
b3+
y12++
y6+
y13++
y8+
y9+

Q96EY7

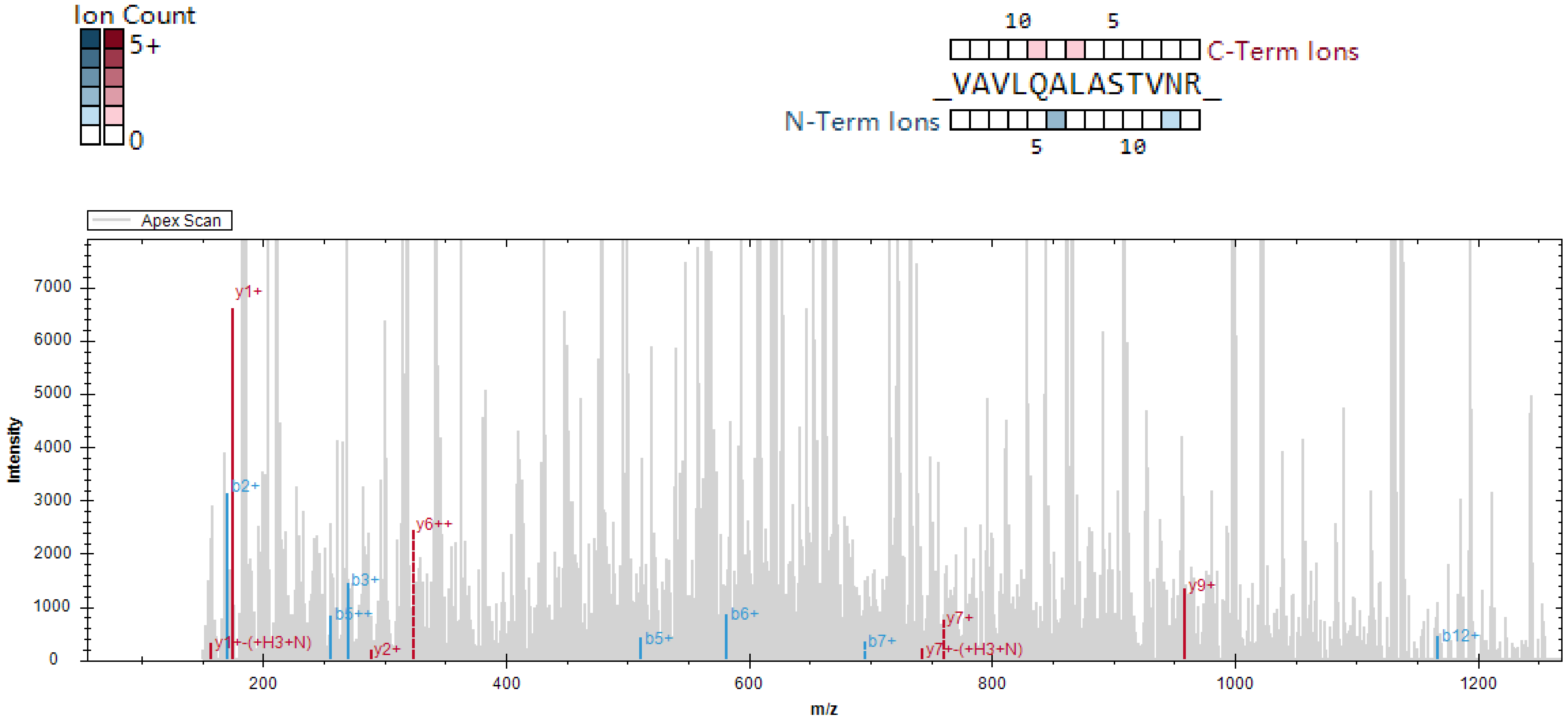

Ion Count
5+
0
10
5
C-Term Ions
_VAVLQALASTVNR_
N-Term Ions
5
10
Apex Scan
Intensity
m/z
y1+
b2+
y6++
b3+
b5++
y1+-(+H3+N)
y2+
b5+
b6+
b7+
y7+
y7+-(+H3+N)
y9+
b12+

Q96FZ5

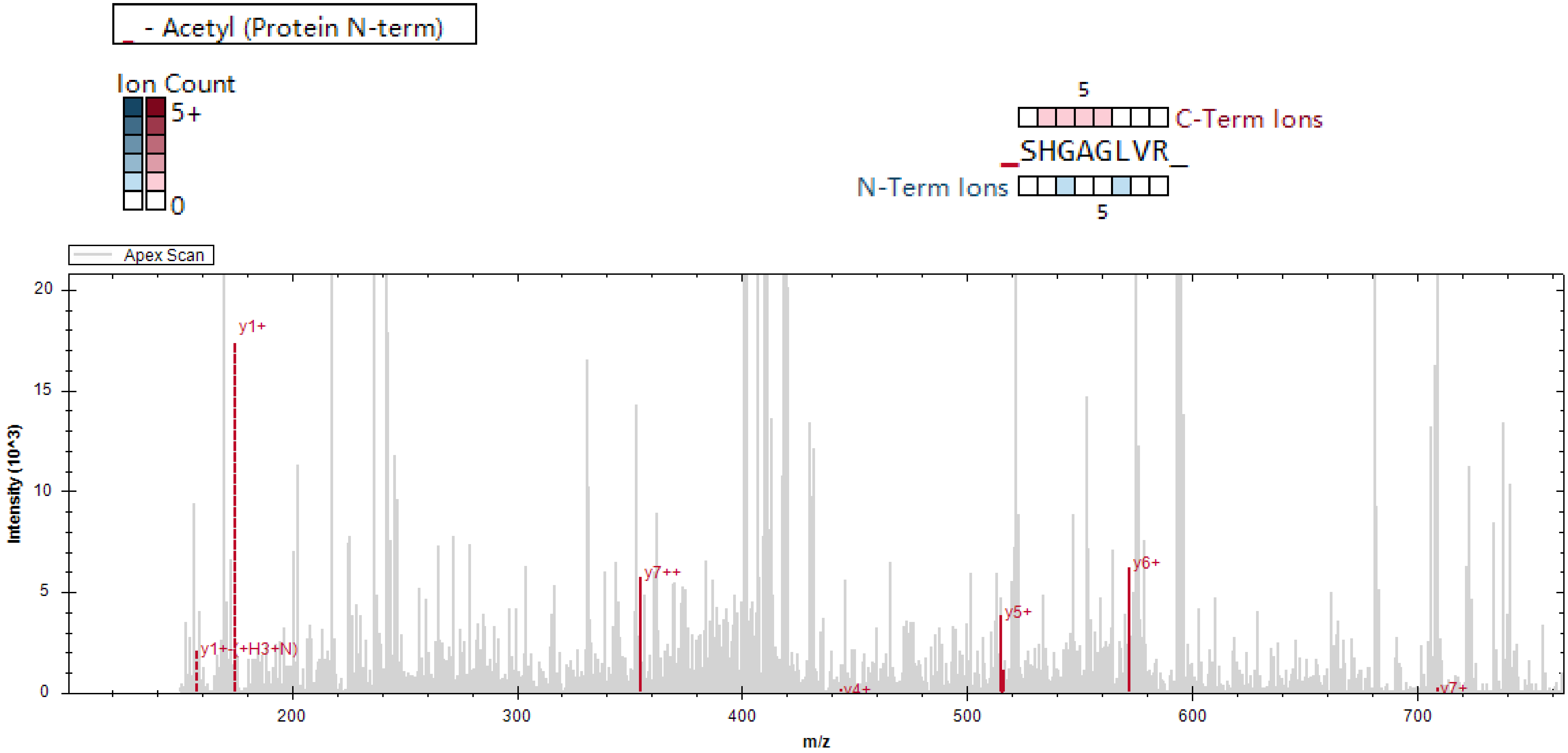

_ - Acetyl (Protein N-term)
Ion Count
5+
0
5
C-Term Ions
_SHGAGLVR_
N-Term Ions
5
Apex Scan
Intensity (10^3)
m/z
y1+
y1+-(+H3+N)
y7++
y5+
y6+
y4+
y7+

Q96GA7

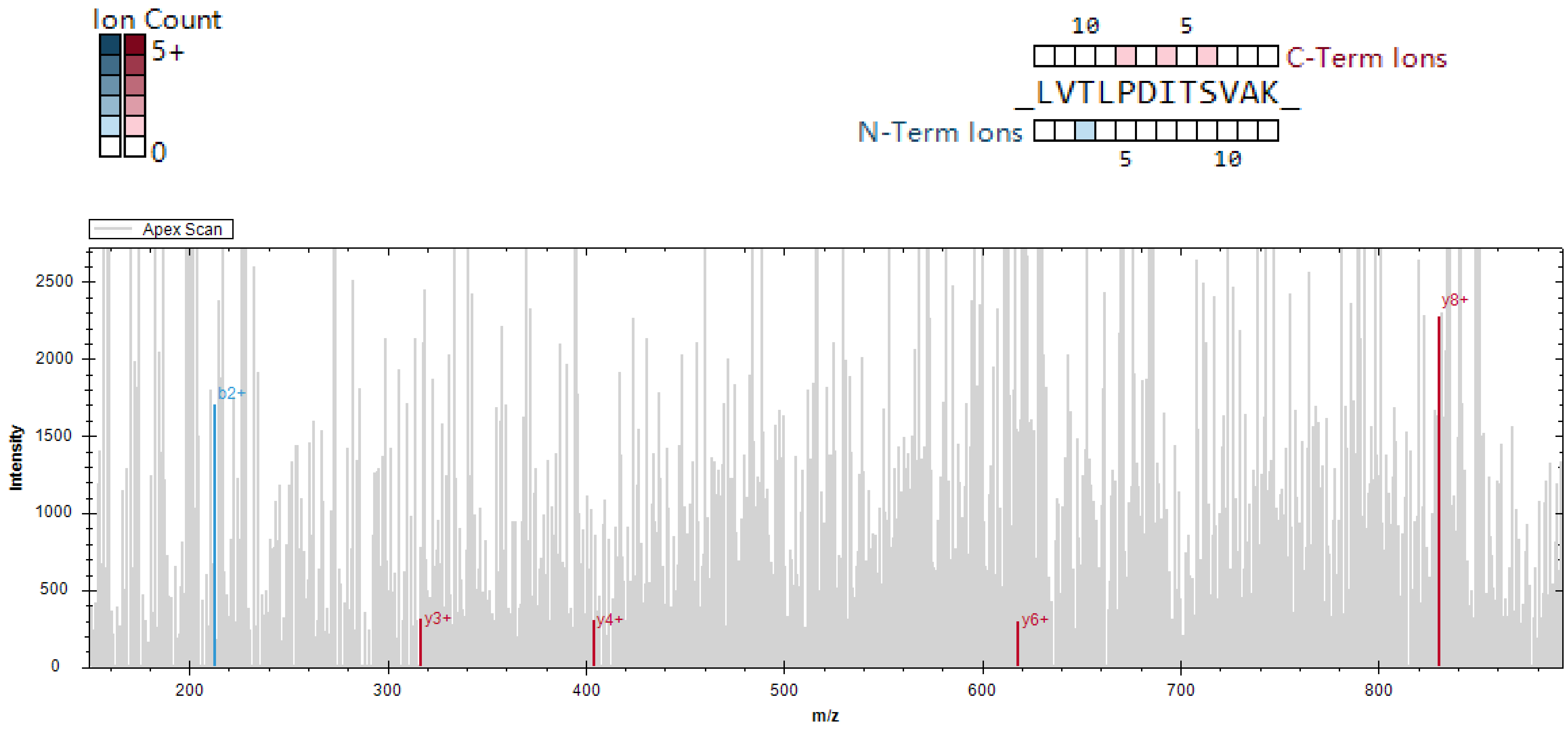

Ion Count
5+
0
10
5
C-Term Ions
_LVTLPDITSVAK_
N-Term Ions
5
10
Apex Scan
b2+
y3+
y4+
y6+
y8+
Intensity
m/z
0
500
1000
1500
2000
2500
200
300
400
500
600
700
800

Q96HU8

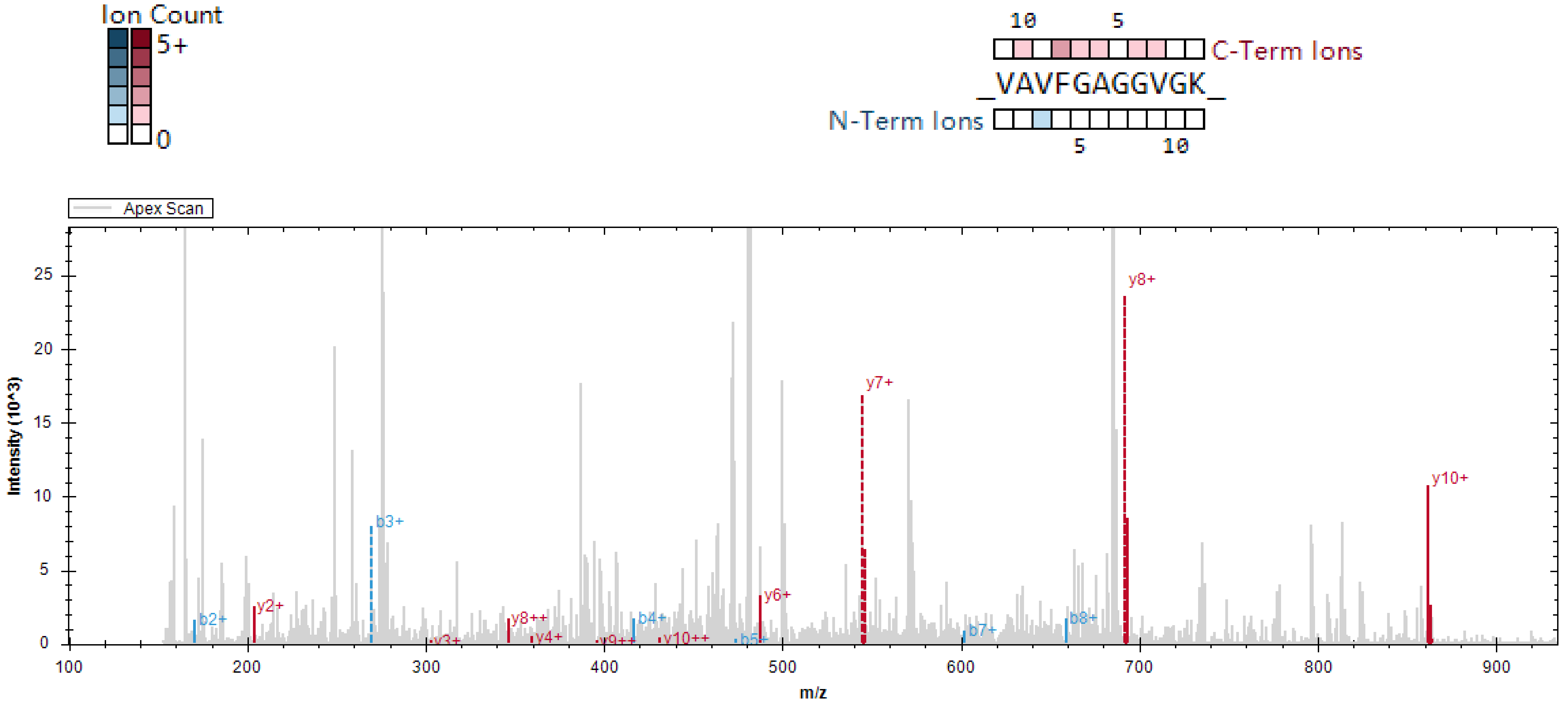
Ion Count
5+
0
10
5
C-Term Ions
_VAVFGAGGVGK_
N-Term Ions
5
10
Apex Scan
y8+
y7+
y10+
b3+
y6+
y2+
b2+
y8++
y4+
y3+
y9++
b4+
y10++
b5+
b7+
b8+
Intensity (10^3)
m/z

Q96IG2

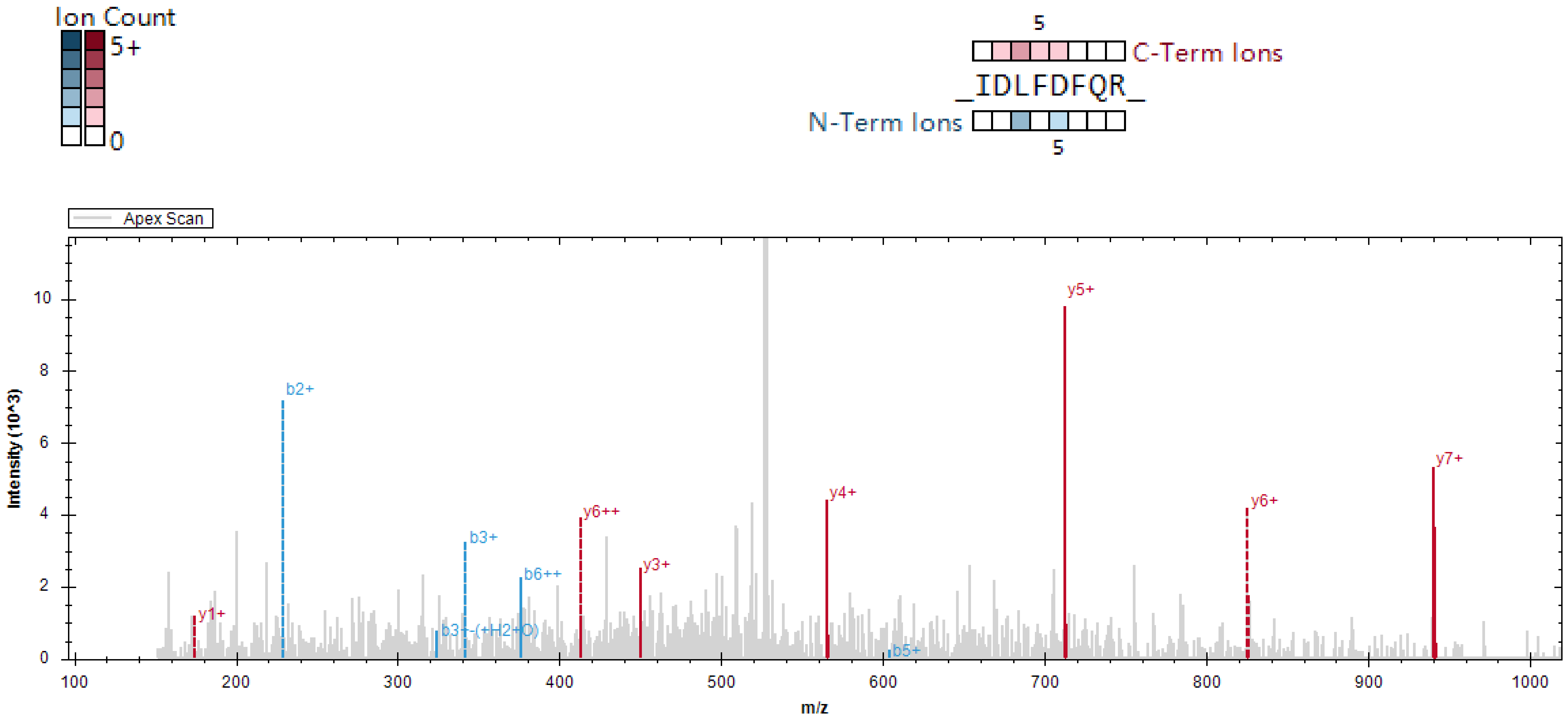

Ion Count
5+
0
5
C-Term Ions
_IDLFDFQR_
N-Term Ions
5
Apex Scan
y5+
b2+
y7+
y4+
y6++
y6+
b3+
y3+
b6++
y1+
b3+-(+H2+O)
b5+
Intensity (10^3)
0
2
4
6
8
10
100
200
300
400
500
600
700
800
900
1000
m/z

Q96JJ6

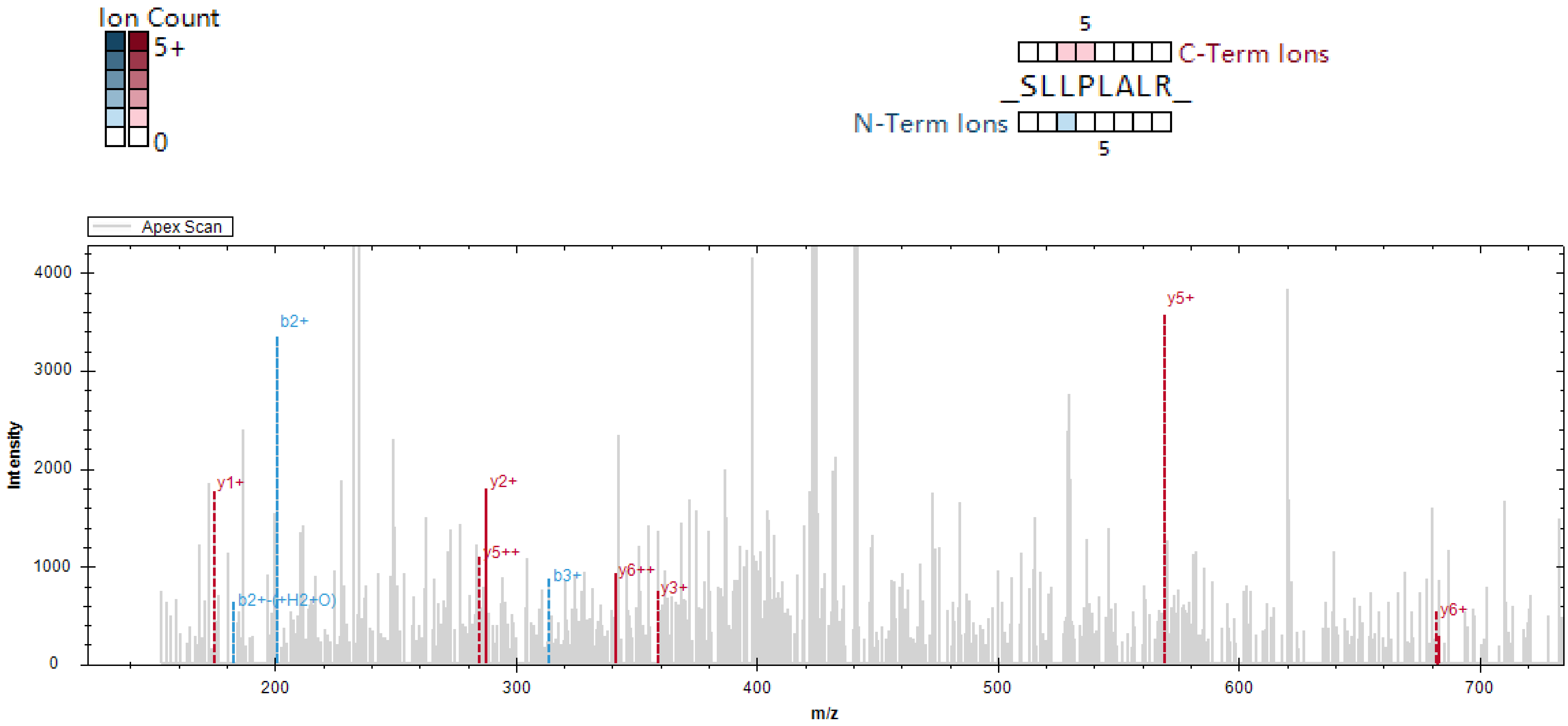

Ion Count
5+
0
5
C-Term Ions
_SLLPLALR_
N-Term Ions
5
Apex Scan
Intensity
m/z
4000
3000
2000
1000
0
200
300
400
500
600
700
b2+
y5+
y1+
y2+
y5++
b3+
y6++
y3+
b2+-(+H2+O)
y6+

Q96L12

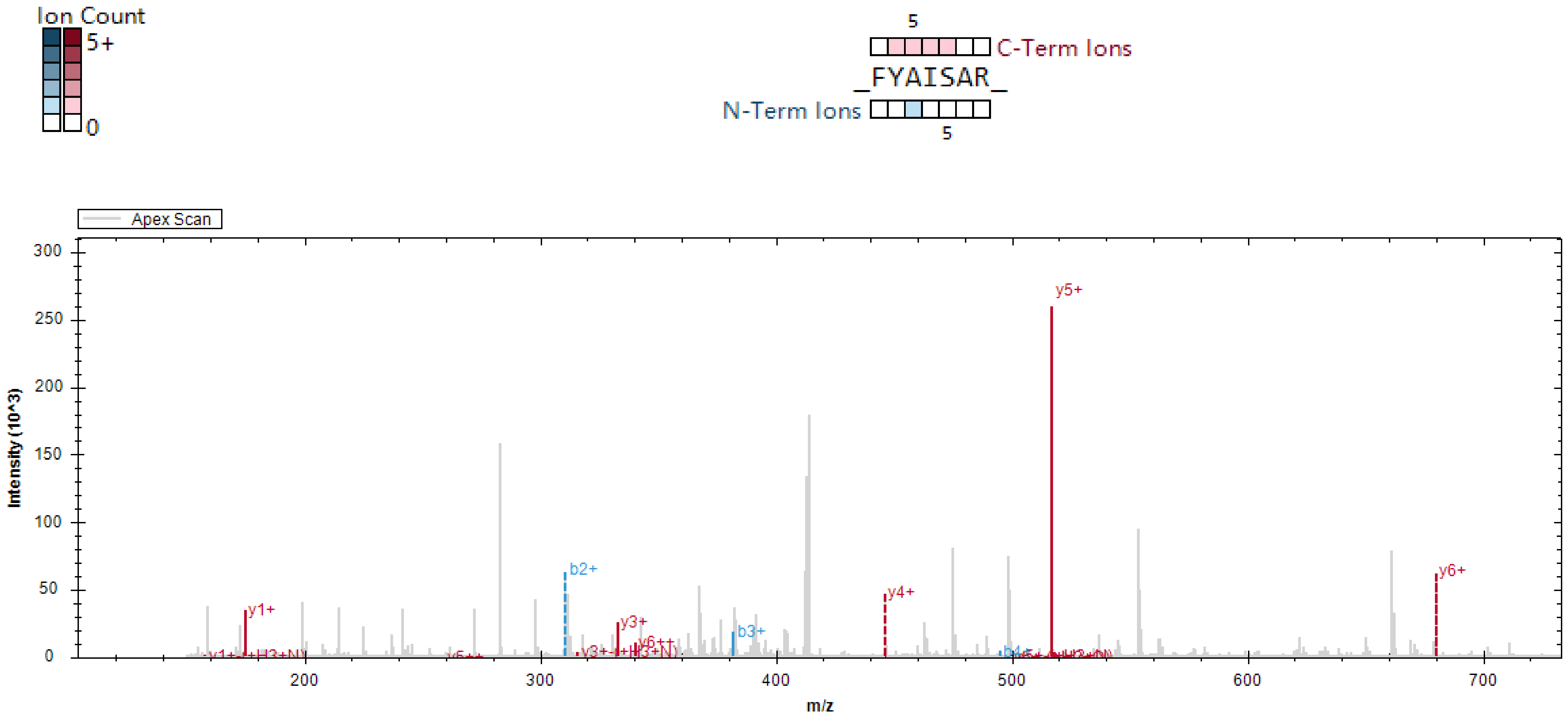

Ion Count
5+
0
5
C-Term Ions
_FYAISAR_
N-Term Ions
5
Apex Scan
Intensity (10^3)
m/z
y5+
y6+
y4+
y1+
b2+
y3+
b3+

Q96MX0

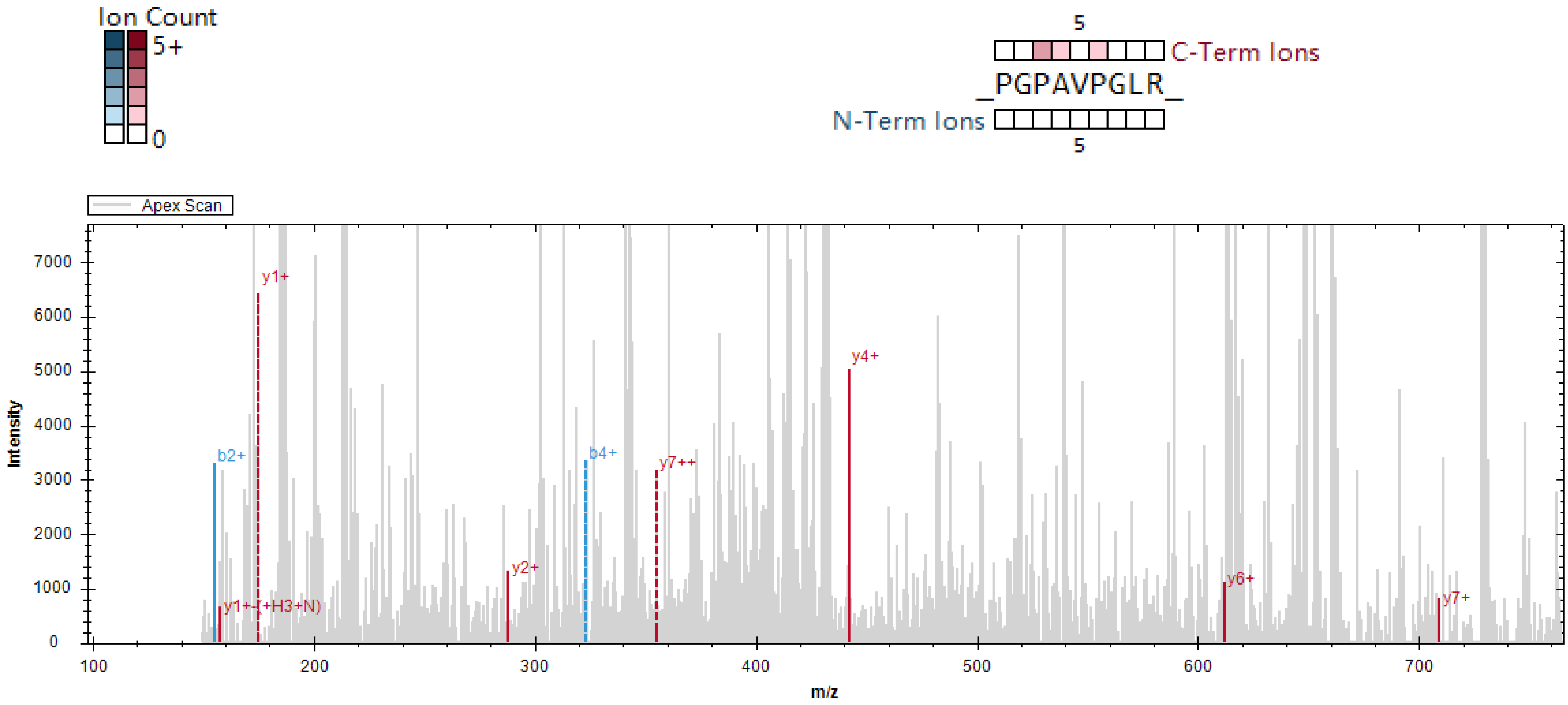

Ion Count
5+
0
5
C-Term Ions
_PGPAVPGLR_
N-Term Ions
5
Apex Scan
Intensity
m/z
y1+
b2+
y1+-(+H3+N)
b4+
y7++
y2+
y4+
y6+
y7+

Q96PF1

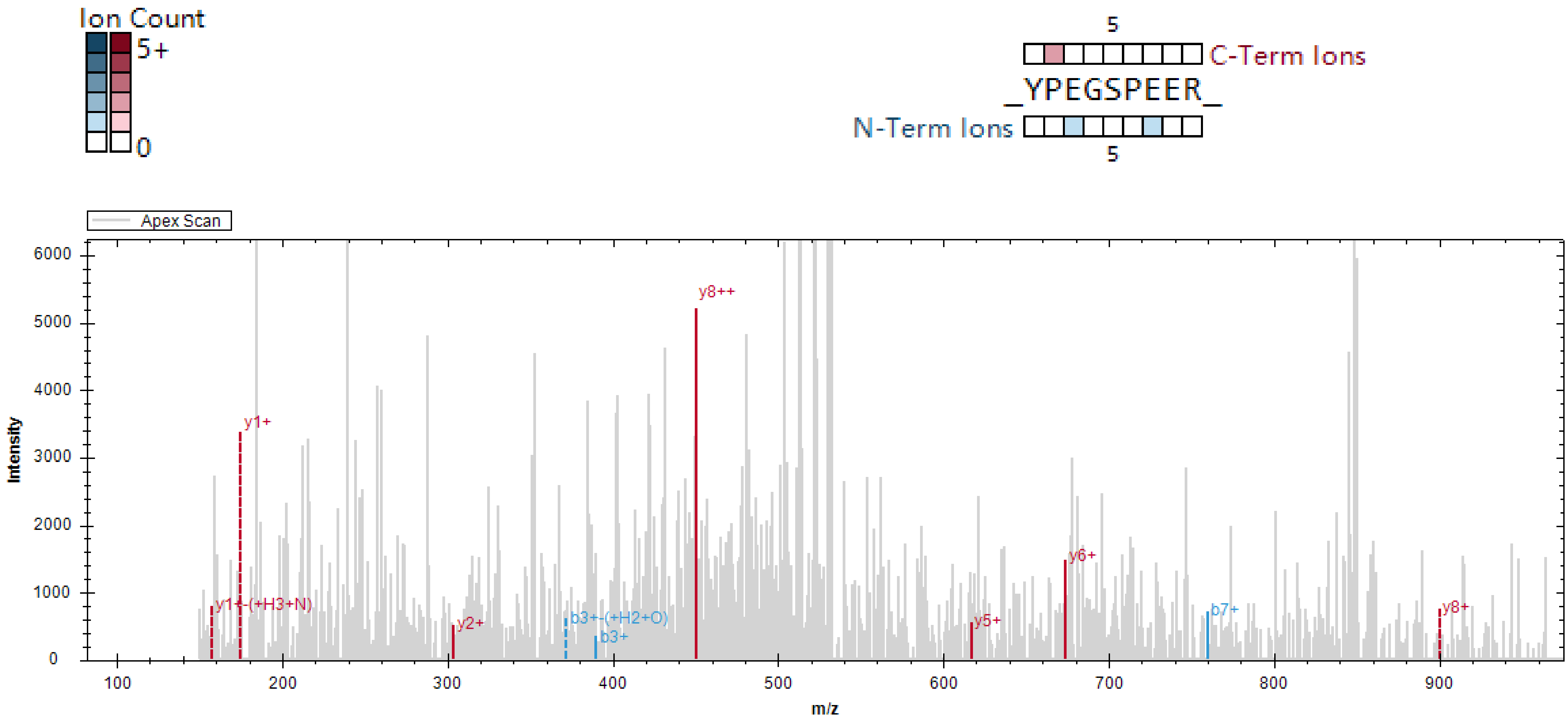

Ion Count
5+
0
5
C-Term Ions
_YPEGSPEER_
N-Term Ions
5
Apex Scan
Intensity
m/z
y8++
y1+
y1+-(+H3+N)
y2+
b3+-(+H2+O)
b3+
y5+
y6+
b7+
y8+

Q96PU5

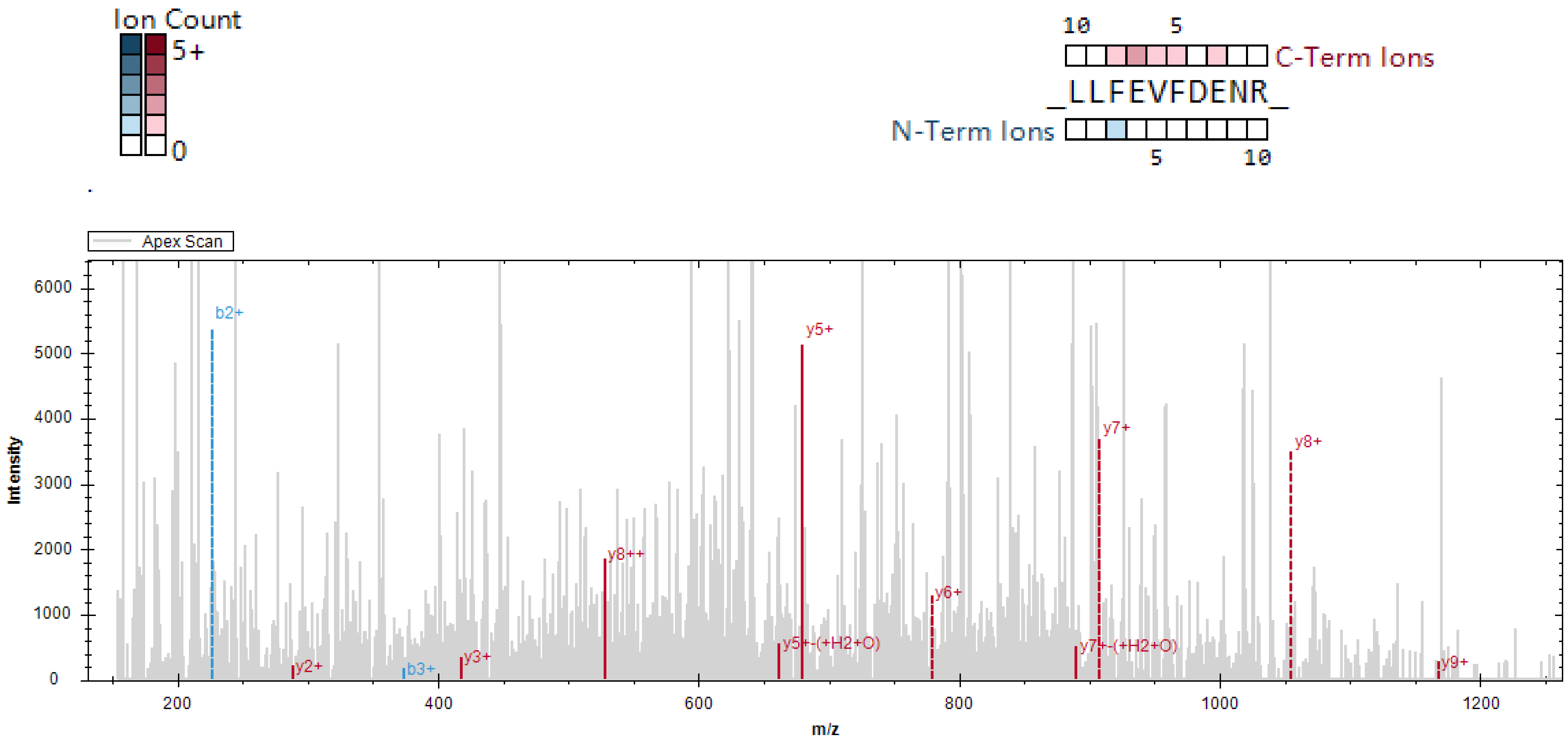
Ion Count
5+
0
10
5
C-Term Ions
_LLFEVFDENR_
N-Term Ions
5
10
Apex Scan
b2+
y5+
y7+
y8+
y8++
y6+
y5+-(+H2+O)
y7+-(+H2+O)
y2+
b3+
y3+
y9+
Intensity
m/z
0
1000
2000
3000
4000
5000
6000
200
400
600
800
1000
1200

Q96QF0

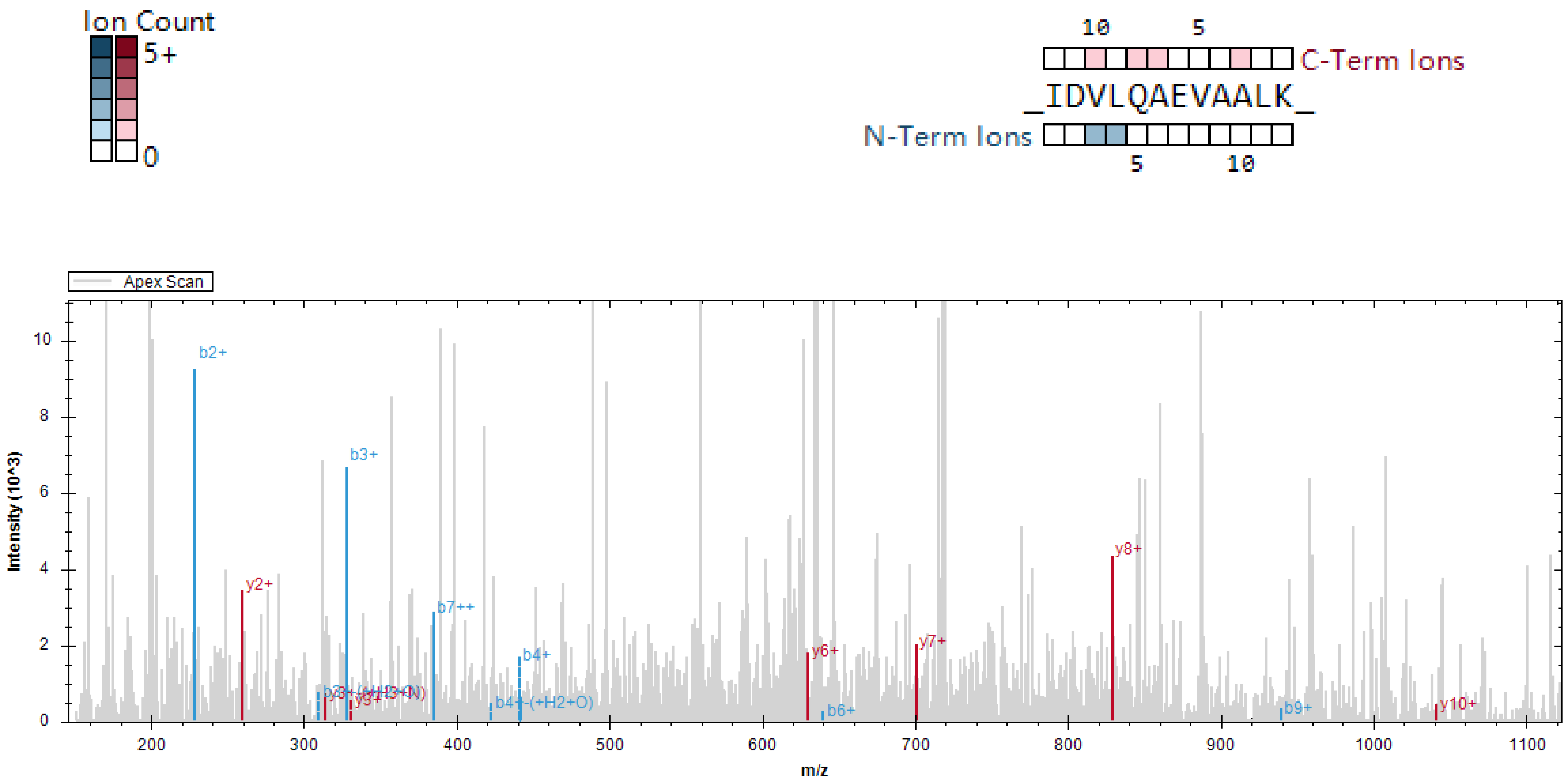
Ion Count
5+
0
10
5
C-Term Ions
_IDVLQAEVAALK_
N-Term Ions
5
10
Apex Scan
Intensity (10^3)
m/z
b2+
b3+
y2+
b7++
b4+
b4+-(+H2+O)
y6+
b6+
y7+
y8+
b9+
y10+

Q96QR1

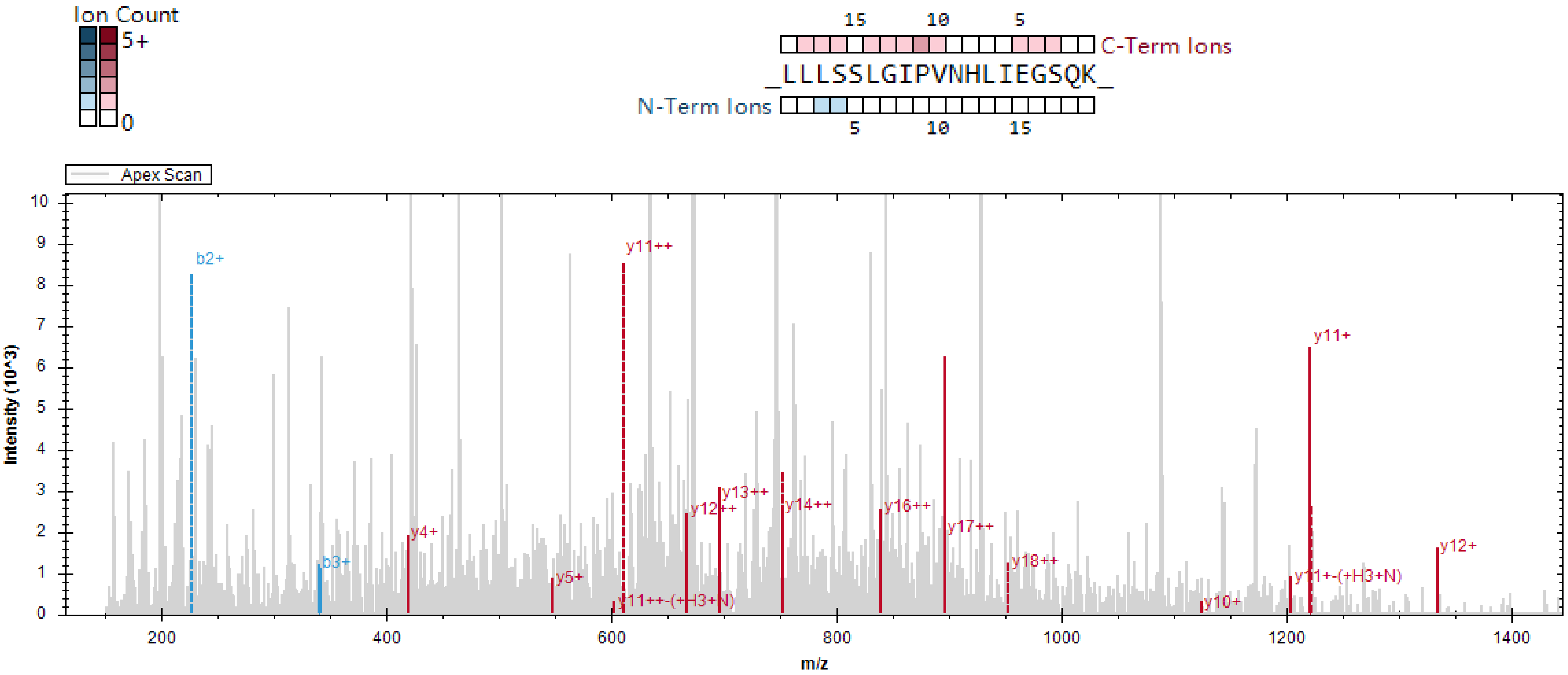

Ion Count
5+
0
15 10 5
C-Term Ions
_LLLSSLGIPVNHLIEGSQK_
N-Term Ions
5 10 15
Apex Scan
Intensity (10^3)
m/z
b2+
b3+
y4+
y5+
y11++
y11++-(+H3+N)
y12++
y13++
y14++
y16++
y17++
y18++
y10+
y11+
y11+-(+H3+N)
y12+

Q96RD6

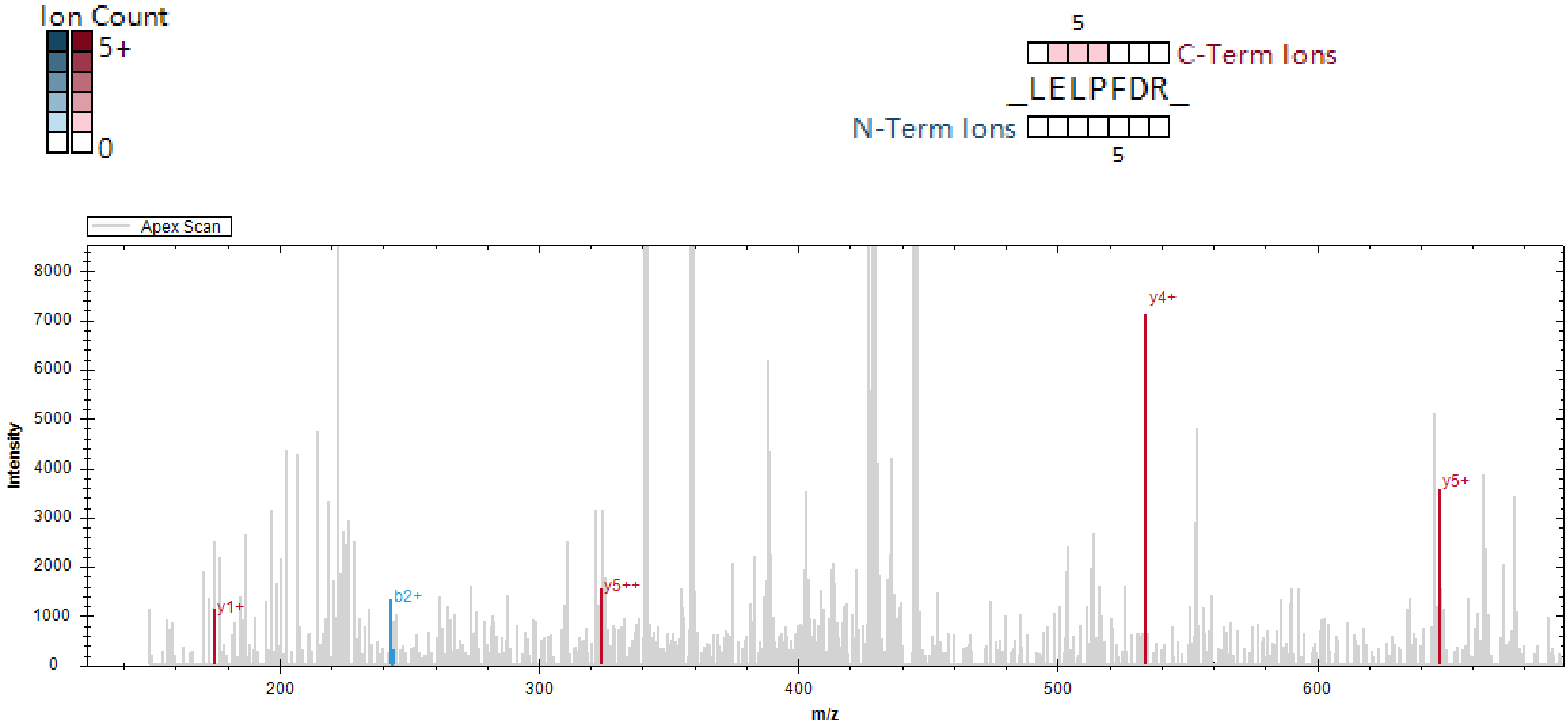

Ion Count
5+
0
5
C-Term Ions
_LELPFDR_
N-Term Ions
5
Apex Scan
y4+
y5+
y5++
y1+
b2+
Intensity
m/z

Q96SZ5

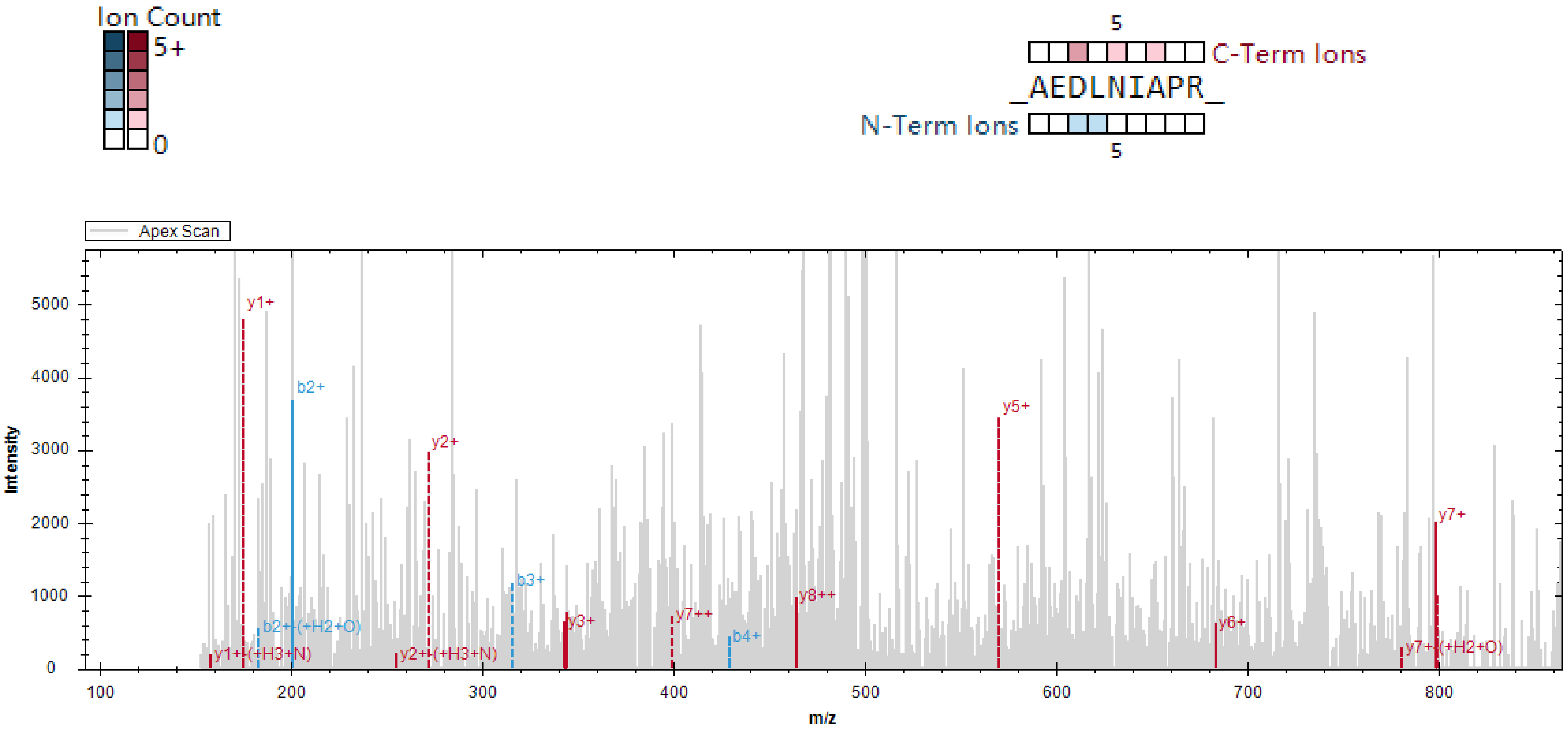

Ion Count
5+
0
5
C-Term Ions
_AEDLNIAPR_
N-Term Ions
5
Apex Scan
Intensity
m/z
y1+
b2+
y2+
y5+
y7+
b3+
y3+
y7++
y8++
b4+
y6+
b2+-(+H2+O)
y1+-(+H3+N)
y2+-(+H3+N)
y7+-(+H2+O)
0
1000
2000
3000
4000
5000
100
200
300
400
500
600
700
800

Q99572

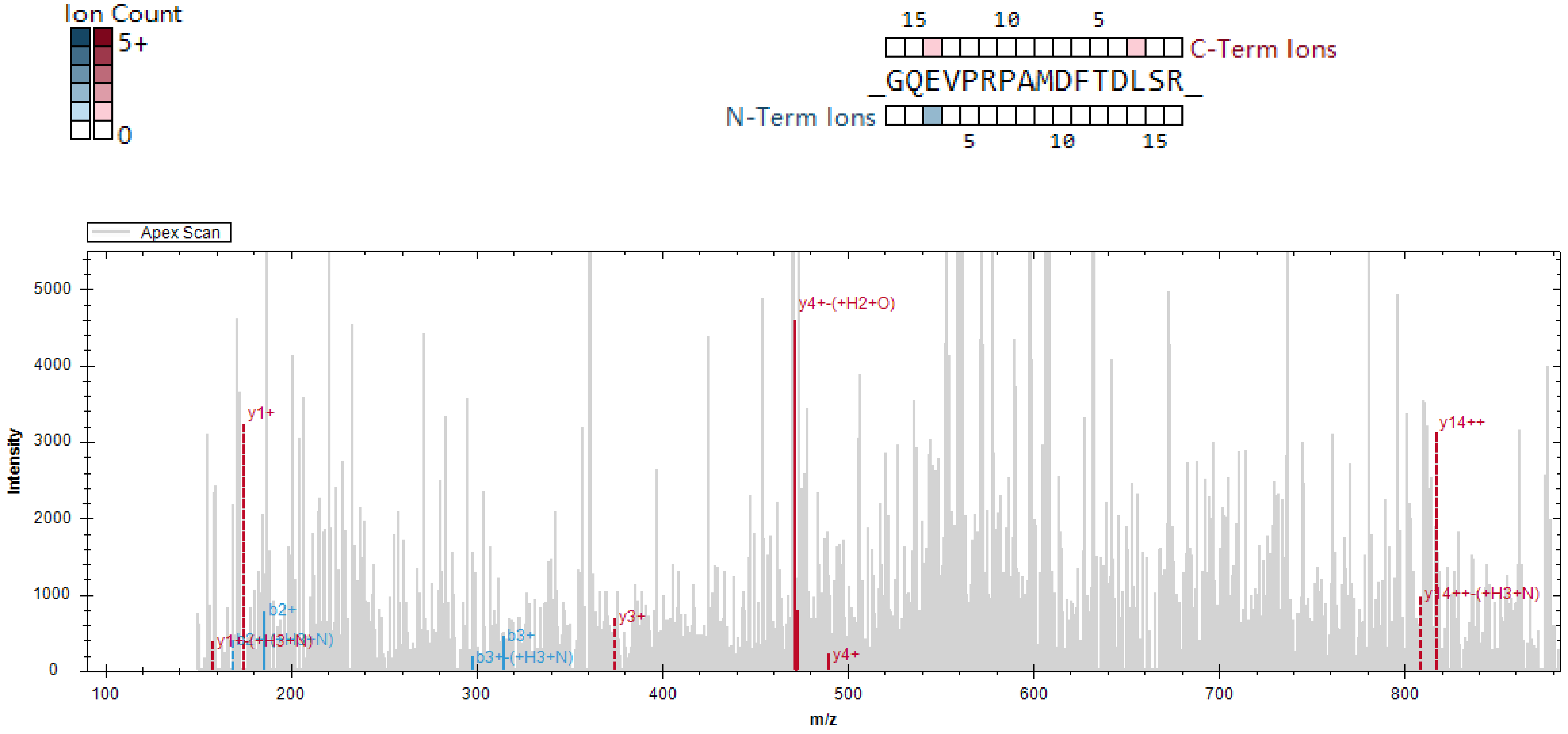
Ion Count
5+
0
15
10
5
C-Term Ions
_GQEVPRPAMDFTDLSR_
N-Term Ions
5
10
15
Apex Scan
Intensity
m/z
y1+
b2+
b3+
b3+-(+H3+N)
y3+
y4+-(+H2+O)
y4+
y14++
y14++-(+H3+N)

Q99613

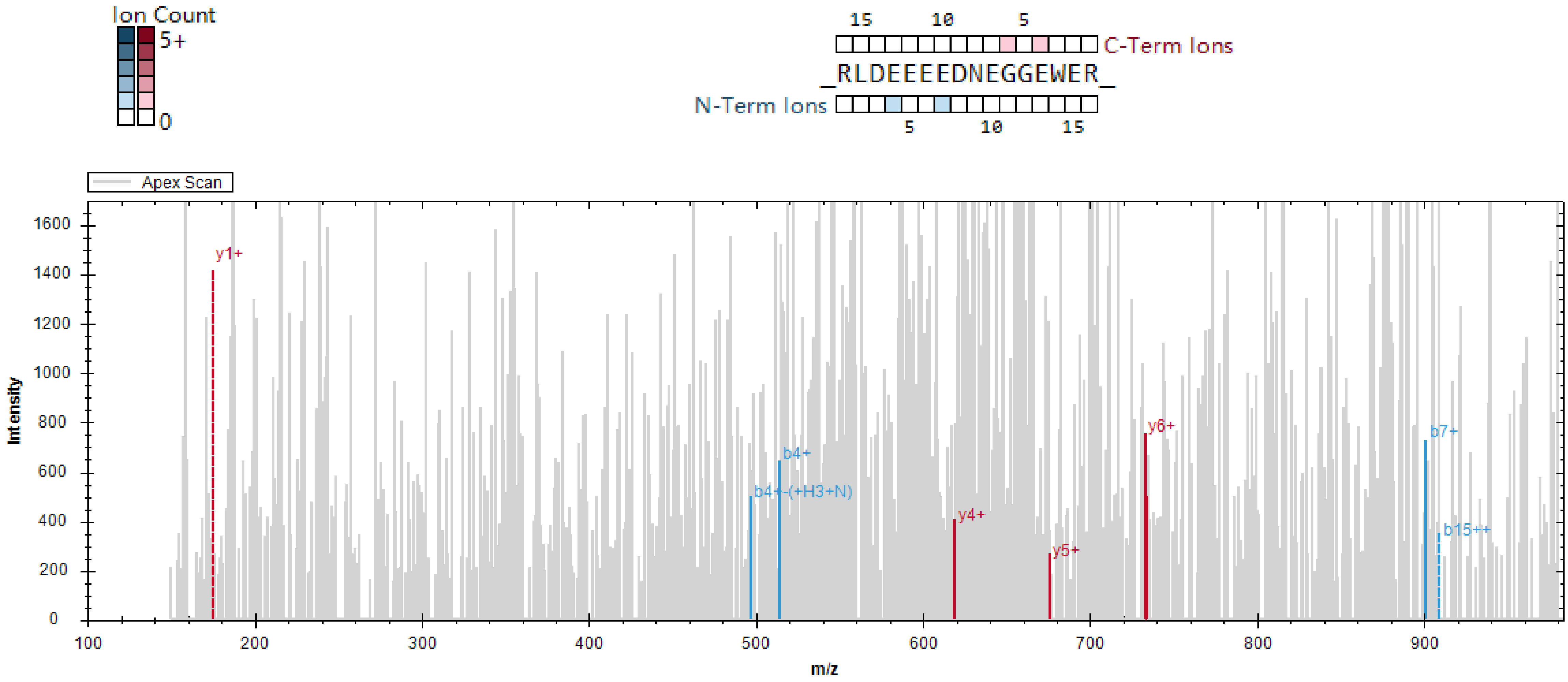

Ion Count
5+
0
15
10
5
C-Term Ions
_RLDEEEEDNEGGEWER_
N-Term Ions
5
10
15
Apex Scan
Intensity
m/z
y1+
b4+
b4+-(+H3+N)
y4+
y5+
y6+
b7+
b15++

Q99933

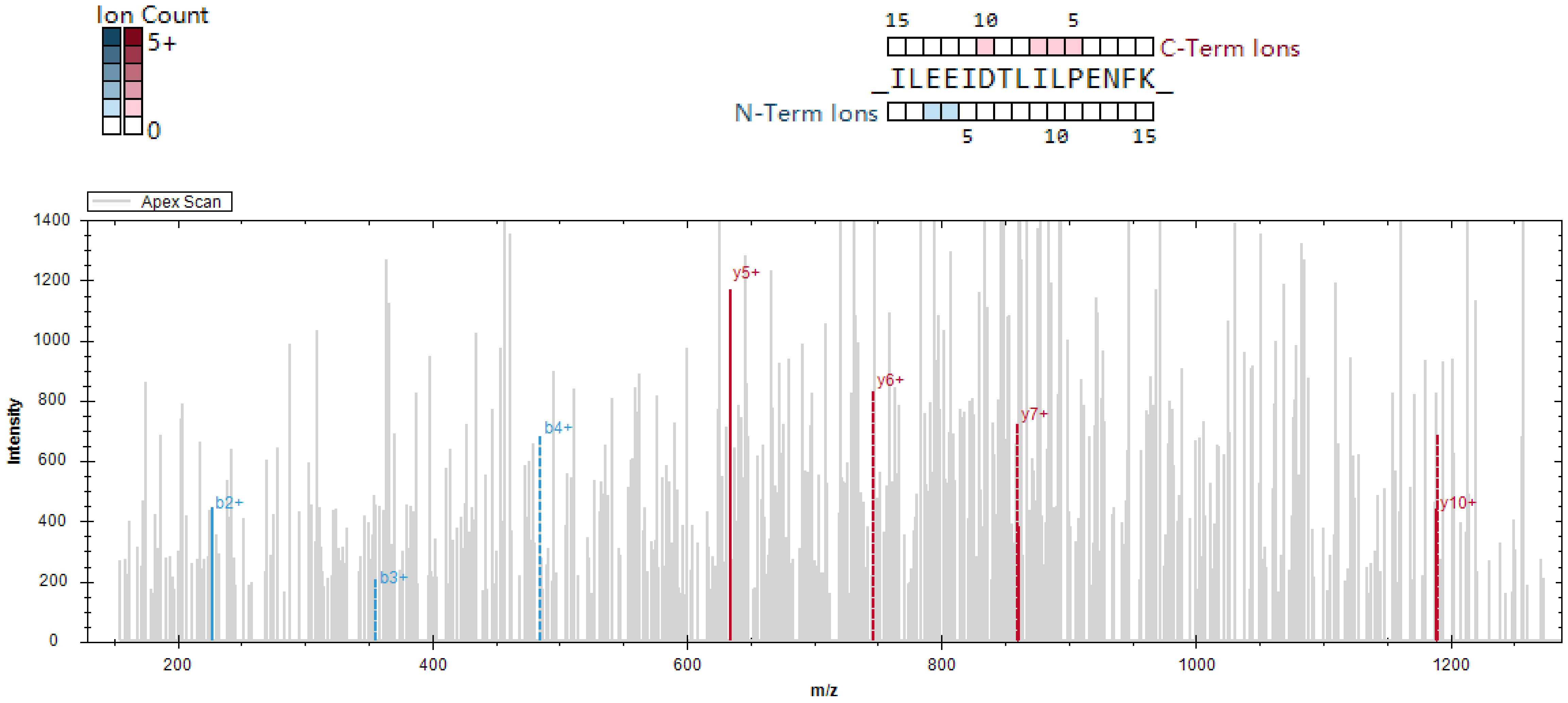

Ion Count
5+
0
15
10
5
C-Term Ions
_ILEEIDTLILPENFK_
N-Term Ions
5
10
15
Apex Scan
Intensity
m/z
y5+
y6+
y7+
y10+
b2+
b3+
b4+

# Q9BRR9

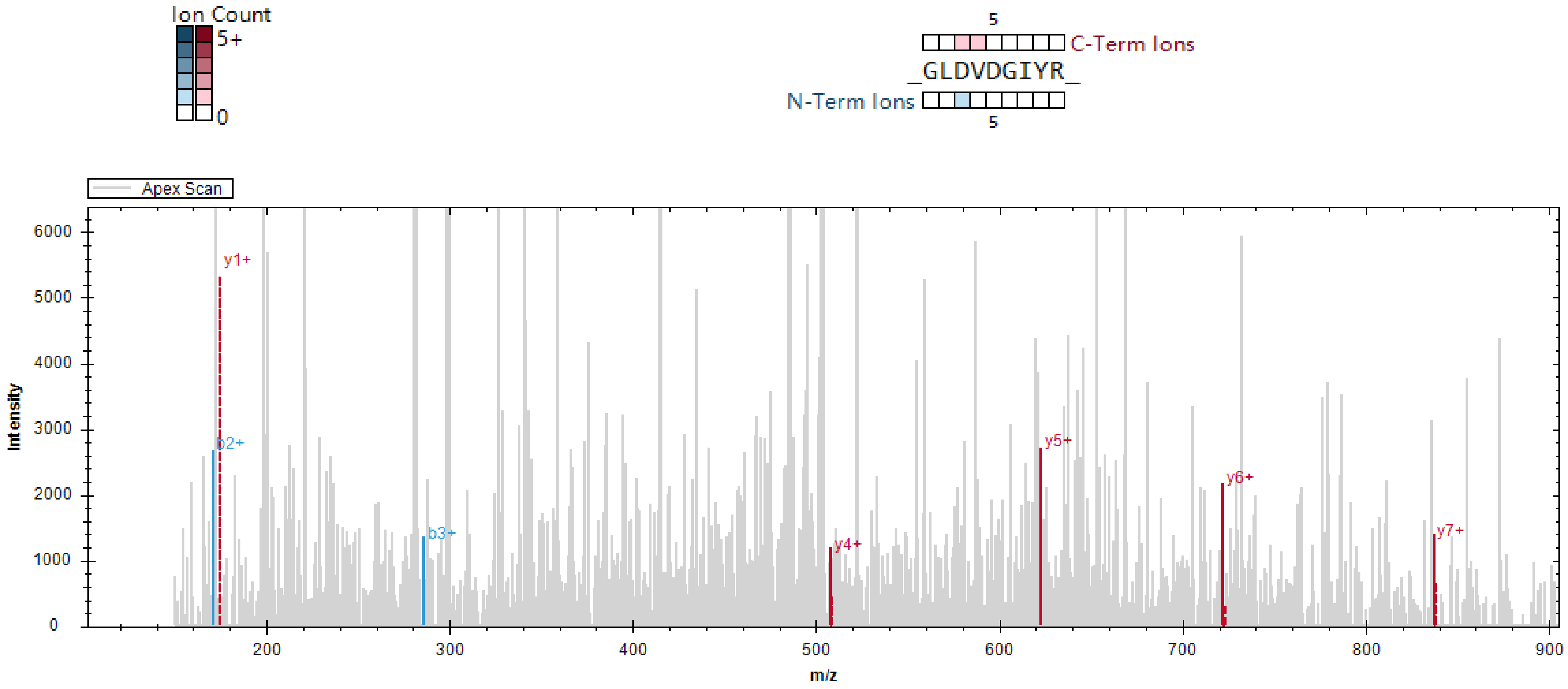

Ion Count
5+
0
5
C-Term Ions
_GLDVDGIYR_
N-Term Ions
5
Apex Scan
Intensity
0
1000
2000
3000
4000
5000
6000
y1+
b2+
b3+
y4+
y5+
y6+
y7+
200
300
400
500
600
700
800
900
m/z

# Q9BRY0

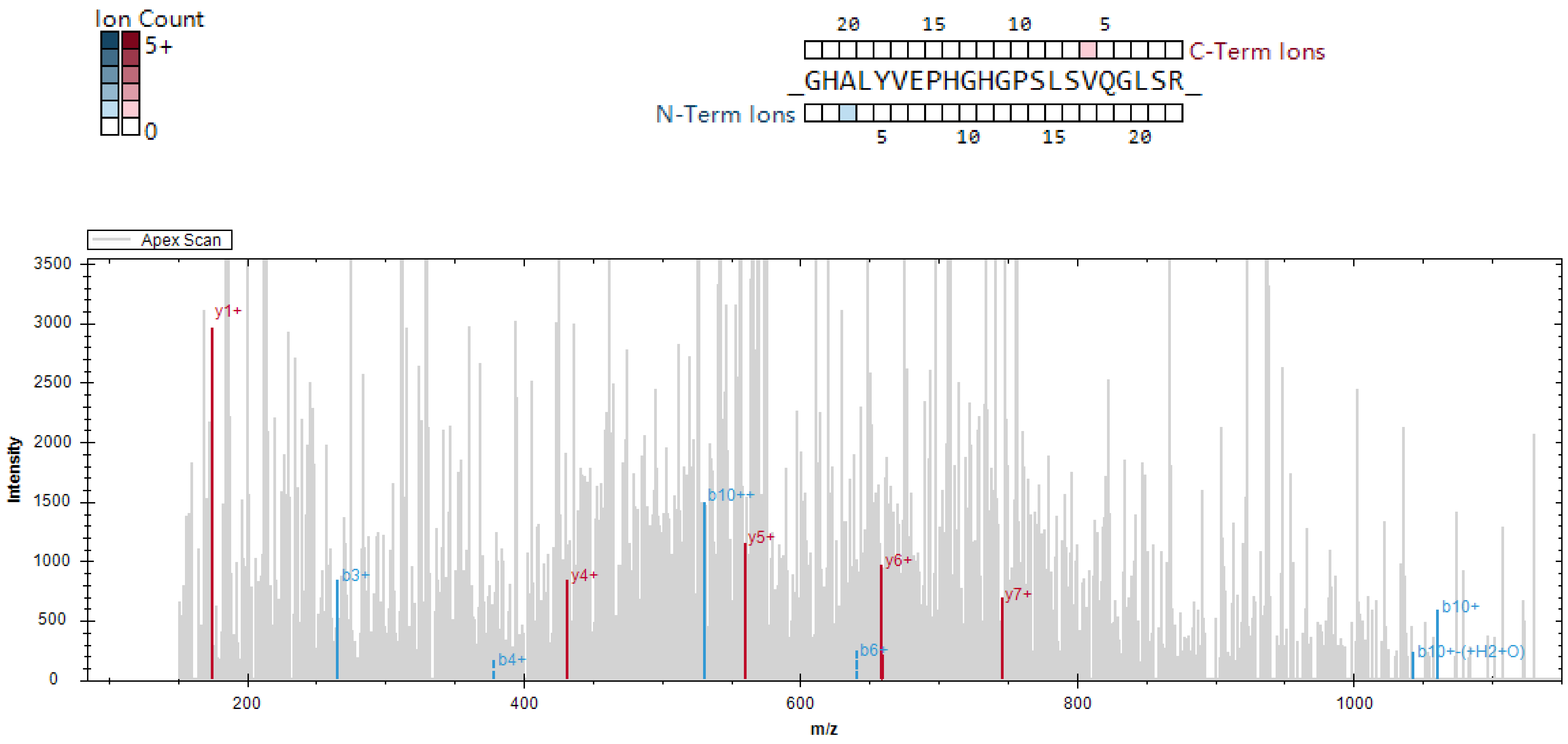

Ion Count
5+
0
20
15
10
5
C-Term Ions
_GHALYVEPHGHGPSLSVQGLSR_
N-Term Ions
5
10
15
20
Apex Scan
Intensity
m/z
y1+
b3+
b4+
y4+
b10++
y5+
b6+
y6+
y7+
b10+
b10+-(+H2+O)

Q9BT23

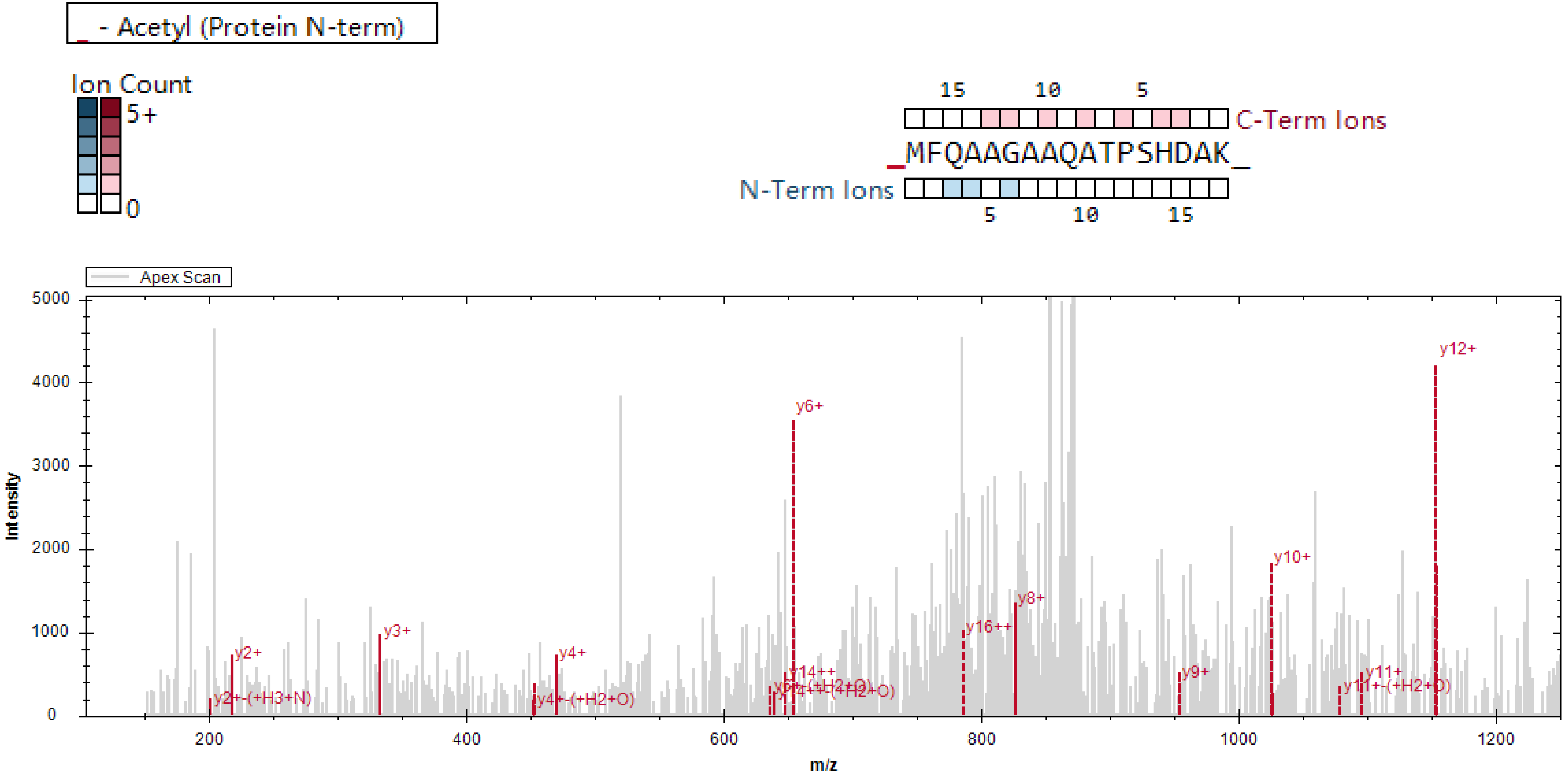

_ - Acetyl (Protein N-term)
Ion Count
5+
0
15
10
5
C-Term Ions
_MFQAAGAAQATPSHDAK_
N-Term Ions
5
10
15
Apex Scan
Intensity
m/z
y12+
y6+
y10+
y8+
y16++
y3+
y2+
y4+
y14++
y9+
y11+
y2+-(+H3+N)
y4+-(+H2+O)
y11+-(+H2+O)

Q9BZW8

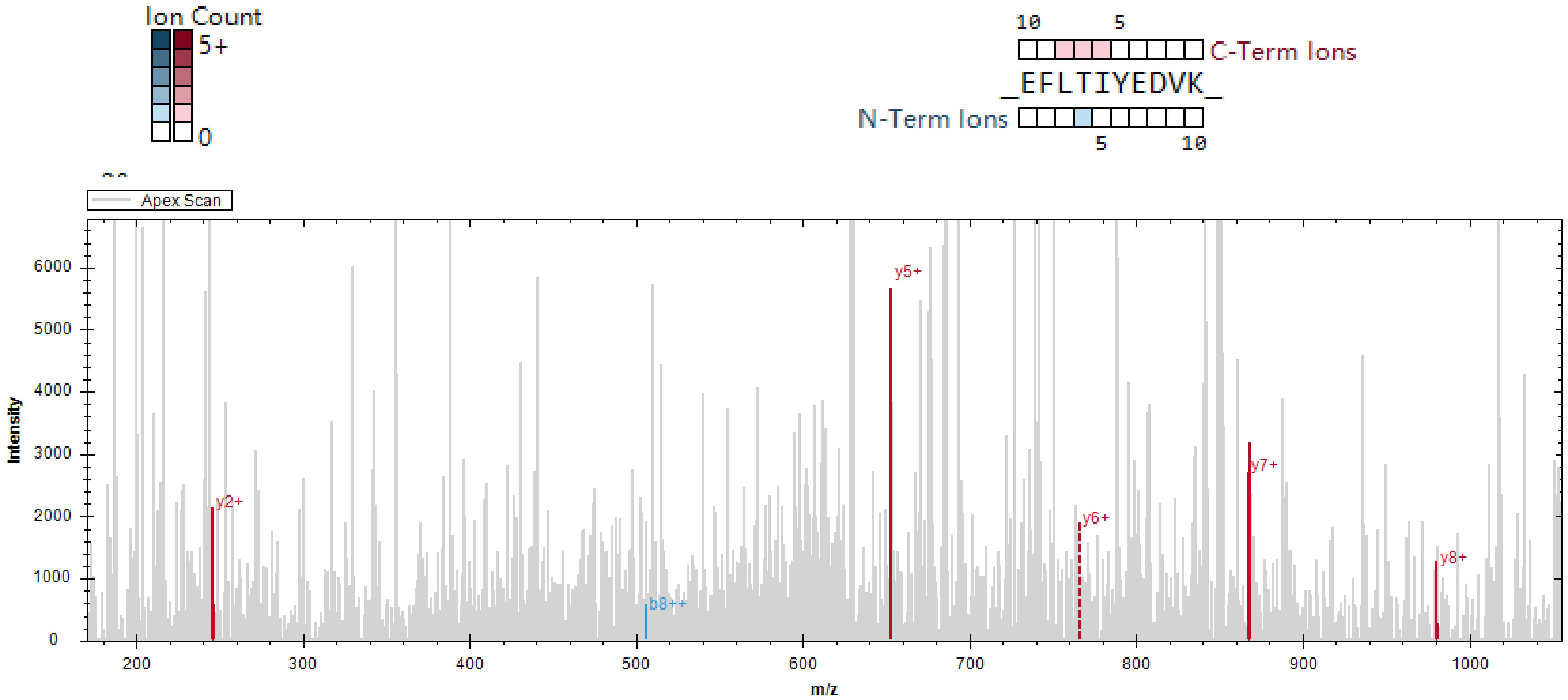

Ion Count
5+
0
10
5
C-Term Ions
_EFLTIYEDVK_
N-Term Ions
5
10
Apex Scan
y5+
y2+
y6+
y7+
y8+
b8++
Intensity
m/z
0
1000
2000
3000
4000
5000
6000
200
300
400
500
600
700
800
900
1000

Q9C030

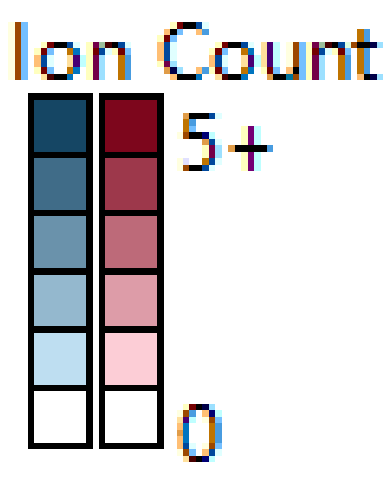

Ion Count
5+
0


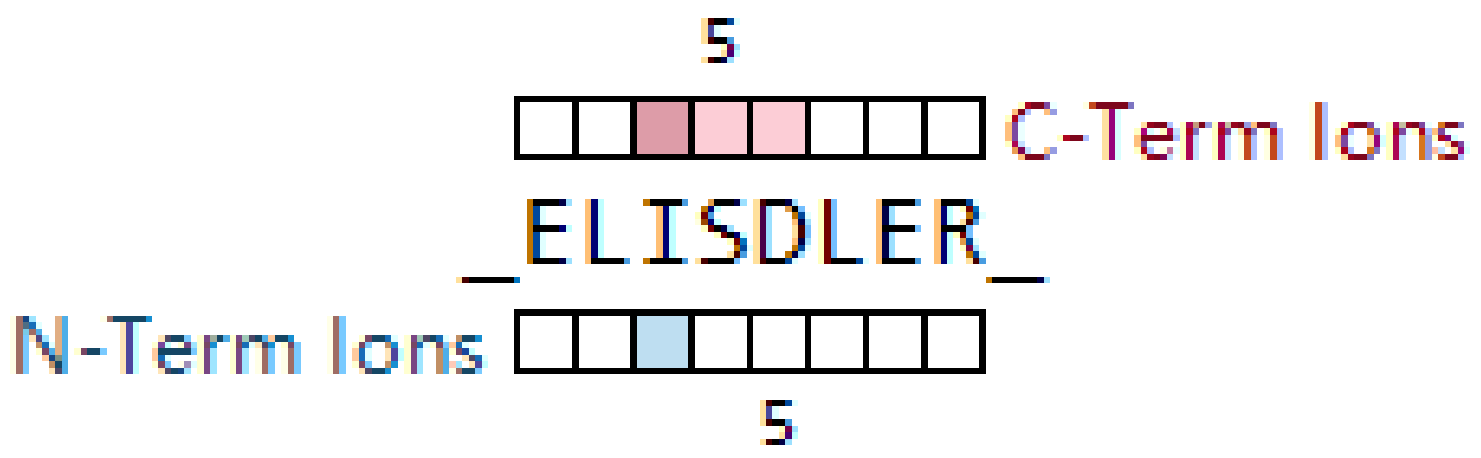

5
C-Term Ions
_ELISDLER_
N-Term Ions
5


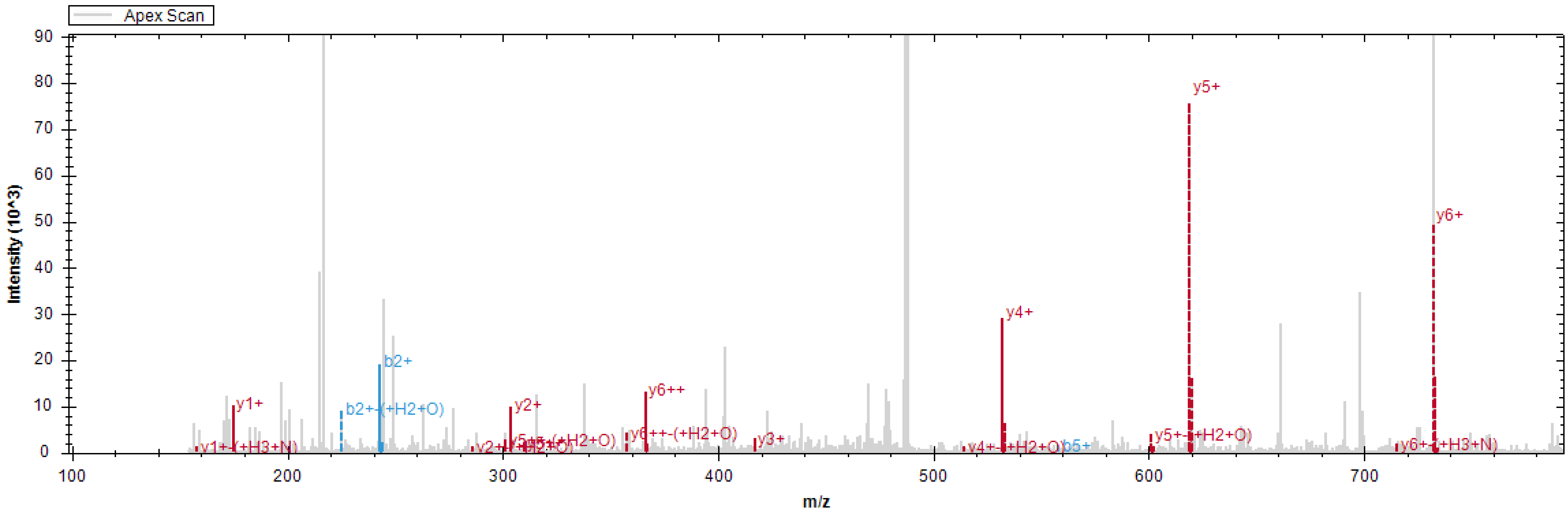

Apex Scan
Intensity (10^3)
m/z
y5+
y6+
y4+
b2+
y6++
y1+
y2+
b2+-(+H2+O)
y6++-(+H2+O)
y3+
y5+-(+H2+O)
y6+-(+H3+N)
y4+-(+H2+O)
b5+

Q9GZN4

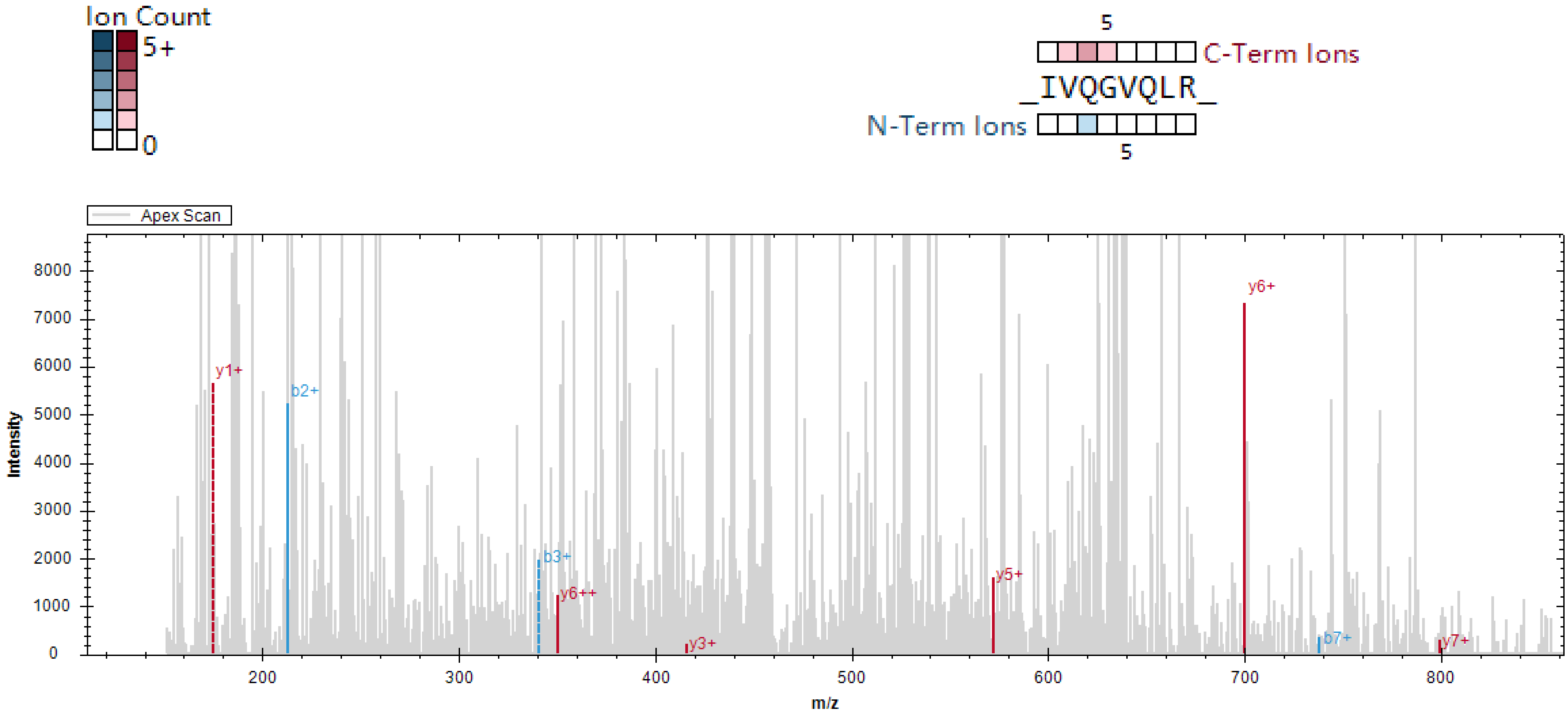

Ion Count
5+
0
5
C-Term Ions
_IVQGVQLR_
N-Term Ions
5
Apex Scan
Intensity
m/z
y1+
b2+
b3+
y6++
y3+
y5+
y6+
b7+
y7+

# Q9H3H5

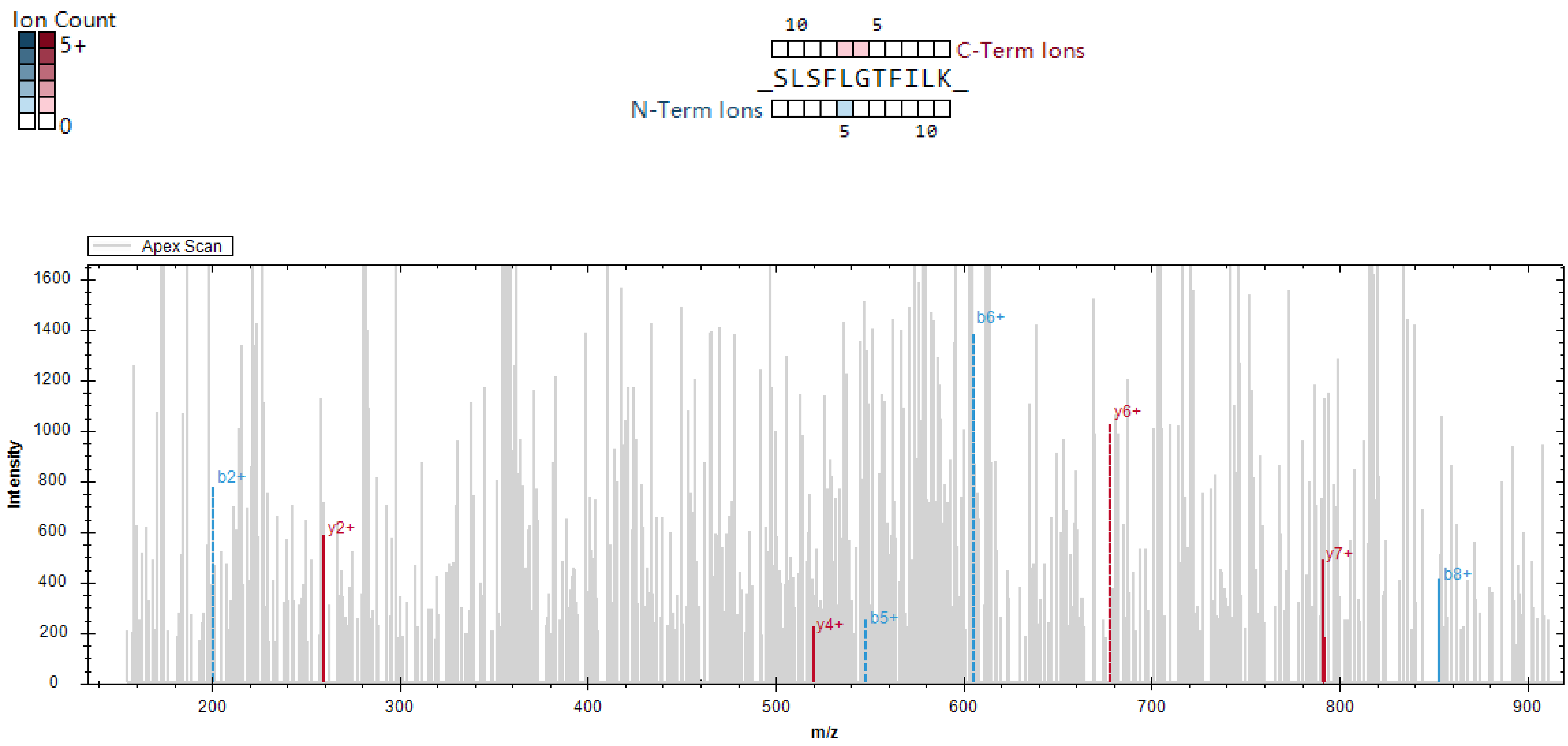
Ion Count
5+
0
10
5
C-Term Ions
_SLSFLGTFILK_
N-Term Ions
5
10
Apex Scan
Intensity
m/z
b2+
y2+
y4+
b5+
b6+
y6+
y7+
b8+

Q9H3S1

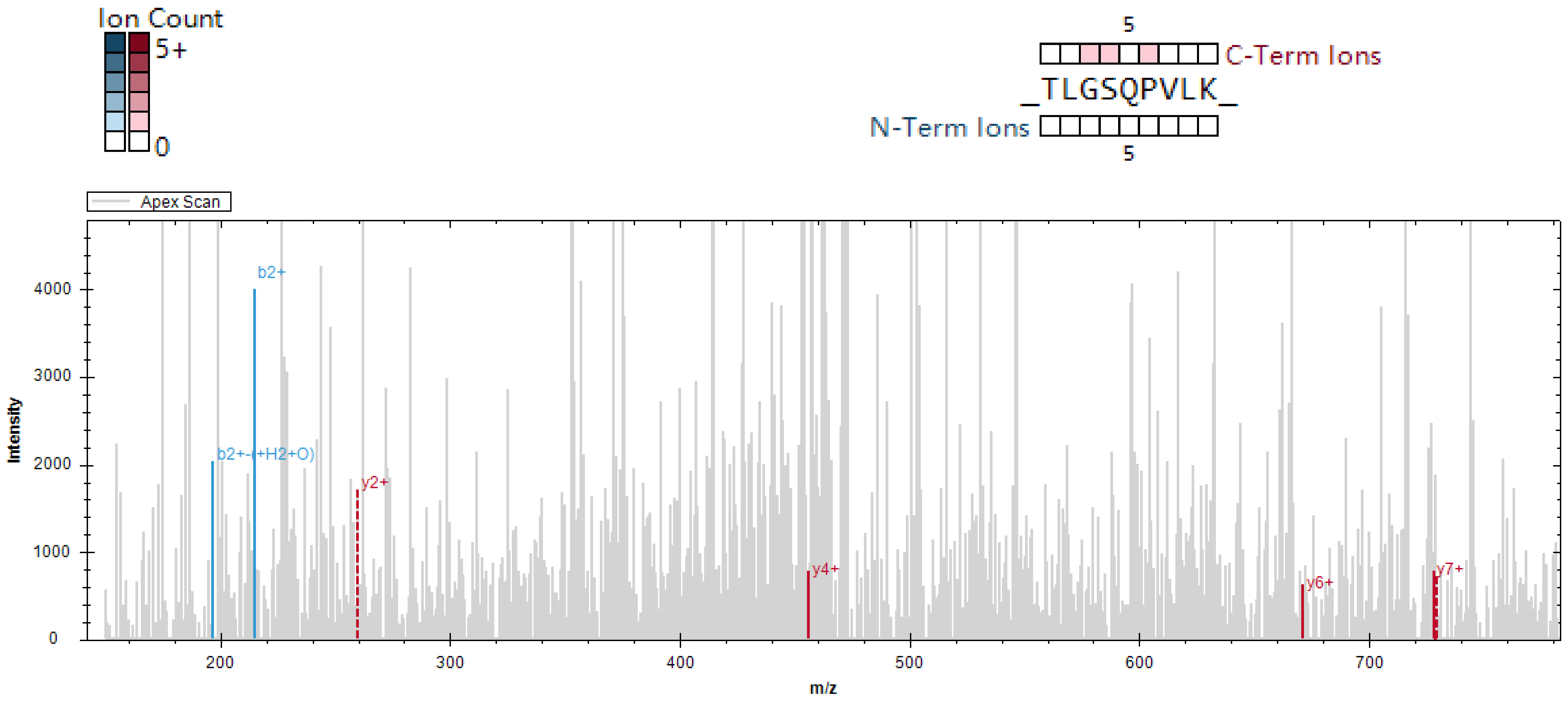

Ion Count
5+
0
5
C-Term Ions
_TLGSQPVLK_
N-Term Ions
5
Apex Scan
b2+
b2+-(+H2+O)
y2+
y4+
y6+
y7+
Intensity
m/z

Q9H832

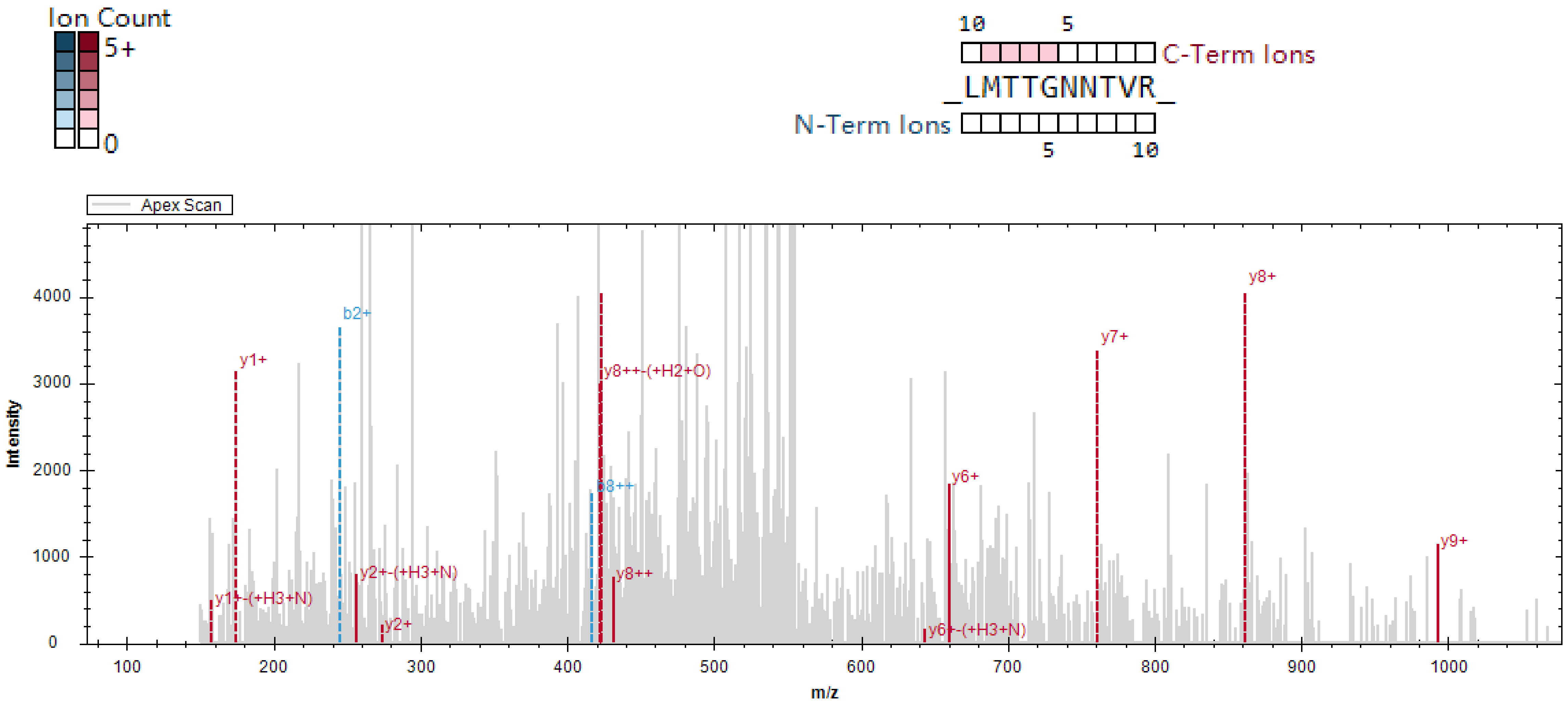

Ion Count
5+
0
10
5
C-Term Ions
_LMTTGNNTVR_
N-Term Ions
5
10
Apex Scan
Intensity
m/z
y1+
y1+-(+H3+N)
b2+
y2+-(+H3+N)
y2+
y8++-(+H2+O)
b8++
y8++
y6+
y6+-(+H3+N)
y7+
y8+
y9+

Q9H8M2

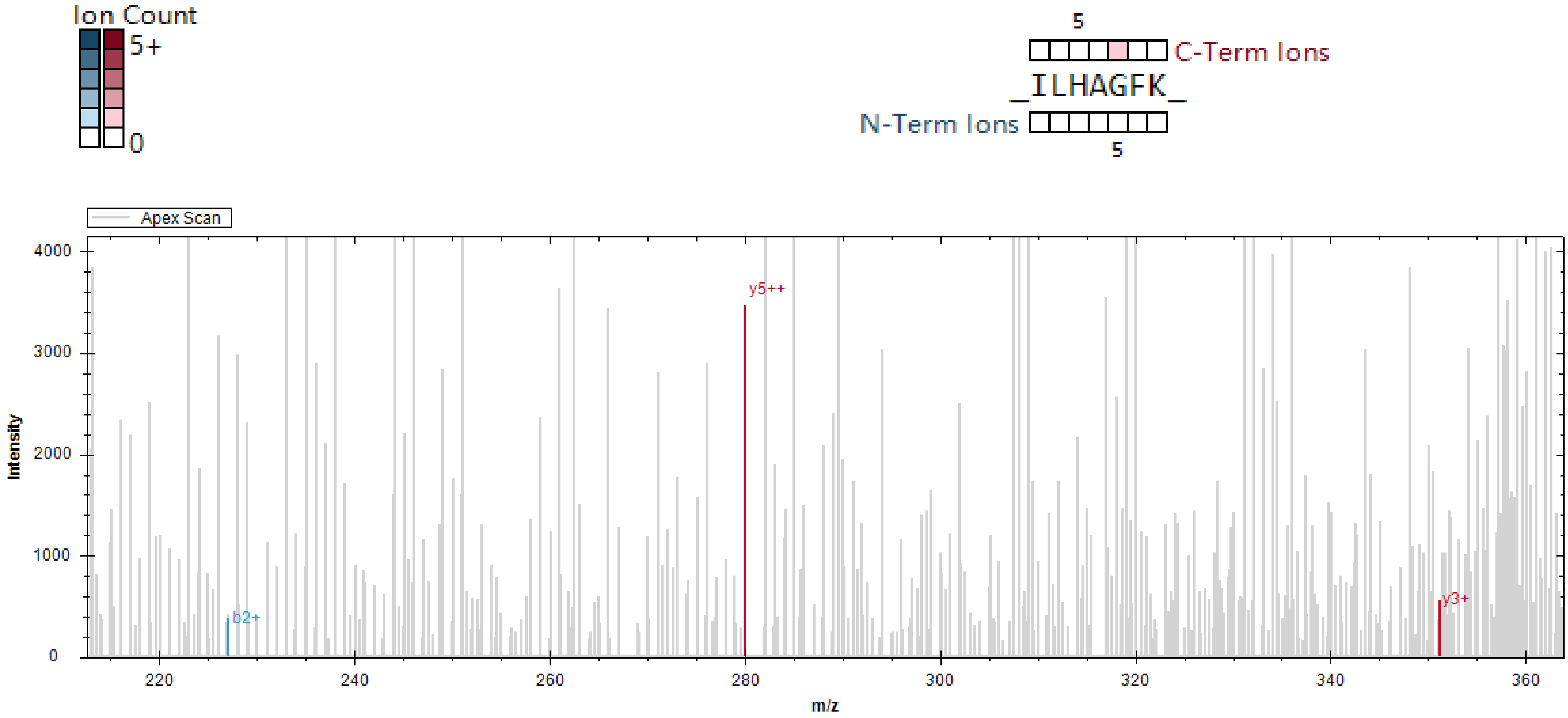

Ion Count
5+
0
5
C-Term Ions
_ILHAGFK_
N-Term Ions
5
Apex Scan
y5++
b2+
y3+
Intensity
m/z
0
1000
2000
3000
4000
220
240
260
280
300
320
340
360

Q9H981

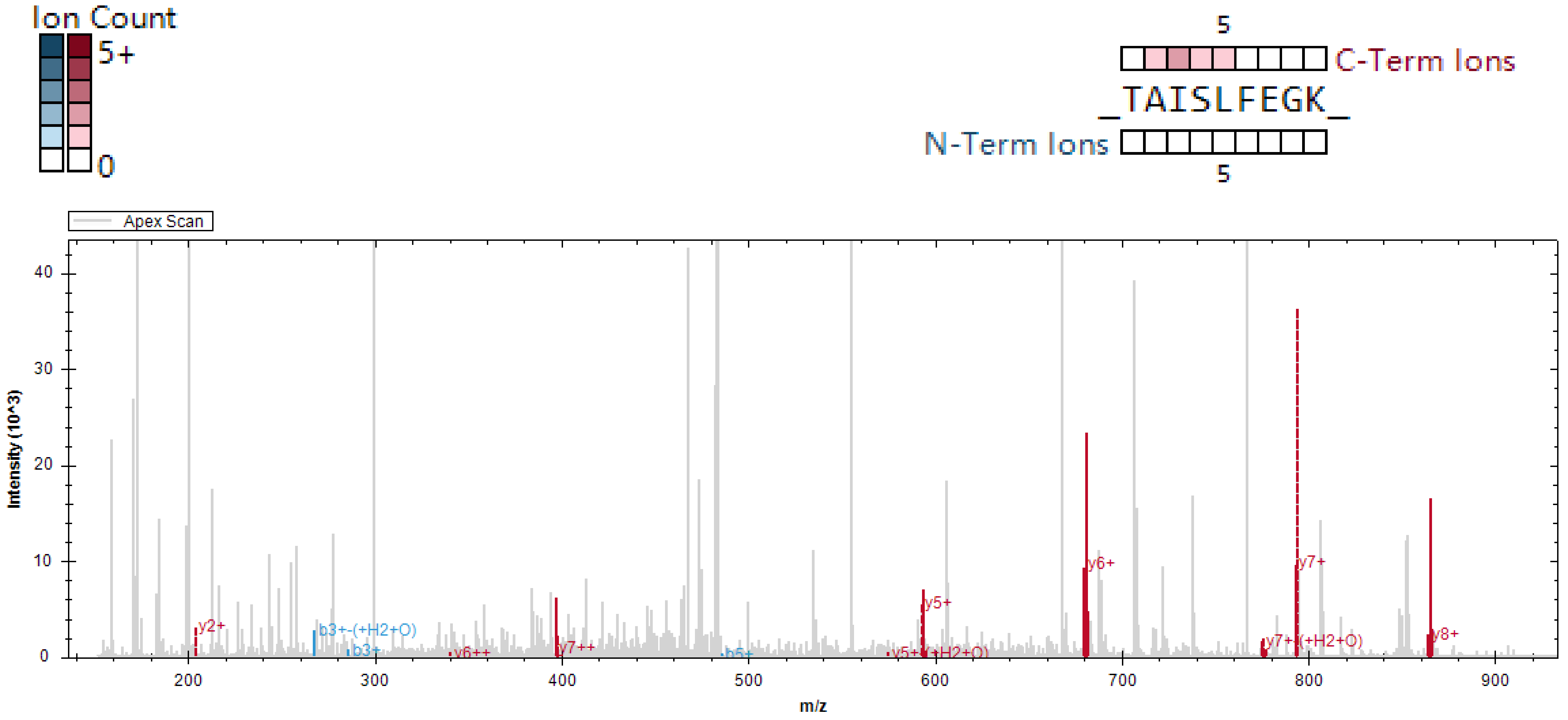

Ion Count
5+
0
5
C-Term Ions
_TAISLFEGK_
N-Term Ions
5
Apex Scan
Intensity (10^3)
m/z
y2+
b3+-(+H2+O)
b3+
y6++
y7++
b5+
y5+
y5+-(+H2+O)
y6+
y7+
y7+-(+H2+O)
y8+

Q9HBE4

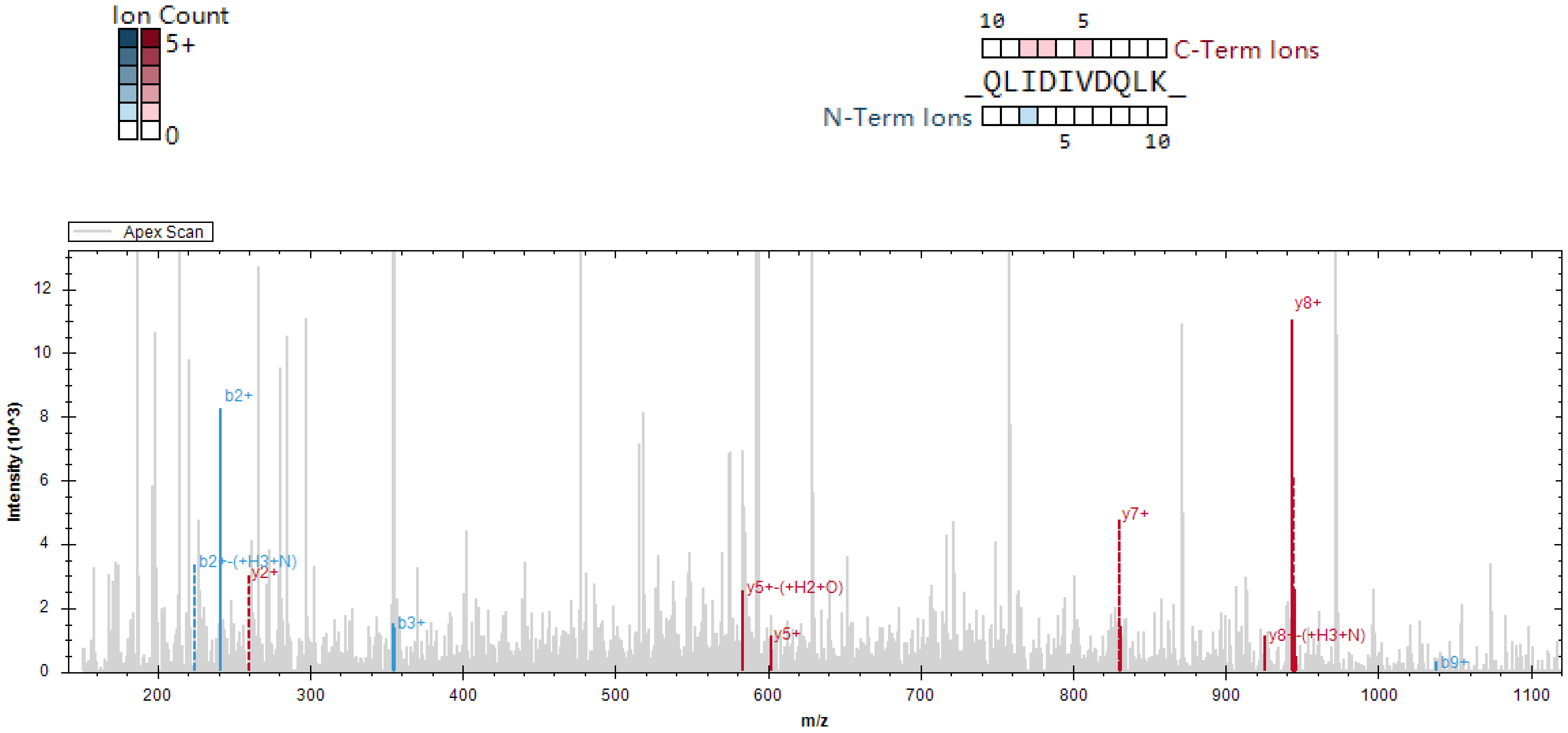

Ion Count
5+
0
10
5
C-Term Ions
_QLIDIVDQLK_
N-Term Ions
5
10
Apex Scan
Intensity (10^3)
m/z
b2+
b2+-(+H3+N)
y2+
b3+
y5+-(+H2+O)
y5+
y7+
y8+
y8+-(+H3+N)
b9+

Q9NPA8

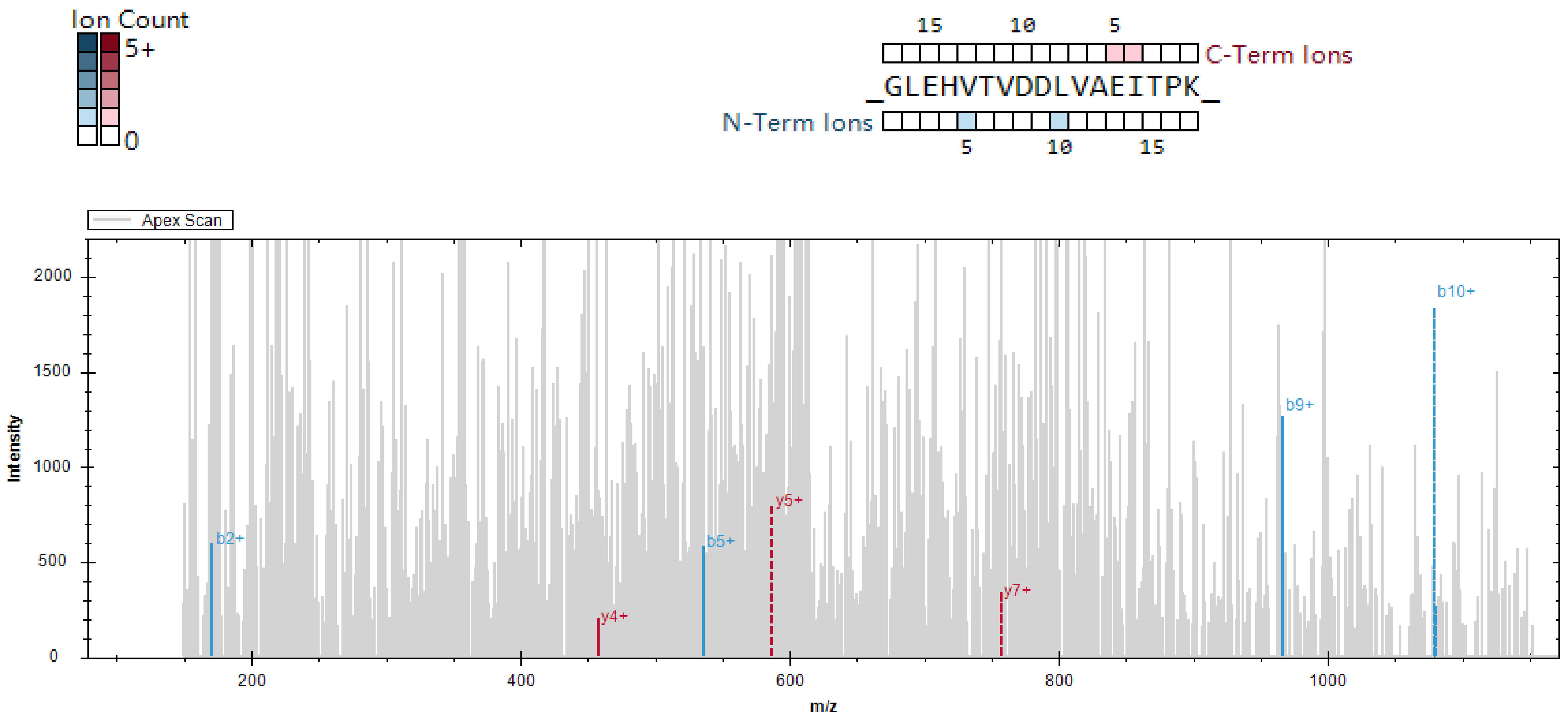
Ion Count
5+
0
15
10
5
C-Term Ions
_GLEHVTVDDLVAEITPK_
N-Term Ions
5
10
15
Apex Scan
Intensity
m/z
b2+
y4+
b5+
y5+
y7+
b9+
b10+
0
500
1000
1500
2000
200
400
600
800
1000

# Q9NQ50

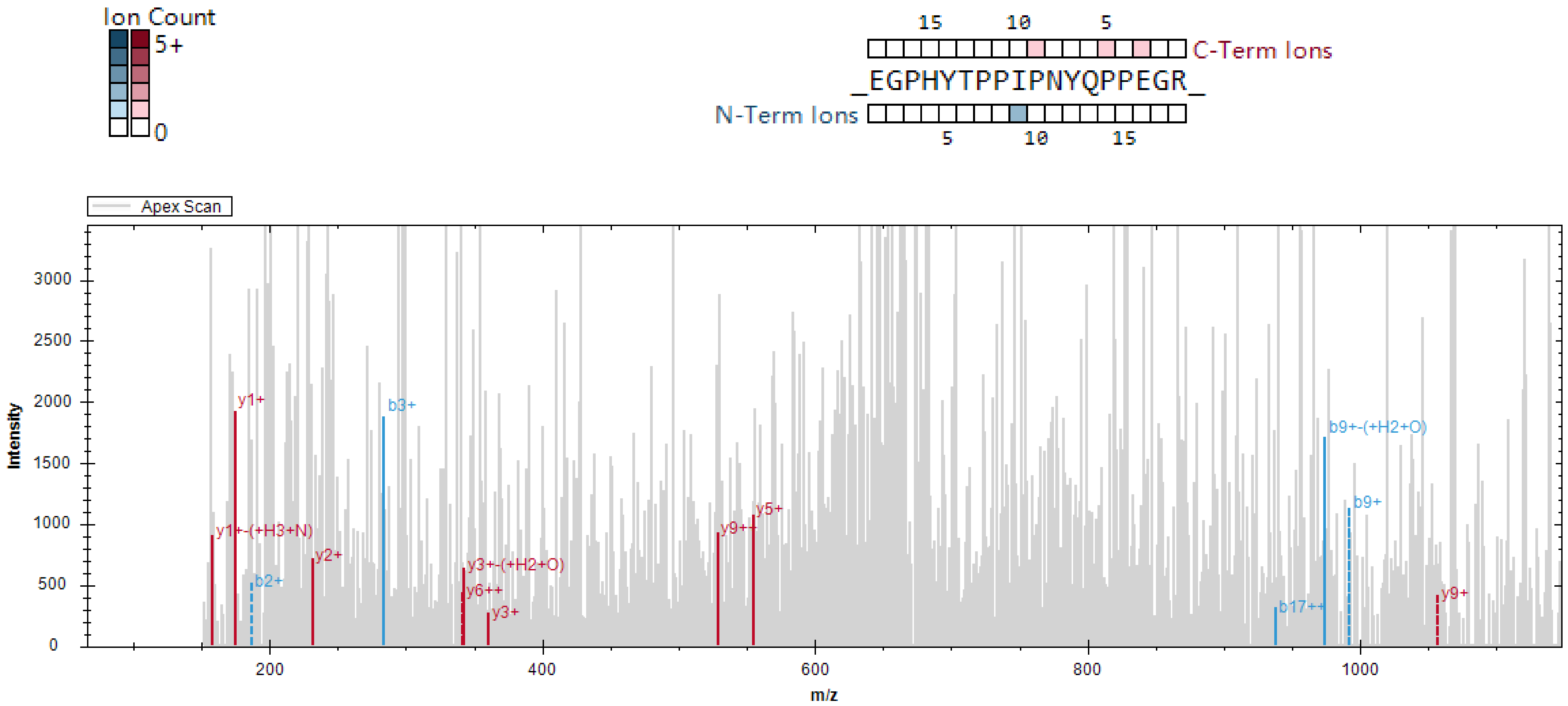

Ion Count
5+
0
15 10 5
C-Term Ions
_EGPHYTPPIPNYQPPEGR_
N-Term Ions
5 10 15
Apex Scan
y1+
y1+-(+H3+N)
b2+
y2+
b3+
y3+-(+H2+O)
y6++
y3+
y9++
y5+
b17++
b9+-(+H2+O)
b9+
y9+
Intensity
m/z

Q9NQ76

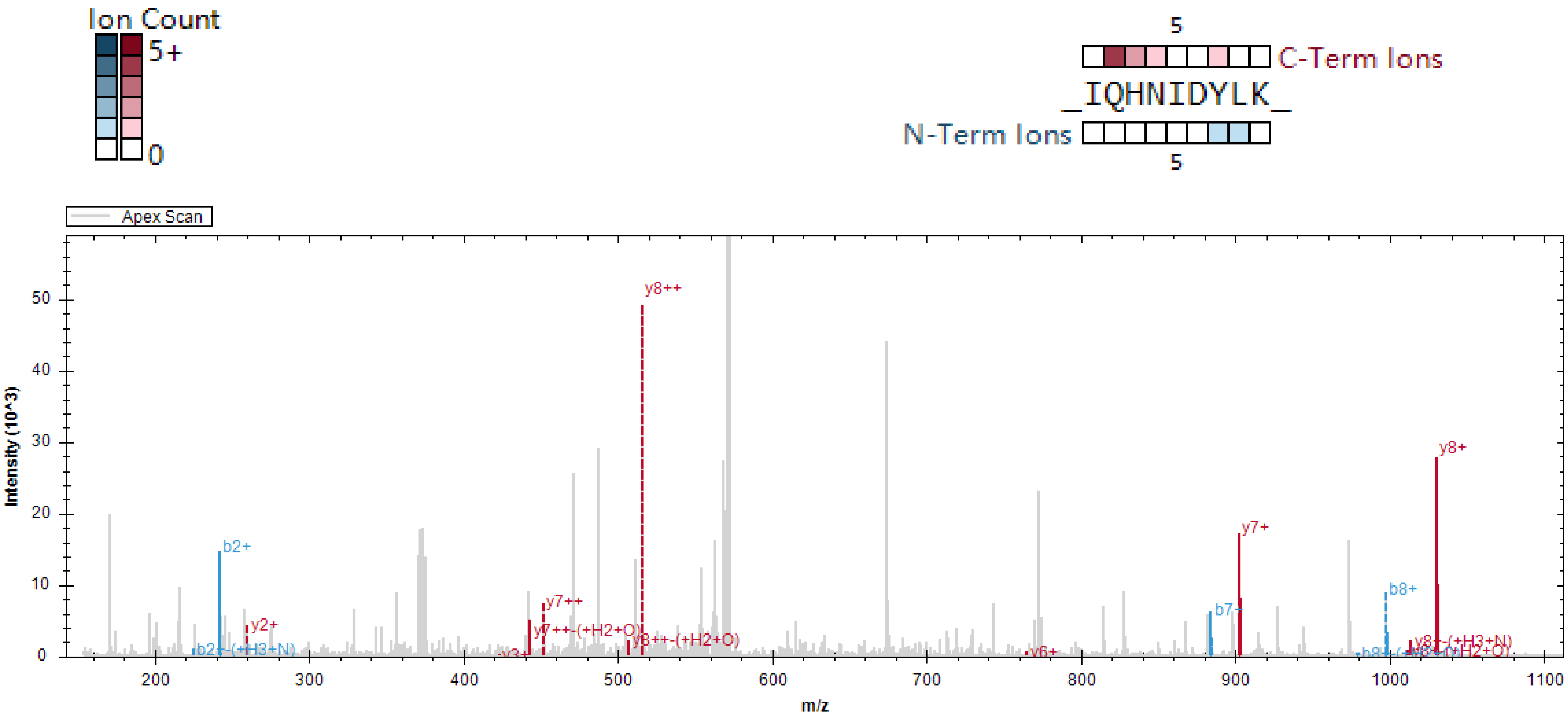

Ion Count
5+
0
5
C-Term Ions
_IQHNIDYLK_
N-Term Ions
5
Apex Scan
Intensity (10^3)
m/z
y8++
y8+
y7+
b2+
b8+
b7+
y7++
y2+
y7++-(+H2+O)
y8++-(+H2+O)
b2+-(+H3+N)
y8+-(+H3+N)
y6+

Q9NQE9

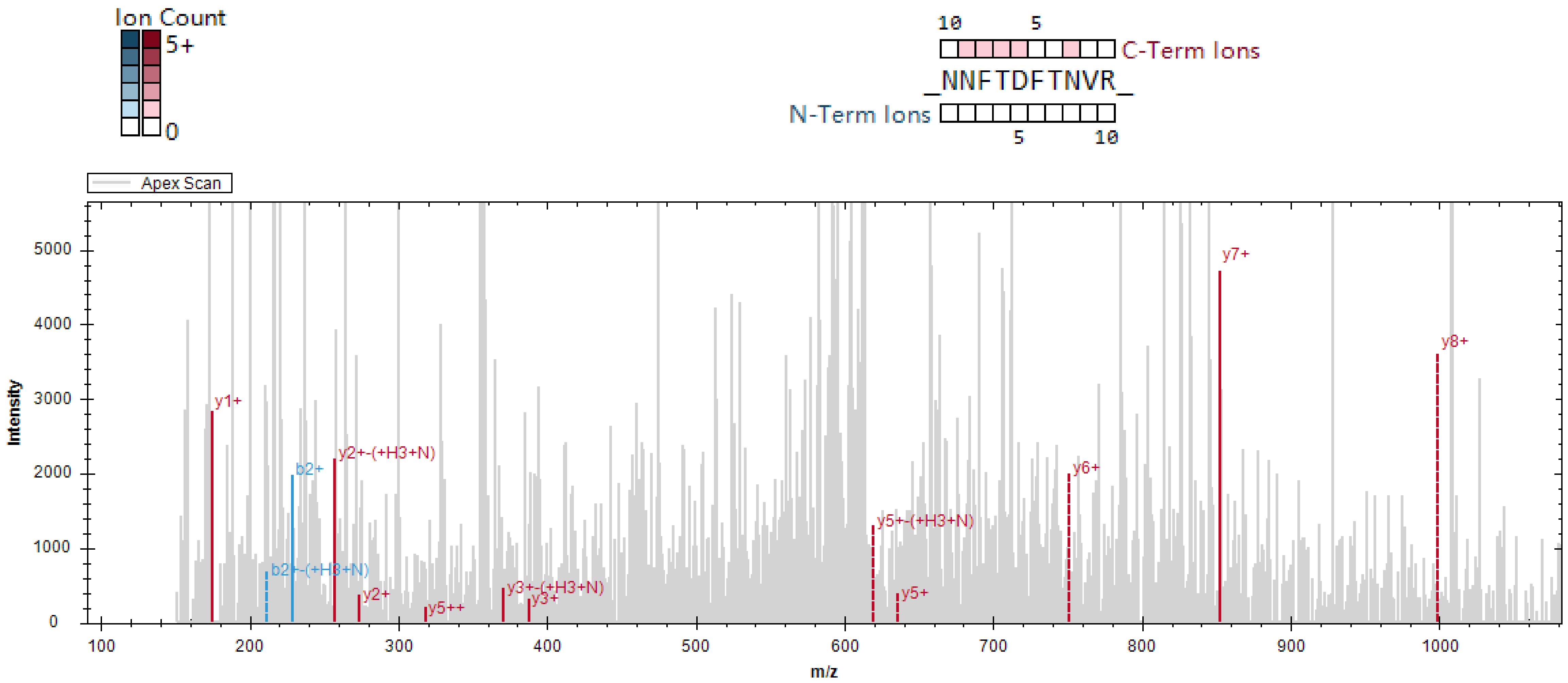

Ion Count
5+
0
10
5
C-Term Ions
_NNFTDFTNVR_
N-Term Ions
5
10
Apex Scan
Intensity
5000
4000
3000
2000
1000
0
y1+
b2+
y2+-(+H3+N)
b2+-(+H3+N)
y2+
y5++
y3+-(+H3+N)
y3+
y5+-(+H3+N)
y5+
y6+
y7+
y8+
100
200
300
400
500
600
700
800
900
1000
m/z

Q9NQS1

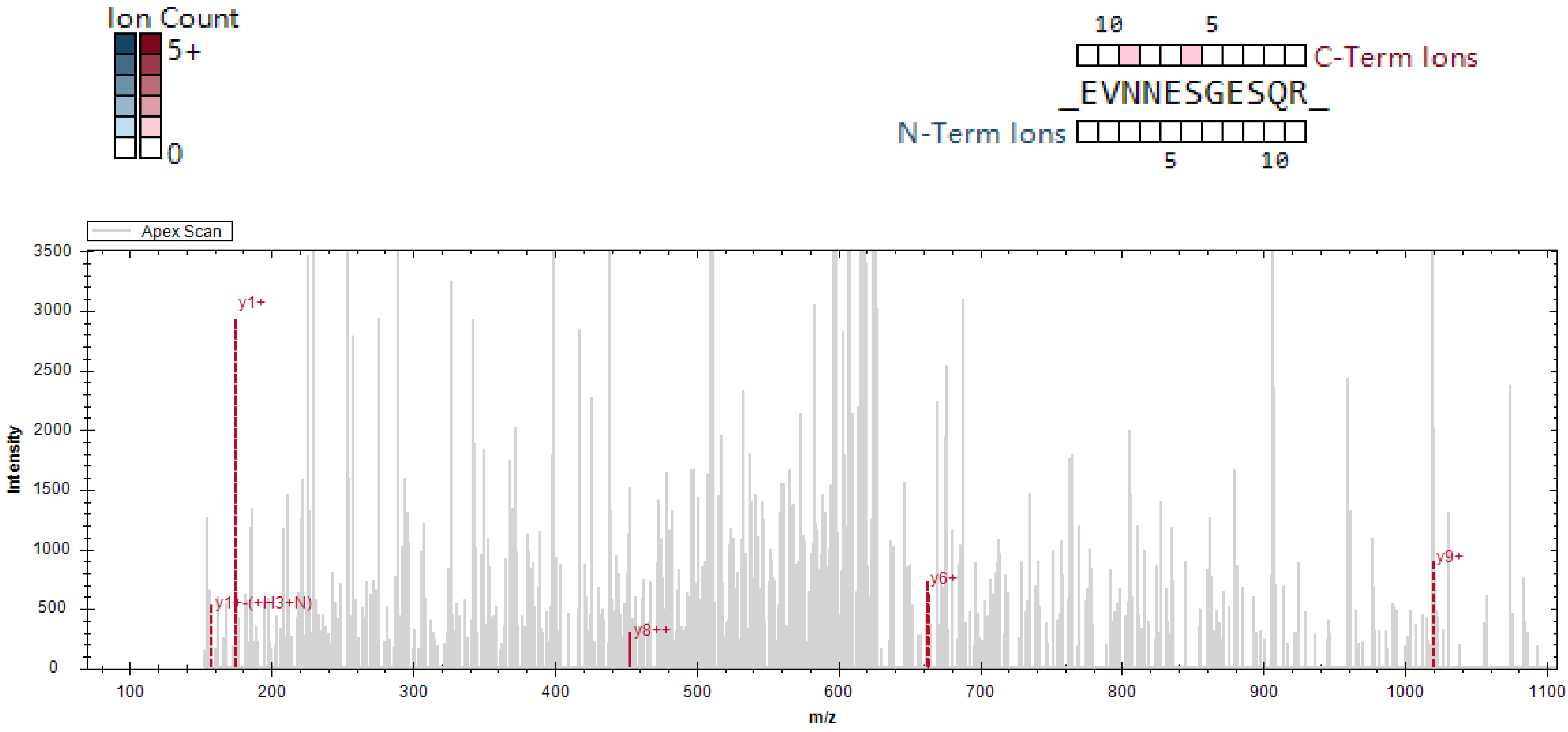

Ion Count
5+
0
10
5
C-Term Ions
_EVNNESGESQR_
N-Term Ions
5
10
Apex Scan
y1+
y1+-(+H3+N)
y8++
y6+
y9+
Intensity
m/z

# Q9NR34

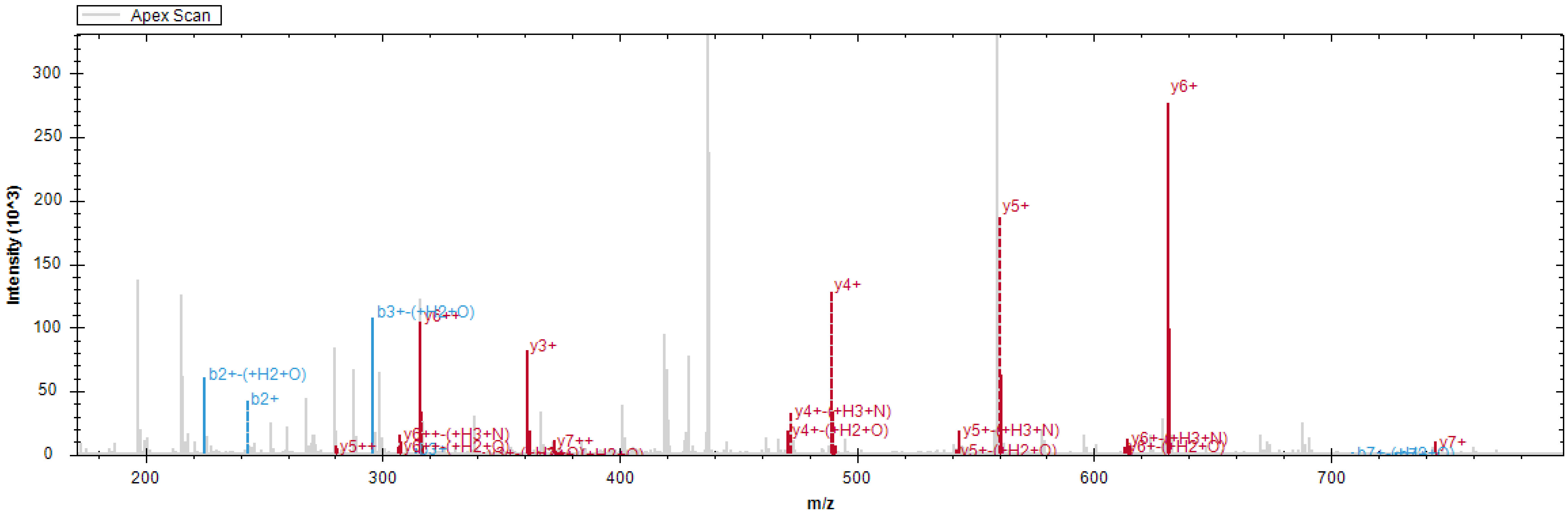

Apex Scan
Intensity (10^3)
m/z
y6+
y5+
y4+
b3+-(+H2+O)
y3+
b2+-(+H2+O)
b2+
y4+-(+H3+N)
y4+-(+H2+O)
y5+-(+H3+N)
y5++
y7++
y7+
0
50
100
150
200
250
300
200
300
400
500
600
700

Q9NRR3

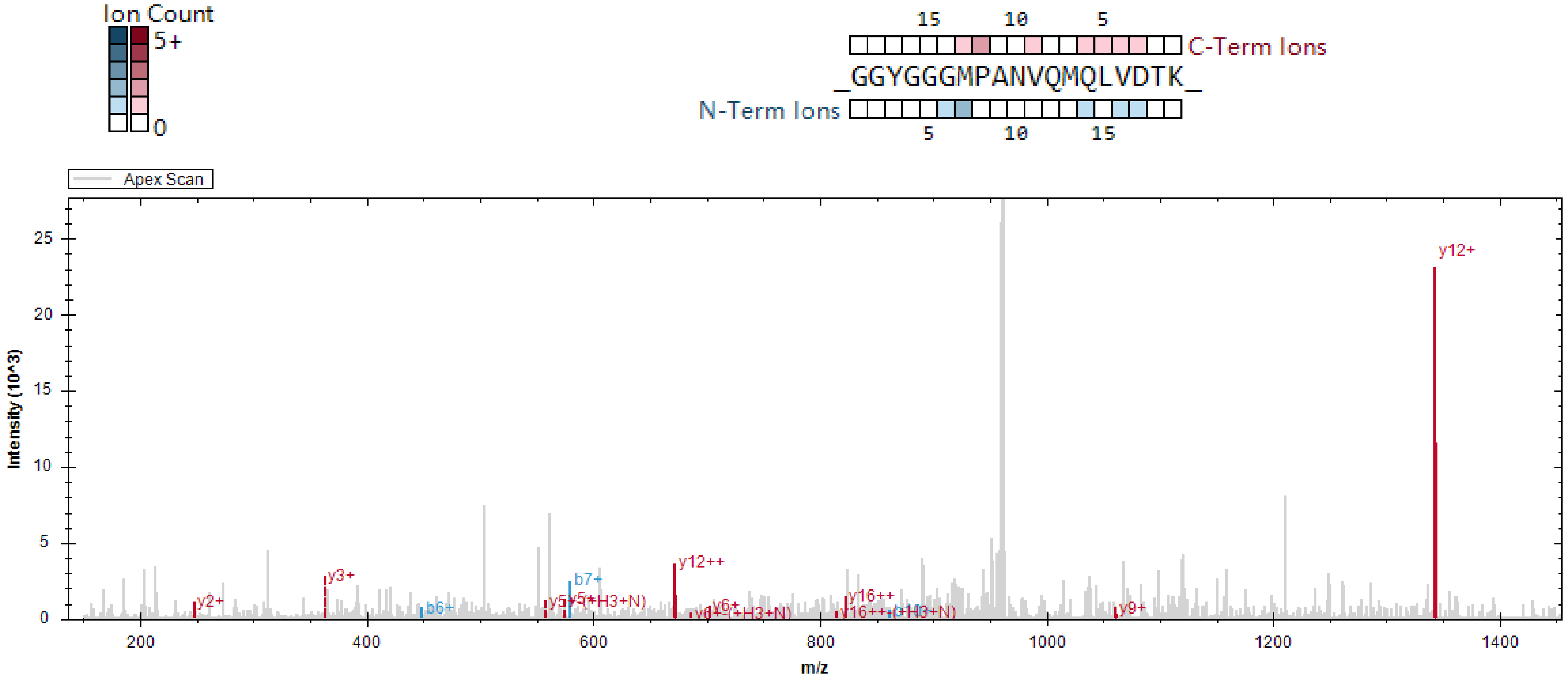

Ion Count
5+
0
15
10
5
C-Term Ions
_GGYGGGMPANVQMQLVDTK_
N-Term Ions
5
10
15
Apex Scan
y12+
y12++
y3+
y2+
b6+
b7+
y9+
Intensity (10^3)
m/z

Q9NUM4

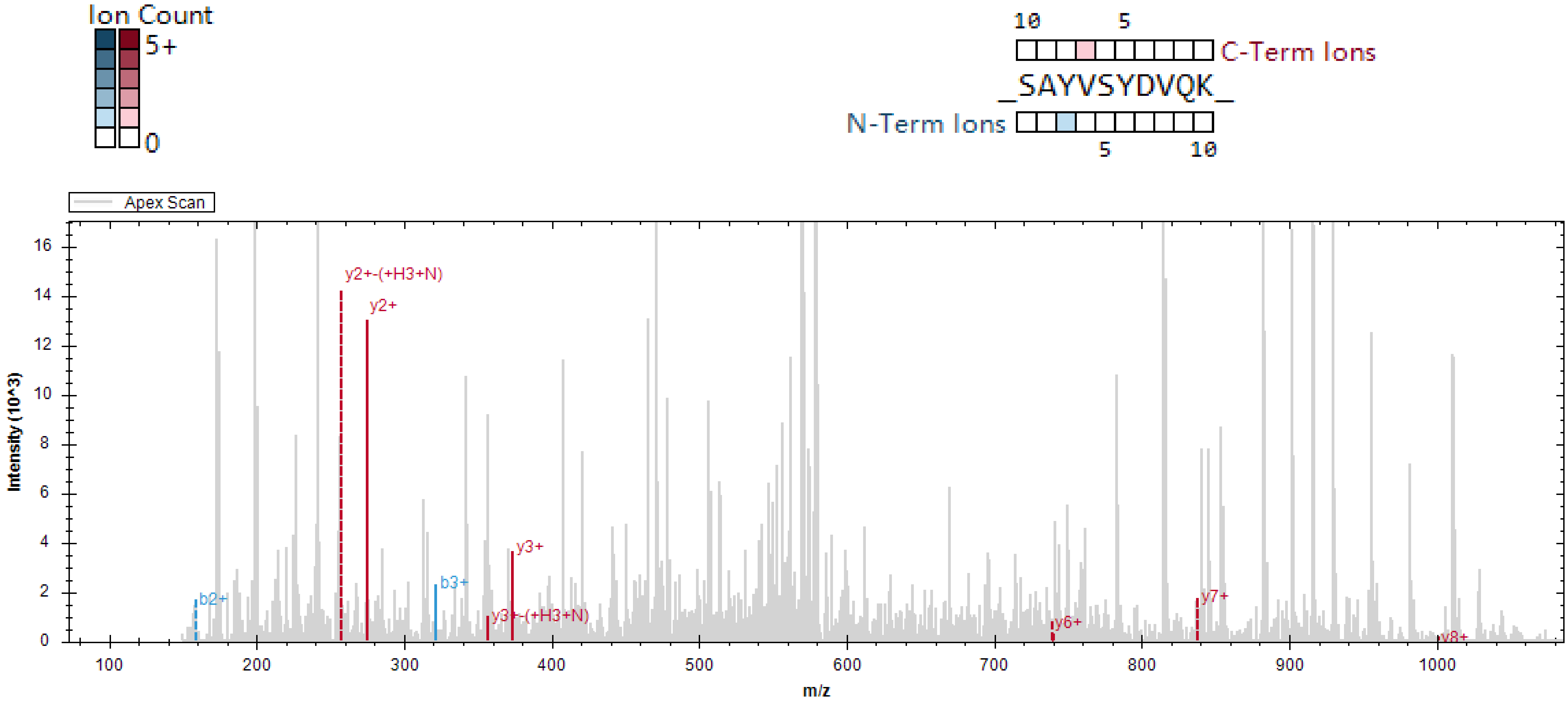
Ion Count
5+
0
10
5
C-Term Ions
_SAYVSYDVQK_
N-Term Ions
5
10
Apex Scan
y2+-(+H3+N)
y2+
y3+
b2+
b3+
y3+-(+H3+N)
y6+
y7+
y8+
Intensity (10^3)
m/z

Q9NUN5

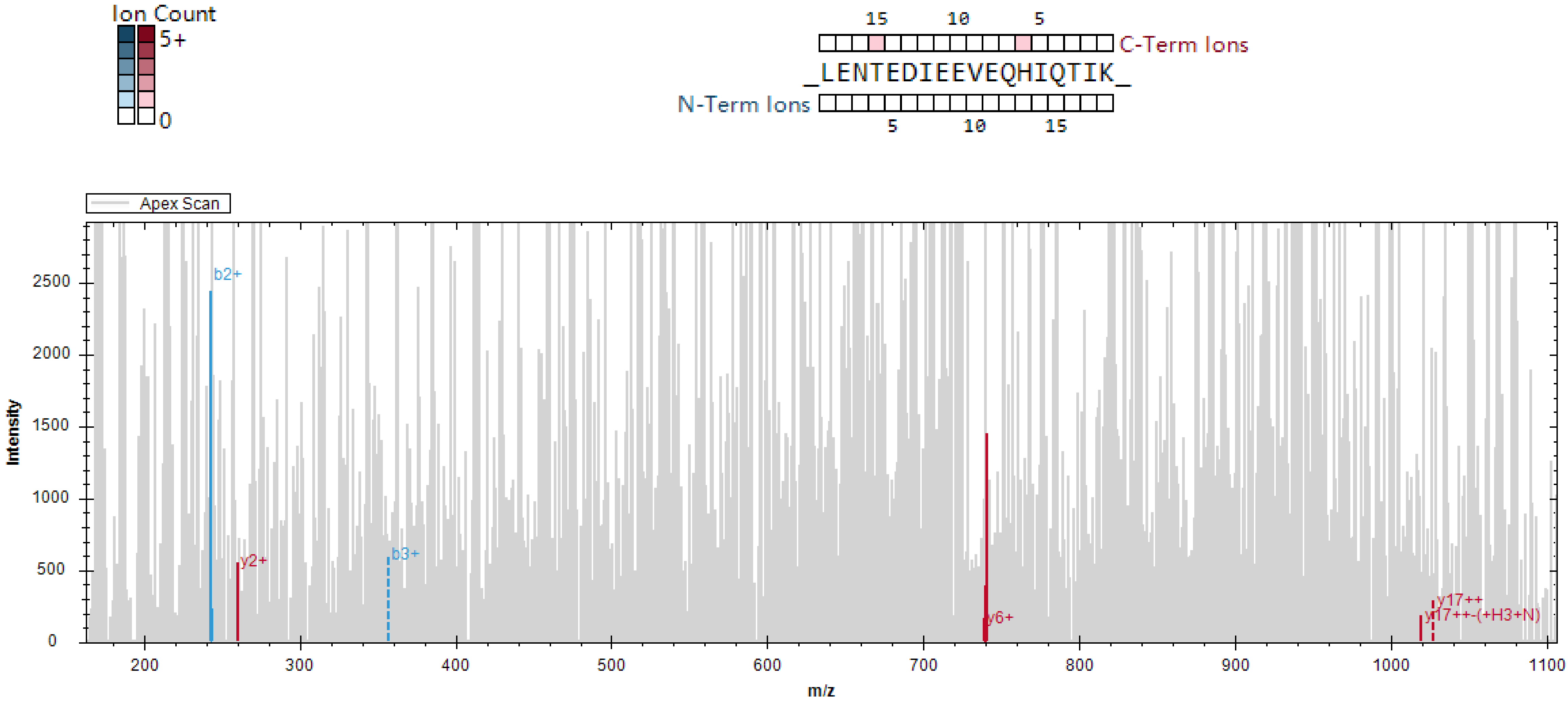

Ion Count
5+
0
15
10
5
C-Term Ions
_LENTEDIEEVEQHIQTIK_
N-Term Ions
5
10
15
Apex Scan
b2+
y2+
b3+
y6+
y17++
y17++-(+H3+N)
Intensity
m/z

Q9NVA1

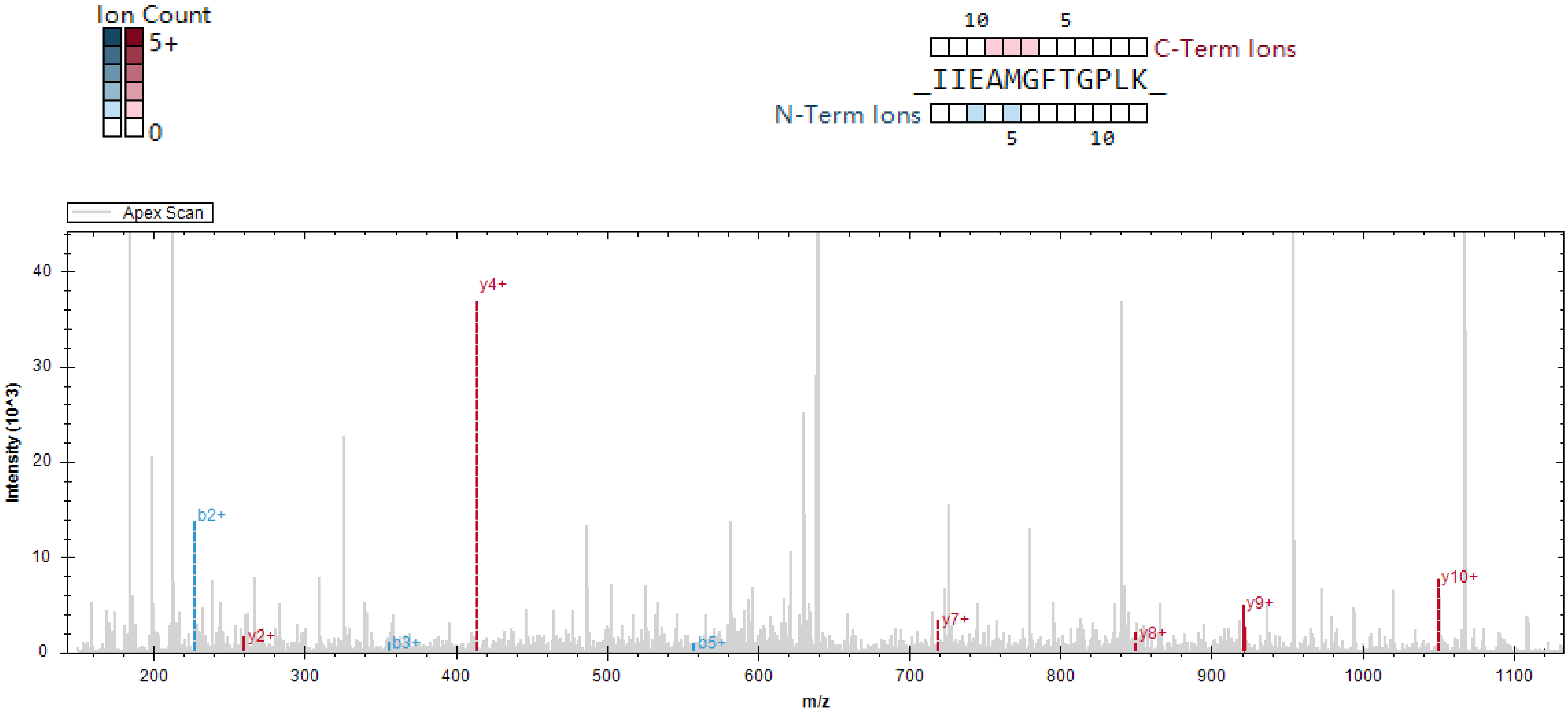

Ion Count
5+
0
10
5
C-Term Ions
_IIEAMGFTGPLK_
N-Term Ions
5
10
Apex Scan
Intensity (10^3)
m/z
y4+
b2+
y2+
b3+
b5+
y7+
y8+
y9+
y10+

Q9NVV5

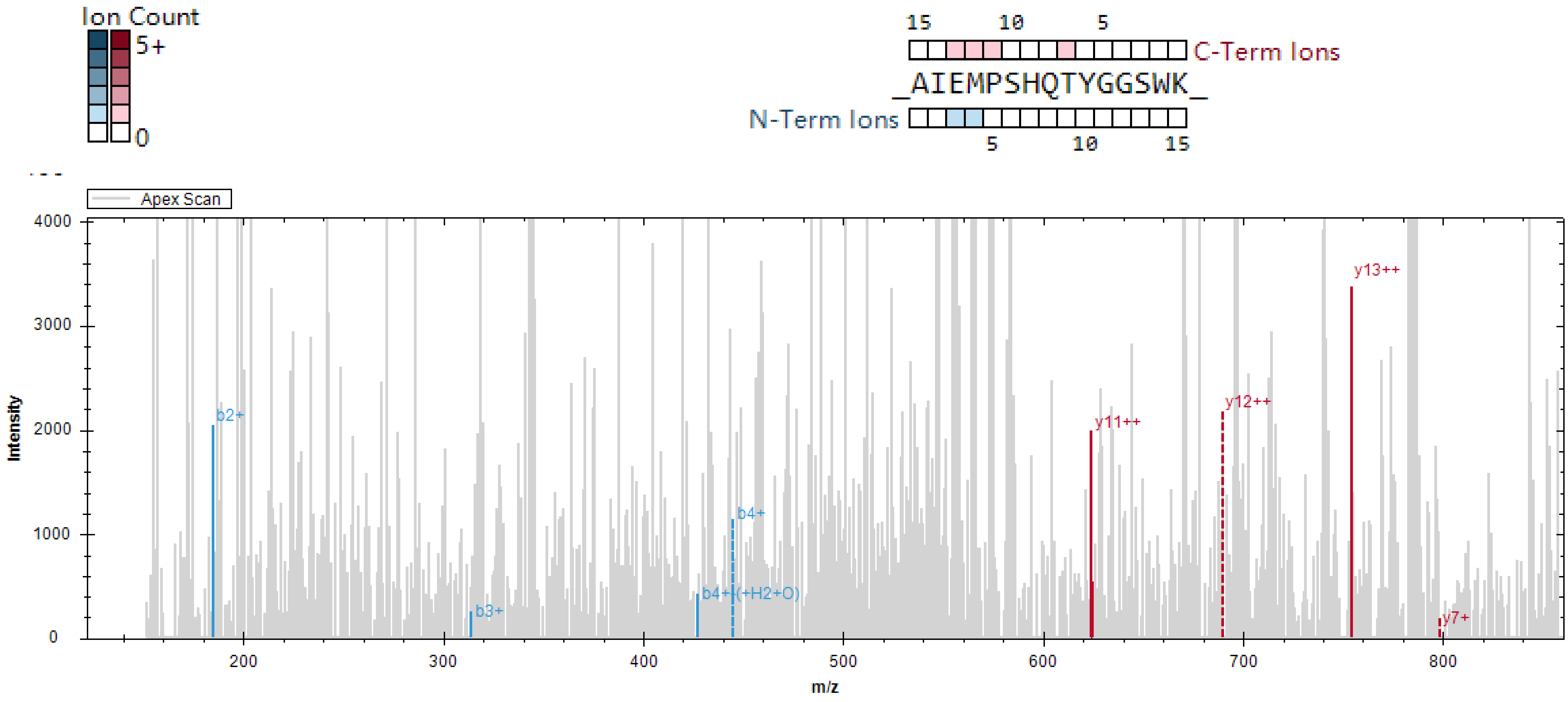
Ion Count
5+
0
15 10 5
C-Term Ions
_AIEMPSHQTYGGSWK_
N-Term Ions
5 10 15
Apex Scan
Intensity
m/z
b2+
b3+
b4+
b4+(+H2+O)
y11++
y12++
y13++
y7+

# Q9NVZ3

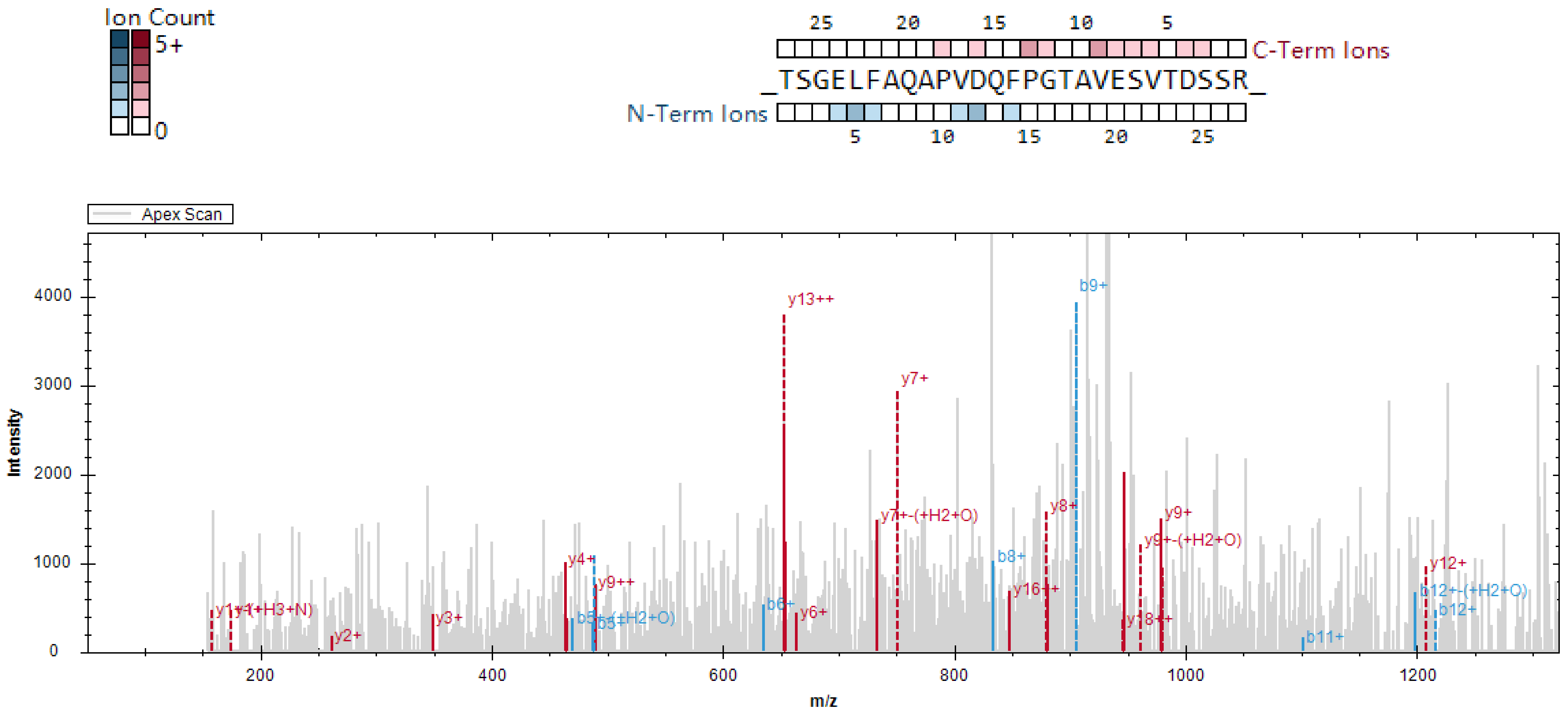

Ion Count
5+
0
C-Term Ions
_TSGELFAQAPVDQFPGTAVESVTDSSR_
N-Term Ions
Apex Scan
Intensity
m/z
y13++
b9+
y7+
y7+-(+H2+O)
y8+
y9+
y9+-(+H2+O)
b8+
y4+
y9++
y16++
y12+
b12+-(+H2+O)
b12+
b6+
y6+
y3+
y2+
y18++
b11+

Q9NWX5

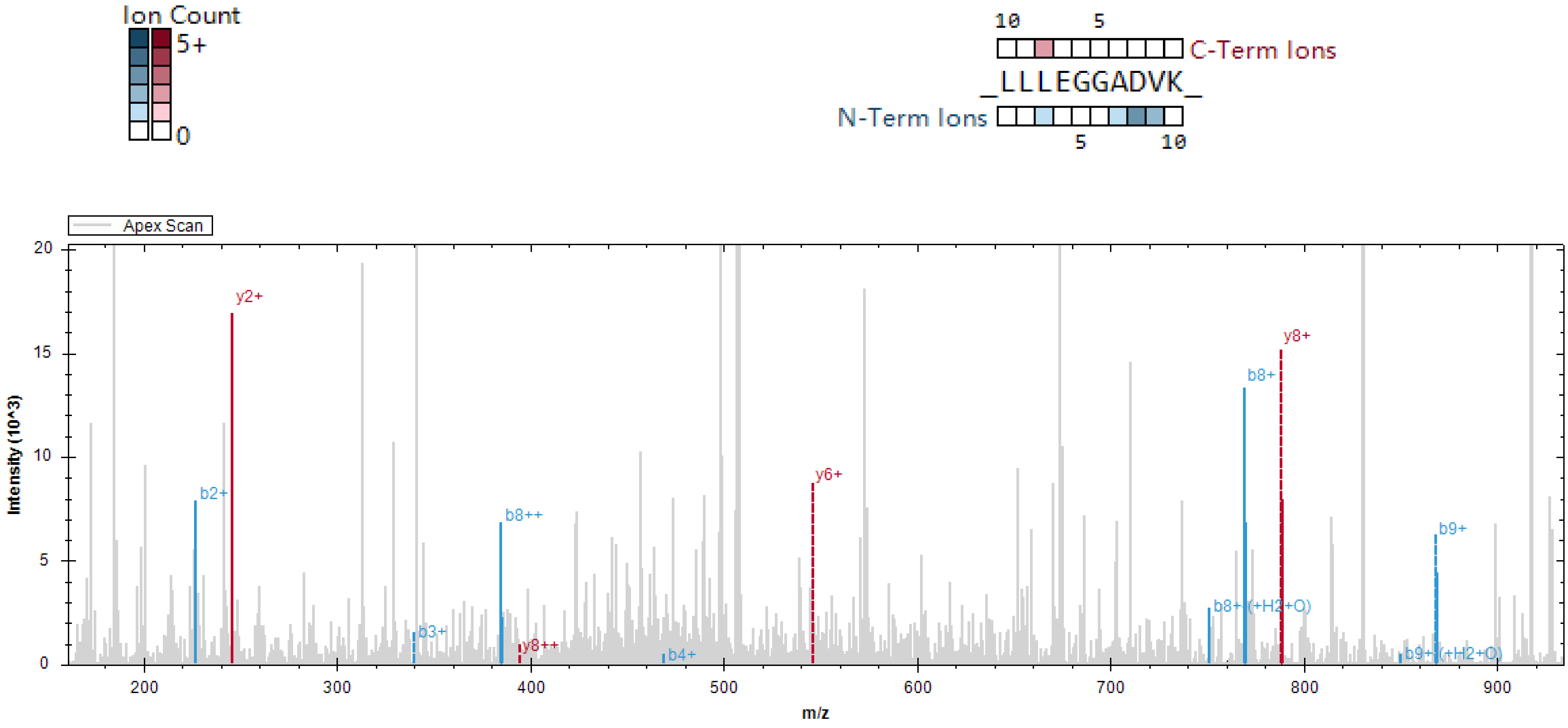

Ion Count
5+
0
10
5
C-Term Ions
_LLLEGGADVK_
N-Term Ions
5
10
Apex Scan
y2+
y8+
b8+
y6+
b2+
b8++
b9+
b8+ (+H2+O)
b3+
y8++
b4+
b9+ (+H2+O)
Intensity (10^3)
m/z
0
5
10
15
20
200
300
400
500
600
700
800
900

# Q9NWX6

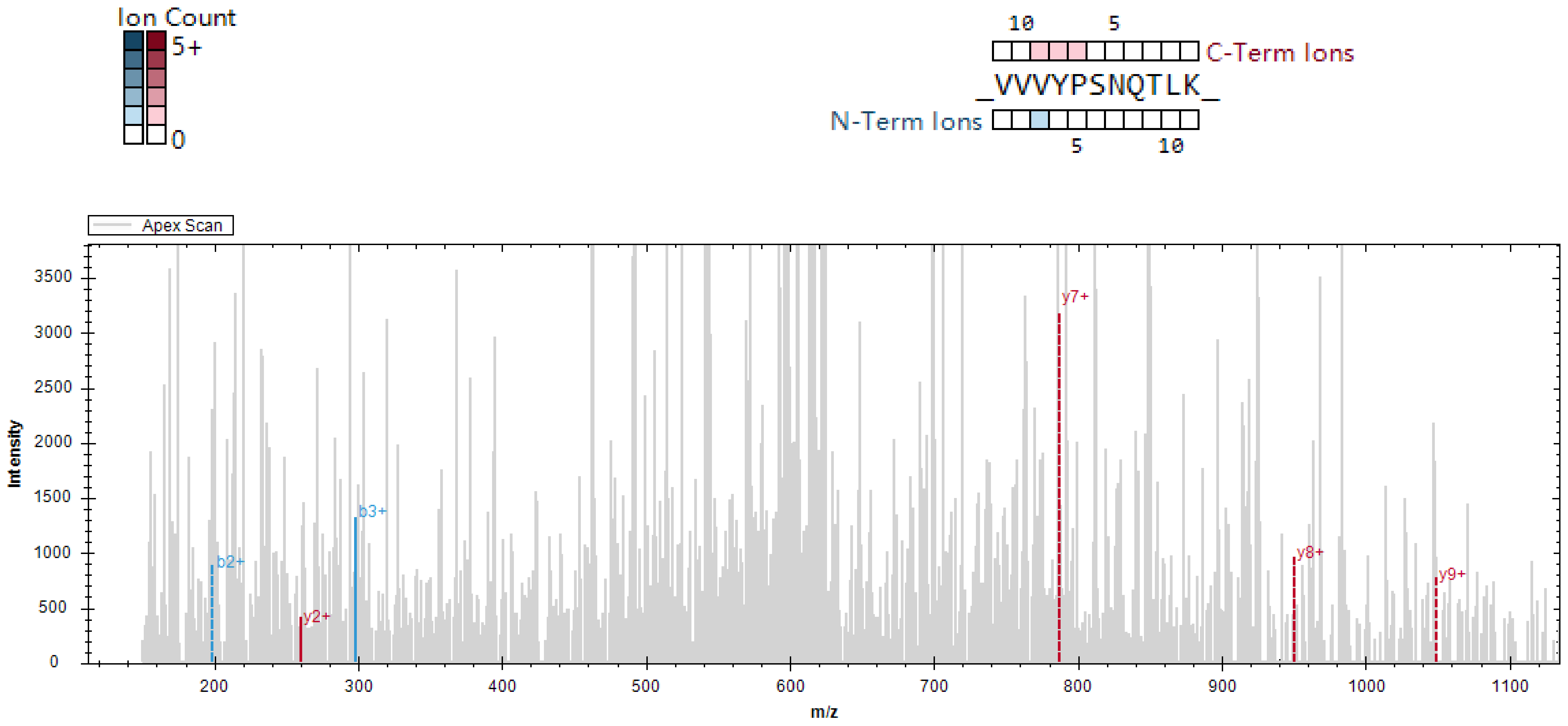

Ion Count
5+
0
10
5
C-Term Ions
_VVVYPSNQTLK_
N-Term Ions
5
10
Apex Scan
Intensity
m/z
y7+
y8+
y9+
y2+
b2+
b3+

Q9NXS2

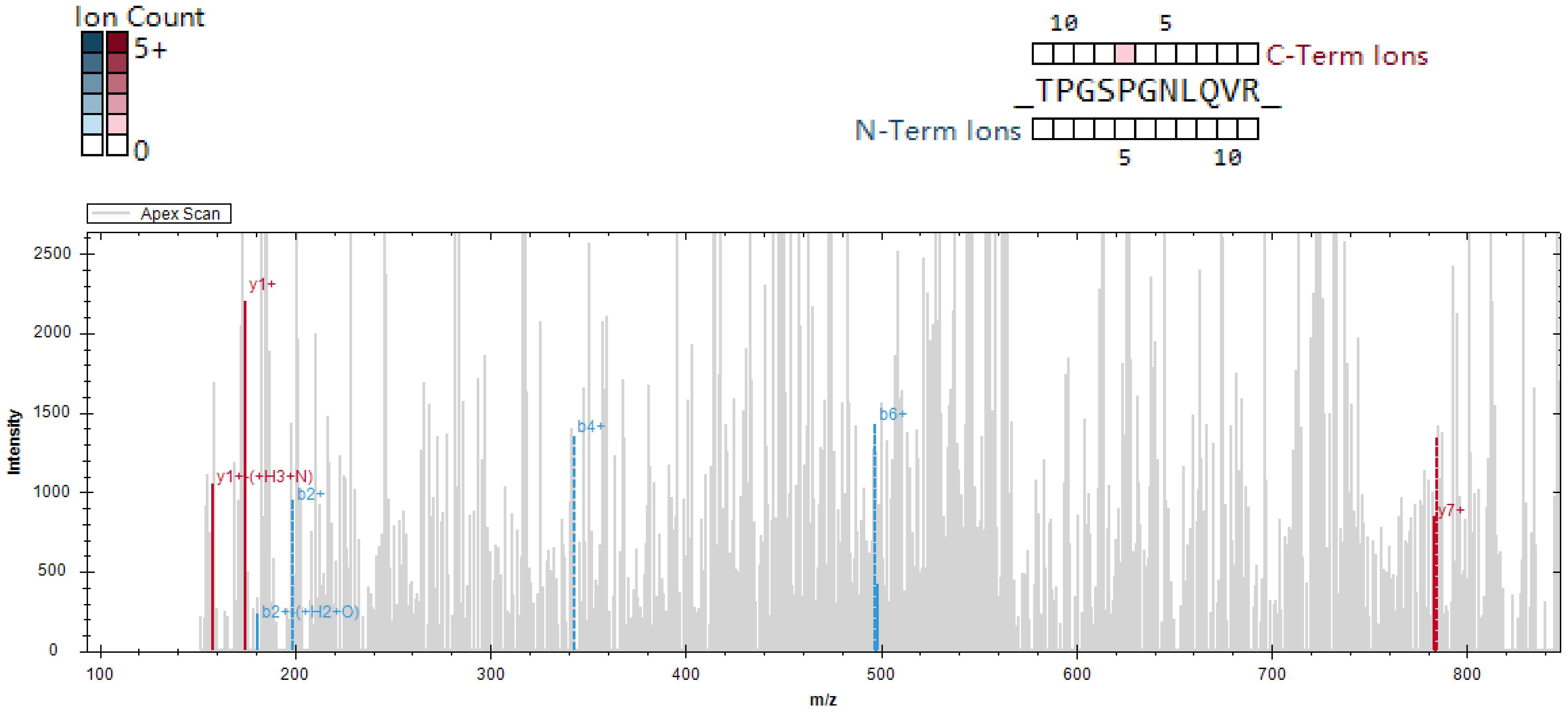

Ion Count
5+
0
10
5
C-Term Ions
_TPGSPGNLQVR_
N-Term Ions
5
10
Apex Scan
Intensity
m/z
y1+
y1+-(+H3+N)
b2+
b2+-(+H2+O)
b4+
b6+
y7+

Q9NYZ1

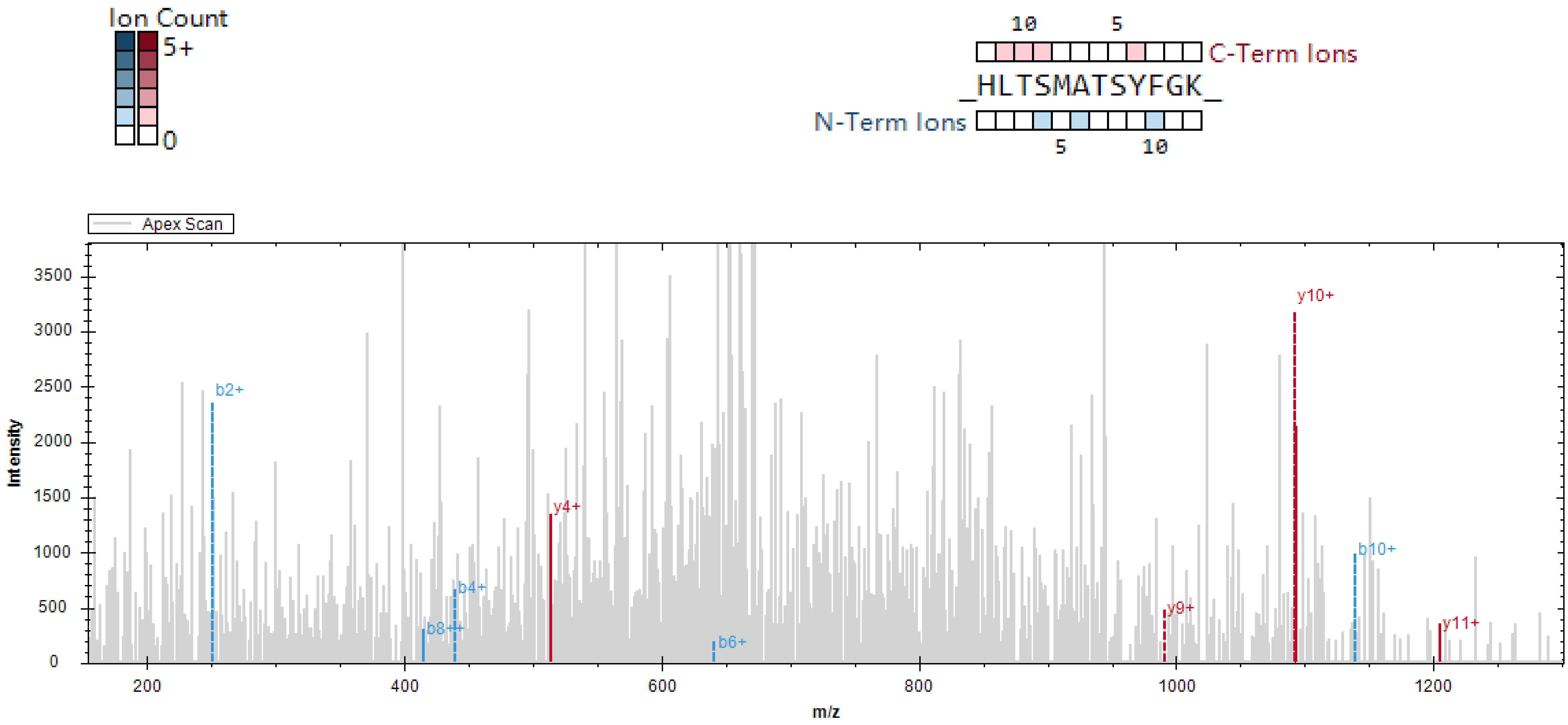

Ion Count
5+
0
10
5
C-Term Ions
_HLTSMATSYFGK_
N-Term Ions
5
10
Apex Scan
Intensity
m/z
b2+
b4+
b6+
b8++
b10+
y4+
y9+
y10+
y11+

Q9NZ42

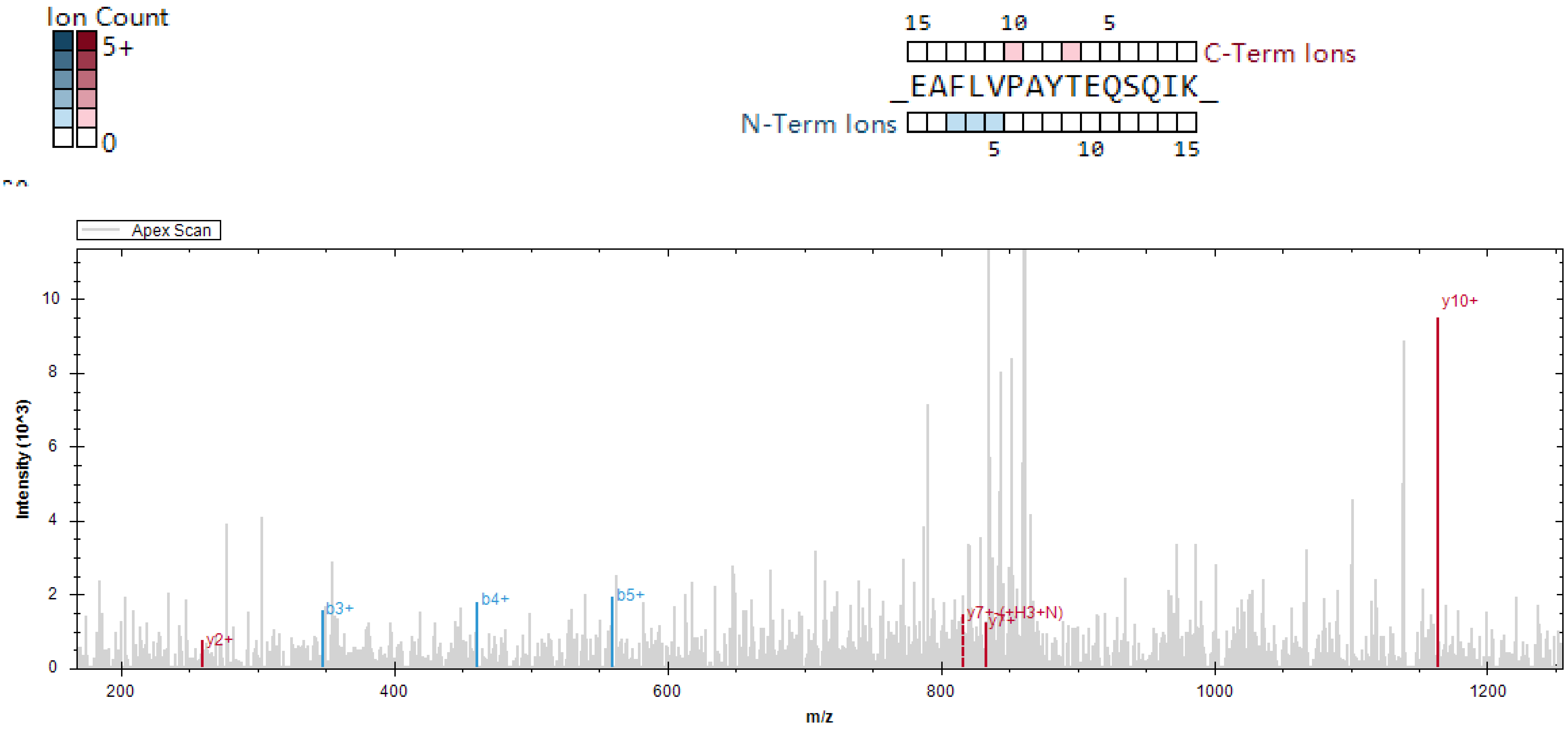

Ion Count
5+
0
15
10
5
C-Term Ions
_EAFLVPAYTEQSQIK_
N-Term Ions
5
10
15
Apex Scan
Intensity (10^3)
m/z
y2+
b3+
b4+
b5+
y7+-(+H3+N)
y7+
y10+

Q9NZM1

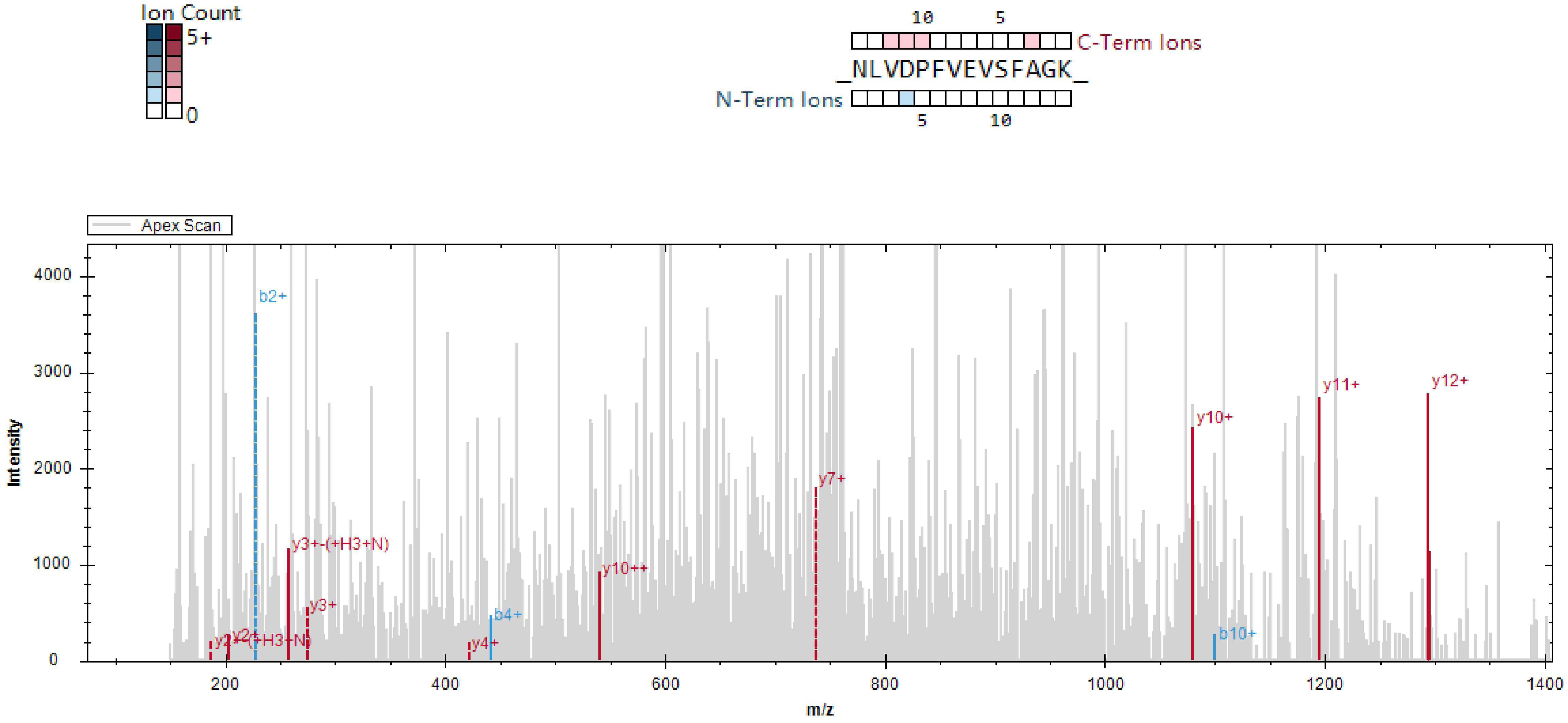

Ion Count
5+
0
10
5
C-Term Ions
_NLVDPFVEVSFAGK_
N-Term Ions
5
10
Apex Scan
Intensity
m/z
b2+
y3+-(+H3+N)
y3+
y2+
y2+-(+H3+N)
y4+
b4+
y10++
y7+
y10+
b10+
y11+
y12+

# Q9P2J2

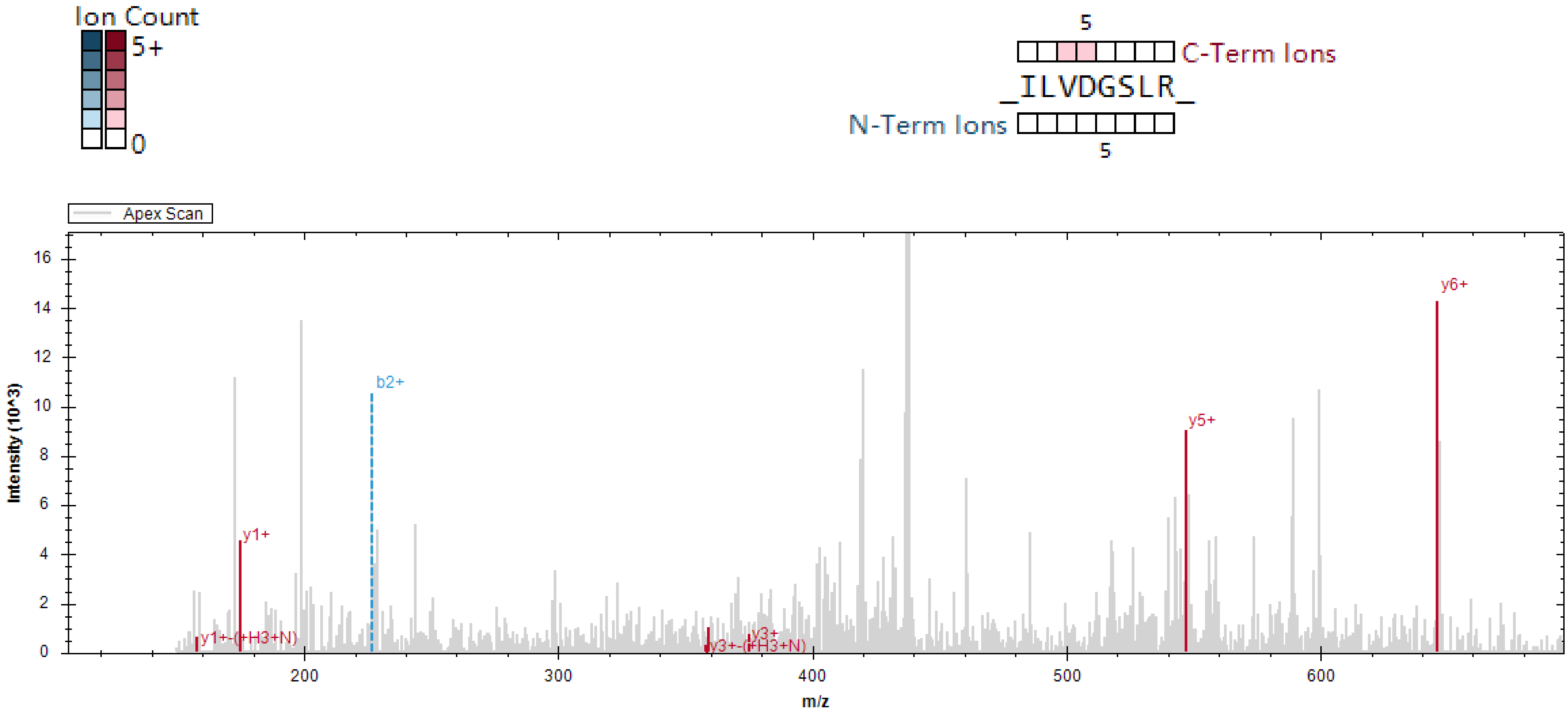

Ion Count
5+
0
5
C-Term Ions
_ILVDGSLR_
N-Term Ions
5
Apex Scan
Intensity (10^3)
m/z
y1+
y1+-(+H3+N)
b2+
y3+
y3+-(+H3+N)
y5+
y6+

# Q9P2S5

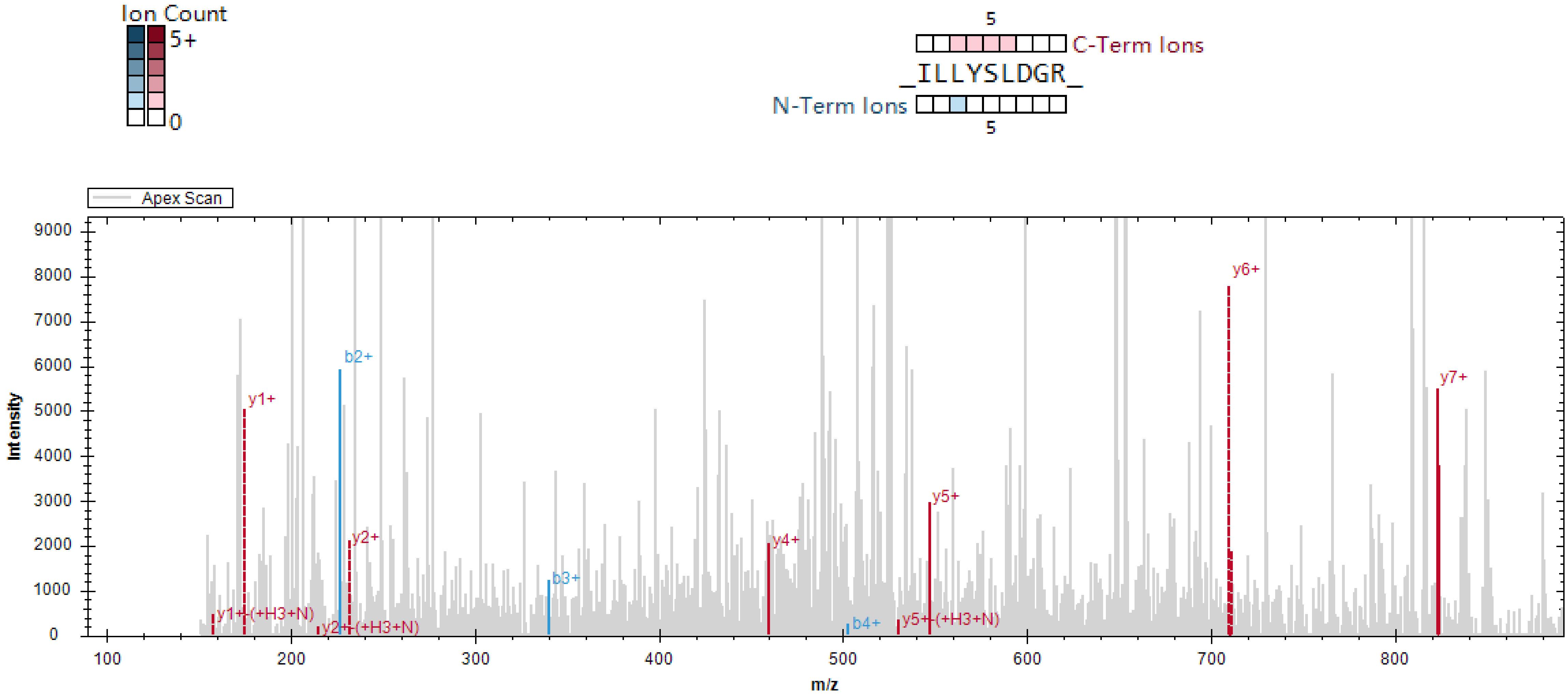

Ion Count
5+
0
5
C-Term Ions
_ILLYSLDGR_
N-Term Ions
5
Apex Scan
Intensity
m/z
y1+
b2+
y2+
b3+
y4+
y5+
y6+
y7+
b4+
y1+-(+H3+N)
y2+-(+H3+N)
y5+-(+H3+N)

# Q9UBV7

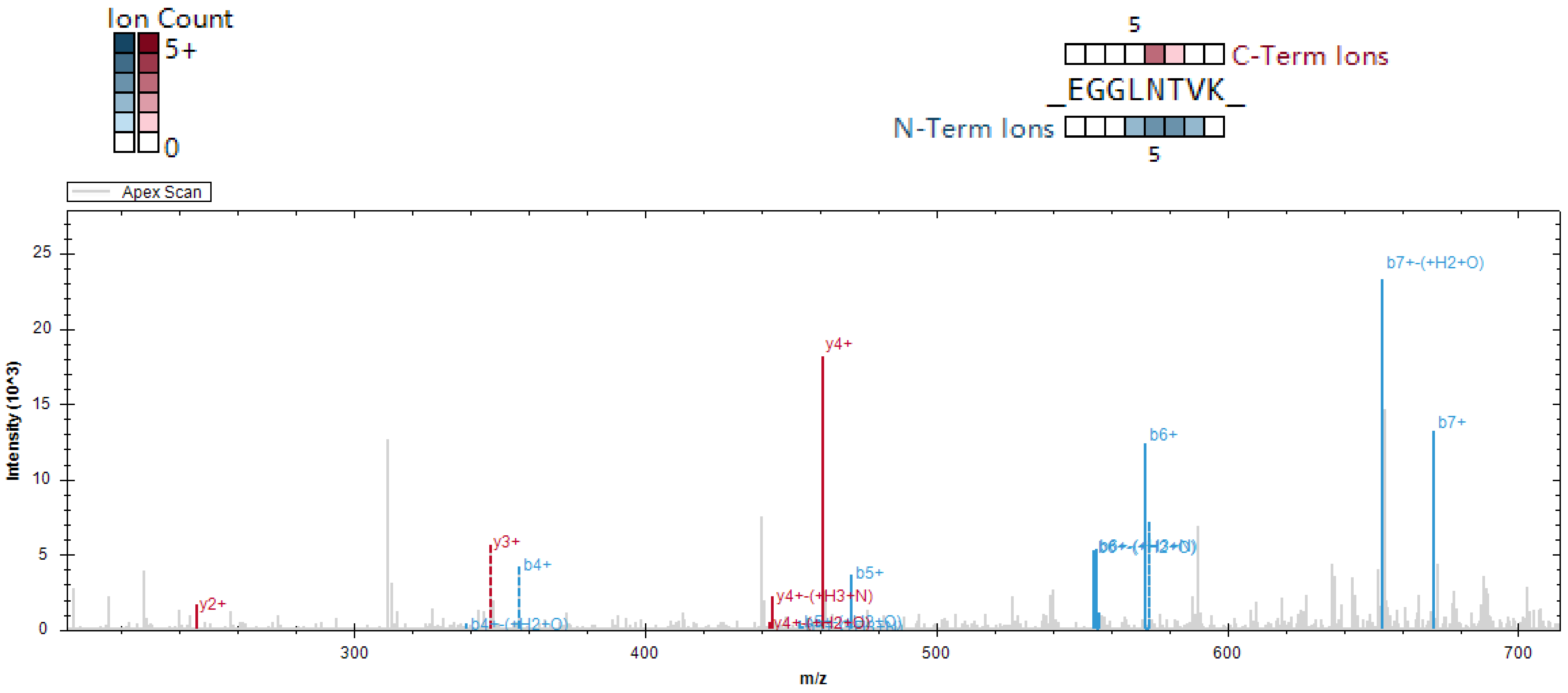

Ion Count
5+
0
5
C-Term Ions
_EGGLNTVK_
N-Term Ions
5
Apex Scan
Intensity (10^3)
m/z
y2+
y3+
b4+
y4+
y4+-(+H3+N)
b5+
b6+
b7+
b7+-(+H2+O)

Q9UBY5

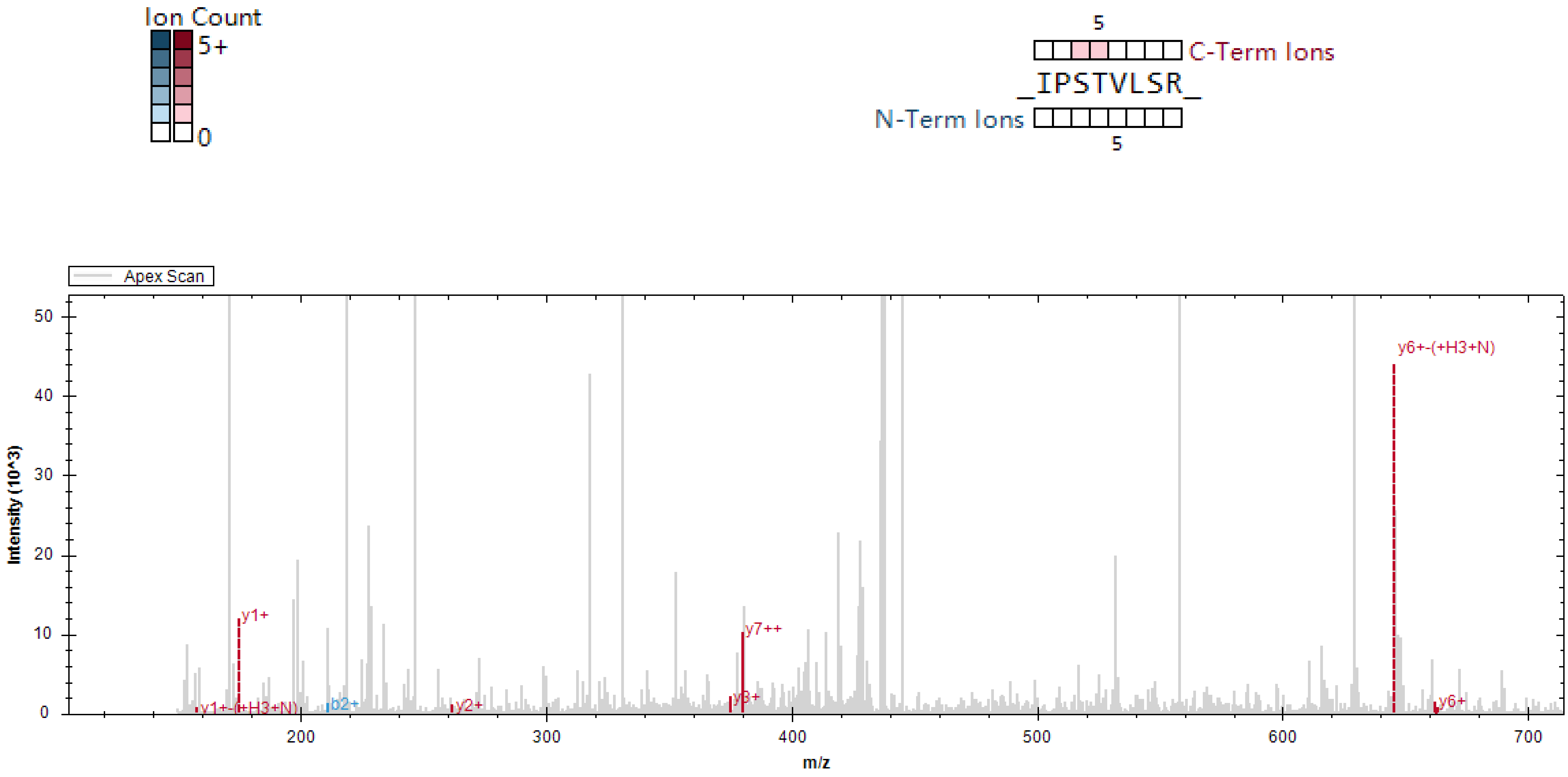

Ion Count
5+
0
5
C-Term Ions
_IPSTVLSR_
N-Term Ions
5
Apex Scan
y6+-(+H3+N)
y1+
y7++
y1+-(+H3+N)
b2+
y2+
y3+
y6+
Intensity (10^3)
m/z

Q9UEW3

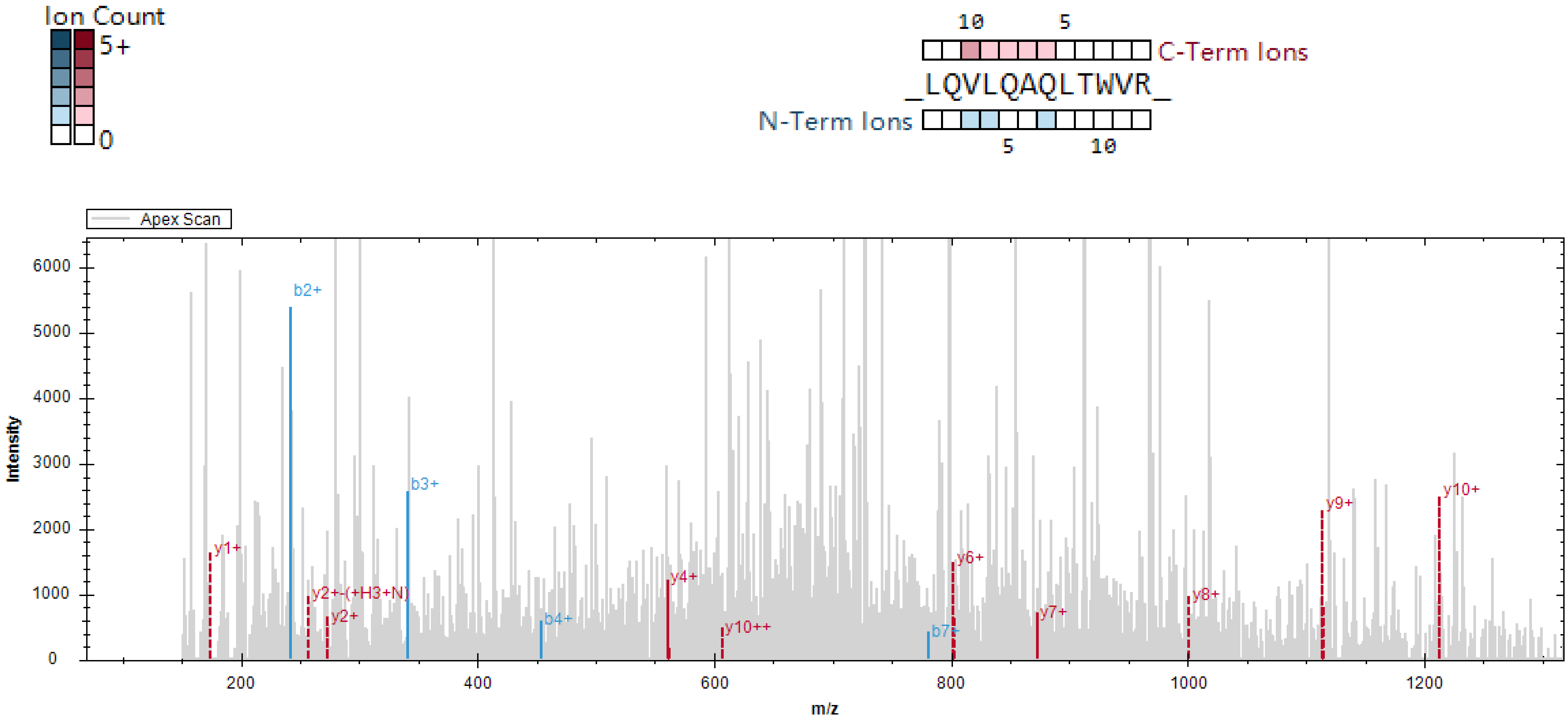

Ion Count
5+
0
10
5
C-Term Ions
_LQVLQAQLTWVR_
N-Term Ions
5
10
Apex Scan
Intensity
m/z
b2+
b3+
b4+
b7+
y1+
y2+-(+H3+N)
y2+
y4+
y10++
y6+
y7+
y8+
y9+
y10+

Q9UHX3

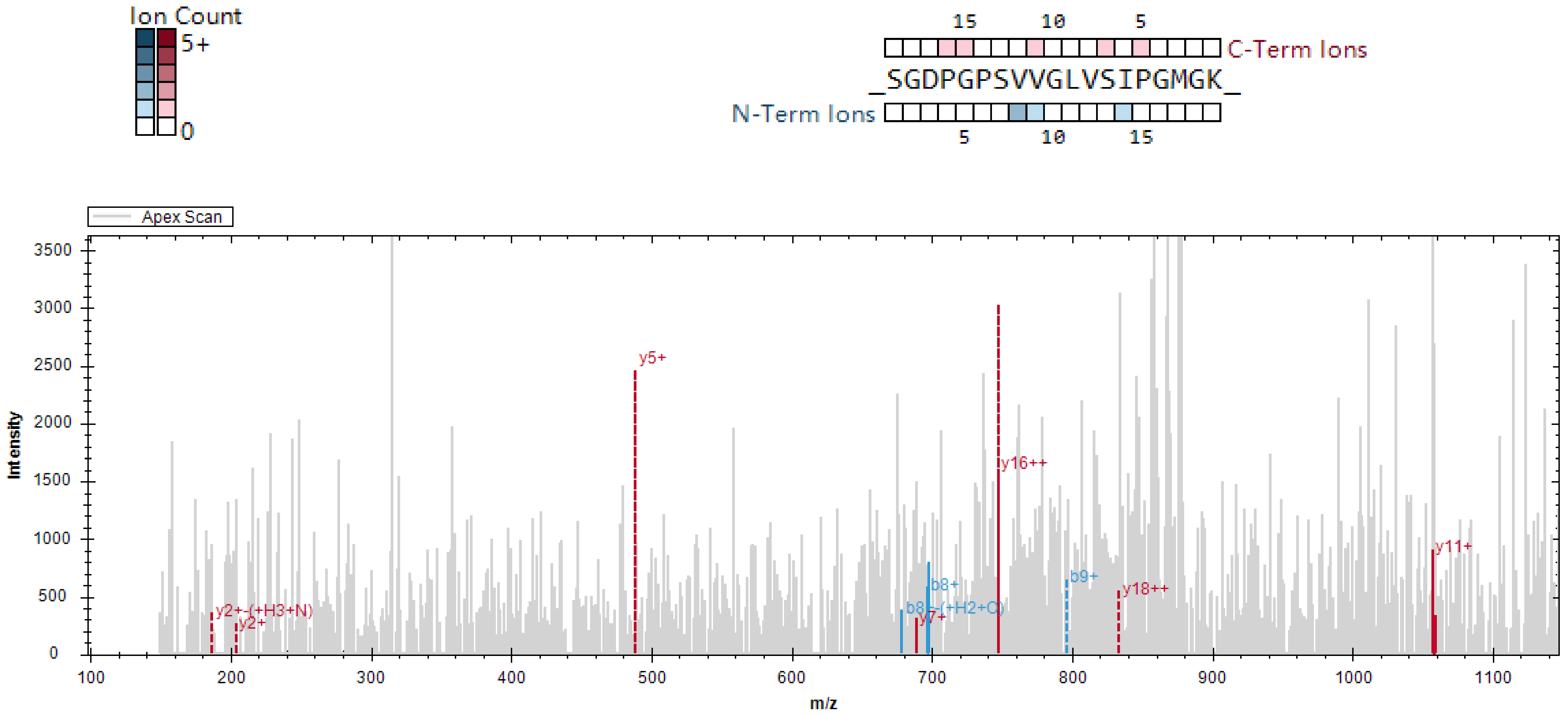

Ion Count
5+
0
15
10
5
C-Term Ions
_SGDPGPSVVGLVSIPGMGK_
N-Term Ions
5
10
15
Apex Scan
Intensity
m/z
y5+
y16++
y11+
b8+
b9+
y18++
b8+-(+H2+O)
y7+
y2+-(+H3+N)
y2+

Q9UI14

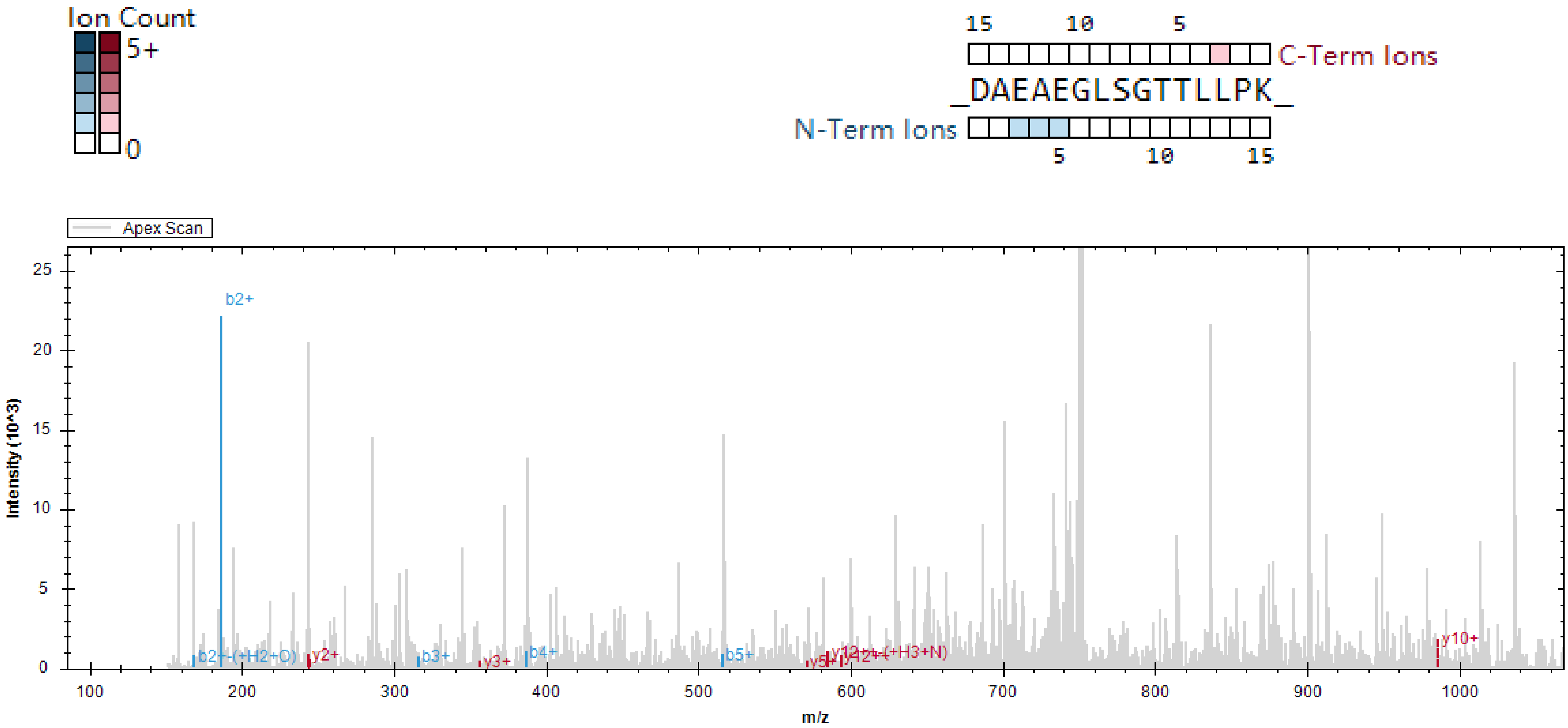

Ion Count
5+
0
15 10 5
C-Term Ions
_DAEAEGLSGTTLLPK_
N-Term Ions
5 10 15
Apex Scan
Intensity (10^3)
m/z
b2+
y10+

Q9UJJ9

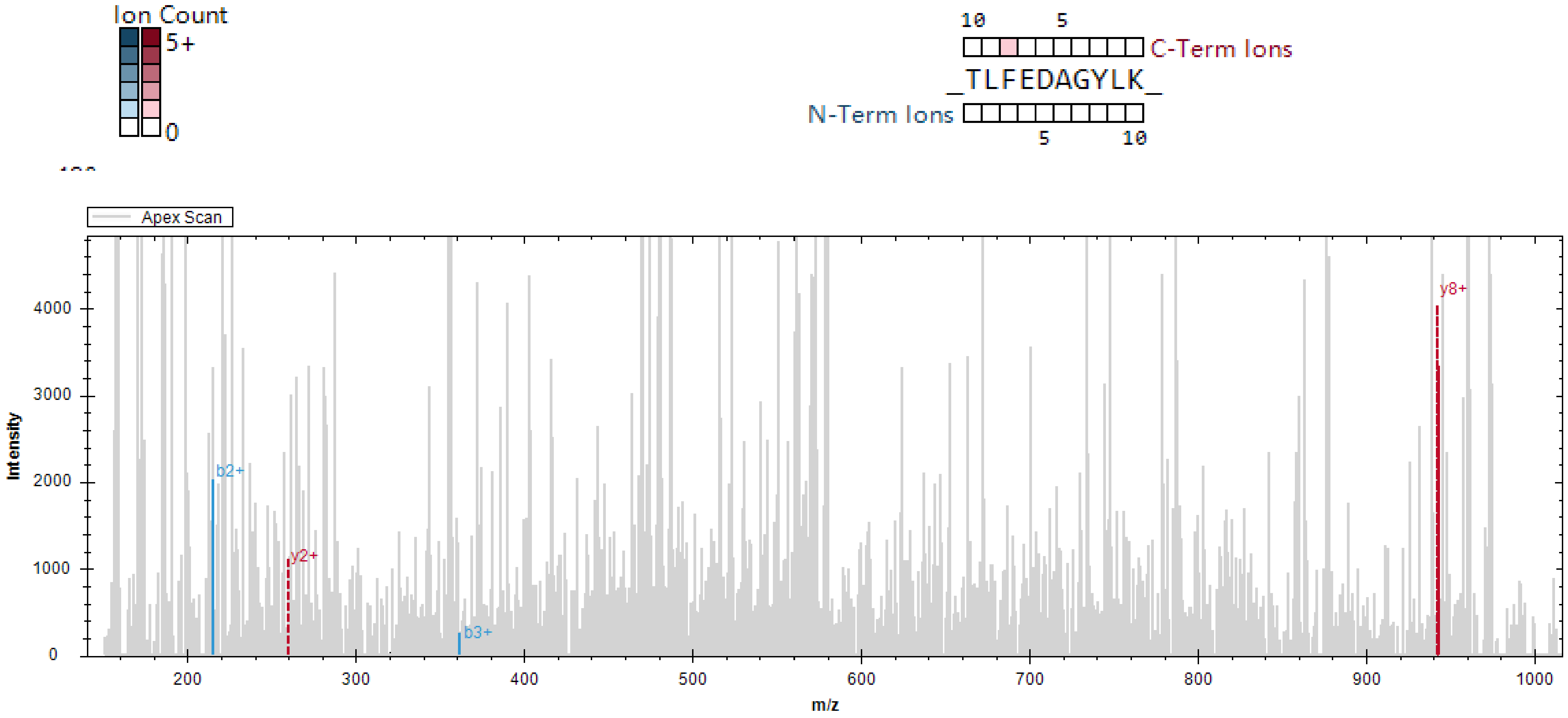
Ion Count
5+
0
10
5
C-Term Ions
_TLFEDAGYLK_
N-Term Ions
5
10
Apex Scan
y8+
b2+
y2+
b3+
Intensity
0
1000
2000
3000
4000
200
300
400
500
600
700
800
900
1000
m/z

# Q9UKA9

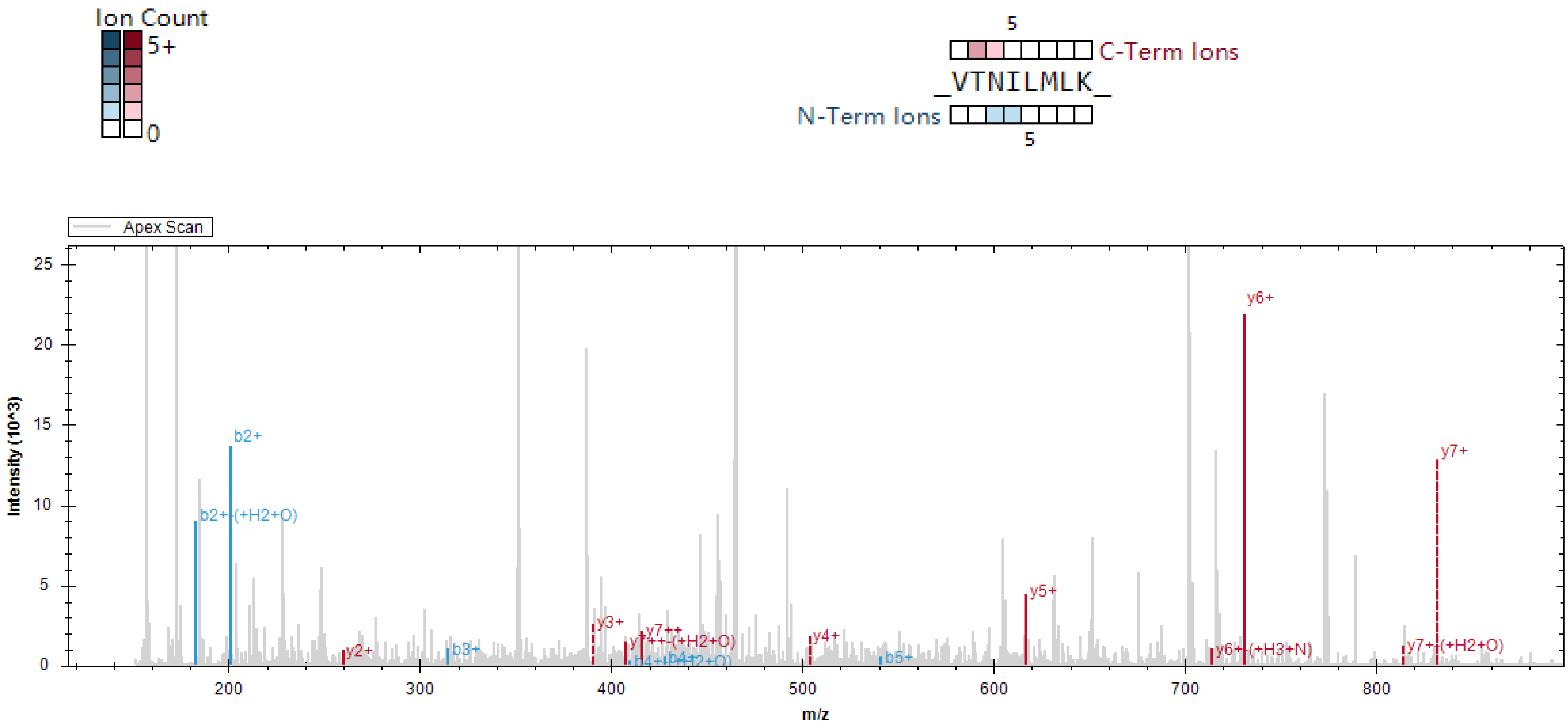

Ion Count
5+
0
5
C-Term Ions
_VTNILMLK_
N-Term Ions
5
Apex Scan
Intensity (10^3)
m/z
b2+
b2+-(+H2+O)
b3+
b5+
y2+
y3+
y4+
y5+
y6+
y7+
y6+-(+H3+N)
y7+-(+H2+O)

Q9UKJ5

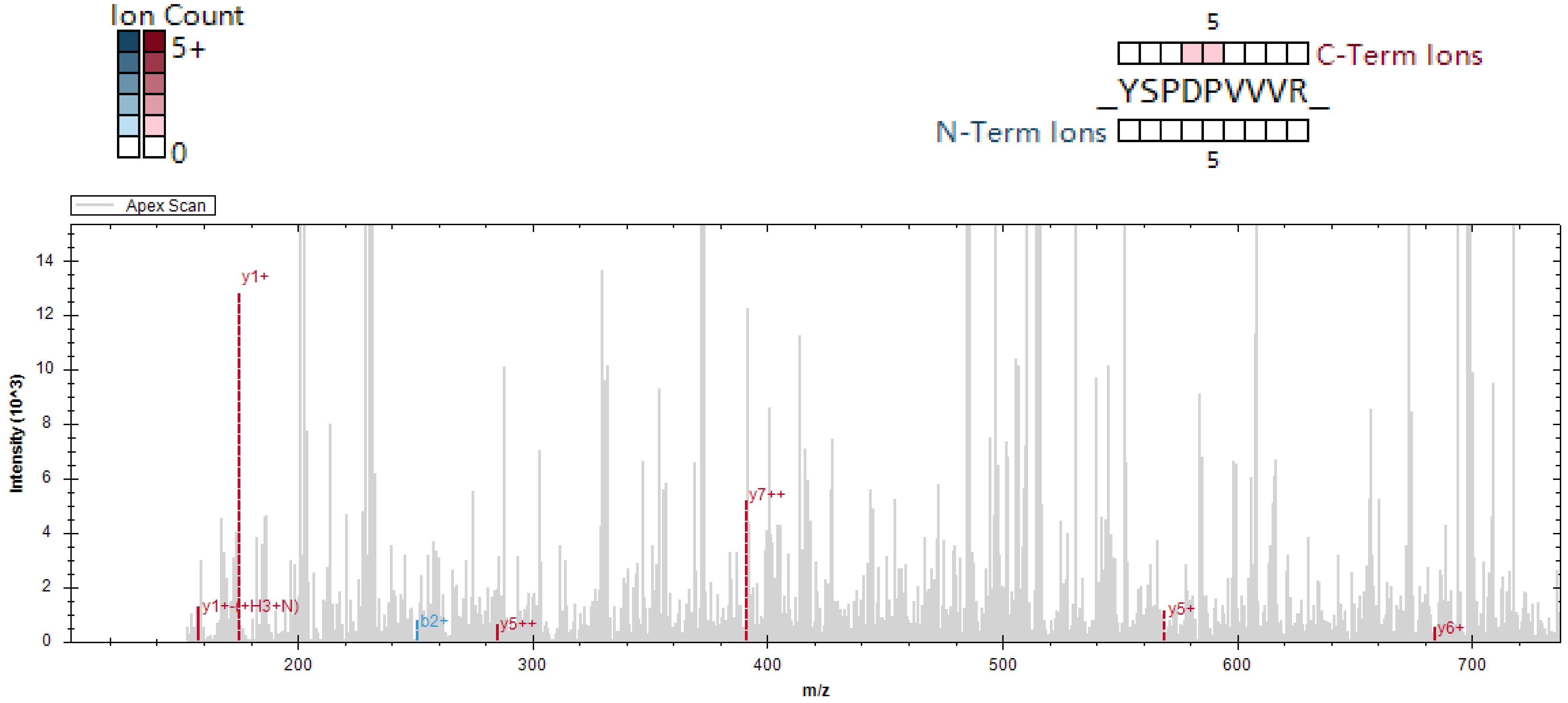

Ion Count
5+
0
5
C-Term Ions
_YSPDPVVVR_
N-Term Ions
5
Apex Scan
Intensity (10^3)
m/z
y1+
y1+-(+H3+N)
b2+
y5++
y7++
y5+
y6+

Q9UL16

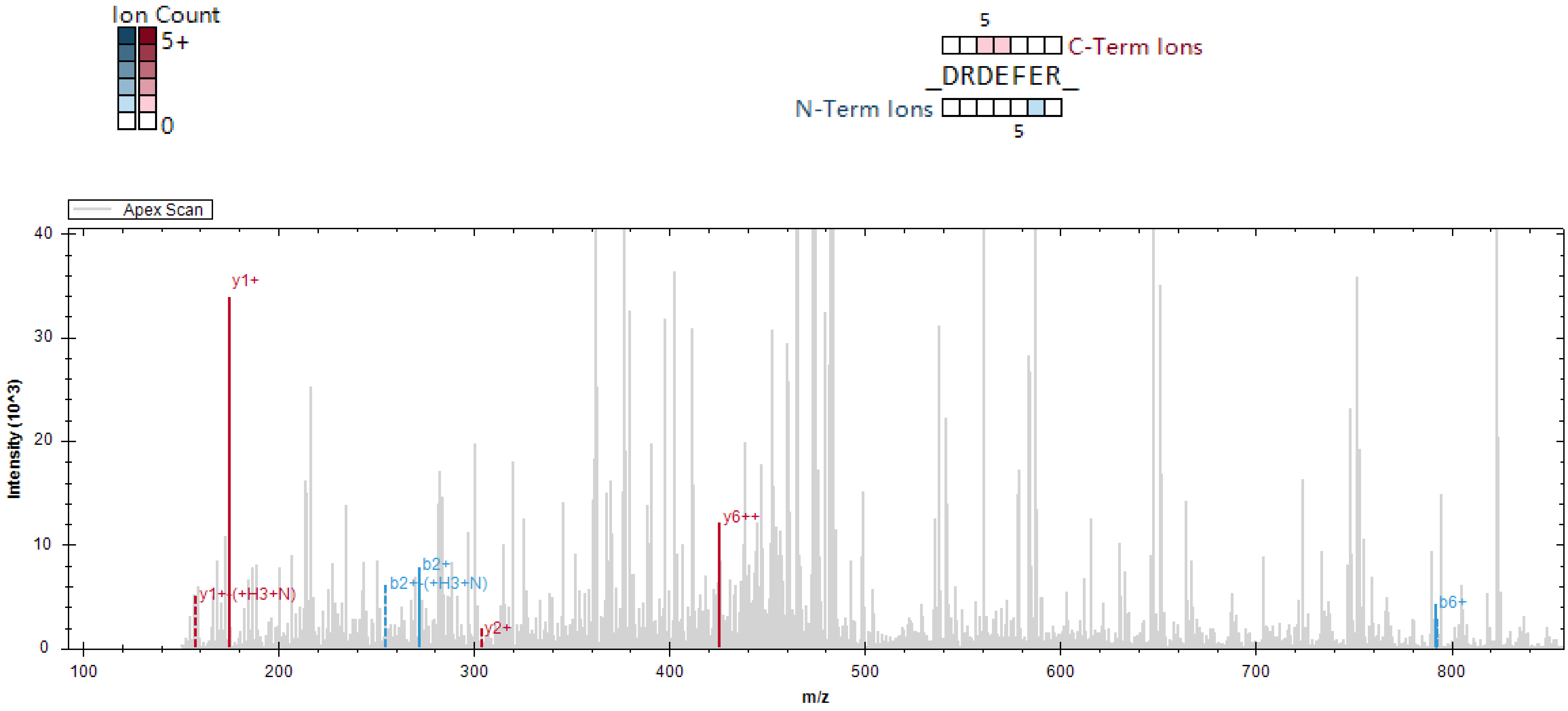

Ion Count
5+
0
5
C-Term Ions
_DRDEFER_
N-Term Ions
5
Apex Scan
y1+
y1+-(+H3+N)
b2+
b2+-(+H3+N)
y2+
y6++
b6+
Intensity (10^3)
m/z

# Q9ULG6

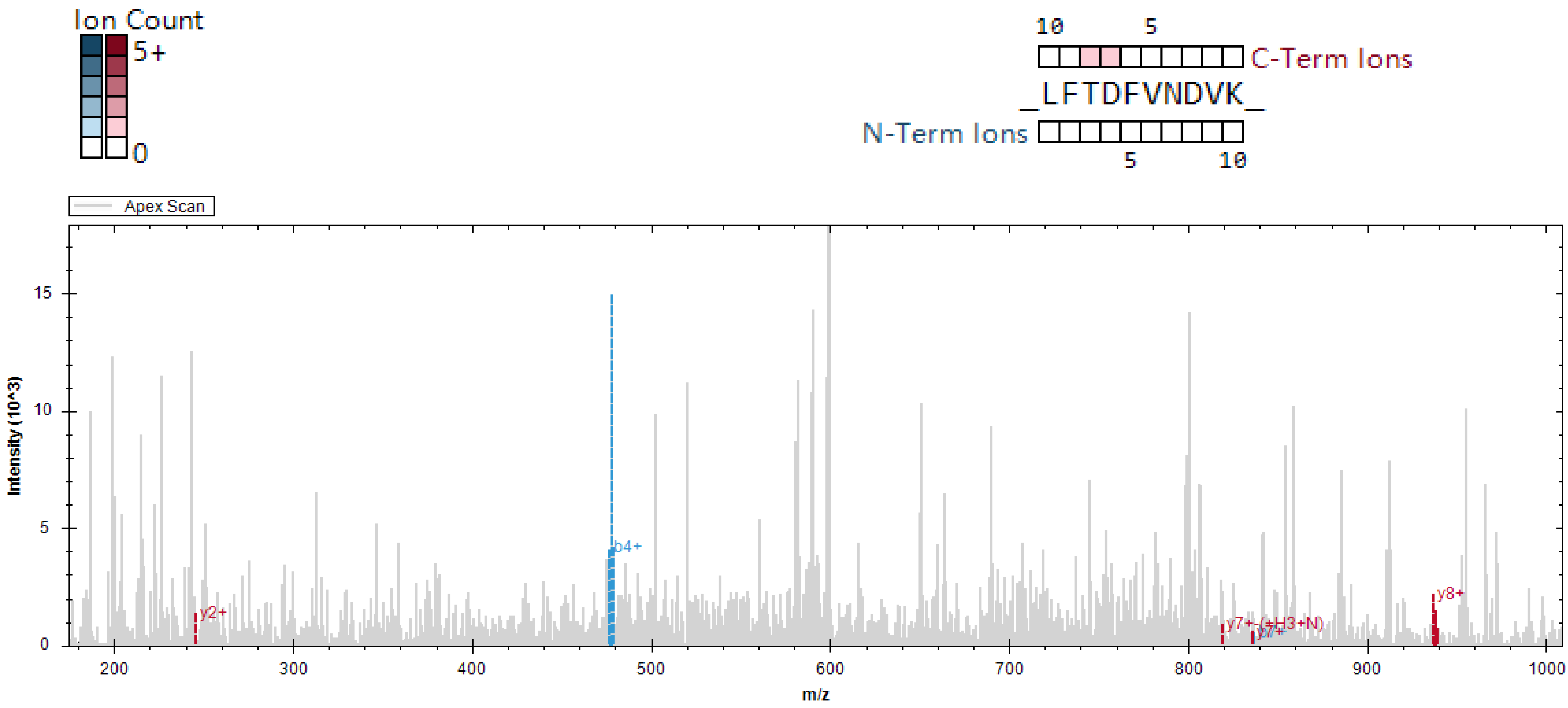

Ion Count
5+
0
10
5
C-Term Ions
_LFTDFVNDVK_
N-Term Ions
5
10
Apex Scan
b4+
y2+
y7+-(+H3+N)
y7+
y8+
Intensity (10^3)
m/z

Q9UP95

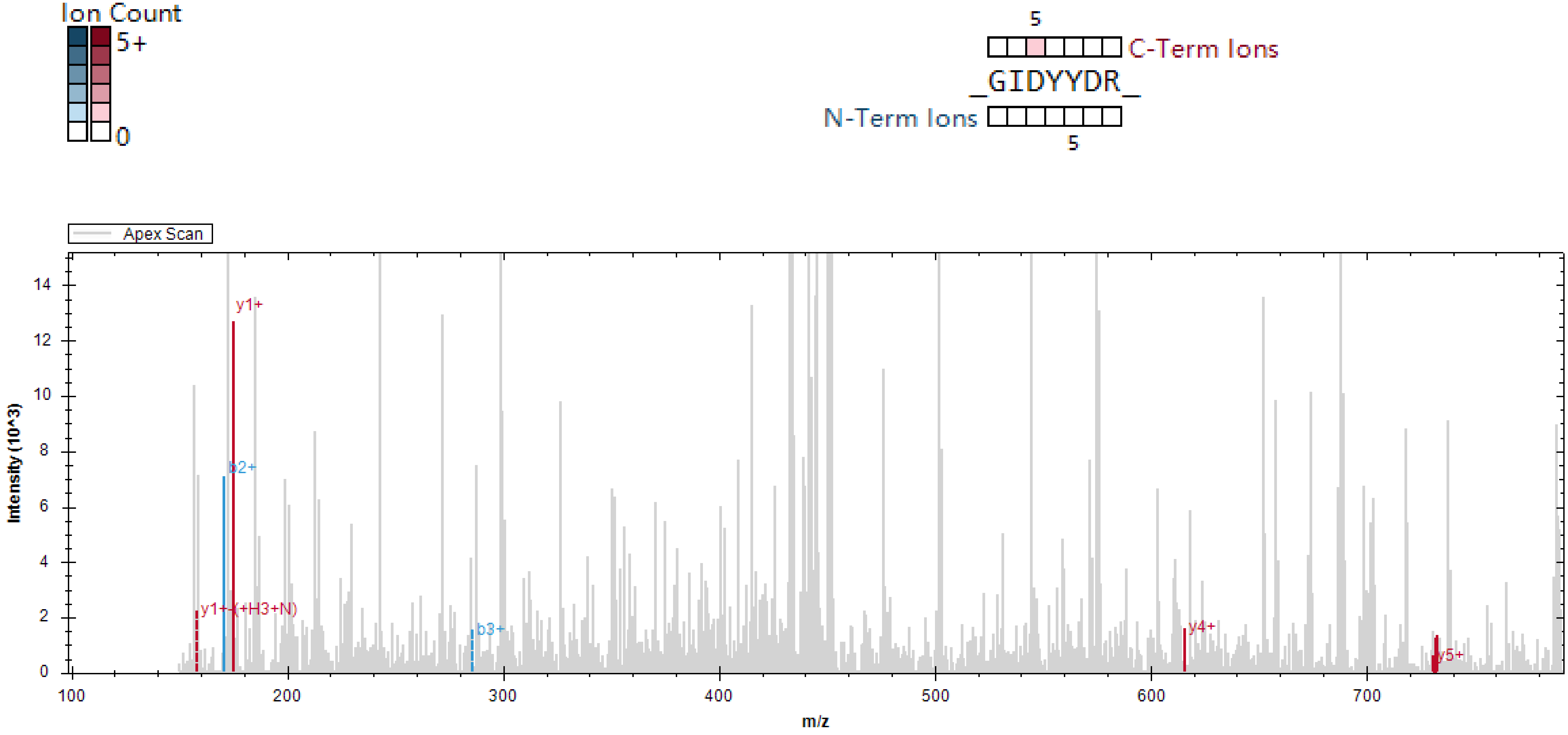

Ion Count
5+
0
5
C-Term Ions
_GIDYYDR_
N-Term Ions
5
Apex Scan
Intensity (10^3)
m/z
y1+
b2+
y1+-(+H3+N)
b3+
y4+
y5+

Q9Y228

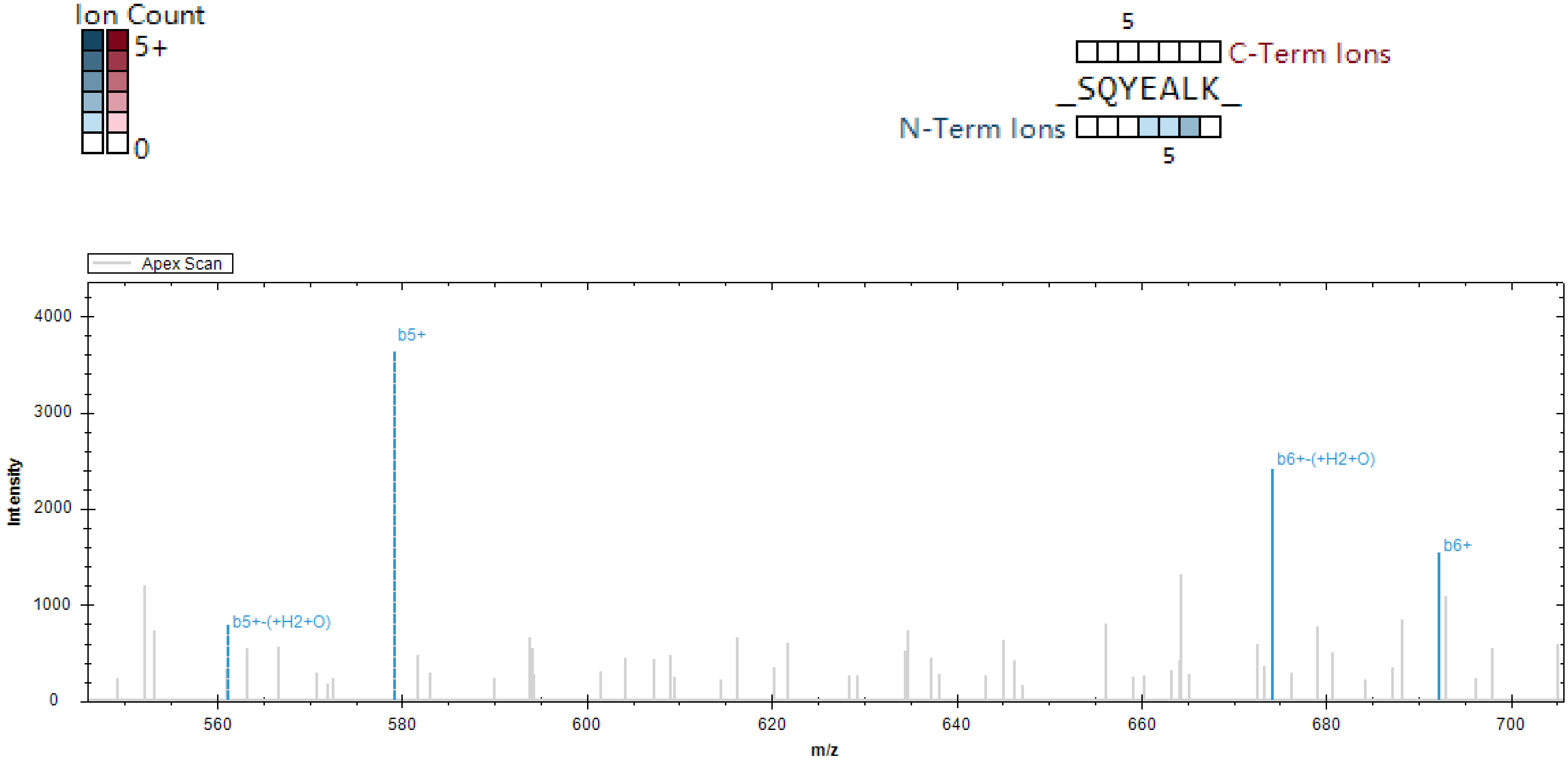

Ion Count
5+
0
5
C-Term Ions
_SQYEALK_
N-Term Ions
5
Apex Scan
4000
3000
2000
1000
0
Intensity
b5+
b5+-(+H2+O)
b6+-(+H2+O)
b6+
560
580
600
620
640
660
680
700
m/z

Q9Y241

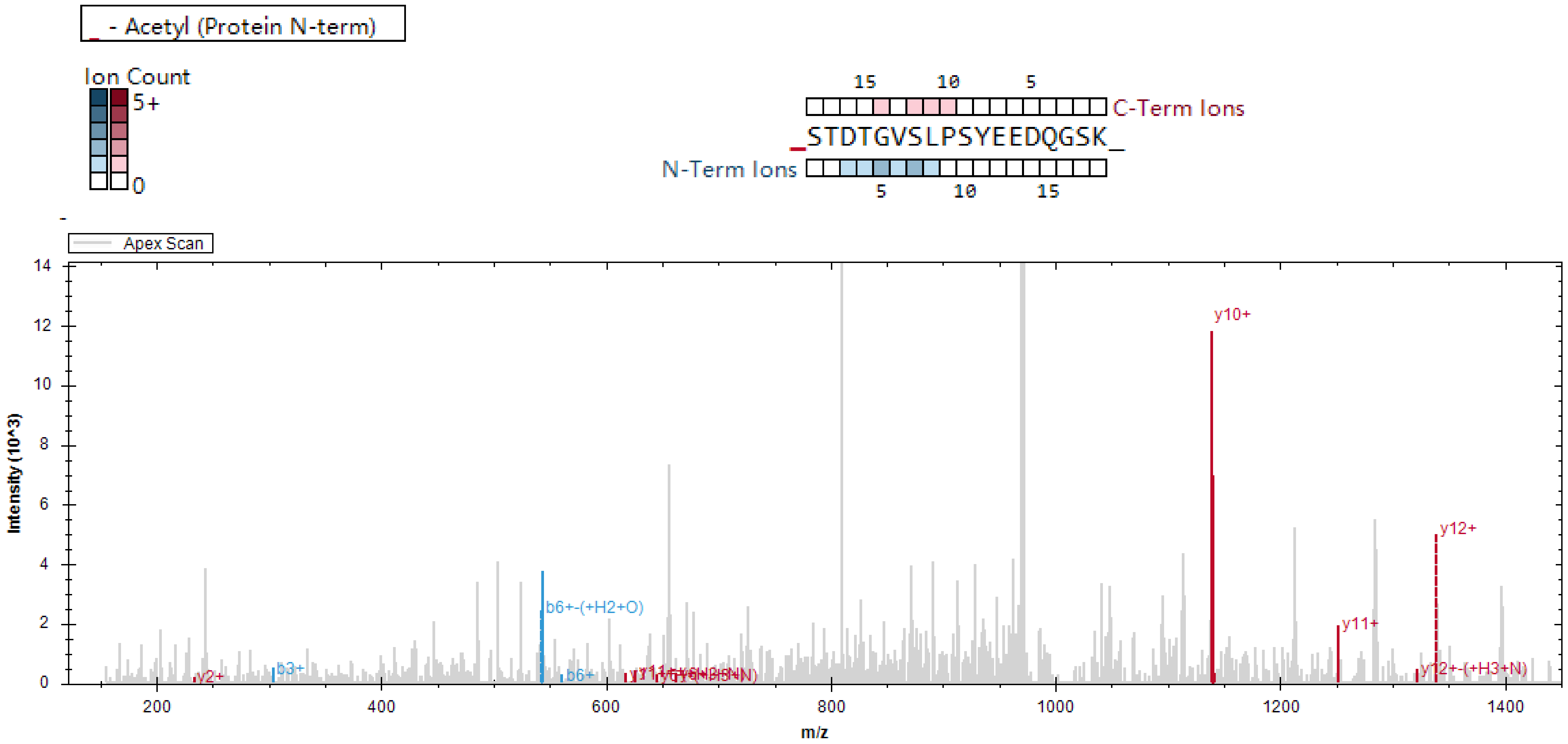

_ - Acetyl (Protein N-term)
Ion Count
5+
0
15 10 5
C-Term Ions
_STDTGVSLPSYEEDQGSK_
N-Term Ions
5 10 15
Apex Scan
Intensity (10^3)
m/z
y10+
y12+
y11+
b6+-(+H2+O)
b3+
y2+
b6+
y12+-(+H3+N)

Q9Y2E5

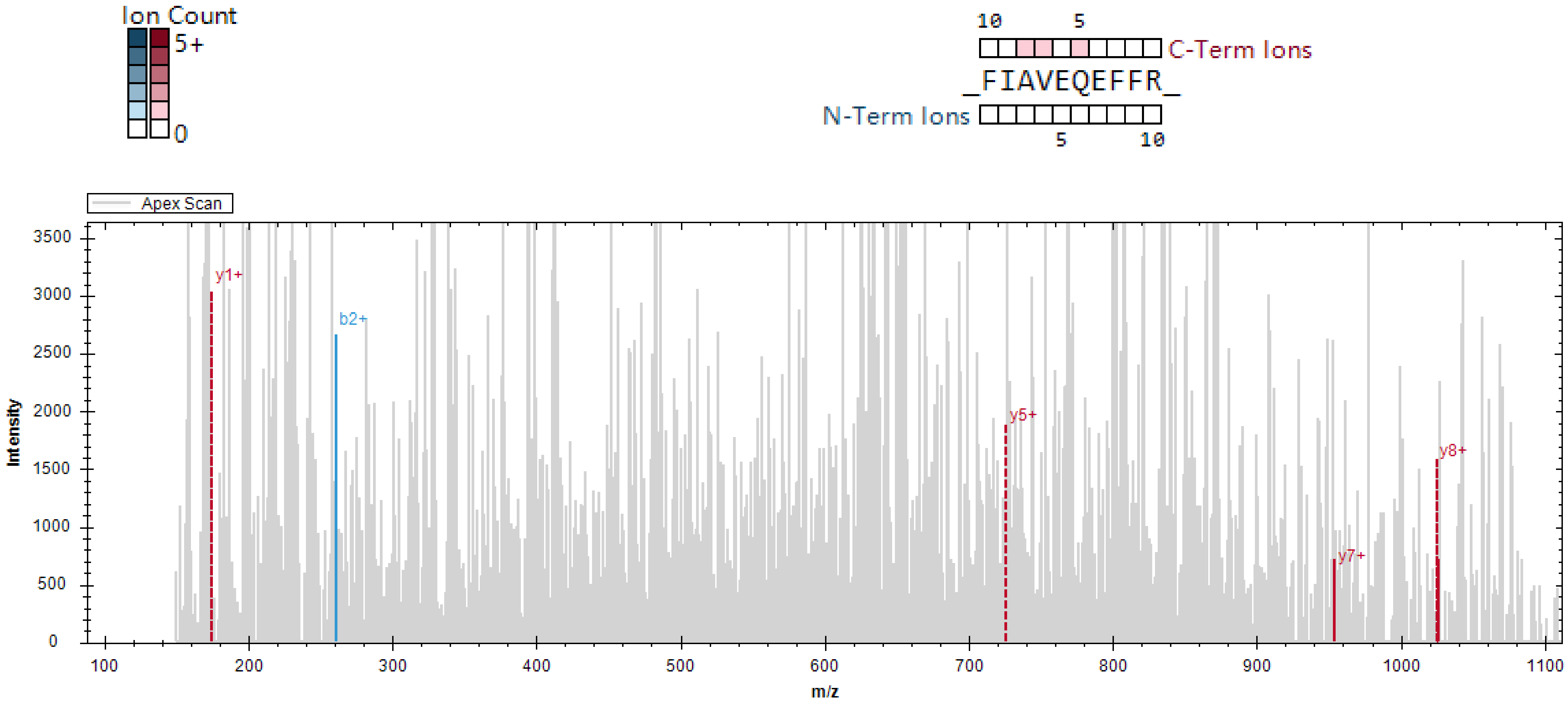
Ion Count
5+
0
10
5
C-Term Ions
_FIAVEQEFFR_
N-Term Ions
5
10
Apex Scan
Intensity
m/z
y1+
b2+
y5+
y7+
y8+
0
500
1000
1500
2000
2500
3000
3500
100
200
300
400
500
600
700
800
900
1000
1100

Q9Y2I9

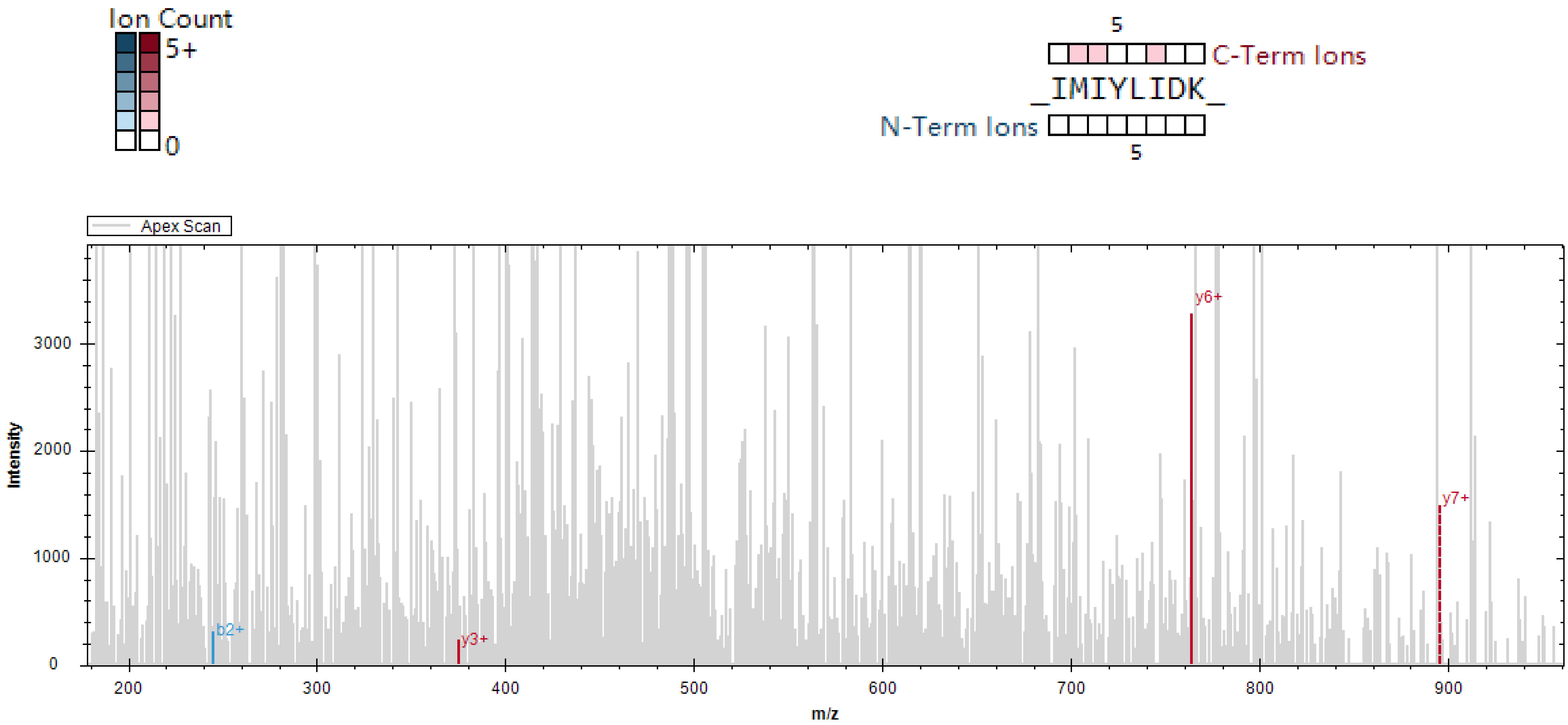
Ion Count
5+
0
5
C-Term Ions
_IMIYLIDK_
N-Term Ions
5
Apex Scan
y6+
y7+
b2+
y3+
Intensity
3000
2000
1000
0
200
300
400
500
600
700
800
900
m/z

Q9Y336

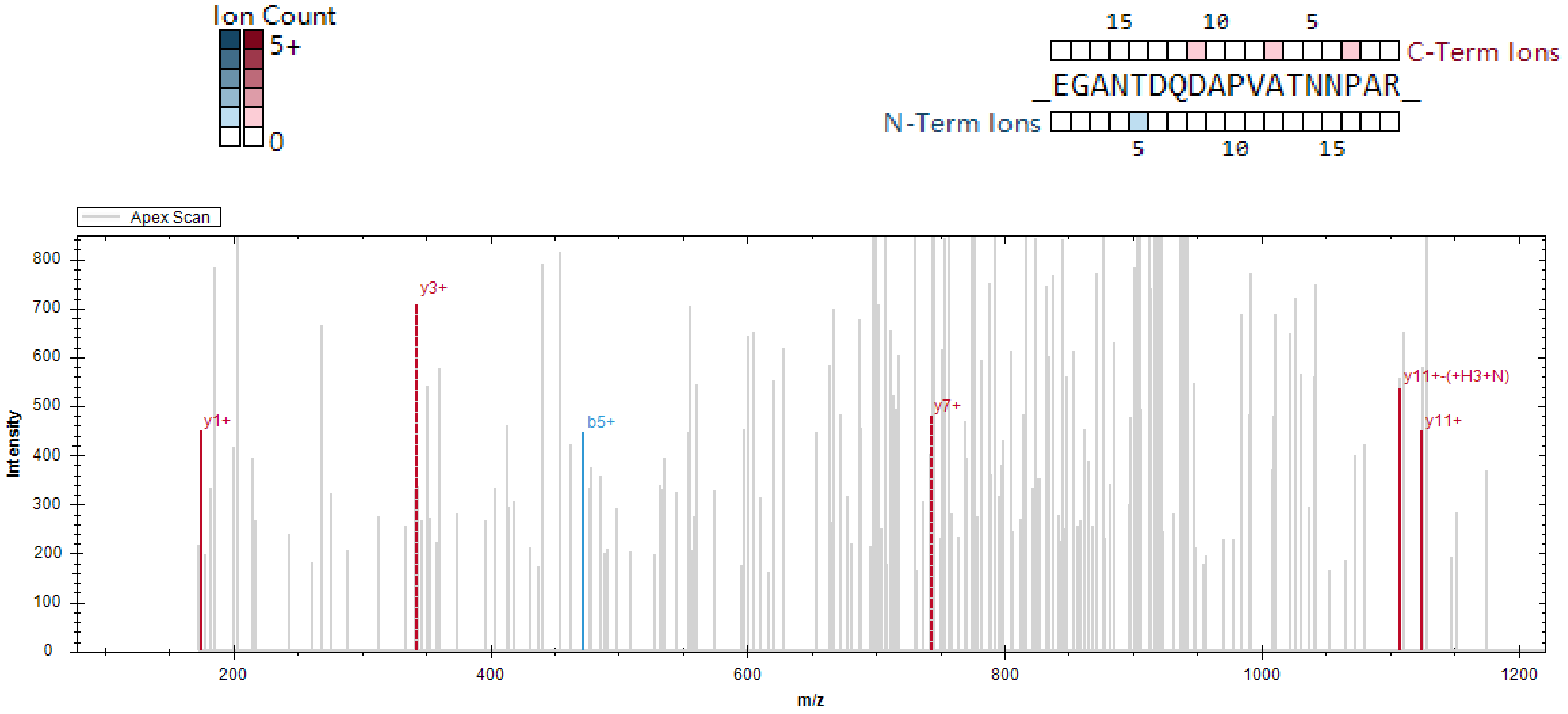

Ion Count
5+
0
15
10
5
C-Term Ions
_EGANTDQDAPVATNNPAR_
N-Term Ions
5
10
15
Apex Scan
Intensity
m/z
y1+
y3+
b5+
y7+
y11+-(+H3+N)
y11+

Q9Y4B5

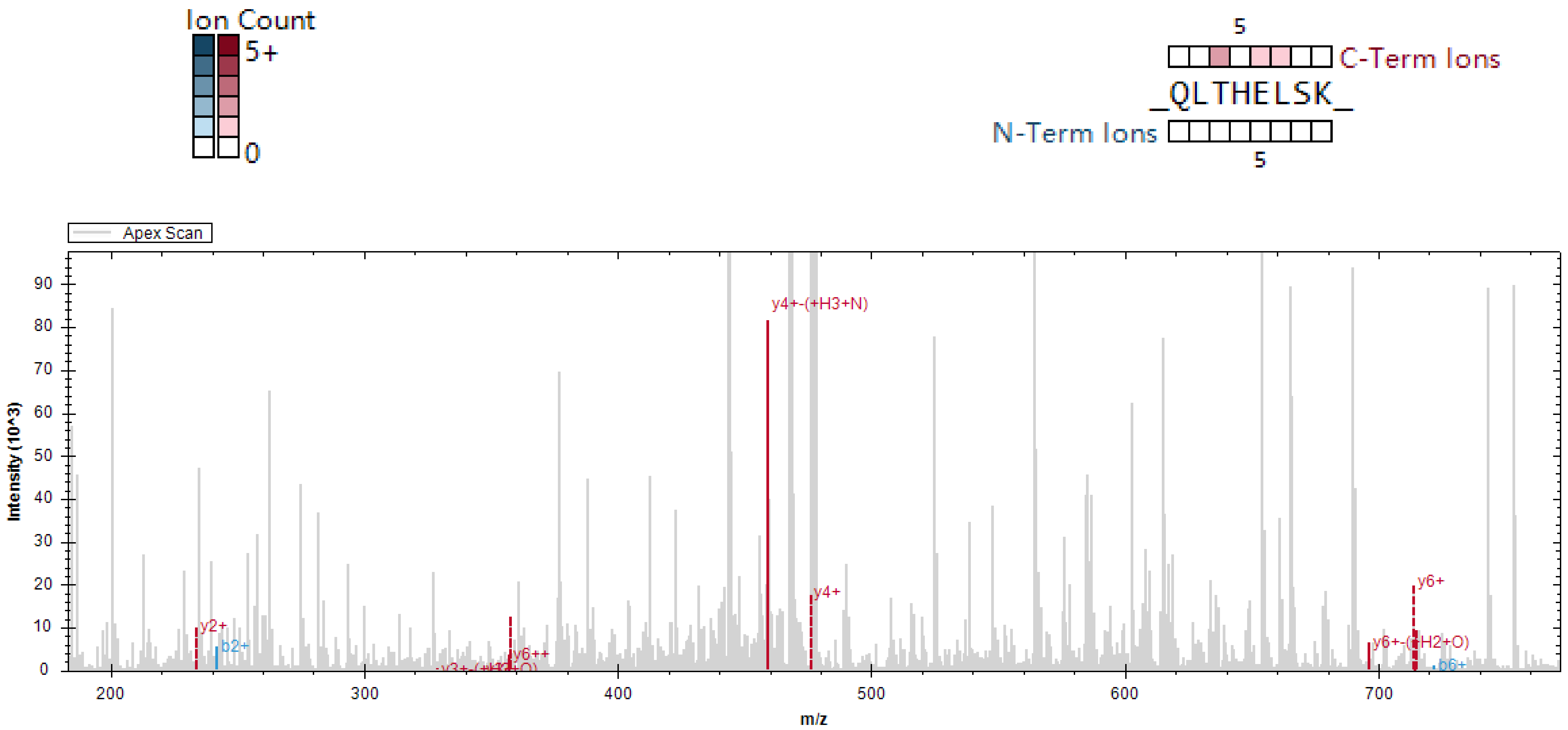

Ion Count
5+
0
5
C-Term Ions
_QLTHELSK_
N-Term Ions
5
Apex Scan
Intensity (10^3)
m/z
y4+-(+H3+N)
y4+
y2+
b2+
y6++
y6+
y6+-(+H2+O)
b6+

Q9Y4B6

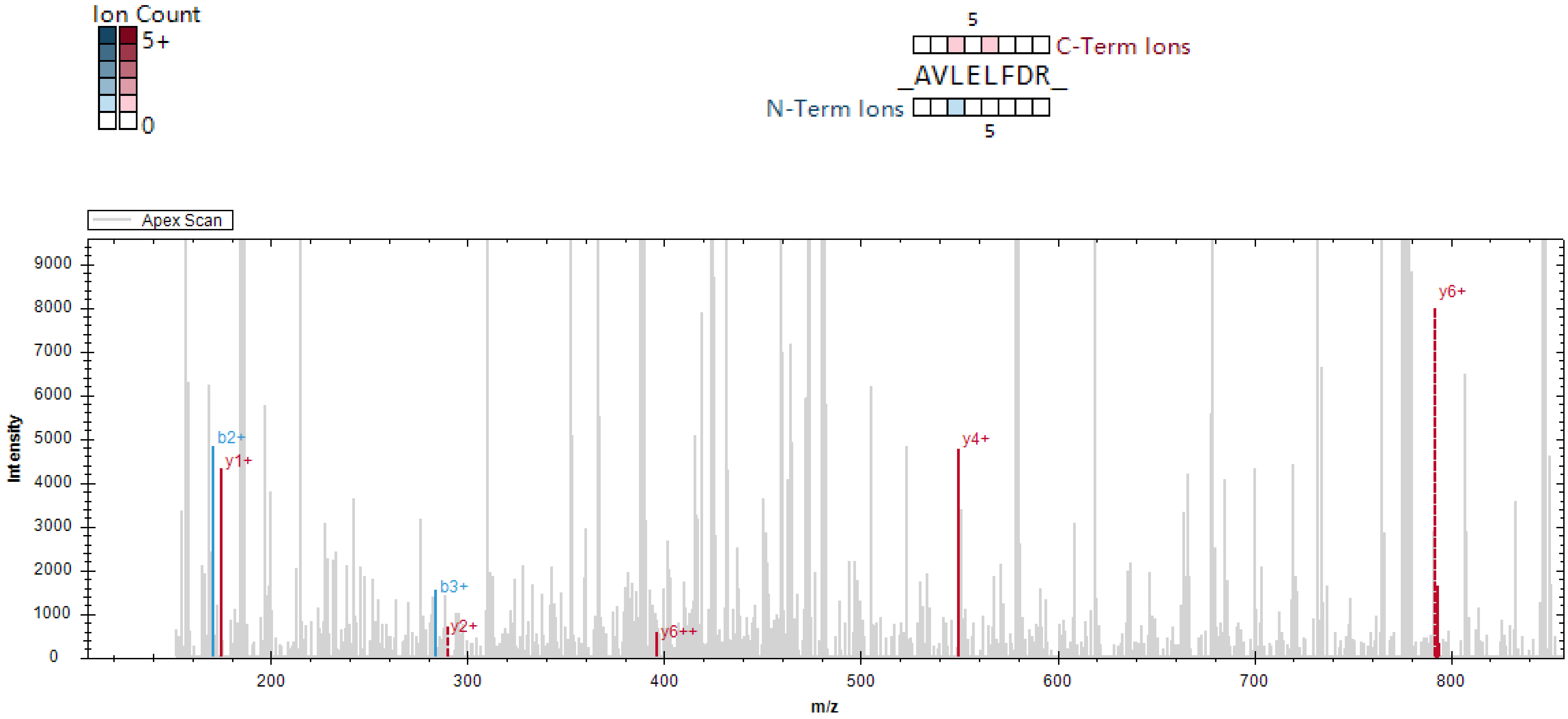

Ion Count
5+
0
5
C-Term Ions
_AVLELFDR_
N-Term Ions
5
Apex Scan
Intensity
m/z
b2+
y1+
b3+
y2+
y6++
y4+
y6+

Q9Y584

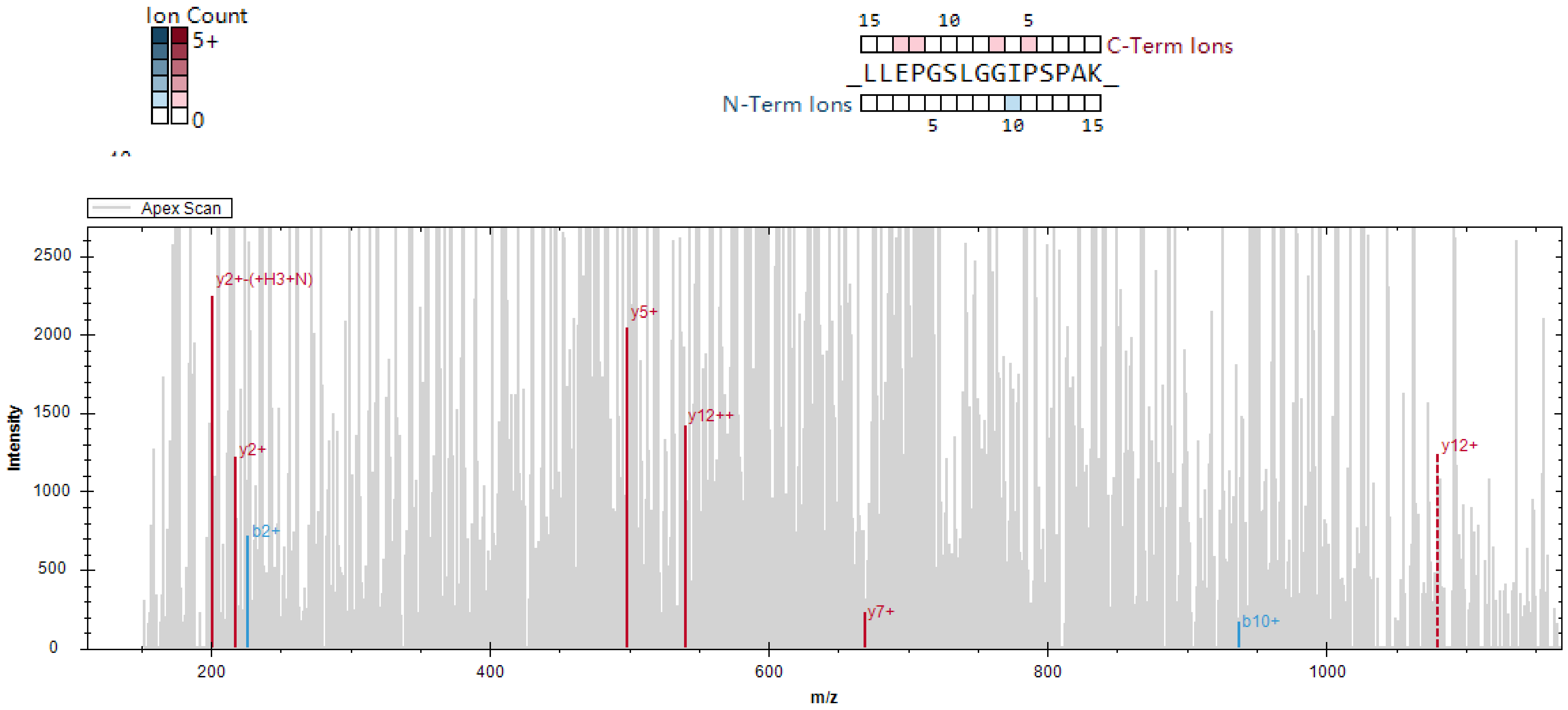

Ion Count
5+
0
15 10 5
C-Term Ions
_LLEPGSLGGIPSPAK_
N-Term Ions
5 10 15
Apex Scan
y2+-(+H3+N)
y5+
y12++
y2+
b2+
y12+
y7+
b10+
Intensity
m/z
0
500
1000
1500
2000
2500
200
400
600
800
1000

Q9Y587

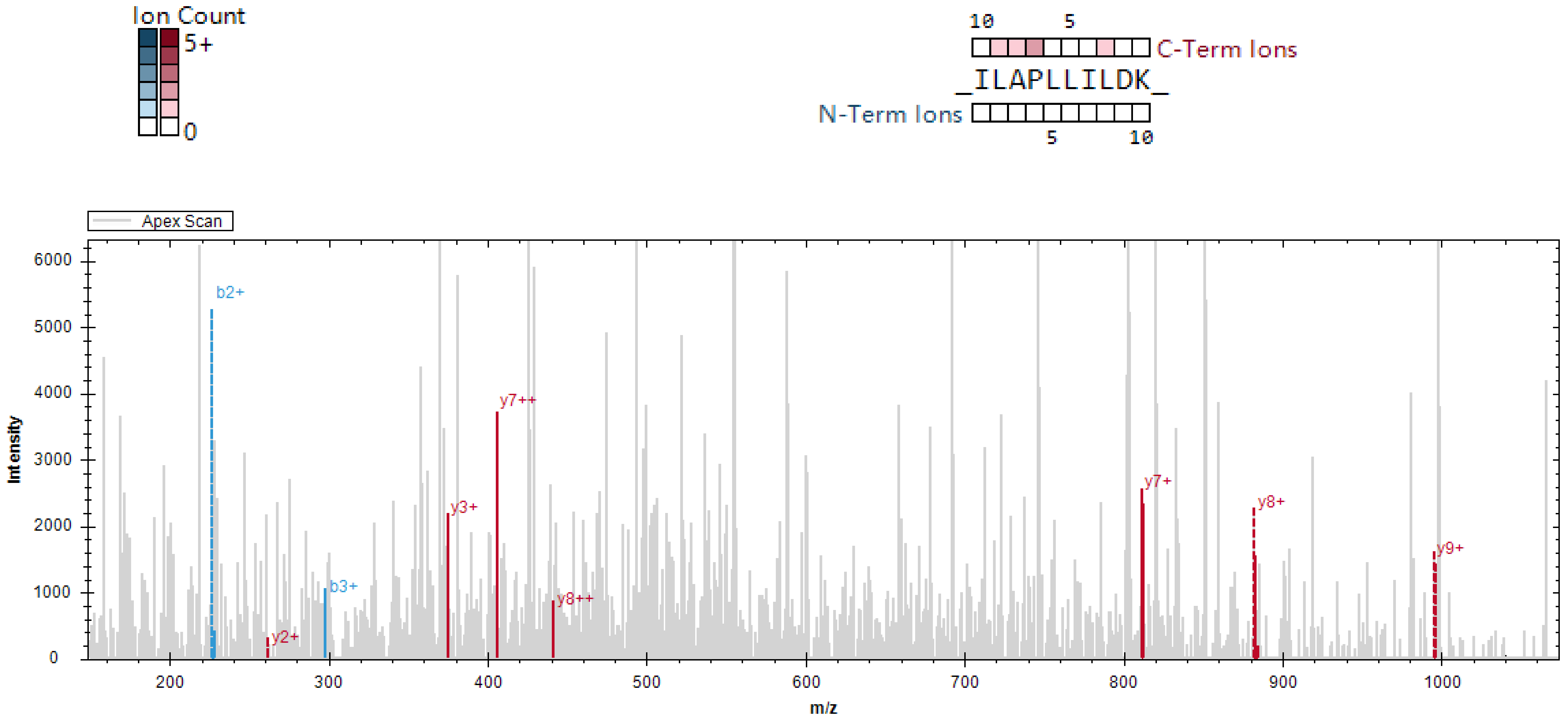

Ion Count
5+
0
10
5
C-Term Ions
_ILAPLLILDK_
N-Term Ions
5
10
Apex Scan
Intensity
m/z
b2+
b3+
y2+
y3+
y7++
y8++
y7+
y8+
y9+

Q9Y5G8

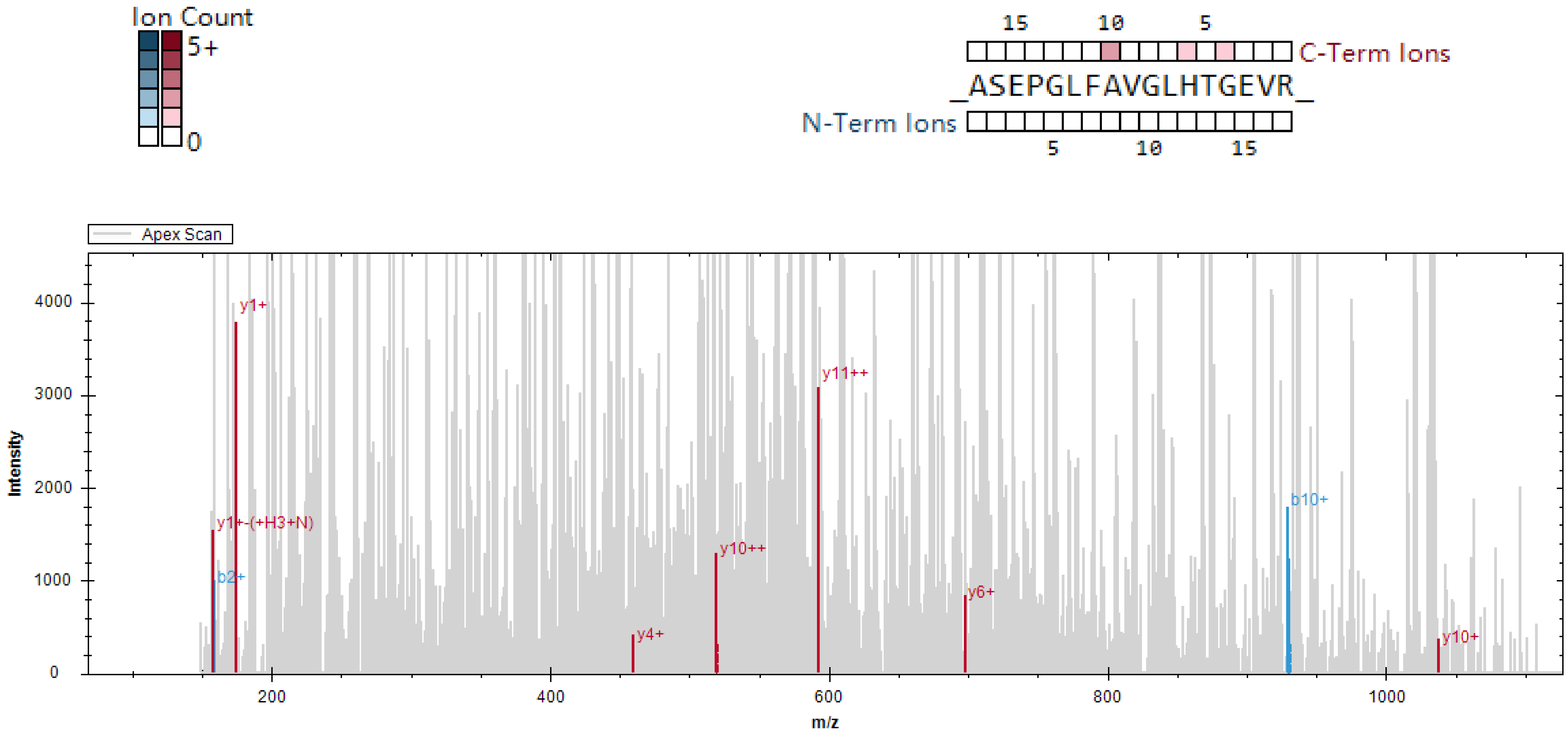
Ion Count
5+
0
15
10
5
C-Term Ions
_ASEPGLFAVGLHTGEVR_
N-Term Ions
5
10
15
Apex Scan
Intensity
m/z
0
1000
2000
3000
4000
200
400
600
800
1000
y1+
y1+-(+H3+N)
b2+
y4+
y10++
y11++
y6+
b10+
y10+

# Q9Y651

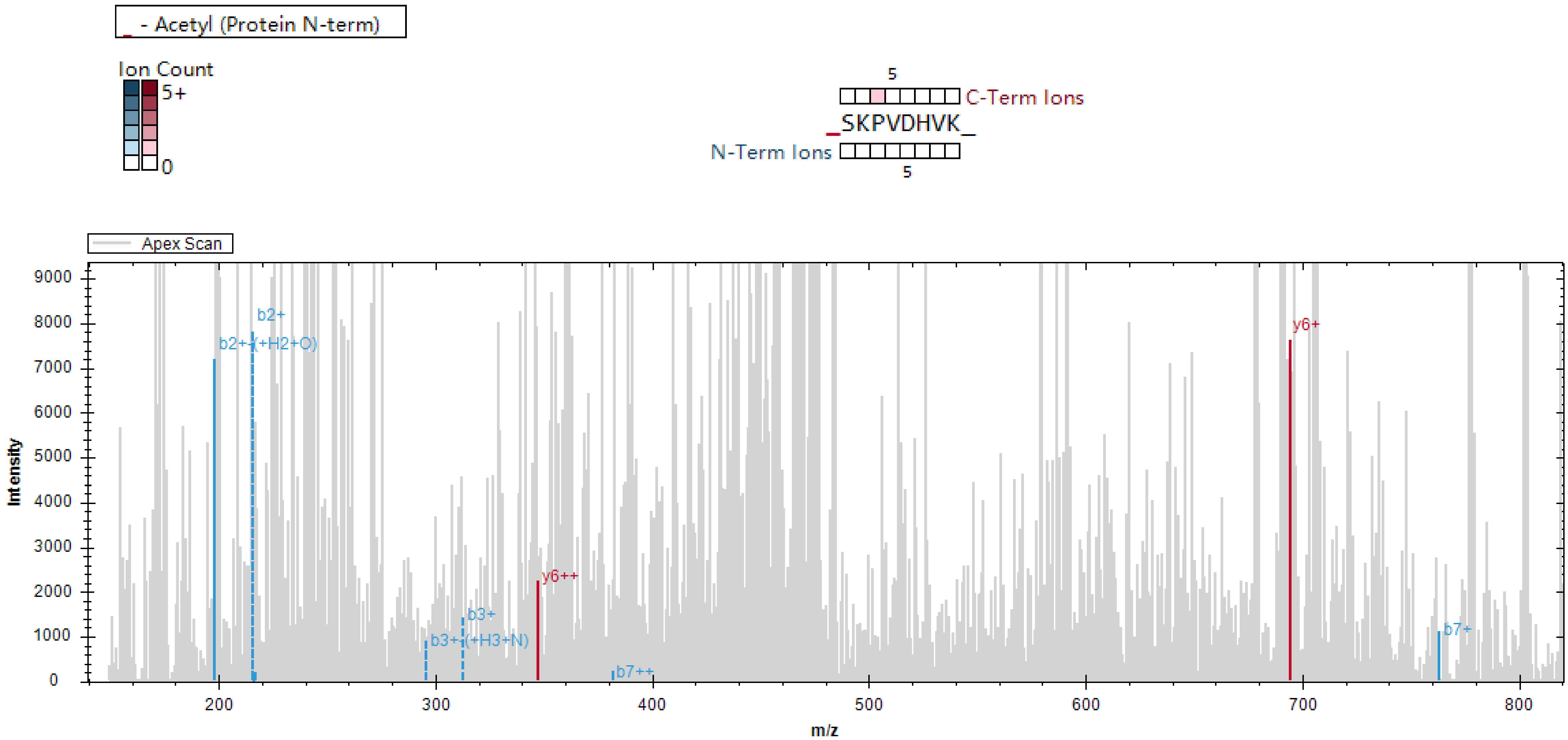

_ - Acetyl (Protein N-term)
Ion Count
5+
0
5
C-Term Ions
_SKPVDHVK_
N-Term Ions
5
Apex Scan
Intensity
m/z
b2+
b2++(+H2+O)
b3+
b3++(+H3+N)
y6++
b7++
y6+
b7+

Q9Y6T7

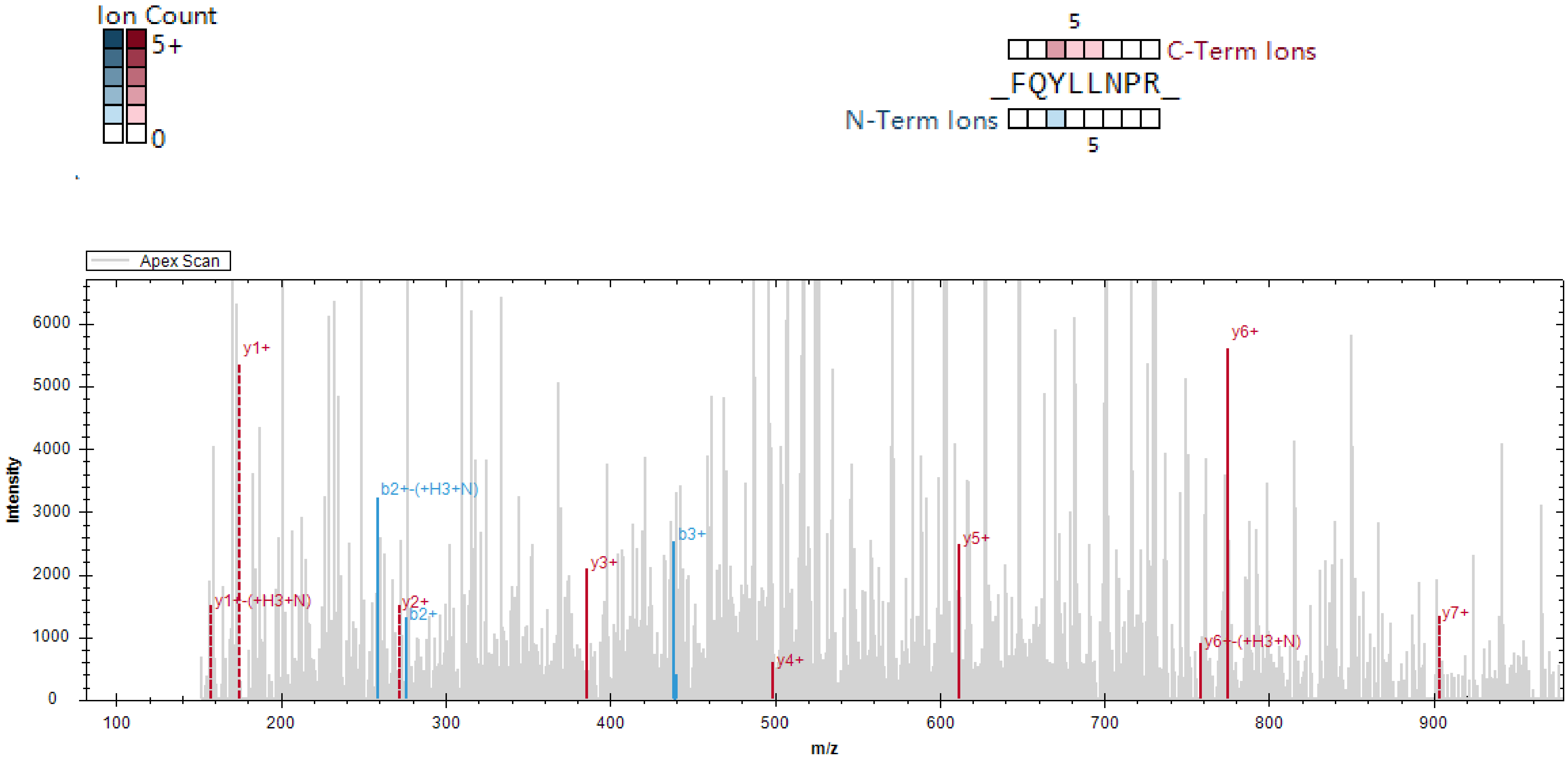

Ion Count
5+
0
5
C-Term Ions
_FQYLLNPR_
N-Term Ions
5
Apex Scan
Intensity
m/z
y1+
y1+-(+H3+N)
b2+-(+H3+N)
y2+
b2+
y3+
b3+
y4+
y5+
y6+
y6+-(+H3+N)
y7+
